# Immunoinformatic prediction of the pathogenicity of bovine viral diarrhea virus genotypes: implications for viral virulence determinants, designing novel diagnostic assays and vaccines development

**DOI:** 10.3389/fvets.2023.1130147

**Published:** 2023-07-06

**Authors:** Anwar A. G. Al-Kubati, Mahmoud Kandeel, Jamal Hussen, Maged Gomaa Hemida, Abdullah I. A. Al-Mubarak

**Affiliations:** ^1^Department of Veterinary Medicine, Faculty of Agriculture and Veterinary Medicine, Thamar University, Thamar, Yemen; ^2^Department of Biomedical Sciences, College of Veterinary Medicine, King Faisal University, Al-Hofuf, Saudi Arabia; ^3^Department of Pharmacology, Faculty of Veterinary Medicine, Kafrelsheikh University, Kafrelsheikh, Egypt; ^4^Department of Microbiology, College of Veterinary Medicine, King Faisal University, Al-Hofuf, Saudi Arabia; ^5^Department of Veterinary Biomedical Sciences, College of Veterinary Medicine, Long Island University, New York, NY, United States

**Keywords:** bovine viral diarrhea virus (BVDV), immunoinformatic, vaccines, diagnostics, virulence

## Abstract

**Introduction:**

Bovine viral diarrhea virus (BVDV) significantly impacts the bovine industries, both dairy and beef sectors. BVDV can infect various domestic and wild animals, most notably cattle. The dynamic variations among BVDV serotypes due to the continuous genetic diversity, especially in BVDV1 (BVDV1), reduce the effectiveness of the currently available vaccines and reduce the specificity/sensitivity of the diagnostic assays. The development of novel, safe, and effective vaccines against BVDV requires deep knowledge of the antigenicity and virulence of the virus. Previous studies on the antigenicity and the virulence of BVDV serotypes have been mainly focused on one or a few BVDV proteins. While however, little is known about the orchestration of all BVDV in the context of viral virulence and immunogenicity. The main aim of the current study was to do a comparative computational evaluation of the immunogenicity, and virulence for all the encoded proteins of both BVDV1 and BVDV2 and their sub-genotypes.

**Methods:**

To achieve this goal, 11,737 protein sequences were retrieved from Virus Pathogen Resource. The analysis involved a total of 4,583 sequences after the removal of short sequences and those with unknown collection time. We used the MP3 tool to map the pathogenic proteins across different BVDV strains. The potential protective and the epitope motifs were predicted using the VaxiJen and EMBOSS antigen tools, respectively.

**Results and discussion:**

The virulence prediction revealed that the NS4B proteins of both BVDV1 and BVDV2 likely have essential roles in BVDV virulence. Similarly, both the capsid (C) and the NS4-A proteins of BVDV1 and the N^pro^ and P7 proteins of BVDV2 are likely important virulent factors. There was a clear trend of increasing predicted virulence with the progression of time in the case of BVDV1 proteins, but that was not the case for the BVDV2 proteins. Most of the proteins of the two BVDV serotypes possess antigens predicted immunogens except N^pro^, P7, and NS4B. However, the predicted antigenicity of the BVDV1 was significantly higher than that of BVDV2. Meanwhile, the predicted immunogenicity of the immunodominant-E2 protein has been decreasing over time. Based on our predicted antigenicity and pathogenicity studies of the two BVDV serotypes, the sub-genotypes (1a, 1f, 1k, 2a, and 2b) may represent ideal candidates for the development of future vaccines against BVDV infection in cattle. In summary, we identified some common differences between the two BVDV genotypes (BVDV1 and BVDV2) and their sub-genotypes regarding their protein antigenicity and pathogenicity. The data presented here will increase our understanding of the molecular pathogenesis of BVDV infection in cattle. It will also pave the way for developing some novel diagnostic assays and novel vaccines against BVDV in the near future.

## 1. Introduction

Bovine viral diarrhea (BVD) is a transboundary virus that affects farm animals, especially cattle, as well as several wild animals worldwide. Bovine viral diarrhea virus (BVDV) causes high economic losses due to high morbidity, mortality, reproductive failure, abortion, stillbirth, congenital malformation of the newborn animals, decrease in milk, and poor weight gains. Additionally, indirect losses results from control and preventive measures (1). To avoid these losses, several countries have implemented an eradication strategies. The meta-analysis of the BVDV surveillance studies showed that the frequency of BVDV-persistent infected (PI) animals decreased in Europe while increasing in North America (2). This was attributed to the genetic and antigenic diversity of the BVDV (3, 4).

Bovine viral diarrhea virus has several serotypes, genotypes (BVDV1 and 2), subgenotypes and biotypes [cytopathic (CP-BVDV) and non-cytopathic (NCP-BVDV)] (5). BVDV belongs to the genus Pestivirus in the family *Flaviviridae* (6). It has a small single-stranded positive-sense RNA genome that contains a single ORF encoding a single long polyprotein. The viral polyprotein is co-and post-translationally cleaved by viral and cellular proteases to produce four structural and eight nonstructural (NS) proteins including (Npro [p20], Capsid [p14], Erns, E1 [gp25], E2 [gp53], P7, NS2 [p54], NS3 [p80], NS4A [p10], NS4B [p30], NS5A [p58] and NS5B [p75]) (7).

Mass vaccination of cattle against BVDV across North America with coverage of up to 80% of the cattle population contributed substantially to the control of BVDV infection in various cattle herds in North America. Both E2 and NS2-3 proteins of the BVDV have been reported to be the main immunogenic proteins and were used to develop mosaic vaccines in an attempt to overcome the poor performance of some developed vaccines (4, 8).

Genetic diversity also affects the biological and pathological characters of the BVDV infection in cattle. For instance, recombination with host RNA may occur and derives a shift in a biotype from NCP-BVDV to CP-BVDV (9, 10), a change that may also be linked to the variations in the viral virulence (8). This shift from NCP-BVDV to CP-BVDV was linked to the cleavage of NS2-3 protein by autoprotease into NS2 and NS3, the latter is an essential component of the replicase. This process is downregulated a few hours after the infection with NCP-BVDV biotype while continuing unrestrictedly in the case of CP-BVDV biotype infection.

During the NCP-BVDV biotype, but not the CP-BVDV infection, this autoprotease activity requires a limiting cellular cofactor, the DNAJC14 chaperone (11, 12). Nevertheless, the NCP-BVDV variant that retained the ability to produce NS3 at a compatible level with the parent biotype, was recovered from CP-BVDV/NADL strain. This change in biotype was linked to the presence of a single (Y2441C) mutation within the NS4B protein (13). Thus, the multifactorial nature of the BVDV virulence emphasized the urgent needs for mining other unexplored virulence-related factors. Better understanding of the virulence and antigenicity factors is fundamental for development of effective vaccines and diagnostic assays against various biotypes of BVDV infection in cattle.

Previously, the *in vivo* virulence-based studies of BVDV were mainly based on the roles of a single or limited number of viral proteins. Studies providing a broad view that cover the whole BVDV still need to be made available. Nowadays, machine learning, artificial intelligence, bioinformatics analysis, and tools are widely used. These tools use the genomics and omics-rich sources of data in versatile biological fields such as vaccine design, studying the virulence and pathogenicity of microbes (14, 15).

Immunoinformatic has been used to predict and design epitope-based vaccines for BVDV and other viruses (4, 16–22). Similarly, bioinformatics has also been used to predict virulence of viral proteins (23–25), bacterial proteins (26–28) and protozoal proteins (29). The present study aimed to evaluate the predicted immunogenicity and virulence of the proteins of BVDV; to use these parameters to elucidate the differences between BVDV1 and BVDV2 in addition to their sub-genotypes, and to link the predicted traits with existing knowledge on BVDV. Moreover, the reported antigenicity scores might help in assisting future vaccine design trials.

## 2. Methodology

### 2.1. Sequence retrieval and processing

All protein sequences of pestivirus A (BVDV1) and pestivirus B (BVDV2) along with their relevant data were retrieved from the Virus Pathogen Resource^1^. It includes sequences of Npro, Capsid (C), Erns, E1, E2-P7, E2, P7, NS2-3, NS3, NS4A, NS4B, NS5A and NS5B proteins. The short sequences with less than 90% of the standard length of each protein were excluded. Furthermore, sequences with an unknown year of collection of the source sample were also excluded. Sequences of each viral protein were aligned using Clustal-W method in MEGA 11 (30) to trim residues beyond the ends of each protein. Sequences of the E2-P7 were trimmed to separate E2 (372 amino acids) from P7 (70 amino acids) sequences. Similarly, NS2-3 sequences were aligned and trimmed to separate NS2 (453 amino acids) from NS3 (683 amino acids) sequences. Finally, a single fasta file containing all sequences in unaligned status was generated and used for the prediction of protein virulence, antigenicity and antigenic motifs.

### 2.2. Phylogenetic analysis of BVDV

Sub-genotypes of some BVDV strains/isolates were documented in, and obtained from, the Genbank (Supplementary Table 1). The phylogenetic analysis was used to tag BVDV strains with unknown or old sub-genotypes. The analysis was performed based on the aligned nucleotide sequences of the Npro-and E2-encoding regions using the Neighbor-Joining method in MEGA11. Ninety-seven BVDV strains were used as references (Supplementary Table 1I). Evolutionary distance was estimated by the Maximum Composite Likelihood method using the number of substitutions per site as a unit and homogenous pattern among lineages.

### 2.3. Prediction of pathogenic/virulent BVDV proteins

Prediction of pathogenic/virulent proteins of BVDV was performed using the MP3 server^2^ (14). Prediction was performed with the default setting (threshold −0.2). The hybrid findings from support vector machine (SVM) + hidden markov model (HMM) were taken into account and SVM pathogenicity scores were used for further analysis. To determine the predicted pathogenic proteins, scores were averaged for each viral protein. Pathogenicity scores of BVDV genotypes and sub-genotypes were compared at the levels of the individual proteins, all pathogenic proteins, and all proteins. Temporal change in the predicted virulence was presented as Spearman correlation between pathogenicity scores and year of collection of sequenced samples.

### 2.4. Prediction of the BVDV antigenic proteins and antigenic motifs

Prediction of the BVDV immunogens was conducted using VaxiJen v2.0 server^3^ (31). Prediction was executed with the selection of virus option under target pathogen and default threshold of 0.4. To determine the predicted antigenic proteins, scores were averaged for each viral protein. Antigenicity scores of BVDV genotypes and sub-genotypes were compared at the levels of the individual proteins, all antigenic proteins, and all proteins. These scores were also correlated with the year of collection of sequenced samples to indicate temporal changes in predicted antigenicity. The prediction of the BVDV antigenic motifs was accomplished using the EMBOSS antigen server.^4^ These predictions were conducted with a 9mer length. Scores of the motifs per each sequence were averaged and correlated with VaxiJen antigenicity scores of the corresponding sequence. The most frequent motifs in each protein were determined and used to generate epitope profiles using GraphPad Prism 8.4.2. software (Graph-Pad Software Inc. La Jolla, CA, United States).

### 2.5. Data analysis

Microsoft Excel was used for data handling and calculation of descriptive statistics. Spearman correlation, comparisons test and generation of graphics were done in GraphPad Prism 8.4.2 software. Based on distribution of the data, normal distribution, unpaired t test with Welch’s correction was used to compare the two groups, while Brown-Forsythe and Welch ANOVA test with Dunnett’s T3 multiple comparisons test was used to compare more than two groups. A value of *p* of < 0.05 was considered significant. Data were presented as Mean ± SD.

## 3. Results

### 3.1. Sequences retrieval and phylogenetic analysis of BVDV

**Figure 1:**
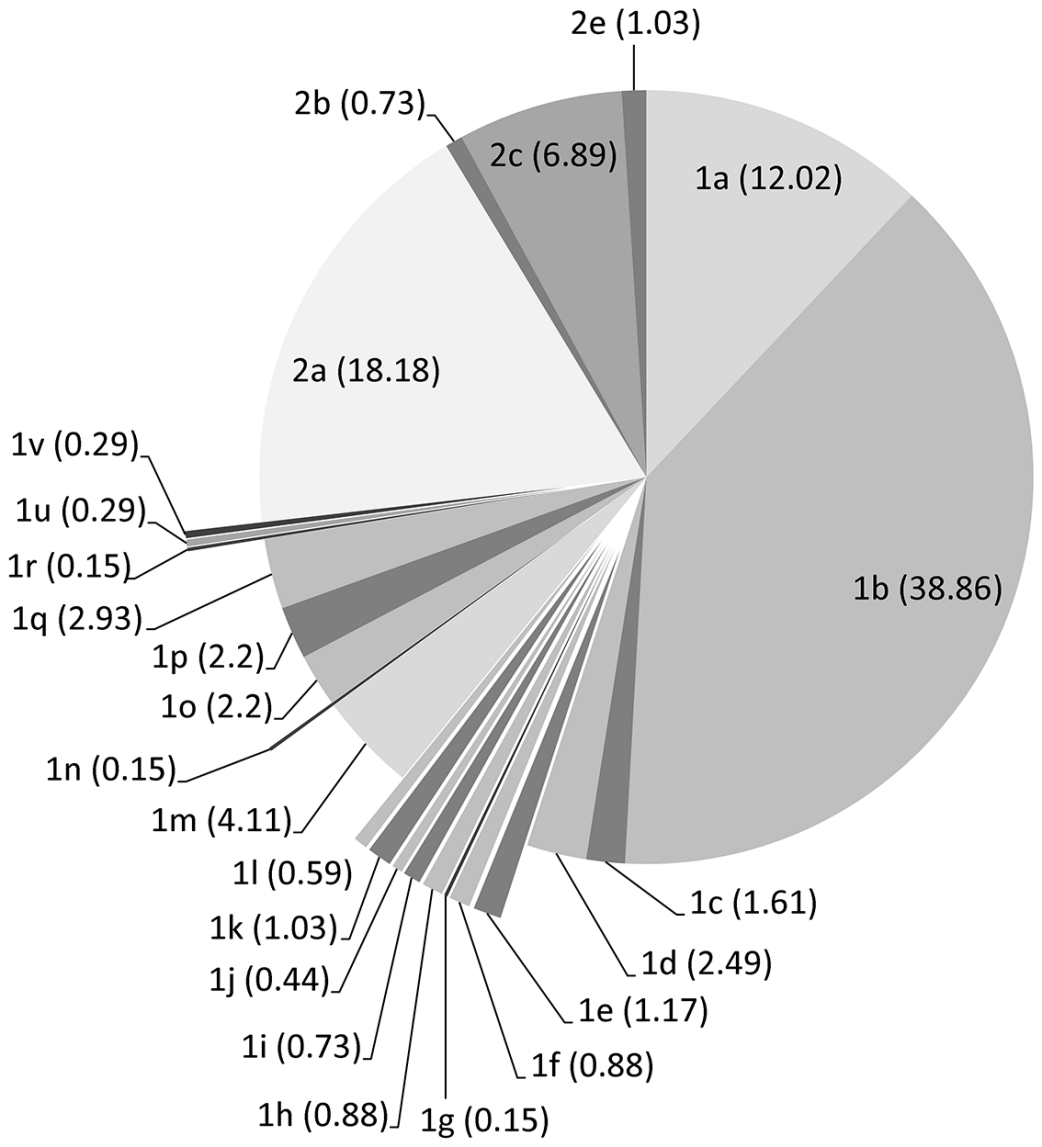
Distribution of the BVDV strains/isolates over subgenotypes according to Genbank and the phylogenetic analysis of the nucleotide sequences encoding the Npro and E2 proteins. Counts of sequences in each subgenotype are shown as percentage within the brackets.

A total of 8,776 and 2,961 protein sequences of Pestivirus-A (BVDV1) and Pestivirus-B (BVDV2), respectively, were retrieved from the virus pathogen resource. A total of 3,003 and 1,580 protein sequences for BVDV1 and BVDV2, respectively, remained after the removal of short sequences or sequences with unknown collection years. The number of sequences used for individual proteins is shown in Table 1. Phylogenetic analysis of the nucleotide sequence encoding the Npro and E2 proteins showed that most of the strains/isolates belong to sub-genotypes 1a, 1b, 2a, 2c, and to a lesser extent 1d, 1m and 1q sub-genotypes as shown in Figure 1. Four sequences that were retrieved as Pestivirus-A turn out to be Pestivirus-B, lifting 2,999 sequences (499 strains) and 1,584 sequences (182 strains) for BVDV1 and BVDV2, respectively (Supplementary Figure 1; Supplementary Table 1).

**Table 1:** Spearman correlation between pathogenicity/antigenicity scores and years of collection of sequenced samples.

Protein	Number of protein sequences	SVM Pathogenicity score		VaxiJen Antigenicity score	
		Spearman R value	P (two-tailed) value	Spearman R value	P (two-tailed) value
*BVDV1*					
Overall	2999^*^	0.04550	0.0127	−0.01353	0.4589
Npro	361	0.1681	0.0014	−0.080308	0.1151
C-protein	237	0.4636	<0.0001	0.02781	0.6702
Erns	274	0.1395	0.0209	−0.2559	<0.0001
E1	233	−0.09752	0.1378	−0.3735	<0.0001
E2	421	0.4251	<0.0001	−0.4256	<0.0001
P7	172	−0.1164	0.1282	0.2245	0.0031
NS2	213	0.1025	0.1360	0.4153	<0.0001
NS3	219	−0.1785	0.0081	−0.08692	0.2001
NS4A	220	−0.2957	<0.0001	0.0906	0.1806
NS4B	217	0.4758	<0.0001	−0.02838	0.6776
NS5A	217	−0.2547	0.0001	0.01125	0.8691
NS5B	215	0.2180	0.0013	−0.1216	0.0751
*BVDV2*					
Overall	1584^*^	0.2624	0.2818	−0.01681	0.5038
Npro	150	0.04899	0.5516	−0.05077	0.5372
C-protein	146	0.1154	0.1655	0.3345	<0.0001
Erns	141	0.1151	0.1742	−0.02695	0.7511
E1	141	0.2487	0.0029	−0.06881	0.4175
E2	172	−0.1131	0.1397	−0.3724	<0.0001
P7	114	0.06772	0.474	−0.07984	0.3984
NS2	114	0.2848	0.0021	−0.08968	0.3427
NS3	119	0.05837	0.5284	−0.1249	0.1757
NS4A	127	0.1173	0.1892	−0.1604	0.0717
NS4B	121	0.01716	0.8518	−0.1676	0.0661
NS5A	117	0.06923	0.4583	0.08153	0.3822
NS5B	122	−0.1852	0.0411	0.05387	0.5556

* Four sequences that were retrieved as pestivirus A (BVDV1) turn out to be pestivirus B (BVDV2). The total number of the sequences changed from 3,003 and 1,580 to 2,999 and 1,584 for BVDV1 and BVDV2, respectively.

### 3.2. Prediction of pathogenicity/virulence of BVDV proteins

**Figure 2:**
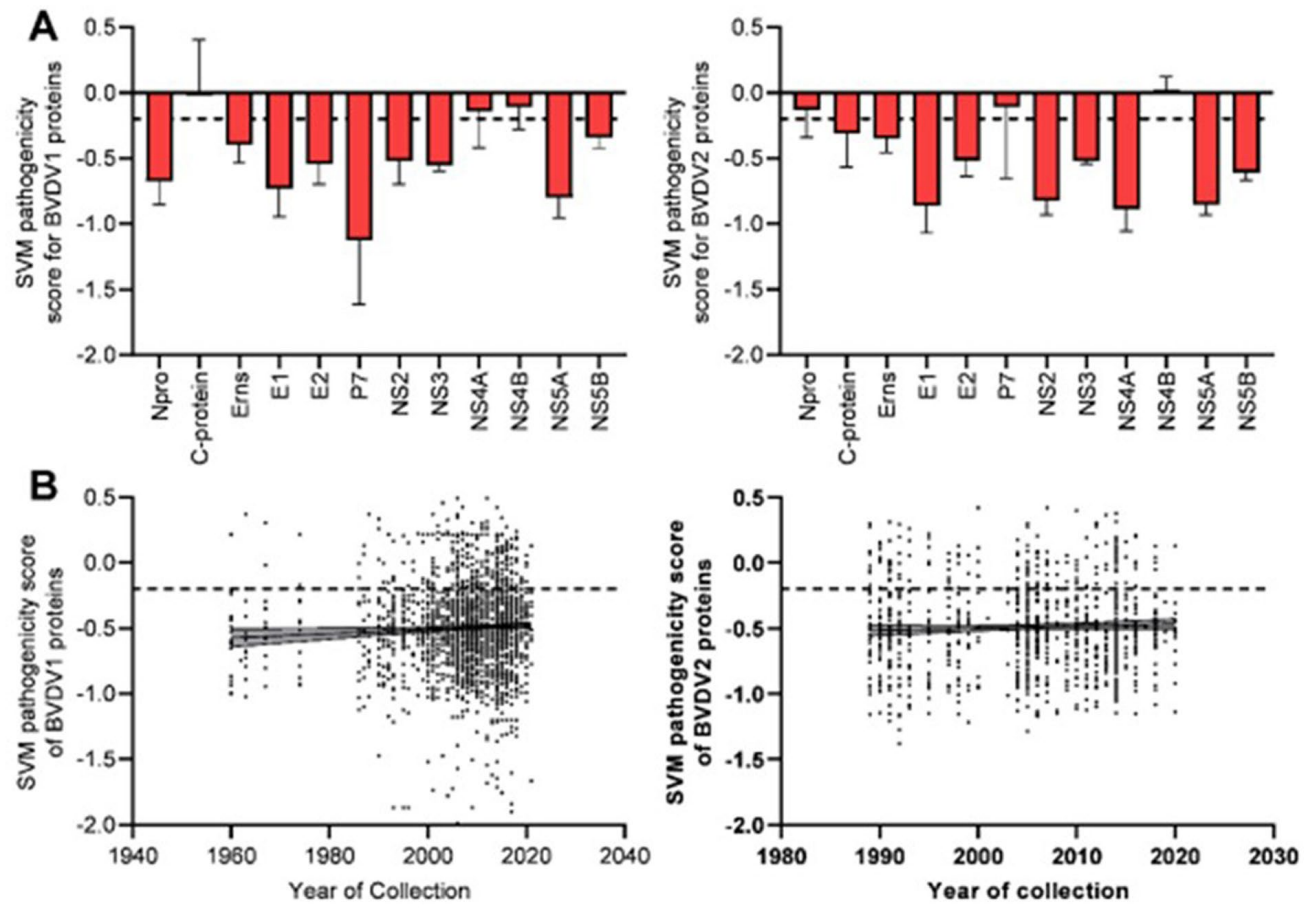
Results of SVM pathogenicity analysis for BVDV1 and BVDV2 proteins, **(A)** mean and SD of the estimated scores, **(B)** correlation between SVM pathogenicity scores and years of collection of the sequenced samples. Dash lines indicate the default threshold point.

Prediction of pathogenic/virulent BVDV proteins was conducted using the MP3 tool. Obtained SVM pathogenicity scores revealed differences in the expected virulent proteins between BVDV1 and BVDV2 (Figure 2A). The NS4B is predicted to be a virulence protein in both types. In addition, both the Capsid (C) and the NS4A proteins of BVDV1; and the N^pro^ and P7 proteins of BVDV2 were predicted to play some essential roles in the viral virulence. There was no significant difference in the overall SVM pathogenicity scores between BVDV1 and BVDV2. Comparing the pathogenicity scores of BVDV1 and BVDV2 based on the individual proteins revealed the presence of significant differences in scores of N^pro^ and P7 proteins (Tables 2-I, III). The correlation between SVM pathogenicity scores and the year of collection of the sequenced samples showed a significant increase in the estimated pathogenicity index over time for BVDV1 (value of *p* = 0.0127) but not for BVDV2 (value of *p* = 0.2818) as shown in Figure 2B and Table 1. At the level of individual proteins, temporal correlation showed a significant increase in scores of six and two proteins of BVDV1 and BVDV2, respectively, and a significant decrease in scores of three and one proteins of BVDV1 and BVDV2, respectively, as shown in Table 1 and Supplementary Figure 2.

**Table 2:** Comparison of the scores of SVM pathogenicity and VaxiJen antigenicity between bovine viral diarrhea virus (BVDV) genotypes and subgenotypes.

Compared groups	Mean diff.	95.00% CI of diff.	Adjusted *p* value
*I-Comparison of overall pathogenicity scores between BVDV1 and BVDV2*			
BVDV1 vs. BVDV2 (scores of all proteins)	0.002739	−0.01950 to 0.02497	0.8092
BVDV1 vs. BVDV2 (scores of pathogenic proteins)^A^	0.00397	−0.03673 to 0.04467	0.8482
*II-Comparison of overall antigenicity scores between BVDV1 and BVDV2*			
BVDV1 vs. BVDV2 (scores of all proteins)	−0.01264	−0.02069 to −0.004594	0.0021
BVDV1 vs. BVDV2 (scores of antigenic proteins)^B^	−0.02151	−0.02603 to −0.01698	<0.0001
*III-Comparison of pathogenicity of individual proteins between BVDV1 and BVDV2*			
BVDV1-Npro vs. BVDV2-Npro	−0.5471	−0.6076 to-0.4865	<0.0001
BVDV1-C-protein vs. BVDV2-C-protein	0.3169	−2.945e+075 to 2.945e+075	>0.9999
BVDV1-Erns vs. BVDV2-Erns	−0.04917	−1.035e+075 to 1.035e+075	>0.9999
BVDV1-E1 vs. BVDV2-E1	0.1283	−1.747e+075 to 1.747e+075	>0.9999
BVDV1-E2 vs. BVDV2-E2	−0.02796	−1.095e+075 to 1.095e+075	>0.9999
BVDV1-P7 vs. BVDV2-P7	−1.019	−1.213 to −0.8240	<0.0001
BVDV1-NS2 vs. BVDV2-NS2	0.3045	−1.290e+075 to 1.290e+075	>0.9999
BVDV1-NS3 vs. BVDV2-NS3	−0.0336	−3.240e+074 to 3.240e+074	>0.9999
BVDV1-NS4A vs. BVDV2-NS4A	0.747	−1.991e+075 to 1.991e+075	>0.9999
BVDV1-NS4B vs. BVDV2-NS4B	−0.133	−1.209e+075 to 1.209e+075	>0.9999
BVDV1-NS5A vs. BVDV2-NS5A	0.05301	−1.028e+075 to 1.028e+075	>0.9999
BVDV1-NS5B vs. BVDV2-NS5B	0.2694	−6.187e+074 to 6.187e+074	>0.9999
*IV-Comparison of antigenicity of individual proteins between BVDV1 and BVDV2*			
BVDV1-Npro vs. BVDV2-Npro	−0.01944	−2.450e+074 to 2.450e+074	>0.9999
BVDV1-C vs. BVDV2-C	0.03812	−2.783e+074 to 2.783e+074	>0.9999
BVDV1-Erns vs. BVDV2-Erns	−0.02764	−2.034e+074 to 2.034e+074	>0.9999
BVDV1-E1 vs. BVDV2-E1	0.003476	−2.345e+074 to 2.345e+074	>0.9999
BVDV1-E2 vs. BVDV2-E2	0.09766	−2.905e+074 to 2.905e+074	>0.9999
BVDV1-P7 vs. BVDV2-P7	−0.02916	−0.04922 to −0.009099	0.0001
BVDV1-NS2 vs. BVDV2-NS2	0.0197	−2.178e+074 to 2.178e+074	>0.9999
BVDV1-NS3 vs. BVDV2-NS3	−0.00544	−4.583e+073 to 4.583e+073	>0.9999
BVDV1-NS4A vs. BVDV2-NS4A	0.03798	−1.864e+074 to 1.864e+074	>0.9999
BVDV1-NS4B vs. BVDV2-NS4B	0.007998	0.004130 to 0.01187	<0.0001
BVDV1-NS5A vs. BVDV2-NS5A	−0.05395	−0.06160 to −0.04630	<0.0001
BVDV1-NS5B vs. BVDV2-NS5B	0.04876	−1.157e+074 to 1.157e+074	>0.9999
*V-Comparison of pathogenicity scores of predicted pathogenic proteins of BVDV1 subgenotypes with highest group (1b)* *^C^*			
1b vs. 1a	0.1731	−2.495e+075 to 2.495e+075	>0.9999
1b vs. 1c	0.1023	−0.1080 to 0.3126	0.661
1b vs. 1d	0.1251	−0.1280 to 0.3781	0.6685
1b vs. 1e	0.1432	−0.05364 to 0.3400	0.2211
1b vs. 1f	0.2864	0.09581 to 0.4769	0.0004
1b vs. 1g	0.03824	−1.704 to 1.781	>0.9999
1b vs. 1h	0.1302	−0.1164 to 0.3767	0.5698
1b vs. 1i	0.1616	−0.07954 to 0.4028	0.2632
1b vs. 1j	0.2467	−1.734 to 2.227	0.7547
1b vs. 1k	0.2693	0.1374 to 0.4011	<0.0001
1b vs. 1m	0.2109	0.01845 to 0.4033	0.0122
1b vs. 1n	0.2912	−1.381 to 1.963	0.5365
1b vs. 1o	0.1196	−0.9742 to 1.213	0.8279
1b vs. 1q	0.1356	−0.06847 to 0.3397	0.2727
1b vs. 1r	0.09139	−2.243 to 2.426	0.9996
1b vs. 1u	−0.5996	−27.69 to 26.49	0.8221
*VI-Comparison of antigenicity scores of predicted antigenic proteins of BVDV1 subgenotypes with highest group (1a)*			
1a vs. 1b	0.01203	0.008838 to 0.01523	<0.0001
1a vs. 1c	−0.00166	−0.03618 to 0.03286	>0.9999
1a vs. 1d	0.007163	−0.02163 to 0.03595	0.9998
1a vs. 1e	0.001186	−0.02905 to 0.03142	>0.9999
1a vs. 1f	0.001581	−0.03844 to 0.04160	>0.9999
1a vs. 1g	0.007357	−0.1314 to 0.1462	>0.9999
1a vs. 1h	0.01286	−0.02112 to 0.04685	0.9734
1a vs. 1i	0.02731	−0.006620 to 0.06125	0.1417
1a vs. 1j	0.008846	−0.09053 to 0.1082	>0.9999
1a vs. 1k	−0.00076	−0.02772 to 0.02621	>0.9999
1a vs. 1m	0.01905	−0.01661 to 0.05471	0.7339
1a vs. 1n	0.02699	−0.08239 to 0.1364	0.9784
1a vs. 1o	0.007315	−0.1052 to 0.1198	>0.9999
1a vs. 1p	0.02589	−0.1229 to 0.1746	0.9981
1a vs. 1q	0.01014	−0.02474 to 0.04502	0.9983
1a vs. 1r	−0.00232	−0.1336 to 0.1289	>0.9999
1a vs. 1u	0.047	−0.04862 to 0.1426	0.3724
*VII-Comparison of pathogenicity scores of predicted pathogenic proteins of BVDV2 subgenotypes with highest group (2c)*			
2c vs. 2a	0.366	0.2426 to 0.4895	<0.0001
2c vs. 2b	0.3879	−0.006928 to 0.7828	0.0277
2c vs. 2e	0.3573	0.1378 to 0.5767	0.0002
*VIII-Comparison of antigenicity scores of predicted antigenic proteins of BVDV2 subgenotype with highest group (2b)*			
2b vs. 2a	0.004194	−0.02583 to 0.03422	0.9712
2b vs. 2c	0.008739	−0.02295 to 0.04042	0.8299
2b vs. 2e	0.007149	−0.02833 to 0.04262	0.9274
*IX-Comparison of antigenicity scores of the E2 protein of BVDV1 subgenotypes with lowest group (1b)*			
1b E2 vs. 1a E2	−0.07307	−0.08362 to −0.06253	<0.0001
1b E2 vs. 1c E2	−0.06246	−0.1401 to 0.01515	0.0715
1b E2 vs. 1d E2	−0.05969	−0.1142 to −0.005175	0.0162
1b E2 vs. 1e E2	−0.07331	−0.1064 to −0.04020	0.0001
1b E2 vs. 1f E2	−0.02315	−0.09995 to 0.05365	0.7382
1b E2 vs. 1h E2	−0.0113	−0.1378 to 0.1152	0.9995
1b E2 vs. 1i E2	−0.06344	−0.2093 to 0.08239	0.3152
1b E2 vs. 1k E2	−0.05073	−0.1185 to 0.01705	0.0889
1b E2 vs. 1m E2	−0.04797	−0.08210 to −0.01384	0.0037
1b E2 vs. 1n E2	−0.0596	−1.139 to 1.020	0.4257
1b E2 vs. 1p E2	−0.083	−0.2521 to 0.08608	0.0987
1b E2 vs. 1q E2	−0.03024	−0.1154 to 0.05494	0.6758
*X-Comparison of antigenicity scores of the E2 protein of BVDV2 subgenotypes with lowest group (2c)*			
2c E2 vs. 2a E2	−0.03653	−0.04386 to −0.02920	<0.0001
2c E2 vs. 2b E2	−0.02719	−0.04538 to −0.008997	0.0046
2c E2 vs. 2e E2	−0.06019	−0.07834 to −0.04205	<0.0001

A Pathogenic proteins are the C, NS4A, and NS4B in BVDV1; and Npro, p7, and NS4B in BVDV2.

B Antigenic proteins are all proteins except Npro, P7, and NS4B in both BVDV1 and BVDV2.

C Selection of the compared group considered the number of the sequences available in subgenotypes as some subgenotypes did not contain the required number of sequences for statistical analysis.

Unpaired *t*-test with Welch’s correction was used in parts I and II, and Dunnett’s T3 multiple comparisons test was used for parts III–X.

**Figure 3:**
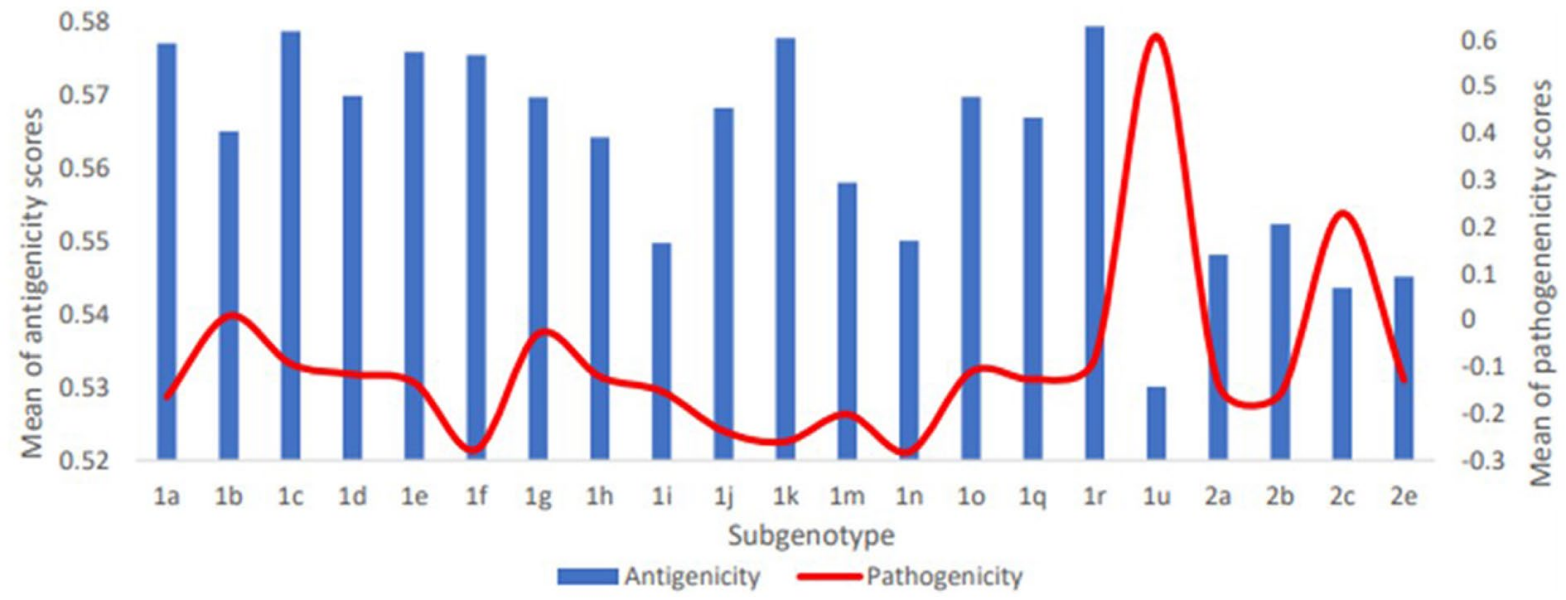
Averaged pathogenicity and antigenicity scores for subgenotypes of BVDV1 and BVDV2. For pathogenicity of BVDV1, scores of C, NS4A and NS4B proteins were averaged; for Pathogenicity of BVDV2, scores of Npro, P7 and NS4B proteins were averaged. For antigenicity of both genotypes, scores of all proteins except Npro, P7 and NS4B proteins were averaged. The shown averages and the numbers of the involved sequences are presented in Supplementary Table 3.

The predicted scores of BVDV proteins involved in the pathogenicity were used to compare the pathogenicity of the different sub-genotypes of the virus (Figure 3). The average pathogenicity scores for the sub-genotypes (1u, 1b, 1g and 2c) were relatively high while those of sub-genotypes (1f, 1k, 1n, 2a and 2b) were relatively low as shown in Figure 3. The significance of this variation is not clear due to the low numbers of sequences available for analysis from some sub-genotypes. The pathogenicity scores of the sub-genotype 1b were significantly higher than the corresponding scores of that of the sub-genotypes (1f, 1k, and 1m; Table 2-V). Similarly, the pathogenicity scores of the sub-genotype 2c were significantly higher than the corresponding scores of the sub-genotypes (2a, 2b and 2e; Table 2-VII). Though not included in the comparative comparison, the averaged pathogenicity score for the Erns protein of the sub-genotypes 1m (*n* = 18), 1p (*n* = 5), 1o (*n* = 2), 1v (*n* = 2), 1g (*n* = 1) and 1u (*n* = 1) and C protein of sub-genotypes 2c (*n* = 22) and 2e (*n* = 7) were also greater than the default threshold of (−0.2). It is most likely play pathogenic roles (Supplementary Tables 2, 3).

### 3.3. Prediction of BVDV antigenic proteins

**Figure 4:**
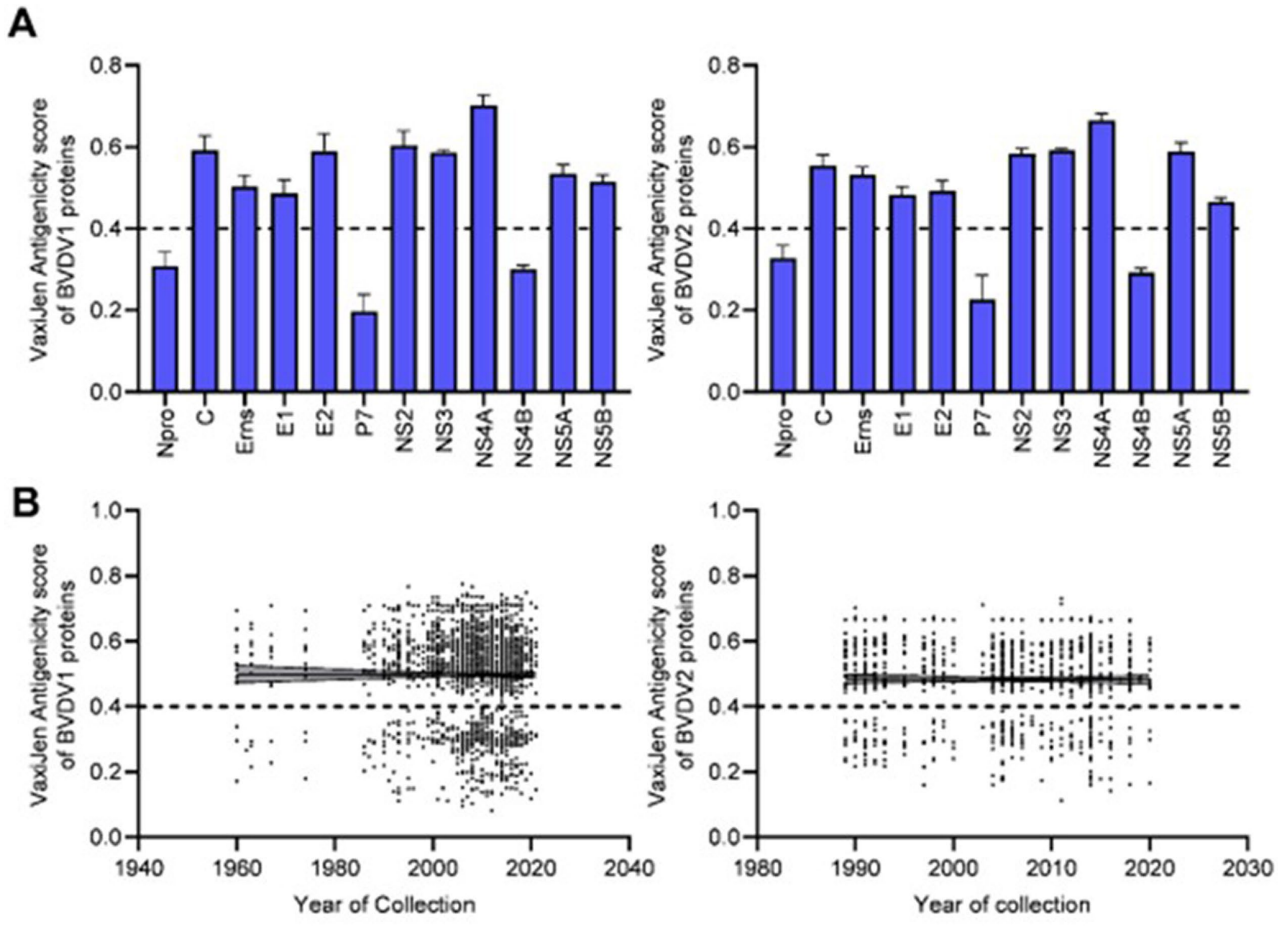
Results of VaxiJen antigenicity analysis for BVDV1 and BVDV2 proteins, **(A)** mean and SD of the estimated scores, **(B)** correlation between VaxiJen antigenicity scores and years of collection of the sequenced samples. Dash lines indicate the default threshold point.

The VaxiJen and EMBOSS antigen prediction tools were used to rate the antigenicity scores of the BVDV proteins. The averages of the EMBOSS-motif scores per each sequence showed high correlation with VaxiJen antigenicity scores of corresponding sequences (value of *p* = <0.0001). Additionally, the obtained motif profiles were almost identical for both BVDV1 and BVDV2 (Supplementary Figure 4). Using the default VaxiJen threshold point (0.4) tools, all the BVDV proteins were antigenic, except (N^pro^, P7 and NS4B) proteins, in both genotypes, as shown in Figure 4A. Comparing the antigenicity scores of BVDV1 and BVDV2 revealed the existence of significant differences in the overall antigenicity as well as in the antigenicity of (P7, NS4B and NS5A) proteins (Tables 2-II, -IV).

The overall temporal correlation of the antigenicity scores for both BVDV genotypes showed relative stability of antigenicity over time as shown in Figure 4B and Table 1. At the level of the individual proteins, temporal correlation showed a significant decrease in the antigenicity of the main immunodominant protein, E2, in both genotypes as well as a significant decrease in antigenicity of E1 and Erns envelope glycoproteins of the BVDV1. On the other hand, there was a significant increase in antigenicity scores of P7 and NS2 of BVDV1 and C protein of BVDV2 as shown in Table 1 and Supplementary Figure 3. Scoring of the proteins with predicted involvement in the antigenicity of BVDV was used to compare the antigenicity of the sub-genotypes (Figure 3). Antigenicity scores of subgenotypes (1a, 1c, 1k, 1r and 2b) were relatively high while those of (1i, 1n, 1u, and 2c) were relatively low. The antigenicity scores of sub-genotypes 1a were significantly higher than corresponding scores of 1b (Table 2-VI). Considering the antigenicity of only the E2 protein, scores of sub-genotype-1b were also significantly lower than that of subgenotypes (1a, 1d, 1e and 1m; Table 2-IX). Regarding the BVDV2, the E2 protein of the sub-genotype-2c showed relatively lower antigenic scores and were significantly different from that of (2a, 2b and 2e) sub-genotypes. However, the differences were not significant when all antigenic proteins of BVDV2 were involved in the analysis (Table 2-VIII, -X; Figure 3). Noteworthy, the relatively high antigenic and low pathogenic sub-genotypes including (1a, 1f, and 1k) of the BVDV1; and (2a and 2b) of the BVDV2 may represent good targets for vaccine development.

## 4. Discussion

Bovine viral diarrhea virus (BVDV) is one of the leading pathogens of ruminants, especially cattle. Efforts have been made to develop requirements of control of this serious viral infection in cattle; however, studies showed that BVDV was able to persist in many cattle herds across the world despite the massive application of vaccines against this virus (2, 4). Continuous genetic and antigenic diversity and the ability to infect a wide range of hosts including wild animals may affect the performance of the currently used BVDV vaccines and diagnostics assays (32, 33). The high demands for effective BVDV vaccines and diagnostics demonstrate the mandate for a better understanding of the molecular pathogenesis, and immune response of BVDV infection in cattle. Herein, we presented a thorough comparative computational analysis of the pathogenicity and antigenicity of BVDV genotypes, and their sub-genotypes and we already linked the predicted traits in this study of various genotypes/sub-genotypes of BVDV with the existing knowledge on BVDV. The prediction of BVDV pathogenic/virulent proteins was accomplished using the MP3 tool (14), a free online tool that has been used to predict the pathogenicity of proteins of other viruses, particularly SARS-CoV-2 (23–25) as well as some other bovine pathogens such as *Chlamydia psittaci*, *E. coli* and *Theileria parva* (26, 27, 29).

Immunoinformatics is a field of bioinformatics that involves the use of computational tools and algorithms to study the immune system and related biological processes. In the context of viral diseases, immunoinformatics can be used to predict the virulence or pathogenicity of a virus based on its genetic makeup and interaction with the host immune system (15). The virulence of BVDV is determined by a combination of viral and host factors, including the genetic diversity of the virus and the immune response of the host. Immunoinformatics approach was used in predicting the virulence of BVDV by analyzing its genetic sequences and identifying key viral epitopes that interact with the host immune system. These analyses can help in identifying specific viral proteins that are targeted by the host immune response. The present computational models predict the virulence of BVDV strains based on their genetic sequences and their interactions with host cells. By combining computational analysis with experimental data, researchers can gain a better understanding of the complex interactions between viruses and the host, leading to the development of more effective diagnostic and therapeutic strategies. The computational data can be used as a predictive tool for future experiments developed in various fields of research seeking virulence evaluation and vaccine development (15). Of special interest, our research group is going to implement these computational models on our current isolates and sequences from infected herds and give a link between the computational model, new sequences, and the observed clinical intensity.

Antigenic proteins are proteins that can trigger an immune response in the host organism, leading to the production of antibodies that can recognize and neutralize the pathogen. These proteins can be used to develop vaccines, as they can stimulate the immune system to produce a protective response without causing disease. Examples of antigenic proteins in our case is surface glycoprotein or envelop protein (E2) (4). Pathogenic proteins, on the other hand, are proteins produced by pathogens that contribute to the development of disease in the host organism. These proteins may interfere with the normal functioning of cells or tissues, cause inflammation, or induce cell death. It can be both surface and internal proteins (34).

The present findings indicate that the NS4B is a virulence protein for both BVDV1 and BVDV2 infections. The BVDV-NS4B is a hydrophobic protein that induces autophagy. This protein has NTPase activity and plays a role in virus replication (7). A single mutation in BVDV-NS4B was reported to change the BVDV biotype (13). Studies with gene silencing showed that targeting genomic region encoding the (C, NS4B and NS5A) of the BVDV reduced the viral load and cytopathic effects induced by the virus (35). Similarly, certain substitutions in the other member of the family *Flaviviridae*; [Swine Fever virus (CSFV)-NS4B] have affected the virus virulence (36). For example, three substitutions in the CSFV proteome (one in E2 and two in NS4B), were linked to attenuation of the vaccine strain GPE^−^. The readaptation of this strain through the passage in pigs highlighted that the manipulation of the spread was mainly based on the E2 substitution while, the NS4B substitutions was required for viral replication (34). Recently, it has been shown that BVDV–NS4B suppresses interferon-β production by interfering with MDA5-mediated signaling (37).

Based on the finding of the present investigation, the NS4A is predicted as a virulent factor for BVDV1 infection. BVDV-NS4A is a protease cofactor that contains a transmembrane domain to anchor the NS3/NS4A complex and has been shown to be essential for the replication of virus particles (7). BVDV1–NS4A protein (KS86-cp, KS86-ncp and nose strains) has also been reported to inhibit the antiviral response in mammalian cells by binding to Adenosine Deaminase acting on RNA (ADAR), an editor of viral dsRNA, thus inhibiting the dsRNA-induced defense mechanisms (38). Recently, it has been shown that CSFV-NS4A binds to dsRNA and small interfering RNA, thus blocking RNA interference, in mammalian cells. This function of the NS4A was reported to be conserved in all pestiviruses; and CSFV lacking this function showed attenuated viral replication (39).

Based on the mentioned findings, the C protein is predicted to play a role in the virulence of BVDV1. Previous studies on BVDV-C protein demonstrated it as a small, basic, cleavable polypeptide with a structural role. Processed C protein lacks secondary structure and binds RNA nonspecifically with low affinity (40). The C protein is the most abundant structural protein in BVD virion (41). CSFV-C protein was suggested to play a transcriptional regulatory role and to protect viral RNA and progeny virus against INF-induced antiviral mechanisms (42, 43). Due to its interaction with host cellular factors, the C protein was expected to play a role in the pathogenesis and persistence of infection with many viruses of the Flaviviridae (44). In this regard, it has been reported that introducing a single amino acid substitution into NS3 protein of almost entirely deleted C protein-CSFV leads to the rescue of attenuated virus. Consequently, it has been proposed that the C protein may not be essential for virus assembly but for virus virulence (45). Similarly, it has been reported that certain mutations in the P7, NS2 and NS3 proteins may rescue infectivity of C protein defective Hepatitis C virus (HCV) (46). Altogether, the functions of the BVDV-C protein are still to be elucidated.

The Npro protein, which is a self-protease that regulates the production of type-I interferons, is predicted to be a virulence protein for BVDV2 (7). Npro of BVDV1, BVDV2 and CSFV has been shown to inhibit the production of IFN-I (47, 48) by inhibition of transcription (49) as well as ubiquitination and proteasomal degradation (50–52) of IFN-Regulatory Factor-3 (IRF-3). Interfering with the IRF-3-mediated IFN-I production was reported in both cp and ncp BVDV1 (strain NADL) and was counteracted by heat inactivation of the E1 ubiquitin-activating enzyme (53). IFN-antagonism is not the only function of the Npro as CSFV with mutated Npro that abolish this function remained virulent while CSFV with entirely deleted Npro was attenuated in pigs (54). CSFV-Npro protein has also been shown to inhibit IFN-III by interfering with IRF-1 (55). Collectively, the role of Npro in BVDV pathogenesis still awaiting clarification.

P7 protein is expected to play a role in the pathogenicity of BVDV2. The mechanism behind the role of BVDV2-P7 protein in virulence is unknown. Studies on P7 of BVDV1 (strain CP7) revealed that in-frame deletion of this protein affects the production of the infectious virus (56). P7 protein is a viroporin that has been reported to play a significant role in the virulence of CSFV with an unknown mechanism (57). It has been anticipated that ion pore activity of the P7 may protect the progeny virus from acidification (7). Additionally, insertion or duplication were reported in P7 encoding region of highly virulent BVDV2 isolates (58, 59). Whether the P7 protein plays a direct role in virulence, or its encoding region represents a hotspot for recombination and other genetic mechanisms that derives virus virulence needs to be addressed by further investigations.

Noteworthy, the predicted pathogenicity scores for BVDV1 showed a significant increase over time, indicating that the virus continues to develop its virulence tools. Surveying the literature on pestiviruses showed no similar study to compare this finding. The lack of virulence data for a large part of BVDV strains makes the evaluation of this finding more difficult. Using the same prediction tool, the MP3, it has been reported that the pathogenicity scores of SARS-CoV-2 variants showed gradual decrease over time, perhaps in an attempt to adapt to the host (23). Similarly, BVDV was observed for the first time in 1946 as an acute disease but observations in the ensuing years showed that BVDV infection became clinically mild. Thereafter, in the 1980s and 1990s, the emergence of virulent strains became more evident (59, 60).

In the present investigation, we used the VaxiJen tool to predict the immunogenicity of BVDV proteins. VaxiJen tool has been used to predict protein antigenicity for HCV, Dengue virus (61), Bovine Leukemia virus and Ephemeral Fever virus in order of preparing corresponding multiepitope vaccines (62, 63). Compatible results were obtained from VaxiJen and EMBOSS antigen tools. VaxiJen antigenicity scores showed that all of the BVDV proteins are antigenic except the Npro, P7 and NS4B proteins. These findings are in agreement with previous reports stating that after BVDV infection, the immune response may developed against most, if not all, of the BVDV proteins (64). Cytotoxic T lymphocyte (CTL) epitopes were predicted to locate in C, Erns, E2, NS2 and NS3 proteins of BVDV (18). Similarly, (22) reported that 28 epitopes across all BVDV proteins, except C and P7, were cross-reactive with purified bovine CD8^+^T cells isolated from BVDV1 and BVDV2 immunized cattle. Likewise, Riitho et al. (20) showed that E2, NS2, NS3 and NS5A but not P7 and C proteins were highly inducers of T cell responses. However, BVDV-C protein has been shown to induce cellular and humoral immune response in mouse model (65) and a B cell epitope that can induce the formation of antibodies was mapped in the C protein of BVDV (66) and other pestiviruses (67). The main inducers of antibody response against BVDV were reported to be the E2 and NS2-3 proteins and to a lesser extent E1 and Erns glycoproteins (8, 68, 69). Additionally, a low level of anti-NS4B antibody was also detected in calves after natural BVDV infection (70). Similarly, recombinant Npro protein was shown to be an inducer of low immunity in cattle, sheep and goats (7). Notably, the predicted antigenicity scores for the three envelope glycoproteins of BVDV1 and the E2 of BVDV2 showed significant decrease over time while it showed significant increase in NS2 protein of the BVDV1. These should be considered when designing new BVDV vaccines.

Studies on BVDV vaccines showed that the need for safe and efficacious vaccines is still exist (4). For a virus with ability to induce PI and to interfere with immune response, choosing of a vaccine strain should consider its virulence. In this regards, concerns on BVDV vaccine-induced immunosuppression have been raised (71). Strong antigenicity and broad cross protectivity with other strains/subgenotypes are additional factors that should be considered when choosing vaccine candidates. For instance, it has been shown that cross neutralization titers of ≥ 1:512 or ≥ 1:256 are required for marked protection and clinical protection, respectively (72). Based on the predicated high antigenicity and low pathogenicity, the presented analysis suggests that subgenotypes 1a, 1f, 1k, 2a and 2b are suitable targets for BVDV vaccine development. This is in agreement with the wide use of strains belonging to subgenotype 1a in commercial vaccines (71). Previous studies revealed the ability of anti-1a sera to cross neutralize with strains from 1b (72, 73), 1h, 1e (73) and 1d subgenotypes (72, 74). On the other hand, strains of 1a subgenotype also showed low cross neutralization with strains of 1b, 1c, 1d, 1j, 1n, 1o (75–77) as well as 1f, 1l subgenotypes (72, 74). Similarly, protection-challenge experiments showed variable results depending on the used criteria. For example, vaccine strain belonging to 1a subgenotype showed ability to provide complete clinical protection against challenge with 1b strain (71). However, contrary findings were also reported (78).

Regarding subgenotype 1f, our finding agrees with the previous suggestion of this subtype as a vaccine candidate (79). On contrary, Alpay and Yesilbag (72) suggested subgenotypes 1h and 1l rather than 1f as vaccines candidates, based on their cross neutralization with other subgenotypes; where cross neutralization produced by anti-1f sera was low against 1a and 1b strains though it was high against 1d and above the reported protective titer against 1h and 1l strains. Nevertheless, in addition to the high cross neutralization with subgenotype 1d, the cross neutralization of 1f with 1a and 1b strains have also been shown to be higher than the reported protective titer (74). Regarding subgenotype 1k, little is known about cross neutralization of strains of this subgenotype. Anti-1k sera showed low cross neutralization titers, but higher than the reported protective titer, with subtypes 1a, 1b, 1h and 1e (73). Referring to BVDV2, the low pathogenicity and high antigenicity of the subgenotypes 2a and 2b presented in the current study suggest it as vaccine candidates. This is in agreement with the frequent use of strains belonging to subgenotype 2a as vaccines, at least in the United States (78). Available data indicate relatively high cross neutralization between 2b and 2a strains (80). Indeed, the presented findings indicate no significant difference in antigenicity or pathogenicity between the subgenotypes 2a and 2b.

The low cross protection between BVDV genotypes is well known and hence strains from both genotypes are usually included in commercial vaccines (71). However, the significance of differences in cross protection between subgenotypes are of growing concern (78). Vaccines containing multiple strains of the subgenotype 1a have been expected to induce higher cross protection against 1b strains than those containing only one strain of 1a (71). Similarly, preparation of vaccines that combine distinct antigenic strains/epitopes would broaden the cross protectivity against other strains of BVDV (4, 81). Overall, genotyping and serotyping of BVDV strains may provide questionable estimate of cross protection between BVDV strains/vaccines, and it is better to use protectotyping for this purpose, as it previously reported for other viruses, like coronavirus (82, 83). Sorting of BVDV strains/subgenotypes into protectotypes where any strain/subtype in particular protectotype provides high *in vivo* cross protection against other strains/subtypes in that protectotype would help in determining which strains/epitopes to include in vaccines.

## 5. Conclusion

Virulence of BVDV is a multifactorial process that depends on the interplay between functions of several viral proteins. Some of the virulence factors of BVDV have been exposed, however, many other factors await discovery and need to be a focus of future research. In particular, the prediction of the P7 protein as a low antigenic, high pathogenic, at least in BVDV2 mandate more investigation. Noteworthy, predicted virulent proteins of BVDV shared a common role of protecting progeny virus and viral nucleic acid, particularly by antagonizing innate immune response. This is not surprising for a virus capable of inducing PI and immunosuppression. The increasing trend of BVDV1 virulence threatens more losses due to BVDV in an uncontrolled situation. Similarly, the decreasing trend of immunogenicity of the envelope glycoproteins of BVDV1 forecasts decreasing effectiveness of the current vaccines and emphasizes the importance of the inclusion of the NS2 protein in future subunit vaccines.

## 6. Outlook

Identification of virulence factors of BVDV needs to be a focus of the coming research on BVDV. Classification of BVDV strains according to their virulence (for example, highly virulent, moderately virulent, and low virulent) is necessary for studying virulence determinants of BVDV. Constructing a database that contains relevant information on each strain such as spatial and temporal distributions, virulence, biotype, affected hosts, and others will be helpful. Determination of factors involved in transplacental transmission and establishment of PI in the fetus by ncp strains and development of relevant interfering tools is fundamental for successful control of BVDV. Research on, and production of, multiepitope vaccines may be beneficial in areas with multiple serotypes circulating.

## 7. Limitation

The lack of a detailed classification of BVDV strains into cp and ncp biotypes makes it difficult to predict the effects of the biotype. Similarly, virulence classification of BVDV strains is required for further studies on virulence.

## Data availability statement

The original contributions presented in the study are included in the article/Supplementary material, further inquiries can be directed to the corresponding authors.

## Author contributions

AA-M, JH, MK, AA-K, and MH: conceived the idea, collected data, data analysis, and wrote the manuscript. All authors contributed to the article and approved the submitted version.

## Funding

This work was supported by King Abdul-Aziz City for Science and Technology (KACST) with their generous funding through the Strategic Technologies Program, Grant No. 12-BIO 3152-06.

## Conflict of interest

The authors declare that the research was conducted in the absence of any commercial or financial relationships that could be construed as a potential conflict of interest.

## Publisher’s note

All claims expressed in this article are solely those of the authors and do not necessarily represent those of their affiliated organizations, or those of the publisher, the editors and the reviewers. Any product that may be evaluated in this article, or claim that may be made by its manufacturer, is not guaranteed or endorsed by the publisher.

## Footnotes

^1^ https://www.viprbrc.org/

^2^ http://metagenomics.iiserb.ac.in/mp3/application.php

^3^ http://www.ddg-pharmfac.net/vaxijen/

^4^ https://www.bioinformatics.nl/cgi - bin/emboss/antigenic
