# Supplementary material for: Immunoinformatic prediction of the pathogenicity of bovine viral diarrhea virus genotypes: implications for viral virulence determinants, designing novel diagnostic assays and vaccines development
Source: Front Vet Sci. 2023 Jul 6;10:1130147. doi: 10.3389/fvets.2023.1130147 (PMC10359904; doi:10.3389/fvets.2023.1130147)
Supplement: Supplementary file 1 [file Data_Sheet_1.pdf]

A

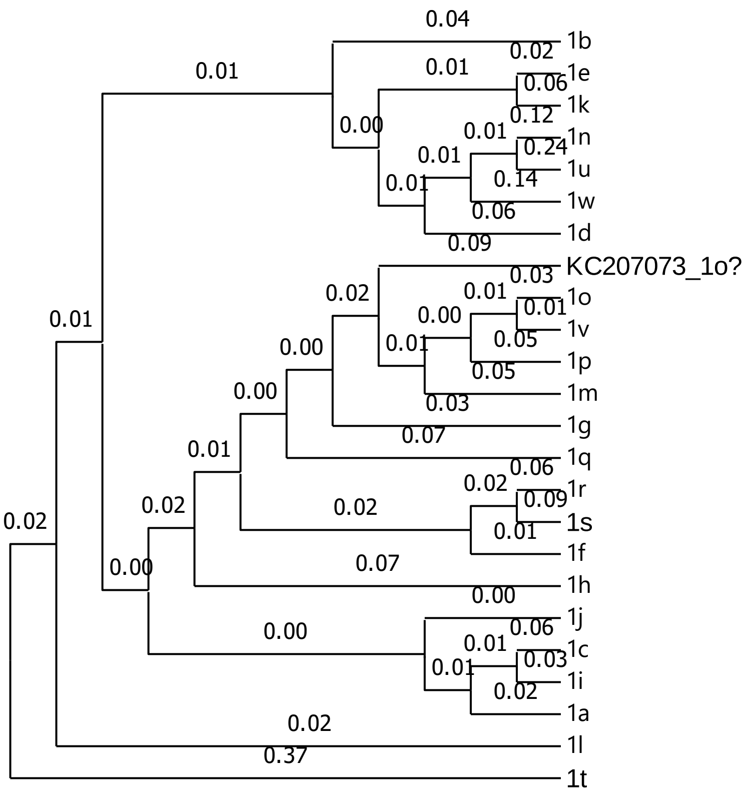
1b
1e
1k
1n
1u
1w
1d
KC207073_1o?
1o
1v
1p
1m
1g
1q
1r
1s
1f
1h
1j
1c
1i
1a
1l
1t

B

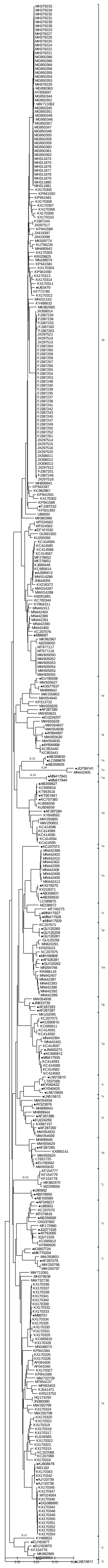

C

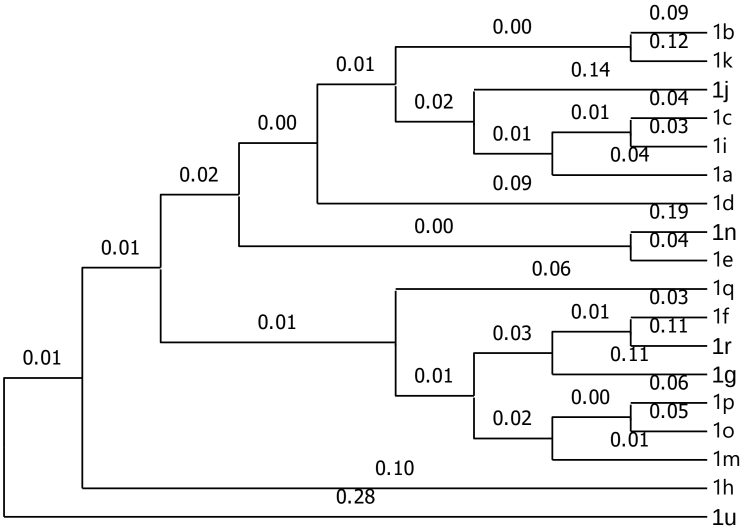

0.00
0.09
1b
0.01
0.12
1k
0.14
0.00
0.02
0.04
1j
0.01
1c
0.03
0.02
0.01
1i
0.04
1a
0.09
1d
0.19
0.01
0.00
1n
0.04
1e
0.06
1q
0.03
0.01
1f
0.11
0.01
0.03
1r
0.11
1g
0.06
0.01
0.00
1p
0.05
0.02
1o
0.01
1m
0.10
1h
0.28
1u

D

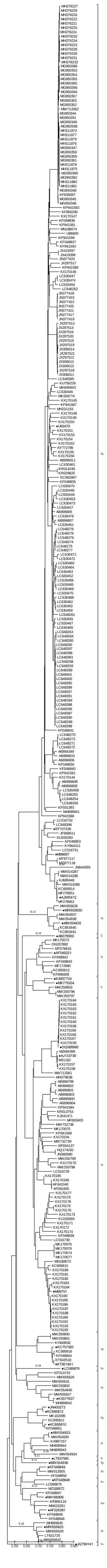

E

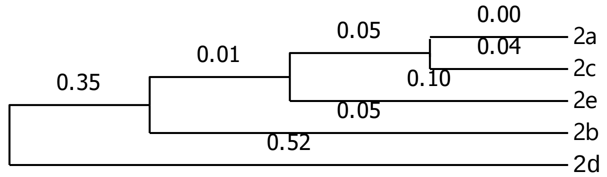

0.00
2a
0.05
0.04
0.01
2c
0.10
0.35
2e
0.05
2b
0.52
2d

F

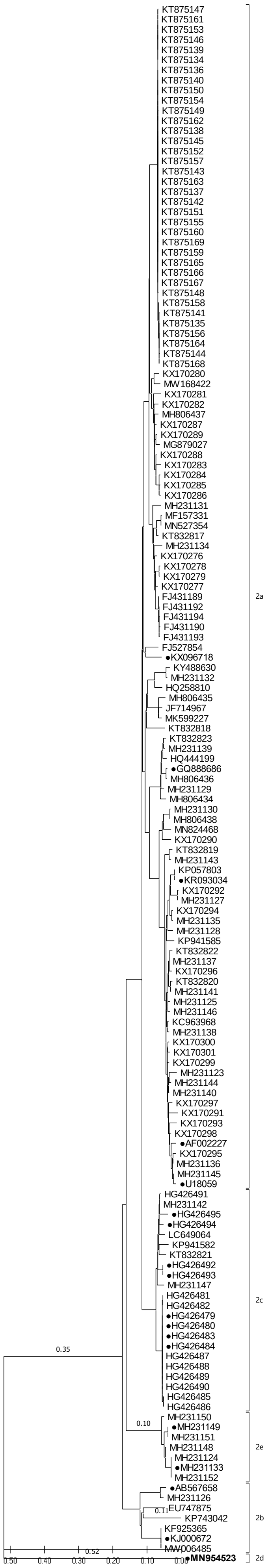

KT875147
KT875161
KT875153
KT875146
KT875139
KT875134
KT875136
KT875140
KT875150
KT875154
KT875149
KT875162
KT875138
KT875145
KT875152
KT875157
KT875143
KT875163
KT875137
KT875142
KT875151
KT875155
KT875160
KT875169
KT875159
KT875165
KT875166
KT875167
KT875148
KT875158
KT875141
KT875135
KT875156
KT875164
KT875144
KT875168
KX170280
MW168422
KX170281
KX170282
MH806437
KX170287
KX170289
MG879027
KX170288
KX170283
KX170284
KX170285
KX170286
MH231131
MF157331
MN527354
KT832817
MH231134
KX170276
KX170278
KX170279
KX170277
FJ431189
FJ431192
FJ431194
FJ431190
FJ431193
FJ527854
KX096718
KY488630
MH231132
HQ258810
MH806435
JF714967
MK599227
KT832818
KT832823
MH231139
HQ444199
GQ888686
MH806436
MH231129
MH806434
MH231130
MH806438
MN824468
KX170290
KT832819
MH231143
KP057803
KR093034
KX170292
MH231127
KX170294
MH231135
MH231128
KP941585
KT832822
MH231137
KX170296
KT832820
MH231141
MH231125
MH231146
KC963968
MH231138
KX170300
KX170301
KX170299
MH231123
MH231144
MH231140
KX170297
KX170291
KX170293
KX170298
AF002227
KX170295
MH231136
MH231145
U18059
HG426491
MH231142
HG426495
HG426494
LC649064
KP941582
KT832821
HG426492
HG426493
MH231147
HG426481
HG426482
HG426479
HG426480
HG426483
HG426484
HG426487
HG426488
HG426489
HG426490
HG426485
HG426486
MH231150
MH231149
MH231151
MH231148
MH231124
MH231133
MH231152
AB567658
MH231126
EU747875
KP743042
KF925365
KJ000672
MW006485
MN954523
0.35
0.10
0.11
0.52
0.50
0.40
0.30
0.20
0.10
0.00
2a
2c
2e
2b
2d

G

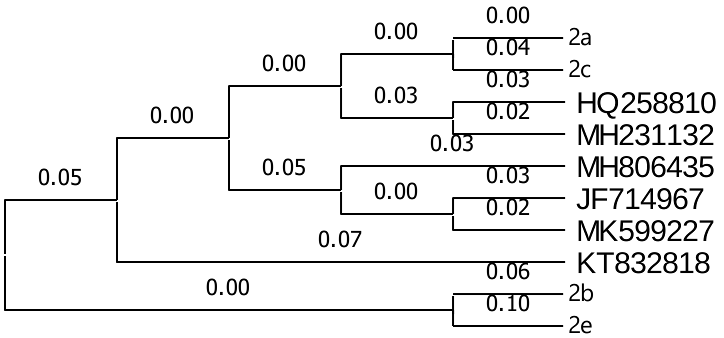
0.00
0.00
2a
0.04
0.00
2c
0.03
0.03
HQ258810
0.00
0.02
MH231132
0.03
0.05
MH806435
0.05
0.03
0.00
JF714967
0.02
MK599227
0.07
KT832818
0.00
0.06
2b
0.10
2e

H

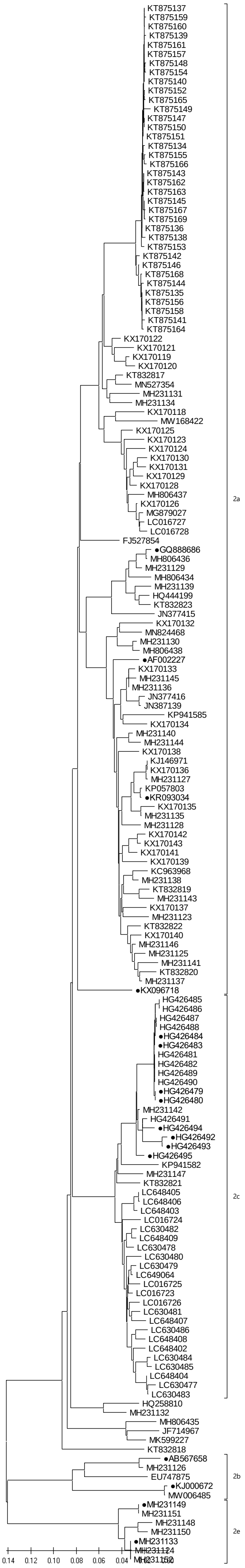
KT875137
KT875159
KT875160
KT875139
KT875161
KT875157
KT875148
KT875154
KT875140
KT875152
KT875165
KT875149
KT875147
KT875150
KT875151
KT875134
KT875155
KT875166
KT875143
KT875162
KT875163
KT875145
KT875167
KT875169
KT875136
KT875138
KT875153
KT875142
KT875146
KT875168
KT875144
KT875135
KT875156
KT875158
KT875141
KT875164
KX170122
KX170121
KX170119
KX170120
KT832817
MN527354
MH231131
MH231134
KX170118
MW168422
KX170125
KX170123
KX170124
KX170130
KX170131
KX170129
KX170128
MH806437
KX170126
MG879027
LC016727
LC016728
FJ527854
●GQ888686
MH806436
MH231129
MH806434
MH231139
HQ444199
KT832823
JN377415
KX170132
MN824468
MH231130
MH806438
●AF002227
KX170133
MH231145
MH231136
JN377416
JN387139
KP941585
KX170134
MH231140
MH231144
KX170138
KJ146971
KX170136
MH231127
KP057803
●KR093034
KX170135
MH231135
MH231128
KX170142
KX170143
KX170141
KX170139
KC963968
MH231138
KT832819
MH231143
KX170137
MH231123
KT832822
KX170140
MH231146
MH231125
MH231141
KT832820
MH231137
●KX096718
HG426485
HG426486
HG426487
HG426488
●HG426484
●HG426483
HG426481
HG426482
HG426489
HG426490
●HG426479
●HG426480
MH231142
HG426491
●HG426494
●HG426492
●HG426493
●HG426495
KP941582
MH231147
KT832821
LC648405
LC648406
LC648403
LC016724
LC630482
LC648409
LC630478
LC630480
LC630479
LC649064
LC016725
LC016723
LC016726
LC630481
LC648407
LC630486
LC648408
LC648402
LC630484
LC630485
LC648404
LC630477
LC630483
HQ258810
MH231132
MH806435
JF714967
MK599227
KT832818
●AB567658
MH231126
EU747875
●KJ000672
MW006485
●MH231149
MH231151
MH231148
MH231150
●MH231133
2a
2c
2b
2e
0.14
0.12
0.10
0.08
0.06
0.04

Supp. figure 1: Results of phylogenetic analysis of the nucleotide sequences encoding Npro and E2 proteins for subtyping of BVDV strains/isolates including A) concise tree for Npro sequences of BVDV1, B) Complete tree of Npro sequences of BVDV1, C) Concise tree of E2 sequences of BVDV1, D) Complete tree of E2 sequences of BVDV1, E) Concise tree of Npro sequences of BVDV2, F) Complete tree of the Npro sequences of BVDV2, G) Concise tree of the E2 sequences of BVDV2, H) Complete tree of the E2 sequences of BVDV2. Reference sequences are tagged with black dot (●). Branch length (≥0.1 in complete tree) are shown above line.

A

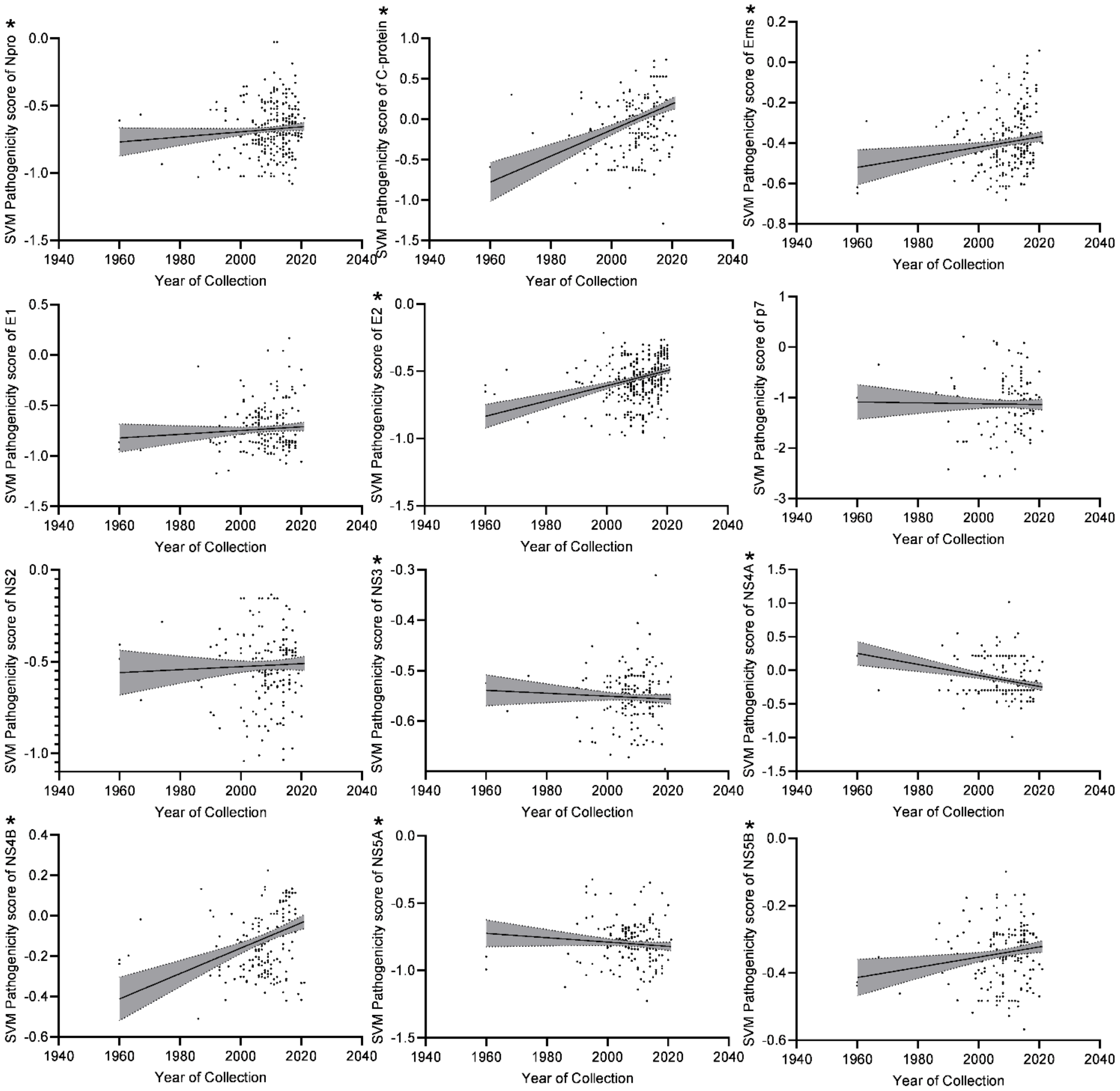
SVM Pathogenicity score of Npro *
SVM Pathogenicity score of C-protein *
SVM Pathogenicity score of Erns *
SVM Pathogenicity score of E1
SVM Pathogenicity score of E2 *
SVM Pathogenicity score of p7
SVM Pathogenicity score of NS2
SVM Pathogenicity score of NS3 *
SVM Pathogenicity score of NS4A *
SVM Pathogenicity score of NS4B *
SVM Pathogenicity score of NS5A *
SVM Pathogenicity score of NS5B *
Year of Collection

B

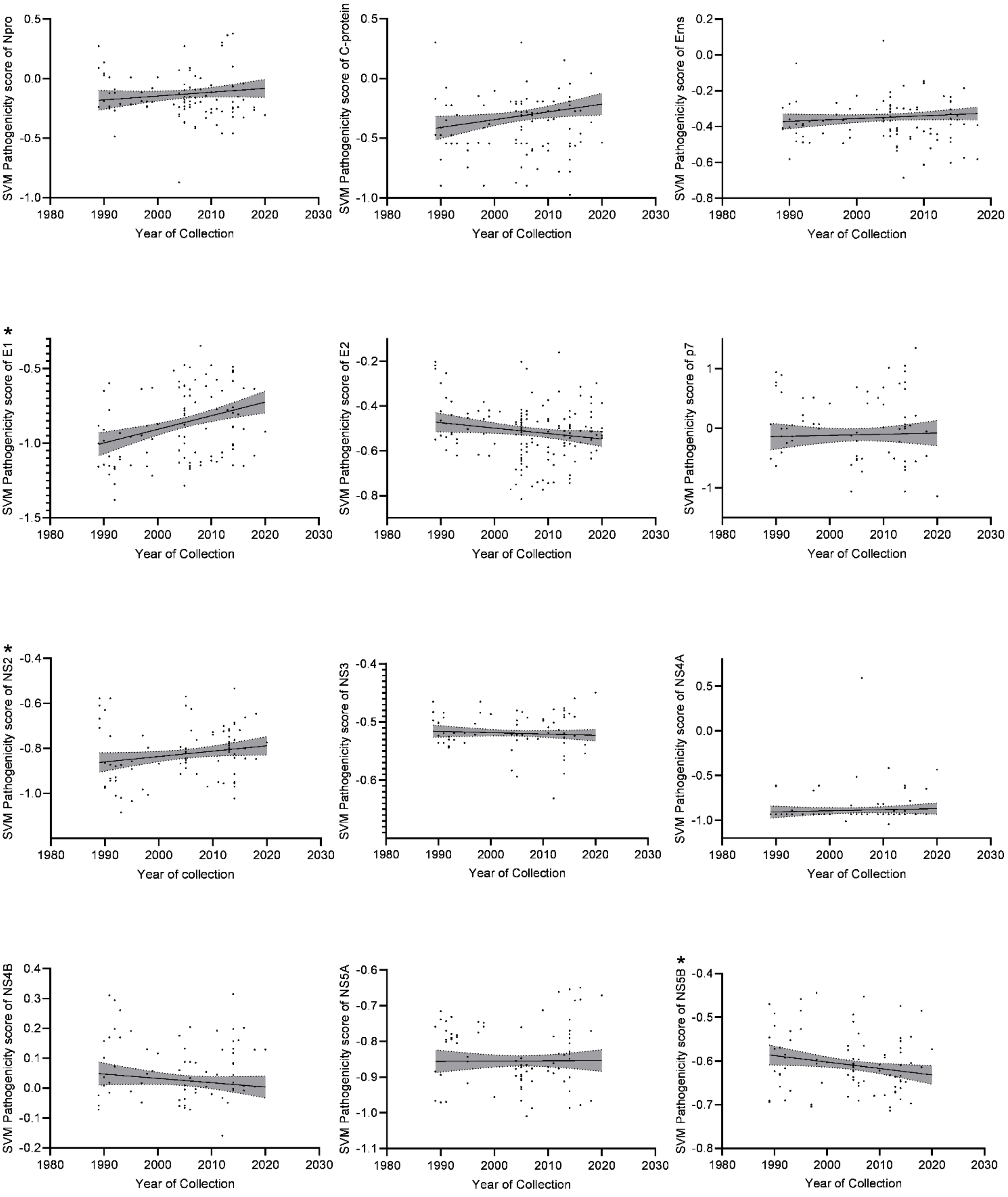

Supp. figure 2: Temporal correlation between SVM pathogenicity scores and years of collection of sequenced samples for A) BVDV1 proteins, B) BVDV2 proteins. Proteins with statistically significant correlation is marked with an asterisk (*).

A

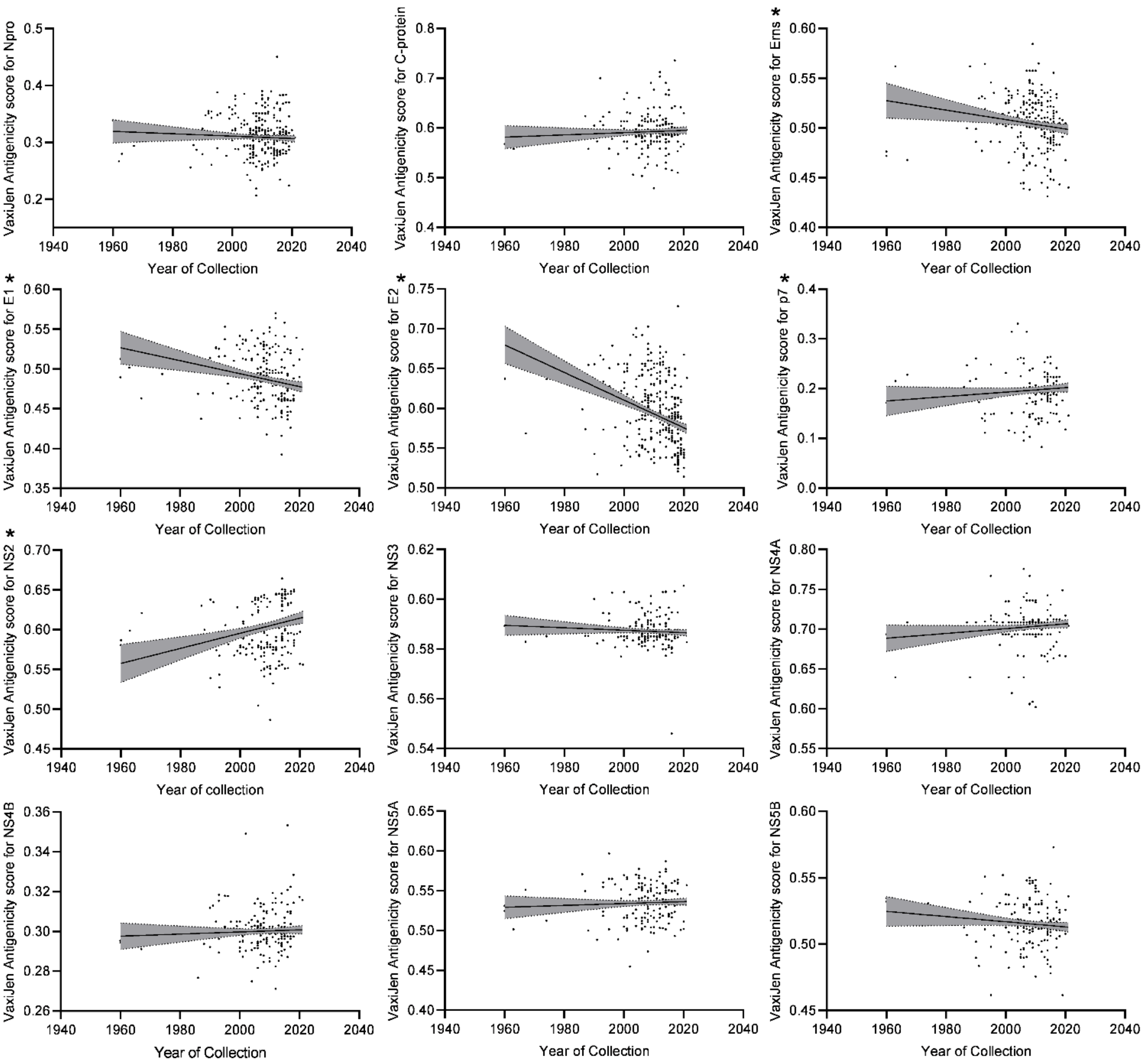
VaxiJen Antigenicity score for Npro
VaxiJen Antigenicity score for C-protein
VaxiJen Antigenicity score for Erns *
VaxiJen Antigenicity score for E1 *
VaxiJen Antigenicity score for E2 *
VaxiJen Antigenicity score for p7 *
VaxiJen Antigenicity score for NS2 *
VaxiJen Antigenicity score for NS3
VaxiJen Antigenicity score for NS4A
VaxiJen Antigenicity score for NS4B
VaxiJen Antigenicity score for NS5A
VaxiJen Antigenicity score for NS5B
Year of Collection
Year of collection
1940
1960
1980
2000
2020
2040

B

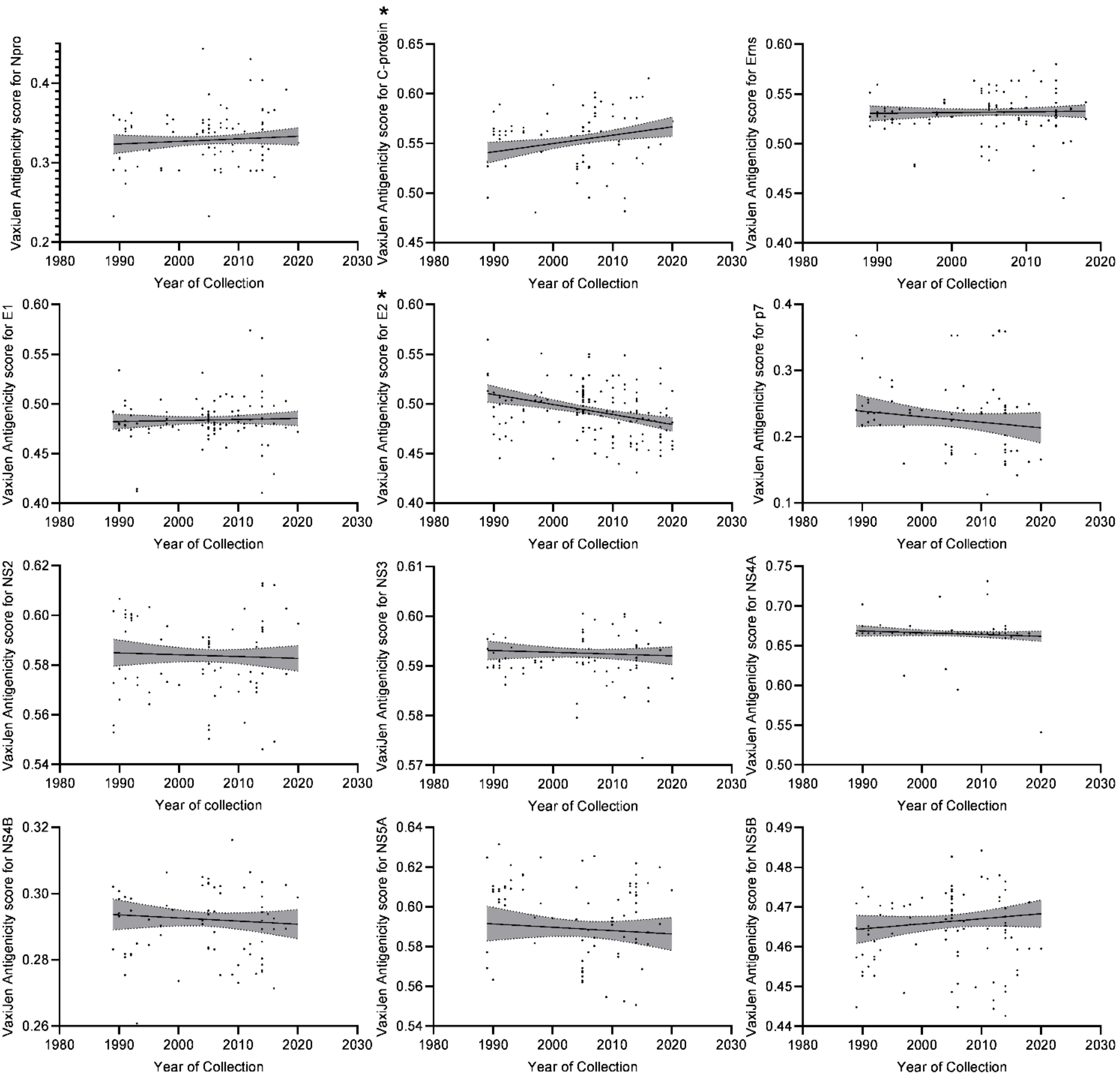

Supp. figure 3: Temporal correlation between VaxiJen antigenicity scores and years of collection of sequenced samples for A) BVDV1 proteins, B) BVDV2 proteins. Proteins with statistically significant correlation is marked with an asterisk (*).

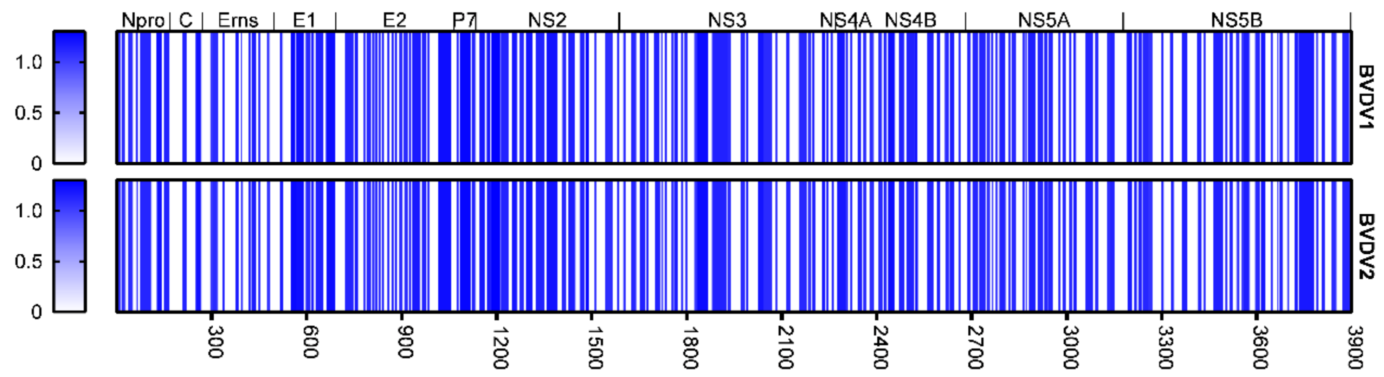

Supp. figure 4: Epitope motif profile for BVDV1 (upper) and BVDV2 (lower). The position of the motifs in BVDV proteome is shown at the bottom. The name of the BVDV proteins is shown on top. Strength of the motifs is shown as color scale on the right.
