# Supplementary material for: Immunoinformatic prediction of the pathogenicity of bovine viral diarrhea virus genotypes: implications for viral virulence determinants, designing novel diagnostic assays and vaccines development
Source: Front Vet Sci. 2023 Jul 6;10:1130147. doi: 10.3389/fvets.2023.1130147 (PMC10359904; doi:10.3389/fvets.2023.1130147)
Supplement: Supplementary file 2 [file Table_1.pdf]

Supp. table 1: Reference strains used for phylogenetic analysis of the nucleotide sequences encoding Npro and E2 proteins and subgenotype of the strains/isolates involved in the current study

1.I: Reference strains used in phylogenetic analysis					
GenBank #	Genotype	Subgenotype	Strain	Reference	Involved tree
AJ133738	BVDV1	1a	type 1	(Mirosław and Polak, 2019)	Npro + E2
DQ088995	BVDV1	1a	Singer_Arg	(Mirosław and Polak, 2019)	Npro + E2
M96751	BVDV1	1a	UNKNOWN-M9675	(Chang et al., 2021)	Npro + E2
AJ585412	BVDV1	1b	VEDEVAC	(Mirosław and Polak, 2019)	Npro + E2
EF101530	BVDV1	1b	KE9	(Mirosław and Polak, 2019)	Npro + E2
M96687	BVDV1	1b	Osloss	(Mirosław and Polak, 2019)	Npro + E2
U63479	BVDV1	1b	CP7	(Han et al., 2018)	Npro + E2
AF049221	BVDV1	1c	Bega	(Chang et al., 2021)	Npro + E2
KC757383	BVDV1	1d	10JJ-SKR	(Mirosław and Polak, 2019)	Npro + E2
KT951841	BVDV1	1d	BJ1308	(Mirosław and Polak, 2019)	Npro + E2
KX577637	BVDV1	1e	SLO/2407/2006	(Mirosław and Polak, 2019)	Npro + E2
KX857724	BVDV1	1i	ACM/BR/2016	(Chang et al., 2021)	Npro + E2
MK775204	BVDV1	1i	CA2006	(Chang et al., 2021)	Npro + E2
AB078950	BVDV1	1j	KS86-1ncp	(Mirosław and Polak, 2019)	Npro + E2
AF526381	BVDV1	1m	ZM-95	(Mirosław and Polak, 2019)	Npro + E2
MH166806	BVDV1	1m	XC	(Mirosław and Polak, 2019)	Npro + E2
LC089876	BVDV1	1n	Shitara/02/06	(Chang et al., 2021)	Npro + E2
JN400273	BVDV1	1q	SD0803	(Mirosław and Polak, 2019)	Npro + E2
KC695810	BVDV1	1q	camel-6	(Mirosław and Polak, 2019)	Npro + E2
KC695812	BVDV1	1q	HB-1	(Han et al., 2018)	Npro + E2
JQ799141	BVDV1	1u	M31182	(Mirosław and Polak, 2019)	Npro + E2
AF002227	BVDV2	2a	C413	(Workman et al., 2016)	Npro + E2
GQ888686	BVDV2	2a	JZ05-1	(Workman et al., 2016)	Npro + E2
KR093034	BVDV2	2a	NY-93	(de Oliveira et al., 2022, Workman e	Npro + E2
KX096718	BVDV2	2a	HB-1511	(de Oliveira et al., 2022)	Npro + E2
AB567658	BVDV2	2b	Hokudai-Lab/09	(de Oliveira et al., 2022, Workman e	Npro + E2
KJ000672	BVDV2	2b	SD1301	(de Oliveira et al., 2022, Workman e	Npro + E2
HG426479	BVDV2	2c	D37-13-2_Dup(-)	(Workman et al., 2016)	Npro + E2
HG426480	BVDV2	2c	D37-13-2_Dup(+)	(Workman et al., 2016)	Npro + E2
HG426483	BVDV2	2c	NRW 12-13_Dup(-)	(Workman et al., 2016)	Npro + E2
HG426484	BVDV2	2c	NRW 12-13_Dup(+)	(Workman et al., 2016)	Npro + E2
HG426492	BVDV2	2c	SH2210-14	(Workman et al., 2016)	Npro + E2
HG426493	BVDV2	2c	SH2210-17	(de Oliveira et al., 2022, Workman e	Npro + E2
HG426494	BVDV2	2c	SH2210-23	(Workman et al., 2016)	Npro + E2
HG426495	BVDV2	2c	VOE 4407	(de Oliveira et al., 2022, Workman e	Npro + E2
MH231133	BVDV2	2e	B69519c	(de Oliveira et al., 2022)	Npro + E2
MH231149	BVDV2	2e	Short	(de Oliveira et al., 2022)	Npro + E2
KJ608478	BVDV1	1a	NADL	(Chang et al., 2021)	Npro
AJ133739	BVDV1	1a	type 1	(Han et al., 2018)	Npro
FJ387232	BVDV1	1b	NY-1	(Mirosław and Polak, 2019)	Npro
AB359926	BVDV1	1c	Shitara/01/05	(Mirosław and Polak, 2019)	Npro
AY763095	BVDV1	1c	VR1007	(Han et al., 2018)	Npro
JQ071526	BVDV1	1c	GS1	(Mirosław and Polak, 2019)	Npro

1.I: Reference strains used in phylogenetic analysis					
GenBank #	Genotype	Subgenotype	Strain	Reference	Involved tree
U80903	BVDV1	1c	Deer-NZ1	(Han et al., 2018)	Npro
AB359927	BVDV1	1d	OK1(CA)NCP/03	(Mirosław and Polak, 2019)	Npro
AF287284	BVDV1	1d	F	(Mirosław and Polak, 2019)	Npro
AF287282	BVDV1	1e	3186V6	(Chang et al., 2021)	Npro
EU180036	BVDV1	1e	CH-05-02	(Mirosław and Polak, 2019)	Npro
AF287286	BVDV1	1f	J	(Mirosław and Polak, 2019)	Npro
AF287290	BVDV1	1f	W	(Mirosław and Polak, 2019)	Npro
AY323876	BVDV1	1f	G-1703/99-43	(Mirosław and Polak, 2019)	Npro
EU224259	BVDV1	1f	B99/05	(Mirosław and Polak, 2019)	Npro
AF287283	BVDV1	1g	A	(Mirosław and Polak, 2019)	Npro
AF287287	BVDV1	1g	L	(Mirosław and Polak, 2019)	Npro
JN833739	BVDV1	1g	48/08	(Mirosław and Polak, 2019)	Npro
AF287285	BVDV1	1h	G	(Mirosław and Polak, 2019)	Npro
EU180042	BVDV1	1h	CH-95-11	(Mirosław and Polak, 2019)	Npro
AF287279	BVDV1	1i	23/15	(Mirosław and Polak, 2019)	Npro
AB105589	BVDV1	1j	190NCP	(Han et al., 2018)	Npro
U80902	BVDV1	1j	Deer-GB1	(Mirosław and Polak, 2019)	Npro
AY894997	BVDV1	1k	CH-Bohni	(Mirosław and Polak, 2019)	Npro
AY894998	BVDV1	1k	CH-Suwa	(Mirosław and Polak, 2019)	Npro
EU224257	BVDV1	1k	B440/06	(Han et al., 2018)	Npro
EU163975	BVDV1	1l	TR27	(Han et al., 2018)	Npro
EU163977	BVDV1	1l	TR29	(Han et al., 2018)	Npro
GU120262	BVDV1	1m	TJ0801	(Han et al., 2018)	Npro
AB359929	BVDV1	1n	So CP/75	(Mirosław and Polak, 2019)	Npro
AB359930	BVDV1	1n	Shitara/02/06	(Mirosław and Polak, 2019)	Npro
AB359931	BVDV1	1o	IS25CP/01	(Mirosław and Polak, 2019)	Npro
AB359932	BVDV1	1o	IS26NCP/01	(Han et al., 2018)	Npro
KC207073	BVDV1	1o	9	(Han et al., 2018)	Npro
KX218370	BVDV1	1o	HA2-12	(Mirosław and Polak, 2019)	Npro
GU120259	BVDV1	1p	BJ0701	(Mirosław and Polak, 2019)	Npro
GU120260	BVDV1	1p	BJ0702	(Mirosław and Polak, 2019)	Npro
GU120261	BVDV1	1p	BJ0703	(Chang et al., 2021)	Npro
MN417935	BVDV1	1q	T4-23	(Chang et al., 2021)	Npro
KY040425	BVDV1	1r	103/11	(Mirosław and Polak, 2019)	Npro
KY040432	BVDV1	1r	79/11	(Mirosław and Polak, 2019)	Npro
LN515609	BVDV1	1r	CA/181/10	(Chang et al., 2021)	Npro
LN515610	BVDV1	1r	VE/245/12	(Chang et al., 2021)	Npro
LN515612	BVDV1	1s	UM/136/08	(Mirosław and Polak, 2019)	Npro
LN515611	BVDV1	1t	SI/207/12	(Mirosław and Polak, 2019)	Npro
MN417927	BVDV1	1v	EN-7	(Deng et al., 2020)	Npro
MN417928	BVDV1	1v	EN-8	(Deng et al., 2020)	Npro
MN417929	BVDV1	1v	EN-9	(Deng et al., 2020)	Npro
MN417943	BVDV1	1w	T6-18	(Deng et al., 2020)	Npro
MN417944	BVDV1	1w	T6-20	(Deng et al., 2020)	Npro
U18059	BVDV2	2a	890	(de Oliveira et al., 2022, Workman e	Npro
MN954523	BVDV2	2d	354	(de Oliveira et al., 2022)	Npro
MW054933	BVDV1	1f	LA/230/14	(Mosena et al., 2022)	E2

1.I: Reference strains used in phylogenetic analysis

GenBank #	Genotype	Subgenotype	Strain	Reference	Involved tree
MW054936	BVDV1	1g	UM/111/06	(Mosena et al., 2022)	E2
MW655625	BVDV1	1h	CH-04-01b	(Mosena et al., 2022)	E2
MW054935	BVDV1	1k	TO/197/11	(Mosena et al., 2022)	E2
MW655630	BVDV1	1k	R5013-96	(Mosena et al., 2022)	E2
KF048848	BVDV1	1o	TJ41	(Lang et al., 2014)	E2
KF048849	BVDV1	1p	LEI01	(Lang et al., 2014)	E2
LT837585	BVDV1	1r	UNKNOWN-LT8375	(Bazzucchi et al., 2017)	E2

1.II: Summary of the source of subgenotypes		
Source of subgenotypes	Number of strains	Note
Genbank (GB), Npro and E2 Phylogenies	105	Compatible findings from Genbank and phylogeny except for 2 strains that showed divergence in E2 phylogeny as well as update of subgenotype of 8 strains (highlighted with gray).
Genbank and Npro Phylogeny	52	Compatible findings from Genbank and phylogeny except for 4 strains that were also reported in variable subgenotypes by other studies (highlighted with red).
Genbank and E2 Phylogeny	72	Compatible findings from Genbank and phylogeny except 3 strains that were retrieved from VIPRBRC as Pestivirus A, documented in Genbank as 2a and clustered in 2c subgenotype (highlighted with red).
Npro and E2 phylogenies	264	Compatible findings from both phylogenies, except 4 strains that showed divergence in E2 phylogeny as were previously reported in other studies (highlighted with gray).
Npro phylogeny	68	
E2 phylogeny	84	
Genbank	36	Strains with no sequence for the Npro or E2 proteins. Genbank accession of the source are shown.
Total	681	

1.III: Details on subgenotyping of strains according to phylogenetic analysis and/or GenBank

Strain	E2 GB #	Npro GB #	Subgenotype	Subgenotype from GB	Subgenotype from Npro phylogeny	Subgenotype from E2 phylogeny	Source of Subgenotype	Note
Av69 SD-1	KC695815	KC695815	1a	1a	1a	1a	GB + Phyl	Compatible subgenotype
GS5	KJ541471	KJ541471	1a	1a	1a	1a	GB + Phyl	Compatible subgenotype
UNKNOWN-MF693403	MF693403	MF693403	1a	1a	1a	1a	GB + Phyl	Compatible subgenotype
PI34	MN188073	MN188073	1a	1a	1a	1a	GB + Phyl	Compatible subgenotype
62-2	MW250798	MW250798	1a	1a	1a	1a	GB + Phyl	Compatible subgenotype
63-1	MW250799	MW250799	1a	1a	1a	1a	GB + Phyl	Compatible subgenotype
BoAEC1190	MW713361	MW713361	1a	1a	1a	1a	GB + Phyl	Compatible subgenotype
YandaSpl	MW732739	MW732739	1a	1a	1a	1a	GB + Phyl	Compatible subgenotype
Aries	JX297513	JX297513	1b	1b	1b	1b	GB + Phyl	Compatible subgenotype
Columba	JX297514	FJ387237	1b	1b	1b	1b	GB + Phyl	Compatible subgenotype
Corona	JX297515	FJ387238	1b	1b	1b	1b	GB + Phyl	Compatible subgenotype
Gemini	JX297516	FJ387241	1b	1b	1b	1b	GB + Phyl	Compatible subgenotype
Hercules	JX297517	FJ387244	1b	1b	1b	1b	GB + Phyl	Compatible subgenotype
Leo	JX297518	FJ387246	1b	1b	1b	1b	GB + Phyl	Compatible subgenotype
Lyra	JX297519	FJ387248	1b	1b	1b	1b	GB + Phyl	Compatible subgenotype
Mars	JX297520	FJ387249	1b	1b	1b	1b	GB + Phyl	Compatible subgenotype
Scorpius	JX297521	FJ387258	1b	1b	1b	1b	GB + Phyl	Compatible subgenotype
Cepheus	JX306011	FJ387235	1b	1b	1b	1b	GB + Phyl	Compatible subgenotype
Kurhah	JX306013	FJ387245	1b	1b	1b	1b	GB + Phyl	Compatible subgenotype
Av69 VEDEVAC	KC695814	KC695814	1b	1b	1b	1b	GB + Phyl	Compatible subgenotype
12F004	KC963967	KC963967	1b	1b	1b	1b	GB + Phyl	Compatible subgenotype
AU526	KF835697	KF835697	1b	1b	1b	1b	GB + Phyl	Compatible subgenotype
GX4	KJ689448	KJ689448	1b	1b	1b	1b	GB + Phyl	Compatible subgenotype
Egy/Ismailia/2014	KR029825	KR029825	1b	1b	1b	1b	GB + Phyl	Compatible subgenotype
HJ-1	KU756226	KU756226	1b	1b	1b	1b	GB + Phyl	Compatible subgenotype
XZ01	MF278651	MF278651	1b	1b	1b	1b	GB + Phyl	Compatible subgenotype
XZ02	MF278652	MF278652	1b	1b	1b	1b	GB + Phyl	Compatible subgenotype
Nebraska	MH231153	MH231153	1b	1b	1b	1b	GB + Phyl	Compatible subgenotype
BVDV BJ-2016	MH490943	MH490943	1b	1b	1b	1b	GB + Phyl	Compatible subgenotype
PI285	MN188074	MN188074	1b	1b	1b	1b	GB + Phyl	Compatible subgenotype
BVDV 1b IT16/5	MT977117	MT977117	1b	1b	1b	1b	GB + Phyl	Compatible subgenotype
BVDV 1b IT16/439	MT977118	MT977118	1b	1b	1b	1b	GB + Phyl	Compatible subgenotype
PI819	MW713362	MW713362	1b	1b	1b	1b	GB + Phyl	Compatible subgenotype
Bega-like	KC695813	KC695813	1c	1c	1c	1c	GB + Phyl	Compatible subgenotype
NM2103	ON337882	ON337882	1c	1c	1c	1c	GB + Phyl	Compatible subgenotype
BJ1201	KT943518	KT943518	1d	1d	1d	1d	GB + Phyl	Compatible subgenotype
cell-con-1	KC695816	KC695816	1d	1d	1d	1d	GB + Phyl	Compatible subgenotype
SLO/2416/2002	KY849592	KY849592	1d	1d	1d	1d	GB + Phyl	Compatible subgenotype
67-1	MW250800	MW250800	1d	1d	1d	1d	GB + Phyl	Compatible subgenotype
67-2	MW250801	MW250801	1d	1d	1d	1d	GB + Phyl	Compatible subgenotype
Carlito	KP313732	KP313732	1e	1e	1e	1e	GB + Phyl	Compatible subgenotype
MA/101/05	MW054940	MW054940	1e	1e	1e	1e	GB + Phyl	Compatible subgenotype
68-1	MW250802	MW250802	1e	1e	1e	1e	GB + Phyl	Compatible subgenotype
Maria	MW655626	MW655626	1e	1e	1e	1e	GB + Phyl	Compatible subgenotype
R2000-95	MW655627	MW655627	1e	1e	1e	1e	GB + Phyl	Compatible subgenotype
S03-1175	MW655631	MW655631	1e	1e	1e	1e	GB + Phyl	Compatible subgenotype
LA/230/14	MW054933	MW054933	1f	1f	1f	1f	GB + Phyl	Compatible subgenotype
LA/87/05	MW054934	MW054934	1f	1f	1f	1f	GB + Phyl	Compatible subgenotype
LO/151/09	MW054939	MW054939	1f	1f	1f	1f	GB + Phyl	Compatible subgenotype
UM/126/07	LT631725	LT631725	1h	1h	1h	1h	GB + Phyl	Compatible subgenotype
CH-04-01b	MW655625	MW655625	1h	1h	1h	1h	GB + Phyl	Compatible subgenotype
R3572-90	MW655629	MW655629	1h	1h	1h	1h	GB + Phyl	Compatible subgenotype
SM09-20	MW655632	MW655632	1h	1h	1h	1h	GB + Phyl	Compatible subgenotype
58-1	MW250796	MW250796	1i	1i	1i	1i	GB + Phyl	Compatible subgenotype
58-2	MW250797	MW250797	1i	1i	1i	1i	GB + Phyl	Compatible subgenotype
69-1	MW250803	MW250803	1i	1i	1i	1i	GB + Phyl	Compatible subgenotype
SuwaCp	KC853441	KC853441	1k	1k	1k	1k	GB + Phyl	Compatible subgenotype
SA/158/09	MW054937	MW054937	1k	1k	1k	1k	GB + Phyl	Compatible subgenotype

1.III: Details on subgenotyping of strains according to phylogenetic analysis and/or GenBank

Strain	E2 GB #	Npro GB #	Subgenotype	Subgenotype from GB	Subgenotype from Npro phylogeny	Subgenotype from E2 phylogeny	Source of Subgenotype	Note
R3230-95	MW655628	MW655628	1k	1k	1k	1k	GB + Phyl	Compatible subgenotype
R5013-96	MW655630	MW655630	1k	1k	1k	1k	GB + Phyl	Compatible subgenotype
SuwaNcp	KC853440	KC853440	1k	1k	1k	1k	GB + Phyl	Compatible subgenotype
SA/159/09	MW054938	MW054938	1k	1k	1k	1k	GB + Phyl	Compatible subgenotype
SD-15	KR866116	KR866116	1m	1m	1m	1m	GB + Phyl	Compatible subgenotype
NX2019/01	MN623291	MN623291	1m	1m	1m	1m	GB + Phyl	Compatible subgenotype
GS-3	KC695811	KC695811	1q	1q	1q	1q	GB + Phyl	Compatible subgenotype
11F011	KC963968	KC963968	2a	2a	2a	2a	GB + Phyl	Compatible subgenotype
125c	MH806434	MH806434	2a	2a	2a	2a	GB + Phyl	Compatible subgenotype
296c	MH806436	MH806436	2a	2a	2a	2a	GB + Phyl	Compatible subgenotype
53637c	MH231127	MH231127	2a	2a	2a	2a	GB + Phyl	Compatible subgenotype
5912c	MH231129	MH231129	2a	2a	2a	2a	GB + Phyl	Compatible subgenotype
95-1501	MH231130	MH231130	2a	2a	2a	2a	GB + Phyl	Compatible subgenotype
AU501	MH231131	MH231131	2a	2a	2a	2a	GB + Phyl	Compatible subgenotype
B9497	MH231134	MH231134	2a	2a	2a	2a	GB + Phyl	Compatible subgenotype
BV1907	MH231135	MH231135	2a	2a	2a	2a	GB + Phyl	Compatible subgenotype
CPAE_contamination/2018	MN824468	MN824468	2a	2a	2a	2a	GB + Phyl	Compatible subgenotype
JV14	MH231136	MH231136	2a	2a	2a	2a	GB + Phyl	Compatible subgenotype
MadSpl	MH231137	MH231137	2a	2a	2a	2a	GB + Phyl	Compatible subgenotype
McCart_c	MH806438	MH806438	2a	2a	2a	2a	GB + Phyl	Compatible subgenotype
MnFetus	MH231138	MH231138	2a	2a	2a	2a	GB + Phyl	Compatible subgenotype
Olwein #12	MH231139	MH231139	2a	2a	2a	2a	GB + Phyl	Compatible subgenotype
PA	MH231140	MH231140	2a	2a	2a	2a	GB + Phyl	Compatible subgenotype
PI28	MH231141	MH231141	2a	2a	2a	2a	GB + Phyl	Compatible subgenotype
RS886	MH231143	MH231143	2a	2a	2a	2a	GB + Phyl	Compatible subgenotype
Sanderson6319	MH231144	MH231144	2a	2a	2a	2a	GB + Phyl	Compatible subgenotype
Victor301	MH231145	MH231145	2a	2a	2a	2a	GB + Phyl	Compatible subgenotype
WiscA	MH231146	MH231146	2a	2a	2a	2a	GB + Phyl	Compatible subgenotype
**ACM/BR/2016**	**KX857724**	**KX857724**	**1i**	**1i**	**1i**	**1i**	**Ref Strain**	Compatible subgenotype
**CA2006**	**MK775204**	**MK775204**	**1i**	**1i**	**1i**	**1i**	**Ref Strain**	Compatible subgenotype
**HB-1511**	**KX096718**	**KX096718**	**2a**	2a	2a	2a	**Ref Strain**	Compatible subgenotype
**NY-93**	**KR093034**	**KR093034**	**2a**	2a	2a	2a	**Ref Strain**	Compatible subgenotype
**HB-1**	**KC695812**	**KC695812**	**1q**	**1q**	**1q**	**1q**	**Ref Strain**	Compatible subgenotype
**10JJ-SKR**	**KC757383**	**KC757383**	**1d**	**1d**	**1d**	**1d**	**Ref Strain**	Compatible subgenotype
**SLO/2407/2006**	**KX577637**	**KX577637**	**1e**	**1e**	**1e**	**1e**	**Ref Strain**	Compatible subgenotype
**XC**	**MH166806**	**MH166806**	**1m**	**1m**	**1m**	**1m**	**Ref Strain**	Compatible subgenotype
**camel-6**	**KC695810**	**KC695810**	**1q**	**1q**	**1q**	**1q**	**Ref Strain**	Compatible subgenotype
AzSpl	MH231132	MH231132	2a	2a	2a	??	GB + Phyl	Compatible subgenotype in GB and Npro phylogeny, E2 show divergence (??) and discarded, seq most similar to 2a (similar to (de Oliveira et al., 2022))
1336H	MH806435	MH806435	2a	2a	2a	2c	GB + Phyl	Compatible subgenotype in GB and Npro phylogeny, E2 show divergence (2c) and discarded, seq most similar to 2a (similar to (de Oliveira et al., 2022))
PI407	MW732738	MW732738	1a	1b	1a	1a	Phyl	incompatible with Npro and E2 phylogenies (both A), GB result was discarded, seq was most similar to 1a, (similar finding was reported by (FALKENBERG et al., 2021))
Parker	MH231142	MH231142	2c	2a	2c	2c	Phyl	incompatible with Npro and E2 phylogenies (2c), GB result was discarded, seq was most similar to 2c (similar finding was obtained by (de Oliveira et al., 2022))
PI12	MH231147	MH231147	2c	2a	2c	2c	Phyl	incompatible with Npro and E2 phylogenies (2c), GB result was discarded, seq wa most similar to 2c (similar finding was reported by (de Oliveira et al., 2022))
1786c	**MH231124**	**MH231124**	**2e**	2c	2e	2e	GB + Phyl	Compatible subgenotype in phylogeny, 2c in Genbank (old)

1.III: Details on subgenotyping of strains according to phylogenetic analysis and/or GenBank

Strain	E2 GB #	Npro GB #	Subgenotype	Subgenotype from GB	Subgenotype from Npro phylogeny	Subgenotype from E2 phylogeny	Source of Subgenotype	Note
**B69519c**	**MH231133**	**MH231133**	**2e**	2c	2e	2e	**Ref Strain**	Compatible subgenotype in phylogeny, 2c in Genbank (old)
12-149150	**MH231148**	**MH231148**	**2e**	2c	2e	2e	GB + Phyl	Compatible subgenotype in phylogeny, 2c in Genbank (old)
**Short**	**MH231149**	**MH231149**	**2e**	2c	2e	2e	**Ref Strain**	Compatible subgenotype in phylogeny, 2c in Genbank (old)
12-151955-317	**MH231150**	**MH231150**	**2e**	2c	2e	2e	GB + Phyl	Compatible subgenotype in phylogeny, 2c in Genbank (old)
1	#N/A	KC207068	1a	1a	1a	#N/A	GB + Phyl	Compatible subgenotype
2	#N/A	KC207069	1a	1a	1a	#N/A	GB + Phyl	Compatible subgenotype
ABART-2	#N/A	MT024564	1a	1a	1a	#N/A	GB + Phyl	Compatible subgenotype
Bootes	#N/A	FJ387233	1b	1b	1b	#N/A	GB + Phyl	Compatible subgenotype
Camelo	#N/A	FJ387234	1b	1b	1b	#N/A	GB + Phyl	Compatible subgenotype
Chara	#N/A	FJ387236	1b	1b	1b	#N/A	GB + Phyl	Compatible subgenotype
Dorado	#N/A	FJ387239	1b	1b	1b	#N/A	GB + Phyl	Compatible subgenotype
Draco	#N/A	FJ387240	1b	1b	1b	#N/A	GB + Phyl	Compatible subgenotype
Gomeisa	#N/A	FJ387242	1b	1b	1b	#N/A	GB + Phyl	Compatible subgenotype
Hamel	#N/A	FJ387243	1b	1b	1b	#N/A	GB + Phyl	Compatible subgenotype
Libra	#N/A	FJ387247	1b	1b	1b	#N/A	GB + Phyl	Compatible subgenotype
Mensa	#N/A	FJ387250	1b	1b	1b	#N/A	GB + Phyl	Compatible subgenotype
Mercury	#N/A	FJ387251	1b	1b	1b	#N/A	GB + Phyl	Compatible subgenotype
Musca	#N/A	FJ387252	1b	1b	1b	#N/A	GB + Phyl	Compatible subgenotype
Nakkar	#N/A	FJ387253	1b	1b	1b	#N/A	GB + Phyl	Compatible subgenotype
Pegasus	#N/A	FJ387254	1b	1b	1b	#N/A	GB + Phyl	Compatible subgenotype
Pluto	#N/A	FJ387255	1b	1b	1b	#N/A	GB + Phyl	Compatible subgenotype
Sadaton	#N/A	FJ387256	1b	1b	1b	#N/A	GB + Phyl	Compatible subgenotype
Saturn	#N/A	FJ387257	1b	1b	1b	#N/A	GB + Phyl	Compatible subgenotype
Sirrah	#N/A	FJ387259	1b	1b	1b	#N/A	GB + Phyl	Compatible subgenotype
Taurus	#N/A	FJ387260	1b	1b	1b	#N/A	GB + Phyl	Compatible subgenotype
Tucan	#N/A	FJ387261	1b	1b	1b	#N/A	GB + Phyl	Compatible subgenotype
Venus	#N/A	FJ387262	1b	1b	1b	#N/A	GB + Phyl	Compatible subgenotype
Virgo	#N/A	FJ387263	1b	1b	1b	#N/A	GB + Phyl	Compatible subgenotype
Eridanus	#N/A	FJ387264	1b	1b	1b	#N/A	GB + Phyl	Compatible subgenotype
12	#N/A	KC207076	1b	1b	1b	#N/A	GB + Phyl	Compatible subgenotype
Dari85	#N/A	KC414584	1b	1b	1b	#N/A	GB + Phyl	Compatible subgenotype
Zeku33	#N/A	KC414585	1b	1b	1b	#N/A	GB + Phyl	Compatible subgenotype
Dari98	#N/A	KC414586	1b	1b	1b	#N/A	GB + Phyl	Compatible subgenotype
Zeku26	#N/A	KC414587	1b	1b	1b	#N/A	GB + Phyl	Compatible subgenotype
GS-4	#N/A	KC700344	1b	1b	1b	#N/A	GB + Phyl	Compatible subgenotype
190919	#N/A	MT024562	1b	1b	1b	#N/A	GB + Phyl	Compatible subgenotype
230919	#N/A	MT024563	1b	1b	1b	#N/A	GB + Phyl	Compatible subgenotype
GS2	#N/A	JQ071528	1c	1c	1c	#N/A	GB + Phyl	Compatible subgenotype
3	#N/A	KC207070	1c	1c	1c	#N/A	GB + Phyl	Compatible subgenotype
DulanD62	#N/A	KC414589	1d	1d	1d	#N/A	GB + Phyl	Compatible subgenotype
DulanD64	#N/A	KC414590	1d	1d	1d	#N/A	GB + Phyl	Compatible subgenotype
Yushu2158	#N/A	KC414594	1d	1d	1d	#N/A	GB + Phyl	Compatible subgenotype
Yushu2121	#N/A	KC414595	1d	1d	1d	#N/A	GB + Phyl	Compatible subgenotype
DulanD44	#N/A	KC414596	1d	1d	1d	#N/A	GB + Phyl	Compatible subgenotype
TR72	#N/A	KF154776	1l	1l	1l	#N/A	GB + Phyl	Compatible subgenotype
11	#N/A	KC207075	1m	1m	1m	#N/A	GB + Phyl	Compatible subgenotype
0001	#N/A	MN394766	1m	1m	1m	#N/A	GB + Phyl	Compatible subgenotype
5	#N/A	KC207071	1p	1p	1p	#N/A	GB + Phyl	Compatible subgenotype
6	#N/A	KC207072	1q	1q	1q	#N/A	GB + Phyl	Compatible subgenotype
NX2019/02	#N/A	MT740275	1v	1v	1v	#N/A	GB + Phyl	Compatible subgenotype
**NY-1**	**#N/A**	**FJ387232**	**1b**	**1b**	**1b**	**#N/A**	**Ref Strain**	
**GS1**	**#N/A**	**JQ071526**	**1c**	**1c**	**1c**	**#N/A**	**Ref Strain**	

1.III: Details on subgenotyping of strains according to phylogenetic analysis and/or GenBank

Strain	E2 GB #	Npro GB #	Subgenotype	Subgenotype from GB	Subgenotype from Npro phylogeny	Subgenotype from E2 phylogeny	Source of Subgenotype	Note
TR73	#N/A	KF154777	1p	1p	1j	#N/A	GB	incompatible with Npro phylogeny (j), Npro results were discarded (seq was most similar to 1g, 1a (also designated as 1r by (Yeşilbağ et al., 2017)
TR75	#N/A	KF154778	1p	1p	1j	#N/A	GB	incompatible with Npro phylogeny (j), Npro results were discarded (seq was most similar to 1g, 1a (also designated as 1r by (Yeşilbağ et al., 2017)
TR70	#N/A	KF154779	1p	1p	1j	#N/A	GB	incompatible with Npro phylogeny (j), Npro results were discarded (seq was most similar to 1g, 1a (also designated as 1r by (Yeşilbağ et al., 2017)
9	**#N/A**	**KC207073**	**1o**	**1o**	**?**	**#N/A**	**Ref Strain**	Ref seq of 1o (Han et al., 2018), showed divergent in Npro tree, seq was most similar to 1g
GS24	KF048838	#N/A	1a	1a	#N/A	1a	GB + Phyl	Compatible subgenotype
M15.1	JN377417	#N/A	1b	1b	#N/A	1b	GB + Phyl	Compatible subgenotype
M15.2	JN377418	#N/A	1b	1b	#N/A	1b	GB + Phyl	Compatible subgenotype
M15.3	JN377419	#N/A	1b	1b	#N/A	1b	GB + Phyl	Compatible subgenotype
M15.4	JN377420	#N/A	1b	1b	#N/A	1b	GB + Phyl	Compatible subgenotype
M15.5	JN377421	#N/A	1b	1b	#N/A	1b	GB + Phyl	Compatible subgenotype
M15.6	JN377422	#N/A	1b	1b	#N/A	1b	GB + Phyl	Compatible subgenotype
M15.7	JN377423	#N/A	1b	1b	#N/A	1b	GB + Phyl	Compatible subgenotype
M181 22	JN377424	#N/A	1b	1b	#N/A	1b	GB + Phyl	Compatible subgenotype
USF02	KF048835	#N/A	1b	1b	#N/A	1b	GB + Phyl	Compatible subgenotype
QUAN2	KF048836	#N/A	1b	1b	#N/A	1b	GB + Phyl	Compatible subgenotype
GS151	KF048837	#N/A	1b	1b	#N/A	1b	GB + Phyl	Compatible subgenotype
DX51	KF048839	#N/A	1b	1b	#N/A	1b	GB + Phyl	Compatible subgenotype
USF12	KF048840	#N/A	1b	1b	#N/A	1b	GB + Phyl	Compatible subgenotype
SJZ01	KF048841	#N/A	1b	1b	#N/A	1b	GB + Phyl	Compatible subgenotype
GS31	KF048853	#N/A	1b	1b	#N/A	1b	GB + Phyl	Compatible subgenotype
BVDV/Taiki/I_22	LC648252	#N/A	1b	1b	#N/A	1b	GB + Phyl	Compatible subgenotype
BVDV/Taiki/A_3	LC648254	#N/A	1b	1b	#N/A	1b	GB + Phyl	Compatible subgenotype
BVDV/Taiki/A_2	LC648255	#N/A	1b	1b	#N/A	1b	GB + Phyl	Compatible subgenotype
BVDV/Taiki/A_1	LC648256	#N/A	1b	1b	#N/A	1b	GB + Phyl	Compatible subgenotype
BVDV/Shihoro/O_40	LC648258	#N/A	1b	1b	#N/A	1b	GB + Phyl	Compatible subgenotype
BVDV/Shihoro/O_39	LC648259	#N/A	1b	1b	#N/A	1b	GB + Phyl	Compatible subgenotype
BVDV/Shihoro/B_41	LC648260	#N/A	1b	1b	#N/A	1b	GB + Phyl	Compatible subgenotype
BVDV/Shihoro/B_6	LC648261	#N/A	1b	1b	#N/A	1b	GB + Phyl	Compatible subgenotype
BVDV/Otohuke/J_26	LC648263	#N/A	1b	1b	#N/A	1b	GB + Phyl	Compatible subgenotype
BVDV/Otohuke/J_25	LC648264	#N/A	1b	1b	#N/A	1b	GB + Phyl	Compatible subgenotype
BVDV/Otohuke/J_23	LC648265	#N/A	1b	1b	#N/A	1b	GB + Phyl	Compatible subgenotype
BVDV/Kamishihoro/L_29	LC648266	#N/A	1b	1b	#N/A	1b	GB + Phyl	Compatible subgenotype
BVDV/Kamishihoro/L_28	LC648267	#N/A	1b	1b	#N/A	1b	GB + Phyl	Compatible subgenotype
BVDV/Honbetu/M_33	LC648268	#N/A	1b	1b	#N/A	1b	GB + Phyl	Compatible subgenotype
BVDV/Honbetu/M_30	LC648269	#N/A	1b	1b	#N/A	1b	GB + Phyl	Compatible subgenotype
BVDV/Hiroo/G_20	LC648270	#N/A	1b	1b	#N/A	1b	GB + Phyl	Compatible subgenotype
BVDV/Hiroo/G_19	LC648271	#N/A	1b	1b	#N/A	1b	GB + Phyl	Compatible subgenotype
BVDV/Hiroo/G_18	LC648272	#N/A	1b	1b	#N/A	1b	GB + Phyl	Compatible subgenotype
BVDV/Hiroo/G_17	LC648273	#N/A	1b	1b	#N/A	1b	GB + Phyl	Compatible subgenotype
BVDV/Hiroo/E_14	LC648274	#N/A	1b	1b	#N/A	1b	GB + Phyl	Compatible subgenotype
BVDV/Hiroo/D_12	LC648275	#N/A	1b	1b	#N/A	1b	GB + Phyl	Compatible subgenotype
BVDV/Hiroo/D_13	LC648276	#N/A	1b	1b	#N/A	1b	GB + Phyl	Compatible subgenotype
BVDV/Hiroo/D_10	LC648277	#N/A	1b	1b	#N/A	1b	GB + Phyl	Compatible subgenotype
BVDV/Hiroo/D_9	LC648278	#N/A	1b	1b	#N/A	1b	GB + Phyl	Compatible subgenotype
BVDV/Hiroo/D_8	LC648279	#N/A	1b	1b	#N/A	1b	GB + Phyl	Compatible subgenotype
BVDV/Rikubetsu/1283/14	LC648387	#N/A	1b	1b	#N/A	1b	GB + Phyl	Compatible subgenotype
BVDV/Rikubetsu/1284/14	LC648388	#N/A	1b	1b	#N/A	1b	GB + Phyl	Compatible subgenotype
BVDV/Makubetsu/1294/1	LC648389	#N/A	1b	1b	#N/A	1b	GB + Phyl	Compatible subgenotype
BVDV/Oozora/1331/16	LC648390	#N/A	1b	1b	#N/A	1b	GB + Phyl	Compatible subgenotype
BVDV/Obihiro/1364/16	LC648391	#N/A	1b	1b	#N/A	1b	GB + Phyl	Compatible subgenotype
BVDV/Kamishihoro/1385/	LC648392	#N/A	1b	1b	#N/A	1b	GB + Phyl	Compatible subgenotype

1.III: Details on subgenotyping of strains according to phylogenetic analysis and/or GenBank

Strain	E2 GB #	Npro GB #	Subgenotype	Subgenotype from GB	Subgenotype from Npro phylogeny	Subgenotype from E2 phylogeny	Source of Subgenotype	Note
BVDV/Sarabetsu/1517/17	LC648393	#N/A	1b	1b	#N/A	1b	GB + Phyl	Compatible subgenotype
BVDV/Teshio/1600/17	LC648394	#N/A	1b	1b	#N/A	1b	GB + Phyl	Compatible subgenotype
BVDV/Saruhutsu/1607/17	LC648395	#N/A	1b	1b	#N/A	1b	GB + Phyl	Compatible subgenotype
BVDV/Setana/1733/17	LC648396	#N/A	1b	1b	#N/A	1b	GB + Phyl	Compatible subgenotype
BVDV/Hokuto/1735/18	LC648397	#N/A	1b	1b	#N/A	1b	GB + Phyl	Compatible subgenotype
BVDV/Shihoro/1754/18	LC648398	#N/A	1b	1b	#N/A	1b	GB + Phyl	Compatible subgenotype
BVDV/Nayoro/1857/19	LC648399	#N/A	1b	1b	#N/A	1b	GB + Phyl	Compatible subgenotype
BVDV/Bie/1860/20	LC648400	#N/A	1b	1b	#N/A	1b	GB + Phyl	Compatible subgenotype
BVDV/Higashikagura/1862	LC648401	#N/A	1b	1b	#N/A	1b	GB + Phyl	Compatible subgenotype
GS35	KF048842	#N/A	1c	1c	#N/A	1c	GB + Phyl	Compatible subgenotype
JINAN01	KF048843	#N/A	1c	1c	#N/A	1c	GB + Phyl	Compatible subgenotype
ISO122	KF048844	#N/A	1d	1d	#N/A	1d	GB + Phyl	Compatible subgenotype
SH202	KF048845	#N/A	1m	1m	#N/A	1m	GB + Phyl	Compatible subgenotype
TJ43	KF048846	#N/A	1m	1m	#N/A	1m	GB + Phyl	Compatible subgenotype
HZ21	KF048847	#N/A	1m	1m	#N/A	1m	GB + Phyl	Compatible subgenotype
TJ41	KF048848	#N/A	1o	1o	#N/A	1o	GB + Phyl	Compatible subgenotype
LEI01	KF048849	#N/A	1p	1p	#N/A	1p	GB + Phyl	Compatible subgenotype
TJ142	KF048850	#N/A	1p	1p	#N/A	1p	GB + Phyl	Compatible subgenotype
3877	MW013505	#N/A	1p	1p	#N/A	1p	GB + Phyl	Compatible subgenotype
ISO101	KF048851	#N/A	1q	1q	#N/A	1q	GB + Phyl	Compatible subgenotype
USF10	KF048852	#N/A	2a	2a	#N/A	2a	GB + Phyl	Compatible subgenotype, retrieved as pestivirus A
MS12	JN387139	#N/A	2a	2a	#N/A	2a	GB + Phyl	Compatible subgenotype
BVDV/Taiki/A_4	LC648253	#N/A	2c	2a	#N/A	2c	Phyl	Incompatible subgenotype with GB, result of GB was discarded, retrieved as pestivirus A
BVDV/Shintoku/F_16	LC648257	#N/A	2c	2a	#N/A	2c	Phyl	Incompatible subgenotype with GB, result of GB was discarded, retrieved as pestivirus A
BVDV/Shihoro/B_5	LC648262	#N/A	2c	2a	#N/A	2c	Phyl	Incompatible subgenotype with GB, result of GB was discarded, retrieved as pestivirus A
**Shitara/02/06**	**LC089876**	**LC089876**	**1n**	#N/A	**1n**	**1n**	**Ref Strain**	Compatible subgenotype
Oregon	AF041040	AF041040	1a	#N/A	1a	1a	Phyl	Compatible subgenotype
Oregon C24V	AF091605	AF091605	1a	#N/A	1a	1a	Phyl	Compatible subgenotype
180	HQ174292	HQ174292	1a	#N/A	1a	1a	Phyl	Compatible subgenotype
6010	JN380080	JN380080	1a	#N/A	1a	1a	Phyl	Compatible subgenotype
USMARC-53875	KP941584	KP941584	1a	#N/A	1a	1a	Phyl	Compatible subgenotype
USMARC-55477	KP941586	KP941586	1a	#N/A	1a	1a	Phyl	Compatible subgenotype
WAX-N	KR013753	KR013753	1a	#N/A	1a	1a	Phyl	Compatible subgenotype
USII-S15	KU159365	KU159365	1a	#N/A	1a	1a	Phyl	Compatible subgenotype
V026	KX170157	KX170342	1a	#N/A	1a	1a	Phyl	Compatible subgenotype
V027	KX170158	KX170343	1a	#N/A	1a	1a	Phyl	Compatible subgenotype
V091	KX170159	KX170345	1a	#N/A	1a	1a	Phyl	Compatible subgenotype
V007	KX170160	KX170349	1a	#N/A	1a	1a	Phyl	Compatible subgenotype
V013	KX170161	KX170350	1a	#N/A	1a	1a	Phyl	Compatible subgenotype
V033	KX170162	KX170351	1a	#N/A	1a	1a	Phyl	Compatible subgenotype
V034	KX170163	KX170352	1a	#N/A	1a	1a	Phyl	Compatible subgenotype
V067	KX170164	KX170353	1a	#N/A	1a	1a	Phyl	Compatible subgenotype
V074	KX170165	KX170354	1a	#N/A	1a	1a	Phyl	Compatible subgenotype
V073	KX170166	KX170348	1a	#N/A	1a	1a	Phyl	Compatible subgenotype
V049	KX170167	KX170346	1a	#N/A	1a	1a	Phyl	Compatible subgenotype
V077	KX170168	KX170347	1a	#N/A	1a	1a	Phyl	Compatible subgenotype
V080	KX170169	KX170344	1a	#N/A	1a	1a	Phyl	Compatible subgenotype
V083	KX170170	KX170324	1a	#N/A	1a	1a	Phyl	Compatible subgenotype
V022	KX170171	KX170322	1a	#N/A	1a	1a	Phyl	Compatible subgenotype
V014	KX170172	KX170323	1a	#N/A	1a	1a	Phyl	Compatible subgenotype
V092	KX170173	KX170315	1a	#N/A	1a	1a	Phyl	Compatible subgenotype
V054	KX170174	KX170316	1a	#N/A	1a	1a	Phyl	Compatible subgenotype
V011	KX170175	KX170320	1a	#N/A	1a	1a	Phyl	Compatible subgenotype
V012	KX170176	KX170321	1a	#N/A	1a	1a	Phyl	Compatible subgenotype
V056	KX170177	KX170318	1a	#N/A	1a	1a	Phyl	Compatible subgenotype

1.III: Details on subgenotyping of strains according to phylogenetic analysis and/or GenBank

Strain	E2 GB #	Npro GB #	Subgenotype	Subgenotype from GB	Subgenotype from Npro phylogeny	Subgenotype from E2 phylogeny	Source of Subgenotype	Note
V057	KX170178	KX170317	1a	#N/A	1a	1a	Phyl	Compatible subgenotype
V006	KX170179	KX170319	1a	#N/A	1a	1a	Phyl	Compatible subgenotype
V001	KX170180	KX170328	1a	#N/A	1a	1a	Phyl	Compatible subgenotype
V016	KX170181	KX170329	1a	#N/A	1a	1a	Phyl	Compatible subgenotype
V010	KX170182	KX170332	1a	#N/A	1a	1a	Phyl	Compatible subgenotype
V008	KX170183	KX170330	1a	#N/A	1a	1a	Phyl	Compatible subgenotype
V009	KX170184	KX170331	1a	#N/A	1a	1a	Phyl	Compatible subgenotype
V040	KX170185	KX170339	1a	#N/A	1a	1a	Phyl	Compatible subgenotype
V035	KX170186	KX170333	1a	#N/A	1a	1a	Phyl	Compatible subgenotype
V042	KX170187	KX170336	1a	#N/A	1a	1a	Phyl	Compatible subgenotype
V043	KX170188	KX170337	1a	#N/A	1a	1a	Phyl	Compatible subgenotype
V052	KX170189	KX170341	1a	#N/A	1a	1a	Phyl	Compatible subgenotype
V050	KX170190	KX170338	1a	#N/A	1a	1a	Phyl	Compatible subgenotype
V046	KX170191	KX170340	1a	#N/A	1a	1a	Phyl	Compatible subgenotype
V039	KX170192	KX170334	1a	#N/A	1a	1a	Phyl	Compatible subgenotype
V041	KX170193	KX170335	1a	#N/A	1a	1a	Phyl	Compatible subgenotype
V099	KX170194	KX170327	1a	#N/A	1a	1a	Phyl	Compatible subgenotype
V048	KX170195	KX170325	1a	#N/A	1a	1a	Phyl	Compatible subgenotype
V059	KX170196	KX170326	1a	#N/A	1a	1a	Phyl	Compatible subgenotype
UNKNOWN-M31182	M31182	M31182	1a	#N/A	1a	1a	Phyl	Compatible subgenotype
Ho916	MH379638	MH379638	1a	#N/A	1a	1a	Phyl	Compatible subgenotype
20-8536	MT654137	MT654137	1a	#N/A	1a	1a	Phyl	Compatible subgenotype
Hamal	JX306012	JX306012	1b	#N/A	1b	1b	Phyl	Compatible subgenotype
Naos	JX306014	JX306014	1b	#N/A	1b	1b	Phyl	Compatible subgenotype
BSU1	JF968611	GU991550	1b	#N/A	1b	1b	Phyl	Compatible subgenotype
3156	JN644055	JN644055	1b	#N/A	1b	1b	Phyl	Compatible subgenotype
10270	JX297512	JX297512	1b	#N/A	1b	1b	Phyl	Compatible subgenotype
UNKNOWN-JX419397	JX419397	JX419397	1b	#N/A	1b	1b	Phyl	Compatible subgenotype
UNKNOWN-JX419398	JX419398	JX419398	1b	#N/A	1b	1b	Phyl	Compatible subgenotype
BVDV JL-1	KF501393	KF501393	1b	#N/A	1b	1b	Phyl	Compatible subgenotype
CC13B	KF772785	KF772785	1b	#N/A	1b	1b	Phyl	Compatible subgenotype
USMARC-51998	KP941581	KP941581	1b	#N/A	1b	1b	Phyl	Compatible subgenotype
USMARC-53874	KP941583	KP941583	1b	#N/A	1b	1b	Phyl	Compatible subgenotype
USMARC-55478	KP941587	KP941587	1b	#N/A	1b	1b	Phyl	Compatible subgenotype
USMARC-55922	KP941588	KP941588	1b	#N/A	1b	1b	Phyl	Compatible subgenotype
USMARC-55923	KP941589	KP941589	1b	#N/A	1b	1b	Phyl	Compatible subgenotype
USMARC-55924	KP941590	KP941590	1b	#N/A	1b	1b	Phyl	Compatible subgenotype
USMARC-55925	KP941591	KP941591	1b	#N/A	1b	1b	Phyl	Compatible subgenotype
USMARC-55926	KP941592	KP941592	1b	#N/A	1b	1b	Phyl	Compatible subgenotype
BE/061536/2014	KU200260	KU200260	1b	#N/A	1b	1b	Phyl	Compatible subgenotype
V015	KX170144	KX170302	1b	#N/A	1b	1b	Phyl	Compatible subgenotype
V100	KX170145	KX170305	1b	#N/A	1b	1b	Phyl	Compatible subgenotype
V075	KX170146	KX170304	1b	#N/A	1b	1b	Phyl	Compatible subgenotype
V060	KX170147	KX170303	1b	#N/A	1b	1b	Phyl	Compatible subgenotype
V070	KX170148	KX170307	1b	#N/A	1b	1b	Phyl	Compatible subgenotype
V036	KX170149	KX170306	1b	#N/A	1b	1b	Phyl	Compatible subgenotype
V098	KX170150	KX170308	1b	#N/A	1b	1b	Phyl	Compatible subgenotype
V020	KX170151	KX170309	1b	#N/A	1b	1b	Phyl	Compatible subgenotype
V029	KX170152	KX170310	1b	#N/A	1b	1b	Phyl	Compatible subgenotype
V045	KX170153	KX170311	1b	#N/A	1b	1b	Phyl	Compatible subgenotype
V078	KX170154	KX170312	1b	#N/A	1b	1b	Phyl	Compatible subgenotype
V031	KX170155	KX170313	1b	#N/A	1b	1b	Phyl	Compatible subgenotype
V087	KX170156	KX170314	1b	#N/A	1b	1b	Phyl	Compatible subgenotype
Y2	KY964311	KY964311	1b	#N/A	1b	1b	Phyl	Compatible subgenotype
B1	MG950345	MG950345	1b	#N/A	1b	1b	Phyl	Compatible subgenotype
B2	MG950346	MG950346	1b	#N/A	1b	1b	Phyl	Compatible subgenotype
B3	MG950347	MG950347	1b	#N/A	1b	1b	Phyl	Compatible subgenotype
B4	MG950348	MG950348	1b	#N/A	1b	1b	Phyl	Compatible subgenotype

1.III: Details on subgenotyping of strains according to phylogenetic analysis and/or GenBank

Strain	E2 GB #	Npro GB #	Subgenotype	Subgenotype from GB	Subgenotype from Npro phylogeny	Subgenotype from E2 phylogeny	Source of Subgenotype	Note
B5	MG950349	MG950349	1b	#N/A	1b	1b	Phyl	Compatible subgenotype
B6	MG950350	MG950350	1b	#N/A	1b	1b	Phyl	Compatible subgenotype
O1	MG950351	MG950351	1b	#N/A	1b	1b	Phyl	Compatible subgenotype
O2	MG950352	MG950352	1b	#N/A	1b	1b	Phyl	Compatible subgenotype
O3	MG950353	MG950353	1b	#N/A	1b	1b	Phyl	Compatible subgenotype
O4	MG950354	MG950354	1b	#N/A	1b	1b	Phyl	Compatible subgenotype
O5	MG950355	MG950355	1b	#N/A	1b	1b	Phyl	Compatible subgenotype
O6	MG950356	MG950356	1b	#N/A	1b	1b	Phyl	Compatible subgenotype
B1A	MG950357	MG950357	1b	#N/A	1b	1b	Phyl	Compatible subgenotype
B2A	MG950358	MG950358	1b	#N/A	1b	1b	Phyl	Compatible subgenotype
B3A	MG950359	MG950359	1b	#N/A	1b	1b	Phyl	Compatible subgenotype
B4A	MG950360	MG950360	1b	#N/A	1b	1b	Phyl	Compatible subgenotype
B5A	MG950361	MG950361	1b	#N/A	1b	1b	Phyl	Compatible subgenotype
B6A	MG950362	MG950362	1b	#N/A	1b	1b	Phyl	Compatible subgenotype
O1A	MG950363	MG950363	1b	#N/A	1b	1b	Phyl	Compatible subgenotype
O2A	MG950364	MG950364	1b	#N/A	1b	1b	Phyl	Compatible subgenotype
O2B	MG950365	MG950365	1b	#N/A	1b	1b	Phyl	Compatible subgenotype
O4A	MG950366	MG950366	1b	#N/A	1b	1b	Phyl	Compatible subgenotype
B2A d168	MH311874	MH311874	1b	#N/A	1b	1b	Phyl	Compatible subgenotype
B3A d168	MH311875	MH311875	1b	#N/A	1b	1b	Phyl	Compatible subgenotype
B4A d84	MH311876	MH311876	1b	#N/A	1b	1b	Phyl	Compatible subgenotype
B4A d168	MH311877	MH311877	1b	#N/A	1b	1b	Phyl	Compatible subgenotype
B5A d84	MH311878	MH311878	1b	#N/A	1b	1b	Phyl	Compatible subgenotype
B5A d168	MH311879	MH311879	1b	#N/A	1b	1b	Phyl	Compatible subgenotype
B6A d84	MH311880	MH311880	1b	#N/A	1b	1b	Phyl	Compatible subgenotype
B6A d168	MH311881	MH311881	1b	#N/A	1b	1b	Phyl	Compatible subgenotype
P1	MH379221	MH379221	1b	#N/A	1b	1b	Phyl	Compatible subgenotype
P2	MH379222	MH379222	1b	#N/A	1b	1b	Phyl	Compatible subgenotype
P5	MH379223	MH379223	1b	#N/A	1b	1b	Phyl	Compatible subgenotype
P6	MH379224	MH379224	1b	#N/A	1b	1b	Phyl	Compatible subgenotype
P7	MH379225	MH379225	1b	#N/A	1b	1b	Phyl	Compatible subgenotype
P5A	MH379226	MH379226	1b	#N/A	1b	1b	Phyl	Compatible subgenotype
P5B	MH379227	MH379227	1b	#N/A	1b	1b	Phyl	Compatible subgenotype
P5C	MH379228	MH379228	1b	#N/A	1b	1b	Phyl	Compatible subgenotype
P5D	MH379229	MH379229	1b	#N/A	1b	1b	Phyl	Compatible subgenotype
P5F	MH379230	MH379230	1b	#N/A	1b	1b	Phyl	Compatible subgenotype
P7A	MH379231	MH379231	1b	#N/A	1b	1b	Phyl	Compatible subgenotype
P7C	MH379232	MH379232	1b	#N/A	1b	1b	Phyl	Compatible subgenotype
P7E	MH379233	MH379233	1b	#N/A	1b	1b	Phyl	Compatible subgenotype
P7F	MH379234	MH379234	1b	#N/A	1b	1b	Phyl	Compatible subgenotype
SLO/3301/2014	MH899941	MH899941	1b	#N/A	1b	1b	Phyl	Compatible subgenotype
BVD1b-JH	MK509774	MK509774	1b	#N/A	1b	1b	Phyl	Compatible subgenotype
GXSS01	MW014286	MW014286	1b	#N/A	1b	1b	Phyl	Compatible subgenotype
GXSS02	MW014287	MW014287	1b	#N/A	1b	1b	Phyl	Compatible subgenotype
GXSS03	MW014288	MW014288	1b	#N/A	1b	1b	Phyl	Compatible subgenotype
ILLNC	U86600	U86600	1b	#N/A	1b	1b	Phyl	Compatible subgenotype
GSTZ	MF172980	MF172980	1c	#N/A	1c	1c	Phyl	Compatible subgenotype
GXNN1	MT079816	MT079816	1c	#N/A	1c	1c	Phyl	Compatible subgenotype
SLO/33529/2015	MH899942	MH899942	1e	#N/A	1e	1e	Phyl	Compatible subgenotype
SLO/1170/2000	KX987157	KX987157	1f	#N/A	1f	1f	Phyl	Compatible subgenotype
SLO/1361/2014	MH899943	MH899943	1f	#N/A	1f	1f	Phyl	Compatible subgenotype
SLO/28537/2017	MH899944	MH899944	1f	#N/A	1f	1f	Phyl	Compatible subgenotype
UM/111/06	MW054936	MW054936	1g	#N/A	1g	1g	Phyl	Compatible subgenotype
SLO/1883/2013	MH899945	MH899945	1h	#N/A	1h	1h	Phyl	Compatible subgenotype
TO/197/11	MW054935	MW054935	1k	#N/A	1k	1k	Phyl	Compatible subgenotype
IS26/01ncp	LC089875	LC089875	1o	#N/A	1o	1o	Phyl	Compatible subgenotype
20170226	MK102095	MK102095	1q	#N/A	1q	1q	Phyl	Compatible subgenotype
UNKNOWN-LT837585	LT837585	LT837585	1r	#N/A	1r	1r	Phyl	Compatible subgenotype

1.III: Details on subgenotyping of strains according to phylogenetic analysis and/or GenBank

Strain	E2 GB #	Npro GB #	Subgenotype	Subgenotype from GB	Subgenotype from Npro phylogeny	Subgenotype from E2 phylogeny	Source of Subgenotype	Note
2139	MH231125	MH231125	2a	#N/A	2a	2a	Phyl	Compatible subgenotype
9231	MH806437	MH806437	2a	#N/A	2a	2a	Phyl	Compatible subgenotype
10406	MH231123	MH231123	2a	#N/A	2a	2a	Phyl	Compatible subgenotype
570152	MH231128	MH231128	2a	#N/A	2a	2a	Phyl	Compatible subgenotype
V002	KX170138	KX170299	2a	#N/A	2a	2a	Phyl	Compatible subgenotype
V005	KX170137	KX170298	2a	#N/A	2a	2a	Phyl	Compatible subgenotype
V017	KX170128	KX170288	2a	#N/A	2a	2a	Phyl	Compatible subgenotype
V021	KX170142	KX170300	2a	#N/A	2a	2a	Phyl	Compatible subgenotype
V023	KX170132	KX170290	2a	#N/A	2a	2a	Phyl	Compatible subgenotype
V024	KX170135	KX170294	2a	#N/A	2a	2a	Phyl	Compatible subgenotype
V025	KX170133	KX170295	2a	#N/A	2a	2a	Phyl	Compatible subgenotype
V028	KX170118	KX170280	2a	#N/A	2a	2a	Phyl	Compatible subgenotype
V037	KX170141	KX170297	2a	#N/A	2a	2a	Phyl	Compatible subgenotype
V044	KX170134	KX170291	2a	#N/A	2a	2a	Phyl	Compatible subgenotype
V047	KX170129	KX170284	2a	#N/A	2a	2a	Phyl	Compatible subgenotype
V051	KX170136	KX170292	2a	#N/A	2a	2a	Phyl	Compatible subgenotype
V062	KX170124	KX170281	2a	#N/A	2a	2a	Phyl	Compatible subgenotype
V063	KX170125	KX170283	2a	#N/A	2a	2a	Phyl	Compatible subgenotype
V076	KX170130	KX170285	2a	#N/A	2a	2a	Phyl	Compatible subgenotype
V079	KX170143	KX170301	2a	#N/A	2a	2a	Phyl	Compatible subgenotype
V081	KX170123	KX170287	2a	#N/A	2a	2a	Phyl	Compatible subgenotype
V082	KX170126	KX170289	2a	#N/A	2a	2a	Phyl	Compatible subgenotype
V085	KX170131	KX170286	2a	#N/A	2a	2a	Phyl	Compatible subgenotype
V086	KX170119	KX170278	2a	#N/A	2a	2a	Phyl	Compatible subgenotype
V088	KX170121	KX170277	2a	#N/A	2a	2a	Phyl	Compatible subgenotype
V089	KX170120	KX170279	2a	#N/A	2a	2a	Phyl	Compatible subgenotype
V095	KX170122	KX170276	2a	#N/A	2a	2a	Phyl	Compatible subgenotype
V097	KX170140	KX170296	2a	#N/A	2a	2a	Phyl	Compatible subgenotype
V065	KX170139	KX170293	2a	#N/A	2a	2a	Phyl	Compatible subgenotype
32W	KT875139	KT875139	2a	#N/A	2a	2a	Phyl	Compatible subgenotype
12W	KT875134	KT875134	2a	#N/A	2a	2a	Phyl	Compatible subgenotype
13Y	KT875135	KT875135	2a	#N/A	2a	2a	Phyl	Compatible subgenotype
24515	KP057803	KP057803	2a	#N/A	2a	2a	Phyl	Compatible subgenotype
27Y	KT875136	KT875136	2a	#N/A	2a	2a	Phyl	Compatible subgenotype
29Y	KT875137	KT875137	2a	#N/A	2a	2a	Phyl	Compatible subgenotype
2Y	KT875138	KT875138	2a	#N/A	2a	2a	Phyl	Compatible subgenotype
34Y	KT875140	KT875140	2a	#N/A	2a	2a	Phyl	Compatible subgenotype
36W	KT875141	KT875141	2a	#N/A	2a	2a	Phyl	Compatible subgenotype
41Y	KT875142	KT875142	2a	#N/A	2a	2a	Phyl	Compatible subgenotype
42W	KT875143	KT875143	2a	#N/A	2a	2a	Phyl	Compatible subgenotype
43Y	KT875144	KT875144	2a	#N/A	2a	2a	Phyl	Compatible subgenotype
47Y	KT875145	KT875145	2a	#N/A	2a	2a	Phyl	Compatible subgenotype
50Y	KT875146	KT875146	2a	#N/A	2a	2a	Phyl	Compatible subgenotype
51W	KT875147	KT875147	2a	#N/A	2a	2a	Phyl	Compatible subgenotype
51Y	KT875148	KT875148	2a	#N/A	2a	2a	Phyl	Compatible subgenotype
53W	KT875149	KT875149	2a	#N/A	2a	2a	Phyl	Compatible subgenotype
58W	KT875150	KT875150	2a	#N/A	2a	2a	Phyl	Compatible subgenotype
58Y	KT875151	KT875151	2a	#N/A	2a	2a	Phyl	Compatible subgenotype
5Y	KT875152	KT875152	2a	#N/A	2a	2a	Phyl	Compatible subgenotype
62Y	KT875153	KT875153	2a	#N/A	2a	2a	Phyl	Compatible subgenotype
65Y	KT875154	KT875154	2a	#N/A	2a	2a	Phyl	Compatible subgenotype
67Y	KT875155	KT875155	2a	#N/A	2a	2a	Phyl	Compatible subgenotype
68W	KT875156	KT875156	2a	#N/A	2a	2a	Phyl	Compatible subgenotype
71Y	KT875157	KT875157	2a	#N/A	2a	2a	Phyl	Compatible subgenotype
73Y	KT875158	KT875158	2a	#N/A	2a	2a	Phyl	Compatible subgenotype
74Y	KT875159	KT875159	2a	#N/A	2a	2a	Phyl	Compatible subgenotype
75W	KT875160	KT875160	2a	#N/A	2a	2a	Phyl	Compatible subgenotype
75Y	KT875161	KT875161	2a	#N/A	2a	2a	Phyl	Compatible subgenotype

1.III: Details on subgenotyping of strains according to phylogenetic analysis and/or GenBank

Strain	E2 GB #	Npro GB #	Subgenotype	Subgenotype from GB	Subgenotype from Npro phylogeny	Subgenotype from E2 phylogeny	Source of Subgenotype	Note
76Y	KT875162	KT875162	2a	#N/A	2a	2a	Phyl	Compatible subgenotype
78W	KT875163	KT875163	2a	#N/A	2a	2a	Phyl	Compatible subgenotype
79W	KT875164	KT875164	2a	#N/A	2a	2a	Phyl	Compatible subgenotype
7W	KT875165	KT875165	2a	#N/A	2a	2a	Phyl	Compatible subgenotype
82W	KT875166	KT875166	2a	#N/A	2a	2a	Phyl	Compatible subgenotype
83Y	KT875167	KT875167	2a	#N/A	2a	2a	Phyl	Compatible subgenotype
90W	KT875168	KT875168	2a	#N/A	2a	2a	Phyl	Compatible subgenotype
91W	KT875169	KT875169	2a	#N/A	2a	2a	Phyl	Compatible subgenotype
USMARC-55476	KP941585	KP941585	2a	#N/A	2a	2a	Phyl	Compatible subgenotype
USMARC-60767	KT832820	KT832820	2a	#N/A	2a	2a	Phyl	Compatible subgenotype
USMARC-60764	KT832817	KT832817	2a	#N/A	2a	2a	Phyl	Compatible subgenotype
USMARC-60779	KT832822	KT832822	2a	#N/A	2a	2a	Phyl	Compatible subgenotype
USMARC-60766	KT832819	KT832819	2a	#N/A	2a	2a	Phyl	Compatible subgenotype
USMARC-60780	KT832823	KT832823	2a	#N/A	2a	2a	Phyl	Compatible subgenotype
XJ-04	FJ527854	FJ527854	2a	#N/A	2a	2a	Phyl	Compatible subgenotype
CN10.2015.821	MG879027	MG879027	2a	#N/A	2a	2a	Phyl	Compatible subgenotype
Ind141353	HQ444199	HQ444199	2a	#N/A	2a	2a	Phyl	Compatible subgenotype
YNJG2020	MW168422	MW168422	2a	#N/A	2a	2a	Phyl	Compatible subgenotype
GS2018	MN527354	MN527354	2a	#N/A	2a	2a	Phyl	Compatible subgenotype
3237	MH231126	MH231126	2b	#N/A	2b	2b	Phyl	Compatible subgenotype
17237	EU747875	EU747875	2b	#N/A	2b	2b	Phyl	Compatible subgenotype
HEN01	MW006485	MW006485	2b	#N/A	2b	2b	Phyl	Compatible subgenotype
NRW 19-13-1_Dup(+)	HG426488	HG426488	2c	#N/A	2c	2c	Phyl	Compatible subgenotype
NRW 19-13-1_Dup(-)	HG426487	HG426487	2c	#N/A	2c	2c	Phyl	Compatible subgenotype
D75-13-609_Dup(+)	HG426482	HG426482	2c	#N/A	2c	2c	Phyl	Compatible subgenotype
NRW 14-13_Dup(+)	HG426486	HG426486	2c	#N/A	2c	2c	Phyl	Compatible subgenotype
Potsdam 1600	HG426491	HG426491	2c	#N/A	2c	2c	Phyl	Compatible subgenotype
USMARC-53873	KP941582	KP941582	2c	#N/A	2c	2c	Phyl	Compatible subgenotype
USMARC-60768	KT832821	KT832821	2c	#N/A	2c	2c	Phyl	Compatible subgenotype
D75-13-609_Dup(-)	HG426481	HG426481	2c	#N/A	2c	2c	Phyl	Compatible subgenotype
NRW 14-13_Dup(-)	HG426485	HG426485	2c	#N/A	2c	2c	Phyl	Compatible subgenotype
NRW 19-13-8_Dup(-)	HG426489	HG426489	2c	#N/A	2c	2c	Phyl	Compatible subgenotype
NRW 19-13-8_Dup(+)	HG426490	HG426490	2c	#N/A	2c	2c	Phyl	Compatible subgenotype
KZ-91-NCP	LC649064	LC649064	2c	#N/A	2c	2c	Phyl	Compatible subgenotype
14622	MH231151	MH231151	2e	#N/A	2e	2e	Phyl	Compatible subgenotype
**UNKNOWN-M96751**	**M96751**	**M96751**	**1a**	**#N/A**	**1a**	**1a**	**Ref Strain**	Compatible subgenotype
2412	**MH231152**	**MH231152**	**2e**	#N/A	2e	2e	**Phyl**	Compatible subgenotype
**SD1301**	**KJ000672**	**KF925365**	**2b**	#N/A	2b	2b	**Ref Strain**	Compatible subgenotype
**SH2210-17**	**HG426493**	**HG426493**	**2c**	#N/A	2c	2c	**Ref Strain**	Compatible subgenotype
**VOE 4407**	**HG426495**	**HG426495**	**2c**	#N/A	2c	2c	**Ref Strain**	Compatible subgenotype
**CP7**	**U63479**	**U63479**	**1b**	**#N/A**	**1b**	**1b**	**Ref Strain**	Compatible subgenotype
**type 1**	**AJ133738**	**AJ133738**	**1a**	**#N/A**	**1a**	**1a**	**Ref Strain**	Compatible subgenotype
**Singer_Arg**	**DQ088995**	**DQ088995**	**1a**	**#N/A**	**1a**	**1a**	**Ref Strain**	Compatible subgenotype
**KE9**	**EF101530**	**EF101530**	**1b**	**#N/A**	**1b**	**1b**	**Ref Strain**	Compatible subgenotype
**Osloss**	**M96687**	**M96687**	**1b**	**#N/A**	**1b**	**1b**	**Ref Strain**	Compatible subgenotype
**KS86-1ncp**	**AB078950**	**AB078950**	**1j**	**#N/A**	**1j**	**1j**	**Ref Strain**	Compatible subgenotype
**ZM-95**	**AF526381**	**AF526381**	**1m**	**#N/A**	**1m**	**1m**	**Ref Strain**	Compatible subgenotype
**SD0803**	**JN400273**	**JN400273**	**1q**	**#N/A**	**1q**	**1q**	**Ref Strain**	Compatible subgenotype
**M31182**	**JQ799141**	**JQ799141**	**1u**	**#N/A**	**1u**	**1u**	**Ref Strain**	Compatible subgenotype
**JZ05-1**	**GQ888686**	**GQ888686**	**2a**	#N/A	2a	2a	**Ref Strain**	Compatible subgenotype
**D37-13-2_Dup(-)**	**HG426479**	**HG426479**	**2c**	#N/A	2c	2c	**Ref Strain**	Compatible subgenotype
**D37-13-2_Dup(+)**	**HG426480**	**HG426480**	**2c**	#N/A	2c	2c	**Ref Strain**	Compatible subgenotype
**NRW 12-13_Dup(-)**	**HG426483**	**HG426483**	**2c**	#N/A	2c	2c	**Ref Strain**	Compatible subgenotype
**NRW 12-13_Dup(+)**	**HG426484**	**HG426484**	**2c**	#N/A	2c	2c	**Ref Strain**	Compatible subgenotype
**SH2210-14**	**HG426492**	**HG426492**	**2c**	#N/A	2c	2c	**Ref Strain**	Compatible subgenotype
**SH2210-23**	**HG426494**	**HG426494**	**2c**	#N/A	2c	2c	**Ref Strain**	Compatible subgenotype

1.III: Details on subgenotyping of strains according to phylogenetic analysis and/or GenBank

Strain	E2 GB #	Npro GB #	Subgenotype	Subgenotype from GB	Subgenotype from Npro phylogeny	Subgenotype from E2 phylogeny	Source of Subgenotype	Note
USMARC-60765	KT832818	KT832818	2a	#N/A	2a	?	Phyl	E2 show divergence (??) and discarded, seq was most simialr to 2a (similar finding was obtained by (de Oliveira et al., 2022))
SH-28	HQ258810	HQ258810	2a	#N/A	2a	??	Phyl	E2 show divergence (??) and discarded, seq was most similar to 2a (similar finding was obtained by (de Oliveira et al., 2022))
SD-1	MK599227	MK599227	2a	#N/A	2a	2c	Phyl	E2 show divergence (2c) and discarded, seq was most similar to 2a (similar finding was obtained by (de Oliveira et al., 2022))
HLJ-10	JF714967	JF714967	2a	#N/A	2a	2c	Phyl	E2 show divergence (2c) and discarded, seq was most similar to 2a (similar finding was obtained by (de Oliveira et al., 2022)).
GS8	#N/A	#N/A	1a	1a	#N/A	#N/A	GB	GB# of the source: KY675205
CCSYD	#N/A	#N/A	1b	1b	#N/A	#N/A	GB	GB# of the source: FJ555203
GS6	#N/A	#N/A	1b	1b	#N/A	#N/A	GB	GB# of the source: KY675202
HB-DCZ	#N/A	#N/A	1b	1b	#N/A	#N/A	GB	GB# of the source: JX046799
QH15	#N/A	#N/A	1b	1b	#N/A	#N/A	GB	GB# of the source: KY675222
QH16	#N/A	#N/A	1b	1b	#N/A	#N/A	GB	GB# of the source: KY675223
QH17	#N/A	#N/A	1b	1b	#N/A	#N/A	GB	GB# of the source: KY675224
QH4	#N/A	#N/A	1b	1b	#N/A	#N/A	GB	GB# of the source: KY675216
QHZK10	#N/A	#N/A	1b	1b	#N/A	#N/A	GB	GB# of the source: JF927789
GS13	#N/A	#N/A	1c	1c	#N/A	#N/A	GB	GB# of the source: KY675208
NX1	#N/A	#N/A	1d	1d	#N/A	#N/A	GB	GB# of the source: MW560180
QH1	#N/A	#N/A	1d	1d	#N/A	#N/A	GB	GB# of the source: KY675214
QH9	#N/A	#N/A	1d	1d	#N/A	#N/A	GB	GB# of the source: KY675219
GS10	#N/A	#N/A	1m	1m	#N/A	#N/A	GB	GB# of the source: KY675203
GS17	#N/A	#N/A	1m	1m	#N/A	#N/A	GB	GB# of the source: KY675209
GS20	#N/A	#N/A	1m	1m	#N/A	#N/A	GB	GB# of the source: KY675210
GS25	#N/A	#N/A	1m	1m	#N/A	#N/A	GB	GB# of the source: KY675212
GS7	#N/A	#N/A	1m	1m	#N/A	#N/A	GB	GB# of the source: KY675204
GS9	#N/A	#N/A	1m	1m	#N/A	#N/A	GB	GB# of the source: KY675206
NX5	#N/A	#N/A	1m	1m	#N/A	#N/A	GB	GB# of the source: MW560184
NX6	#N/A	#N/A	1m	1m	#N/A	#N/A	GB	GB# of the source: MW560185
QH12	#N/A	#N/A	1m	1m	#N/A	#N/A	GB	GB# of the source: KY675221
QH20	#N/A	#N/A	1m	1m	#N/A	#N/A	GB	GB# of the source: KY675226
QH23	#N/A	#N/A	1m	1m	#N/A	#N/A	GB	GB# of the source: KY675227
GS23	#N/A	#N/A	1o	1o	#N/A	#N/A	GB	GB# of the source: KY675211
Camel-5	#N/A	#N/A	1p	1p	#N/A	#N/A	GB	GB# of the source: KY675228
GS11	#N/A	#N/A	1p	1p	#N/A	#N/A	GB	GB# of the source: KY675207
QH11	#N/A	#N/A	1p	1p	#N/A	#N/A	GB	GB# of the source: KY675220
QH18	#N/A	#N/A	1p	1p	#N/A	#N/A	GB	GB# of the source: KY675225
QH2	#N/A	#N/A	1p	1p	#N/A	#N/A	GB	GB# of the source: KY675215
GS26	#N/A	#N/A	1q	1q	#N/A	#N/A	GB	GB# of the source: KY675213
NX2	#N/A	#N/A	1q	1q	#N/A	#N/A	GB	GB# of the source: MW560181
QH5	#N/A	#N/A	1q	1q	#N/A	#N/A	GB	GB# of the source: KY675217
QH8	#N/A	#N/A	1q	1q	#N/A	#N/A	GB	GB# of the source: KY675218
NX201902	#N/A	#N/A	1v	1v	#N/A	#N/A	GB	GB# of the source: MW560183
560615-F0-22	#N/A	#N/A	2a	2a	#N/A	#N/A	GB	GB# of the source: MT024568
IndMDV18697/12	#N/A	KM261881	1b	#N/A	1b	#N/A	Phyl	
JR1-2	#N/A	KX218372	1b	#N/A	1b	#N/A	Phyl	
FarsB	#N/A	KY488632	1b	#N/A	1b	#N/A	Phyl	
NSF116	#N/A	MK982965	1b	#N/A	1b	#N/A	Phyl	
KSF1322	#N/A	MK982966	1b	#N/A	1b	#N/A	Phyl	
KBF955	#N/A	MK982967	1b	#N/A	1b	#N/A	Phyl	
HN1437	#N/A	MN442389	1b	#N/A	1b	#N/A	Phyl	
HN1506	#N/A	MN442390	1b	#N/A	1b	#N/A	Phyl	
HN1522	#N/A	MN442391	1b	#N/A	1b	#N/A	Phyl	
HN1727	#N/A	MN442400	1b	#N/A	1b	#N/A	Phyl	

1.III: Details on subgenotyping of strains according to phylogenetic analysis and/or GenBank

Strain	E2 GB #	Npro GB #	Subgenotype	Subgenotype from GB	Subgenotype from Npro phylogeny	Subgenotype from E2 phylogeny	Source of Subgenotype	Note
HN1753	#N/A	MN442404	1b	#N/A	1b	#N/A	Phyl	
HN1877	#N/A	MN442411	1b	#N/A	1b	#N/A	Phyl	
433/16	#N/A	MW605050	1b	#N/A	1b	#N/A	Phyl	
438/16	#N/A	MW605051	1b	#N/A	1b	#N/A	Phyl	
439/16	#N/A	MW605052	1b	#N/A	1b	#N/A	Phyl	
441/16	#N/A	MW605053	1b	#N/A	1b	#N/A	Phyl	
504/16	#N/A	MW605054	1b	#N/A	1b	#N/A	Phyl	
5/16	#N/A	MW605055	1b	#N/A	1b	#N/A	Phyl	
TR-K2019-02	#N/A	MZ209055	1b	#N/A	1b	#N/A	Phyl	
71982/2011/PA	#N/A	KU856558	1d	#N/A	1d	#N/A	Phyl	
71982/2011/2PA	#N/A	KU856559	1d	#N/A	1d	#N/A	Phyl	
Bov/Ita/124.15-14	#N/A	KX890141	1h	#N/A	1h	#N/A	Phyl	
NBF1383	#N/A	MK982970	1j	#N/A	1j	#N/A	Phyl	
TR-U2018-01	#N/A	MZ209056	1j	#N/A	1j	#N/A	Phyl	
FarsA	#N/A	KY488631	1l	#N/A	1l	#N/A	Phyl	
ABF710	#N/A	MK982969	1l	#N/A	1l	#N/A	Phyl	
TR-A2019-01	#N/A	MZ209052	1l	#N/A	1l	#N/A	Phyl	
TY05	#N/A	GU120258	1m	#N/A	1m	#N/A	Phyl	
HN1539	#N/A	MN442392	1m	#N/A	1m	#N/A	Phyl	
HN1613	#N/A	MN442393	1m	#N/A	1m	#N/A	Phyl	
HN1711	#N/A	MN442397	1m	#N/A	1m	#N/A	Phyl	
HN1720	#N/A	MN442398	1m	#N/A	1m	#N/A	Phyl	
HN1725	#N/A	MN442399	1m	#N/A	1m	#N/A	Phyl	
HN1821	#N/A	MN442407	1m	#N/A	1m	#N/A	Phyl	
JS12/02	#N/A	KX218371	1o	#N/A	1o	#N/A	Phyl	
HN1626	#N/A	MN442395	1o	#N/A	1o	#N/A	Phyl	
HN1641	#N/A	MN442396	1o	#N/A	1o	#N/A	Phyl	
HN1732	#N/A	MN442402	1o	#N/A	1o	#N/A	Phyl	
HN1736	#N/A	MN442403	1o	#N/A	1o	#N/A	Phyl	
HN1814	#N/A	MN442406	1o	#N/A	1o	#N/A	Phyl	
HN1852	#N/A	MN442408	1o	#N/A	1o	#N/A	Phyl	
HN1859	#N/A	MN442409	1o	#N/A	1o	#N/A	Phyl	
HN1864	#N/A	MN442410	1o	#N/A	1o	#N/A	Phyl	
HN1918	#N/A	MN442412	1o	#N/A	1o	#N/A	Phyl	
Zhiduo17	#N/A	KC414582	1q	#N/A	1q	#N/A	Phyl	
Zhiduo11	#N/A	KC414583	1q	#N/A	1q	#N/A	Phyl	
Yushu2219	#N/A	KC414588	1q	#N/A	1q	#N/A	Phyl	
Xinghai6007	#N/A	KC414591	1q	#N/A	1q	#N/A	Phyl	
Xinghai6003	#N/A	KC414592	1q	#N/A	1q	#N/A	Phyl	
Zhiduo28	#N/A	KC414593	1q	#N/A	1q	#N/A	Phyl	
Yushu2202	#N/A	KC414597	1q	#N/A	1q	#N/A	Phyl	
HN1618	#N/A	MN442394	1q	#N/A	1q	#N/A	Phyl	
HN1729	#N/A	MN442401	1q	#N/A	1q	#N/A	Phyl	
HN1802	#N/A	MN442405	1u	#N/A	1u	#N/A	Phyl	
FarsB	#N/A	KY488630	2a	#N/A	2a	#N/A	Phyl	
V019	#N/A	KX170282	2a	#N/A	2a	#N/A	Phyl	
53099	#N/A	FJ431189	2a	#N/A	2a	#N/A	Phyl	
53100	#N/A	FJ431190	2a	#N/A	2a	#N/A	Phyl	
65	#N/A	FJ431192	2a	#N/A	2a	#N/A	Phyl	
68	#N/A	FJ431193	2a	#N/A	2a	#N/A	Phyl	
73	#N/A	FJ431194	2a	#N/A	2a	#N/A	Phyl	
Ind_3012339	#N/A	MF157331	2a	#N/A	2a	#N/A	Phyl	
LV/Hipra01/12	#N/A	KP743042	2b	#N/A	2b	#N/A	Phyl	
**BJ0703**	**#N/A**	**GU120261**	**1p**	**#N/A**	**1p**	**#N/A**	**Ref Strain**	
**TJ0801**	**#N/A**	**GU120262**	**1m**	**#N/A**	**1m**	**#N/A**	**Ref Strain**	
**HA2-12**	**#N/A**	**KX218370**	**1o**	**#N/A**	**1o**	**#N/A**	**Ref Strain**	
**BJ0701**	**#N/A**	**GU120259**	**1p**	**#N/A**	**1p**	**#N/A**	**Ref Strain**	
**BJ0702**	**#N/A**	**GU120260**	**1p**	**#N/A**	**1p**	**#N/A**	**Ref Strain**	

1.III: Details on subgenotyping of strains according to phylogenetic analysis and/or GenBank

Strain	E2 GB #	Npro GB #	Subgenotype	Subgenotype from GB	Subgenotype from Npro phylogeny	Subgenotype from E2 phylogeny	Source of Subgenotype	Note
BVDV/Ibaraki/32/15	LC630446	#N/A	1b	#N/A	#N/A	1b	Phyl	Compatible subgenotype with (Nishimori et al., 2022)
BVDV/Ibaraki/36/15	LC630447	#N/A	1b	#N/A	#N/A	1b	Phyl	Compatible subgenotype with (Nishimori et al., 2022)
BVDV/Gunma/01/16	LC630448	#N/A	1b	#N/A	#N/A	1b	Phyl	Compatible subgenotype with (Nishimori et al., 2022)
BVDV/Gunma/03/16	LC630449	#N/A	1b	#N/A	#N/A	1b	Phyl	Compatible subgenotype with (Nishimori et al., 2022)
BVDV/Gunma/05/16	LC630450	#N/A	1b	#N/A	#N/A	1b	Phyl	Compatible subgenotype with (Nishimori et al., 2022)
BVDV/Gunma/01/17	LC630451	#N/A	1b	#N/A	#N/A	1b	Phyl	Compatible subgenotype with (Nishimori et al., 2022)
BVDV/Gunma/02/17	LC630452	#N/A	1b	#N/A	#N/A	1b	Phyl	Compatible subgenotype with (Nishimori et al., 2022)
BVDV/Gunma/03/17	LC630453	#N/A	1b	#N/A	#N/A	1b	Phyl	Compatible subgenotype with (Nishimori et al., 2022)
BVDV/Gunma/06/17	LC630454	#N/A	1b	#N/A	#N/A	1b	Phyl	Compatible subgenotype with (Nishimori et al., 2022)
BVDV/Gunma/07/17	LC630455	#N/A	1b	#N/A	#N/A	1b	Phyl	Compatible subgenotype with (Nishimori et al., 2022)
BVDV/Gunma/11/17	LC630456	#N/A	1b	#N/A	#N/A	1b	Phyl	Compatible subgenotype with (Nishimori et al., 2022)
BVDV/Gunma/01/18	LC630457	#N/A	1b	#N/A	#N/A	1b	Phyl	Compatible subgenotype with (Nishimori et al., 2022)
BVDV/Gunma/04/18	LC630458	#N/A	1b	#N/A	#N/A	1b	Phyl	Compatible subgenotype with (Nishimori et al., 2022)
BVDV/Gunma/12/18	LC630459	#N/A	1b	#N/A	#N/A	1b	Phyl	Compatible subgenotype with (Nishimori et al., 2022)
BVDV/Gunma/13/18	LC630460	#N/A	1b	#N/A	#N/A	1b	Phyl	Compatible subgenotype with (Nishimori et al., 2022)
BVDV/Gunma/14/18	LC630461	#N/A	1b	#N/A	#N/A	1b	Phyl	Compatible subgenotype with (Nishimori et al., 2022)
BVDV/Gunma/19/18	LC630462	#N/A	1b	#N/A	#N/A	1b	Phyl	Compatible subgenotype with (Nishimori et al., 2022)
BVDV/Gunma/22/18	LC630463	#N/A	1b	#N/A	#N/A	1b	Phyl	Compatible subgenotype with (Nishimori et al., 2022)
BVDV/Gunma/23/18	LC630464	#N/A	1b	#N/A	#N/A	1b	Phyl	Compatible subgenotype with (Nishimori et al., 2022)
BVDV/Gunma/24/18	LC630465	#N/A	1b	#N/A	#N/A	1b	Phyl	Compatible subgenotype with (Nishimori et al., 2022)
BVDV/Gunma/05/19	LC630466	#N/A	1b	#N/A	#N/A	1b	Phyl	Compatible subgenotype with (Nishimori et al., 2022)
BVDV/Gunma/07/19	LC630467	#N/A	1b	#N/A	#N/A	1b	Phyl	Compatible subgenotype with (Nishimori et al., 2022)
BVDV/Gunma/11/19	LC630468	#N/A	1b	#N/A	#N/A	1b	Phyl	Compatible subgenotype with (Nishimori et al., 2022)
BVDV/Gunma/15/19	LC630469	#N/A	1b	#N/A	#N/A	1b	Phyl	Compatible subgenotype with (Nishimori et al., 2022)
BVDV/Okayama/11/18	LC630470	#N/A	1b	#N/A	#N/A	1b	Phyl	Compatible subgenotype with (Nishimori et al., 2022)
BVDV/Okayama/21/19	LC630471	#N/A	1b	#N/A	#N/A	1b	Phyl	Compatible subgenotype with (Nishimori et al., 2022)
BVDV/Okayama/24/20	LC630472	#N/A	1b	#N/A	#N/A	1b	Phyl	Compatible subgenotype with (Nishimori et al., 2022)
BVDV/Kumamoto/01/20	LC630473	#N/A	1b	#N/A	#N/A	1b	Phyl	Compatible subgenotype with (Nishimori et al., 2022)
BVDV/Oita/01/18	LC630474	#N/A	1b	#N/A	#N/A	1b	Phyl	Compatible subgenotype with (Nishimori et al., 2022)
BVDV/Oita/07/18	LC630475	#N/A	1b	#N/A	#N/A	1b	Phyl	Compatible subgenotype with (Nishimori et al., 2022)
BVDV/Oita/10/18	LC630476	#N/A	1b	#N/A	#N/A	1b	Phyl	Compatible subgenotype with (Nishimori et al., 2022)
BVDV/Kamiyuubetsu/08/0	AB896799	#N/A	1a	#N/A	#N/A	1a	Phyl	
BVDV/Monbetsu/03/01-C	AB896800	#N/A	1a	#N/A	#N/A	1a	Phyl	
BVDV/Monbetsu/205/04	AB896801	#N/A	1a	#N/A	#N/A	1a	Phyl	
BVDV/Oketo/277/04	AB896802	#N/A	1a	#N/A	#N/A	1a	Phyl	
BVDV/Betsukai/503/07	AB896803	#N/A	1a	#N/A	#N/A	1a	Phyl	
BVDV/Betsukai/669/08	AB896804	#N/A	1a	#N/A	#N/A	1a	Phyl	
BVDV/Akkeshi/710/09	LC016729	#N/A	1a	#N/A	#N/A	1a	Phyl	
BVDV/Hamatonbetsu/119	LC016730	#N/A	1a	#N/A	#N/A	1a	Phyl	
BSC-2	MK170074	#N/A	1a	#N/A	#N/A	1a	Phyl	
BSC-3	MK170075	#N/A	1a	#N/A	#N/A	1a	Phyl	
BSC-4	MK170076	#N/A	1a	#N/A	#N/A	1a	Phyl	
BSC-5	MK170077	#N/A	1a	#N/A	#N/A	1a	Phyl	
BSC-6	MK170078	#N/A	1a	#N/A	#N/A	1a	Phyl	
Hubei	MZ484396	#N/A	1a	#N/A	#N/A	1a	Phyl	
BVDV/Nakashibetsu/881/	AB894349	#N/A	1b	#N/A	#N/A	1b	Phyl	
BVDV/Yuubetsu/10/01	AB896805	#N/A	1b	#N/A	#N/A	1b	Phyl	
BVDV/Saroma/23/02	AB896806	#N/A	1b	#N/A	#N/A	1b	Phyl	
BVDV/Okoppe/89/01	AB896807	#N/A	1b	#N/A	#N/A	1b	Phyl	
BVDV/Nakasatsunai/583/(	AB896808	#N/A	1b	#N/A	#N/A	1b	Phyl	
BVDV/Nakasatsunai/719/(	AB896809	#N/A	1b	#N/A	#N/A	1b	Phyl	
BVDV/Nakashibetsu/856/	AB896810	#N/A	1b	#N/A	#N/A	1b	Phyl	
BVDV/Betsukai/884/10	AB896811	#N/A	1b	#N/A	#N/A	1b	Phyl	
XJ	KF856290	#N/A	1b	#N/A	#N/A	1b	Phyl	
BVDV/Hamanaka/646/08	LC016731	#N/A	1b	#N/A	#N/A	1b	Phyl	
BVDV/Setana/1103/12	LC016732	#N/A	1b	#N/A	#N/A	1b	Phyl	
BSC-1	MK170073	#N/A	1c	#N/A	#N/A	1c	Phyl	
BVDV/Hamanaka/843/10	LC016727	#N/A	2a	#N/A	#N/A	2a	Phyl	

1.III: Details on subgenotyping of strains according to phylogenetic analysis and/or GenBank

Strain	E2 GB #	Npro GB #	Subgenotype	Subgenotype from GB	Subgenotype from Npro phylogeny	Subgenotype from E2 phylogeny	Source of Subgenotype	Note
BVDV/Shikaoi/909/10	LC016728	#N/A	2a	#N/A	#N/A	2a	Phyl	
A063379B	KJ146971	#N/A	2a	#N/A	#N/A	2a	Phyl	
M10 3432	JN377416	#N/A	2a	#N/A	#N/A	2a	Phyl	
M10 5347	JN377415	#N/A	2a	#N/A	#N/A	2a	Phyl	
BVDV/Akkeshi/1170/13	LC016726	#N/A	2c	#N/A	#N/A	2c	Phyl	
BVDV/Akkeshi/1897/20	LC648409	#N/A	2c	#N/A	#N/A	2c	Phyl	
BVDV/Engaru/1308/15	LC648403	#N/A	2c	#N/A	#N/A	2c	Phyl	
BVDV/Honbetsu/597/07	LC016725	#N/A	2c	#N/A	#N/A	2c	Phyl	
BVDV/Monbetsu/1333/16	LC648405	#N/A	2c	#N/A	#N/A	2c	Phyl	
BVDV/Nakashibetsu/1682	LC648408	#N/A	2c	#N/A	#N/A	2c	Phyl	
BVDV/Nayoro/1623/18	LC648406	#N/A	2c	#N/A	#N/A	2c	Phyl	
BVDV/Okoppe/458/05	LC016724	#N/A	2c	#N/A	#N/A	2c	Phyl	
BVDV/Oumu/1311/15	LC648404	#N/A	2c	#N/A	#N/A	2c	Phyl	
BVDV/Oumu/1654/17	LC648407	#N/A	2c	#N/A	#N/A	2c	Phyl	
BVDV/Shihoro/1258/14	LC648402	#N/A	2c	#N/A	#N/A	2c	Phyl	
BVDV/Yuubetsu/71/01	LC016723	#N/A	2c	#N/A	#N/A	2c	Phyl	
BVDV/Gunma/01/19	LC630480	#N/A	2c	#N/A	#N/A	2c	Phyl	
BVDV/Gunma/06/18	LC630477	#N/A	2c	#N/A	#N/A	2c	Phyl	
BVDV/Gunma/08/19	LC630481	#N/A	2c	#N/A	#N/A	2c	Phyl	
BVDV/Gunma/20/18	LC630478	#N/A	2c	#N/A	#N/A	2c	Phyl	
BVDV/Gunma/30/18	LC630479	#N/A	2c	#N/A	#N/A	2c	Phyl	
BVDV/Kumamoto/05/20	LC630484	#N/A	2c	#N/A	#N/A	2c	Phyl	
BVDV/Kumamoto/09/20	LC630485	#N/A	2c	#N/A	#N/A	2c	Phyl	
BVDV/Oita/17/18	LC630486	#N/A	2c	#N/A	#N/A	2c	Phyl	
BVDV/Okayama/16/19	LC630482	#N/A	2c	#N/A	#N/A	2c	Phyl	
BVDV/Okayama/25/20	LC630483	#N/A	2c	#N/A	#N/A	2c	Phyl	

References

CHANG, L., QI, Y., LIU, D., DU, Q., ZHAO, X. & TONG, D. 2021. Molecular detection and genotyping of bovine viral diarrhea virus in Western China. BMC veterinary research, 17, 1-7.

DE OLIVEIRA, P. S. B., SILVA JÚNIOR, J. V. J., WEIBLEN, R. & FLORES, E. F. 2022. A new (old) bovine viral diarrhea virus 2 subtype: BVDV-2e. Archives of Virology, 1-9.

DENG, M., CHEN, N., GUIDARINI, C., XU, Z., ZHANG, J., CAI, L., YUAN, S., SUN, Y. & METCALFE, L. 2020. Prevalence and genetic diversity of bovine viral diarrhea virus in dairy herds of China. Veterinary microbiology, 242, 108565.

FALKENBERG, S. M., DASSANAYAKE, R. P., TERHAAR, B., RIDPATH, J. F., NEILL, J. D. & ROTH, J. A. 2021. Evaluation of Antigenic Comparisons Among BVDV Isolates as it Relates to Humoral and Cell Mediated Responses. Frontiers in Veterinary Science, 8, 642.

HAN, D.-G., RYU, J.-H., PARK, J. & CHOI, K.-S. 2018. Identification of a new bovine viral diarrhea virus subtype in the Republic of Korea. BMC veterinary research, 14, 1-7.

MIROSŁAW, P. & POLAK, M. 2019. Increased genetic variation of bovine viral diarrhea virus in dairy cattle in Poland. BMC veterinary research, 15, 1-12.

NISHIMORI, A., HIROSE, S., OGINO, S., ANDOH, K., ISODA, N. & SAKODA, Y. 2022. Endemic infections of bovine viral diarrhea virus genotypes 1b and 2a isolated from cattle in Japan between 2014 and 2020. Journal of Veterinary Medical Science, 84, 228-232.

WORKMAN, A. M., HEATON, M. P., HARHAY, G. P., SMITH, T. P., GROTELUESCHEN, D. M., SJEKLOCHA, D., BRODERSEN, B., PETERSEN, J. L. & CHITKO-MCKOWN, C. G. 2016. Resolving Bovine viral diarrhea virus subtypes from persistently infected US beef calves with complete genome sequence. Journal of Veterinary Diagnostic Investigation, 28, 519-528.

BAZZUCCHI, M., BERTOLOTTI, L., CEGLIE, L., GIAMMARIOLI, M., ROSSI, E., ROSATI, S. & DE MIA, G. M. 2017. Complete nucleotide sequence of a novel bovine viral diarrhea virus subtype 1 isolate from Italy. Archives of virology, 162, 3545-3548.

LANG, Y., GAO, S., DU, J., SHAO, J., CONG, G., LIN, T., ZHAO, F., LIU, L. & CHANG, H. 2014. Polymorphic genetic characterization of E2 gene of bovine viral diarrhea virus in China. Veterinary Microbiology, 174, 554-559.

MOSENA, A. C. S., FALKENBERG, S. M., MA, H., CASAS, E., DASSANAYAKE, R. P., BOOTH, R., DE MIA, G. M., SCHWEIZER, M., CANAL, C. W. & NEILL, J. D. 2022. Use of multivariate analysis to evaluate antigenic relationships between US BVDV vaccine strains and non-US genetically divergent isolates. Journal of virological methods, 299, 114328.

YEŞILBAĞ, K., ALPAY, G. & BECHER, P. 2017. Variability and global distribution of subgenotypes of bovine viral diarrhea virus. Viruses, 9, 128.
