# Supplementary material for: Immunoinformatic prediction of the pathogenicity of bovine viral diarrhea virus genotypes: implications for viral virulence determinants, designing novel diagnostic assays and vaccines development
Source: Front Vet Sci. 2023 Jul 6;10:1130147. doi: 10.3389/fvets.2023.1130147 (PMC10359904; doi:10.3389/fvets.2023.1130147)
Supplement: Supplementary file 3 [file Table_2.pdf]

Supp. table 2: Scores of the predicted parameters for individual protein sequence

Species according to VIPRBRC	GenBank Accession	GenBank Protein Accession	Subgenotype	Protein	Strain Name	Collection Year	SVM Patho. Score	Vaxijen Antig. Score	Averged score of EMBOSS motifs
Pestivirus A	GU120258	ACZ06076.1	1m	Npro	TY05	2005	-0.7822	0.3325	1.1304
Pestivirus A	GU120259	ACZ06077.1	1p	Npro	BJ0701	2007	-0.6434	0.2778	1.1346
Pestivirus A	GU120260	ACZ06078.1	1p	Npro	BJ0702	2007	-0.5139	0.3147	1.1350
Pestivirus A	GU120261	ACZ06079.1	1p	Npro	BJ0703	2007	-0.7388	0.2736	1.1346
Pestivirus A	GU120262	ACZ06080.1	1m	Npro	TJ0801	2008	-0.4850	0.3690	1.1241
Pestivirus A	KX218370	AOO32402.1	1o	Npro	HA2-12	2012	-0.0265	0.3502	1.1287
Pestivirus A	KX218371	AOO32403.1	1o	Npro	JS12/02	2011	-0.0265	0.3502	1.1287
Pestivirus A	KX218372	AOO32404.1	1b	Npro	JR1-2	2013	-0.3004	0.3222	1.1216
Pestivirus A	MW605050	QUE40296.1	1b	Npro	433/16	2016	-0.5012	0.3192	1.1276
Pestivirus A	MW605051	QUE40297.1	1b	Npro	438/16	2016	-0.5197	0.3259	1.1276
Pestivirus A	MW605052	QUE40298.1	1b	Npro	439/16	2016	-0.5012	0.3192	1.1276
Pestivirus A	MW605053	QUE40299.1	1b	Npro	441/16	2016	-0.5012	0.3192	1.1276
Pestivirus A	MW605054	QUE40300.1	1b	Npro	504/16	2016	-0.5012	0.3192	1.1276
Pestivirus A	MW605055	QUE40301.1	1b	Npro	5/16	2016	-0.5012	0.3192	1.1276
Pestivirus A	KU856558	VIPR_ALG4_1016106647_	1d	Npro	71982/2011/PA	2011	-0.3968	0.3702	1.1123
Pestivirus A	KU856559	VIPR_ALG4_1016106649_	1d	Npro	71982/2011/2PA	2011	-0.3821	0.3620	1.1123
Pestivirus A	KU159365	VIPR_ALG4_1039262063_	1a	Npro	USII-S15	2015	-0.9207	0.3124	1.1207
Pestivirus A	KX218370	VIPR_ALG4_1063455487_	1o	Npro	HA2-12	2012	-0.0265	0.3502	1.1287
Pestivirus A	KX218371	VIPR_ALG4_1063455489_	1o	Npro	JS12/02	2011	-0.0265	0.3502	1.1287
Pestivirus A	KX218372	VIPR_ALG4_1063455491_	1b	Npro	JR1-2	2013	-0.3004	0.3222	1.1216
Pestivirus A	KU756226	VIPR_ALG4_1072900294_	1b	Npro	HJ-1	2010	-1.0021	0.2860	1.1184
Pestivirus A	KT943518	VIPR_ALG4_1093530908_	1d	Npro	BJ1201	2012	-0.5033	0.3778	1.1123
Pestivirus A	KX890141	VIPR_ALG4_1109523065_	1h	Npro	Bov/Ita/124.15-14	2015	-0.4354	0.4506	1.1223
Pestivirus A	LT631725	VIPR_ALG4_1112914034_	1h	Npro	UM/126/07	2007	-0.7864	0.2882	1.1250
Pestivirus A	KX170302	VIPR_ALG4_1129879870_	1b	Npro	V015	2001	-0.4203	0.2883	1.1184
Pestivirus A	KX170303	VIPR_ALG4_1129879872_	1b	Npro	V060	2004	-0.6920	0.2927	1.1260
Pestivirus A	KX170304	VIPR_ALG4_1129879874_	1b	Npro	V075	2011	-0.6952	0.2625	1.1200
Pestivirus A	KX170305	VIPR_ALG4_1129879876_	1b	Npro	V100	1997	-0.7074	0.3098	1.1206
Pestivirus A	KX170306	VIPR_ALG4_1129879878_	1b	Npro	V036	2007	-0.4525	0.2810	1.1176
Pestivirus A	KX170307	VIPR_ALG4_1129879880_	1b	Npro	V070	2007	-0.7908	0.2635	1.1184
Pestivirus A	KX170308	VIPR_ALG4_1129879882_	1b	Npro	V098	1999	-0.6819	0.3620	1.1184
Pestivirus A	KX170309	VIPR_ALG4_1129879884_	1b	Npro	V020	2005	-0.7063	0.3072	1.1184
Pestivirus A	KX170310	VIPR_ALG4_1129879886_	1b	Npro	V029	2006	-0.7203	0.3103	1.1184
Pestivirus A	KX170311	VIPR_ALG4_1129879888_	1b	Npro	V045	2009	-0.7432	0.3260	1.1137
Pestivirus A	KX170312	VIPR_ALG4_1129879890_	1b	Npro	V078	2012	-0.8019	0.2892	1.1184
Pestivirus A	KX170313	VIPR_ALG4_1129879892_	1b	Npro	V031	2006	-0.7866	0.2891	1.1137
Pestivirus A	KX170314	VIPR_ALG4_1129879894_	1b	Npro	V087	2006	-0.6607	0.2898	1.1160
Pestivirus A	KX170315	VIPR_ALG4_1129879896_	1a	Npro	V092	2004	-0.7799	0.3852	1.1206
Pestivirus A	KX170316	VIPR_ALG4_1129879898_	1a	Npro	V054	2013	-0.8197	0.3215	1.1203
Pestivirus A	KX170317	VIPR_ALG4_1129879900_	1a	Npro	V057	2009	-0.7962	0.3427	1.1206
Pestivirus A	KX170318	VIPR_ALG4_1129879902_	1a	Npro	V056	2009	-0.7103	0.3431	1.1216
Pestivirus A	KX170319	VIPR_ALG4_1129879904_	1a	Npro	V006	2000	-0.8528	0.3580	1.1216
Pestivirus A	KX170320	VIPR_ALG4_1129879906_	1a	Npro	V011	2001	-0.7878	0.3762	1.1224
Pestivirus A	KX170321	VIPR_ALG4_1129879908_	1a	Npro	V012	2001	-0.7123	0.3649	1.1224
Pestivirus A	KX170322	VIPR_ALG4_1129879910_	1a	Npro	V022	2006	-0.9057	0.3173	1.1179
Pestivirus A	KX170323	VIPR_ALG4_1129879912_	1a	Npro	V014	2001	-0.6749	0.3245	1.1206

Species according to VIPRBRC	GenBank Accession	GenBank Protein Accession	Subgenotype	Protein	Strain Name	Collection Year	SVM Patho. Score	Vaxijen Antig. Score	Averged score of EMBOSS motifs
Pestivirus A	KX170324	VIPR_ALG4_1129879914_	1a	Npro	V083	2008	-0.6311	0.3171	1.1221
Pestivirus A	KX170325	VIPR_ALG4_1129879916_	1a	Npro	V048	2009	-0.6093	0.3386	1.1224
Pestivirus A	KX170326	VIPR_ALG4_1129879918_	1a	Npro	V059	2004	-0.6093	0.3386	1.1224
Pestivirus A	KX170327	VIPR_ALG4_1129879920_	1a	Npro	V099	1998	-0.8150	0.2763	1.1226
Pestivirus A	KX170328	VIPR_ALG4_1129879922_	1a	Npro	V001	1999	-0.6002	0.3417	1.1233
Pestivirus A	KX170329	VIPR_ALG4_1129879924_	1a	Npro	V016	2002	-0.3553	0.3434	1.1226
Pestivirus A	KX170330	VIPR_ALG4_1129879926_	1a	Npro	V008	2000	-0.4256	0.3492	1.1158
Pestivirus A	KX170331	VIPR_ALG4_1129879928_	1a	Npro	V009	2000	-0.4256	0.3492	1.1158
Pestivirus A	KX170332	VIPR_ALG4_1129879930_	1a	Npro	V010	2001	-0.4715	0.3512	1.1226
Pestivirus A	KX170333	VIPR_ALG4_1129879932_	1a	Npro	V035	2007	-0.4715	0.3512	1.1226
Pestivirus A	KX170334	VIPR_ALG4_1129879934_	1a	Npro	V039	2008	-0.4715	0.3512	1.1226
Pestivirus A	KX170335	VIPR_ALG4_1129879936_	1a	Npro	V041	2008	-0.4715	0.3512	1.1226
Pestivirus A	KX170336	VIPR_ALG4_1129879938_	1a	Npro	V042	2008	-0.5386	0.3461	1.1253
Pestivirus A	KX170337	VIPR_ALG4_1129879940_	1a	Npro	V043	2008	-0.5386	0.3461	1.1253
Pestivirus A	KX170338	VIPR_ALG4_1129879942_	1a	Npro	V050	2009	-0.4715	0.3512	1.1226
Pestivirus A	KX170339	VIPR_ALG4_1129879944_	1a	Npro	V040	2008	-0.4715	0.3512	1.1226
Pestivirus A	KX170340	VIPR_ALG4_1129879946_	1a	Npro	V046	2009	-0.4715	0.3512	1.1226
Pestivirus A	KX170341	VIPR_ALG4_1129879948_	1a	Npro	V052	2010	-0.4715	0.3512	1.1226
Pestivirus A	KX170342	VIPR_ALG4_1129879950_	1a	Npro	V026	2006	-0.6842	0.2800	1.1218
Pestivirus A	KX170343	VIPR_ALG4_1129879952_	1a	Npro	V027	2006	-0.6842	0.2800	1.1218
Pestivirus A	KX170344	VIPR_ALG4_1129879954_	1a	Npro	V080	2009	-0.9386	0.3243	1.1341
Pestivirus A	KX170345	VIPR_ALG4_1129879956_	1a	Npro	V091	2003	-0.8908	0.3330	1.1300
Pestivirus A	KX170346	VIPR_ALG4_1129879958_	1a	Npro	V049	2009	-0.9333	0.3202	1.1341
Pestivirus A	KX170347	VIPR_ALG4_1129879960_	1a	Npro	V077	2012	-0.9333	0.3202	1.1341
Pestivirus A	KX170348	VIPR_ALG4_1129879962_	1a	Npro	V073	2011	-1.0232	0.3214	1.1296
Pestivirus A	KX170349	VIPR_ALG4_1129879964_	1a	Npro	V007	2000	-1.0232	0.3214	1.1296
Pestivirus A	KX170350	VIPR_ALG4_1129879966_	1a	Npro	V013	2001	-1.0232	0.3214	1.1296
Pestivirus A	KX170351	VIPR_ALG4_1129879968_	1a	Npro	V033	2007	-1.0232	0.3214	1.1296
Pestivirus A	KX170352	VIPR_ALG4_1129879970_	1a	Npro	V034	2007	-1.0232	0.3214	1.1296
Pestivirus A	KX170353	VIPR_ALG4_1129879972_	1a	Npro	V067	2006	-1.0232	0.3214	1.1296
Pestivirus A	KX170354	VIPR_ALG4_1129879974_	1a	Npro	V074	2010	-1.0232	0.3214	1.1296
Pestivirus A	EF101530	VIPR_ALG4_118498779_3	1b	Npro	KE9	2007	-0.4566	0.2915	1.1243
Pestivirus A	DQ088995	VIPR_ALG4_145309048_3	1a	Npro	Singer_Arg	1974	-0.9333	0.3202	1.1341
Pestivirus A	U63479	VIPR_ALG4_1518836_369	1b	Npro	CP7	1987	-0.7026	0.2951	1.1184
Pestivirus A	FJ387232	VIPR_ALG4_212657541_2	1b	Npro	NY-1	1962	-0.7458	0.2669	1.1211
Pestivirus A	FJ387233	VIPR_ALG4_212657545_1	1b	Npro	Bootes	2005	-0.7536	0.2944	1.1129
Pestivirus A	FJ387234	VIPR_ALG4_212657548_1	1b	Npro	Camelo	2005	-0.7536	0.2944	1.1129
Pestivirus A	FJ387235	VIPR_ALG4_212657550_1	1b	Npro	Cepheus	2005	-0.7536	0.2944	1.1129
Pestivirus A	FJ387236	VIPR_ALG4_212657553_1	1b	Npro	Chara	2005	-0.7536	0.2944	1.1129
Pestivirus A	FJ387237	VIPR_ALG4_212657555_1	1b	Npro	Columba	2005	-0.7536	0.2944	1.1129
Pestivirus A	FJ387238	VIPR_ALG4_212657558_1	1b	Npro	Corona	2005	-0.7536	0.2944	1.1129
Pestivirus A	FJ387239	VIPR_ALG4_212657560_1	1b	Npro	Dorado	2005	-0.7536	0.2944	1.1129
Pestivirus A	FJ387240	VIPR_ALG4_212657562_1	1b	Npro	Draco	2005	-0.7536	0.2944	1.1129
Pestivirus A	FJ387241	VIPR_ALG4_212657565_1	1b	Npro	Gemini	2005	-0.7536	0.2944	1.1129
Pestivirus A	FJ387242	VIPR_ALG4_212657568_1	1b	Npro	Gomeisa	2005	-0.7536	0.2944	1.1129
Pestivirus A	FJ387243	VIPR_ALG4_212657571_1	1b	Npro	Hamel	2005	-0.7536	0.2944	1.1129
Pestivirus A	FJ387244	VIPR_ALG4_212657574_1	1b	Npro	Hercules	2006	-0.6958	0.2732	1.1234

Species according to VIPRBRC	GenBank Accession	GenBank Protein Accession	Subgenotype	Protein	Strain Name	Collection Year	SVM Patho. Score	Vaxijen Antig. Score	Averged score of EMBOSS motifs
Pestivirus A	FJ387245	VIPR_ALG4_212657577_1	1b	Npro	Kurhah	2006	-0.7536	0.2944	1.1129
Pestivirus A	FJ387246	VIPR_ALG4_212657580_1	1b	Npro	Leo	2006	-0.7536	0.2944	1.1129
Pestivirus A	FJ387247	VIPR_ALG4_212657583_1	1b	Npro	Libra	2006	-0.7536	0.2944	1.1129
Pestivirus A	FJ387248	VIPR_ALG4_212657586_1	1b	Npro	Lyra	2006	-0.7536	0.2944	1.1129
Pestivirus A	FJ387249	VIPR_ALG4_212657589_1	1b	Npro	Mars	2006	-0.7536	0.2944	1.1129
Pestivirus A	FJ387250	VIPR_ALG4_212657592_1	1b	Npro	Mensa	2006	-0.7536	0.2944	1.1129
Pestivirus A	FJ387251	VIPR_ALG4_212657596_1	1b	Npro	Mercury	2006	-0.7494	0.2955	1.1129
Pestivirus A	FJ387252	VIPR_ALG4_212657599_1	1b	Npro	Musca	2006	-0.7536	0.2944	1.1129
Pestivirus A	FJ387253	VIPR_ALG4_212657602_1	1b	Npro	Nakkar	2006	-0.7536	0.2944	1.1129
Pestivirus A	FJ387254	VIPR_ALG4_212657605_1	1b	Npro	Pegasus	2006	-0.7536	0.2944	1.1129
Pestivirus A	FJ387255	VIPR_ALG4_212657608_1	1b	Npro	Pluto	2006	-0.7536	0.2944	1.1129
Pestivirus A	FJ387256	VIPR_ALG4_212657611_1	1b	Npro	Sadaton	2006	-0.7536	0.2944	1.1129
Pestivirus A	FJ387257	VIPR_ALG4_212657614_1	1b	Npro	Saturn	2006	-0.7536	0.2944	1.1129
Pestivirus A	FJ387258	VIPR_ALG4_212657617_1	1b	Npro	Scorpius	2006	-0.7536	0.2944	1.1129
Pestivirus A	FJ387259	VIPR_ALG4_212657620_1	1b	Npro	Sirrah	2006	-0.7536	0.2944	1.1129
Pestivirus A	FJ387260	VIPR_ALG4_212657623_1	1b	Npro	Taurus	2006	-0.7536	0.2944	1.1129
Pestivirus A	FJ387261	VIPR_ALG4_212657626_1	1b	Npro	Tucan	2006	-0.7536	0.2944	1.1129
Pestivirus A	FJ387262	VIPR_ALG4_212657629_1	1b	Npro	Venus	2004	-0.6796	0.2922	1.1129
Pestivirus A	FJ387263	VIPR_ALG4_212657633_1	1b	Npro	Virgo	2004	-0.7536	0.2944	1.1129
Pestivirus A	FJ387264	VIPR_ALG4_212657636_1	1b	Npro	Eridanus	2005	-0.7536	0.2944	1.1129
Pestivirus A	U86600	VIPR_ALG4_2149469_385	1b	Npro	ILLNC	1991	-0.9168	0.3475	1.1207
Pestivirus A	GU120258	VIPR_ALG4_268527799_1	1m	Npro	TY05	2005	-0.7822	0.3325	1.1304
Pestivirus A	GU120259	VIPR_ALG4_268527801_1	1p	Npro	BJ0701	2007	-0.6434	0.2778	1.1346
Pestivirus A	GU120260	VIPR_ALG4_268527803_1	1p	Npro	BJ0702	2007	-0.5139	0.3147	1.1350
Pestivirus A	GU120261	VIPR_ALG4_268527805_1	1p	Npro	BJ0703	2007	-0.7388	0.2736	1.1346
Pestivirus A	GU120262	VIPR_ALG4_268527807_1	1m	Npro	TJ0801	2008	-0.4850	0.3690	1.1241
Pestivirus A	AF041040	VIPR_ALG4_2789677_370	1a	Npro	Oregon	1960	-0.6093	0.3386	1.1224
Pestivirus A	M96751	VIPR_ALG4_289508_386_	1a	Npro	UNKNOWN-M96751	1992	-0.4715	0.3512	1.1226
Pestivirus A	GU991550	VIPR_ALG4_293338762_1	1b	Npro	BSU1	2008	-0.4747	0.3515	1.1307
Pestivirus A	HQ174292	VIPR_ALG4_323145267_1	1a	Npro	180	2010	-0.8987	0.3306	1.1267
Pestivirus A	M31182	VIPR_ALG4_323206_386_	1a	Npro	UNKNOWN-M31182	1988	-0.6799	0.2869	1.1218
Pestivirus A	M96687	VIPR_ALG4_323230_384_	1b	Npro	Osloss	1967	-0.5659	0.2939	1.1205
Pestivirus A	JN400273	VIPR_ALG4_363990275_1	1q	Npro	SD0803	2008	-0.7653	0.3198	1.1392
Pestivirus A	AF091605	VIPR_ALG4_3661566_386	1a	Npro	Oregon C24V	1960	-0.6093	0.3386	1.1224
Pestivirus A	JN644055	VIPR_ALG4_373939303_1	1b	Npro	3156	2011	-0.3754	0.3087	1.1216
Pestivirus A	JN380080	VIPR_ALG4_378753653_1	1a	Npro	6010	2010	-0.8987	0.3306	1.1267
Pestivirus A	JQ071526	VIPR_ALG4_380719827_1	1c	Npro	GS1	2010	-0.6692	0.2516	1.1213
Pestivirus A	JQ071528	VIPR_ALG4_380719830_1	1c	Npro	GS2	2010	-0.5624	0.2886	1.1306
Pestivirus A	JQ799141	VIPR_ALG4_390132765_2	1u	Npro	M31182	2010	-0.6902	0.3773	1.1366
Pestivirus A	JX419397	VIPR_ALG4_404363562_2	1b	Npro	UNKNOWN-JX419397	2008	-0.4777	0.3375	1.1120
Pestivirus A	JX419398	VIPR_ALG4_404363564_2	1b	Npro	UNKNOWN-JX419398	2008	-0.5315	0.3133	1.1129
Pestivirus A	AF526381	VIPR_ALG4_42476348_33	1m	Npro	ZM-95	1995	-0.5179	0.3882	1.1299
Pestivirus A	KC207068	VIPR_ALG4_455898601_1	1a	Npro	1	2010	-0.7295	0.3901	1.1256
Pestivirus A	KC207069	VIPR_ALG4_455898603_1	1a	Npro	2	2010	-0.6624	0.3831	1.1254
Pestivirus A	KC207070	VIPR_ALG4_455898605_1	1c	Npro	3	2010	-0.7694	0.3044	1.1279
Pestivirus A	KC207071	VIPR_ALG4_455898607_1	1p	Npro	5	2010	-0.7503	0.3120	1.1345
Pestivirus A	KC207072	VIPR_ALG4_455898609_1	1q	Npro	6	2010	-0.8207	0.2540	1.1306

Species according to VIPRBRC	GenBank Accession	GenBank Protein Accession	Subgenotype	Protein	Strain Name	Collection Year	SVM Patho. Score	Vaxijen Antig. Score	Averged score of EMBOSS motifs
Pestivirus A	KC207073	VIPR_ALG4_455898611_1	1o	Npro	9	2010	-0.8561	0.3400	1.1182
Pestivirus A	KC207075	VIPR_ALG4_455898615_1	1m	Npro	11	2010	-0.6051	0.3649	1.1343
Pestivirus A	KC207076	VIPR_ALG4_455898617_1	1b	Npro	12	2010	-0.5198	0.3050	1.1168
Pestivirus A	JX297512	VIPR_ALG4_459284067_1	1b	Npro	10270	2007	-0.7536	0.2944	1.1129
Pestivirus A	JX297513	VIPR_ALG4_459284069_1	1b	Npro	Aries	2005	-0.7536	0.2944	1.1129
Pestivirus A	JX297514	VIPR_ALG4_459284071_1	1b	Npro	Columba	2005	-0.7536	0.2944	1.1129
Pestivirus A	JX297515	VIPR_ALG4_459284073_1	1b	Npro	Corona	2005	-0.7536	0.2944	1.1129
Pestivirus A	JX297516	VIPR_ALG4_459284075_1	1b	Npro	Gemini	2005	-0.7536	0.2944	1.1129
Pestivirus A	JX297517	VIPR_ALG4_459284077_1	1b	Npro	Hercules	2006	-0.6958	0.2732	1.1234
Pestivirus A	JX297518	VIPR_ALG4_459284079_1	1b	Npro	Leo	2006	-0.7536	0.2944	1.1129
Pestivirus A	JX297519	VIPR_ALG4_459284081_1	1b	Npro	Lyra	2006	-0.7536	0.2944	1.1129
Pestivirus A	JX297520	VIPR_ALG4_459284083_1	1b	Npro	Mars	2006	-0.7536	0.2944	1.1129
Pestivirus A	JX297521	VIPR_ALG4_459284085_1	1b	Npro	Scorpius	2006	-0.7536	0.2944	1.1129
Pestivirus A	JX306011	VIPR_ALG4_459284087_1	1b	Npro	Cepheus	2005	-0.7536	0.2944	1.1129
Pestivirus A	JX306012	VIPR_ALG4_459284089_1	1b	Npro	Hamal	2006	-0.7536	0.2944	1.1129
Pestivirus A	JX306013	VIPR_ALG4_459284091_1	1b	Npro	Kurhah	2006	-0.7536	0.2944	1.1129
Pestivirus A	JX306014	VIPR_ALG4_459284093_1	1b	Npro	Naos	2006	-0.7536	0.2944	1.1129
Pestivirus A	KC414582	VIPR_ALG4_471271116_1	1q	Npro	Zhiduo17	2011	-0.7496	0.3103	1.1263
Pestivirus A	KC414583	VIPR_ALG4_471271118_1	1q	Npro	Zhiduo11	2011	-0.7296	0.2992	1.1263
Pestivirus A	KC414584	VIPR_ALG4_471271120_1	1b	Npro	Dari85	2011	-0.2550	0.3148	1.1264
Pestivirus A	KC414585	VIPR_ALG4_471271122_1	1b	Npro	Zeku33	2010	-0.4049	0.3050	1.1216
Pestivirus A	KC414586	VIPR_ALG4_471271124_1	1b	Npro	Dari98	2011	-0.3660	0.3182	1.1184
Pestivirus A	KC414587	VIPR_ALG4_471271126_1	1b	Npro	Zeku26	2010	-0.4407	0.3225	1.1216
Pestivirus A	KC414588	VIPR_ALG4_471271128_1	1q	Npro	Yushu2219	2012	-0.6145	0.3249	1.1238
Pestivirus A	KC414589	VIPR_ALG4_471271130_1	1d	Npro	DulanD62	2011	-0.5230	0.3514	1.1167
Pestivirus A	KC414590	VIPR_ALG4_471271132_1	1d	Npro	DulanD64	2011	-0.5230	0.3514	1.1167
Pestivirus A	KC414591	VIPR_ALG4_471271134_1	1q	Npro	Xinghai6007	2011	-0.7814	0.2807	1.1274
Pestivirus A	KC414592	VIPR_ALG4_471271136_1	1q	Npro	Xinghai6003	2011	-0.7306	0.2957	1.1227
Pestivirus A	KC414593	VIPR_ALG4_471271138_1	1q	Npro	Zhiduo28	2011	-0.8678	0.2832	1.1269
Pestivirus A	KC414594	VIPR_ALG4_471271140_1	1d	Npro	Yushu2158	2012	-0.4735	0.3536	1.1164
Pestivirus A	KC414595	VIPR_ALG4_471271142_1	1d	Npro	Yushu2121	2012	-0.5230	0.3514	1.1167
Pestivirus A	KC414596	VIPR_ALG4_471271144_1	1d	Npro	DulanD44	2011	-0.4826	0.3139	1.1167
Pestivirus A	KC414597	VIPR_ALG4_471271146_1	1q	Npro	Yushu2202	2012	-0.6675	0.3199	1.1207
Pestivirus A	KC853440	VIPR_ALG4_507144146_1	1k	Npro	SuwaNcp	1993	-0.8643	0.3544	1.1151
Pestivirus A	KC853441	VIPR_ALG4_507144148_1	1k	Npro	SuwaCp	1993	-0.8643	0.3544	1.1151
Pestivirus A	KC695810	VIPR_ALG4_507866685_1	1q	Npro	camel-6	2010	-0.7296	0.2608	1.1257
Pestivirus A	KC695811	VIPR_ALG4_507866687_1	1q	Npro	GS-3	2012	-0.6030	0.2575	1.1325
Pestivirus A	KC695812	VIPR_ALG4_507866689_1	1q	Npro	HB-1	2012	-0.8418	0.2904	1.1207
Pestivirus A	KC695813	VIPR_ALG4_507866693_1	1c	Npro	Bega-like	2012	-0.7228	0.3650	1.1259
Pestivirus A	KC695814	VIPR_ALG4_507866704_1	1b	Npro	Av69 VEDEVAC	2011	-0.3754	0.3087	1.1216
Pestivirus A	KC695815	VIPR_ALG4_507866706_1	1a	Npro	Av69 SD-1	2011	-0.6001	0.3516	1.1134
Pestivirus A	KC695816	VIPR_ALG4_507866709_1	1d	Npro	cell-con-1	2012	-0.4655	0.3755	1.1123
Pestivirus A	KC700344	VIPR_ALG4_508083101_1	1b	Npro	GS-4	2012	-0.7021	0.3132	1.1243
Pestivirus A	KC757383	VIPR_ALG4_511775165_1	1d	Npro	10JJ-SKR	2010	-0.4655	0.3755	1.1123
Pestivirus A	KC963967	VIPR_ALG4_530291194_1	1b	Npro	12F004	2012	-0.9399	0.2642	1.1184
Pestivirus A	KF154776	VIPR_ALG4_562744970_1	1l	Npro	TR72	2007	-0.7378	0.3031	1.1093
Pestivirus A	KF154777	VIPR_ALG4_562744972_1	1p	Npro	TR73	2007	-0.9512	0.2828	1.1365

Species according to VIPRBRC	GenBank Accession	GenBank Protein Accession	Subgenotype	Protein	Strain Name	Collection Year	SVM Patho. Score	Vaxijen Antig. Score	Averged score of EMBOSS motifs
Pestivirus A	KF154778	VIPR_ALG4_562744974_1	1p	Npro	TR75	2007	-0.9512	0.2828	1.1365
Pestivirus A	KF154779	VIPR_ALG4_562744976_1	1p	Npro	TR70	2007	-0.9512	0.2828	1.1365
Pestivirus A	KF772785	VIPR_ALG4_575471151_3	1b	Npro	CC13B	2013	-0.7046	0.2904	1.1184
Pestivirus A	KF896608	VIPR_ALG4_586616532_2	1c	Npro	Bega-like	2012	-0.7228	0.3650	1.1259
Pestivirus A	KF835697	VIPR_ALG4_597437474_2	1b	Npro	AU526	2013	-0.7173	0.2804	1.1221
Pestivirus A	KJ541471	VIPR_ALG4_633265982_2	1a	Npro	GS5	2013	-0.9176	0.3897	1.1276
Pestivirus A	KJ689448	VIPR_ALG4_635172915_3	1b	Npro	GX4	2012	-0.3754	0.3087	1.1216
Pestivirus A	KF501393	VIPR_ALG4_669206614_3	1b	Npro	BVDV JL-1	2009	-0.7458	0.2669	1.1211
Pestivirus A	KM261881	VIPR_ALG4_697348509_1	1b	Npro	IndMDV18697/12	2012	-0.3862	0.3108	1.1227
Pestivirus A	AJ133738	VIPR_ALG4_7960754_38	1a	Npro	type 1	1963	-0.6842	0.2800	1.1218
Pestivirus A	KP941581	VIPR_ALG4_800924313_3	1b	Npro	USMARC-51998	2014	-0.8784	0.2898	1.1204
Pestivirus A	KP941583	VIPR_ALG4_800924317_3	1b	Npro	USMARC-53874	2014	-0.5917	0.2966	1.1184
Pestivirus A	KP941584	VIPR_ALG4_800924319_3	1a	Npro	USMARC-53875	2014	-0.6966	0.3000	1.1177
Pestivirus A	KP941586	VIPR_ALG4_800924323_3	1a	Npro	USMARC-55477	2014	-0.7770	0.3607	1.1207
Pestivirus A	KP941587	VIPR_ALG4_800924325_3	1b	Npro	USMARC-55478	2014	-0.8592	0.2878	1.1181
Pestivirus A	KP941588	VIPR_ALG4_800924327_3	1b	Npro	USMARC-55922	2014	-0.8041	0.2719	1.1184
Pestivirus A	KP941589	VIPR_ALG4_800924329_3	1b	Npro	USMARC-55923	2014	-0.8808	0.2591	1.1160
Pestivirus A	KP941590	VIPR_ALG4_800924331_3	1b	Npro	USMARC-55924	2014	-0.7379	0.2773	1.1184
Pestivirus A	KP941591	VIPR_ALG4_800924333_3	1b	Npro	USMARC-55925	2014	-0.6075	0.2832	1.1184
Pestivirus A	KP941592	VIPR_ALG4_800924335_3	1b	Npro	USMARC-55926	2014	-0.4870	0.3140	1.1179
Pestivirus A	KP313732	VIPR_ALG4_816850387_3	1e	Npro	Carlito	2014	-0.6421	0.3436	1.1204
Pestivirus A	KR013753	VIPR_ALG4_871332680_3	1a	Npro	WAX-N	1992	-0.5300	0.3550	1.1231
Pestivirus A	KR029825	VIPR_ALG4_887497286_3	1b	Npro	Egy/Ismailia/2014	2014	-0.9415	0.2977	1.1217
Pestivirus A	LC089875	VIPR_ALG4_939106262_3	1o	Npro	IS26/01ncp	2001	-0.3574	0.3821	1.1287
Pestivirus A	LC089876	VIPR_ALG4_939106264_3	1n	Npro	Shitara/02/06	2006	-0.7794	0.3138	1.1169
Pestivirus A	KR866116	VIPR_ALG4_941508008_3	1m	Npro	SD-15	2015	-0.5921	0.3689	1.1193
Pestivirus A	KU200260	VIPR_ALG4_972905813_3	1b	Npro	BE/061536/2014	2014	-0.5594	0.3041	1.1309
Pestivirus A	KX577637	VIPR_ALG4_AOR50934_1	1e	Npro	SLO/2407/2006	2006	-0.4226	0.2641	1.1243
Pestivirus A	KX987157	VIPR_ALG4_APG30987_1	1f	Npro	SLO/1170/2000	2000	-0.7299	0.3032	1.1167
Pestivirus A	KX857724	VIPR_ALG4_APZ85839_1	1i	Npro	ACM/BR/2016	2016	-1.0587	0.2348	1.1273
Pestivirus A	KY488631	VIPR_ALG4_AQS23377_1	1l	Npro	FarsA	2014	-1.0033	0.2603	1.1327
Pestivirus A	KY488632	VIPR_ALG4_AQS23378_1	1b	Npro	FarsB	2014	-0.7138	0.3163	1.1129
Pestivirus A	KY849592	VIPR_ALG4_ART90617_1	1d	Npro	SLO/2416/2002	2002	-0.4119	0.3083	1.1194
Pestivirus A	MF278651	VIPR_ALG4_ASW18434_1	1b	Npro	XZ01	2016	-0.3754	0.3087	1.1216
Pestivirus A	MF278652	VIPR_ALG4_ASW18435_1	1b	Npro	XZ02	2016	-0.3754	0.3087	1.1216
Pestivirus A	MF693403	VIPR_ALG4_ATG71375_1	1a	Npro	UNKNOWN-MF693403	2016	-1.0127	0.2834	1.1226
Pestivirus A	KY964311	VIPR_ALG4_ATN39078_1	1b	Npro	Y2	2014	-0.4805	0.3048	1.1241
Pestivirus A	MF172980	VIPR_ALG4_AVI10261_1_	1c	Npro	GSTZ	2012	-0.5516	0.3003	1.1371
Pestivirus A	MH379638	VIPR_ALG4_AWW14171_	1a	Npro	Ho916	1993	-0.9709	0.2608	1.1230
Pestivirus A	MG950344	VIPR_ALG4_AWW87346_	1b	Npro	AU526	2014	-0.7173	0.2804	1.1221
Pestivirus A	MG950345	VIPR_ALG4_AWW87347_	1b	Npro	B1	2015	-0.7173	0.2804	1.1221
Pestivirus A	MG950346	VIPR_ALG4_AWW87348_	1b	Npro	B2	2015	-0.7821	0.2853	1.1221
Pestivirus A	MG950347	VIPR_ALG4_AWW87349_	1b	Npro	B3	2015	-0.7056	0.2823	1.1203
Pestivirus A	MG950348	VIPR_ALG4_AWW87350_	1b	Npro	B4	2015	-0.7056	0.2823	1.1203
Pestivirus A	MG950349	VIPR_ALG4_AWW87351_	1b	Npro	B5	2015	-0.6586	0.2792	1.1259
Pestivirus A	MG950350	VIPR_ALG4_AWW87352_	1b	Npro	B6	2015	-0.7056	0.2823	1.1203
Pestivirus A	MG950351	VIPR_ALG4_AWW87353_	1b	Npro	O1	2015	-0.7173	0.2804	1.1221

Species according to VIPRBRC	GenBank Accession	GenBank Protein Accession	Subgenotype	Protein	Strain Name	Collection Year	SVM Patho. Score	Vaxijen Antig. Score	Averged score of EMBOSS motifs
Pestivirus A	MG950352	VIPR_ALG4_AWW87354_	1b	Npro	O2	2015	-0.7173	0.2804	1.1221
Pestivirus A	MG950353	VIPR_ALG4_AWW87355_	1b	Npro	O3	2015	-0.7173	0.2804	1.1221
Pestivirus A	MG950354	VIPR_ALG4_AWW87356_	1b	Npro	O4	2015	-0.7173	0.2804	1.1221
Pestivirus A	MG950355	VIPR_ALG4_AWW87357_	1b	Npro	O5	2015	-0.7173	0.2804	1.1221
Pestivirus A	MG950356	VIPR_ALG4_AWW87358_	1b	Npro	O6	2015	-0.7173	0.2804	1.1221
Pestivirus A	MG950357	VIPR_ALG4_AWW87359_	1b	Npro	B1A	2015	-0.7173	0.2804	1.1221
Pestivirus A	MG950358	VIPR_ALG4_AWW87360_	1b	Npro	B2A	2016	-0.7056	0.2823	1.1203
Pestivirus A	MG950359	VIPR_ALG4_AWW87361_	1b	Npro	B3A	2016	-0.7056	0.2823	1.1203
Pestivirus A	MG950360	VIPR_ALG4_AWW87362_	1b	Npro	B4A	2016	-0.7056	0.2823	1.1203
Pestivirus A	MG950361	VIPR_ALG4_AWW87363_	1b	Npro	B5A	2016	-0.7056	0.2823	1.1203
Pestivirus A	MG950362	VIPR_ALG4_AWW87364_	1b	Npro	B6A	2016	-0.7056	0.2823	1.1203
Pestivirus A	MG950363	VIPR_ALG4_AWW87365_	1b	Npro	O1A	2015	-0.8545	0.3023	1.1221
Pestivirus A	MG950364	VIPR_ALG4_AWW87366_	1b	Npro	O2A	2015	-0.7173	0.2804	1.1221
Pestivirus A	MG950365	VIPR_ALG4_AWW87367_	1b	Npro	O2B	2015	-0.7173	0.2804	1.1221
Pestivirus A	MG950366	VIPR_ALG4_AWW87368_	1b	Npro	O4A	2015	-0.7173	0.2804	1.1221
Pestivirus A	MH311874	VIPR_ALG4_AWW87369_	1b	Npro	B2A d168	2016	-0.7056	0.2823	1.1203
Pestivirus A	MH311875	VIPR_ALG4_AWW87370_	1b	Npro	B3A d168	2016	-0.7056	0.2823	1.1203
Pestivirus A	MH311876	VIPR_ALG4_AWW87371_	1b	Npro	B4A d84	2016	-0.7056	0.2823	1.1203
Pestivirus A	MH311877	VIPR_ALG4_AWW87372_	1b	Npro	B4A d168	2016	-0.7056	0.2823	1.1203
Pestivirus A	MH311878	VIPR_ALG4_AWW87373_	1b	Npro	B5A d84	2016	-0.7056	0.2823	1.1203
Pestivirus A	MH311879	VIPR_ALG4_AWW87374_	1b	Npro	B5A d168	2016	-0.7056	0.2823	1.1203
Pestivirus A	MH311880	VIPR_ALG4_AWW87375_	1b	Npro	B6A d84	2016	-0.7056	0.2823	1.1203
Pestivirus A	MH311881	VIPR_ALG4_AWW87376_	1b	Npro	B6A d168	2016	-0.7056	0.2823	1.1203
Pestivirus A	MH379221	VIPR_ALG4_AWW87377_	1b	Npro	P1	2017	-0.7173	0.2804	1.1221
Pestivirus A	MH379222	VIPR_ALG4_AWW87378_	1b	Npro	P2	2017	-0.7173	0.2804	1.1221
Pestivirus A	MH379223	VIPR_ALG4_AWW87379_	1b	Npro	P5	2017	-0.7173	0.2804	1.1221
Pestivirus A	MH379224	VIPR_ALG4_AWW87380_	1b	Npro	P6	2017	-0.7173	0.2804	1.1221
Pestivirus A	MH379225	VIPR_ALG4_AWW87381_	1b	Npro	P7	2017	-0.7173	0.2804	1.1221
Pestivirus A	MH379226	VIPR_ALG4_AWW87382_	1b	Npro	P5A	2017	-0.7173	0.2804	1.1221
Pestivirus A	MH379227	VIPR_ALG4_AWW87383_	1b	Npro	P5B	2017	-0.7173	0.2804	1.1221
Pestivirus A	MH379228	VIPR_ALG4_AWW87384_	1b	Npro	P5C	2017	-0.7173	0.2804	1.1221
Pestivirus A	MH379229	VIPR_ALG4_AWW87385_	1b	Npro	P5D	2017	-0.7173	0.2804	1.1221
Pestivirus A	MH379230	VIPR_ALG4_AWW87386_	1b	Npro	P5F	2017	-0.7173	0.2804	1.1221
Pestivirus A	MH379231	VIPR_ALG4_AWW87387_	1b	Npro	P7A	2018	-0.7173	0.2804	1.1221
Pestivirus A	MH379232	VIPR_ALG4_AWW87388_	1b	Npro	P7C	2018	-0.7173	0.2804	1.1221
Pestivirus A	MH379233	VIPR_ALG4_AWW87389_	1b	Npro	P7E	2018	-0.7173	0.2804	1.1221
Pestivirus A	MH379234	VIPR_ALG4_AWW87390_	1b	Npro	P7F	2018	-0.7173	0.2804	1.1221
Pestivirus A	MH166806	VIPR_ALG4_AYA62524_1	1m	Npro	XC	2015	-0.6118	0.3707	1.1343
Pestivirus A	MH490943	VIPR_ALG4_AZB53078_1	1b	Npro	BVDV BJ-2016	2016	-0.8821	0.2852	1.1184
Pestivirus A	MH231153	VIPR_ALG4_AZQ00677_1	1b	Npro	Nebraska	1990	-0.5288	0.3243	1.1086
Pestivirus A	AB078950	VIPR_ALG4_BAC55961_1	1j	Npro	KS86-1ncp	1986	-1.0298	0.2560	1.1216
Pestivirus A	MH899941	VIPR_ALG4_QCE30388_1	1b	Npro	SLO/3301/2014	2014	-0.7480	0.2849	1.1150
Pestivirus A	MH899942	VIPR_ALG4_QCE30389_1	1e	Npro	SLO/33529/2015	2015	-0.4753	0.2769	1.1243
Pestivirus A	MH899943	VIPR_ALG4_QCE30390_1	1f	Npro	SLO/1361/2014	2014	-0.8273	0.3480	1.1284
Pestivirus A	MH899944	VIPR_ALG4_QCE30391_1	1f	Npro	SLO/28537/2017	2017	-0.8482	0.3227	1.1219
Pestivirus A	MH899945	VIPR_ALG4_QCE30392_1	1h	Npro	SLO/1883/2013	2013	-0.7179	0.2808	1.1269
Pestivirus A	MK102095	VIPR_ALG4_QCQ84262_1	1q	Npro	20170226	2017	-0.9009	0.3097	1.1320

Species according to VIPRBRC	GenBank Accession	GenBank Protein Accession	Subgenotype	Protein	Strain Name	Collection Year	SVM Patho. Score	Vaxijen Antig. Score	Averged score of EMBOSS motifs
Pestivirus A	MK509774	VIPR_ALG4_QEK23510_1	1b	Npro	BVD1b-JH	2008	-0.7966	0.2576	1.1203
Pestivirus A	MK775204	VIPR_ALG4_QFX66041_1	1i	Npro	CA2006	2006	-0.8805	0.2336	1.1220
Pestivirus A	MK982965	VIPR_ALG4_QGX89883_1	1b	Npro	NSF116	2016	-0.8214	0.3373	1.1162
Pestivirus A	MK982966	VIPR_ALG4_QGX89884_1	1b	Npro	KSF1322	2015	-0.7773	0.3381	1.1316
Pestivirus A	MK982967	VIPR_ALG4_QGX89885_1	1b	Npro	KBF955	2016	-0.7606	0.3595	1.1278
Pestivirus A	MK982969	VIPR_ALG4_QGX89887_1	1l	Npro	ABF710	2016	-0.7036	0.3166	1.1228
Pestivirus A	MK982970	VIPR_ALG4_QGX89888_1	1j	Npro	NBF1383	2016	-0.8202	0.3265	1.1368
Pestivirus A	MN188073	VIPR_ALG4_QGZ19414_1	1a	Npro	PI34	2017	-0.1852	0.3111	1.1251
Pestivirus A	MN188074	VIPR_ALG4_QGZ19415_1	1b	Npro	PI285	2017	-1.0799	0.3104	1.1148
Pestivirus A	MN442389	VIPR_ALG4_QHG11656_1	1b	Npro	HN1437	2014	-0.4078	0.2869	1.1205
Pestivirus A	MN442390	VIPR_ALG4_QHG11657_1	1b	Npro	HN1506	2015	-0.3766	0.2643	1.1205
Pestivirus A	MN442391	VIPR_ALG4_QHG11658_1	1b	Npro	HN1522	2015	-0.4078	0.2869	1.1205
Pestivirus A	MN442392	VIPR_ALG4_QHG11659_1	1m	Npro	HN1539	2015	-0.6172	0.3683	1.1280
Pestivirus A	MN442393	VIPR_ALG4_QHG11660_1	1m	Npro	HN1613	2016	-0.5766	0.3828	1.1242
Pestivirus A	MN442394	VIPR_ALG4_QHG11661_1	1q	Npro	HN1618	2016	-0.8488	0.2785	1.1330
Pestivirus A	MN442395	VIPR_ALG4_QHG11662_1	1o	Npro	HN1626	2016	-0.6195	0.3143	1.1282
Pestivirus A	MN442396	VIPR_ALG4_QHG11663_1	1o	Npro	HN1641	2016	-0.5969	0.3097	1.1282
Pestivirus A	MN442397	VIPR_ALG4_QHG11664_1	1m	Npro	HN1711	2017	-0.5250	0.3838	1.1242
Pestivirus A	MN442398	VIPR_ALG4_QHG11665_1	1m	Npro	HN1720	2017	-0.5766	0.3828	1.1242
Pestivirus A	MN442399	VIPR_ALG4_QHG11666_1	1m	Npro	HN1725	2017	-0.6172	0.3683	1.1280
Pestivirus A	MN442400	VIPR_ALG4_QHG11667_1	1b	Npro	HN1727	2017	-0.4078	0.2869	1.1205
Pestivirus A	MN442401	VIPR_ALG4_QHG11668_1	1q	Npro	HN1729	2017	-0.8746	0.3185	1.1427
Pestivirus A	MN442402	VIPR_ALG4_QHG11669_1	1o	Npro	HN1732	2017	-0.6300	0.3131	1.1282
Pestivirus A	MN442403	VIPR_ALG4_QHG11670_1	1o	Npro	HN1736	2017	-0.6300	0.3131	1.1282
Pestivirus A	MN442404	VIPR_ALG4_QHG11671_1	1b	Npro	HN1753	2017	-0.5632	0.2994	1.1205
Pestivirus A	MN442405	VIPR_ALG4_QHG11672_1	1u	Npro	HN1802	2018	-0.5018	0.3703	1.1207
Pestivirus A	MN442406	VIPR_ALG4_QHG11673_1	1o	Npro	HN1814	2018	-0.6195	0.3143	1.1282
Pestivirus A	MN442407	VIPR_ALG4_QHG11674_1	1m	Npro	HN1821	2018	-0.5250	0.3838	1.1242
Pestivirus A	MN442408	VIPR_ALG4_QHG11675_1	1o	Npro	HN1852	2018	-0.7023	0.3166	1.1313
Pestivirus A	MN442409	VIPR_ALG4_QHG11676_1	1o	Npro	HN1859	2018	-0.7200	0.3275	1.1313
Pestivirus A	MN442410	VIPR_ALG4_QHG11677_1	1o	Npro	HN1864	2018	-0.6300	0.3131	1.1282
Pestivirus A	MN442411	VIPR_ALG4_QHG11678_1	1b	Npro	HN1877	2018	-0.5884	0.2846	1.1243
Pestivirus A	MN442412	VIPR_ALG4_QHG11679_1	1o	Npro	HN1918	2019	-0.7023	0.3166	1.1313
Pestivirus A	MN394766	VIPR_ALG4_QIB02049_1	1m	Npro	0001	2016	-0.6268	0.3565	1.1306
Pestivirus A	MT079816	VIPR_ALG4_QIM55913_1	1c	Npro	GXNN1	2018	-0.6622	0.2984	1.1259
Pestivirus A	MN623291	VIPR_ALG4_QLL27013_1	1m	Npro	NX2019/01	2019	-0.7601	0.3704	1.1304
Pestivirus A	MW014286	VIPR_ALG4_QPJ59878_1	1b	Npro	GXSS01	2018	-0.4322	0.3137	1.1216
Pestivirus A	MW014287	VIPR_ALG4_QPJ59879_1	1b	Npro	GXSS02	2018	-0.3288	0.3072	1.1269
Pestivirus A	MW014288	VIPR_ALG4_QPJ59880_1	1b	Npro	GXSS03	2018	-0.2762	0.3150	1.1250
Pestivirus A	MT024562	VIPR_ALG4_QPK41175_1	1b	Npro	190919	2019	-0.4746	0.2868	1.1243
Pestivirus A	MT024563	VIPR_ALG4_QPK41176_1	1b	Npro	230919	2019	-0.5280	0.2932	1.1243
Pestivirus A	MT024564	VIPR_ALG4_QPK41177_1	1a	Npro	ABART-2	2018	-0.8982	0.3199	1.1341
Pestivirus A	MT977117	VIPR_ALG4_QRZ20359_1	1b	Npro	BVDV 1b IT16/5	2016	-0.5012	0.3192	1.1276
Pestivirus A	MT977118	VIPR_ALG4_QRZ20360_1	1b	Npro	BVDV 1b IT16/439	2016	-0.5012	0.3192	1.1276
Pestivirus A	MT654137	VIPR_ALG4_QVK82311_1	1a	Npro	20-8536	2020	-0.7341	0.3215	1.1207
Pestivirus A	LT837585	VIPR_ALG4_SLV80196_1	1r	Npro	UNKNOWN-LT837585	2012	-0.5720	0.2671	1.1237
Pestivirus A	MT740275	VIPR_ALG4_UAD82114_1	1v	Npro	NX2019/02	2019	-0.5641	0.2242	1.1307

Species according to VIPRBRC	GenBank Accession	GenBank Protein Accession	Subgenotype	Protein	Strain Name	Collection Year	SVM Patho. Score	Vaxijen Antig. Score	Averged score of EMBOSS motifs
Pestivirus A	MW054933	VIPR_ALG4_UEC94252_1	1f	Npro	LA/230/14	2014	-0.5919	0.2615	1.1217
Pestivirus A	MW054934	VIPR_ALG4_UEC94253_1	1f	Npro	LA/87/05	2005	-0.7150	0.2429	1.1108
Pestivirus A	MW054935	VIPR_ALG4_UEC94254_1	1k	Npro	TO/197/11	2011	-0.9065	0.3378	1.1196
Pestivirus A	MW054936	VIPR_ALG4_UEC94255_1	1g	Npro	UM/111/06	2006	-0.7688	0.3243	1.1253
Pestivirus A	MW054937	VIPR_ALG4_UEC94256_1	1k	Npro	SA/158/09	2009	-0.6309	0.3428	1.1216
Pestivirus A	MW054938	VIPR_ALG4_UEC94257_1	1k	Npro	SA/159/09	2009	-0.6309	0.3428	1.1216
Pestivirus A	MW054939	VIPR_ALG4_UEC94258_1	1f	Npro	LO/151/09	2009	-0.6773	0.3017	1.1217
Pestivirus A	MW054940	VIPR_ALG4_UEC94259_1	1e	Npro	MA/101/05	2005	-0.6389	0.2701	1.1269
Pestivirus A	MW250796	VIPR_ALG4_UEC94260_1	1i	Npro	58-1	2008	-0.8550	0.2068	1.1237
Pestivirus A	MW250797	VIPR_ALG4_UEC94261_1	1i	Npro	58-2	2008	-0.8550	0.2068	1.1237
Pestivirus A	MW250798	VIPR_ALG4_UEC94262_1	1a	Npro	62-2	2008	-0.6401	0.3249	1.1224
Pestivirus A	MW250799	VIPR_ALG4_UEC94263_1	1a	Npro	63-1	2008	-0.7461	0.3269	1.1220
Pestivirus A	MW250800	VIPR_ALG4_UEC94264_1	1d	Npro	67-1	2008	-0.8224	0.3120	1.1196
Pestivirus A	MW250801	VIPR_ALG4_UEC94265_1	1d	Npro	67-2	2008	-0.8435	0.3144	1.1135
Pestivirus A	MW250802	VIPR_ALG4_UEC94266_1	1e	Npro	68-1	2008	-0.3043	0.2766	1.1289
Pestivirus A	MW250803	VIPR_ALG4_UEC94267_1	1i	Npro	69-1	2008	-0.9927	0.2191	1.1251
Pestivirus A	MW655625	VIPR_ALG4_UEC94268_1	1h	Npro	CH-04-01b	2004	-0.6206	0.2642	1.1200
Pestivirus A	MW655626	VIPR_ALG4_UEC94269_1	1e	Npro	Maria	2004	-0.7541	0.3309	1.1206
Pestivirus A	MW655627	VIPR_ALG4_UEC94270_1	1e	Npro	R2000-95	1995	-0.6101	0.2899	1.1255
Pestivirus A	MW655628	VIPR_ALG4_UEC94271_1	1k	Npro	R3230-95	1995	-0.5379	0.3481	1.1270
Pestivirus A	MW655629	VIPR_ALG4_UEC94272_1	1h	Npro	R3572-90	1990	-0.7194	0.2794	1.1250
Pestivirus A	MW655630	VIPR_ALG4_UEC94273_1	1k	Npro	R5013-96	1996	-0.9591	0.3511	1.1244
Pestivirus A	MW655631	VIPR_ALG4_UEC94274_1	1e	Npro	S03-1175	2003	-0.6749	0.3262	1.1280
Pestivirus A	MW655632	VIPR_ALG4_UEC94275_1	1h	Npro	SM09-20	2002	-0.7864	0.2882	1.1250
Pestivirus A	MW713361	VIPR_ALG4_UEC94276_1	1a	Npro	BoAEC1190	2007	-0.8370	0.3041	1.1250
Pestivirus A	MW713362	VIPR_ALG4_UEC94277_1	1b	Npro	PI819	2017	-0.7173	0.2804	1.1221
Pestivirus A	MW732738	VIPR_ALG4_UEC94278_1	1a	Npro	PI407	2015	-0.8620	0.3261	1.1380
Pestivirus A	MW732739	VIPR_ALG4_UEC94279_1	1a	Npro	YandaSpl	1993	-0.6947	0.3431	1.1267
Pestivirus A	MZ209052	VIPR_ALG4_UIB81631_1_	1l	Npro	TR-A2019-01	2019	-0.7216	0.3081	1.1245
Pestivirus A	MZ209055	VIPR_ALG4_UIB81634_1_	1b	Npro	TR-K2019-02	2019	-0.7266	0.3458	1.1278
Pestivirus A	MZ209056	VIPR_ALG4_UIB81635_1_	1j	Npro	TR-U2018-01	2018	-0.9618	0.3177	1.1368
Pestivirus A	MZ188972	VIPR_ALG4_UML14262_1	1q	Npro	HB-1	2020	-0.7070	0.3016	1.1203
Pestivirus A	ON337882	VIPR_ALG4_USZ80113_1_	1c	Npro	NM2103	2021	-0.5913	0.3084	1.1339
Pestivirus A	KU159365	VIPR_ALG4_1039262063_	1a	NS5A	USII-S15	2015	-0.7682	0.5197	1.1018
Pestivirus A	KU756226	VIPR_ALG4_1072900294_	1b	NS5A	HJ-1	2010	-0.7437	0.5438	1.1150
Pestivirus A	KT943518	VIPR_ALG4_1093530908_	1d	NS5A	BJ1201	2012	-1.0106	0.5032	1.1075
Pestivirus A	LT631725	VIPR_ALG4_1112914034_	1h	NS5A	UM/126/07	2007	-0.8119	0.5354	1.1029
Pestivirus A	KX170598	VIPR_ALG4_1129880462_	1b	NS5A	V015	2001	-0.9987	0.5644	1.1123
Pestivirus A	KX170599	VIPR_ALG4_1129880464_	1a	NS5A	V056	2009	-0.9141	0.5637	1.1136
Pestivirus A	KX170600	VIPR_ALG4_1129880466_	1b	NS5A	V075	2011	-0.7835	0.5539	1.1159
Pestivirus A	KX170601	VIPR_ALG4_1129880468_	1b	NS5A	V100	1997	-0.6911	0.5581	1.1170
Pestivirus A	KX170602	VIPR_ALG4_1129880470_	1b	NS5A	V060	2004	-0.7616	0.5520	1.1106
Pestivirus A	KX170603	VIPR_ALG4_1129880472_	1b	NS5A	V036	2007	-0.8269	0.5428	1.1137
Pestivirus A	KX170604	VIPR_ALG4_1129880474_	1b	NS5A	V020	2005	-0.7715	0.5400	1.1104
Pestivirus A	KX170605	VIPR_ALG4_1129880476_	1b	NS5A	V029	2006	-0.8171	0.5519	1.1102
Pestivirus A	KX170606	VIPR_ALG4_1129880478_	1b	NS5A	V078	2012	-0.7072	0.5462	1.1175
Pestivirus A	KX170607	VIPR_ALG4_1129880480_	1b	NS5A	V045	2009	-0.8126	0.5532	1.1132

Species according to VIPRBRC	GenBank Accession	GenBank Protein Accession	Subgenotype	Protein	Strain Name	Collection Year	SVM Patho. Score	Vaxijen Antig. Score	Averged score of EMBOSS motifs
Pestivirus A	KX170608	VIPR_ALG4_1129880482_	1b	NS5A	V031	2006	-0.7743	0.5506	1.1152
Pestivirus A	KX170609	VIPR_ALG4_1129880484_	1b	NS5A	V087	2006	-0.7134	0.5508	1.1161
Pestivirus A	KX170610	VIPR_ALG4_1129880486_	1b	NS5A	V070	2007	-0.8344	0.5569	1.1155
Pestivirus A	KX170611	VIPR_ALG4_1129880488_	1b	NS5A	V098	1999	-0.8917	0.5516	1.1159
Pestivirus A	KX170612	VIPR_ALG4_1129880490_	1a	NS5A	V083	2008	-0.8756	0.5213	1.1079
Pestivirus A	KX170613	VIPR_ALG4_1129880492_	1a	NS5A	V001	1999	-0.6920	0.5403	1.1008
Pestivirus A	KX170614	VIPR_ALG4_1129880494_	1a	NS5A	V010	2001	-0.8184	0.5667	1.1034
Pestivirus A	KX170615	VIPR_ALG4_1129880496_	1a	NS5A	V008	2000	-0.8241	0.5339	1.1026
Pestivirus A	KX170616	VIPR_ALG4_1129880498_	1a	NS5A	V009	2000	-0.8241	0.5339	1.1026
Pestivirus A	KX170617	VIPR_ALG4_1129880500_	1a	NS5A	V043	2008	-0.7272	0.5366	1.1042
Pestivirus A	KX170618	VIPR_ALG4_1129880502_	1a	NS5A	V046	2009	-0.7929	0.5330	1.1033
Pestivirus A	KX170619	VIPR_ALG4_1129880504_	1a	NS5A	V035	2007	-0.7929	0.5330	1.1033
Pestivirus A	KX170620	VIPR_ALG4_1129880506_	1a	NS5A	V052	2010	-0.8417	0.5349	1.1027
Pestivirus A	KX170621	VIPR_ALG4_1129880508_	1a	NS5A	V040	2008	-0.7929	0.5330	1.1033
Pestivirus A	KX170622	VIPR_ALG4_1129880510_	1a	NS5A	V042	2008	-0.7929	0.5330	1.1033
Pestivirus A	KX170623	VIPR_ALG4_1129880512_	1a	NS5A	V050	2009	-0.8119	0.5363	1.1063
Pestivirus A	KX170624	VIPR_ALG4_1129880514_	1a	NS5A	V039	2008	-0.7929	0.5330	1.1033
Pestivirus A	KX170625	VIPR_ALG4_1129880516_	1a	NS5A	V041	2008	-0.7929	0.5330	1.1033
Pestivirus A	KX170626	VIPR_ALG4_1129880518_	1a	NS5A	V059	2004	-0.9619	0.5264	1.1093
Pestivirus A	KX170627	VIPR_ALG4_1129880520_	1a	NS5A	V099	1998	-1.0105	0.4889	1.1066
Pestivirus A	KX170628	VIPR_ALG4_1129880522_	1a	NS5A	V026	2006	-0.7244	0.5015	1.1105
Pestivirus A	KX170629	VIPR_ALG4_1129880524_	1a	NS5A	V027	2006	-0.7244	0.5015	1.1105
Pestivirus A	KX170630	VIPR_ALG4_1129880526_	1a	NS5A	V080	2009	-0.7179	0.5185	1.1085
Pestivirus A	KX170631	VIPR_ALG4_1129880528_	1a	NS5A	V091	2003	-0.5850	0.5216	1.1055
Pestivirus A	KX170632	VIPR_ALG4_1129880530_	1a	NS5A	V073	2011	-0.6558	0.5116	1.1076
Pestivirus A	KX170633	VIPR_ALG4_1129880532_	1a	NS5A	V034	2007	-0.6848	0.5332	1.1068
Pestivirus A	KX170634	VIPR_ALG4_1129880534_	1a	NS5A	V049	2009	-0.6598	0.5038	1.1080
Pestivirus A	KX170635	VIPR_ALG4_1129880536_	1a	NS5A	V074	2010	-0.6781	0.5162	1.1068
Pestivirus A	KX170636	VIPR_ALG4_1129880538_	1a	NS5A	V007	2000	-0.6781	0.5162	1.1068
Pestivirus A	KX170637	VIPR_ALG4_1129880540_	1a	NS5A	V013	2001	-0.6781	0.5162	1.1068
Pestivirus A	KX170638	VIPR_ALG4_1129880542_	1a	NS5A	V033	2007	-0.6781	0.5162	1.1068
Pestivirus A	KX170639	VIPR_ALG4_1129880544_	1a	NS5A	V067	2006	-0.6781	0.5162	1.1068
Pestivirus A	KX170640	VIPR_ALG4_1129880546_	1a	NS5A	V077	2012	-0.6781	0.5162	1.1068
Pestivirus A	KX170641	VIPR_ALG4_1129880548_	1a	NS5A	V092	2004	-0.8604	0.5057	1.1032
Pestivirus A	KX170642	VIPR_ALG4_1129880550_	1a	NS5A	V022	2006	-0.8246	0.5029	1.1002
Pestivirus A	KX170643	VIPR_ALG4_1129880552_	1a	NS5A	V054	2013	-0.8479	0.5031	1.1004
Pestivirus A	KX170644	VIPR_ALG4_1129880554_	1a	NS5A	V011	2001	-0.7395	0.5023	1.0993
Pestivirus A	KX170645	VIPR_ALG4_1129880556_	1a	NS5A	V012	2001	-0.7264	0.5000	1.0993
Pestivirus A	KX170646	VIPR_ALG4_1129880558_	1a	NS5A	V057	2009	-0.7509	0.4976	1.1045
Pestivirus A	KX170647	VIPR_ALG4_1129880560_	1a	NS5A	V006	2000	-0.9082	0.5049	1.1034
Pestivirus A	EF101530	VIPR_ALG4_118498779_8	1b	NS5A	KE9	2007	-0.9031	0.5220	1.1044
Pestivirus A	DQ088995	VIPR_ALG4_145309048_8	1a	NS5A	Singer_Arg	1974	-0.6830	0.5125	1.1073
Pestivirus A	U63479	VIPR_ALG4_1518836_844	1b	NS5A	CP7	1987	-0.8761	0.5495	1.1154
Pestivirus A	U86600	VIPR_ALG4_2149469_843	1b	NS5A	ILLNC	1991	-0.9426	0.4884	1.1173
Pestivirus A	AF041040	VIPR_ALG4_2789677_841	1a	NS5A	Oregon	1960	-0.8972	0.5247	1.1093
Pestivirus A	M96751	VIPR_ALG4_289508_8435	1a	NS5A	UNKNOWN-M96751	1992	-0.7949	0.5365	1.1066
Pestivirus A	HQ174292	VIPR_ALG4_323145267_8	1a	NS5A	180	2010	-1.1412	0.5249	1.1014

Species according to VIPRBRC	GenBank Accession	GenBank Protein Accession	Subgenotype	Protein	Strain Name	Collection Year	SVM Patho. Score	Vaxijen Antig. Score	Averged score of EMBOSS motifs
Pestivirus A	M31182	VIPR_ALG4_323206_870	1a	NS5A	UNKNOWN-M31182	1988	-0.7604	0.5000	1.1140
Pestivirus A	M96687	VIPR_ALG4_323230_866	1b	NS5A	Osloss	1967	-0.8028	0.5512	1.1181
Pestivirus A	JN400273	VIPR_ALG4_363990275_8	1q	NS5A	SD0803	2008	-0.8137	0.5110	1.1069
Pestivirus A	AF091605	VIPR_ALG4_3661566_84	1a	NS5A	Oregon C24V	1960	-0.9924	0.5312	1.1080
Pestivirus A	JN644055	VIPR_ALG4_373939303_8	1b	NS5A	3156	2011	-0.7069	0.5401	1.1005
Pestivirus A	JN380080	VIPR_ALG4_378753653_8	1a	NS5A	6010	2010	-1.1412	0.5249	1.1014
Pestivirus A	JX419397	VIPR_ALG4_404363562_8	1b	NS5A	UNKNOWN-JX419397	2008	-0.7179	0.5409	1.1147
Pestivirus A	JX419398	VIPR_ALG4_404363564_8	1b	NS5A	UNKNOWN-JX419398	2008	-0.7179	0.5409	1.1147
Pestivirus A	AF526381	VIPR_ALG4_42476348_8	1m	NS5A	ZM-95	1995	-0.5975	0.5016	1.1110
Pestivirus A	JX297512	VIPR_ALG4_459284067_8	1b	NS5A	10270	2007	-0.7025	0.5659	1.1163
Pestivirus A	JX297513	VIPR_ALG4_459284069_8	1b	NS5A	Aries	2005	-0.6650	0.5660	1.1126
Pestivirus A	JX297514	VIPR_ALG4_459284071_8	1b	NS5A	Columba	2005	-0.6924	0.5592	1.1197
Pestivirus A	JX297515	VIPR_ALG4_459284073_8	1b	NS5A	Corona	2005	-0.6924	0.5592	1.1197
Pestivirus A	JX297516	VIPR_ALG4_459284075_8	1b	NS5A	Gemini	2005	-0.7065	0.5691	1.1151
Pestivirus A	JX297517	VIPR_ALG4_459284077_8	1b	NS5A	Hercules	2006	-0.7272	0.5410	1.1185
Pestivirus A	JX297518	VIPR_ALG4_459284079_8	1b	NS5A	Leo	2006	-0.6924	0.5592	1.1197
Pestivirus A	JX297519	VIPR_ALG4_459284081_8	1b	NS5A	Lyra	2006	-0.7457	0.5519	1.1213
Pestivirus A	JX297520	VIPR_ALG4_459284083_8	1b	NS5A	Mars	2006	-0.6924	0.5592	1.1197
Pestivirus A	JX297521	VIPR_ALG4_459284085_8	1b	NS5A	Scorpius	2006	-0.7705	0.5533	1.1213
Pestivirus A	KC853440	VIPR_ALG4_507144146_8	1k	NS5A	SuwaNcp	1993	-0.3757	0.5547	1.1052
Pestivirus A	KC853441	VIPR_ALG4_507144148_8	1k	NS5A	SuwaCp	1993	-0.4211	0.5603	1.1052
Pestivirus A	KC695810	VIPR_ALG4_507866685_8	1q	NS5A	camel-6	2010	-0.9909	0.5043	1.1071
Pestivirus A	KC695814	VIPR_ALG4_507866704_8	1b	NS5A	Av69 VEDEVAC	2011	-1.0645	0.5471	1.1074
Pestivirus A	KC757383	VIPR_ALG4_511775165_8	1d	NS5A	10JJ-SKR	2010	-0.9782	0.4968	1.1091
Pestivirus A	KC963967	VIPR_ALG4_530291194_8	1b	NS5A	12F004	2012	-0.7914	0.5207	1.1130
Pestivirus A	KF772785	VIPR_ALG4_575471151_8	1b	NS5A	CC13B	2013	-0.7770	0.5590	1.1155
Pestivirus A	KF896608	VIPR_ALG4_586616532_8	1c	NS5A	Bega-like	2012	-0.7089	0.5277	1.1063
Pestivirus A	KF835697	VIPR_ALG4_597437474_8	1b	NS5A	AU526	2013	-0.8384	0.5336	1.1130
Pestivirus A	KJ541471	VIPR_ALG4_633265982_8	1a	NS5A	GS5	2013	-1.2254	0.5392	1.1140
Pestivirus A	KJ689448	VIPR_ALG4_635172915_8	1b	NS5A	GX4	2012	-1.0828	0.5469	1.1083
Pestivirus A	KF501393	VIPR_ALG4_669206614_8	1b	NS5A	BVDV JL-1	2009	-0.8405	0.5507	1.1109
Pestivirus A	AJ133738	VIPR_ALG4_7960754_870	1a	NS5A	type 1	1963	-0.7244	0.5015	1.1105
Pestivirus A	KP941581	VIPR_ALG4_800924313_8	1b	NS5A	USMARC-51998	2014	-0.8105	0.5473	1.1159
Pestivirus A	KP941583	VIPR_ALG4_800924317_8	1b	NS5A	USMARC-53874	2014	-0.6182	0.5348	1.1115
Pestivirus A	KP941584	VIPR_ALG4_800924319_8	1a	NS5A	USMARC-53875	2014	-0.9334	0.5387	1.1083
Pestivirus A	KP941586	VIPR_ALG4_800924323_8	1a	NS5A	USMARC-55477	2014	-0.8341	0.5015	1.1078
Pestivirus A	KP941587	VIPR_ALG4_800924325_8	1b	NS5A	USMARC-55478	2014	-0.7975	0.5562	1.1137
Pestivirus A	KP941588	VIPR_ALG4_800924327_8	1b	NS5A	USMARC-55922	2014	-0.9034	0.5546	1.1132
Pestivirus A	KP941589	VIPR_ALG4_800924329_8	1b	NS5A	USMARC-55923	2014	-0.6077	0.5474	1.1123
Pestivirus A	KP941590	VIPR_ALG4_800924331_8	1b	NS5A	USMARC-55924	2014	-0.6275	0.5355	1.1124
Pestivirus A	KP941591	VIPR_ALG4_800924333_8	1b	NS5A	USMARC-55925	2014	-1.0788	0.5741	1.1138
Pestivirus A	KP941592	VIPR_ALG4_800924335_8	1b	NS5A	USMARC-55926	2014	-1.0842	0.5380	1.1176
Pestivirus A	KP313732	VIPR_ALG4_816850387_8	1e	NS5A	Carlito	2014	-0.9697	0.5771	1.1009
Pestivirus A	KR029825	VIPR_ALG4_887497286_8	1b	NS5A	Egy/Ismailia/2014	2014	-0.8028	0.5296	1.1112
Pestivirus A	LC089875	VIPR_ALG4_939106262_8	1o	NS5A	IS26/01ncp	2001	-0.4281	0.5191	1.1073
Pestivirus A	LC089876	VIPR_ALG4_939106264_8	1n	NS5A	Shitara/02/06	2006	-0.7890	0.5088	1.1016
Pestivirus A	KR866116	VIPR_ALG4_941508008_8	1m	NS5A	SD-15	2015	-0.5815	0.5209	1.1066

Species according to VIPRBRC	GenBank Accession	GenBank Protein Accession	Subgenotype	Protein	Strain Name	Collection Year	SVM Patho. Score	Vaxijen Antig. Score	Averged score of EMBOSS motifs
Pestivirus A	KU200260	VIPR_ALG4_972905813_8	1b	NS5A	BE/061536/2014	2014	-1.0797	0.5298	1.1089
Pestivirus A	KX577637	VIPR_ALG4_AOR50934_1	1e	NS5A	SLO/2407/2006	2006	-0.8110	0.5551	1.1102
Pestivirus A	KX987157	VIPR_ALG4_APG30987_1	1f	NS5A	SLO/1170/2000	2000	-0.8305	0.5695	1.1037
Pestivirus A	KX857724	VIPR_ALG4_APZ85839_1	1i	NS5A	ACM/BR/2016	2016	-0.6936	0.5165	1.1075
Pestivirus A	KY849592	VIPR_ALG4_ART90617_1	1d	NS5A	SLO/2416/2002	2002	-1.0285	0.4549	1.1022
Pestivirus A	MF278651	VIPR_ALG4_ASW18434_1	1b	NS5A	XZ01	2016	-0.9939	0.5596	1.1059
Pestivirus A	MF278652	VIPR_ALG4_ASW18435_1	1b	NS5A	XZ02	2016	-1.0236	0.5491	1.1057
Pestivirus A	MF693403	VIPR_ALG4_ATG71375_1	1a	NS5A	UNKNOWN-MF693403	2016	-0.9167	0.5329	1.1093
Pestivirus A	KY964311	VIPR_ALG4_ATN39078_1	1b	NS5A	Y2	2014	-0.6433	0.5503	1.1125
Pestivirus A	MF172980	VIPR_ALG4_AVI10261_1	1c	NS5A	GSTZ	2012	-0.6658	0.5732	1.1146
Pestivirus A	MH379638	VIPR_ALG4_AWW14171_	1a	NS5A	Ho916	1993	-0.6098	0.5238	1.1107
Pestivirus A	MG950344	VIPR_ALG4_AWW87346_	1b	NS5A	AU526	2014	-0.8384	0.5336	1.1130
Pestivirus A	MG950345	VIPR_ALG4_AWW87347_	1b	NS5A	B1	2015	-0.8384	0.5336	1.1130
Pestivirus A	MG950346	VIPR_ALG4_AWW87348_	1b	NS5A	B2	2015	-0.8384	0.5336	1.1130
Pestivirus A	MG950347	VIPR_ALG4_AWW87349_	1b	NS5A	B3	2015	-0.8384	0.5336	1.1130
Pestivirus A	MG950348	VIPR_ALG4_AWW87350_	1b	NS5A	B4	2015	-0.8384	0.5336	1.1130
Pestivirus A	MG950349	VIPR_ALG4_AWW87351_	1b	NS5A	B5	2015	-0.8384	0.5336	1.1130
Pestivirus A	MG950350	VIPR_ALG4_AWW87352_	1b	NS5A	B6	2015	-0.8384	0.5336	1.1130
Pestivirus A	MG950351	VIPR_ALG4_AWW87353_	1b	NS5A	O1	2015	-0.8384	0.5336	1.1130
Pestivirus A	MG950352	VIPR_ALG4_AWW87354_	1b	NS5A	O2	2015	-0.8272	0.5326	1.1130
Pestivirus A	MG950353	VIPR_ALG4_AWW87355_	1b	NS5A	O3	2015	-0.8384	0.5336	1.1130
Pestivirus A	MG950354	VIPR_ALG4_AWW87356_	1b	NS5A	O4	2015	-0.8384	0.5336	1.1130
Pestivirus A	MG950355	VIPR_ALG4_AWW87357_	1b	NS5A	O5	2015	-0.8384	0.5336	1.1130
Pestivirus A	MG950356	VIPR_ALG4_AWW87358_	1b	NS5A	O6	2015	-0.8384	0.5336	1.1130
Pestivirus A	MG950357	VIPR_ALG4_AWW87359_	1b	NS5A	B1A	2015	-0.8384	0.5336	1.1130
Pestivirus A	MG950358	VIPR_ALG4_AWW87360_	1b	NS5A	B2A	2016	-0.8384	0.5336	1.1130
Pestivirus A	MG950359	VIPR_ALG4_AWW87361_	1b	NS5A	B3A	2016	-0.8384	0.5336	1.1130
Pestivirus A	MG950360	VIPR_ALG4_AWW87362_	1b	NS5A	B4A	2016	-0.8384	0.5336	1.1130
Pestivirus A	MG950361	VIPR_ALG4_AWW87363_	1b	NS5A	B5A	2016	-0.8384	0.5336	1.1130
Pestivirus A	MG950362	VIPR_ALG4_AWW87364_	1b	NS5A	B6A	2016	-0.8482	0.5351	1.1126
Pestivirus A	MG950363	VIPR_ALG4_AWW87365_	1b	NS5A	O1A	2015	-0.8384	0.5336	1.1130
Pestivirus A	MG950364	VIPR_ALG4_AWW87366_	1b	NS5A	O2A	2015	-0.8384	0.5336	1.1130
Pestivirus A	MG950365	VIPR_ALG4_AWW87367_	1b	NS5A	O2B	2015	-0.8384	0.5336	1.1130
Pestivirus A	MG950366	VIPR_ALG4_AWW87368_	1b	NS5A	O4A	2015	-0.8384	0.5336	1.1130
Pestivirus A	MH311874	VIPR_ALG4_AWW87369_	1b	NS5A	B2A d168	2016	-0.8384	0.5336	1.1130
Pestivirus A	MH311875	VIPR_ALG4_AWW87370_	1b	NS5A	B3A d168	2016	-0.8384	0.5336	1.1130
Pestivirus A	MH311876	VIPR_ALG4_AWW87371_	1b	NS5A	B4A d84	2016	-0.8384	0.5336	1.1130
Pestivirus A	MH311877	VIPR_ALG4_AWW87372_	1b	NS5A	B4A d168	2016	-0.8384	0.5336	1.1130
Pestivirus A	MH311878	VIPR_ALG4_AWW87373_	1b	NS5A	B5A d84	2016	-0.8384	0.5336	1.1130
Pestivirus A	MH311879	VIPR_ALG4_AWW87374_	1b	NS5A	B5A d168	2016	-0.8384	0.5336	1.1130
Pestivirus A	MH311880	VIPR_ALG4_AWW87375_	1b	NS5A	B6A d84	2016	-0.8482	0.5351	1.1126
Pestivirus A	MH311881	VIPR_ALG4_AWW87376_	1b	NS5A	B6A d168	2016	-0.8482	0.5351	1.1126
Pestivirus A	MH379221	VIPR_ALG4_AWW87377_	1b	NS5A	P1	2017	-0.8856	0.5341	1.1138
Pestivirus A	MH379222	VIPR_ALG4_AWW87378_	1b	NS5A	P2	2017	-0.8856	0.5341	1.1138
Pestivirus A	MH379223	VIPR_ALG4_AWW87379_	1b	NS5A	P5	2017	-0.8856	0.5341	1.1138
Pestivirus A	MH379224	VIPR_ALG4_AWW87380_	1b	NS5A	P6	2017	-0.8856	0.5341	1.1138
Pestivirus A	MH379225	VIPR_ALG4_AWW87381_	1b	NS5A	P7	2017	-0.8856	0.5341	1.1138

Species according to VIPRBRC	GenBank Accession	GenBank Protein Accession	Subgenotype	Protein	Strain Name	Collection Year	SVM Patho. Score	Vaxijen Antig. Score	Averged score of EMBOSS motifs
Pestivirus A	MH379226	VIPR_ALG4_AWW87382_	1b	NS5A	P5A	2017	-0.8856	0.5341	1.1138
Pestivirus A	MH379227	VIPR_ALG4_AWW87383_	1b	NS5A	P5B	2017	-0.8856	0.5341	1.1138
Pestivirus A	MH379228	VIPR_ALG4_AWW87384_	1b	NS5A	P5C	2017	-0.8856	0.5341	1.1138
Pestivirus A	MH379229	VIPR_ALG4_AWW87385_	1b	NS5A	P5D	2017	-0.8856	0.5341	1.1138
Pestivirus A	MH379230	VIPR_ALG4_AWW87386_	1b	NS5A	P5F	2017	-0.8856	0.5341	1.1138
Pestivirus A	MH379231	VIPR_ALG4_AWW87387_	1b	NS5A	P7A	2018	-0.8856	0.5341	1.1138
Pestivirus A	MH379232	VIPR_ALG4_AWW87388_	1b	NS5A	P7C	2018	-0.8856	0.5341	1.1138
Pestivirus A	MH379233	VIPR_ALG4_AWW87389_	1b	NS5A	P7E	2018	-0.8856	0.5341	1.1138
Pestivirus A	MH379234	VIPR_ALG4_AWW87390_	1b	NS5A	P7F	2018	-0.8856	0.5341	1.1138
Pestivirus A	MH166806	VIPR_ALG4_AYA62524_1	1m	NS5A	XC	2015	-0.6504	0.5179	1.1056
Pestivirus A	MH490943	VIPR_ALG4_AZB53078_1	1b	NS5A	BVDV BJ-2016	2016	-0.9251	0.5220	1.1175
Pestivirus A	MH231153	VIPR_ALG4_AZQ00677_1	1b	NS5A	Nebraska	1990	-0.9332	0.5328	1.1158
Pestivirus A	AB078950	VIPR_ALG4_BAC55961_1	1j	NS5A	KS86-1ncp	1986	-1.1229	0.5707	1.1074
Pestivirus A	MH899941	VIPR_ALG4_QCE30388_1	1b	NS5A	SLO/3301/2014	2014	-0.8761	0.5536	1.1120
Pestivirus A	MH899942	VIPR_ALG4_QCE30389_1	1e	NS5A	SLO/33529/2015	2015	-0.9275	0.5506	1.1125
Pestivirus A	MH899943	VIPR_ALG4_QCE30390_1	1f	NS5A	SLO/1361/2014	2014	-0.3464	0.5869	1.1094
Pestivirus A	MH899944	VIPR_ALG4_QCE30391_1	1f	NS5A	SLO/28537/2017	2017	-0.6997	0.5621	1.1053
Pestivirus A	MH899945	VIPR_ALG4_QCE30392_1	1h	NS5A	SLO/1883/2013	2013	-0.6989	0.5187	1.1011
Pestivirus A	MK102095	VIPR_ALG4_QCQ84262_1	1q	NS5A	20170226	2017	-0.7481	0.5498	1.1015
Pestivirus A	MK509774	VIPR_ALG4_QEK23510_1	1b	NS5A	BVD1b-JH	2008	-0.8049	0.5502	1.1130
Pestivirus A	MK775204	VIPR_ALG4_QFX66041_1	1i	NS5A	CA2006	2006	-0.7298	0.5119	1.1060
Pestivirus A	MN188073	VIPR_ALG4_QGZ19414_1	1a	NS5A	PI34	2017	-0.7509	0.4932	1.1032
Pestivirus A	MN188074	VIPR_ALG4_QGZ19415_1	1b	NS5A	PI285	2017	-0.7248	0.5234	1.1131
Pestivirus A	MT079816	VIPR_ALG4_QIM55913_1	1c	NS5A	GXNN1	2018	-0.4247	0.5639	1.1158
Pestivirus A	MN623291	VIPR_ALG4_QLL27013_1	1m	NS5A	NX2019/01	2019	-0.5896	0.5010	1.1083
Pestivirus A	MW014286	VIPR_ALG4_QPJ59878_1	1b	NS5A	GXSS01	2018	-1.0214	0.5457	1.1083
Pestivirus A	MW014287	VIPR_ALG4_QPJ59879_1	1b	NS5A	GXSS02	2018	-0.9840	0.5588	1.1095
Pestivirus A	MW014288	VIPR_ALG4_QPJ59880_1	1b	NS5A	GXSS03	2018	-0.9673	0.5601	1.1095
Pestivirus A	MT977117	VIPR_ALG4_QRZ20359_1	1b	NS5A	BVDV 1b IT16/5	2016	-1.0101	0.5498	1.1069
Pestivirus A	MT977118	VIPR_ALG4_QRZ20360_1	1b	NS5A	BVDV 1b IT16/439	2016	-1.0101	0.5498	1.1069
Pestivirus A	MT654137	VIPR_ALG4_QVK82311_1	1a	NS5A	20-8536	2020	-0.9217	0.5114	1.1064
Pestivirus A	LT837585	VIPR_ALG4_SLV80196_1	1r	NS5A	UNKNOWN-LT837585	2012	-0.3786	0.5679	1.1091
Pestivirus A	MW054933	VIPR_ALG4_UEC94252_1	1f	NS5A	LA/230/14	2014	-0.7630	0.5374	1.1035
Pestivirus A	MW054934	VIPR_ALG4_UEC94253_1	1f	NS5A	LA/87/05	2005	-0.8982	0.5773	1.1058
Pestivirus A	MW054935	VIPR_ALG4_UEC94254_1	1k	NS5A	TO/197/11	2011	-0.4140	0.5623	1.1054
Pestivirus A	MW054936	VIPR_ALG4_UEC94255_1	1g	NS5A	UM/111/06	2006	-0.6864	0.5355	1.1081
Pestivirus A	MW054937	VIPR_ALG4_UEC94256_1	1k	NS5A	SA/158/09	2009	-0.5112	0.5576	1.1159
Pestivirus A	MW054938	VIPR_ALG4_UEC94257_1	1k	NS5A	SA/159/09	2009	-0.5112	0.5576	1.1159
Pestivirus A	MW054939	VIPR_ALG4_UEC94258_1	1f	NS5A	LO/151/09	2009	-0.6700	0.5493	1.1010
Pestivirus A	MW054940	VIPR_ALG4_UEC94259_1	1e	NS5A	MA/101/05	2005	-0.9041	0.5542	1.1090
Pestivirus A	MW250796	VIPR_ALG4_UEC94260_1	1i	NS5A	58-1	2008	-0.7962	0.5363	1.1075
Pestivirus A	MW250797	VIPR_ALG4_UEC94261_1	1i	NS5A	58-2	2008	-0.7962	0.5363	1.1075
Pestivirus A	MW250798	VIPR_ALG4_UEC94262_1	1a	NS5A	62-2	2008	-0.8144	0.5136	1.1054
Pestivirus A	MW250799	VIPR_ALG4_UEC94263_1	1a	NS5A	63-1	2008	-0.8392	0.5301	1.1089
Pestivirus A	MW250800	VIPR_ALG4_UEC94264_1	1d	NS5A	67-1	2008	-0.9278	0.4736	1.1048
Pestivirus A	MW250801	VIPR_ALG4_UEC94265_1	1d	NS5A	67-2	2008	-0.9278	0.4736	1.1048
Pestivirus A	MW250802	VIPR_ALG4_UEC94266_1	1e	NS5A	68-1	2008	-0.7495	0.5483	1.1113

Species according to VIPRBRC	GenBank Accession	GenBank Protein Accession	Subgenotype	Protein	Strain Name	Collection Year	SVM Patho. Score	Vaxijen Antig. Score	Averged score of EMBOSS motifs
Pestivirus A	MW250803	VIPR_ALG4_UEC94267_1	1i	NS5A	69-1	2008	-0.7597	0.5274	1.1070
Pestivirus A	MW655625	VIPR_ALG4_UEC94268_1	1h	NS5A	CH-04-01b	2004	-0.7653	0.5154	1.1022
Pestivirus A	MW655626	VIPR_ALG4_UEC94269_1	1e	NS5A	Maria	2004	-1.0453	0.5702	1.1134
Pestivirus A	MW655627	VIPR_ALG4_UEC94270_1	1e	NS5A	R2000-95	1995	-0.9506	0.5967	1.1120
Pestivirus A	MW655628	VIPR_ALG4_UEC94271_1	1k	NS5A	R3230-95	1995	-0.3230	0.5650	1.1045
Pestivirus A	MW655629	VIPR_ALG4_UEC94272_1	1h	NS5A	R3572-90	1990	-0.6527	0.5291	1.1002
Pestivirus A	MW655630	VIPR_ALG4_UEC94273_1	1k	NS5A	R5013-96	1996	-0.4364	0.5649	1.1077
Pestivirus A	MW655631	VIPR_ALG4_UEC94274_1	1e	NS5A	S03-1175	2003	-0.7653	0.5327	1.1055
Pestivirus A	MW655632	VIPR_ALG4_UEC94275_1	1h	NS5A	SM09-20	2002	-0.6657	0.5450	1.1008
Pestivirus A	MW713361	VIPR_ALG4_UEC94276_1	1a	NS5A	BoAEC1190	2007	-0.7614	0.5049	1.1108
Pestivirus A	MW713362	VIPR_ALG4_UEC94277_1	1b	NS5A	PI819	2017	-0.8291	0.5311	1.1130
Pestivirus A	MW732738	VIPR_ALG4_UEC94278_1	1a	NS5A	PI407	2015	-1.0499	0.5088	1.1096
Pestivirus A	MW732739	VIPR_ALG4_UEC94279_1	1a	NS5A	YandaSpl	1993	-1.0350	0.5185	1.1042
Pestivirus A	MZ188972	VIPR_ALG4_UML14262_1	1q	NS5A	HB-1	2020	-0.5149	0.5020	1.1098
Pestivirus A	ON337882	VIPR_ALG4_USZ80113_1	1c	NS5A	NM2103	2021	-0.7711	0.5569	1.1072
Pestivirus A	KU159365	VIPR_ALG4_1039262063	1a	NS5B	USII-S15	2015	-0.2846	0.5148	1.1159
Pestivirus A	KU756226	VIPR_ALG4_1072900294	1b	NS5B	HJ-1	2010	-0.2845	0.5127	1.1155
Pestivirus A	KT943518	VIPR_ALG4_1093530908	1d	NS5B	BJ1201	2012	-0.2716	0.5175	1.1181
Pestivirus A	LT631725	VIPR_ALG4_1112914034	1h	NS5B	UM/126/07	2007	-0.4709	0.5006	1.1129
Pestivirus A	KX170656	VIPR_ALG4_1129880578	1b	NS5B	V015	2001	-0.2089	0.5159	1.1143
Pestivirus A	KX170657	VIPR_ALG4_1129880580	1b	NS5B	V060	2004	-0.3009	0.5078	1.1137
Pestivirus A	KX170658	VIPR_ALG4_1129880582	1b	NS5B	V036	2007	-0.3375	0.5156	1.1173
Pestivirus A	KX170659	VIPR_ALG4_1129880584	1b	NS5B	V020	2005	-0.3149	0.5153	1.1131
Pestivirus A	KX170660	VIPR_ALG4_1129880586	1b	NS5B	V029	2006	-0.2432	0.5173	1.1151
Pestivirus A	KX170661	VIPR_ALG4_1129880588	1b	NS5B	V045	2009	-0.3118	0.5006	1.1101
Pestivirus A	KX170662	VIPR_ALG4_1129880590	1b	NS5B	V078	2012	-0.3501	0.5127	1.1125
Pestivirus A	KX170663	VIPR_ALG4_1129880592	1b	NS5B	V031	2006	-0.3397	0.5145	1.1118
Pestivirus A	KX170664	VIPR_ALG4_1129880594	1b	NS5B	V087	2006	-0.3314	0.5163	1.1128
Pestivirus A	KX170665	VIPR_ALG4_1129880596	1b	NS5B	V070	2007	-0.2847	0.5113	1.1128
Pestivirus A	KX170666	VIPR_ALG4_1129880598	1b	NS5B	V098	1999	-0.4162	0.5177	1.1155
Pestivirus A	KX170667	VIPR_ALG4_1129880600	1b	NS5B	V075	2011	-0.2868	0.5239	1.1137
Pestivirus A	KX170668	VIPR_ALG4_1129880602	1b	NS5B	V100	1997	-0.2464	0.5282	1.1110
Pestivirus A	KX170669	VIPR_ALG4_1129880604	1a	NS5B	V092	2004	-0.3207	0.5122	1.1140
Pestivirus A	KX170670	VIPR_ALG4_1129880606	1a	NS5B	V022	2006	-0.2063	0.5094	1.1160
Pestivirus A	KX170671	VIPR_ALG4_1129880608	1a	NS5B	V054	2013	-0.3560	0.5160	1.1174
Pestivirus A	KX170672	VIPR_ALG4_1129880610	1a	NS5B	V012	2001	-0.3652	0.5089	1.1134
Pestivirus A	KX170673	VIPR_ALG4_1129880612	1a	NS5B	V006	2000	-0.3471	0.5125	1.1146
Pestivirus A	KX170674	VIPR_ALG4_1129880614	1a	NS5B	V057	2009	-0.3583	0.5134	1.1151
Pestivirus A	KX170675	VIPR_ALG4_1129880616	1a	NS5B	V083	2008	-0.4705	0.5164	1.1118
Pestivirus A	KX170676	VIPR_ALG4_1129880618	1a	NS5B	V059	2004	-0.4275	0.5346	1.1169
Pestivirus A	KX170677	VIPR_ALG4_1129880620	1a	NS5B	V099	1998	-0.5184	0.5309	1.1126
Pestivirus A	KX170678	VIPR_ALG4_1129880622	1a	NS5B	V026	2006	-0.3997	0.5283	1.1105
Pestivirus A	KX170679	VIPR_ALG4_1129880624	1a	NS5B	V027	2006	-0.3997	0.5283	1.1105
Pestivirus A	KX170680	VIPR_ALG4_1129880626	1a	NS5B	V091	2003	-0.4828	0.5300	1.1090
Pestivirus A	KX170681	VIPR_ALG4_1129880628	1a	NS5B	V067	2006	-0.4828	0.5300	1.1090
Pestivirus A	KX170682	VIPR_ALG4_1129880630	1a	NS5B	V007	2000	-0.4828	0.5300	1.1090
Pestivirus A	KX170683	VIPR_ALG4_1129880632	1a	NS5B	V013	2001	-0.4828	0.5300	1.1090

Species according to VIPRBRC	GenBank Accession	GenBank Protein Accession	Subgenotype	Protein	Strain Name	Collection Year	SVM Patho. Score	Vaxijen Antig. Score	Averged score of EMBOSS motifs
Pestivirus A	KX170684	VIPR_ALG4_1129880634_	1a	NS5B	V033	2007	-0.4828	0.5300	1.1090
Pestivirus A	KX170685	VIPR_ALG4_1129880636_	1a	NS5B	V034	2007	-0.4828	0.5300	1.1090
Pestivirus A	KX170686	VIPR_ALG4_1129880638_	1a	NS5B	V074	2010	-0.4828	0.5300	1.1090
Pestivirus A	KX170687	VIPR_ALG4_1129880640_	1a	NS5B	V077	2012	-0.4828	0.5300	1.1090
Pestivirus A	KX170688	VIPR_ALG4_1129880642_	1a	NS5B	V073	2011	-0.4828	0.5300	1.1090
Pestivirus A	KX170689	VIPR_ALG4_1129880644_	1a	NS5B	V049	2009	-0.3036	0.5462	1.1127
Pestivirus A	KX170690	VIPR_ALG4_1129880646_	1a	NS5B	V016	2002	-0.3575	0.5168	1.1128
Pestivirus A	KX170691	VIPR_ALG4_1129880648_	1a	NS5B	V001	1999	-0.4024	0.5520	1.1170
Pestivirus A	KX170692	VIPR_ALG4_1129880650_	1a	NS5B	V035	2007	-0.3541	0.5480	1.1130
Pestivirus A	KX170693	VIPR_ALG4_1129880652_	1a	NS5B	V050	2009	-0.3495	0.5477	1.1137
Pestivirus A	KX170694	VIPR_ALG4_1129880654_	1a	NS5B	V040	2008	-0.3714	0.5499	1.1160
Pestivirus A	KX170695	VIPR_ALG4_1129880656_	1a	NS5B	V039	2008	-0.3495	0.5477	1.1137
Pestivirus A	KX170696	VIPR_ALG4_1129880658_	1a	NS5B	V041	2008	-0.3495	0.5477	1.1137
Pestivirus A	KX170697	VIPR_ALG4_1129880660_	1a	NS5B	V043	2008	-0.3603	0.5456	1.1137
Pestivirus A	KX170698	VIPR_ALG4_1129880662_	1a	NS5B	V052	2010	-0.3479	0.5482	1.1137
Pestivirus A	KX170699	VIPR_ALG4_1129880664_	1a	NS5B	V042	2008	-0.3495	0.5477	1.1137
Pestivirus A	KX170700	VIPR_ALG4_1129880666_	1a	NS5B	V046	2009	-0.3495	0.5477	1.1137
Pestivirus A	KX170701	VIPR_ALG4_1129880668_	1a	NS5B	V010	2001	-0.4012	0.5371	1.1150
Pestivirus A	KX170702	VIPR_ALG4_1129880670_	1a	NS5B	V008	2000	-0.3915	0.5374	1.1134
Pestivirus A	KX170703	VIPR_ALG4_1129880672_	1a	NS5B	V009	2000	-0.3915	0.5374	1.1134
Pestivirus A	EF101530	VIPR_ALG4_118498779_9	1b	NS5B	KE9	2007	-0.3566	0.5137	1.1111
Pestivirus A	DQ088995	VIPR_ALG4_145309048_9	1a	NS5B	Singer_Arg	1974	-0.4611	0.5307	1.1090
Pestivirus A	U63479	VIPR_ALG4_1518836_99	1b	NS5B	CP7	1987	-0.2523	0.5068	1.1118
Pestivirus A	U86600	VIPR_ALG4_2149469_99	1b	NS5B	ILLNC	1991	-0.3271	0.4836	1.1123
Pestivirus A	AF041040	VIPR_ALG4_2789677_99	1a	NS5B	Oregon	1960	-0.4376	0.5319	1.1195
Pestivirus A	M96751	VIPR_ALG4_289508_992	1a	NS5B	UNKNOWN-M96751	1992	-0.3757	0.5380	1.1137
Pestivirus A	HQ174292	VIPR_ALG4_323145267_9	1a	NS5B	180	2010	-0.5280	0.5440	1.1136
Pestivirus A	M31182	VIPR_ALG4_323206_101	1a	NS5B	UNKNOWN-M31182	1988	-0.3997	0.5318	1.1112
Pestivirus A	M96687	VIPR_ALG4_323230_101	1b	NS5B	Osloss	1967	-0.3530	0.5224	1.1080
Pestivirus A	JN400273	VIPR_ALG4_363990275_9	1q	NS5B	SD0803	2008	-0.4102	0.5014	1.1239
Pestivirus A	AF091605	VIPR_ALG4_3661566_99	1a	NS5B	Oregon C24V	1960	-0.4275	0.5346	1.1169
Pestivirus A	JN644055	VIPR_ALG4_373939303_9	1b	NS5B	3156	2011	-0.3503	0.5396	1.1131
Pestivirus A	JN380080	VIPR_ALG4_378753653_9	1a	NS5B	6010	2010	-0.5050	0.5394	1.1152
Pestivirus A	JX419397	VIPR_ALG4_404363562_9	1b	NS5B	UNKNOWN-JX419397	2008	-0.3539	0.5234	1.1132
Pestivirus A	JX419398	VIPR_ALG4_404363564_9	1b	NS5B	UNKNOWN-JX419398	2008	-0.3643	0.5259	1.1131
Pestivirus A	AF526381	VIPR_ALG4_42476348_98	1m	NS5B	ZM-95	1995	-0.2797	0.4616	1.1165
Pestivirus A	JX297512	VIPR_ALG4_459284067_9	1b	NS5B	10270	2007	-0.2361	0.4903	1.1125
Pestivirus A	JX297513	VIPR_ALG4_459284069_9	1b	NS5B	Aries	2005	-0.2918	0.4903	1.1125
Pestivirus A	JX297514	VIPR_ALG4_459284071_9	1b	NS5B	Columba	2005	-0.2808	0.4897	1.1125
Pestivirus A	JX297515	VIPR_ALG4_459284073_9	1b	NS5B	Corona	2005	-0.2808	0.4897	1.1125
Pestivirus A	JX297516	VIPR_ALG4_459284075_9	1b	NS5B	Gemini	2005	-0.2849	0.4925	1.1125
Pestivirus A	JX297517	VIPR_ALG4_459284077_9	1b	NS5B	Hercules	2006	-0.3304	0.5123	1.1136
Pestivirus A	JX297518	VIPR_ALG4_459284079_9	1b	NS5B	Leo	2006	-0.2808	0.4897	1.1125
Pestivirus A	JX297519	VIPR_ALG4_459284081_9	1b	NS5B	Lyra	2006	-0.2808	0.4897	1.1125
Pestivirus A	JX297520	VIPR_ALG4_459284083_9	1b	NS5B	Mars	2006	-0.2808	0.4897	1.1125
Pestivirus A	JX297521	VIPR_ALG4_459284085_9	1b	NS5B	Scorpius	2006	-0.2853	0.4829	1.1093
Pestivirus A	KC853440	VIPR_ALG4_507144146_9	1k	NS5B	SuwaNcp	1993	-0.2902	0.5111	1.1151

Species according to VIPRBRC	GenBank Accession	GenBank Protein Accession	Subgenotype	Protein	Strain Name	Collection Year	SVM Patho. Score	Vaxijen Antig. Score	Averged score of EMBOSS motifs
Pestivirus A	KC853441	VIPR_ALG4_507144148_1	1k	NS5B	SuwaCp	1993	-0.2902	0.5111	1.1151
Pestivirus A	KC695810	VIPR_ALG4_507866685_9	1q	NS5B	camel-6	2010	-0.2701	0.4754	1.1156
Pestivirus A	KC695814	VIPR_ALG4_507866704_9	1b	NS5B	Av69 VEDEVAC	2011	-0.3512	0.5114	1.1109
Pestivirus A	KC757383	VIPR_ALG4_511775165_9	1d	NS5B	10JJ-SKR	2010	-0.2934	0.5419	1.1171
Pestivirus A	KC963967	VIPR_ALG4_530291194_9	1b	NS5B	12F004	2012	-0.1685	0.5022	1.1136
Pestivirus A	KF772785	VIPR_ALG4_575471151_9	1b	NS5B	CC13B	2013	-0.3633	0.5127	1.1118
Pestivirus A	KF896608	VIPR_ALG4_586616532_9	1c	NS5B	Bega-like	2012	-0.3450	0.5327	1.1118
Pestivirus A	KF835697	VIPR_ALG4_597437474_9	1b	NS5B	AU526	2013	-0.3086	0.5122	1.1137
Pestivirus A	KJ541471	VIPR_ALG4_633265982_9	1a	NS5B	GS5	2013	-0.4997	0.5029	1.1185
Pestivirus A	KJ689448	VIPR_ALG4_635172915_9	1b	NS5B	GX4	2012	-0.3944	0.5117	1.1131
Pestivirus A	KF501393	VIPR_ALG4_669206614_9	1b	NS5B	BVDV JL-1	2009	-0.1998	0.5114	1.1170
Pestivirus A	AJ133738	VIPR_ALG4_7960754_10	1a	NS5B	type 1	1963	-0.4070	0.5300	1.1131
Pestivirus A	KP941581	VIPR_ALG4_800924313_9	1b	NS5B	USMARC-51998	2014	-0.3045	0.5098	1.1162
Pestivirus A	KP941583	VIPR_ALG4_800924317_9	1b	NS5B	USMARC-53874	2014	-0.3681	0.5071	1.1131
Pestivirus A	KP941584	VIPR_ALG4_800924319_9	1a	NS5B	USMARC-53875	2014	-0.4460	0.5373	1.1182
Pestivirus A	KP941586	VIPR_ALG4_800924323_9	1a	NS5B	USMARC-55477	2014	-0.4496	0.5199	1.1114
Pestivirus A	KP941587	VIPR_ALG4_800924325_9	1b	NS5B	USMARC-55478	2014	-0.2873	0.5076	1.1142
Pestivirus A	KP941588	VIPR_ALG4_800924327_9	1b	NS5B	USMARC-55922	2014	-0.3186	0.5028	1.1143
Pestivirus A	KP941589	VIPR_ALG4_800924329_9	1b	NS5B	USMARC-55923	2014	-0.3670	0.5205	1.1143
Pestivirus A	KP941590	VIPR_ALG4_800924331_9	1b	NS5B	USMARC-55924	2014	-0.2617	0.5090	1.1131
Pestivirus A	KP941591	VIPR_ALG4_800924333_9	1b	NS5B	USMARC-55925	2014	-0.1954	0.5228	1.1118
Pestivirus A	KP941592	VIPR_ALG4_800924335_9	1b	NS5B	USMARC-55926	2014	-0.2192	0.5116	1.1167
Pestivirus A	KP313732	VIPR_ALG4_816850387_9	1e	NS5B	Carlito	2014	-0.3264	0.4881	1.1057
Pestivirus A	KR029825	VIPR_ALG4_887497286_9	1b	NS5B	Egy/Ismailia/2014	2014	-0.2514	0.5147	1.1125
Pestivirus A	LC089875	VIPR_ALG4_939106262_9	1o	NS5B	IS26/01ncp	2001	-0.4461	0.4822	1.1163
Pestivirus A	LC089876	VIPR_ALG4_939106264_9	1n	NS5B	Shitara/02/06	2006	-0.1770	0.4971	1.1170
Pestivirus A	KR866116	VIPR_ALG4_941508008_9	1m	NS5B	SD-15	2015	-0.2313	0.4779	1.1162
Pestivirus A	KU200260	VIPR_ALG4_972905813_9	1b	NS5B	BE/061536/2014	2014	-0.3563	0.5158	1.1076
Pestivirus A	KX577637	VIPR_ALG4_AOR50934_1	1e	NS5B	SLO/2407/2006	2006	-0.1669	0.5168	1.1073
Pestivirus A	KX987157	VIPR_ALG4_APG30987_1	1f	NS5B	SLO/1170/2000	2000	-0.3103	0.4938	1.1080
Pestivirus A	KX857724	VIPR_ALG4_APZ85839_1	1i	NS5B	ACM/BR/2016	2016	-0.4539	0.5192	1.1145
Pestivirus A	KY849592	VIPR_ALG4_ART90617_1	1d	NS5B	SLO/2416/2002	2002	-0.3962	0.5226	1.1085
Pestivirus A	MF278651	VIPR_ALG4_ASW18434_1	1b	NS5B	XZ01	2016	-0.3512	0.5114	1.1109
Pestivirus A	MF278652	VIPR_ALG4_ASW18435_1	1b	NS5B	XZ02	2016	-0.3422	0.5140	1.1109
Pestivirus A	MF693403	VIPR_ALG4_ATG71375_1	1a	NS5B	UNKNOWN-MF693403	2016	-0.4316	0.5730	1.1079
Pestivirus A	KY964311	VIPR_ALG4_ATN39078_1	1b	NS5B	Y2	2014	-0.3045	0.5195	1.1162
Pestivirus A	MF172980	VIPR_ALG4_AVI10261_1_	1c	NS5B	GSTZ	2012	-0.2878	0.5309	1.1089
Pestivirus A	MH379638	VIPR_ALG4_AWW14171_	1a	NS5B	Ho916	1993	-0.4812	0.5204	1.1182
Pestivirus A	MG950344	VIPR_ALG4_AWW87346_	1b	NS5B	AU526	2014	-0.3086	0.5122	1.1137
Pestivirus A	MG950345	VIPR_ALG4_AWW87347_	1b	NS5B	B1	2015	-0.3086	0.5122	1.1137
Pestivirus A	MG950346	VIPR_ALG4_AWW87348_	1b	NS5B	B2	2015	-0.2743	0.5128	1.1137
Pestivirus A	MG950347	VIPR_ALG4_AWW87349_	1b	NS5B	B3	2015	-0.3086	0.5122	1.1137
Pestivirus A	MG950348	VIPR_ALG4_AWW87350_	1b	NS5B	B4	2015	-0.3086	0.5122	1.1137
Pestivirus A	MG950349	VIPR_ALG4_AWW87351_	1b	NS5B	B5	2015	-0.2730	0.5111	1.1137
Pestivirus A	MG950350	VIPR_ALG4_AWW87352_	1b	NS5B	B6	2015	-0.3086	0.5122	1.1137
Pestivirus A	MG950351	VIPR_ALG4_AWW87353_	1b	NS5B	O1	2015	-0.3086	0.5122	1.1137
Pestivirus A	MG950352	VIPR_ALG4_AWW87354_	1b	NS5B	O2	2015	-0.2582	0.5062	1.1120

Species according to VIPRBRC	GenBank Accession	GenBank Protein Accession	Subgenotype	Protein	Strain Name	Collection Year	SVM Patho. Score	Vaxijen Antig. Score	Averged score of EMBOSS motifs
Pestivirus A	MG950353	VIPR_ALG4_AWW87355_	1b	NS5B	O3	2015	-0.3056	0.5108	1.1120
Pestivirus A	MG950354	VIPR_ALG4_AWW87356_	1b	NS5B	O4	2015	-0.2991	0.5089	1.1122
Pestivirus A	MG950355	VIPR_ALG4_AWW87357_	1b	NS5B	O5	2015	-0.2991	0.5089	1.1122
Pestivirus A	MG950356	VIPR_ALG4_AWW87358_	1b	NS5B	O6	2015	-0.2991	0.5089	1.1122
Pestivirus A	MG950357	VIPR_ALG4_AWW87359_	1b	NS5B	B1A	2015	-0.2890	0.5124	1.1137
Pestivirus A	MG950358	VIPR_ALG4_AWW87360_	1b	NS5B	B2A	2016	-0.2743	0.5128	1.1137
Pestivirus A	MG950359	VIPR_ALG4_AWW87361_	1b	NS5B	B3A	2016	-0.3086	0.5122	1.1137
Pestivirus A	MG950360	VIPR_ALG4_AWW87362_	1b	NS5B	B4A	2016	-0.3086	0.5122	1.1137
Pestivirus A	MG950361	VIPR_ALG4_AWW87363_	1b	NS5B	B5A	2016	-0.3086	0.5122	1.1137
Pestivirus A	MG950362	VIPR_ALG4_AWW87364_	1b	NS5B	B6A	2016	-0.3086	0.5122	1.1137
Pestivirus A	MG950363	VIPR_ALG4_AWW87365_	1b	NS5B	O1A	2015	-0.2903	0.5180	1.1137
Pestivirus A	MG950364	VIPR_ALG4_AWW87366_	1b	NS5B	O2A	2015	-0.2825	0.5105	1.1139
Pestivirus A	MG950365	VIPR_ALG4_AWW87367_	1b	NS5B	O2B	2015	-0.2825	0.5105	1.1139
Pestivirus A	MG950366	VIPR_ALG4_AWW87368_	1b	NS5B	O4A	2015	-0.2991	0.5089	1.1122
Pestivirus A	MH311874	VIPR_ALG4_AWW87369_	1b	NS5B	B2A d168	2016	-0.3086	0.5122	1.1137
Pestivirus A	MH311875	VIPR_ALG4_AWW87370_	1b	NS5B	B3A d168	2016	-0.3086	0.5122	1.1137
Pestivirus A	MH311876	VIPR_ALG4_AWW87371_	1b	NS5B	B4A d84	2016	-0.3086	0.5122	1.1137
Pestivirus A	MH311877	VIPR_ALG4_AWW87372_	1b	NS5B	B4A d168	2016	-0.3086	0.5122	1.1137
Pestivirus A	MH311878	VIPR_ALG4_AWW87373_	1b	NS5B	B5A d84	2016	-0.3086	0.5122	1.1137
Pestivirus A	MH311879	VIPR_ALG4_AWW87374_	1b	NS5B	B5A d168	2016	-0.3086	0.5122	1.1137
Pestivirus A	MH311880	VIPR_ALG4_AWW87375_	1b	NS5B	B6A d84	2016	-0.3086	0.5122	1.1137
Pestivirus A	MH311881	VIPR_ALG4_AWW87376_	1b	NS5B	B6A d168	2016	-0.3086	0.5122	1.1137
Pestivirus A	MH379221	VIPR_ALG4_AWW87377_	1b	NS5B	P1	2017	-0.2825	0.5105	1.1139
Pestivirus A	MH379222	VIPR_ALG4_AWW87378_	1b	NS5B	P2	2017	-0.2825	0.5105	1.1139
Pestivirus A	MH379223	VIPR_ALG4_AWW87379_	1b	NS5B	P5	2017	-0.2825	0.5105	1.1139
Pestivirus A	MH379224	VIPR_ALG4_AWW87380_	1b	NS5B	P6	2017	-0.2825	0.5105	1.1139
Pestivirus A	MH379225	VIPR_ALG4_AWW87381_	1b	NS5B	P7	2017	-0.2825	0.5105	1.1139
Pestivirus A	MH379226	VIPR_ALG4_AWW87382_	1b	NS5B	P5A	2017	-0.2825	0.5105	1.1139
Pestivirus A	MH379227	VIPR_ALG4_AWW87383_	1b	NS5B	P5B	2017	-0.2825	0.5105	1.1139
Pestivirus A	MH379228	VIPR_ALG4_AWW87384_	1b	NS5B	P5C	2017	-0.2825	0.5105	1.1139
Pestivirus A	MH379229	VIPR_ALG4_AWW87385_	1b	NS5B	P5D	2017	-0.2825	0.5105	1.1139
Pestivirus A	MH379230	VIPR_ALG4_AWW87386_	1b	NS5B	P5F	2017	-0.2825	0.5105	1.1139
Pestivirus A	MH379231	VIPR_ALG4_AWW87387_	1b	NS5B	P7A	2018	-0.2825	0.5105	1.1139
Pestivirus A	MH379232	VIPR_ALG4_AWW87388_	1b	NS5B	P7C	2018	-0.2864	0.5060	1.1139
Pestivirus A	MH379233	VIPR_ALG4_AWW87389_	1b	NS5B	P7E	2018	-0.2825	0.5105	1.1139
Pestivirus A	MH379234	VIPR_ALG4_AWW87390_	1b	NS5B	P7F	2018	-0.2825	0.5105	1.1139
Pestivirus A	MH166806	VIPR_ALG4_AYA62524_1	1m	NS5B	XC	2015	-0.4072	0.4830	1.1176
Pestivirus A	MH490943	VIPR_ALG4_AZB53078_1	1b	NS5B	BVDV BJ-2016	2016	-0.2571	0.5186	1.1137
Pestivirus A	MH231153	VIPR_ALG4_AZQ00677_1	1b	NS5B	Nebraska	1990	-0.3075	0.5018	1.1124
Pestivirus A	AB078950	VIPR_ALG4_BAC55961_1	1j	NS5B	KS86-1ncp	1986	-0.2996	0.5124	1.1143
Pestivirus A	MH899941	VIPR_ALG4_QCE30388_1	1b	NS5B	SLO/3301/2014	2014	-0.2739	0.5273	1.1139
Pestivirus A	MH899942	VIPR_ALG4_QCE30389_1	1e	NS5B	SLO/33529/2015	2015	-0.1669	0.5168	1.1073
Pestivirus A	MH899943	VIPR_ALG4_QCE30390_1	1f	NS5B	SLO/1361/2014	2014	-0.3676	0.4850	1.1078
Pestivirus A	MH899944	VIPR_ALG4_QCE30391_1	1f	NS5B	SLO/28537/2017	2017	-0.4166	0.4975	1.1117
Pestivirus A	MH899945	VIPR_ALG4_QCE30392_1	1h	NS5B	SLO/1883/2013	2013	-0.4850	0.4902	1.1148
Pestivirus A	MK102095	VIPR_ALG4_QCQ84262_1	1q	NS5B	20170226	2017	-0.4446	0.5275	1.1243
Pestivirus A	MK509774	VIPR_ALG4_QEK23510_1	1b	NS5B	BVD1b-JH	2008	-0.2851	0.5170	1.1200

Species according to VIPRBRC	GenBank Accession	GenBank Protein Accession	Subgenotype	Protein	Strain Name	Collection Year	SVM Patho. Score	Vaxijen Antig. Score	Averged score of EMBOSS motifs
Pestivirus A	MK775204	VIPR_ALG4_QFX66041_1	1i	NS5B	CA2006	2006	-0.4956	0.5275	1.1136
Pestivirus A	MN188073	VIPR_ALG4_QGZ19414_1	1a	NS5B	PI34	2017	-0.3910	0.5318	1.1192
Pestivirus A	MN188074	VIPR_ALG4_QGZ19415_1	1b	NS5B	PI285	2017	-0.3238	0.5041	1.1161
Pestivirus A	MT079816	VIPR_ALG4_QIM55913_1	1c	NS5B	GXNN1	2018	-0.2243	0.5257	1.1096
Pestivirus A	MN623291	VIPR_ALG4_QLL27013_1	1m	NS5B	NX2019/01	2019	-0.2942	0.4616	1.1175
Pestivirus A	MW014286	VIPR_ALG4_QPJ59878_1	1b	NS5B	GXSS01	2018	-0.3899	0.5127	1.1131
Pestivirus A	MW014287	VIPR_ALG4_QPJ59879_1	1b	NS5B	GXSS02	2018	-0.3636	0.5186	1.1097
Pestivirus A	MW014288	VIPR_ALG4_QPJ59880_1	1b	NS5B	GXSS03	2018	-0.4597	0.5238	1.1093
Pestivirus A	MT977117	VIPR_ALG4_QRZ20359_1	1b	NS5B	BVDV 1b IT16/5	2016	-0.3508	0.5116	1.1126
Pestivirus A	MT977118	VIPR_ALG4_QRZ20360_1	1b	NS5B	BVDV 1b IT16/439	2016	-0.3503	0.5110	1.1126
Pestivirus A	MT654137	VIPR_ALG4_QVK82311_1	1a	NS5B	20-8536	2020	-0.3742	0.5257	1.1124
Pestivirus A	LT837585	VIPR_ALG4_SLV80196_1	1r	NS5B	UNKNOWN-LT837585	2012	-0.3641	0.4943	1.1105
Pestivirus A	MW054933	VIPR_ALG4_UEC94252_1	1f	NS5B	LA/230/14	2014	-0.3053	0.4952	1.1088
Pestivirus A	MW054934	VIPR_ALG4_UEC94253_1	1f	NS5B	LA/87/05	2005	-0.3793	0.4958	1.1074
Pestivirus A	MW054935	VIPR_ALG4_UEC94254_1	1k	NS5B	TO/197/11	2011	-0.3379	0.4992	1.1132
Pestivirus A	MW054936	VIPR_ALG4_UEC94255_1	1g	NS5B	UM/111/06	2006	-0.4704	0.4959	1.1164
Pestivirus A	MW054937	VIPR_ALG4_UEC94256_1	1k	NS5B	SA/158/09	2009	-0.0989	0.5096	1.1134
Pestivirus A	MW054938	VIPR_ALG4_UEC94257_1	1k	NS5B	SA/159/09	2009	-0.0989	0.5096	1.1134
Pestivirus A	MW054939	VIPR_ALG4_UEC94258_1	1f	NS5B	LO/151/09	2009	-0.3527	0.4973	1.1075
Pestivirus A	MW054940	VIPR_ALG4_UEC94259_1	1e	NS5B	MA/101/05	2005	-0.3383	0.5184	1.1088
Pestivirus A	MW250796	VIPR_ALG4_UEC94260_1	1i	NS5B	58-1	2008	-0.4597	0.5020	1.1135
Pestivirus A	MW250797	VIPR_ALG4_UEC94261_1	1i	NS5B	58-2	2008	-0.4597	0.5020	1.1135
Pestivirus A	MW250798	VIPR_ALG4_UEC94262_1	1a	NS5B	62-2	2008	-0.4277	0.5335	1.1127
Pestivirus A	MW250799	VIPR_ALG4_UEC94263_1	1a	NS5B	63-1	2008	-0.4320	0.5348	1.1166
Pestivirus A	MW250800	VIPR_ALG4_UEC94264_1	1d	NS5B	67-1	2008	-0.2867	0.5085	1.1195
Pestivirus A	MW250801	VIPR_ALG4_UEC94265_1	1d	NS5B	67-2	2008	-0.2867	0.5085	1.1195
Pestivirus A	MW250802	VIPR_ALG4_UEC94266_1	1e	NS5B	68-1	2008	-0.4045	0.5271	1.1083
Pestivirus A	MW250803	VIPR_ALG4_UEC94267_1	1i	NS5B	69-1	2008	-0.4068	0.5106	1.1152
Pestivirus A	MW655625	VIPR_ALG4_UEC94268_1	1h	NS5B	CH-04-01b	2004	-0.4423	0.5021	1.1168
Pestivirus A	MW655626	VIPR_ALG4_UEC94269_1	1e	NS5B	Maria	2004	-0.3155	0.5188	1.1072
Pestivirus A	MW655627	VIPR_ALG4_UEC94270_1	1e	NS5B	R2000-95	1995	-0.2145	0.5153	1.1088
Pestivirus A	MW655628	VIPR_ALG4_UEC94271_1	1k	NS5B	R3230-95	1995	-0.3728	0.4979	1.1164
Pestivirus A	MW655629	VIPR_ALG4_UEC94272_1	1h	NS5B	R3572-90	1990	-0.4331	0.4897	1.1176
Pestivirus A	MW655630	VIPR_ALG4_UEC94273_1	1k	NS5B	R5013-96	1996	-0.1761	0.5065	1.1176
Pestivirus A	MW655631	VIPR_ALG4_UEC94274_1	1e	NS5B	S03-1175	2003	-0.4505	0.5238	1.1074
Pestivirus A	MW655632	VIPR_ALG4_UEC94275_1	1h	NS5B	SM09-20	2002	-0.3860	0.5034	1.1167
Pestivirus A	MW713361	VIPR_ALG4_UEC94276_1	1a	NS5B	BoAEC1190	2007	-0.4066	0.5293	1.1141
Pestivirus A	MW713362	VIPR_ALG4_UEC94277_1	1b	NS5B	PI819	2017	-0.3338	0.5171	1.1133
Pestivirus A	MW732738	VIPR_ALG4_UEC94278_1	1a	NS5B	PI407	2015	-0.5677	0.5455	1.1157
Pestivirus A	MW732739	VIPR_ALG4_UEC94279_1	1a	NS5B	YandaSpl	1993	-0.3434	0.5508	1.1130
Pestivirus A	MZ188972	VIPR_ALG4_UML14262_1	1q	NS5B	HB-1	2020	-0.4700	0.5077	1.1140
Pestivirus A	ON337882	VIPR_ALG4_USZ80113_1	1c	NS5B	NM2103	2021	-0.3890	0.5360	1.1129
Pestivirus A	KU159365	VIPR_ALG4_1039262063_	1a	NS2	USII-S15	2015	-0.1970	0.5784	1.1468
Pestivirus A	KU756226	VIPR_ALG4_1072900294_	1b	NS2	HJ-1	2010	-0.7296	0.6348	1.1405
Pestivirus A	KT943518	VIPR_ALG4_1093530908_	1d	NS2	BJ1201	2012	-0.5452	0.5509	1.1541
Pestivirus A	LT631725	VIPR_ALG4_1112914034_	1h	NS2	UM/126/07	2007	-0.9286	0.5663	1.1495
Pestivirus A	KX170371	VIPR_ALG4_1129880008_	1b	NS2	V015	2001	-1.0417	0.5913	1.1395

Species according to VIPRBRC	GenBank Accession	GenBank Protein Accession	Subgenotype	Protein	Strain Name	Collection Year	SVM Patho. Score	Vaxijen Antig. Score	Averged score of EMBOSS motifs
Pestivirus A	KX170372	VIPR_ALG4_1129880010_	1b	NS2	V070	2007	-0.5173	0.6410	1.1388
Pestivirus A	KX170373	VIPR_ALG4_1129880012_	1b	NS2	V078	2012	-0.6330	0.6448	1.1419
Pestivirus A	KX170374	VIPR_ALG4_1129880014_	1b	NS2	V045	2009	-0.5693	0.6391	1.1350
Pestivirus A	KX170375	VIPR_ALG4_1129880016_	1b	NS2	V031	2006	-0.6962	0.6285	1.1371
Pestivirus A	KX170376	VIPR_ALG4_1129880018_	1b	NS2	V087	2006	-0.7009	0.6241	1.1367
Pestivirus A	KX170377	VIPR_ALG4_1129880020_	1b	NS2	V098	1999	-0.5401	0.6281	1.1440
Pestivirus A	KX170378	VIPR_ALG4_1129880022_	1b	NS2	V036	2007	-0.5599	0.6419	1.1401
Pestivirus A	KX170379	VIPR_ALG4_1129880024_	1b	NS2	V020	2005	-0.6619	0.6402	1.1349
Pestivirus A	KX170380	VIPR_ALG4_1129880026_	1b	NS2	V029	2006	-0.6959	0.6216	1.1374
Pestivirus A	KX170381	VIPR_ALG4_1129880028_	1a	NS2	V091	2003	-0.2232	0.5817	1.1481
Pestivirus A	KX170382	VIPR_ALG4_1129880030_	1a	NS2	V007	2000	-0.1555	0.5778	1.1481
Pestivirus A	KX170383	VIPR_ALG4_1129880032_	1a	NS2	V013	2001	-0.1555	0.5778	1.1481
Pestivirus A	KX170384	VIPR_ALG4_1129880034_	1a	NS2	V033	2007	-0.1555	0.5778	1.1481
Pestivirus A	KX170385	VIPR_ALG4_1129880036_	1a	NS2	V034	2007	-0.1555	0.5778	1.1481
Pestivirus A	KX170386	VIPR_ALG4_1129880038_	1a	NS2	V049	2009	-0.1555	0.5778	1.1481
Pestivirus A	KX170387	VIPR_ALG4_1129880040_	1a	NS2	V067	2006	-0.1555	0.5778	1.1481
Pestivirus A	KX170388	VIPR_ALG4_1129880042_	1a	NS2	V073	2011	-0.1555	0.5778	1.1481
Pestivirus A	KX170389	VIPR_ALG4_1129880044_	1a	NS2	V077	2012	-0.1555	0.5778	1.1481
Pestivirus A	KX170390	VIPR_ALG4_1129880046_	1a	NS2	V080	2009	-0.1555	0.5778	1.1481
Pestivirus A	KX170391	VIPR_ALG4_1129880048_	1a	NS2	V074	2010	-0.1354	0.5780	1.1506
Pestivirus A	KX170392	VIPR_ALG4_1129880050_	1a	NS2	V022	2006	-0.2292	0.5792	1.1543
Pestivirus A	KX170393	VIPR_ALG4_1129880052_	1a	NS2	V092	2004	-0.2453	0.5698	1.1538
Pestivirus A	KX170394	VIPR_ALG4_1129880054_	1a	NS2	V054	2013	-0.4222	0.6019	1.1520
Pestivirus A	KX170395	VIPR_ALG4_1129880056_	1a	NS2	V011	2001	-0.3595	0.5886	1.1547
Pestivirus A	KX170396	VIPR_ALG4_1129880058_	1a	NS2	V012	2001	-0.3595	0.5886	1.1547
Pestivirus A	KX170397	VIPR_ALG4_1129880060_	1a	NS2	V056	2009	-0.4029	0.5836	1.1533
Pestivirus A	KX170398	VIPR_ALG4_1129880062_	1a	NS2	V057	2009	-0.4405	0.5837	1.1493
Pestivirus A	KX170399	VIPR_ALG4_1129880064_	1a	NS2	V006	2000	-0.4545	0.5799	1.1514
Pestivirus A	KX170400	VIPR_ALG4_1129880066_	1a	NS2	V001	1999	-0.3511	0.5733	1.1431
Pestivirus A	KX170401	VIPR_ALG4_1129880068_	1a	NS2	V008	2000	-0.4270	0.5778	1.1433
Pestivirus A	KX170402	VIPR_ALG4_1129880070_	1a	NS2	V009	2000	-0.4270	0.5778	1.1433
Pestivirus A	KX170403	VIPR_ALG4_1129880072_	1a	NS2	V010	2001	-0.3513	0.6075	1.1419
Pestivirus A	KX170404	VIPR_ALG4_1129880074_	1a	NS2	V035	2007	-0.5154	0.5486	1.1463
Pestivirus A	KX170405	VIPR_ALG4_1129880076_	1a	NS2	V050	2009	-0.5129	0.5523	1.1474
Pestivirus A	KX170406	VIPR_ALG4_1129880078_	1a	NS2	V039	2008	-0.5701	0.5593	1.1438
Pestivirus A	KX170407	VIPR_ALG4_1129880080_	1a	NS2	V041	2008	-0.5459	0.5584	1.1438
Pestivirus A	KX170408	VIPR_ALG4_1129880082_	1a	NS2	V042	2008	-0.6030	0.5511	1.1463
Pestivirus A	KX170409	VIPR_ALG4_1129880084_	1a	NS2	V043	2008	-0.5784	0.5502	1.1463
Pestivirus A	KX170410	VIPR_ALG4_1129880086_	1a	NS2	V040	2008	-0.5091	0.5669	1.1463
Pestivirus A	KX170411	VIPR_ALG4_1129880088_	1a	NS2	V046	2009	-0.5233	0.5534	1.1463
Pestivirus A	KX170412	VIPR_ALG4_1129880090_	1a	NS2	V052	2010	-0.5471	0.5543	1.1463
Pestivirus A	KX170413	VIPR_ALG4_1129880092_	1a	NS2	V083	2008	-0.5441	0.5763	1.1524
Pestivirus A	KX170414	VIPR_ALG4_1129880094_	1a	NS2	V099	1998	-0.6259	0.5677	1.1549
Pestivirus A	KX170415	VIPR_ALG4_1129880096_	1a	NS2	V059	2004	-0.4858	0.5865	1.1446
Pestivirus A	KX170416	VIPR_ALG4_1129880098_	1b	NS2	V060	2004	-0.5833	0.6351	1.1486
Pestivirus A	EF101530	VIPR_ALG4_118498779_	1b	NS2	KE9	2007	-0.6310	0.5755	1.1483
Pestivirus A	DQ088995	VIPR_ALG4_145309048_	1a	NS2	Singer_Arg	1974	-0.2824	0.5781	1.1457

Species according to VIPRBRC	GenBank Accession	GenBank Protein Accession	Subgenotype	Protein	Strain Name	Collection Year	SVM Patho. Score	Vaxijen Antig. Score	Averged score of EMBOSS motifs
Pestivirus A	U63479	VIPR_ALG4_1518836_37	1b	NS2	CP7	1987	-0.6389	0.6300	1.1351
Pestivirus A	U86600	VIPR_ALG4_2149469_379	1b	NS2	ILLNC	1991	-0.7920	0.6345	1.1395
Pestivirus A	AF041040	VIPR_ALG4_2789677_37	1a	NS2	Oregon	1960	-0.4073	0.5806	1.1433
Pestivirus A	M96751	VIPR_ALG4_289508_379	1a	NS2	UNKNOWN-M96751	1992	-0.4094	0.5741	1.1419
Pestivirus A	HQ174292	VIPR_ALG4_323145267_	1a	NS2	180	2010	-0.5658	0.5509	1.1486
Pestivirus A	M31182	VIPR_ALG4_323206_379	1a	NS2	UNKNOWN-M31182	1988	-0.5595	0.5997	1.1367
Pestivirus A	M96687	VIPR_ALG4_323230_379	1b	NS2	Osloss	1967	-0.7104	0.6207	1.1366
Pestivirus A	JN400273	VIPR_ALG4_363990275_	1q	NS2	SD0803	2008	-0.8518	0.5998	1.1531
Pestivirus A	AF091605	VIPR_ALG4_3661566_379	1a	NS2	Oregon C24V	1960	-0.4858	0.5865	1.1446
Pestivirus A	JN644055	VIPR_ALG4_373939303_	1b	NS2	3156	2011	-0.3905	0.5321	1.1395
Pestivirus A	JN380080	VIPR_ALG4_378753653_	1a	NS2	6010	2010	-0.5658	0.5509	1.1486
Pestivirus A	JQ799141	VIPR_ALG4_390132765_	1u	NS2	M31182	2010	-0.6283	0.4863	1.1618
Pestivirus A	JX419397	VIPR_ALG4_404363562_	1b	NS2	UNKNOWN-JX419397	2008	-0.6181	0.6399	1.1429
Pestivirus A	JX419398	VIPR_ALG4_404363564_	1b	NS2	UNKNOWN-JX419398	2008	-0.6181	0.6399	1.1429
Pestivirus A	AF526381	VIPR_ALG4_42476348_37	1m	NS2	ZM-95	1995	-0.4527	0.6056	1.1456
Pestivirus A	JX297512	VIPR_ALG4_459284067_	1b	NS2	10270	2007	-0.4826	0.6351	1.1411
Pestivirus A	JX297513	VIPR_ALG4_459284069_	1b	NS2	Aries	2005	-0.4530	0.6373	1.1411
Pestivirus A	JX297514	VIPR_ALG4_459284071_	1b	NS2	Columba	2005	-0.4530	0.6373	1.1411
Pestivirus A	JX297515	VIPR_ALG4_459284073_	1b	NS2	Corona	2005	-0.4530	0.6373	1.1411
Pestivirus A	JX297516	VIPR_ALG4_459284075_	1b	NS2	Gemini	2005	-0.4795	0.6321	1.1411
Pestivirus A	JX297517	VIPR_ALG4_459284077_	1b	NS2	Hercules	2006	-0.5536	0.6396	1.1352
Pestivirus A	JX297518	VIPR_ALG4_459284079_	1b	NS2	Leo	2006	-0.4530	0.6373	1.1411
Pestivirus A	JX297519	VIPR_ALG4_459284081_	1b	NS2	Lyra	2006	-0.4530	0.6373	1.1411
Pestivirus A	JX297520	VIPR_ALG4_459284083_	1b	NS2	Mars	2006	-0.4530	0.6373	1.1411
Pestivirus A	JX297521	VIPR_ALG4_459284085_	1b	NS2	Scorpius	2006	-0.4530	0.6373	1.1411
Pestivirus A	KC853440	VIPR_ALG4_507144146_	1k	NS2	SuwaNcp	1993	-0.4679	0.5672	1.1463
Pestivirus A	KC853441	VIPR_ALG4_507144148_	1k	NS2	SuwaCp	1993	-0.5124	0.5274	1.1426
Pestivirus A	KC695810	VIPR_ALG4_507866685_	1q	NS2	camel-6	2010	-0.8131	0.5811	1.1504
Pestivirus A	KC695814	VIPR_ALG4_507866704_	1b	NS2	Av69 VEDEVAC	2011	-0.6109	0.5744	1.1391
Pestivirus A	KC757383	VIPR_ALG4_511775165_	1d	NS2	10JJ-SKR	2010	-0.4476	0.5443	1.1526
Pestivirus A	KC963967	VIPR_ALG4_530291194_	1b	NS2	12F004	2012	-0.5363	0.6438	1.1385
Pestivirus A	KF772785	VIPR_ALG4_575471151_	1b	NS2	CC13B	2013	-0.6058	0.6448	1.1373
Pestivirus A	KF896608	VIPR_ALG4_586616532_	1c	NS2	Bega-like	2012	-0.5614	0.5906	1.1577
Pestivirus A	KF835697	VIPR_ALG4_597437474_	1b	NS2	AU526	2013	-0.4375	0.6449	1.1379
Pestivirus A	KJ541471	VIPR_ALG4_633265982_	1a	NS2	GS5	2013	-0.5352	0.5601	1.1484
Pestivirus A	KJ689448	VIPR_ALG4_635172915_	1b	NS2	GX4	2012	-0.6395	0.5858	1.1439
Pestivirus A	KF501393	VIPR_ALG4_669206614_	1b	NS2	BVDV JL-1	2009	-0.6485	0.6284	1.1378
Pestivirus A	AJ133738	VIPR_ALG4_7960754_379	1a	NS2	type 1	1963	-0.5872	0.5986	1.1367
Pestivirus A	KP941581	VIPR_ALG4_800924313_	1b	NS2	USMARC-51998	2014	-0.5466	0.6294	1.1447
Pestivirus A	KP941583	VIPR_ALG4_800924317_	1b	NS2	USMARC-53874	2014	-0.3781	0.6506	1.1433
Pestivirus A	KP941584	VIPR_ALG4_800924319_	1a	NS2	USMARC-53875	2014	-0.3269	0.5560	1.1383
Pestivirus A	KP941586	VIPR_ALG4_800924323_	1a	NS2	USMARC-55477	2014	-0.4082	0.5678	1.1514
Pestivirus A	KP941587	VIPR_ALG4_800924325_	1b	NS2	USMARC-55478	2014	-0.6663	0.6643	1.1378
Pestivirus A	KP941588	VIPR_ALG4_800924327_	1b	NS2	USMARC-55922	2014	-0.7205	0.6100	1.1373
Pestivirus A	KP941589	VIPR_ALG4_800924329_	1b	NS2	USMARC-55923	2014	-0.4640	0.6236	1.1306
Pestivirus A	KP941590	VIPR_ALG4_800924331_	1b	NS2	USMARC-55924	2014	-0.5190	0.6426	1.1410
Pestivirus A	KP941591	VIPR_ALG4_800924333_	1b	NS2	USMARC-55925	2014	-0.9756	0.5894	1.1445

Species according to VIPRBRC	GenBank Accession	GenBank Protein Accession	Subgenotype	Protein	Strain Name	Collection Year	SVM Patho. Score	Vaxijen Antig. Score	Averged score of EMBOSS motifs
Pestivirus A	KP941592	VIPR_ALG4_800924335_1	1b	NS2	USMARC-55926	2014	-0.3810	0.6394	1.1397
Pestivirus A	KP313732	VIPR_ALG4_816850387_1	1e	NS2	Carlito	2014	-0.2704	0.5948	1.1551
Pestivirus A	KR029825	VIPR_ALG4_887497286_1	1b	NS2	Egy/Ismailia/2014	2014	-0.6004	0.6357	1.1402
Pestivirus A	LC089875	VIPR_ALG4_939106262_1	1o	NS2	IS26/01ncp	2001	-0.8240	0.6102	1.1472
Pestivirus A	LC089876	VIPR_ALG4_939106264_1	1n	NS2	Shitara/02/06	2006	-0.8517	0.5044	1.1591
Pestivirus A	KR866116	VIPR_ALG4_941508008_1	1m	NS2	SD-15	2015	-0.4277	0.6007	1.1438
Pestivirus A	KU200260	VIPR_ALG4_972905813_1	1b	NS2	BE/061536/2014	2014	-0.7406	0.5863	1.1429
Pestivirus A	KX577637	VIPR_ALG4_AOR50934_1	1e	NS2	SLO/2407/2006	2006	-0.3066	0.5799	1.1524
Pestivirus A	KX987157	VIPR_ALG4_APG30987_1	1f	NS2	SLO/1170/2000	2000	-0.7512	0.5701	1.1504
Pestivirus A	KX857724	VIPR_ALG4_APZ85839_1	1i	NS2	ACM/BR/2016	2016	-0.6231	0.5954	1.1501
Pestivirus A	KY849592	VIPR_ALG4_ART90617_1	1d	NS2	SLO/2416/2002	2002	-0.8508	0.5759	1.1513
Pestivirus A	MF278651	VIPR_ALG4_ASW18434_1	1b	NS2	XZ01	2016	-0.6160	0.5711	1.1374
Pestivirus A	MF278652	VIPR_ALG4_ASW18435_1	1b	NS2	XZ02	2016	-0.6501	0.6309	1.1378
Pestivirus A	MF693403	VIPR_ALG4_ATG71375_1	1a	NS2	UNKNOWN-MF693403	2016	-0.2144	0.5516	1.1514
Pestivirus A	KY964311	VIPR_ALG4_ATN39078_1	1b	NS2	Y2	2014	-0.6276	0.5774	1.1568
Pestivirus A	MF172980	VIPR_ALG4_AVI10261_1	1c	NS2	GSTZ	2012	-0.5248	0.5743	1.1687
Pestivirus A	MH379638	VIPR_ALG4_AWW14171_	1a	NS2	Ho916	1993	-0.3213	0.5812	1.1473
Pestivirus A	MG950344	VIPR_ALG4_AWW87346_	1b	NS2	AU526	2014	-0.4375	0.6449	1.1379
Pestivirus A	MG950345	VIPR_ALG4_AWW87347_	1b	NS2	B1	2015	-0.3779	0.6388	1.1379
Pestivirus A	MG950346	VIPR_ALG4_AWW87348_	1b	NS2	B2	2015	-0.4375	0.6449	1.1379
Pestivirus A	MG950347	VIPR_ALG4_AWW87349_	1b	NS2	B3	2015	-0.4375	0.6449	1.1379
Pestivirus A	MG950348	VIPR_ALG4_AWW87350_	1b	NS2	B4	2015	-0.4375	0.6449	1.1379
Pestivirus A	MG950349	VIPR_ALG4_AWW87351_	1b	NS2	B5	2015	-0.4526	0.6437	1.1379
Pestivirus A	MG950350	VIPR_ALG4_AWW87352_	1b	NS2	B6	2015	-0.4375	0.6449	1.1379
Pestivirus A	MG950351	VIPR_ALG4_AWW87353_	1b	NS2	O1	2015	-0.4375	0.6449	1.1379
Pestivirus A	MG950352	VIPR_ALG4_AWW87354_	1b	NS2	O2	2015	-0.4620	0.6400	1.1379
Pestivirus A	MG950353	VIPR_ALG4_AWW87355_	1b	NS2	O3	2015	-0.4484	0.6479	1.1379
Pestivirus A	MG950354	VIPR_ALG4_AWW87356_	1b	NS2	O4	2015	-0.4484	0.6479	1.1379
Pestivirus A	MG950355	VIPR_ALG4_AWW87357_	1b	NS2	O5	2015	-0.4484	0.6479	1.1379
Pestivirus A	MG950356	VIPR_ALG4_AWW87358_	1b	NS2	O6	2015	-0.4484	0.6479	1.1379
Pestivirus A	MG950357	VIPR_ALG4_AWW87359_	1b	NS2	B1A	2015	-0.4574	0.6440	1.1402
Pestivirus A	MG950358	VIPR_ALG4_AWW87360_	1b	NS2	B2A	2016	-0.4375	0.6449	1.1379
Pestivirus A	MG950359	VIPR_ALG4_AWW87361_	1b	NS2	B3A	2016	-0.4375	0.6449	1.1379
Pestivirus A	MG950360	VIPR_ALG4_AWW87362_	1b	NS2	B4A	2016	-0.4375	0.6449	1.1379
Pestivirus A	MG950361	VIPR_ALG4_AWW87363_	1b	NS2	B5A	2016	-0.4375	0.6449	1.1379
Pestivirus A	MG950362	VIPR_ALG4_AWW87364_	1b	NS2	B6A	2016	-0.4375	0.6449	1.1379
Pestivirus A	MG950363	VIPR_ALG4_AWW87365_	1b	NS2	O1A	2015	-0.5378	0.6501	1.1402
Pestivirus A	MG950364	VIPR_ALG4_AWW87366_	1b	NS2	O2A	2015	-0.4484	0.6479	1.1379
Pestivirus A	MG950365	VIPR_ALG4_AWW87367_	1b	NS2	O2B	2015	-0.4484	0.6479	1.1379
Pestivirus A	MG950366	VIPR_ALG4_AWW87368_	1b	NS2	O4A	2015	-0.4484	0.6479	1.1379
Pestivirus A	MH311874	VIPR_ALG4_AWW87369_	1b	NS2	B2A d168	2016	-0.4375	0.6449	1.1379
Pestivirus A	MH311875	VIPR_ALG4_AWW87370_	1b	NS2	B3A d168	2016	-0.4375	0.6449	1.1379
Pestivirus A	MH311876	VIPR_ALG4_AWW87371_	1b	NS2	B4A d84	2016	-0.4375	0.6449	1.1379
Pestivirus A	MH311877	VIPR_ALG4_AWW87372_	1b	NS2	B4A d168	2016	-0.4375	0.6449	1.1379
Pestivirus A	MH311878	VIPR_ALG4_AWW87373_	1b	NS2	B5A d84	2016	-0.4375	0.6449	1.1379
Pestivirus A	MH311879	VIPR_ALG4_AWW87374_	1b	NS2	B5A d168	2016	-0.4375	0.6449	1.1379
Pestivirus A	MH311880	VIPR_ALG4_AWW87375_	1b	NS2	B6A d84	2016	-0.4375	0.6449	1.1379

Species according to VIPRBRC	GenBank Accession	GenBank Protein Accession	Subgenotype	Protein	Strain Name	Collection Year	SVM Patho. Score	Vaxijen Antig. Score	Averged score of EMBOSS motifs
Pestivirus A	MH311881	VIPR_ALG4_AWW87376_	1b	NS2	B6A d168	2016	-0.4375	0.6449	1.1379
Pestivirus A	MH379221	VIPR_ALG4_AWW87377_	1b	NS2	P1	2017	-0.4484	0.6479	1.1379
Pestivirus A	MH379222	VIPR_ALG4_AWW87378_	1b	NS2	P2	2017	-0.4484	0.6479	1.1379
Pestivirus A	MH379223	VIPR_ALG4_AWW87379_	1b	NS2	P5	2017	-0.4667	0.6480	1.1383
Pestivirus A	MH379224	VIPR_ALG4_AWW87380_	1b	NS2	P6	2017	-0.4484	0.6479	1.1379
Pestivirus A	MH379225	VIPR_ALG4_AWW87381_	1b	NS2	P7	2017	-0.4484	0.6479	1.1379
Pestivirus A	MH379226	VIPR_ALG4_AWW87382_	1b	NS2	P5A	2017	-0.4484	0.6479	1.1379
Pestivirus A	MH379227	VIPR_ALG4_AWW87383_	1b	NS2	P5B	2017	-0.4484	0.6479	1.1379
Pestivirus A	MH379228	VIPR_ALG4_AWW87384_	1b	NS2	P5C	2017	-0.4667	0.6480	1.1383
Pestivirus A	MH379229	VIPR_ALG4_AWW87385_	1b	NS2	P5D	2017	-0.4484	0.6479	1.1379
Pestivirus A	MH379230	VIPR_ALG4_AWW87386_	1b	NS2	P5F	2017	-0.4667	0.6480	1.1383
Pestivirus A	MH379231	VIPR_ALG4_AWW87387_	1b	NS2	P7A	2018	-0.4667	0.6480	1.1383
Pestivirus A	MH379232	VIPR_ALG4_AWW87388_	1b	NS2	P7C	2018	-0.4488	0.6505	1.1383
Pestivirus A	MH379233	VIPR_ALG4_AWW87389_	1b	NS2	P7E	2018	-0.4667	0.6480	1.1383
Pestivirus A	MH379234	VIPR_ALG4_AWW87390_	1b	NS2	P7F	2018	-0.4667	0.6480	1.1383
Pestivirus A	MH166806	VIPR_ALG4_AYA62524_1	1m	NS2	XC	2015	-0.4989	0.5991	1.1418
Pestivirus A	MH490943	VIPR_ALG4_AZB53078_1	1b	NS2	BVDV BJ-2016	2016	-0.5724	0.6329	1.1437
Pestivirus A	MH231153	VIPR_ALG4_AZQ00677_1	1b	NS2	Nebraska	1990	-0.4195	0.6376	1.1401
Pestivirus A	AB078950	VIPR_ALG4_BAC55961_1	1j	NS2	KS86-1ncp	1986	-0.6020	0.5884	1.1546
Pestivirus A	MH899941	VIPR_ALG4_QCE30388_1	1b	NS2	SLO/3301/2014	2014	-0.8080	0.6316	1.1354
Pestivirus A	MH899942	VIPR_ALG4_QCE30389_1	1e	NS2	SLO/33529/2015	2015	-0.3138	0.5989	1.1528
Pestivirus A	MH899943	VIPR_ALG4_QCE30390_1	1f	NS2	SLO/1361/2014	2014	-1.0353	0.5913	1.1403
Pestivirus A	MH899944	VIPR_ALG4_QCE30391_1	1f	NS2	SLO/28537/2017	2017	-0.9729	0.6087	1.1424
Pestivirus A	MH899945	VIPR_ALG4_QCE30392_1	1h	NS2	SLO/1883/2013	2013	-0.8366	0.5544	1.1482
Pestivirus A	MK102095	VIPR_ALG4_QCQ84262_1	1q	NS2	20170226	2017	-0.7755	0.5957	1.1478
Pestivirus A	MK509774	VIPR_ALG4_QEK23510_1	1b	NS2	BVD1b-JH	2008	-0.6298	0.6434	1.1423
Pestivirus A	MN188073	VIPR_ALG4_QGZ19414_1	1a	NS2	PI34	2017	-0.3299	0.5697	1.1414
Pestivirus A	MN188074	VIPR_ALG4_QGZ19415_1	1b	NS2	PI285	2017	-0.5022	0.6336	1.1392
Pestivirus A	MT079816	VIPR_ALG4_QIM55913_1	1c	NS2	GXNN1	2018	-0.6136	0.5863	1.1644
Pestivirus A	MN623291	VIPR_ALG4_QLL27013_1	1m	NS2	NX2019/01	2019	-0.5638	0.5946	1.1409
Pestivirus A	MW014286	VIPR_ALG4_QPJ59878_1	1b	NS2	GXSS01	2018	-0.6688	0.5940	1.1428
Pestivirus A	MW014287	VIPR_ALG4_QPJ59879_1	1b	NS2	GXSS02	2018	-0.3947	0.5832	1.1539
Pestivirus A	MW014288	VIPR_ALG4_QPJ59880_1	1b	NS2	GXSS03	2018	-0.4271	0.5799	1.1519
Pestivirus A	MT977117	VIPR_ALG4_QRZ20359_1	1b	NS2	BVDV 1b IT16/5	2016	-0.5987	0.5860	1.1447
Pestivirus A	MT977118	VIPR_ALG4_QRZ20360_1	1b	NS2	BVDV 1b IT16/439	2016	-0.5987	0.5860	1.1447
Pestivirus A	MT654137	VIPR_ALG4_QVK82311_1	1a	NS2	20-8536	2020	-0.7985	0.5569	1.1463
Pestivirus A	LT837585	VIPR_ALG4_SLV80196_1	1r	NS2	UNKNOWN-LT837585	2012	-0.8393	0.5754	1.1550
Pestivirus A	MW054933	VIPR_ALG4_UEC94252_1	1f	NS2	LA/230/14	2014	-0.8460	0.5878	1.1459
Pestivirus A	MW054934	VIPR_ALG4_UEC94253_1	1f	NS2	LA/87/05	2005	-0.7865	0.5956	1.1478
Pestivirus A	MW054935	VIPR_ALG4_UEC94254_1	1k	NS2	TO/197/11	2011	-0.5098	0.5697	1.1491
Pestivirus A	MW054936	VIPR_ALG4_UEC94255_1	1g	NS2	UM/111/06	2006	-1.0109	0.5792	1.1399
Pestivirus A	MW054937	VIPR_ALG4_UEC94256_1	1k	NS2	SA/158/09	2009	-0.6395	0.5928	1.1459
Pestivirus A	MW054938	VIPR_ALG4_UEC94257_1	1k	NS2	SA/159/09	2009	-0.6395	0.5928	1.1459
Pestivirus A	MW054939	VIPR_ALG4_UEC94258_1	1f	NS2	LO/151/09	2009	-0.8764	0.5811	1.1492
Pestivirus A	MW054940	VIPR_ALG4_UEC94259_1	1e	NS2	MA/101/05	2005	-0.5107	0.5627	1.1455
Pestivirus A	MW250796	VIPR_ALG4_UEC94260_1	1i	NS2	58-1	2008	-0.5579	0.5771	1.1541
Pestivirus A	MW250797	VIPR_ALG4_UEC94261_1	1i	NS2	58-2	2008	-0.5579	0.5771	1.1541

Species according to VIPRBRC	GenBank Accession	GenBank Protein Accession	Subgenotype	Protein	Strain Name	Collection Year	SVM Patho. Score	Vaxijen Antig. Score	Averged score of EMBOSS motifs
Pestivirus A	MW250798	VIPR_ALG4_UEC94262_1	1a	NS2	62-2	2008	-0.5256	0.6030	1.1529
Pestivirus A	MW250799	VIPR_ALG4_UEC94263_1	1a	NS2	63-1	2008	-0.6683	0.5968	1.1498
Pestivirus A	MW250800	VIPR_ALG4_UEC94264_1	1d	NS2	67-1	2008	-0.7594	0.5412	1.1448
Pestivirus A	MW250801	VIPR_ALG4_UEC94265_1	1d	NS2	67-2	2008	-0.7594	0.5412	1.1448
Pestivirus A	MW250802	VIPR_ALG4_UEC94266_1	1e	NS2	68-1	2008	-0.5491	0.5712	1.1450
Pestivirus A	MW250803	VIPR_ALG4_UEC94267_1	1i	NS2	69-1	2008	-0.4382	0.5955	1.1378
Pestivirus A	MW655625	VIPR_ALG4_UEC94268_1	1h	NS2	CH-04-01b	2004	-0.7724	0.5773	1.1511
Pestivirus A	MW655626	VIPR_ALG4_UEC94269_1	1e	NS2	Maria	2004	-0.3541	0.6185	1.1580
Pestivirus A	MW655627	VIPR_ALG4_UEC94270_1	1e	NS2	R2000-95	1995	-0.3793	0.5928	1.1466
Pestivirus A	MW655628	VIPR_ALG4_UEC94271_1	1k	NS2	R3230-95	1995	-0.5070	0.5975	1.1422
Pestivirus A	MW655629	VIPR_ALG4_UEC94272_1	1h	NS2	R3572-90	1990	-0.7763	0.5390	1.1516
Pestivirus A	MW655630	VIPR_ALG4_UEC94273_1	1k	NS2	R5013-96	1996	-0.5308	0.5815	1.1519
Pestivirus A	MW655631	VIPR_ALG4_UEC94274_1	1e	NS2	S03-1175	2003	-0.4151	0.5974	1.1516
Pestivirus A	MW655632	VIPR_ALG4_UEC94275_1	1h	NS2	SM09-20	2002	-0.9082	0.5685	1.1509
Pestivirus A	MW713361	VIPR_ALG4_UEC94276_1	1a	NS2	BoAEC1190	2007	-0.3085	0.5844	1.1505
Pestivirus A	MW713362	VIPR_ALG4_UEC94277_1	1b	NS2	PI819	2017	-0.4375	0.6449	1.1379
Pestivirus A	MW732738	VIPR_ALG4_UEC94278_1	1a	NS2	PI407	2015	-0.4877	0.5509	1.1417
Pestivirus A	MW732739	VIPR_ALG4_UEC94279_1	1a	NS2	YandaSpl	1993	-0.8626	0.5435	1.1522
Pestivirus A	MZ188972	VIPR_ALG4_UML14262_1	1q	NS2	HB-1	2020	-0.6331	0.5971	1.1405
Pestivirus A	ON337882	VIPR_ALG4_USZ80113_1	1c	NS2	NM2103	2021	-0.2274	0.5560	1.1608
Pestivirus A	KU159365	VIPR_ALG4_1039262063	1a	C	USII-S15	2015	0.1153	0.6079	1.1260
Pestivirus A	KU756226	VIPR_ALG4_1072900294	1b	C	HJ-1	2010	-0.1925	0.6128	1.1417
Pestivirus A	KT943518	VIPR_ALG4_1093530908	1d	C	BJ1201	2012	-0.0926	0.5582	1.1595
Pestivirus A	LT631725	VIPR_ALG4_1112914034	1h	C	UM/126/07	2007	0.2317	0.5897	1.1655
Pestivirus A	KX169986	VIPR_ALG4_1129879238	1b	C	V015	2001	0.0314	0.5931	1.1725
Pestivirus A	KX169987	VIPR_ALG4_1129879240	1b	C	V070	2007	0.0512	0.5740	1.1820
Pestivirus A	KX169988	VIPR_ALG4_1129879242	1b	C	V060	2004	0.3279	0.5975	1.1560
Pestivirus A	KX169989	VIPR_ALG4_1129879244	1b	C	V036	2007	0.1735	0.6182	1.1610
Pestivirus A	KX169990	VIPR_ALG4_1129879246	1b	C	V100	1997	-0.0599	0.5848	1.1820
Pestivirus A	KX169991	VIPR_ALG4_1129879248	1b	C	V075	2011	0.2612	0.6046	1.1725
Pestivirus A	KX169992	VIPR_ALG4_1129879250	1b	C	V098	1999	0.1592	0.6042	1.1725
Pestivirus A	KX169993	VIPR_ALG4_1129879252	1b	C	V078	2012	0.3138	0.5945	1.1730
Pestivirus A	KX169994	VIPR_ALG4_1129879254	1b	C	V045	2009	0.3061	0.5744	1.1660
Pestivirus A	KX169995	VIPR_ALG4_1129879256	1b	C	V031	2006	0.1592	0.6042	1.1725
Pestivirus A	KX169996	VIPR_ALG4_1129879258	1b	C	V087	2006	0.0408	0.5814	1.1725
Pestivirus A	KX169997	VIPR_ALG4_1129879260	1b	C	V020	2005	0.1592	0.6042	1.1725
Pestivirus A	KX169998	VIPR_ALG4_1129879262	1b	C	V029	2006	0.1592	0.6042	1.1725
Pestivirus A	KX169999	VIPR_ALG4_1129879264	1a	C	V026	2006	-0.8476	0.5795	1.1645
Pestivirus A	KX170000	VIPR_ALG4_1129879266	1a	C	V027	2006	-0.8476	0.5795	1.1645
Pestivirus A	KX170001	VIPR_ALG4_1129879268	1a	C	V007	2000	-0.1695	0.5724	1.1157
Pestivirus A	KX170002	VIPR_ALG4_1129879270	1a	C	V013	2001	-0.1695	0.5724	1.1157
Pestivirus A	KX170003	VIPR_ALG4_1129879272	1a	C	V033	2007	-0.1695	0.5724	1.1157
Pestivirus A	KX170004	VIPR_ALG4_1129879274	1a	C	V034	2007	-0.1695	0.5724	1.1157
Pestivirus A	KX170005	VIPR_ALG4_1129879276	1a	C	V049	2009	-0.1695	0.5724	1.1157
Pestivirus A	KX170006	VIPR_ALG4_1129879278	1a	C	V067	2006	-0.1695	0.5724	1.1157
Pestivirus A	KX170007	VIPR_ALG4_1129879280	1a	C	V073	2011	-0.1695	0.5724	1.1157
Pestivirus A	KX170008	VIPR_ALG4_1129879282	1a	C	V074	2010	-0.1695	0.5724	1.1157

Species according to VIPRBRC	GenBank Accession	GenBank Protein Accession	Subgenotype	Protein	Strain Name	Collection Year	SVM Patho. Score	Vaxijen Antig. Score	Averged score of EMBOSS motifs
Pestivirus A	KX170009	VIPR_ALG4_1129879284_	1a	C	V077	2012	-0.1695	0.5724	1.1157
Pestivirus A	KX170010	VIPR_ALG4_1129879286_	1a	C	V080	2009	-0.1695	0.5724	1.1157
Pestivirus A	KX170011	VIPR_ALG4_1129879288_	1a	C	V091	2003	-0.3283	0.5890	1.1260
Pestivirus A	KX170012	VIPR_ALG4_1129879290_	1a	C	V083	2008	-0.2767	0.5896	1.1060
Pestivirus A	KX170013	VIPR_ALG4_1129879292_	1a	C	V016	2002	-0.5779	0.6294	1.1605
Pestivirus A	KX170014	VIPR_ALG4_1129879294_	1a	C	V001	1999	-0.6290	0.6133	1.1397
Pestivirus A	KX170015	VIPR_ALG4_1129879296_	1a	C	V039	2008	-0.6290	0.6133	1.1397
Pestivirus A	KX170016	VIPR_ALG4_1129879298_	1a	C	V041	2008	-0.6290	0.6133	1.1397
Pestivirus A	KX170017	VIPR_ALG4_1129879300_	1a	C	V050	2009	-0.6290	0.6133	1.1397
Pestivirus A	KX170018	VIPR_ALG4_1129879302_	1a	C	V052	2010	-0.6290	0.6133	1.1397
Pestivirus A	KX170019	VIPR_ALG4_1129879304_	1a	C	V035	2007	-0.6290	0.6133	1.1397
Pestivirus A	KX170020	VIPR_ALG4_1129879306_	1a	C	V040	2008	-0.6290	0.6133	1.1397
Pestivirus A	KX170021	VIPR_ALG4_1129879308_	1a	C	V042	2008	-0.6290	0.6133	1.1397
Pestivirus A	KX170022	VIPR_ALG4_1129879310_	1a	C	V043	2008	-0.6290	0.6133	1.1397
Pestivirus A	KX170023	VIPR_ALG4_1129879312_	1a	C	V046	2009	-0.6290	0.6133	1.1397
Pestivirus A	KX170024	VIPR_ALG4_1129879314_	1a	C	V010	2001	-0.6290	0.6133	1.1397
Pestivirus A	KX170025	VIPR_ALG4_1129879316_	1a	C	V008	2000	-0.6290	0.6133	1.1397
Pestivirus A	KX170026	VIPR_ALG4_1129879318_	1a	C	V009	2000	-0.6290	0.6133	1.1397
Pestivirus A	KX170027	VIPR_ALG4_1129879320_	1a	C	V048	2009	-0.5922	0.5674	1.1470
Pestivirus A	KX170028	VIPR_ALG4_1129879322_	1a	C	V059	2004	-0.5922	0.5674	1.1470
Pestivirus A	KX170029	VIPR_ALG4_1129879324_	1a	C	V099	1998	-0.3054	0.5474	1.1157
Pestivirus A	KX170030	VIPR_ALG4_1129879326_	1a	C	V014	2001	-0.5139	0.6311	1.1260
Pestivirus A	KX170031	VIPR_ALG4_1129879328_	1a	C	V022	2006	-0.3799	0.5918	1.1370
Pestivirus A	KX170032	VIPR_ALG4_1129879330_	1a	C	V054	2013	-0.2130	0.5884	1.1260
Pestivirus A	KX170033	VIPR_ALG4_1129879332_	1a	C	V092	2004	-0.4172	0.5840	1.1260
Pestivirus A	KX170034	VIPR_ALG4_1129879334_	1a	C	V011	2001	-0.0819	0.5587	1.1260
Pestivirus A	KX170035	VIPR_ALG4_1129879336_	1a	C	V012	2001	-0.0819	0.5587	1.1260
Pestivirus A	KX170036	VIPR_ALG4_1129879338_	1a	C	V056	2009	-0.2130	0.5884	1.1260
Pestivirus A	KX170037	VIPR_ALG4_1129879340_	1a	C	V057	2009	-0.2285	0.6170	1.1260
Pestivirus A	KX170038	VIPR_ALG4_1129879342_	1a	C	V006	2000	-0.1348	0.5987	1.1260
Pestivirus A	EF101530	VIPR_ALG4_118498779_8	1b	C	KE9	2007	-0.1752	0.5955	1.1660
Pestivirus A	DQ088995	VIPR_ALG4_145309048_8	1a	C	Singer_Arg	1974	-0.1695	0.5724	1.1157
Pestivirus A	U63479	VIPR_ALG4_1518836_873	1b	C	CP7	1987	0.0657	0.6236	1.1327
Pestivirus A	U86600	VIPR_ALG4_2149469_889	1b	C	ILLNC	1991	-0.4261	0.5730	1.1480
Pestivirus A	AF041040	VIPR_ALG4_2789677_874	1a	C	Oregon	1960	-0.5922	0.5674	1.1470
Pestivirus A	M96751	VIPR_ALG4_289508_890_	1a	C	UNKNOWN-M96751	1992	-0.6290	0.6133	1.1397
Pestivirus A	GU991550	VIPR_ALG4_293338762_4	1b	C	BSU1	2008	-0.1742	0.5602	1.1820
Pestivirus A	HQ174292	VIPR_ALG4_323145267_5	1a	C	180	2010	-0.5404	0.6769	1.1097
Pestivirus A	M31182	VIPR_ALG4_323206_890_	1a	C	UNKNOWN-M31182	1988	-0.7906	0.5577	1.1250
Pestivirus A	M96687	VIPR_ALG4_323230_888_	1b	C	Osloss	1967	0.3053	0.5719	1.1825
Pestivirus A	JN400273	VIPR_ALG4_363990275_8	1q	C	SD0803	2008	0.0387	0.6389	1.1485
Pestivirus A	AF091605	VIPR_ALG4_3661566_890	1a	C	Oregon C24V	1960	-0.5922	0.5674	1.1470
Pestivirus A	JN644055	VIPR_ALG4_373939303_5	1b	C	3156	2011	0.3026	0.5245	1.1820
Pestivirus A	JN380080	VIPR_ALG4_378753653_5	1a	C	6010	2010	-0.5404	0.6769	1.1097
Pestivirus A	JQ799141	VIPR_ALG4_390132765_7	1u	C	M31182	2010	0.2002	0.4787	1.1565
Pestivirus A	JX419397	VIPR_ALG4_404363562_7	1b	C	UNKNOWN-JX419397	2008	0.6039	0.6118	1.1725
Pestivirus A	JX419398	VIPR_ALG4_404363564_7	1b	C	UNKNOWN-JX419398	2008	0.6039	0.6118	1.1725

Species according to VIPRBRC	GenBank Accession	GenBank Protein Accession	Subgenotype	Protein	Strain Name	Collection Year	SVM Patho. Score	Vaxijen Antig. Score	Averged score of EMBOSS motifs
Pestivirus A	AF526381	VIPR_ALG4_42476348_83	1m	C	ZM-95	1995	-0.4334	0.6314	1.1350
Pestivirus A	JX297512	VIPR_ALG4_459284067_5	1b	C	10270	2007	-0.0007	0.5835	1.1770
Pestivirus A	JX297513	VIPR_ALG4_459284069_5	1b	C	Aries	2005	-0.0007	0.5835	1.1770
Pestivirus A	JX297514	VIPR_ALG4_459284071_5	1b	C	Columba	2005	-0.0007	0.5835	1.1770
Pestivirus A	JX297515	VIPR_ALG4_459284073_5	1b	C	Corona	2005	-0.0558	0.5864	1.1770
Pestivirus A	JX297516	VIPR_ALG4_459284075_5	1b	C	Gemini	2005	0.2106	0.6062	1.1770
Pestivirus A	JX297517	VIPR_ALG4_459284077_5	1b	C	Hercules	2006	0.3473	0.6235	1.1760
Pestivirus A	JX297518	VIPR_ALG4_459284079_5	1b	C	Leo	2006	-0.0007	0.5835	1.1770
Pestivirus A	JX297519	VIPR_ALG4_459284081_5	1b	C	Lyra	2006	-0.0007	0.5835	1.1770
Pestivirus A	JX297520	VIPR_ALG4_459284083_5	1b	C	Mars	2006	-0.0007	0.5835	1.1770
Pestivirus A	JX297521	VIPR_ALG4_459284085_5	1b	C	Scorpius	2006	-0.0007	0.5835	1.1770
Pestivirus A	JX306011	VIPR_ALG4_459284087_5	1b	C	Cepheus	2005	-0.0007	0.5835	1.1770
Pestivirus A	JX306012	VIPR_ALG4_459284089_5	1b	C	Hamal	2006	-0.0007	0.5835	1.1770
Pestivirus A	JX306013	VIPR_ALG4_459284091_5	1b	C	Kurhah	2006	-0.0007	0.5835	1.1770
Pestivirus A	JX306014	VIPR_ALG4_459284093_5	1b	C	Naos	2006	-0.0007	0.5835	1.1770
Pestivirus A	KC853440	VIPR_ALG4_507144146_8	1k	C	SuwaNcp	1993	-0.1189	0.5879	1.1545
Pestivirus A	KC853441	VIPR_ALG4_507144148_8	1k	C	SuwaCp	1993	-0.1189	0.5879	1.1545
Pestivirus A	KC695810	VIPR_ALG4_507866685_7	1q	C	camel-6	2010	0.1070	0.6411	1.1380
Pestivirus A	KC695811	VIPR_ALG4_507866687_7	1q	C	GS-3	2012	-0.0342	0.6248	1.1495
Pestivirus A	KC695812	VIPR_ALG4_507866689_5	1q	C	HB-1	2012	0.0648	0.7121	1.1485
Pestivirus A	KC695813	VIPR_ALG4_507866693_7	1c	C	Bega-like	2012	-0.2394	0.6413	1.1360
Pestivirus A	KC695814	VIPR_ALG4_507866704_8	1b	C	Av69 VEDEVAC	2011	0.2747	0.5827	1.1820
Pestivirus A	KC695815	VIPR_ALG4_507866706_7	1a	C	Av69 SD-1	2011	-0.6380	0.6151	1.1397
Pestivirus A	KC695816	VIPR_ALG4_507866709_7	1d	C	cell-con-1	2012	-0.0926	0.5582	1.1595
Pestivirus A	KC700344	VIPR_ALG4_508083101_5	1b	C	GS-4	2012	0.0915	0.6250	1.1725
Pestivirus A	KC757383	VIPR_ALG4_511775165_8	1d	C	10JJ-SKR	2010	-0.4983	0.5949	1.1595
Pestivirus A	KC963967	VIPR_ALG4_530291194_8	1b	C	12F004	2012	0.2612	0.6046	1.1725
Pestivirus A	KF772785	VIPR_ALG4_575471151_8	1b	C	CC13B	2013	0.0765	0.6164	1.1725
Pestivirus A	KF896608	VIPR_ALG4_586616532_7	1c	C	Bega-like	2012	-0.2394	0.6413	1.1360
Pestivirus A	KF835697	VIPR_ALG4_597437474_7	1b	C	AU526	2013	0.5303	0.5860	1.1730
Pestivirus A	KJ541471	VIPR_ALG4_633265982_7	1a	C	GS5	2013	-0.3642	0.6055	1.1133
Pestivirus A	KJ689448	VIPR_ALG4_635172915_8	1b	C	GX4	2012	0.2747	0.5827	1.1820
Pestivirus A	KF501393	VIPR_ALG4_669206614_8	1b	C	BVDV JL-1	2009	0.1359	0.5831	1.1710
Pestivirus A	AJ133738	VIPR_ALG4_7960754_89	1a	C	type 1	1963	-0.7906	0.5577	1.1250
Pestivirus A	KP941581	VIPR_ALG4_800924313_8	1b	C	USMARC-51998	2014	0.0314	0.5931	1.1725
Pestivirus A	KP941583	VIPR_ALG4_800924317_8	1b	C	USMARC-53874	2014	0.0010	0.5303	1.1725
Pestivirus A	KP941584	VIPR_ALG4_800924319_8	1a	C	USMARC-53875	2014	-0.3886	0.5586	1.1323
Pestivirus A	KP941586	VIPR_ALG4_800924323_8	1a	C	USMARC-55477	2014	-0.0814	0.5439	1.1103
Pestivirus A	KP941587	VIPR_ALG4_800924325_8	1b	C	USMARC-55478	2014	0.0928	0.5943	1.1725
Pestivirus A	KP941588	VIPR_ALG4_800924327_8	1b	C	USMARC-55922	2014	0.4228	0.5951	1.1725
Pestivirus A	KP941589	VIPR_ALG4_800924329_8	1b	C	USMARC-55923	2014	0.1650	0.6126	1.1745
Pestivirus A	KP941590	VIPR_ALG4_800924331_8	1b	C	USMARC-55924	2014	0.1099	0.6068	1.1610
Pestivirus A	KP941591	VIPR_ALG4_800924333_8	1b	C	USMARC-55925	2014	0.1194	0.5885	1.1825
Pestivirus A	KP941592	VIPR_ALG4_800924335_8	1b	C	USMARC-55926	2014	0.7244	0.6189	1.1725
Pestivirus A	KP313732	VIPR_ALG4_816850387_8	1e	C	Carlito	2014	-0.2216	0.5178	1.1660
Pestivirus A	KR013753	VIPR_ALG4_871332680_5	1a	C	WAX-N	1992	-0.2197	0.6997	1.1207
Pestivirus A	KR029825	VIPR_ALG4_887497286_8	1b	C	Egy/Ismailia/2014	2014	0.6460	0.6091	1.1725

Species according to VIPRBRC	GenBank Accession	GenBank Protein Accession	Subgenotype	Protein	Strain Name	Collection Year	SVM Patho. Score	Vaxijen Antig. Score	Averged score of EMBOSS motifs
Pestivirus A	LC089875	VIPR_ALG4_939106262_8	1o	C	IS26/01ncp	2001	-0.0683	0.6701	1.1515
Pestivirus A	LC089876	VIPR_ALG4_939106264_8	1n	C	Shitara/02/06	2006	-0.3198	0.5475	1.2050
Pestivirus A	KR866116	VIPR_ALG4_941508008_8	1m	C	SD-15	2015	-0.2113	0.6662	1.1420
Pestivirus A	KU200260	VIPR_ALG4_972905813_8	1b	C	BE/061536/2014	2014	0.1942	0.5773	1.1725
Pestivirus A	KX577637	VIPR_ALG4_AOR50934_1	1e	C	SLO/2407/2006	2006	0.3692	0.5579	1.1660
Pestivirus A	KX987157	VIPR_ALG4_APG30987_1	1f	C	SLO/1170/2000	2000	-0.3490	0.6577	1.1540
Pestivirus A	KX857724	VIPR_ALG4_APZ85839_1	1i	C	ACM/BR/2016	2016	-0.5109	0.5149	1.1260
Pestivirus A	KY849592	VIPR_ALG4_ART90617_1	1d	C	SLO/2416/2002	2002	-0.0361	0.5883	1.1425
Pestivirus A	MF278651	VIPR_ALG4_ASW18434_1	1b	C	XZ01	2016	0.2747	0.5827	1.1820
Pestivirus A	MF278652	VIPR_ALG4_ASW18435_1	1b	C	XZ02	2016	0.2747	0.5827	1.1820
Pestivirus A	MF693403	VIPR_ALG4_ATG71375_1	1a	C	UNKNOWN-MF693403	2016	0.1410	0.5989	1.1470
Pestivirus A	KY964311	VIPR_ALG4_ATN39078_1	1b	C	Y2	2014	0.1677	0.5710	1.1680
Pestivirus A	MF172980	VIPR_ALG4_AVI10261_1	1c	C	GSTZ	2012	-0.0352	0.5890	1.1550
Pestivirus A	MH379638	VIPR_ALG4_AWW14171_	1a	C	Ho916	1993	-0.1359	0.6226	1.1660
Pestivirus A	MG950344	VIPR_ALG4_AWW87346_	1b	C	AU526	2014	0.5303	0.5860	1.1730
Pestivirus A	MG950345	VIPR_ALG4_AWW87347_	1b	C	B1	2015	0.5303	0.5860	1.1730
Pestivirus A	MG950346	VIPR_ALG4_AWW87348_	1b	C	B2	2015	0.5303	0.5860	1.1730
Pestivirus A	MG950347	VIPR_ALG4_AWW87349_	1b	C	B3	2015	0.5303	0.5860	1.1730
Pestivirus A	MG950348	VIPR_ALG4_AWW87350_	1b	C	B4	2015	0.5303	0.5860	1.1730
Pestivirus A	MG950349	VIPR_ALG4_AWW87351_	1b	C	B5	2015	0.5303	0.5860	1.1730
Pestivirus A	MG950350	VIPR_ALG4_AWW87352_	1b	C	B6	2015	0.5303	0.5860	1.1730
Pestivirus A	MG950351	VIPR_ALG4_AWW87353_	1b	C	O1	2015	0.5303	0.5860	1.1730
Pestivirus A	MG950352	VIPR_ALG4_AWW87354_	1b	C	O2	2015	0.5303	0.5860	1.1730
Pestivirus A	MG950353	VIPR_ALG4_AWW87355_	1b	C	O3	2015	0.5303	0.5860	1.1730
Pestivirus A	MG950354	VIPR_ALG4_AWW87356_	1b	C	O4	2015	0.5303	0.5860	1.1730
Pestivirus A	MG950355	VIPR_ALG4_AWW87357_	1b	C	O5	2015	0.5303	0.5860	1.1730
Pestivirus A	MG950356	VIPR_ALG4_AWW87358_	1b	C	O6	2015	0.5303	0.5860	1.1730
Pestivirus A	MG950357	VIPR_ALG4_AWW87359_	1b	C	B1A	2015	0.5303	0.5860	1.1730
Pestivirus A	MG950358	VIPR_ALG4_AWW87360_	1b	C	B2A	2016	0.5303	0.5860	1.1730
Pestivirus A	MG950359	VIPR_ALG4_AWW87361_	1b	C	B3A	2016	0.5303	0.5860	1.1730
Pestivirus A	MG950360	VIPR_ALG4_AWW87362_	1b	C	B4A	2016	0.5303	0.5860	1.1730
Pestivirus A	MG950361	VIPR_ALG4_AWW87363_	1b	C	B5A	2016	0.5303	0.5860	1.1730
Pestivirus A	MG950362	VIPR_ALG4_AWW87364_	1b	C	B6A	2016	0.5303	0.5860	1.1730
Pestivirus A	MG950363	VIPR_ALG4_AWW87365_	1b	C	O1A	2015	0.5303	0.5860	1.1730
Pestivirus A	MG950364	VIPR_ALG4_AWW87366_	1b	C	O2A	2015	0.5303	0.5860	1.1730
Pestivirus A	MG950365	VIPR_ALG4_AWW87367_	1b	C	O2B	2015	0.3458	0.5892	1.1730
Pestivirus A	MG950366	VIPR_ALG4_AWW87368_	1b	C	O4A	2015	0.5303	0.5860	1.1730
Pestivirus A	MH311874	VIPR_ALG4_AWW87369_	1b	C	B2A d168	2016	0.5303	0.5860	1.1730
Pestivirus A	MH311875	VIPR_ALG4_AWW87370_	1b	C	B3A d168	2016	0.5303	0.5860	1.1730
Pestivirus A	MH311876	VIPR_ALG4_AWW87371_	1b	C	B4A d84	2016	0.5303	0.5860	1.1730
Pestivirus A	MH311877	VIPR_ALG4_AWW87372_	1b	C	B4A d168	2016	0.5303	0.5860	1.1730
Pestivirus A	MH311878	VIPR_ALG4_AWW87373_	1b	C	B5A d84	2016	0.5303	0.5860	1.1730
Pestivirus A	MH311879	VIPR_ALG4_AWW87374_	1b	C	B5A d168	2016	0.5303	0.5860	1.1730
Pestivirus A	MH311880	VIPR_ALG4_AWW87375_	1b	C	B6A d84	2016	0.5303	0.5860	1.1730
Pestivirus A	MH311881	VIPR_ALG4_AWW87376_	1b	C	B6A d168	2016	0.5303	0.5860	1.1730
Pestivirus A	MH379221	VIPR_ALG4_AWW87377_	1b	C	P1	2017	0.5303	0.5860	1.1730
Pestivirus A	MH379222	VIPR_ALG4_AWW87378_	1b	C	P2	2017	0.5303	0.5860	1.1730

Species according to VIPRBRC	GenBank Accession	GenBank Protein Accession	Subgenotype	Protein	Strain Name	Collection Year	SVM Patho. Score	Vaxijen Antig. Score	Averged score of EMBOSS motifs
Pestivirus A	MH379223	VIPR_ALG4_AWW87379_	1b	C	P5	2017	0.5303	0.5860	1.1730
Pestivirus A	MH379224	VIPR_ALG4_AWW87380_	1b	C	P6	2017	0.5303	0.5860	1.1730
Pestivirus A	MH379225	VIPR_ALG4_AWW87381_	1b	C	P7	2017	0.5303	0.5860	1.1730
Pestivirus A	MH379226	VIPR_ALG4_AWW87382_	1b	C	P5A	2017	0.5303	0.5860	1.1730
Pestivirus A	MH379227	VIPR_ALG4_AWW87383_	1b	C	P5B	2017	0.5303	0.5860	1.1730
Pestivirus A	MH379228	VIPR_ALG4_AWW87384_	1b	C	P5C	2017	0.5303	0.5860	1.1730
Pestivirus A	MH379229	VIPR_ALG4_AWW87385_	1b	C	P5D	2017	0.5303	0.5860	1.1730
Pestivirus A	MH379230	VIPR_ALG4_AWW87386_	1b	C	P5F	2017	0.5303	0.5860	1.1730
Pestivirus A	MH379231	VIPR_ALG4_AWW87387_	1b	C	P7A	2018	0.5303	0.5860	1.1730
Pestivirus A	MH379232	VIPR_ALG4_AWW87388_	1b	C	P7C	2018	0.5303	0.5860	1.1730
Pestivirus A	MH379233	VIPR_ALG4_AWW87389_	1b	C	P7E	2018	0.5303	0.5860	1.1730
Pestivirus A	MH379234	VIPR_ALG4_AWW87390_	1b	C	P7F	2018	0.7390	0.5903	1.1730
Pestivirus A	MH166806	VIPR_ALG4_AYA62524_1	1m	C	XC	2015	-0.1180	0.6763	1.1520
Pestivirus A	MH490943	VIPR_ALG4_AZB53078_1	1b	C	BVDV BJ-2016	2016	0.1140	0.6020	1.1865
Pestivirus A	MH231153	VIPR_ALG4_AZQ00677_1	1b	C	Nebraska	1990	0.2612	0.6046	1.1725
Pestivirus A	AB078950	VIPR_ALG4_BAC55961_1	1j	C	KS86-1ncp	1986	-0.1993	0.5849	1.1650
Pestivirus A	MH899941	VIPR_ALG4_QCE30388_1	1b	C	SLO/3301/2014	2014	0.1156	0.5922	1.1725
Pestivirus A	MH899942	VIPR_ALG4_QCE30389_1	1e	C	SLO/33529/2015	2015	0.2124	0.5470	1.1660
Pestivirus A	MH899943	VIPR_ALG4_QCE30390_1	1f	C	SLO/1361/2014	2014	-0.6817	0.6676	1.1380
Pestivirus A	MH899944	VIPR_ALG4_QCE30391_1	1f	C	SLO/28537/2017	2017	0.0366	0.7354	1.1380
Pestivirus A	MH899945	VIPR_ALG4_QCE30392_1	1h	C	SLO/1883/2013	2013	-0.0607	0.6068	1.1357
Pestivirus A	MK102095	VIPR_ALG4_QCQ84262_1	1q	C	20170226	2017	-0.2524	0.6006	1.1485
Pestivirus A	MK509774	VIPR_ALG4_QEK23510_1	1b	C	BVD1b-JH	2008	0.1268	0.6280	1.1770
Pestivirus A	MK775204	VIPR_ALG4_QFX66041_1	1i	C	CA2006	2006	-0.1135	0.5033	1.1370
Pestivirus A	MN188073	VIPR_ALG4_QGZ19414_1	1a	C	PI34	2017	-1.2911	0.6434	1.1700
Pestivirus A	MN188074	VIPR_ALG4_QGZ19415_1	1b	C	PI285	2017	0.1843	0.5911	1.1610
Pestivirus A	MN394766	VIPR_ALG4_QIB02049_1	1m	C	0001	2016	-0.0121	0.6400	1.1350
Pestivirus A	MT079816	VIPR_ALG4_QIM55913_1	1c	C	GXNN1	2018	0.1073	0.5099	1.1490
Pestivirus A	MN623291	VIPR_ALG4_QLL27013_1	1m	C	NX2019/01	2019	-0.1989	0.6416	1.1585
Pestivirus A	MW014286	VIPR_ALG4_QPJ59878_1	1b	C	GXSS01	2018	0.2405	0.5956	1.1820
Pestivirus A	MW014287	VIPR_ALG4_QPJ59879_1	1b	C	GXSS02	2018	0.1795	0.5532	1.1820
Pestivirus A	MW014288	VIPR_ALG4_QPJ59880_1	1b	C	GXSS03	2018	0.1795	0.5532	1.1820
Pestivirus A	MT024562	VIPR_ALG4_QPK41175_1	1b	C	190919	2019	-0.4656	0.5620	1.1660
Pestivirus A	MT024563	VIPR_ALG4_QPK41176_1	1b	C	230919	2019	-0.4237	0.5978	1.1660
Pestivirus A	MT024564	VIPR_ALG4_QPK41177_1	1a	C	ABART-2	2018	-0.1695	0.5724	1.1157
Pestivirus A	MT977117	VIPR_ALG4_QRZ20359_1	1b	C	BVDV 1b IT16/5	2016	0.1362	0.6081	1.1820
Pestivirus A	MT977118	VIPR_ALG4_QRZ20360_1	1b	C	BVDV 1b IT16/439	2016	0.1362	0.6081	1.1820
Pestivirus A	MT654137	VIPR_ALG4_QVK82311_1	1a	C	20-8536	2020	-0.3454	0.5673	1.1133
Pestivirus A	LT837585	VIPR_ALG4_SLV80196_1	1r	C	UNKNOWN-LT837585	2012	0.2615	0.7032	1.1490
Pestivirus A	MW054933	VIPR_ALG4_UEC94252_1	1f	C	LA/230/14	2014	-0.3167	0.6905	1.1360
Pestivirus A	MW054934	VIPR_ALG4_UEC94253_1	1f	C	LA/87/05	2005	-0.5894	0.6604	1.1380
Pestivirus A	MW054935	VIPR_ALG4_UEC94254_1	1k	C	TO/197/11	2011	-0.2213	0.5801	1.1360
Pestivirus A	MW054936	VIPR_ALG4_UEC94255_1	1g	C	UM/111/06	2006	0.1967	0.6148	1.1545
Pestivirus A	MW054937	VIPR_ALG4_UEC94256_1	1k	C	SA/158/09	2009	-0.1189	0.5879	1.1545
Pestivirus A	MW054938	VIPR_ALG4_UEC94257_1	1k	C	SA/159/09	2009	-0.1189	0.5879	1.1545
Pestivirus A	MW054939	VIPR_ALG4_UEC94258_1	1f	C	LO/151/09	2009	-0.3167	0.6905	1.1360
Pestivirus A	MW054940	VIPR_ALG4_UEC94259_1	1e	C	MA/101/05	2005	0.1137	0.5398	1.1660

Species according to VIPRBRC	GenBank Accession	GenBank Protein Accession	Subgenotype	Protein	Strain Name	Collection Year	SVM Patho. Score	Vaxijen Antig. Score	Averged score of EMBOSS motifs
Pestivirus A	MW250796	VIPR_ALG4_UEC94260_1	1i	C	58-1	2008	-0.2370	0.5272	1.1103
Pestivirus A	MW250797	VIPR_ALG4_UEC94261_1	1i	C	58-2	2008	-0.2370	0.5272	1.1103
Pestivirus A	MW250798	VIPR_ALG4_UEC94262_1	1a	C	62-2	2008	-0.3003	0.5649	1.1060
Pestivirus A	MW250799	VIPR_ALG4_UEC94263_1	1a	C	63-1	2008	-0.3293	0.5967	1.1060
Pestivirus A	MW250800	VIPR_ALG4_UEC94264_1	1d	C	67-1	2008	0.0370	0.6221	1.1825
Pestivirus A	MW250801	VIPR_ALG4_UEC94265_1	1d	C	67-2	2008	0.0370	0.6221	1.1825
Pestivirus A	MW250802	VIPR_ALG4_UEC94266_1	1e	C	68-1	2008	-0.3489	0.5418	1.1385
Pestivirus A	MW250803	VIPR_ALG4_UEC94267_1	1i	C	69-1	2008	-0.2370	0.5272	1.1103
Pestivirus A	MW655625	VIPR_ALG4_UEC94268_1	1h	C	CH-04-01b	2004	0.2378	0.5817	1.1430
Pestivirus A	MW655626	VIPR_ALG4_UEC94269_1	1e	C	Maria	2004	-0.4704	0.5898	1.1490
Pestivirus A	MW655627	VIPR_ALG4_UEC94270_1	1e	C	R2000-95	1995	0.2083	0.5582	1.1660
Pestivirus A	MW655628	VIPR_ALG4_UEC94271_1	1k	C	R3230-95	1995	-0.5512	0.5186	1.1605
Pestivirus A	MW655629	VIPR_ALG4_UEC94272_1	1h	C	R3572-90	1990	0.3335	0.5883	1.1655
Pestivirus A	MW655630	VIPR_ALG4_UEC94273_1	1k	C	R5013-96	1996	0.1533	0.5777	1.1545
Pestivirus A	MW655631	VIPR_ALG4_UEC94274_1	1e	C	S03-1175	2003	0.4565	0.5057	1.1203
Pestivirus A	MW655632	VIPR_ALG4_UEC94275_1	1h	C	SM09-20	2002	0.2317	0.5897	1.1655
Pestivirus A	MW713361	VIPR_ALG4_UEC94276_1	1a	C	BoAEC1190	2007	-0.6013	0.5195	1.1253
Pestivirus A	MW713362	VIPR_ALG4_UEC94277_1	1b	C	PI819	2017	0.5303	0.5860	1.1730
Pestivirus A	MW732738	VIPR_ALG4_UEC94278_1	1a	C	PI407	2015	-0.3790	0.5724	1.1133
Pestivirus A	MW732739	VIPR_ALG4_UEC94279_1	1a	C	YandaSpl	1993	-0.3642	0.6055	1.1133
Pestivirus A	MZ188972	VIPR_ALG4_UML14262_1	1q	C	HB-1	2020	-0.0249	0.6295	1.1180
Pestivirus A	ON337882	VIPR_ALG4_USZ80113_1	1c	C	NM2103	2021	-0.2025	0.5962	1.1660
Pestivirus A	MW560180	UQW60629.1	1d	Erns	NX1	2017	-0.1684	0.5340	1.1040
Pestivirus A	MW560181	UQW60630.1	1q	Erns	NX2	2019	-0.2043	0.4911	1.1045
Pestivirus A	MW560183	UQW60632.1	1v	Erns	NX201902	2019	-0.1132	0.4990	1.1123
Pestivirus A	MW560184	UQW60633.1	1m	Erns	NX5	2019	-0.2463	0.5079	1.1173
Pestivirus A	MW560185	UQW60634.1	1m	Erns	NX6	2019	-0.1312	0.5003	1.1150
Pestivirus A	KU159365	VIPR_ALG4_1039262063_	1a	Erns	USII-S15	2015	-0.4717	0.5313	1.0997
Pestivirus A	KU756226	VIPR_ALG4_1072900294_	1b	Erns	HJ-1	2010	-0.4966	0.5096	1.1134
Pestivirus A	KT943518	VIPR_ALG4_1093530908_	1d	Erns	BJ1201	2012	-0.2672	0.5312	1.1013
Pestivirus A	LT631725	VIPR_ALG4_1112914034_	1h	Erns	UM/126/07	2007	-0.1377	0.5109	1.1038
Pestivirus A	KX170223	VIPR_ALG4_1129879712_	1b	Erns	V015	2001	-0.5042	0.5096	1.1117
Pestivirus A	KX170224	VIPR_ALG4_1129879714_	1b	Erns	V060	2004	-0.3308	0.5129	1.1090
Pestivirus A	KX170225	VIPR_ALG4_1129879716_	1b	Erns	V075	2011	-0.5652	0.5281	1.1141
Pestivirus A	KX170226	VIPR_ALG4_1129879718_	1b	Erns	V036	2007	-0.2762	0.5015	1.1091
Pestivirus A	KX170227	VIPR_ALG4_1129879720_	1b	Erns	V098	1999	-0.5638	0.5039	1.1092
Pestivirus A	KX170228	VIPR_ALG4_1129879722_	1b	Erns	V100	1997	-0.3854	0.5036	1.1072
Pestivirus A	KX170229	VIPR_ALG4_1129879724_	1b	Erns	V078	2012	-0.4673	0.5230	1.1066
Pestivirus A	KX170230	VIPR_ALG4_1129879726_	1b	Erns	V070	2007	-0.5616	0.5184	1.1070
Pestivirus A	KX170231	VIPR_ALG4_1129879728_	1b	Erns	V020	2005	-0.4050	0.5070	1.1072
Pestivirus A	KX170232	VIPR_ALG4_1129879730_	1b	Erns	V029	2006	-0.4050	0.5070	1.1072
Pestivirus A	KX170233	VIPR_ALG4_1129879732_	1b	Erns	V031	2006	-0.1497	0.5234	1.1091
Pestivirus A	KX170234	VIPR_ALG4_1129879734_	1b	Erns	V045	2009	-0.3809	0.5154	1.1072
Pestivirus A	KX170235	VIPR_ALG4_1129879736_	1b	Erns	V087	2006	-0.3504	0.5159	1.1072
Pestivirus A	KX170236	VIPR_ALG4_1129879738_	1a	Erns	V092	2004	-0.4723	0.5379	1.1052
Pestivirus A	KX170237	VIPR_ALG4_1129879740_	1a	Erns	V014	2001	-0.3590	0.5153	1.1011
Pestivirus A	KX170238	VIPR_ALG4_1129879742_	1a	Erns	V022	2006	-0.4362	0.5319	1.0997

Species according to VIPRBRC	GenBank Accession	GenBank Protein Accession	Subgenotype	Protein	Strain Name	Collection Year	SVM Patho. Score	Vaxijen Antig. Score	Averged score of EMBOSS motifs
Pestivirus A	KX170239	VIPR_ALG4_1129879744_	1a	Erns	V054	2013	-0.4229	0.5352	1.1074
Pestivirus A	KX170240	VIPR_ALG4_1129879746_	1a	Erns	V057	2009	-0.4123	0.5347	1.0997
Pestivirus A	KX170241	VIPR_ALG4_1129879748_	1a	Erns	V011	2001	-0.4361	0.5371	1.1030
Pestivirus A	KX170242	VIPR_ALG4_1129879750_	1a	Erns	V012	2001	-0.4432	0.5328	1.1035
Pestivirus A	KX170243	VIPR_ALG4_1129879752_	1a	Erns	V006	2000	-0.5465	0.5398	1.0997
Pestivirus A	KX170244	VIPR_ALG4_1129879754_	1a	Erns	V056	2009	-0.4081	0.5372	1.0933
Pestivirus A	KX170245	VIPR_ALG4_1129879756_	1a	Erns	V016	2002	-0.5413	0.5302	1.1041
Pestivirus A	KX170246	VIPR_ALG4_1129879758_	1a	Erns	V001	1999	-0.4518	0.5143	1.1061
Pestivirus A	KX170247	VIPR_ALG4_1129879760_	1a	Erns	V010	2001	-0.5094	0.5277	1.1041
Pestivirus A	KX170248	VIPR_ALG4_1129879762_	1a	Erns	V008	2000	-0.5101	0.5248	1.1054
Pestivirus A	KX170249	VIPR_ALG4_1129879764_	1a	Erns	V009	2000	-0.5101	0.5248	1.1054
Pestivirus A	KX170250	VIPR_ALG4_1129879766_	1a	Erns	V042	2008	-0.5571	0.5481	1.1064
Pestivirus A	KX170251	VIPR_ALG4_1129879768_	1a	Erns	V043	2008	-0.5002	0.5473	1.1064
Pestivirus A	KX170252	VIPR_ALG4_1129879770_	1a	Erns	V050	2009	-0.6258	0.5206	1.1049
Pestivirus A	KX170253	VIPR_ALG4_1129879772_	1a	Erns	V035	2007	-0.6138	0.5576	1.1041
Pestivirus A	KX170254	VIPR_ALG4_1129879774_	1a	Erns	V039	2008	-0.6138	0.5576	1.1041
Pestivirus A	KX170255	VIPR_ALG4_1129879776_	1a	Erns	V040	2008	-0.6138	0.5576	1.1041
Pestivirus A	KX170256	VIPR_ALG4_1129879778_	1a	Erns	V041	2008	-0.6138	0.5576	1.1041
Pestivirus A	KX170257	VIPR_ALG4_1129879780_	1a	Erns	V046	2009	-0.6138	0.5576	1.1041
Pestivirus A	KX170258	VIPR_ALG4_1129879782_	1a	Erns	V052	2010	-0.6138	0.5576	1.1041
Pestivirus A	KX170259	VIPR_ALG4_1129879784_	1a	Erns	V026	2006	-0.2847	0.5268	1.1081
Pestivirus A	KX170260	VIPR_ALG4_1129879786_	1a	Erns	V027	2006	-0.2847	0.5268	1.1081
Pestivirus A	KX170261	VIPR_ALG4_1129879788_	1a	Erns	V091	2003	-0.4072	0.5173	1.1042
Pestivirus A	KX170262	VIPR_ALG4_1129879790_	1a	Erns	V034	2007	-0.4015	0.5252	1.1064
Pestivirus A	KX170263	VIPR_ALG4_1129879792_	1a	Erns	V067	2006	-0.4015	0.5252	1.1064
Pestivirus A	KX170264	VIPR_ALG4_1129879794_	1a	Erns	V007	2000	-0.4015	0.5252	1.1064
Pestivirus A	KX170265	VIPR_ALG4_1129879796_	1a	Erns	V013	2001	-0.4015	0.5252	1.1064
Pestivirus A	KX170266	VIPR_ALG4_1129879798_	1a	Erns	V033	2007	-0.4015	0.5252	1.1064
Pestivirus A	KX170267	VIPR_ALG4_1129879800_	1a	Erns	V049	2009	-0.4015	0.5252	1.1064
Pestivirus A	KX170268	VIPR_ALG4_1129879802_	1a	Erns	V073	2011	-0.4015	0.5252	1.1064
Pestivirus A	KX170269	VIPR_ALG4_1129879804_	1a	Erns	V074	2010	-0.4015	0.5252	1.1064
Pestivirus A	KX170270	VIPR_ALG4_1129879806_	1a	Erns	V077	2012	-0.4015	0.5252	1.1064
Pestivirus A	KX170271	VIPR_ALG4_1129879808_	1a	Erns	V080	2009	-0.4015	0.5252	1.1064
Pestivirus A	KX170272	VIPR_ALG4_1129879810_	1a	Erns	V083	2008	-0.3148	0.5233	1.1020
Pestivirus A	KX170273	VIPR_ALG4_1129879812_	1a	Erns	V099	1998	-0.5862	0.4859	1.1063
Pestivirus A	KX170274	VIPR_ALG4_1129879814_	1a	Erns	V048	2009	-0.5574	0.4720	1.0993
Pestivirus A	KX170275	VIPR_ALG4_1129879816_	1a	Erns	V059	2004	-0.5756	0.4791	1.0993
Pestivirus A	EF101530	VIPR_ALG4_118498779_1	1b	Erns	KE9	2007	-0.3828	0.4795	1.1092
Pestivirus A	DQ088995	VIPR_ALG4_145309048_1	1a	Erns	Singer_Arg	1974	-0.4378	0.5177	1.1064
Pestivirus A	U63479	VIPR_ALG4_1518836_117	1b	Erns	CP7	1987	-0.4383	0.4997	1.1072
Pestivirus A	U86600	VIPR_ALG4_2149469_119	1b	Erns	ILLNC	1991	-0.5025	0.5298	1.1149
Pestivirus A	FJ555203	VIPR_ALG4_221325922_1	1b	Erns	CCSYD	2004	-0.5209	0.4910	1.1134
Pestivirus A	AF041040	VIPR_ALG4_2789677_118	1a	Erns	Oregon	1960	-0.6195	0.4720	1.0993
Pestivirus A	M96751	VIPR_ALG4_289508_1196	1a	Erns	UNKNOWN-M96751	1992	-0.5877	0.5318	1.1082
Pestivirus A	HQ174292	VIPR_ALG4_323145267_8	1a	Erns	180	2010	-0.3755	0.5244	1.1016
Pestivirus A	M31182	VIPR_ALG4_323206_1196	1a	Erns	UNKNOWN-M31182	1988	-0.2911	0.5620	1.1081
Pestivirus A	M96687	VIPR_ALG4_323230_1194	1b	Erns	Osloss	1967	-0.4522	0.4677	1.1156

Species according to VIPRBRC	GenBank Accession	GenBank Protein Accession	Subgenotype	Protein	Strain Name	Collection Year	SVM Patho. Score	Vaxijen Antig. Score	Averged score of EMBOSS motifs
Pestivirus A	JF927789	VIPR_ALG4_342674152_1	1b	Erns	QHZK10	2010	-0.5113	0.5184	1.1058
Pestivirus A	JN400273	VIPR_ALG4_363990275_1	1q	Erns	SD0803	2008	-0.2998	0.5007	1.1046
Pestivirus A	AF091605	VIPR_ALG4_3661566_119	1a	Erns	Oregon C24V	1960	-0.6485	0.4763	1.0993
Pestivirus A	JN644055	VIPR_ALG4_373939303_8	1b	Erns	3156	2011	-0.4625	0.4794	1.1134
Pestivirus A	JN380080	VIPR_ALG4_378753653_8	1a	Erns	6010	2010	-0.3372	0.5335	1.0975
Pestivirus A	JQ799141	VIPR_ALG4_390132765_1	1u	Erns	M31182	2010	-0.0745	0.5095	1.1164
Pestivirus A	JX046799	VIPR_ALG4_398359628_1	1b	Erns	HB-DCZ	2011	-0.5335	0.5125	1.1121
Pestivirus A	JX419397	VIPR_ALG4_404363562_1	1b	Erns	UNKNOWN-JX419397	2008	-0.3564	0.5379	1.1070
Pestivirus A	JX419398	VIPR_ALG4_404363564_1	1b	Erns	UNKNOWN-JX419398	2008	-0.3564	0.5379	1.1070
Pestivirus A	AF526381	VIPR_ALG4_42476348_11	1m	Erns	ZM-95	1995	-0.2506	0.5122	1.1201
Pestivirus A	JX297512	VIPR_ALG4_459284067_8	1b	Erns	10270	2007	-0.5089	0.4623	1.1051
Pestivirus A	JX297513	VIPR_ALG4_459284069_8	1b	Erns	Aries	2005	-0.3872	0.4917	1.1103
Pestivirus A	JX297514	VIPR_ALG4_459284071_8	1b	Erns	Columba	2005	-0.4699	0.4443	1.1171
Pestivirus A	JX297515	VIPR_ALG4_459284073_8	1b	Erns	Corona	2005	-0.4549	0.4851	1.1103
Pestivirus A	JX297516	VIPR_ALG4_459284075_8	1b	Erns	Gemini	2005	-0.3546	0.5206	1.1124
Pestivirus A	JX297517	VIPR_ALG4_459284077_8	1b	Erns	Hercules	2006	-0.5157	0.4821	1.1077
Pestivirus A	JX297518	VIPR_ALG4_459284079_8	1b	Erns	Leo	2006	-0.4084	0.4574	1.1111
Pestivirus A	JX297519	VIPR_ALG4_459284081_8	1b	Erns	Lyra	2006	-0.4950	0.4827	1.1083
Pestivirus A	JX297520	VIPR_ALG4_459284083_8	1b	Erns	Mars	2006	-0.4606	0.4389	1.1071
Pestivirus A	JX297521	VIPR_ALG4_459284085_8	1b	Erns	Scorpius	2006	-0.4868	0.4821	1.1083
Pestivirus A	JX306011	VIPR_ALG4_459284087_8	1b	Erns	Cepheus	2005	-0.4549	0.4851	1.1103
Pestivirus A	JX306012	VIPR_ALG4_459284089_8	1b	Erns	Hamal	2006	-0.4697	0.4451	1.1103
Pestivirus A	JX306013	VIPR_ALG4_459284091_8	1b	Erns	Kurhah	2006	-0.4549	0.4851	1.1103
Pestivirus A	JX306014	VIPR_ALG4_459284093_8	1b	Erns	Naos	2006	-0.4455	0.4722	1.1071
Pestivirus A	KC853440	VIPR_ALG4_507144146_1	1k	Erns	SuwaNcp	1993	-0.3560	0.5296	1.1057
Pestivirus A	KC853441	VIPR_ALG4_507144148_1	1k	Erns	SuwaCp	1993	-0.3405	0.5644	1.1048
Pestivirus A	KC695810	VIPR_ALG4_507866685_1	1q	Erns	camel-6	2010	-0.1590	0.4994	1.1077
Pestivirus A	KC695811	VIPR_ALG4_507866687_1	1q	Erns	GS-3	2012	-0.4217	0.4449	1.1103
Pestivirus A	KC695812	VIPR_ALG4_507866689_8	1q	Erns	HB-1	2012	-0.3183	0.4984	1.1046
Pestivirus A	KC695813	VIPR_ALG4_507866693_1	1c	Erns	Bega-like	2012	-0.5217	0.5178	1.1056
Pestivirus A	KC695814	VIPR_ALG4_507866704_1	1b	Erns	Av69 VEDEVAC	2011	-0.5276	0.5063	1.1134
Pestivirus A	KC695815	VIPR_ALG4_507866706_1	1a	Erns	Av69 SD-1	2011	-0.4605	0.5035	1.1058
Pestivirus A	KC695816	VIPR_ALG4_507866709_1	1d	Erns	cell-con-1	2012	-0.2295	0.5381	1.0985
Pestivirus A	KC700344	VIPR_ALG4_508083101_8	1b	Erns	GS-4	2012	-0.4595	0.4920	1.1103
Pestivirus A	KC757383	VIPR_ALG4_511775165_1	1d	Erns	10JJ-SKR	2010	-0.3241	0.5319	1.1002
Pestivirus A	KC963967	VIPR_ALG4_530291194_1	1b	Erns	12F004	2012	-0.4083	0.4984	1.1131
Pestivirus A	KF772785	VIPR_ALG4_575471151_1	1b	Erns	CC13B	2013	-0.5253	0.5103	1.1065
Pestivirus A	KF896608	VIPR_ALG4_586616532_1	1c	Erns	Bega-like	2012	-0.5217	0.5178	1.1056
Pestivirus A	KF835697	VIPR_ALG4_597437474_1	1b	Erns	AU526	2013	-0.4767	0.4602	1.1081
Pestivirus A	KJ541471	VIPR_ALG4_633265982_1	1a	Erns	GS5	2013	-0.4298	0.5061	1.1075
Pestivirus A	KJ689448	VIPR_ALG4_635172915_1	1b	Erns	GX4	2012	-0.4966	0.5096	1.1134
Pestivirus A	KF501393	VIPR_ALG4_669206614_1	1b	Erns	BVDV JL-1	2009	-0.6824	0.5067	1.1085
Pestivirus A	AJ133738	VIPR_ALG4_7960754_119	1a	Erns	type 1	1963	-0.2911	0.5620	1.1081
Pestivirus A	KP941581	VIPR_ALG4_800924313_1	1b	Erns	USMARC-51998	2014	-0.6055	0.4894	1.1079
Pestivirus A	KP941583	VIPR_ALG4_800924317_1	1b	Erns	USMARC-53874	2014	-0.3277	0.4313	1.1049
Pestivirus A	KP941584	VIPR_ALG4_800924319_1	1a	Erns	USMARC-53875	2014	-0.4920	0.5055	1.1042
Pestivirus A	KP941586	VIPR_ALG4_800924323_1	1a	Erns	USMARC-55477	2014	-0.3055	0.5106	1.1055

Species according to VIPRBRC	GenBank Accession	GenBank Protein Accession	Subgenotype	Protein	Strain Name	Collection Year	SVM Patho. Score	Vaxijen Antig. Score	Averged score of EMBOSS motifs
Pestivirus A	KP941587	VIPR_ALG4_800924325_1	1b	Erns	USMARC-55478	2014	-0.2848	0.4690	1.1032
Pestivirus A	KP941588	VIPR_ALG4_800924327_1	1b	Erns	USMARC-55922	2014	-0.4035	0.5157	1.1100
Pestivirus A	KP941589	VIPR_ALG4_800924329_1	1b	Erns	USMARC-55923	2014	-0.4526	0.4400	1.1097
Pestivirus A	KP941590	VIPR_ALG4_800924331_1	1b	Erns	USMARC-55924	2014	-0.4672	0.4683	1.1125
Pestivirus A	KP941591	VIPR_ALG4_800924333_1	1b	Erns	USMARC-55925	2014	-0.6011	0.4993	1.1127
Pestivirus A	KP941592	VIPR_ALG4_800924335_1	1b	Erns	USMARC-55926	2014	-0.4249	0.5212	1.1056
Pestivirus A	KP313732	VIPR_ALG4_816850387_1	1e	Erns	Carlito	2014	-0.4563	0.5210	1.1117
Pestivirus A	KR013753	VIPR_ALG4_871332680_8	1a	Erns	WAX-N	1992	-0.3713	0.4771	1.1034
Pestivirus A	KR029825	VIPR_ALG4_887497286_1	1b	Erns	Egy/Ismailia/2014	2014	-0.4944	0.4520	1.1054
Pestivirus A	LC089875	VIPR_ALG4_939106262_1	1o	Erns	IS26/01ncp	2001	-0.0901	0.4653	1.1162
Pestivirus A	LC089876	VIPR_ALG4_939106264_1	1n	Erns	Shitara/02/06	2006	-0.6440	0.5139	1.1073
Pestivirus A	KR866116	VIPR_ALG4_941508008_1	1m	Erns	SD-15	2015	-0.2155	0.4561	1.1134
Pestivirus A	KU200260	VIPR_ALG4_972905813_1	1b	Erns	BE/061536/2014	2014	-0.3496	0.5156	1.1091
Pestivirus A	KX577637	VIPR_ALG4_AOR50934_1	1e	Erns	SLO/2407/2006	2006	-0.5869	0.4702	1.1114
Pestivirus A	KX987157	VIPR_ALG4_APG30987_1	1f	Erns	SLO/1170/2000	2000	-0.2507	0.5019	1.1037
Pestivirus A	KX857724	VIPR_ALG4_APZ85839_1	1i	Erns	ACM/BR/2016	2016	-0.0533	0.4477	1.1013
Pestivirus A	KY849592	VIPR_ALG4_ART90617_1	1d	Erns	SLO/2416/2002	2002	-0.3797	0.5326	1.1056
Pestivirus A	MF278651	VIPR_ALG4_ASW18434_1	1b	Erns	XZ01	2016	-0.5276	0.5063	1.1134
Pestivirus A	MF278652	VIPR_ALG4_ASW18435_1	1b	Erns	XZ02	2016	-0.4966	0.5096	1.1134
Pestivirus A	MF693403	VIPR_ALG4_ATG71375_1	1a	Erns	UNKNOWN-MF693403	2016	-0.5523	0.5097	1.0989
Pestivirus A	KY964311	VIPR_ALG4_ATN39078_1	1b	Erns	Y2	2014	-0.3599	0.4793	1.1022
Pestivirus A	KY675201	VIPR_ALG4_AUQ32831_1	1a	Erns	GS5	2013	-0.4298	0.5061	1.1075
Pestivirus A	KY675202	VIPR_ALG4_AUQ32832_1	1b	Erns	GS6	2013	-0.3221	0.4954	1.1145
Pestivirus A	KY675203	VIPR_ALG4_AUQ32833_1	1m	Erns	GS10	2013	-0.2429	0.5105	1.1146
Pestivirus A	KY675204	VIPR_ALG4_AUQ32834_1	1m	Erns	GS7	2013	-0.2037	0.4745	1.1156
Pestivirus A	KY675205	VIPR_ALG4_AUQ32835_1	1a	Erns	GS8	2013	-0.4303	0.4907	1.1098
Pestivirus A	KY675206	VIPR_ALG4_AUQ32836_1	1m	Erns	GS9	2014	-0.1124	0.4762	1.1133
Pestivirus A	KY675207	VIPR_ALG4_AUQ32837_1	1p	Erns	GS11	2014	-0.1816	0.4678	1.1172
Pestivirus A	KY675208	VIPR_ALG4_AUQ32838_1	1c	Erns	GS13	2014	-0.3637	0.5271	1.1084
Pestivirus A	KY675209	VIPR_ALG4_AUQ32839_1	1m	Erns	GS17	2014	-0.2742	0.5027	1.1233
Pestivirus A	KY675210	VIPR_ALG4_AUQ32840_1	1m	Erns	GS20	2014	-0.2534	0.4853	1.1156
Pestivirus A	KY675211	VIPR_ALG4_AUQ32841_1	1o	Erns	GS23	2015	-0.2887	0.4874	1.1149
Pestivirus A	KY675212	VIPR_ALG4_AUQ32842_1	1m	Erns	GS25	2015	-0.0820	0.5088	1.1070
Pestivirus A	KY675213	VIPR_ALG4_AUQ32843_1	1q	Erns	GS26	2015	-0.3078	0.5274	1.1085
Pestivirus A	KY675214	VIPR_ALG4_AUQ32844_1	1d	Erns	QH1	2015	-0.2295	0.5381	1.0985
Pestivirus A	KY675215	VIPR_ALG4_AUQ32845_1	1p	Erns	QH2	2015	-0.0484	0.4790	1.1062
Pestivirus A	KY675216	VIPR_ALG4_AUQ32846_1	1b	Erns	QH4	2016	-0.4264	0.5064	1.1103
Pestivirus A	KY675217	VIPR_ALG4_AUQ32847_1	1q	Erns	QH5	2016	-0.2231	0.4959	1.1054
Pestivirus A	KY675218	VIPR_ALG4_AUQ32848_1	1q	Erns	QH8	2016	-0.2671	0.4923	1.1109
Pestivirus A	KY675219	VIPR_ALG4_AUQ32849_1	1d	Erns	QH9	2016	-0.0063	0.5558	1.1102
Pestivirus A	KY675220	VIPR_ALG4_AUQ32850_1	1p	Erns	QH11	2016	-0.3998	0.5553	1.1174
Pestivirus A	KY675221	VIPR_ALG4_AUQ32851_1	1m	Erns	QH12	2016	-0.1284	0.5148	1.1159
Pestivirus A	KY675222	VIPR_ALG4_AUQ32852_1	1b	Erns	QH15	2016	-0.3932	0.5172	1.1109
Pestivirus A	KY675223	VIPR_ALG4_AUQ32853_1	1b	Erns	QH16	2016	-0.4166	0.4881	1.1122
Pestivirus A	KY675224	VIPR_ALG4_AUQ32854_1	1b	Erns	QH17	2016	-0.4191	0.5212	1.1109
Pestivirus A	KY675225	VIPR_ALG4_AUQ32855_1	1p	Erns	QH18	2016	0.0323	0.4905	1.1072
Pestivirus A	KY675226	VIPR_ALG4_AUQ32856_1	1m	Erns	QH20	2016	-0.3232	0.4999	1.1121

Species according to VIPRBRC	GenBank Accession	GenBank Protein Accession	Subgenotype	Protein	Strain Name	Collection Year	SVM Patho. Score	Vaxijen Antig. Score	Averged score of EMBOSS motifs
Pestivirus A	KY675227	VIPR_ALG4_AUQ32857_1	1m	Erns	QH23	2016	-0.2058	0.5102	1.1156
Pestivirus A	KY675228	VIPR_ALG4_AUQ32858_1	1p	Erns	Camel-5	2010	-0.0572	0.4623	1.1172
Pestivirus A	MF172980	VIPR_ALG4_AVI10261_1_	1c	Erns	GSTZ	2012	-0.4661	0.5434	1.1049
Pestivirus A	MH379638	VIPR_ALG4_AWW14171_	1a	Erns	Ho916	1993	-0.4214	0.5164	1.1019
Pestivirus A	MG950344	VIPR_ALG4_AWW87346_	1b	Erns	AU526	2014	-0.4383	0.4997	1.1072
Pestivirus A	MG950345	VIPR_ALG4_AWW87347_	1b	Erns	B1	2015	-0.4383	0.4997	1.1072
Pestivirus A	MG950346	VIPR_ALG4_AWW87348_	1b	Erns	B2	2015	-0.4451	0.4903	1.1098
Pestivirus A	MG950347	VIPR_ALG4_AWW87349_	1b	Erns	B3	2015	-0.4383	0.4997	1.1072
Pestivirus A	MG950348	VIPR_ALG4_AWW87350_	1b	Erns	B4	2015	-0.4383	0.4997	1.1072
Pestivirus A	MG950349	VIPR_ALG4_AWW87351_	1b	Erns	B5	2015	-0.3778	0.4959	1.1072
Pestivirus A	MG950350	VIPR_ALG4_AWW87352_	1b	Erns	B6	2015	-0.4383	0.4997	1.1072
Pestivirus A	MG950351	VIPR_ALG4_AWW87353_	1b	Erns	O1	2015	-0.4383	0.4997	1.1072
Pestivirus A	MG950352	VIPR_ALG4_AWW87354_	1b	Erns	O2	2015	-0.3834	0.4844	1.1070
Pestivirus A	MG950353	VIPR_ALG4_AWW87355_	1b	Erns	O3	2015	-0.4417	0.4886	1.1072
Pestivirus A	MG950354	VIPR_ALG4_AWW87356_	1b	Erns	O4	2015	-0.4417	0.4886	1.1072
Pestivirus A	MG950355	VIPR_ALG4_AWW87357_	1b	Erns	O5	2015	-0.4417	0.4886	1.1072
Pestivirus A	MG950356	VIPR_ALG4_AWW87358_	1b	Erns	O6	2015	-0.4417	0.4886	1.1072
Pestivirus A	MG950357	VIPR_ALG4_AWW87359_	1b	Erns	B1A	2015	-0.3897	0.4856	1.1072
Pestivirus A	MG950358	VIPR_ALG4_AWW87360_	1b	Erns	B2A	2016	-0.4383	0.4997	1.1072
Pestivirus A	MG950359	VIPR_ALG4_AWW87361_	1b	Erns	B3A	2016	-0.4383	0.4997	1.1072
Pestivirus A	MG950360	VIPR_ALG4_AWW87362_	1b	Erns	B4A	2016	-0.4383	0.4997	1.1072
Pestivirus A	MG950361	VIPR_ALG4_AWW87363_	1b	Erns	B5A	2016	-0.4383	0.4997	1.1072
Pestivirus A	MG950362	VIPR_ALG4_AWW87364_	1b	Erns	B6A	2016	-0.4383	0.4997	1.1072
Pestivirus A	MG950363	VIPR_ALG4_AWW87365_	1b	Erns	O1A	2015	-0.4088	0.4862	1.1070
Pestivirus A	MG950364	VIPR_ALG4_AWW87366_	1b	Erns	O2A	2015	-0.4417	0.4886	1.1072
Pestivirus A	MG950365	VIPR_ALG4_AWW87367_	1b	Erns	O2B	2015	-0.4541	0.4486	1.1072
Pestivirus A	MG950366	VIPR_ALG4_AWW87368_	1b	Erns	O4A	2015	-0.4417	0.4886	1.1072
Pestivirus A	MH311874	VIPR_ALG4_AWW87369_	1b	Erns	B2A d168	2016	-0.4383	0.4997	1.1072
Pestivirus A	MH311875	VIPR_ALG4_AWW87370_	1b	Erns	B3A d168	2016	-0.4383	0.4997	1.1072
Pestivirus A	MH311876	VIPR_ALG4_AWW87371_	1b	Erns	B4A d84	2016	-0.4383	0.4997	1.1072
Pestivirus A	MH311877	VIPR_ALG4_AWW87372_	1b	Erns	B4A d168	2016	-0.4383	0.4997	1.1072
Pestivirus A	MH311878	VIPR_ALG4_AWW87373_	1b	Erns	B5A d84	2016	-0.4383	0.4997	1.1072
Pestivirus A	MH311879	VIPR_ALG4_AWW87374_	1b	Erns	B5A d168	2016	-0.4383	0.4997	1.1072
Pestivirus A	MH311880	VIPR_ALG4_AWW87375_	1b	Erns	B6A d84	2016	-0.4383	0.4997	1.1072
Pestivirus A	MH311881	VIPR_ALG4_AWW87376_	1b	Erns	B6A d168	2016	-0.4383	0.4997	1.1072
Pestivirus A	MH379221	VIPR_ALG4_AWW87377_	1b	Erns	P1	2017	-0.4492	0.4832	1.1093
Pestivirus A	MH379222	VIPR_ALG4_AWW87378_	1b	Erns	P2	2017	-0.4492	0.4832	1.1093
Pestivirus A	MH379223	VIPR_ALG4_AWW87379_	1b	Erns	P5	2017	-0.4492	0.4832	1.1093
Pestivirus A	MH379224	VIPR_ALG4_AWW87380_	1b	Erns	P6	2017	-0.4492	0.4832	1.1093
Pestivirus A	MH379225	VIPR_ALG4_AWW87381_	1b	Erns	P7	2017	-0.4492	0.4832	1.1093
Pestivirus A	MH379226	VIPR_ALG4_AWW87382_	1b	Erns	P5A	2017	-0.4492	0.4832	1.1093
Pestivirus A	MH379227	VIPR_ALG4_AWW87383_	1b	Erns	P5B	2017	-0.4492	0.4832	1.1093
Pestivirus A	MH379228	VIPR_ALG4_AWW87384_	1b	Erns	P5C	2017	-0.4492	0.4832	1.1093
Pestivirus A	MH379229	VIPR_ALG4_AWW87385_	1b	Erns	P5D	2017	-0.4492	0.4832	1.1093
Pestivirus A	MH379230	VIPR_ALG4_AWW87386_	1b	Erns	P5F	2017	-0.4492	0.4832	1.1093
Pestivirus A	MH379231	VIPR_ALG4_AWW87387_	1b	Erns	P7A	2018	-0.4492	0.4832	1.1093
Pestivirus A	MH379232	VIPR_ALG4_AWW87388_	1b	Erns	P7C	2018	-0.4492	0.4832	1.1093

Species according to VIPRBRC	GenBank Accession	GenBank Protein Accession	Subgenotype	Protein	Strain Name	Collection Year	SVM Patho. Score	Vaxijen Antig. Score	Averged score of EMBOSS motifs
Pestivirus A	MH379233	VIPR_ALG4_AWW87389_	1b	Erns	P7E	2018	-0.4612	0.4432	1.1093
Pestivirus A	MH379234	VIPR_ALG4_AWW87390_	1b	Erns	P7F	2018	-0.4612	0.4432	1.1093
Pestivirus A	MH166806	VIPR_ALG4_AYA62524_1	1m	Erns	XC	2015	-0.2249	0.4881	1.1156
Pestivirus A	MH490943	VIPR_ALG4_AZB53078_1	1b	Erns	BVDV BJ-2016	2016	-0.4147	0.5119	1.1078
Pestivirus A	MH231153	VIPR_ALG4_AZQ00677_1	1b	Erns	Nebraska	1990	-0.4126	0.4991	1.1072
Pestivirus A	AB078950	VIPR_ALG4_BAC55961_1	1j	Erns	KS86-1ncp	1986	-0.3200	0.5078	1.1098
Pestivirus A	MH899941	VIPR_ALG4_QCE30388_1	1b	Erns	SLO/3301/2014	2014	-0.5036	0.4844	1.1111
Pestivirus A	MH899942	VIPR_ALG4_QCE30389_1	1e	Erns	SLO/33529/2015	2015	-0.6055	0.4579	1.1082
Pestivirus A	MH899943	VIPR_ALG4_QCE30390_1	1f	Erns	SLO/1361/2014	2014	-0.2571	0.5356	1.1053
Pestivirus A	MH899944	VIPR_ALG4_QCE30391_1	1f	Erns	SLO/28537/2017	2017	-0.2690	0.4994	1.1053
Pestivirus A	MH899945	VIPR_ALG4_QCE30392_1	1h	Erns	SLO/1883/2013	2013	-0.2512	0.4940	1.1063
Pestivirus A	MK102095	VIPR_ALG4_QCQ84262_1	1q	Erns	20170226	2017	-0.4078	0.5302	1.1100
Pestivirus A	MK509774	VIPR_ALG4_QEK23510_1	1b	Erns	BVD1b-JH	2008	-0.4408	0.5109	1.1072
Pestivirus A	MK775204	VIPR_ALG4_QFX66041_1	1i	Erns	CA2006	2006	-0.2319	0.4738	1.0990
Pestivirus A	MN188073	VIPR_ALG4_QGZ19414_1	1a	Erns	PI34	2017	-0.5040	0.5161	1.1019
Pestivirus A	MN188074	VIPR_ALG4_QGZ19415_1	1b	Erns	PI285	2017	-0.4951	0.5160	1.1138
Pestivirus A	MN394766	VIPR_ALG4_QIB02049_1	1m	Erns	0001	2016	-0.1502	0.4826	1.1136
Pestivirus A	MT079816	VIPR_ALG4_QIM55913_1	1c	Erns	GXNN1	2018	-0.5250	0.5051	1.1050
Pestivirus A	MN623291	VIPR_ALG4_QLL27013_1	1m	Erns	NX2019/01	2019	-0.1427	0.4913	1.1173
Pestivirus A	MW014286	VIPR_ALG4_QPJ59878_1	1b	Erns	GXSS01	2018	-0.4966	0.5096	1.1134
Pestivirus A	MW014287	VIPR_ALG4_QPJ59879_1	1b	Erns	GXSS02	2018	-0.4818	0.4974	1.1157
Pestivirus A	MW014288	VIPR_ALG4_QPJ59880_1	1b	Erns	GXSS03	2018	-0.4818	0.4974	1.1157
Pestivirus A	MT977117	VIPR_ALG4_QRZ20359_1	1b	Erns	BVDV 1b IT16/5	2016	-0.5528	0.4852	1.1153
Pestivirus A	MT977118	VIPR_ALG4_QRZ20360_1	1b	Erns	BVDV 1b IT16/439	2016	-0.5658	0.4772	1.1162
Pestivirus A	MT654137	VIPR_ALG4_QVK82311_1	1a	Erns	20-8536	2020	-0.3818	0.5047	1.0997
Pestivirus A	LT837585	VIPR_ALG4_SLV80196_1	1r	Erns	UNKNOWN-LT837585	2012	-0.3892	0.4745	1.1061
Pestivirus A	MW054933	VIPR_ALG4_UEC94252_1	1f	Erns	LA/230/14	2014	-0.3269	0.5270	1.1060
Pestivirus A	MW054934	VIPR_ALG4_UEC94253_1	1f	Erns	LA/87/05	2005	-0.0192	0.4954	1.1003
Pestivirus A	MW054935	VIPR_ALG4_UEC94254_1	1k	Erns	TO/197/11	2011	-0.3442	0.5649	1.1117
Pestivirus A	MW054936	VIPR_ALG4_UEC94255_1	1g	Erns	UM/111/06	2006	-0.1051	0.4443	1.1101
Pestivirus A	MW054937	VIPR_ALG4_UEC94256_1	1k	Erns	SA/158/09	2009	-0.3354	0.5446	1.1078
Pestivirus A	MW054938	VIPR_ALG4_UEC94257_1	1k	Erns	SA/159/09	2009	-0.3279	0.5846	1.1078
Pestivirus A	MW054939	VIPR_ALG4_UEC94258_1	1f	Erns	LO/151/09	2009	-0.4404	0.5202	1.1037
Pestivirus A	MW054940	VIPR_ALG4_UEC94259_1	1e	Erns	MA/101/05	2005	-0.3756	0.5159	1.1098
Pestivirus A	MW250796	VIPR_ALG4_UEC94260_1	1i	Erns	58-1	2008	-0.5622	0.4641	1.1068
Pestivirus A	MW250797	VIPR_ALG4_UEC94261_1	1i	Erns	58-2	2008	-0.5488	0.4652	1.1077
Pestivirus A	MW250798	VIPR_ALG4_UEC94262_1	1a	Erns	62-2	2008	-0.3909	0.5144	1.1036
Pestivirus A	MW250799	VIPR_ALG4_UEC94263_1	1a	Erns	63-1	2008	-0.5096	0.5455	1.1150
Pestivirus A	MW250800	VIPR_ALG4_UEC94264_1	1d	Erns	67-1	2008	-0.3517	0.5346	1.1012
Pestivirus A	MW250801	VIPR_ALG4_UEC94265_1	1d	Erns	67-2	2008	-0.3630	0.5483	1.1012
Pestivirus A	MW250802	VIPR_ALG4_UEC94266_1	1e	Erns	68-1	2008	-0.4290	0.5283	1.0981
Pestivirus A	MW250803	VIPR_ALG4_UEC94267_1	1i	Erns	69-1	2008	-0.4029	0.4379	1.1090
Pestivirus A	MW655625	VIPR_ALG4_UEC94268_1	1h	Erns	CH-04-01b	2004	-0.4184	0.5342	1.1110
Pestivirus A	MW655626	VIPR_ALG4_UEC94269_1	1e	Erns	Maria	2004	-0.6162	0.5114	1.1103
Pestivirus A	MW655627	VIPR_ALG4_UEC94270_1	1e	Erns	R2000-95	1995	-0.4963	0.4863	1.1108
Pestivirus A	MW655628	VIPR_ALG4_UEC94271_1	1k	Erns	R3230-95	1995	-0.3307	0.5457	1.1114
Pestivirus A	MW655629	VIPR_ALG4_UEC94272_1	1h	Erns	R3572-90	1990	-0.2714	0.4861	1.1051

Species according to VIPRBRC	GenBank Accession	GenBank Protein Accession	Subgenotype	Protein	Strain Name	Collection Year	SVM Patho. Score	Vaxijen Antig. Score	Averged score of EMBOSS motifs
Pestivirus A	MW655630	VIPR_ALG4_UEC94273_1	1k	Erns	R5013-96	1996	-0.2879	0.5353	1.1091
Pestivirus A	MW655631	VIPR_ALG4_UEC94274_1	1e	Erns	S03-1175	2003	-0.6291	0.4758	1.1074
Pestivirus A	MW655632	VIPR_ALG4_UEC94275_1	1h	Erns	SM09-20	2002	-0.2247	0.5003	1.1071
Pestivirus A	MW713361	VIPR_ALG4_UEC94276_1	1a	Erns	BoAEC1190	2007	-0.4803	0.5038	1.1100
Pestivirus A	MW713362	VIPR_ALG4_UEC94277_1	1b	Erns	PI819	2017	-0.3506	0.4993	1.1072
Pestivirus A	MW732738	VIPR_ALG4_UEC94278_1	1a	Erns	PI407	2015	-0.5649	0.5217	1.1094
Pestivirus A	MW732739	VIPR_ALG4_UEC94279_1	1a	Erns	YandaSpl	1993	-0.4495	0.5191	1.1035
Pestivirus A	MZ188972	VIPR_ALG4_UML14262_1	1q	Erns	HB-1	2020	0.0577	0.4676	1.1156
Pestivirus A	MW560180	VIPR_ALG4_UQW60629_	1d	Erns	NX1	2017	-0.1684	0.5340	1.1040
Pestivirus A	MW560181	VIPR_ALG4_UQW60630_	1q	Erns	NX2	2019	-0.2043	0.4911	1.1045
Pestivirus A	MW560183	VIPR_ALG4_UQW60632_	1v	Erns	NX201902	2019	-0.1132	0.4990	1.1123
Pestivirus A	MW560184	VIPR_ALG4_UQW60633_	1m	Erns	NX5	2019	-0.2463	0.5079	1.1173
Pestivirus A	MW560185	VIPR_ALG4_UQW60634_	1m	Erns	NX6	2019	-0.1312	0.5003	1.1150
Pestivirus A	ON337882	VIPR_ALG4_USZ80113_1	1c	Erns	NM2103	2021	-0.3992	0.4401	1.1028
Pestivirus A	KU159365	VIPR_ALG4_1039262063	1a	E1	USII-S15	2015	-1.0742	0.5408	1.1674
Pestivirus A	KU756226	VIPR_ALG4_1072900294	1b	E1	HJ-1	2010	-0.5605	0.5177	1.2133
Pestivirus A	KT943518	VIPR_ALG4_1093530908	1d	E1	BJ1201	2012	-0.6125	0.5638	1.1647
Pestivirus A	LT631725	VIPR_ALG4_1112914034	1h	E1	UM/126/07	2007	-0.8265	0.5020	1.1933
Pestivirus A	KX170065	VIPR_ALG4_1129879396	1b	E1	V015	2001	-0.4609	0.4604	1.1957
Pestivirus A	KX170066	VIPR_ALG4_1129879398	1b	E1	V075	2011	-0.4280	0.4785	1.2016
Pestivirus A	KX170067	VIPR_ALG4_1129879400	1b	E1	V060	2004	-0.9885	0.4595	1.1909
Pestivirus A	KX170068	VIPR_ALG4_1129879402	1b	E1	V070	2007	-0.6263	0.4695	1.1833
Pestivirus A	KX170069	VIPR_ALG4_1129879404	1b	E1	V036	2007	-0.7337	0.4595	1.1969
Pestivirus A	KX170070	VIPR_ALG4_1129879406	1b	E1	V100	1997	-0.7817	0.4630	1.2016
Pestivirus A	KX170071	VIPR_ALG4_1129879408	1b	E1	V098	1999	-0.5842	0.4653	1.1989
Pestivirus A	KX170072	VIPR_ALG4_1129879410	1b	E1	V020	2005	-0.8664	0.4511	1.2016
Pestivirus A	KX170073	VIPR_ALG4_1129879412	1b	E1	V029	2006	-0.8660	0.4537	1.2005
Pestivirus A	KX170074	VIPR_ALG4_1129879414	1b	E1	V087	2006	-0.6879	0.4395	1.1907
Pestivirus A	KX170075	VIPR_ALG4_1129879416	1b	E1	V045	2009	-0.7821	0.4489	1.1969
Pestivirus A	KX170076	VIPR_ALG4_1129879418	1b	E1	V078	2012	-0.5930	0.4385	1.2165
Pestivirus A	KX170077	VIPR_ALG4_1129879420	1b	E1	V031	2006	-0.7904	0.4694	1.1969
Pestivirus A	KX170078	VIPR_ALG4_1129879422	1a	E1	V026	2006	-0.5687	0.5002	1.1830
Pestivirus A	KX170079	VIPR_ALG4_1129879424	1a	E1	V027	2006	-0.5687	0.5002	1.1830
Pestivirus A	KX170080	VIPR_ALG4_1129879426	1a	E1	V091	2003	-0.8541	0.4738	1.1601
Pestivirus A	KX170081	VIPR_ALG4_1129879428	1a	E1	V007	2000	-0.7802	0.4949	1.1652
Pestivirus A	KX170082	VIPR_ALG4_1129879430	1a	E1	V013	2001	-0.7802	0.4949	1.1652
Pestivirus A	KX170083	VIPR_ALG4_1129879432	1a	E1	V033	2007	-0.7802	0.4949	1.1652
Pestivirus A	KX170084	VIPR_ALG4_1129879434	1a	E1	V034	2007	-0.7802	0.4949	1.1652
Pestivirus A	KX170085	VIPR_ALG4_1129879436	1a	E1	V049	2009	-0.7802	0.4949	1.1652
Pestivirus A	KX170086	VIPR_ALG4_1129879438	1a	E1	V067	2006	-0.7802	0.4949	1.1652
Pestivirus A	KX170087	VIPR_ALG4_1129879440	1a	E1	V073	2011	-0.7802	0.4949	1.1652
Pestivirus A	KX170088	VIPR_ALG4_1129879442	1a	E1	V074	2010	-0.7802	0.4949	1.1652
Pestivirus A	KX170089	VIPR_ALG4_1129879444	1a	E1	V077	2012	-0.7802	0.4949	1.1652
Pestivirus A	KX170090	VIPR_ALG4_1129879446	1a	E1	V080	2009	-0.7769	0.4994	1.1652
Pestivirus A	KX170091	VIPR_ALG4_1129879448	1a	E1	V022	2006	-0.9265	0.5385	1.1838
Pestivirus A	KX170092	VIPR_ALG4_1129879450	1a	E1	V054	2013	-0.8372	0.5286	1.1824
Pestivirus A	KX170093	VIPR_ALG4_1129879452	1a	E1	V011	2001	-0.9538	0.5131	1.1780

Species according to VIPRBRC	GenBank Accession	GenBank Protein Accession	Subgenotype	Protein	Strain Name	Collection Year	SVM Patho. Score	Vaxijen Antig. Score	Averged score of EMBOSS motifs
Pestivirus A	KX170094	VIPR_ALG4_1129879454_	1a	E1	V012	2001	-0.9538	0.5131	1.1780
Pestivirus A	KX170095	VIPR_ALG4_1129879456_	1a	E1	V057	2009	-0.9775	0.5065	1.1764
Pestivirus A	KX170096	VIPR_ALG4_1129879458_	1a	E1	V006	2000	-0.9588	0.5247	1.1714
Pestivirus A	KX170097	VIPR_ALG4_1129879460_	1a	E1	V056	2009	-0.8470	0.5201	1.1771
Pestivirus A	KX170098	VIPR_ALG4_1129879462_	1a	E1	V014	2001	-0.6722	0.5318	1.1827
Pestivirus A	KX170099	VIPR_ALG4_1129879464_	1a	E1	V092	2004	-0.8618	0.5483	1.1681
Pestivirus A	KX170100	VIPR_ALG4_1129879466_	1a	E1	V083	2008	-0.9279	0.4860	1.1841
Pestivirus A	KX170101	VIPR_ALG4_1129879468_	1a	E1	V001	1999	-0.7454	0.5300	1.1733
Pestivirus A	KX170102	VIPR_ALG4_1129879470_	1a	E1	V010	2001	-0.4441	0.5369	1.1771
Pestivirus A	KX170103	VIPR_ALG4_1129879472_	1a	E1	V035	2007	-0.8251	0.5319	1.1733
Pestivirus A	KX170104	VIPR_ALG4_1129879474_	1a	E1	V043	2008	-0.8921	0.5341	1.1759
Pestivirus A	KX170105	VIPR_ALG4_1129879476_	1a	E1	V042	2008	-0.8404	0.5444	1.1686
Pestivirus A	KX170106	VIPR_ALG4_1129879478_	1a	E1	V039	2008	-0.7939	0.5246	1.1711
Pestivirus A	KX170107	VIPR_ALG4_1129879480_	1a	E1	V041	2008	-0.7160	0.5295	1.1711
Pestivirus A	KX170108	VIPR_ALG4_1129879482_	1a	E1	V050	2009	-0.8556	0.5300	1.1711
Pestivirus A	KX170109	VIPR_ALG4_1129879484_	1a	E1	V040	2008	-0.8556	0.5300	1.1711
Pestivirus A	KX170110	VIPR_ALG4_1129879486_	1a	E1	V046	2009	-0.8556	0.5300	1.1711
Pestivirus A	KX170111	VIPR_ALG4_1129879488_	1a	E1	V052	2010	-0.8225	0.4840	1.1791
Pestivirus A	KX170112	VIPR_ALG4_1129879490_	1a	E1	V016	2002	-0.6995	0.5356	1.1747
Pestivirus A	KX170113	VIPR_ALG4_1129879492_	1a	E1	V008	2000	-0.8610	0.5416	1.1640
Pestivirus A	KX170114	VIPR_ALG4_1129879494_	1a	E1	V009	2000	-0.8610	0.5416	1.1640
Pestivirus A	KX170115	VIPR_ALG4_1129879496_	1a	E1	V048	2009	-0.6186	0.5110	1.1745
Pestivirus A	KX170116	VIPR_ALG4_1129879498_	1a	E1	V059	2004	-0.6186	0.5110	1.1745
Pestivirus A	KX170117	VIPR_ALG4_1129879500_	1a	E1	V099	1998	-0.8117	0.5102	1.1789
Pestivirus A	EF101530	VIPR_ALG4_118498779_1	1b	E1	KE9	2007	-0.5397	0.4935	1.1701
Pestivirus A	DQ088995	VIPR_ALG4_145309048_1	1a	E1	Singer_Arg	1974	-0.7873	0.4935	1.1652
Pestivirus A	U63479	VIPR_ALG4_1518836_186	1b	E1	CP7	1987	-0.8038	0.4374	1.2143
Pestivirus A	U86600	VIPR_ALG4_2149469_187	1b	E1	ILLNC	1991	-0.8686	0.4692	1.2057
Pestivirus A	AF041040	VIPR_ALG4_2789677_186	1a	E1	Oregon	1960	-0.8638	0.4895	1.1856
Pestivirus A	M96751	VIPR_ALG4_289508_1877	1a	E1	UNKNOWN-M96751	1992	-0.8862	0.5273	1.1733
Pestivirus A	HQ174292	VIPR_ALG4_323145267_1	1a	E1	180	2010	-0.9655	0.5184	1.1886
Pestivirus A	M31182	VIPR_ALG4_323206_1877	1a	E1	UNKNOWN-M31182	1988	-0.7429	0.5110	1.1830
Pestivirus A	M96687	VIPR_ALG4_323230_1875	1b	E1	Osloss	1967	-0.9440	0.4631	1.1973
Pestivirus A	JN400273	VIPR_ALG4_363990275_1	1q	E1	SD0803	2008	-0.8592	0.5285	1.1743
Pestivirus A	AF091605	VIPR_ALG4_3661566_187	1a	E1	Oregon C24V	1960	-0.9328	0.5123	1.1813
Pestivirus A	JN644055	VIPR_ALG4_373939303_1	1b	E1	3156	2011	-0.9043	0.4374	1.1927
Pestivirus A	JN380080	VIPR_ALG4_378753653_1	1a	E1	6010	2010	-0.9781	0.5259	1.1846
Pestivirus A	JQ799141	VIPR_ALG4_390132765_1	1u	E1	M31182	2010	-0.2856	0.4986	1.1796
Pestivirus A	JX419397	VIPR_ALG4_404363562_1	1b	E1	UNKNOWN-JX419397	2008	-0.7299	0.4606	1.2007
Pestivirus A	JX419398	VIPR_ALG4_404363564_1	1b	E1	UNKNOWN-JX419398	2008	-0.7299	0.4606	1.2007
Pestivirus A	AF526381	VIPR_ALG4_42476348_18	1m	E1	ZM-95	1995	-0.6142	0.5371	1.1959
Pestivirus A	JX297512	VIPR_ALG4_459284067_1	1b	E1	10270	2007	-0.6153	0.4778	1.1989
Pestivirus A	JX297513	VIPR_ALG4_459284069_1	1b	E1	Aries	2005	-0.6153	0.4778	1.1989
Pestivirus A	JX297514	VIPR_ALG4_459284071_1	1b	E1	Columba	2005	-0.6153	0.4778	1.1989
Pestivirus A	JX297515	VIPR_ALG4_459284073_1	1b	E1	Corona	2005	-0.6153	0.4778	1.1989
Pestivirus A	JX297516	VIPR_ALG4_459284075_1	1b	E1	Gemini	2005	-0.5521	0.4636	1.1989
Pestivirus A	JX297517	VIPR_ALG4_459284077_1	1b	E1	Hercules	2006	-0.7291	0.4498	1.2034

Species according to VIPRBRC	GenBank Accession	GenBank Protein Accession	Subgenotype	Protein	Strain Name	Collection Year	SVM Patho. Score	Vaxijen Antig. Score	Averged score of EMBOSS motifs
Pestivirus A	JX297518	VIPR_ALG4_459284079_1	1b	E1	Leo	2006	-0.6153	0.4778	1.1989
Pestivirus A	JX297519	VIPR_ALG4_459284081_1	1b	E1	Lyra	2006	-0.6434	0.4764	1.2049
Pestivirus A	JX297520	VIPR_ALG4_459284083_1	1b	E1	Mars	2006	-0.6153	0.4778	1.1989
Pestivirus A	JX297521	VIPR_ALG4_459284085_1	1b	E1	Scorpius	2006	-0.6434	0.4764	1.2049
Pestivirus A	JX306011	VIPR_ALG4_459284087_1	1b	E1	Cepheus	2005	-0.6153	0.4778	1.1989
Pestivirus A	JX306012	VIPR_ALG4_459284089_1	1b	E1	Hamal	2006	-0.5997	0.4754	1.1989
Pestivirus A	JX306013	VIPR_ALG4_459284091_1	1b	E1	Kurhah	2006	-0.6153	0.4778	1.1989
Pestivirus A	JX306014	VIPR_ALG4_459284093_1	1b	E1	Naos	2006	-0.6434	0.4764	1.2049
Pestivirus A	KC853440	VIPR_ALG4_507144146_1	1k	E1	SuwaNcp	1993	-0.9558	0.5065	1.2033
Pestivirus A	KC853441	VIPR_ALG4_507144148_1	1k	E1	SuwaCp	1993	-0.9558	0.5065	1.2033
Pestivirus A	KC695810	VIPR_ALG4_507866685_1	1q	E1	camel-6	2010	-0.7456	0.5519	1.2087
Pestivirus A	KC695811	VIPR_ALG4_507866687_1	1q	E1	GS-3	2012	-0.6728	0.5699	1.2153
Pestivirus A	KC695812	VIPR_ALG4_507866689_1	1q	E1	HB-1	2012	-0.6048	0.5209	1.1651
Pestivirus A	KC695813	VIPR_ALG4_507866693_1	1c	E1	Bega-like	2012	-0.2606	0.4989	1.1776
Pestivirus A	KC695814	VIPR_ALG4_507866704_1	1b	E1	Av69 VEDEVAC	2011	-0.9201	0.4667	1.1927
Pestivirus A	KC695815	VIPR_ALG4_507866706_1	1a	E1	Av69 SD-1	2011	-0.7012	0.5276	1.1733
Pestivirus A	KC695816	VIPR_ALG4_507866709_1	1d	E1	cell-con-1	2012	-0.7853	0.5417	1.1791
Pestivirus A	KC700344	VIPR_ALG4_508083101_1	1b	E1	GS-4	2012	-0.8613	0.4531	1.1946
Pestivirus A	KC757383	VIPR_ALG4_511775165_1	1d	E1	10JJ-SKR	2010	-0.6094	0.5465	1.1769
Pestivirus A	KC963967	VIPR_ALG4_530291194_1	1b	E1	12F004	2012	-0.6272	0.4795	1.2096
Pestivirus A	KF772785	VIPR_ALG4_575471151_1	1b	E1	CC13B	2013	-0.8194	0.4628	1.1786
Pestivirus A	KF896608	VIPR_ALG4_586616532_1	1c	E1	Bega-like	2012	-0.2606	0.4989	1.1776
Pestivirus A	KF835697	VIPR_ALG4_597437474_1	1b	E1	AU526	2013	-0.8634	0.4618	1.2116
Pestivirus A	KJ541471	VIPR_ALG4_633265982_1	1a	E1	GS5	2013	-1.0396	0.4860	1.1989
Pestivirus A	KJ689448	VIPR_ALG4_635172915_1	1b	E1	GX4	2012	-0.9201	0.4667	1.1927
Pestivirus A	KF501393	VIPR_ALG4_669206614_1	1b	E1	BVDV JL-1	2009	-0.6314	0.4919	1.2049
Pestivirus A	AJ133738	VIPR_ALG4_7960754_18	1a	E1	type 1	1963	-0.8916	0.5016	1.1669
Pestivirus A	KP941581	VIPR_ALG4_800924313_1	1b	E1	USMARC-51998	2014	-0.6996	0.4617	1.2069
Pestivirus A	KP941583	VIPR_ALG4_800924317_1	1b	E1	USMARC-53874	2014	-0.6912	0.4658	1.2037
Pestivirus A	KP941584	VIPR_ALG4_800924319_1	1a	E1	USMARC-53875	2014	-0.6889	0.5118	1.1921
Pestivirus A	KP941586	VIPR_ALG4_800924323_1	1a	E1	USMARC-55477	2014	-0.9031	0.4658	1.1877
Pestivirus A	KP941587	VIPR_ALG4_800924325_1	1b	E1	USMARC-55478	2014	-0.5741	0.4954	1.2143
Pestivirus A	KP941588	VIPR_ALG4_800924327_1	1b	E1	USMARC-55922	2014	-0.7136	0.5023	1.1937
Pestivirus A	KP941589	VIPR_ALG4_800924329_1	1b	E1	USMARC-55923	2014	-0.8072	0.4596	1.2069
Pestivirus A	KP941590	VIPR_ALG4_800924331_1	1b	E1	USMARC-55924	2014	-0.8551	0.4696	1.2096
Pestivirus A	KP941591	VIPR_ALG4_800924333_1	1b	E1	USMARC-55925	2014	-0.5449	0.4460	1.1957
Pestivirus A	KP941592	VIPR_ALG4_800924335_1	1b	E1	USMARC-55926	2014	-0.7206	0.4394	1.2021
Pestivirus A	KP313732	VIPR_ALG4_816850387_1	1e	E1	Carlito	2014	-0.4215	0.4868	1.1890
Pestivirus A	KR013753	VIPR_ALG4_871332680_1	1a	E1	WAX-N	1992	-1.1725	0.5258	1.1983
Pestivirus A	KR029825	VIPR_ALG4_887497286_1	1b	E1	Egy/Ismailia/2014	2014	-0.9412	0.4477	1.2049
Pestivirus A	LC089875	VIPR_ALG4_939106262_1	1o	E1	IS26/01ncp	2001	-0.3083	0.4728	1.1865
Pestivirus A	LC089876	VIPR_ALG4_939106264_1	1n	E1	Shitara/02/06	2006	-0.6724	0.4870	1.1843
Pestivirus A	KR866116	VIPR_ALG4_941508008_1	1m	E1	SD-15	2015	-0.4211	0.5581	1.2148
Pestivirus A	KU200260	VIPR_ALG4_972905813_1	1b	E1	BE/061536/2014	2014	-0.6227	0.4687	1.1873
Pestivirus A	KX577637	VIPR_ALG4_AOR50934_1	1e	E1	SLO/2407/2006	2006	-0.5275	0.5181	1.1671
Pestivirus A	KX987157	VIPR_ALG4_APG30987_1	1f	E1	SLO/1170/2000	2000	-0.2503	0.4381	1.2068
Pestivirus A	KX857724	VIPR_ALG4_APZ85839_1	1i	E1	ACM/BR/2016	2016	-0.4193	0.4938	1.1769

Species according to VIPRBRC	GenBank Accession	GenBank Protein Accession	Subgenotype	Protein	Strain Name	Collection Year	SVM Patho. Score	Vaxijen Antig. Score	Averged score of EMBOSS motifs
Pestivirus A	KY849592	VIPR_ALG4_ART90617_1	1d	E1	SLO/2416/2002	2002	-0.9655	0.5366	1.1739
Pestivirus A	MF278651	VIPR_ALG4_ASW18434_1	1b	E1	XZ01	2016	-0.9152	0.4416	1.1927
Pestivirus A	MF278652	VIPR_ALG4_ASW18435_1	1b	E1	XZ02	2016	-0.8568	0.4365	1.1954
Pestivirus A	MF693403	VIPR_ALG4_ATG71375_1	1a	E1	UNKNOWN-MF693403	2016	0.1670	0.4917	1.1545
Pestivirus A	KY964311	VIPR_ALG4_ATN39078_1	1b	E1	Y2	2014	-0.6750	0.4440	1.2024
Pestivirus A	MF172980	VIPR_ALG4_AVI10261_1_	1c	E1	GSTZ	2012	-0.3479	0.5312	1.1944
Pestivirus A	MH379638	VIPR_ALG4_AWW14171_	1a	E1	Ho916	1993	-0.6999	0.5254	1.1854
Pestivirus A	MG950344	VIPR_ALG4_AWW87346_	1b	E1	AU526	2014	-0.8634	0.4618	1.2116
Pestivirus A	MG950345	VIPR_ALG4_AWW87347_	1b	E1	B1	2015	-0.8634	0.4618	1.2116
Pestivirus A	MG950346	VIPR_ALG4_AWW87348_	1b	E1	B2	2015	-0.8634	0.4618	1.2116
Pestivirus A	MG950347	VIPR_ALG4_AWW87349_	1b	E1	B3	2015	-0.8634	0.4618	1.2116
Pestivirus A	MG950348	VIPR_ALG4_AWW87350_	1b	E1	B4	2015	-0.8877	0.4634	1.2116
Pestivirus A	MG950349	VIPR_ALG4_AWW87351_	1b	E1	B5	2015	-0.8634	0.4618	1.2116
Pestivirus A	MG950350	VIPR_ALG4_AWW87352_	1b	E1	B6	2015	-0.8634	0.4618	1.2116
Pestivirus A	MG950351	VIPR_ALG4_AWW87353_	1b	E1	O1	2015	-0.8634	0.4618	1.2116
Pestivirus A	MG950352	VIPR_ALG4_AWW87354_	1b	E1	O2	2015	-0.8439	0.4691	1.2116
Pestivirus A	MG950353	VIPR_ALG4_AWW87355_	1b	E1	O3	2015	-0.8679	0.4601	1.2116
Pestivirus A	MG950354	VIPR_ALG4_AWW87356_	1b	E1	O4	2015	-0.8679	0.4601	1.2116
Pestivirus A	MG950355	VIPR_ALG4_AWW87357_	1b	E1	O5	2015	-0.8679	0.4601	1.2116
Pestivirus A	MG950356	VIPR_ALG4_AWW87358_	1b	E1	O6	2015	-0.8679	0.4601	1.2116
Pestivirus A	MG950357	VIPR_ALG4_AWW87359_	1b	E1	B1A	2015	-0.7143	0.4825	1.2116
Pestivirus A	MG950358	VIPR_ALG4_AWW87360_	1b	E1	B2A	2016	-0.8634	0.4618	1.2116
Pestivirus A	MG950359	VIPR_ALG4_AWW87361_	1b	E1	B3A	2016	-0.8611	0.4605	1.2143
Pestivirus A	MG950360	VIPR_ALG4_AWW87362_	1b	E1	B4A	2016	-0.9534	0.4460	1.2116
Pestivirus A	MG950361	VIPR_ALG4_AWW87363_	1b	E1	B5A	2016	-0.8950	0.4734	1.2116
Pestivirus A	MG950362	VIPR_ALG4_AWW87364_	1b	E1	B6A	2016	-0.8634	0.4618	1.2116
Pestivirus A	MG950363	VIPR_ALG4_AWW87365_	1b	E1	O1A	2015	-0.8493	0.4831	1.2116
Pestivirus A	MG950364	VIPR_ALG4_AWW87366_	1b	E1	O2A	2015	-0.8679	0.4601	1.2116
Pestivirus A	MG950365	VIPR_ALG4_AWW87367_	1b	E1	O2B	2015	-0.8679	0.4601	1.2116
Pestivirus A	MG950366	VIPR_ALG4_AWW87368_	1b	E1	O4A	2015	-0.8679	0.4601	1.2116
Pestivirus A	MH311874	VIPR_ALG4_AWW87369_	1b	E1	B2A d168	2016	-0.8634	0.4618	1.2116
Pestivirus A	MH311875	VIPR_ALG4_AWW87370_	1b	E1	B3A d168	2016	-0.8874	0.4723	1.2143
Pestivirus A	MH311876	VIPR_ALG4_AWW87371_	1b	E1	B4A d84	2016	-0.8634	0.4618	1.2116
Pestivirus A	MH311877	VIPR_ALG4_AWW87372_	1b	E1	B4A d168	2016	-0.8634	0.4618	1.2116
Pestivirus A	MH311878	VIPR_ALG4_AWW87373_	1b	E1	B5A d84	2016	-0.8509	0.5250	1.1954
Pestivirus A	MH311879	VIPR_ALG4_AWW87374_	1b	E1	B5A d168	2016	-0.8634	0.4618	1.2116
Pestivirus A	MH311880	VIPR_ALG4_AWW87375_	1b	E1	B6A d84	2016	-0.8634	0.4618	1.2116
Pestivirus A	MH311881	VIPR_ALG4_AWW87376_	1b	E1	B6A d168	2016	-0.8634	0.4618	1.2116
Pestivirus A	MH379221	VIPR_ALG4_AWW87377_	1b	E1	P1	2017	-0.8679	0.4601	1.2116
Pestivirus A	MH379222	VIPR_ALG4_AWW87378_	1b	E1	P2	2017	-0.8679	0.4601	1.2116
Pestivirus A	MH379223	VIPR_ALG4_AWW87379_	1b	E1	P5	2017	-0.8679	0.4601	1.2116
Pestivirus A	MH379224	VIPR_ALG4_AWW87380_	1b	E1	P6	2017	-0.8679	0.4601	1.2116
Pestivirus A	MH379225	VIPR_ALG4_AWW87381_	1b	E1	P7	2017	-0.8679	0.4601	1.2116
Pestivirus A	MH379226	VIPR_ALG4_AWW87382_	1b	E1	P5A	2017	-0.8679	0.4601	1.2116
Pestivirus A	MH379227	VIPR_ALG4_AWW87383_	1b	E1	P5B	2017	-0.8679	0.4601	1.2116
Pestivirus A	MH379228	VIPR_ALG4_AWW87384_	1b	E1	P5C	2017	-0.8679	0.4601	1.2116
Pestivirus A	MH379229	VIPR_ALG4_AWW87385_	1b	E1	P5D	2017	-0.8679	0.4601	1.2116

Species according to VIPRBRC	GenBank Accession	GenBank Protein Accession	Subgenotype	Protein	Strain Name	Collection Year	SVM Patho. Score	Vaxijen Antig. Score	Averged score of EMBOSS motifs
Pestivirus A	MH379230	VIPR_ALG4_AWW87386_	1b	E1	P5F	2017	-0.8679	0.4601	1.2116
Pestivirus A	MH379231	VIPR_ALG4_AWW87387_	1b	E1	P7A	2018	-0.8679	0.4601	1.2116
Pestivirus A	MH379232	VIPR_ALG4_AWW87388_	1b	E1	P7C	2018	-0.8679	0.4601	1.2116
Pestivirus A	MH379233	VIPR_ALG4_AWW87389_	1b	E1	P7E	2018	-0.8679	0.4601	1.2116
Pestivirus A	MH379234	VIPR_ALG4_AWW87390_	1b	E1	P7F	2018	-0.7892	0.4624	1.2116
Pestivirus A	MH166806	VIPR_ALG4_AYA62524_1	1m	E1	XC	2015	-0.4071	0.4835	1.2190
Pestivirus A	MH490943	VIPR_ALG4_AZB53078_1	1b	E1	BVDV BJ-2016	2016	-0.8466	0.4526	1.2096
Pestivirus A	MH231153	VIPR_ALG4_AZQ00677_1	1b	E1	Nebraska	1990	-0.8408	0.4945	1.1994
Pestivirus A	AB078950	VIPR_ALG4_BAC55961_1	1j	E1	KS86-1ncp	1986	-0.1123	0.4686	1.2105
Pestivirus A	MH899941	VIPR_ALG4_QCE30388_1	1b	E1	SLO/3301/2014	2014	-0.7719	0.4160	1.1891
Pestivirus A	MH899942	VIPR_ALG4_QCE30389_1	1e	E1	SLO/33529/2015	2015	-0.5808	0.5013	1.1843
Pestivirus A	MH899943	VIPR_ALG4_QCE30390_1	1f	E1	SLO/1361/2014	2014	-0.6278	0.4165	1.1887
Pestivirus A	MH899944	VIPR_ALG4_QCE30391_1	1f	E1	SLO/28537/2017	2017	-0.3144	0.4464	1.1947
Pestivirus A	MH899945	VIPR_ALG4_QCE30392_1	1h	E1	SLO/1883/2013	2013	-0.5943	0.4951	1.2182
Pestivirus A	MK102095	VIPR_ALG4_QCQ84262_1	1q	E1	20170226	2017	-0.3422	0.5234	1.1906
Pestivirus A	MK509774	VIPR_ALG4_QEK23510_1	1b	E1	BVD1b-JH	2008	-0.7197	0.4616	1.2069
Pestivirus A	MK775204	VIPR_ALG4_QFX66041_1	1i	E1	CA2006	2006	-0.6935	0.4766	1.1669
Pestivirus A	MN188073	VIPR_ALG4_QGZ19414_1	1a	E1	PI34	2017	-0.8505	0.5197	1.1681
Pestivirus A	MN188074	VIPR_ALG4_QGZ19415_1	1b	E1	PI285	2017	-0.6984	0.4261	1.2096
Pestivirus A	MN394766	VIPR_ALG4_QIB02049_1	1m	E1	0001	2016	-0.1141	0.4760	1.2029
Pestivirus A	MT079816	VIPR_ALG4_QIM55913_1	1c	E1	GXNN1	2018	-0.3767	0.5042	1.1836
Pestivirus A	MN623291	VIPR_ALG4_QLL27013_1	1m	E1	NX2019/01	2019	-0.5529	0.5390	1.1923
Pestivirus A	MW014286	VIPR_ALG4_QPJ59878_1	1b	E1	GXSS01	2018	-0.9201	0.4667	1.1927
Pestivirus A	MW014287	VIPR_ALG4_QPJ59879_1	1b	E1	GXSS02	2018	-0.8569	0.4880	1.1883
Pestivirus A	MW014288	VIPR_ALG4_QPJ59880_1	1b	E1	GXSS03	2018	-0.8983	0.4661	1.1927
Pestivirus A	MT977117	VIPR_ALG4_QRZ20359_1	1b	E1	BVDV 1b IT16/5	2016	-0.7078	0.4598	1.1843
Pestivirus A	MT977118	VIPR_ALG4_QRZ20360_1	1b	E1	BVDV 1b IT16/439	2016	-0.7078	0.4598	1.1843
Pestivirus A	MT654137	VIPR_ALG4_QVK82311_1	1a	E1	20-8536	2020	-1.0575	0.4899	1.1866
Pestivirus A	LT837585	VIPR_ALG4_SLV80196_1	1r	E1	UNKNOWN-LT837585	2012	-0.3068	0.4837	1.2212
Pestivirus A	MW054933	VIPR_ALG4_UEC94252_1	1f	E1	LA/230/14	2014	0.0417	0.3924	1.1969
Pestivirus A	MW054934	VIPR_ALG4_UEC94253_1	1f	E1	LA/87/05	2005	-0.5648	0.4818	1.1546
Pestivirus A	MW054935	VIPR_ALG4_UEC94254_1	1k	E1	TO/197/11	2011	-0.9814	0.4976	1.2200
Pestivirus A	MW054936	VIPR_ALG4_UEC94255_1	1g	E1	UM/111/06	2006	-0.5792	0.4780	1.1717
Pestivirus A	MW054937	VIPR_ALG4_UEC94256_1	1k	E1	SA/158/09	2009	-0.9033	0.5002	1.1896
Pestivirus A	MW054938	VIPR_ALG4_UEC94257_1	1k	E1	SA/159/09	2009	-0.9033	0.5002	1.1896
Pestivirus A	MW054939	VIPR_ALG4_UEC94258_1	1f	E1	LO/151/09	2009	0.0434	0.4176	1.2068
Pestivirus A	MW054940	VIPR_ALG4_UEC94259_1	1e	E1	MA/101/05	2005	-0.6704	0.5289	1.1723
Pestivirus A	MW250796	VIPR_ALG4_UEC94260_1	1i	E1	58-1	2008	-0.4765	0.4915	1.1914
Pestivirus A	MW250797	VIPR_ALG4_UEC94261_1	1i	E1	58-2	2008	-0.4765	0.4915	1.1914
Pestivirus A	MW250798	VIPR_ALG4_UEC94262_1	1a	E1	62-2	2008	-0.8440	0.5095	1.1798
Pestivirus A	MW250799	VIPR_ALG4_UEC94263_1	1a	E1	63-1	2008	-0.9575	0.4966	1.1908
Pestivirus A	MW250800	VIPR_ALG4_UEC94264_1	1d	E1	67-1	2008	-0.6328	0.5307	1.1634
Pestivirus A	MW250801	VIPR_ALG4_UEC94265_1	1d	E1	67-2	2008	-0.6328	0.5307	1.1634
Pestivirus A	MW250802	VIPR_ALG4_UEC94266_1	1e	E1	68-1	2008	-0.5161	0.5140	1.1840
Pestivirus A	MW250803	VIPR_ALG4_UEC94267_1	1i	E1	69-1	2008	-0.5573	0.5135	1.1837
Pestivirus A	MW655625	VIPR_ALG4_UEC94268_1	1h	E1	CH-04-01b	2004	-0.6875	0.5128	1.1683
Pestivirus A	MW655626	VIPR_ALG4_UEC94269_1	1e	E1	Maria	2004	-0.4084	0.4771	1.1949

Species according to VIPRBRC	GenBank Accession	GenBank Protein Accession	Subgenotype	Protein	Strain Name	Collection Year	SVM Patho. Score	Vaxijen Antig. Score	Averged score of EMBOSS motifs
Pestivirus A	MW655627	VIPR_ALG4_UEC94270_1	1e	E1	R2000-95	1995	-0.7746	0.5161	1.1646
Pestivirus A	MW655628	VIPR_ALG4_UEC94271_1	1k	E1	R3230-95	1995	-0.8584	0.5531	1.2100
Pestivirus A	MW655629	VIPR_ALG4_UEC94272_1	1h	E1	R3572-90	1990	-0.8604	0.5136	1.1865
Pestivirus A	MW655630	VIPR_ALG4_UEC94273_1	1k	E1	R5013-96	1996	-1.1449	0.4815	1.1901
Pestivirus A	MW655631	VIPR_ALG4_UEC94274_1	1e	E1	S03-1175	2003	-0.5910	0.5229	1.1855
Pestivirus A	MW655632	VIPR_ALG4_UEC94275_1	1h	E1	SM09-20	2002	-0.6213	0.5117	1.1940
Pestivirus A	MW713361	VIPR_ALG4_UEC94276_1	1a	E1	BoAEC1190	2007	-0.8791	0.4838	1.1709
Pestivirus A	MW713362	VIPR_ALG4_UEC94277_1	1b	E1	PI819	2017	-0.5252	0.4798	1.2096
Pestivirus A	MW732738	VIPR_ALG4_UEC94278_1	1a	E1	PI407	2015	-0.9939	0.5207	1.1824
Pestivirus A	MW732739	VIPR_ALG4_UEC94279_1	1a	E1	YandaSpl	1993	-0.8723	0.5155	1.1856
Pestivirus A	MZ188972	VIPR_ALG4_UML14262_1	1q	E1	HB-1	2020	-0.1439	0.5117	1.1785
Pestivirus A	ON337882	VIPR_ALG4_USZ80113_1	1c	E1	NM2103	2021	-0.2990	0.5246	1.1899
Pestivirus A	JF968611	AEQ66844.1	1b	E2	BSU1	2008	-0.4598	0.6123	1.1310
Pestivirus A	JN377417	AEV54355.1	1b	E2	M15.1	2009	-0.5012	0.5612	1.1285
Pestivirus A	JN377418	AEV54356.1	1b	E2	M15.2	2009	-0.5148	0.5620	1.1276
Pestivirus A	JN377419	AEV54357.1	1b	E2	M15.3	2009	-0.5023	0.5362	1.1293
Pestivirus A	JN377420	AEV54358.1	1b	E2	M15.4	2009	-0.5168	0.5624	1.1291
Pestivirus A	JN377421	AEV54359.1	1b	E2	M15.5	2009	-0.5168	0.5624	1.1291
Pestivirus A	JN377422	AEV54360.1	1b	E2	M15.6	2009	-0.4671	0.5636	1.1276
Pestivirus A	JN377423	AEV54361.1	1b	E2	M15.7	2009	-0.5148	0.5620	1.1276
Pestivirus A	JN377424	AEV54362.1	1b	E2	M181 22	2009	-0.6335	0.5620	1.1259
Pestivirus A	LC630446	BCV19807.1	1b	E2	BVDV/Ibaraki/32/15	2015	-0.6341	0.5899	1.1298
Pestivirus A	LC630447	BCV19808.1	1b	E2	BVDV/Ibaraki/36/15	2015	-0.5943	0.5818	1.1329
Pestivirus A	LC630448	BCV19809.1	1b	E2	BVDV/Gunma/01/16	2016	-0.4918	0.5719	1.1307
Pestivirus A	LC630449	BCV19810.1	1b	E2	BVDV/Gunma/03/16	2016	-0.5256	0.5652	1.1307
Pestivirus A	LC630450	BCV19811.1	1b	E2	BVDV/Gunma/05/16	2016	-0.3874	0.5587	1.1250
Pestivirus A	LC630451	BCV19812.1	1b	E2	BVDV/Gunma/01/17	2017	-0.4351	0.5415	1.1347
Pestivirus A	LC630452	BCV19813.1	1b	E2	BVDV/Gunma/02/17	2017	-0.4099	0.5411	1.1310
Pestivirus A	LC630453	BCV19814.1	1b	E2	BVDV/Gunma/03/17	2017	-0.5681	0.6056	1.1291
Pestivirus A	LC630454	BCV19815.1	1b	E2	BVDV/Gunma/06/17	2017	-0.4271	0.5950	1.1317
Pestivirus A	LC630455	BCV19816.1	1b	E2	BVDV/Gunma/07/17	2017	-0.4748	0.5872	1.1254
Pestivirus A	LC630456	BCV19817.1	1b	E2	BVDV/Gunma/11/17	2017	-0.3831	0.5404	1.1304
Pestivirus A	LC630457	BCV19818.1	1b	E2	BVDV/Gunma/01/18	2018	-0.4463	0.5326	1.1306
Pestivirus A	LC630458	BCV19819.1	1b	E2	BVDV/Gunma/04/18	2018	-0.6391	0.6133	1.1299
Pestivirus A	LC630459	BCV19820.1	1b	E2	BVDV/Gunma/12/18	2018	-0.3874	0.5587	1.1250
Pestivirus A	LC630460	BCV19821.1	1b	E2	BVDV/Gunma/13/18	2018	-0.3098	0.5242	1.1311
Pestivirus A	LC630461	BCV19822.1	1b	E2	BVDV/Gunma/14/18	2018	-0.4635	0.5800	1.1302
Pestivirus A	LC630462	BCV19823.1	1b	E2	BVDV/Gunma/19/18	2018	-0.3841	0.5682	1.1292
Pestivirus A	LC630463	BCV19824.1	1b	E2	BVDV/Gunma/22/18	2018	-0.3278	0.5504	1.1288
Pestivirus A	LC630464	BCV19825.1	1b	E2	BVDV/Gunma/23/18	2018	-0.3642	0.5207	1.1311
Pestivirus A	LC630466	BCV19826.1	1b	E2	BVDV/Gunma/05/19	2019	-0.4068	0.5439	1.1331
Pestivirus A	LC630467	BCV19827.1	1b	E2	BVDV/Gunma/07/19	2019	-0.3661	0.5896	1.1284
Pestivirus A	LC630468	BCV19828.1	1b	E2	BVDV/Gunma/11/19	2019	-0.3714	0.5623	1.1269
Pestivirus A	LC630469	BCV19829.1	1b	E2	BVDV/Gunma/15/19	2019	-0.3503	0.5498	1.1266
Pestivirus A	LC630470	BCV19830.1	1b	E2	BVDV/Okayama/11/18	2018	-0.5820	0.5338	1.1424
Pestivirus A	LC630471	BCV19831.1	1b	E2	BVDV/Okayama/21/19	2019	-0.4555	0.5606	1.1363
Pestivirus A	LC630472	BCV19832.1	1b	E2	BVDV/Okayama/24/20	2020	-0.4547	0.5480	1.1341

Species according to VIPRBRC	GenBank Accession	GenBank Protein Accession	Subgenotype	Protein	Strain Name	Collection Year	SVM Patho. Score	Vaxijen Antig. Score	Averged score of EMBOSS motifs
Pestivirus A	LC630473	BCV19833.1	1b	E2	BVDV/Kumamoto/01/20	2020	-0.6509	0.5377	1.1275
Pestivirus A	LC630474	BCV19834.1	1b	E2	BVDV/Oita/01/18	2018	-0.5913	0.5808	1.1359
Pestivirus A	LC630475	BCV19835.1	1b	E2	BVDV/Oita/07/18	2018	-0.3515	0.5288	1.1305
Pestivirus A	LC630476	BCV19836.1	1b	E2	BVDV/Oita/10/18	2018	-0.5207	0.5406	1.1287
Pestivirus A	LC630465	BCW91505.1	1b	E2	BVDV/Gunma/24/18	2018	-0.3886	0.5335	1.1304
Pestivirus A	LC648252	BDA82227.1	1b	E2	BVDV/Taiki/I_22	2019	-0.5555	0.6108	1.1271
Pestivirus A	LC648253	BDA82228.1	2c	E2	BVDV/Taiki/A_4	2018	-0.3874	0.4533	1.1095
Pestivirus A	LC648254	BDA82229.1	1b	E2	BVDV/Taiki/A_3	2018	-0.5700	0.5950	1.1304
Pestivirus A	LC648255	BDA82230.1	1b	E2	BVDV/Taiki/A_2	2018	-0.6019	0.5820	1.1286
Pestivirus A	LC648256	BDA82231.1	1b	E2	BVDV/Taiki/A_1	2018	-0.5700	0.5950	1.1304
Pestivirus A	LC648257	BDA82232.1	2c	E2	BVDV/Shintoku/F_16	2018	-0.3591	0.4911	1.1126
Pestivirus A	LC648258	BDA82233.1	1b	E2	BVDV/Shihoro/O_40	2020	-0.3072	0.5443	1.1318
Pestivirus A	LC648259	BDA82234.1	1b	E2	BVDV/Shihoro/O_39	2020	-0.3325	0.5369	1.1318
Pestivirus A	LC648260	BDA82235.1	1b	E2	BVDV/Shihoro/B_41	2020	-0.3912	0.5440	1.1335
Pestivirus A	LC648261	BDA82236.1	1b	E2	BVDV/Shihoro/B_6	2018	-0.4808	0.5760	1.1251
Pestivirus A	LC648262	BDA82237.1	2c	E2	BVDV/Shihoro/B_5	2018	-0.5135	0.4670	1.1134
Pestivirus A	LC648263	BDA82238.1	1b	E2	BVDV/Otohuke/J_26	2019	-0.3401	0.5463	1.1312
Pestivirus A	LC648264	BDA82239.1	1b	E2	BVDV/Otohuke/J_25	2019	-0.3540	0.5545	1.1312
Pestivirus A	LC648265	BDA82240.1	1b	E2	BVDV/Otohuke/J_23	2019	-0.3261	0.5472	1.1301
Pestivirus A	LC648266	BDA82241.1	1b	E2	BVDV/Kamishihoro/L_29	2020	-0.3716	0.5383	1.1312
Pestivirus A	LC648267	BDA82242.1	1b	E2	BVDV/Kamishihoro/L_28	2020	-0.3716	0.5383	1.1312
Pestivirus A	LC648268	BDA82243.1	1b	E2	BVDV/Honbetu/M_33	2020	-0.3672	0.5140	1.1276
Pestivirus A	LC648269	BDA82244.1	1b	E2	BVDV/Honbetu/M_30	2020	-0.3473	0.5250	1.1312
Pestivirus A	LC648270	BDA82245.1	1b	E2	BVDV/Hiroo/G_20	2019	-0.3506	0.6077	1.1332
Pestivirus A	LC648271	BDA82246.1	1b	E2	BVDV/Hiroo/G_19	2019	-0.2660	0.5869	1.1327
Pestivirus A	LC648272	BDA82247.1	1b	E2	BVDV/Hiroo/G_18	2019	-0.2660	0.5869	1.1327
Pestivirus A	LC648273	BDA82248.1	1b	E2	BVDV/Hiroo/G_17	2019	-0.3669	0.6106	1.1346
Pestivirus A	LC648274	BDA82249.1	1b	E2	BVDV/Hiroo/E_14	2018	-0.3672	0.5315	1.1339
Pestivirus A	LC648275	BDA82250.1	1b	E2	BVDV/Hiroo/D_12	2018	-0.3672	0.5315	1.1339
Pestivirus A	LC648276	BDA82251.1	1b	E2	BVDV/Hiroo/D_13	2018	-0.2679	0.5358	1.1390
Pestivirus A	LC648277	BDA82252.1	1b	E2	BVDV/Hiroo/D_10	2018	-0.3638	0.5304	1.1350
Pestivirus A	LC648278	BDA82253.1	1b	E2	BVDV/Hiroo/D_9	2018	-0.3207	0.5318	1.1339
Pestivirus A	LC648279	BDA82254.1	1b	E2	BVDV/Hiroo/D_8	2018	-0.3207	0.5318	1.1339
Pestivirus A	KU159365	VIPR_ALG4_1039262063_	1a	E2	USII-S15	2015	-0.6605	0.6474	1.1278
Pestivirus A	KU756226	VIPR_ALG4_1072900294_	1b	E2	HJ-1	2010	-0.6534	0.6288	1.1277
Pestivirus A	KT943518	VIPR_ALG4_1093530908_	1d	E2	BJ1201	2012	-0.5890	0.6342	1.1261
Pestivirus A	LT631725	VIPR_ALG4_1112914034_	1h	E2	UM/126/07	2007	-0.6195	0.5859	1.1283
Pestivirus A	KX170144	VIPR_ALG4_1129879554_	1b	E2	V015	2001	-0.5373	0.5832	1.1328
Pestivirus A	KX170145	VIPR_ALG4_1129879556_	1b	E2	V100	1997	-0.4646	0.5762	1.1328
Pestivirus A	KX170146	VIPR_ALG4_1129879558_	1b	E2	V075	2011	-0.6757	0.5690	1.1388
Pestivirus A	KX170147	VIPR_ALG4_1129879560_	1b	E2	V060	2004	-0.5573	0.5843	1.1279
Pestivirus A	KX170148	VIPR_ALG4_1129879562_	1b	E2	V070	2007	-0.7052	0.5487	1.1283
Pestivirus A	KX170149	VIPR_ALG4_1129879564_	1b	E2	V036	2007	-0.3719	0.5908	1.1291
Pestivirus A	KX170150	VIPR_ALG4_1129879566_	1b	E2	V098	1999	-0.2157	0.5282	1.1278
Pestivirus A	KX170151	VIPR_ALG4_1129879568_	1b	E2	V020	2005	-0.5871	0.5675	1.1318
Pestivirus A	KX170152	VIPR_ALG4_1129879570_	1b	E2	V029	2006	-0.5539	0.5913	1.1368
Pestivirus A	KX170153	VIPR_ALG4_1129879572_	1b	E2	V045	2009	-0.5282	0.5743	1.1306

Species according to VIPRBRC	GenBank Accession	GenBank Protein Accession	Subgenotype	Protein	Strain Name	Collection Year	SVM Patho. Score	Vaxijen Antig. Score	Averged score of EMBOSS motifs
Pestivirus A	KX170154	VIPR_ALG4_1129879574_	1b	E2	V078	2012	-0.5529	0.6037	1.1264
Pestivirus A	KX170155	VIPR_ALG4_1129879576_	1b	E2	V031	2006	-0.7182	0.5609	1.1218
Pestivirus A	KX170156	VIPR_ALG4_1129879578_	1b	E2	V087	2006	-0.5548	0.5509	1.1320
Pestivirus A	KX170157	VIPR_ALG4_1129879580_	1a	E2	V026	2006	-0.6163	0.6412	1.1152
Pestivirus A	KX170158	VIPR_ALG4_1129879582_	1a	E2	V027	2006	-0.6163	0.6412	1.1152
Pestivirus A	KX170159	VIPR_ALG4_1129879584_	1a	E2	V091	2003	-0.8520	0.6924	1.1128
Pestivirus A	KX170160	VIPR_ALG4_1129879586_	1a	E2	V007	2000	-0.8235	0.6560	1.1128
Pestivirus A	KX170161	VIPR_ALG4_1129879588_	1a	E2	V013	2001	-0.8235	0.6560	1.1128
Pestivirus A	KX170162	VIPR_ALG4_1129879590_	1a	E2	V033	2007	-0.8235	0.6560	1.1128
Pestivirus A	KX170163	VIPR_ALG4_1129879592_	1a	E2	V034	2007	-0.8235	0.6560	1.1128
Pestivirus A	KX170164	VIPR_ALG4_1129879594_	1a	E2	V067	2006	-0.8235	0.6560	1.1128
Pestivirus A	KX170165	VIPR_ALG4_1129879596_	1a	E2	V074	2010	-0.8235	0.6560	1.1128
Pestivirus A	KX170166	VIPR_ALG4_1129879598_	1a	E2	V073	2011	-0.8235	0.6560	1.1128
Pestivirus A	KX170167	VIPR_ALG4_1129879600_	1a	E2	V049	2009	-0.8444	0.6697	1.1128
Pestivirus A	KX170168	VIPR_ALG4_1129879602_	1a	E2	V077	2012	-0.8444	0.6697	1.1128
Pestivirus A	KX170169	VIPR_ALG4_1129879604_	1a	E2	V080	2009	-0.8444	0.6697	1.1128
Pestivirus A	KX170170	VIPR_ALG4_1129879606_	1a	E2	V083	2008	-0.5932	0.6163	1.1246
Pestivirus A	KX170171	VIPR_ALG4_1129879608_	1a	E2	V022	2006	-0.6677	0.6920	1.1233
Pestivirus A	KX170172	VIPR_ALG4_1129879610_	1a	E2	V014	2001	-0.5842	0.6465	1.1234
Pestivirus A	KX170173	VIPR_ALG4_1129879612_	1a	E2	V092	2004	-0.6754	0.7006	1.1222
Pestivirus A	KX170174	VIPR_ALG4_1129879614_	1a	E2	V054	2013	-0.5434	0.6547	1.1183
Pestivirus A	KX170175	VIPR_ALG4_1129879616_	1a	E2	V011	2001	-0.6019	0.6507	1.1248
Pestivirus A	KX170176	VIPR_ALG4_1129879618_	1a	E2	V012	2001	-0.5964	0.6551	1.1255
Pestivirus A	KX170177	VIPR_ALG4_1129879620_	1a	E2	V056	2009	-0.6181	0.6418	1.1203
Pestivirus A	KX170178	VIPR_ALG4_1129879622_	1a	E2	V057	2009	-0.6298	0.6493	1.1219
Pestivirus A	KX170179	VIPR_ALG4_1129879624_	1a	E2	V006	2000	-0.4598	0.6457	1.1206
Pestivirus A	KX170180	VIPR_ALG4_1129879626_	1a	E2	V001	1999	-0.7233	0.6218	1.1121
Pestivirus A	KX170181	VIPR_ALG4_1129879628_	1a	E2	V016	2002	-0.5852	0.6063	1.1123
Pestivirus A	KX170182	VIPR_ALG4_1129879630_	1a	E2	V010	2001	-0.6407	0.6179	1.1136
Pestivirus A	KX170183	VIPR_ALG4_1129879632_	1a	E2	V008	2000	-0.6954	0.6253	1.1149
Pestivirus A	KX170184	VIPR_ALG4_1129879634_	1a	E2	V009	2000	-0.6644	0.6324	1.1133
Pestivirus A	KX170185	VIPR_ALG4_1129879636_	1a	E2	V040	2008	-0.5811	0.6031	1.1159
Pestivirus A	KX170186	VIPR_ALG4_1129879638_	1a	E2	V035	2007	-0.5969	0.6150	1.1147
Pestivirus A	KX170187	VIPR_ALG4_1129879640_	1a	E2	V042	2008	-0.5694	0.6033	1.1143
Pestivirus A	KX170188	VIPR_ALG4_1129879642_	1a	E2	V043	2008	-0.6150	0.6097	1.1143
Pestivirus A	KX170189	VIPR_ALG4_1129879644_	1a	E2	V052	2010	-0.5982	0.6082	1.1147
Pestivirus A	KX170190	VIPR_ALG4_1129879646_	1a	E2	V050	2009	-0.6570	0.6163	1.1145
Pestivirus A	KX170191	VIPR_ALG4_1129879648_	1a	E2	V046	2009	-0.6343	0.6304	1.1146
Pestivirus A	KX170192	VIPR_ALG4_1129879650_	1a	E2	V039	2008	-0.6043	0.6118	1.1154
Pestivirus A	KX170193	VIPR_ALG4_1129879652_	1a	E2	V041	2008	-0.5942	0.6139	1.1154
Pestivirus A	KX170194	VIPR_ALG4_1129879654_	1a	E2	V099	1998	-0.4919	0.6553	1.1204
Pestivirus A	KX170195	VIPR_ALG4_1129879656_	1a	E2	V048	2009	-0.6328	0.5843	1.1123
Pestivirus A	KX170196	VIPR_ALG4_1129879658_	1a	E2	V059	2004	-0.6022	0.6366	1.1174
Pestivirus A	EF101530	VIPR_ALG4_118498779_2	1b	E2	KE9	2007	-0.3815	0.6181	1.1293
Pestivirus A	DQ088995	VIPR_ALG4_145309048_2	1a	E2	Singer_Arg	1974	-0.8785	0.6366	1.1115
Pestivirus A	U63479	VIPR_ALG4_1518836_244	1b	E2	CP7	1987	-0.5111	0.5736	1.1348
Pestivirus A	U86600	VIPR_ALG4_2149469_246	1b	E2	ILLNC	1991	-0.5834	0.5171	1.1268

Species according to VIPRBRC	GenBank Accession	GenBank Protein Accession	Subgenotype	Protein	Strain Name	Collection Year	SVM Patho. Score	Vaxijen Antig. Score	Averged score of EMBOSS motifs
Pestivirus A	AF041040	VIPR_ALG4_2789677_244	1a	E2	Oregon	1960	-0.6520	0.6373	1.1181
Pestivirus A	M96751	VIPR_ALG4_289508_2462	1a	E2	UNKNOWN-M96751	1992	-0.6394	0.6204	1.1130
Pestivirus A	HQ174292	VIPR_ALG4_323145267_2	1a	E2	180	2010	-0.4871	0.6557	1.1171
Pestivirus A	M31182	VIPR_ALG4_323206_2462	1a	E2	UNKNOWN-M31182	1988	-0.6702	0.6545	1.1162
Pestivirus A	M96687	VIPR_ALG4_323230_2460	1b	E2	Osloss	1967	-0.4900	0.5685	1.1393
Pestivirus A	JN377417	VIPR_ALG4_359720341_1	1b	E2	M15.1	2009	-0.5012	0.5612	1.1285
Pestivirus A	JN377418	VIPR_ALG4_359720343_1	1b	E2	M15.2	2009	-0.5148	0.5620	1.1276
Pestivirus A	JN377419	VIPR_ALG4_359720345_1	1b	E2	M15.3	2009	-0.5023	0.5362	1.1293
Pestivirus A	JN377420	VIPR_ALG4_359720347_1	1b	E2	M15.4	2009	-0.5168	0.5624	1.1291
Pestivirus A	JN377421	VIPR_ALG4_359720349_1	1b	E2	M15.5	2009	-0.5168	0.5624	1.1291
Pestivirus A	JN377422	VIPR_ALG4_359720351_1	1b	E2	M15.6	2009	-0.4671	0.5636	1.1276
Pestivirus A	JN377423	VIPR_ALG4_359720353_1	1b	E2	M15.7	2009	-0.5148	0.5620	1.1276
Pestivirus A	JN377424	VIPR_ALG4_359720355_1	1b	E2	M181 22	2009	-0.6335	0.5620	1.1259
Pestivirus A	JN400273	VIPR_ALG4_363990275_2	1q	E2	SD0803	2008	-0.9084	0.5451	1.1209
Pestivirus A	AF091605	VIPR_ALG4_3661566_246	1a	E2	Oregon C24V	1960	-0.6026	0.6365	1.1215
Pestivirus A	JN644055	VIPR_ALG4_373939303_2	1b	E2	3156	2011	-0.5615	0.6445	1.1231
Pestivirus A	JN380080	VIPR_ALG4_378753653_2	1a	E2	6010	2010	-0.4336	0.6565	1.1185
Pestivirus A	JQ799141	VIPR_ALG4_390132765_2	1u	E2	M31182	2010	-0.5267	0.5484	1.1305
Pestivirus A	JX419397	VIPR_ALG4_404363562_2	1b	E2	UNKNOWN-JX419397	2008	-0.6305	0.6017	1.1276
Pestivirus A	JX419398	VIPR_ALG4_404363564_2	1b	E2	UNKNOWN-JX419398	2008	-0.6471	0.6065	1.1276
Pestivirus A	AF526381	VIPR_ALG4_42476348_24	1m	E2	ZM-95	1995	-0.6557	0.6141	1.1208
Pestivirus A	JX297512	VIPR_ALG4_459284067_2	1b	E2	10270	2007	-0.3744	0.5603	1.1308
Pestivirus A	JX297513	VIPR_ALG4_459284069_2	1b	E2	Aries	2005	-0.3827	0.5738	1.1339
Pestivirus A	JX297514	VIPR_ALG4_459284071_2	1b	E2	Columba	2005	-0.3656	0.5534	1.1334
Pestivirus A	JX297515	VIPR_ALG4_459284073_2	1b	E2	Corona	2005	-0.3170	0.5415	1.1334
Pestivirus A	JX297516	VIPR_ALG4_459284075_2	1b	E2	Gemini	2005	-0.3656	0.5534	1.1334
Pestivirus A	JX297517	VIPR_ALG4_459284077_2	1b	E2	Hercules	2006	-0.5477	0.6080	1.1172
Pestivirus A	JX297518	VIPR_ALG4_459284079_2	1b	E2	Leo	2006	-0.3777	0.5524	1.1334
Pestivirus A	JX297519	VIPR_ALG4_459284081_2	1b	E2	Lyra	2006	-0.3656	0.5534	1.1334
Pestivirus A	JX297520	VIPR_ALG4_459284083_2	1b	E2	Mars	2006	-0.3656	0.5534	1.1334
Pestivirus A	JX297521	VIPR_ALG4_459284085_2	1b	E2	Scorpius	2006	-0.3490	0.5500	1.1329
Pestivirus A	JX306011	VIPR_ALG4_459284087_2	1b	E2	Cepheus	2005	-0.4808	0.5401	1.1245
Pestivirus A	JX306012	VIPR_ALG4_459284089_2	1b	E2	Hamal	2006	-0.4708	0.5613	1.1245
Pestivirus A	JX306013	VIPR_ALG4_459284091_2	1b	E2	Kurhah	2006	-0.4708	0.5613	1.1245
Pestivirus A	JX306014	VIPR_ALG4_459284093_2	1b	E2	Naos	2006	-0.4708	0.5613	1.1245
Pestivirus A	KC853440	VIPR_ALG4_507144146_2	1k	E2	SuwaNcp	1993	-0.6638	0.6336	1.1249
Pestivirus A	KC853441	VIPR_ALG4_507144148_2	1k	E2	SuwaCp	1993	-0.6574	0.6298	1.1236
Pestivirus A	KC695810	VIPR_ALG4_507866685_2	1q	E2	camel-6	2010	-0.6716	0.6307	1.1266
Pestivirus A	KC695811	VIPR_ALG4_507866687_2	1q	E2	GS-3	2012	-0.8576	0.6403	1.1302
Pestivirus A	KC695812	VIPR_ALG4_507866689_2	1q	E2	HB-1	2012	-0.9038	0.5374	1.1254
Pestivirus A	KC695813	VIPR_ALG4_507866693_2	1c	E2	Bega-like	2012	-0.6907	0.6123	1.1214
Pestivirus A	KC695814	VIPR_ALG4_507866704_2	1b	E2	Av69 VEDEVAC	2011	-0.5228	0.6144	1.1316
Pestivirus A	KC695815	VIPR_ALG4_507866706_2	1a	E2	Av69 SD-1	2011	-0.7402	0.6369	1.1152
Pestivirus A	KC695816	VIPR_ALG4_507866709_2	1d	E2	cell-con-1	2012	-0.5979	0.6571	1.1318
Pestivirus A	KC757383	VIPR_ALG4_511775165_2	1d	E2	10JJ-SKR	2010	-0.4056	0.6510	1.1249
Pestivirus A	KC963967	VIPR_ALG4_530291194_2	1b	E2	12F004	2012	-0.5031	0.5819	1.1258
Pestivirus A	KF048835	VIPR_ALG4_542716379_1	1b	E2	USF02	2012	-0.5704	0.5719	1.1292

Species according to VIPRBRC	GenBank Accession	GenBank Protein Accession	Subgenotype	Protein	Strain Name	Collection Year	SVM Patho. Score	Vaxijen Antig. Score	Averged score of EMBOSS motifs
Pestivirus A	KF048836	VIPR_ALG4_542716381_1	1b	E2	QUAN2	2012	-0.5814	0.5381	1.1334
Pestivirus A	KF048837	VIPR_ALG4_542716383_1	1b	E2	GS151	2012	-0.4102	0.5701	1.1323
Pestivirus A	KF048838	VIPR_ALG4_542716385_1	1a	E2	GS24	2012	-0.8383	0.6402	1.1174
Pestivirus A	KF048839	VIPR_ALG4_542716387_1	1b	E2	DX51	2012	-0.5785	0.5963	1.1325
Pestivirus A	KF048840	VIPR_ALG4_542716389_1	1b	E2	USF12	2012	-0.5332	0.6457	1.1298
Pestivirus A	KF048841	VIPR_ALG4_542716391_1	1b	E2	SJZ01	2012	-0.5043	0.5574	1.1366
Pestivirus A	KF048842	VIPR_ALG4_542716393_1	1c	E2	GS35	2012	-0.3023	0.6627	1.1229
Pestivirus A	KF048843	VIPR_ALG4_542716395_1	1c	E2	JINAN01	2012	-0.6105	0.5666	1.1238
Pestivirus A	KF048844	VIPR_ALG4_542716397_1	1d	E2	ISO122	2012	-0.5979	0.6571	1.1318
Pestivirus A	KF048845	VIPR_ALG4_542716399_1	1m	E2	SH202	2012	-0.6520	0.5949	1.1176
Pestivirus A	KF048846	VIPR_ALG4_542716401_1	1m	E2	TJ43	2012	-0.9428	0.6012	1.1187
Pestivirus A	KF048847	VIPR_ALG4_542716403_1	1m	E2	HZ21	2012	-0.9561	0.6334	1.1316
Pestivirus A	KF048848	VIPR_ALG4_542716405_1	1o	E2	TJ41	2012	-0.8278	0.6452	1.1194
Pestivirus A	KF048849	VIPR_ALG4_542716407_1	1p	E2	LEI01	2012	-0.5429	0.6775	1.1299
Pestivirus A	KF048850	VIPR_ALG4_542716409_1	1p	E2	TJ142	2012	-0.9529	0.6346	1.1361
Pestivirus A	KF048851	VIPR_ALG4_542716411_1	1q	E2	ISO101	2012	-0.6290	0.6547	1.1284
Pestivirus A	KF048852	VIPR_ALG4_542716413_1	2a	E2	USF10	2012	-0.4888	0.5196	1.1187
Pestivirus A	KF048853	VIPR_ALG4_542716415_1	1b	E2	GS31	2012	-0.6353	0.5658	1.1139
Pestivirus A	AB894349	VIPR_ALG4_565410642_1	1b	E2	BVDV/Nakashibetsu/881/10	2010	-0.3732	0.5460	1.1313
Pestivirus A	AB896799	VIPR_ALG4_568213782_1	1a	E2	BVDV/Kamiyuubetsu/08/02	2002	-0.3788	0.6670	1.1173
Pestivirus A	AB896800	VIPR_ALG4_568213784_1	1a	E2	BVDV/Monbetsu/03/01-CP	2001	-0.6733	0.6554	1.1167
Pestivirus A	AB896801	VIPR_ALG4_568213786_1	1a	E2	BVDV/Monbetsu/205/04	2004	-0.6291	0.6062	1.1255
Pestivirus A	AB896802	VIPR_ALG4_568213788_1	1a	E2	BVDV/Oketo/277/04	2004	-0.5383	0.6894	1.1204
Pestivirus A	AB896803	VIPR_ALG4_568213790_1	1a	E2	BVDV/Betsukai/503/07	2007	-0.5934	0.6230	1.1282
Pestivirus A	AB896804	VIPR_ALG4_568213792_1	1a	E2	BVDV/Betsukai/669/08	2008	-0.7154	0.6599	1.1253
Pestivirus A	AB896805	VIPR_ALG4_568213794_1	1b	E2	BVDV/Yuubetsu/10/01	2001	-0.4424	0.5643	1.1315
Pestivirus A	AB896806	VIPR_ALG4_568213796_1	1b	E2	BVDV/Saroma/23/02	2002	-0.4871	0.5811	1.1336
Pestivirus A	AB896807	VIPR_ALG4_568213798_1	1b	E2	BVDV/Okoppe/89/01	2001	-0.3923	0.5475	1.1321
Pestivirus A	AB896808	VIPR_ALG4_568213800_1	1b	E2	BVDV/Nakasatsunai/583/07	2007	-0.6828	0.6099	1.1364
Pestivirus A	AB896809	VIPR_ALG4_568213802_1	1b	E2	BVDV/Nakasatsunai/719/09-CP	2009	-0.7381	0.6174	1.1325
Pestivirus A	AB896810	VIPR_ALG4_568213804_1	1b	E2	BVDV/Nakashibetsu/856/10	2010	-0.4145	0.5313	1.1325
Pestivirus A	AB896811	VIPR_ALG4_568213806_1	1b	E2	BVDV/Betsukai/884/10	2010	-0.4414	0.5387	1.1292
Pestivirus A	KF772785	VIPR_ALG4_575471151_2	1b	E2	CC13B	2013	-0.6742	0.5795	1.1274
Pestivirus A	KF856290	VIPR_ALG4_583844139_1	1b	E2	XJ	2013	-0.5865	0.6047	1.1332
Pestivirus A	KF896608	VIPR_ALG4_586616532_2	1c	E2	Bega-like	2012	-0.6907	0.6123	1.1214
Pestivirus A	KF835697	VIPR_ALG4_597437474_2	1b	E2	AU526	2013	-0.5345	0.5855	1.1312
Pestivirus A	KJ541471	VIPR_ALG4_633265982_2	1a	E2	GS5	2013	-0.5201	0.6582	1.1141
Pestivirus A	KJ689448	VIPR_ALG4_635172915_2	1b	E2	GX4	2012	-0.4821	0.6210	1.1313
Pestivirus A	KF501393	VIPR_ALG4_669206614_2	1b	E2	BVDV JL-1	2009	-0.4290	0.6088	1.1314
Pestivirus A	LC016729	VIPR_ALG4_740922466_1	1a	E2	BVDV/Akkeshi/710/09	2009	-0.6719	0.6596	1.1152
Pestivirus A	LC016730	VIPR_ALG4_740922468_1	1a	E2	BVDV/Hamatonbetsu/1191/13	2013	-0.6604	0.6647	1.1191
Pestivirus A	LC016731	VIPR_ALG4_740922470_1	1b	E2	BVDV/Hamanaka/646/08	2008	-0.8419	0.5921	1.1279
Pestivirus A	LC016732	VIPR_ALG4_740922472_1	1b	E2	BVDV/Setana/1103/12	2012	-0.3518	0.6315	1.1235
Pestivirus A	AJ133738	VIPR_ALG4_7960754_246	1a	E2	type 1	1963	-0.6702	0.6545	1.1162

Species according to VIPRBRC	GenBank Accession	GenBank Protein Accession	Subgenotype	Protein	Strain Name	Collection Year	SVM Patho. Score	Vaxijen Antig. Score	Averged score of EMBOSS motifs
Pestivirus A	KP941581	VIPR_ALG4_800924313_1	1b	E2	USMARC-51998	2014	-0.5543	0.5738	1.1301
Pestivirus A	KP941583	VIPR_ALG4_800924317_1	1b	E2	USMARC-53874	2014	-0.3408	0.6190	1.1422
Pestivirus A	KP941584	VIPR_ALG4_800924319_1	1a	E2	USMARC-53875	2014	-0.5696	0.6347	1.1178
Pestivirus A	KP941586	VIPR_ALG4_800924323_1	1a	E2	USMARC-55477	2014	-0.5613	0.6705	1.1208
Pestivirus A	KP941587	VIPR_ALG4_800924325_1	1b	E2	USMARC-55478	2014	-0.3824	0.5517	1.1449
Pestivirus A	KP941588	VIPR_ALG4_800924327_1	1b	E2	USMARC-55922	2014	-0.5046	0.6206	1.1348
Pestivirus A	KP941589	VIPR_ALG4_800924329_1	1b	E2	USMARC-55923	2014	-0.8147	0.5997	1.1312
Pestivirus A	KP941590	VIPR_ALG4_800924331_1	1b	E2	USMARC-55924	2014	-0.7336	0.5725	1.1290
Pestivirus A	KP941591	VIPR_ALG4_800924333_1	1b	E2	USMARC-55925	2014	-0.7019	0.5836	1.1329
Pestivirus A	KP941592	VIPR_ALG4_800924335_1	1b	E2	USMARC-55926	2014	-0.3084	0.5856	1.1299
Pestivirus A	KP313732	VIPR_ALG4_816850387_1	1e	E2	Carlito	2014	-0.8172	0.6210	1.1254
Pestivirus A	KR013753	VIPR_ALG4_871332680_1	1a	E2	WAX-N	1992	-0.5920	0.6664	1.1170
Pestivirus A	KR014249	VIPR_ALG4_887497284_1	1b	E2	Egy/Ismailia/2014	2014	-0.4162	0.5846	1.1294
Pestivirus A	KR029825	VIPR_ALG4_887497286_1	1b	E2	Egy/Ismailia/2014	2014	-0.4162	0.5846	1.1294
Pestivirus A	LC089875	VIPR_ALG4_939106262_1	1o	E2	IS26/01ncp	2001	-0.9702	0.6108	1.1190
Pestivirus A	LC089876	VIPR_ALG4_939106264_1	1n	E2	Shitara/02/06	2006	-0.2898	0.5869	1.1433
Pestivirus A	KR866116	VIPR_ALG4_941508008_1	1m	E2	SD-15	2015	-0.7578	0.5981	1.1202
Pestivirus A	KU200260	VIPR_ALG4_972905813_1	1b	E2	BE/061536/2014	2014	-0.4122	0.6330	1.1232
Pestivirus A	KX577637	VIPR_ALG4_AOR50934_1	1e	E2	SLO/2407/2006	2006	-0.8427	0.6484	1.1300
Pestivirus A	KX987157	VIPR_ALG4_APG30987_1	1f	E2	SLO/1170/2000	2000	-0.6248	0.5527	1.1268
Pestivirus A	KX857724	VIPR_ALG4_APZ85839_1	1i	E2	ACM/BR/2016	2016	-0.6169	0.5995	1.1217
Pestivirus A	KY849592	VIPR_ALG4_ART90617_1	1d	E2	SLO/2416/2002	2002	-0.3258	0.5812	1.1384
Pestivirus A	MF278651	VIPR_ALG4_ASW18434_1	1b	E2	XZ01	2016	-0.5228	0.6144	1.1316
Pestivirus A	MF278652	VIPR_ALG4_ASW18435_1	1b	E2	XZ02	2016	-0.5228	0.6144	1.1316
Pestivirus A	MF693403	VIPR_ALG4_ATG71375_1	1a	E2	UNKNOWN-MF693403	2016	-0.6107	0.6254	1.1218
Pestivirus A	KY964311	VIPR_ALG4_ATN39078_1	1b	E2	Y2	2014	-0.5359	0.5849	1.1232
Pestivirus A	MF172980	VIPR_ALG4_AVI10261_1_	1c	E2	GSTZ	2012	-0.8502	0.5974	1.1233
Pestivirus A	MH379638	VIPR_ALG4_AWW14171_	1a	E2	Ho916	1993	-0.8492	0.5733	1.1124
Pestivirus A	MG950344	VIPR_ALG4_AWW87346_	1b	E2	AU526	2014	-0.5231	0.5760	1.1290
Pestivirus A	MG950345	VIPR_ALG4_AWW87347_	1b	E2	B1	2015	-0.5436	0.5748	1.1312
Pestivirus A	MG950346	VIPR_ALG4_AWW87348_	1b	E2	B2	2015	-0.5794	0.5807	1.1312
Pestivirus A	MG950347	VIPR_ALG4_AWW87349_	1b	E2	B3	2015	-0.4778	0.5724	1.1302
Pestivirus A	MG950348	VIPR_ALG4_AWW87350_	1b	E2	B4	2015	-0.4778	0.5724	1.1302
Pestivirus A	MG950349	VIPR_ALG4_AWW87351_	1b	E2	B5	2015	-0.5041	0.5614	1.1290
Pestivirus A	MG950350	VIPR_ALG4_AWW87352_	1b	E2	B6	2015	-0.4778	0.5724	1.1302
Pestivirus A	MG950351	VIPR_ALG4_AWW87353_	1b	E2	O1	2015	-0.4834	0.5746	1.1306
Pestivirus A	MG950352	VIPR_ALG4_AWW87354_	1b	E2	O2	2015	-0.5174	0.5745	1.1285
Pestivirus A	MG950353	VIPR_ALG4_AWW87355_	1b	E2	O3	2015	-0.5627	0.5834	1.1285
Pestivirus A	MG950354	VIPR_ALG4_AWW87356_	1b	E2	O4	2015	-0.5627	0.5834	1.1285
Pestivirus A	MG950355	VIPR_ALG4_AWW87357_	1b	E2	O5	2015	-0.5627	0.5834	1.1285
Pestivirus A	MG950356	VIPR_ALG4_AWW87358_	1b	E2	O6	2015	-0.5614	0.5787	1.1302
Pestivirus A	MG950357	VIPR_ALG4_AWW87359_	1b	E2	B1A	2015	-0.5350	0.5699	1.1250
Pestivirus A	MG950358	VIPR_ALG4_AWW87360_	1b	E2	B2A	2016	-0.5389	0.5689	1.1278
Pestivirus A	MG950359	VIPR_ALG4_AWW87361_	1b	E2	B3A	2016	-0.4778	0.5724	1.1302
Pestivirus A	MG950360	VIPR_ALG4_AWW87362_	1b	E2	B4A	2016	-0.5695	0.5601	1.1283
Pestivirus A	MG950361	VIPR_ALG4_AWW87363_	1b	E2	B5A	2016	-0.4778	0.5724	1.1302
Pestivirus A	MG950362	VIPR_ALG4_AWW87364_	1b	E2	B6A	2016	-0.4778	0.5724	1.1302

Species according to VIPRBRC	GenBank Accession	GenBank Protein Accession	Subgenotype	Protein	Strain Name	Collection Year	SVM Patho. Score	Vaxijen Antig. Score	Averged score of EMBOSS motifs
Pestivirus A	MG950363	VIPR_ALG4_AWW87365_	1b	E2	O1A	2015	-0.5674	0.5884	1.1286
Pestivirus A	MG950364	VIPR_ALG4_AWW87366_	1b	E2	O2A	2015	-0.5459	0.5859	1.1266
Pestivirus A	MG950365	VIPR_ALG4_AWW87367_	1b	E2	O2B	2015	-0.5627	0.5834	1.1285
Pestivirus A	MG950366	VIPR_ALG4_AWW87368_	1b	E2	O4A	2015	-0.5937	0.5851	1.1302
Pestivirus A	MH311874	VIPR_ALG4_AWW87369_	1b	E2	B2A d168	2016	-0.5141	0.5792	1.1257
Pestivirus A	MH311875	VIPR_ALG4_AWW87370_	1b	E2	B3A d168	2016	-0.4493	0.5877	1.1311
Pestivirus A	MH311876	VIPR_ALG4_AWW87371_	1b	E2	B4A d84	2016	-0.4862	0.5629	1.1302
Pestivirus A	MH311877	VIPR_ALG4_AWW87372_	1b	E2	B4A d168	2016	-0.5226	0.5796	1.1302
Pestivirus A	MH311878	VIPR_ALG4_AWW87373_	1b	E2	B5A d84	2016	-0.4778	0.5724	1.1302
Pestivirus A	MH311879	VIPR_ALG4_AWW87374_	1b	E2	B5A d168	2016	-0.5226	0.5796	1.1302
Pestivirus A	MH311880	VIPR_ALG4_AWW87375_	1b	E2	B6A d84	2016	-0.4778	0.5724	1.1302
Pestivirus A	MH311881	VIPR_ALG4_AWW87376_	1b	E2	B6A d168	2016	-0.4778	0.5724	1.1302
Pestivirus A	MH379221	VIPR_ALG4_AWW87377_	1b	E2	P1	2017	-0.5626	0.5897	1.1285
Pestivirus A	MH379222	VIPR_ALG4_AWW87378_	1b	E2	P2	2017	-0.5678	0.5902	1.1285
Pestivirus A	MH379223	VIPR_ALG4_AWW87379_	1b	E2	P5	2017	-0.5626	0.5897	1.1285
Pestivirus A	MH379224	VIPR_ALG4_AWW87380_	1b	E2	P6	2017	-0.5626	0.5897	1.1285
Pestivirus A	MH379225	VIPR_ALG4_AWW87381_	1b	E2	P7	2017	-0.5626	0.5897	1.1285
Pestivirus A	MH379226	VIPR_ALG4_AWW87382_	1b	E2	P5A	2017	-0.5678	0.5902	1.1285
Pestivirus A	MH379227	VIPR_ALG4_AWW87383_	1b	E2	P5B	2017	-0.6110	0.5919	1.1292
Pestivirus A	MH379228	VIPR_ALG4_AWW87384_	1b	E2	P5C	2017	-0.5626	0.5897	1.1285
Pestivirus A	MH379229	VIPR_ALG4_AWW87385_	1b	E2	P5D	2017	-0.5678	0.5902	1.1285
Pestivirus A	MH379230	VIPR_ALG4_AWW87386_	1b	E2	P5F	2017	-0.5626	0.5897	1.1285
Pestivirus A	MH379231	VIPR_ALG4_AWW87387_	1b	E2	P7A	2018	-0.5216	0.6036	1.1308
Pestivirus A	MH379232	VIPR_ALG4_AWW87388_	1b	E2	P7C	2018	-0.5990	0.5918	1.1249
Pestivirus A	MH379233	VIPR_ALG4_AWW87389_	1b	E2	P7E	2018	-0.6300	0.5735	1.1318
Pestivirus A	MH379234	VIPR_ALG4_AWW87390_	1b	E2	P7F	2018	-0.5579	0.5882	1.1292
Pestivirus A	MH166806	VIPR_ALG4_AYA62524_1	1m	E2	XC	2015	-0.7648	0.6457	1.1231
Pestivirus A	MH490943	VIPR_ALG4_AZB53078_1	1b	E2	BVDV BJ-2016	2016	-0.5932	0.5903	1.1252
Pestivirus A	MH231153	VIPR_ALG4_AZQ00677_1	1b	E2	Nebraska	1990	-0.4770	0.5426	1.1268
Pestivirus A	AB078950	VIPR_ALG4_BAC55961_1	1j	E2	KS86-1ncp	1986	-0.8371	0.5987	1.1291
Pestivirus A	LC630446	VIPR_ALG4_BCV19807_1	1b	E2	BVDV/Ibaraki/32/15	2015	-0.6341	0.5899	1.1298
Pestivirus A	LC630447	VIPR_ALG4_BCV19808_1	1b	E2	BVDV/Ibaraki/36/15	2015	-0.5943	0.5818	1.1329
Pestivirus A	LC630448	VIPR_ALG4_BCV19809_1	1b	E2	BVDV/Gunma/01/16	2016	-0.4918	0.5719	1.1307
Pestivirus A	LC630449	VIPR_ALG4_BCV19810_1	1b	E2	BVDV/Gunma/03/16	2016	-0.5256	0.5652	1.1307
Pestivirus A	LC630450	VIPR_ALG4_BCV19811_1	1b	E2	BVDV/Gunma/05/16	2016	-0.3874	0.5587	1.1250
Pestivirus A	LC630451	VIPR_ALG4_BCV19812_1	1b	E2	BVDV/Gunma/01/17	2017	-0.4351	0.5415	1.1347
Pestivirus A	LC630452	VIPR_ALG4_BCV19813_1	1b	E2	BVDV/Gunma/02/17	2017	-0.4099	0.5411	1.1310
Pestivirus A	LC630453	VIPR_ALG4_BCV19814_1	1b	E2	BVDV/Gunma/03/17	2017	-0.5681	0.6056	1.1291
Pestivirus A	LC630454	VIPR_ALG4_BCV19815_1	1b	E2	BVDV/Gunma/06/17	2017	-0.4271	0.5950	1.1317
Pestivirus A	LC630455	VIPR_ALG4_BCV19816_1	1b	E2	BVDV/Gunma/07/17	2017	-0.4748	0.5872	1.1254
Pestivirus A	LC630456	VIPR_ALG4_BCV19817_1	1b	E2	BVDV/Gunma/11/17	2017	-0.3831	0.5404	1.1304
Pestivirus A	LC630457	VIPR_ALG4_BCV19818_1	1b	E2	BVDV/Gunma/01/18	2018	-0.4463	0.5326	1.1306
Pestivirus A	LC630458	VIPR_ALG4_BCV19819_1	1b	E2	BVDV/Gunma/04/18	2018	-0.6391	0.6133	1.1299
Pestivirus A	LC630459	VIPR_ALG4_BCV19820_1	1b	E2	BVDV/Gunma/12/18	2018	-0.3874	0.5587	1.1250
Pestivirus A	LC630460	VIPR_ALG4_BCV19821_1	1b	E2	BVDV/Gunma/13/18	2018	-0.3098	0.5242	1.1311
Pestivirus A	LC630461	VIPR_ALG4_BCV19822_1	1b	E2	BVDV/Gunma/14/18	2018	-0.4635	0.5800	1.1302
Pestivirus A	LC630462	VIPR_ALG4_BCV19823_1	1b	E2	BVDV/Gunma/19/18	2018	-0.3841	0.5682	1.1292

Species according to VIPRBRC	GenBank Accession	GenBank Protein Accession	Subgenotype	Protein	Strain Name	Collection Year	SVM Patho. Score	Vaxijen Antig. Score	Averged score of EMBOSS motifs
Pestivirus A	LC630463	VIPR_ALG4_BCV19824_1	1b	E2	BVDV/Gunma/22/18	2018	-0.3278	0.5504	1.1288
Pestivirus A	LC630464	VIPR_ALG4_BCV19825_1	1b	E2	BVDV/Gunma/23/18	2018	-0.3642	0.5207	1.1311
Pestivirus A	LC630466	VIPR_ALG4_BCV19826_1	1b	E2	BVDV/Gunma/05/19	2019	-0.4068	0.5439	1.1331
Pestivirus A	LC630467	VIPR_ALG4_BCV19827_1	1b	E2	BVDV/Gunma/07/19	2019	-0.3661	0.5896	1.1284
Pestivirus A	LC630468	VIPR_ALG4_BCV19828_1	1b	E2	BVDV/Gunma/11/19	2019	-0.3714	0.5623	1.1269
Pestivirus A	LC630469	VIPR_ALG4_BCV19829_1	1b	E2	BVDV/Gunma/15/19	2019	-0.3503	0.5498	1.1266
Pestivirus A	LC630470	VIPR_ALG4_BCV19830_1	1b	E2	BVDV/Okayama/11/18	2018	-0.5820	0.5338	1.1424
Pestivirus A	LC630471	VIPR_ALG4_BCV19831_1	1b	E2	BVDV/Okayama/21/19	2019	-0.4555	0.5606	1.1363
Pestivirus A	LC630472	VIPR_ALG4_BCV19832_1	1b	E2	BVDV/Okayama/24/20	2020	-0.4547	0.5480	1.1341
Pestivirus A	LC630473	VIPR_ALG4_BCV19833_1	1b	E2	BVDV/Kumamoto/01/20	2020	-0.6509	0.5377	1.1275
Pestivirus A	LC630474	VIPR_ALG4_BCV19834_1	1b	E2	BVDV/Oita/01/18	2018	-0.5913	0.5808	1.1359
Pestivirus A	LC630475	VIPR_ALG4_BCV19835_1	1b	E2	BVDV/Oita/07/18	2018	-0.3515	0.5288	1.1305
Pestivirus A	LC630476	VIPR_ALG4_BCV19836_1	1b	E2	BVDV/Oita/10/18	2018	-0.5207	0.5406	1.1287
Pestivirus A	LC630465	VIPR_ALG4_BCW91505_1	1b	E2	BVDV/Gunma/24/18	2018	-0.3886	0.5335	1.1304
Pestivirus A	LC648252	VIPR_ALG4_BDA82227_1	1b	E2	BVDV/Taiki/I_22	2019	-0.5555	0.6108	1.1271
Pestivirus A	LC648254	VIPR_ALG4_BDA82229_1	1b	E2	BVDV/Taiki/A_3	2018	-0.5700	0.5950	1.1304
Pestivirus A	LC648255	VIPR_ALG4_BDA82230_1	1b	E2	BVDV/Taiki/A_2	2018	-0.6019	0.5820	1.1286
Pestivirus A	LC648256	VIPR_ALG4_BDA82231_1	1b	E2	BVDV/Taiki/A_1	2018	-0.5700	0.5950	1.1304
Pestivirus A	LC648258	VIPR_ALG4_BDA82233_1	1b	E2	BVDV/Shihoro/O_40	2020	-0.3072	0.5443	1.1318
Pestivirus A	LC648259	VIPR_ALG4_BDA82234_1	1b	E2	BVDV/Shihoro/O_39	2020	-0.3325	0.5369	1.1318
Pestivirus A	LC648260	VIPR_ALG4_BDA82235_1	1b	E2	BVDV/Shihoro/B_41	2020	-0.3912	0.5440	1.1335
Pestivirus A	LC648261	VIPR_ALG4_BDA82236_1	1b	E2	BVDV/Shihoro/B_6	2018	-0.4808	0.5760	1.1251
Pestivirus A	LC648263	VIPR_ALG4_BDA82238_1	1b	E2	BVDV/Otohuke/J_26	2019	-0.3401	0.5463	1.1312
Pestivirus A	LC648264	VIPR_ALG4_BDA82239_1	1b	E2	BVDV/Otohuke/J_25	2019	-0.3540	0.5545	1.1312
Pestivirus A	LC648265	VIPR_ALG4_BDA82240_1	1b	E2	BVDV/Otohuke/J_23	2019	-0.3261	0.5472	1.1301
Pestivirus A	LC648266	VIPR_ALG4_BDA82241_1	1b	E2	BVDV/Kamishihoro/L_29	2020	-0.3716	0.5383	1.1312
Pestivirus A	LC648267	VIPR_ALG4_BDA82242_1	1b	E2	BVDV/Kamishihoro/L_28	2020	-0.3716	0.5383	1.1312
Pestivirus A	LC648268	VIPR_ALG4_BDA82243_1	1b	E2	BVDV/Honbetu/M_33	2020	-0.3672	0.5140	1.1276
Pestivirus A	LC648269	VIPR_ALG4_BDA82244_1	1b	E2	BVDV/Honbetu/M_30	2020	-0.3473	0.5250	1.1312
Pestivirus A	LC648270	VIPR_ALG4_BDA82245_1	1b	E2	BVDV/Hiroo/G_20	2019	-0.3506	0.6077	1.1332
Pestivirus A	LC648271	VIPR_ALG4_BDA82246_1	1b	E2	BVDV/Hiroo/G_19	2019	-0.2660	0.5869	1.1327
Pestivirus A	LC648272	VIPR_ALG4_BDA82247_1	1b	E2	BVDV/Hiroo/G_18	2019	-0.2660	0.5869	1.1327
Pestivirus A	LC648273	VIPR_ALG4_BDA82248_1	1b	E2	BVDV/Hiroo/G_17	2019	-0.3669	0.6106	1.1346
Pestivirus A	LC648274	VIPR_ALG4_BDA82249_1	1b	E2	BVDV/Hiroo/E_14	2018	-0.3672	0.5315	1.1339
Pestivirus A	LC648275	VIPR_ALG4_BDA82250_1	1b	E2	BVDV/Hiroo/D_12	2018	-0.3672	0.5315	1.1339
Pestivirus A	LC648276	VIPR_ALG4_BDA82251_1	1b	E2	BVDV/Hiroo/D_13	2018	-0.2679	0.5358	1.1390
Pestivirus A	LC648277	VIPR_ALG4_BDA82252_1	1b	E2	BVDV/Hiroo/D_10	2018	-0.3638	0.5304	1.1350
Pestivirus A	LC648278	VIPR_ALG4_BDA82253_1	1b	E2	BVDV/Hiroo/D_9	2018	-0.3207	0.5318	1.1339
Pestivirus A	LC648279	VIPR_ALG4_BDA82254_1	1b	E2	BVDV/Hiroo/D_8	2018	-0.3207	0.5318	1.1339
Pestivirus A	LC648387	VIPR_ALG4_BDB04029_1	1b	E2	BVDV/Rikubetsu/1283/14	2014	-0.3716	0.5383	1.1312
Pestivirus A	LC648388	VIPR_ALG4_BDB04030_1	1b	E2	BVDV/Rikubetsu/1284/14	2014	-0.3716	0.5383	1.1312
Pestivirus A	LC648389	VIPR_ALG4_BDB04031_1	1b	E2	BVDV/Makubetsu/1294/14	2014	-0.3716	0.5383	1.1312
Pestivirus A	LC648390	VIPR_ALG4_BDB04032_1	1b	E2	BVDV/Oozora/1331/16	2016	-0.3716	0.5383	1.1312
Pestivirus A	LC648391	VIPR_ALG4_BDB04033_1	1b	E2	BVDV/Obihiro/1364/16	2016	-0.3716	0.5383	1.1312
Pestivirus A	LC648392	VIPR_ALG4_BDB04034_1	1b	E2	BVDV/Kamishihoro/1385/16	2016	-0.3716	0.5383	1.1312
Pestivirus A	LC648393	VIPR_ALG4_BDB04035_1	1b	E2	BVDV/Sarabetsu/1517/17	2017	-0.3614	0.5427	1.1321

Species according to VIPRBRC	GenBank Accession	GenBank Protein Accession	Subgenotype	Protein	Strain Name	Collection Year	SVM Patho. Score	Vaxijen Antig. Score	Averged score of EMBOSS motifs
Pestivirus A	LC648394	VIPR_ALG4_BDB04036_1	1b	E2	BVDV/Teshio/1600/17	2017	-0.3623	0.5292	1.1312
Pestivirus A	LC648395	VIPR_ALG4_BDB04037_1	1b	E2	BVDV/Saruhutsu/1607/17	2017	-0.4897	0.5585	1.1294
Pestivirus A	LC648396	VIPR_ALG4_BDB04038_1	1b	E2	BVDV/Setana/1733/17	2017	-0.4008	0.6047	1.1238
Pestivirus A	LC648397	VIPR_ALG4_BDB04039_1	1b	E2	BVDV/Hokuto/1735/18	2018	-0.3866	0.5439	1.1312
Pestivirus A	LC648398	VIPR_ALG4_BDB04040_1	1b	E2	BVDV/Shihoro/1754/18	2018	-0.3716	0.5383	1.1312
Pestivirus A	LC648399	VIPR_ALG4_BDB04041_1	1b	E2	BVDV/Nayoro/1857/19	2019	-0.3807	0.5421	1.1312
Pestivirus A	LC648400	VIPR_ALG4_BDB04042_1	1b	E2	BVDV/Bie/1860/20	2020	-0.3716	0.5383	1.1312
Pestivirus A	LC648401	VIPR_ALG4_BDB04043_1	1b	E2	BVDV/Higashikagura/1862/20	2020	-0.4053	0.5413	1.1312
Pestivirus A	MH899941	VIPR_ALG4_QCE30388_1	1b	E2	SLO/3301/2014	2014	-0.5071	0.6502	1.1324
Pestivirus A	MH899942	VIPR_ALG4_QCE30389_1	1e	E2	SLO/33529/2015	2015	-0.8050	0.6510	1.1271
Pestivirus A	MH899943	VIPR_ALG4_QCE30390_1	1f	E2	SLO/1361/2014	2014	-0.7010	0.6138	1.1334
Pestivirus A	MH899944	VIPR_ALG4_QCE30391_1	1f	E2	SLO/28537/2017	2017	-0.7417	0.6497	1.1231
Pestivirus A	MH899945	VIPR_ALG4_QCE30392_1	1h	E2	SLO/1883/2013	2013	-0.8754	0.5728	1.1291
Pestivirus A	MK102095	VIPR_ALG4_QCQ84262_1	1q	E2	20170226	2017	-0.8414	0.6059	1.1305
Pestivirus A	MK170073	VIPR_ALG4_QDC12613_1	1c	E2	BSC-1	2017	-0.7272	0.6292	1.1264
Pestivirus A	MK170074	VIPR_ALG4_QDC12614_1	1a	E2	BSC-2	2017	-0.6747	0.6015	1.1184
Pestivirus A	MK170075	VIPR_ALG4_QDC12615_1	1a	E2	BSC-3	2017	-0.4393	0.6410	1.1160
Pestivirus A	MK170076	VIPR_ALG4_QDC12616_1	1a	E2	BSC-4	2017	-0.6747	0.6015	1.1184
Pestivirus A	MK170077	VIPR_ALG4_QDC12617_1	1a	E2	BSC-5	2017	-0.6971	0.6116	1.1171
Pestivirus A	MK170078	VIPR_ALG4_QDC12618_1	1a	E2	BSC-6	2017	-0.6747	0.6015	1.1184
Pestivirus A	MK509774	VIPR_ALG4_QEK23510_1	1b	E2	BVD1b-JH	2008	-0.6464	0.6003	1.1265
Pestivirus A	MK775204	VIPR_ALG4_QFX66041_1	1i	E2	CA2006	2006	-0.4775	0.5616	1.1142
Pestivirus A	MN188073	VIPR_ALG4_QGZ19414_1	1a	E2	PI34	2017	-0.6007	0.6135	1.1127
Pestivirus A	MN188074	VIPR_ALG4_QGZ19415_1	1b	E2	PI285	2017	-0.5084	0.5565	1.1269
Pestivirus A	MT079816	VIPR_ALG4_QIM55913_1	1c	E2	GXNN1	2018	-0.7856	0.7282	1.1290
Pestivirus A	MN623291	VIPR_ALG4_QLL27013_1	1m	E2	NX2019/01	2019	-0.9930	0.6272	1.1323
Pestivirus A	MW014286	VIPR_ALG4_QPJ59878_1	1b	E2	GXSS01	2018	-0.6789	0.6124	1.1294
Pestivirus A	MW014287	VIPR_ALG4_QPJ59879_1	1b	E2	GXSS02	2018	-0.5613	0.5883	1.1312
Pestivirus A	MW014288	VIPR_ALG4_QPJ59880_1	1b	E2	GXSS03	2018	-0.5453	0.5895	1.1312
Pestivirus A	MT977117	VIPR_ALG4_QRZ20359_1	1b	E2	BVDV 1b IT16/5	2016	-0.5182	0.5936	1.1335
Pestivirus A	MT977118	VIPR_ALG4_QRZ20360_1	1b	E2	BVDV 1b IT16/439	2016	-0.4699	0.5644	1.1361
Pestivirus A	MW013505	VIPR_ALG4_QVE48461_1	1p	E2	3877	2009	-0.6812	0.6421	1.1300
Pestivirus A	MT654137	VIPR_ALG4_QVK82311_1	1a	E2	20-8536	2020	-0.6073	0.6673	1.1140
Pestivirus A	LT837585	VIPR_ALG4_SLV80196_1	1r	E2	UNKNOWN-LT837585	2012	-0.7108	0.6100	1.1269
Pestivirus A	MW054933	VIPR_ALG4_UEC94252_1	1f	E2	LA/230/14	2014	-0.6553	0.5632	1.1259
Pestivirus A	MW054934	VIPR_ALG4_UEC94253_1	1f	E2	LA/87/05	2005	-0.6909	0.5858	1.1241
Pestivirus A	MW054935	VIPR_ALG4_UEC94254_1	1k	E2	TO/197/11	2011	-0.5070	0.6187	1.1192
Pestivirus A	MW054936	VIPR_ALG4_UEC94255_1	1g	E2	UM/111/06	2006	-0.9773	0.6425	1.1285
Pestivirus A	MW054937	VIPR_ALG4_UEC94256_1	1k	E2	SA/158/09	2009	-0.5766	0.5709	1.1241
Pestivirus A	MW054938	VIPR_ALG4_UEC94257_1	1k	E2	SA/159/09	2009	-0.6095	0.5747	1.1224
Pestivirus A	MW054939	VIPR_ALG4_UEC94258_1	1f	E2	LO/151/09	2009	-0.5563	0.5841	1.1200
Pestivirus A	MW054940	VIPR_ALG4_UEC94259_1	1e	E2	MA/101/05	2005	-0.9532	0.6406	1.1375
Pestivirus A	MW250796	VIPR_ALG4_UEC94260_1	1i	E2	58-1	2008	-0.9040	0.6478	1.1176
Pestivirus A	MW250797	VIPR_ALG4_UEC94261_1	1i	E2	58-2	2008	-0.9040	0.6478	1.1176
Pestivirus A	MW250798	VIPR_ALG4_UEC94262_1	1a	E2	62-2	2008	-0.6425	0.6673	1.1274
Pestivirus A	MW250799	VIPR_ALG4_UEC94263_1	1a	E2	63-1	2008	-0.6120	0.6745	1.1297

Species according to VIPRBRC	GenBank Accession	GenBank Protein Accession	Subgenotype	Protein	Strain Name	Collection Year	SVM Patho. Score	Vaxijen Antig. Score	Averged score of EMBOSS motifs
Pestivirus A	MW250800	VIPR_ALG4_UEC94264_1	1d	E2	67-1	2008	-0.4459	0.6120	1.1359
Pestivirus A	MW250801	VIPR_ALG4_UEC94265_1	1d	E2	67-2	2008	-0.3735	0.6040	1.1359
Pestivirus A	MW250802	VIPR_ALG4_UEC94266_1	1e	E2	68-1	2008	-0.8618	0.6812	1.1259
Pestivirus A	MW250803	VIPR_ALG4_UEC94267_1	1i	E2	69-1	2008	-0.8034	0.7025	1.1194
Pestivirus A	MW655625	VIPR_ALG4_UEC94268_1	1h	E2	CH-04-01b	2004	-0.7257	0.5453	1.1200
Pestivirus A	MW655626	VIPR_ALG4_UEC94269_1	1e	E2	Maria	2004	-0.5127	0.6271	1.1227
Pestivirus A	MW655627	VIPR_ALG4_UEC94270_1	1e	E2	R2000-95	1995	-0.9067	0.6505	1.1306
Pestivirus A	MW655628	VIPR_ALG4_UEC94271_1	1k	E2	R3230-95	1995	-0.5811	0.6276	1.1257
Pestivirus A	MW655629	VIPR_ALG4_UEC94272_1	1h	E2	R3572-90	1990	-0.5782	0.6553	1.1244
Pestivirus A	MW655630	VIPR_ALG4_UEC94273_1	1k	E2	R5013-96	1996	-0.5331	0.6786	1.1281
Pestivirus A	MW655631	VIPR_ALG4_UEC94274_1	1e	E2	S03-1175	2003	-0.5591	0.6139	1.1299
Pestivirus A	MW655632	VIPR_ALG4_UEC94275_1	1h	E2	SM09-20	2002	-0.6557	0.5392	1.1213
Pestivirus A	MW713361	VIPR_ALG4_UEC94276_1	1a	E2	BoAEC1190	2007	-0.8036	0.6530	1.1169
Pestivirus A	MW713362	VIPR_ALG4_UEC94277_1	1b	E2	PI819	2017	-0.4106	0.5781	1.1265
Pestivirus A	MW732738	VIPR_ALG4_UEC94278_1	1a	E2	PI407	2015	-0.6450	0.6418	1.1212
Pestivirus A	MW732739	VIPR_ALG4_UEC94279_1	1a	E2	YandaSpl	1993	-0.4206	0.6613	1.1145
Pestivirus A	MZ188972	VIPR_ALG4_UML14262_1	1q	E2	HB-1	2020	-0.8534	0.5764	1.1220
Pestivirus A	MZ484396	VIPR_ALG4_URN48308_1	1a	E2	Hubei	2017	-0.7080	0.6488	1.1151
Pestivirus A	ON337882	VIPR_ALG4_USZ80113_1_	1c	E2	NM2103	2021	-0.5580	0.6382	1.1318
Pestivirus A	MZ484396	URN48308.1	1a	P7	Hubei	2017	-1.0222	0.2152	1.2390
Pestivirus A	KU159365	VIPR_ALG4_1039262063_	1a	P7	USII-S15	2015	-0.9232	0.1795	1.2295
Pestivirus A	KU756226	VIPR_ALG4_1072900294_	1b	P7	HJ-1	2010	-1.2915	0.1879	1.2155
Pestivirus A	KT943518	VIPR_ALG4_1093530908_	1d	P7	BJ1201	2012	-0.8628	0.1400	1.2345
Pestivirus A	LT631725	VIPR_ALG4_1112914034_	1h	P7	UM/126/07	2007	-2.5539	0.3141	1.2345
Pestivirus A	EF101530	VIPR_ALG4_118498779_3	1b	P7	KE9	2007	-1.1748	0.1700	1.2155
Pestivirus A	DQ088995	VIPR_ALG4_145309048_3	1a	P7	Singer_Arg	1974	-0.9232	0.1795	1.2295
Pestivirus A	U63479	VIPR_ALG4_1518836_356	1b	P7	CP7	1987	-1.1007	0.2470	1.2155
Pestivirus A	U86600	VIPR_ALG4_2149469_358	1b	P7	ILLNC	1991	-0.6091	0.1729	1.2155
Pestivirus A	AF041040	VIPR_ALG4_2789677_356	1a	P7	Oregon	1960	-1.0018	0.1717	1.2295
Pestivirus A	M96751	VIPR_ALG4_289508_3584	1a	P7	UNKNOWN-M96751	1992	-0.7712	0.1402	1.2205
Pestivirus A	HQ174292	VIPR_ALG4_323145267_3	1a	P7	180	2010	-0.8881	0.2064	1.2295
Pestivirus A	M31182	VIPR_ALG4_323206_3584	1a	P7	UNKNOWN-M31182	1988	-1.0222	0.2152	1.2390
Pestivirus A	M96687	VIPR_ALG4_323230_3585	1b	P7	Osloss	1967	-0.3447	0.2277	1.2155
Pestivirus A	JN400273	VIPR_ALG4_363990275_3	1q	P7	SD0803	2008	-1.0642	0.2368	1.2460
Pestivirus A	AF091605	VIPR_ALG4_3661566_358	1a	P7	Oregon C24V	1960	-1.0018	0.1717	1.2295
Pestivirus A	JN644055	VIPR_ALG4_373939303_3	1b	P7	3156	2011	-0.7712	0.1402	1.2205
Pestivirus A	JN380080	VIPR_ALG4_378753653_3	1a	P7	6010	2010	-0.8881	0.2064	1.2295
Pestivirus A	JQ799141	VIPR_ALG4_390132765_3	1u	P7	M31182	2010	-0.9333	0.1741	1.1846
Pestivirus A	JX419397	VIPR_ALG4_404363562_3	1b	P7	UNKNOWN-JX419397	2008	-0.6766	0.1807	1.2155
Pestivirus A	JX419398	VIPR_ALG4_404363564_3	1b	P7	UNKNOWN-JX419398	2008	-0.6766	0.1807	1.2155
Pestivirus A	AF526381	VIPR_ALG4_42476348_35	1m	P7	ZM-95	1995	-1.1230	0.1811	1.2645
Pestivirus A	JX297512	VIPR_ALG4_459284067_3	1b	P7	10270	2007	-1.1345	0.1996	1.2155
Pestivirus A	JX297513	VIPR_ALG4_459284069_3	1b	P7	Aries	2005	-1.1774	0.1959	1.2155
Pestivirus A	JX297514	VIPR_ALG4_459284071_3	1b	P7	Columba	2005	-1.1774	0.1959	1.2155
Pestivirus A	JX297515	VIPR_ALG4_459284073_3	1b	P7	Corona	2005	-1.1774	0.1959	1.2155
Pestivirus A	JX297516	VIPR_ALG4_459284075_3	1b	P7	Gemini	2005	-1.1774	0.1959	1.2155
Pestivirus A	JX297517	VIPR_ALG4_459284077_3	1b	P7	Hercules	2006	-1.3946	0.2607	1.2105

Species according to VIPRBRC	GenBank Accession	GenBank Protein Accession	Subgenotype	Protein	Strain Name	Collection Year	SVM Patho. Score	Vaxijen Antig. Score	Averged score of EMBOSS motifs
Pestivirus A	JX297518	VIPR_ALG4_459284079_1	1b	P7	Leo	2006	-1.1774	0.1959	1.2155
Pestivirus A	JX297519	VIPR_ALG4_459284081_1	1b	P7	Lyra	2006	-1.1846	0.2022	1.2155
Pestivirus A	JX297520	VIPR_ALG4_459284083_1	1b	P7	Mars	2006	-1.1774	0.1959	1.2155
Pestivirus A	JX297521	VIPR_ALG4_459284085_1	1b	P7	Scorpius	2006	-1.1774	0.1959	1.2155
Pestivirus A	KC853440	VIPR_ALG4_507144146_1	1k	P7	SuwaNcp	1993	-1.8699	0.1494	1.2345
Pestivirus A	KC853441	VIPR_ALG4_507144148_1	1k	P7	SuwaCp	1993	-1.8699	0.1494	1.2345
Pestivirus A	KC695810	VIPR_ALG4_507866685_1	1q	P7	camel-6	2010	-0.0603	0.1687	1.2460
Pestivirus A	KC695814	VIPR_ALG4_507866704_1	1b	P7	Av69 VEDEVAC	2011	-0.9197	0.1934	1.2250
Pestivirus A	KC695815	VIPR_ALG4_507866706_1	1a	P7	Av69 SD-1	2011	-0.3684	0.1281	1.2205
Pestivirus A	KC757383	VIPR_ALG4_511775165_1	1d	P7	10JJ-SKR	2010	-0.8264	0.1381	1.2345
Pestivirus A	KC963967	VIPR_ALG4_530291194_1	1b	P7	12F004	2012	-1.3506	0.1782	1.2155
Pestivirus A	KF772785	VIPR_ALG4_575471151_1	1b	P7	CC13B	2013	-0.1146	0.2533	1.2155
Pestivirus A	KF896608	VIPR_ALG4_586616532_1	1c	P7	Bega-like	2012	-1.6815	0.1896	1.2460
Pestivirus A	KF835697	VIPR_ALG4_597437474_1	1b	P7	AU526	2013	-1.1988	0.2235	1.2155
Pestivirus A	KJ541471	VIPR_ALG4_633265982_1	1a	P7	GS5	2013	-0.9216	0.1893	1.2225
Pestivirus A	KJ689448	VIPR_ALG4_635172915_1	1b	P7	GX4	2012	-1.3209	0.1947	1.2250
Pestivirus A	KF501393	VIPR_ALG4_669206614_1	1b	P7	BVDV JL-1	2009	-0.5786	0.1732	1.2155
Pestivirus A	AJ133738	VIPR_ALG4_7960754_358	1a	P7	type 1	1963	-1.0222	0.2152	1.2390
Pestivirus A	KP941581	VIPR_ALG4_800924313_1	1b	P7	USMARC-51998	2014	-1.2150	0.2022	1.2320
Pestivirus A	KP941583	VIPR_ALG4_800924317_1	1b	P7	USMARC-53874	2014	-0.4702	0.1742	1.2155
Pestivirus A	KP941584	VIPR_ALG4_800924319_1	1a	P7	USMARC-53875	2014	-1.6446	0.2138	1.2295
Pestivirus A	KP941586	VIPR_ALG4_800924323_1	1a	P7	USMARC-55477	2014	-1.7108	0.1858	1.2295
Pestivirus A	KP941587	VIPR_ALG4_800924325_1	1b	P7	USMARC-55478	2014	-1.2196	0.1317	1.2155
Pestivirus A	KP941588	VIPR_ALG4_800924327_1	1b	P7	USMARC-55922	2014	-0.5255	0.1790	1.2155
Pestivirus A	KP941589	VIPR_ALG4_800924329_1	1b	P7	USMARC-55923	2014	-0.7734	0.1991	1.2155
Pestivirus A	KP941590	VIPR_ALG4_800924331_1	1b	P7	USMARC-55924	2014	-0.3919	0.1907	1.2140
Pestivirus A	KP941591	VIPR_ALG4_800924333_1	1b	P7	USMARC-55925	2014	-1.0535	0.2003	1.2305
Pestivirus A	KP941592	VIPR_ALG4_800924335_1	1b	P7	USMARC-55926	2014	-1.3610	0.1771	1.2320
Pestivirus A	KP313732	VIPR_ALG4_816850387_1	1e	P7	Carlito	2014	-0.6616	0.2291	1.2575
Pestivirus A	KR013753	VIPR_ALG4_871332680_1	1a	P7	WAX-N	1992	-0.8183	0.1994	1.2295
Pestivirus A	KR029825	VIPR_ALG4_887497286_1	1b	P7	Egy/Ismailia/2014	2014	-0.6049	0.1811	1.2155
Pestivirus A	LC089875	VIPR_ALG4_939106262_1	1o	P7	IS26/01ncp	2001	-1.7334	0.1157	1.2370
Pestivirus A	LC089876	VIPR_ALG4_939106264_1	1n	P7	Shitara/02/06	2006	0.0557	0.1123	1.2155
Pestivirus A	KR866116	VIPR_ALG4_941508008_1	1m	P7	SD-15	2015	-1.3168	0.1737	1.2645
Pestivirus A	KU200260	VIPR_ALG4_972905813_1	1b	P7	BE/061536/2014	2014	-0.9556	0.2628	1.1913
Pestivirus A	KX577637	VIPR_ALG4_AOR50934_1	1e	P7	SLO/2407/2006	2006	0.0855	0.2507	1.2340
Pestivirus A	KX987157	VIPR_ALG4_APG30987_1	1f	P7	SLO/1170/2000	2000	-1.5170	0.2315	1.2480
Pestivirus A	KX857724	VIPR_ALG4_APZ85839_1	1i	P7	ACM/BR/2016	2016	-1.3065	0.1501	1.2315
Pestivirus A	KY849592	VIPR_ALG4_ART90617_1	1d	P7	SLO/2416/2002	2002	-0.4218	0.1508	1.2305
Pestivirus A	MF278651	VIPR_ALG4_ASW18434_1	1b	P7	XZ01	2016	-0.9197	0.1934	1.2250
Pestivirus A	MF278652	VIPR_ALG4_ASW18435_1	1b	P7	XZ02	2016	-0.7000	0.2110	1.2120
Pestivirus A	MF693403	VIPR_ALG4_ATG71375_1	1a	P7	UNKNOWN-MF693403	2016	-0.9216	0.1893	1.2225
Pestivirus A	KY964311	VIPR_ALG4_ATN39078_1	1b	P7	Y2	2014	-0.1277	0.2181	1.2155
Pestivirus A	MF172980	VIPR_ALG4_AVI10261_1_	1c	P7	GSTZ	2012	-1.5449	0.1346	1.2390
Pestivirus A	MH379638	VIPR_ALG4_AWW14171_	1a	P7	Ho916	1993	-1.2154	0.1113	1.2315
Pestivirus A	MG950344	VIPR_ALG4_AWW87346_	1b	P7	AU526	2014	-1.1988	0.2235	1.2155
Pestivirus A	MG950345	VIPR_ALG4_AWW87347_	1b	P7	B1	2015	-1.1988	0.2235	1.2155

Species according to VIPRBRC	GenBank Accession	GenBank Protein Accession	Subgenotype	Protein	Strain Name	Collection Year	SVM Patho. Score	Vaxijen Antig. Score	Averged score of EMBOSS motifs
Pestivirus A	MG950346	VIPR_ALG4_AWW87348_	1b	P7	B2	2015	-1.1988	0.2235	1.2155
Pestivirus A	MG950347	VIPR_ALG4_AWW87349_	1b	P7	B3	2015	-1.1988	0.2235	1.2155
Pestivirus A	MG950348	VIPR_ALG4_AWW87350_	1b	P7	B4	2015	-1.1988	0.2235	1.2155
Pestivirus A	MG950349	VIPR_ALG4_AWW87351_	1b	P7	B5	2015	-1.1988	0.2235	1.2155
Pestivirus A	MG950350	VIPR_ALG4_AWW87352_	1b	P7	B6	2015	-1.1988	0.2235	1.2155
Pestivirus A	MG950351	VIPR_ALG4_AWW87353_	1b	P7	O1	2015	-1.1988	0.2235	1.2155
Pestivirus A	MG950352	VIPR_ALG4_AWW87354_	1b	P7	O2	2015	-1.1988	0.2235	1.2155
Pestivirus A	MG950353	VIPR_ALG4_AWW87355_	1b	P7	O3	2015	-1.1988	0.2235	1.2155
Pestivirus A	MG950354	VIPR_ALG4_AWW87356_	1b	P7	O4	2015	-1.1988	0.2235	1.2155
Pestivirus A	MG950355	VIPR_ALG4_AWW87357_	1b	P7	O5	2015	-1.1988	0.2235	1.2155
Pestivirus A	MG950356	VIPR_ALG4_AWW87358_	1b	P7	O6	2015	-1.1988	0.2235	1.2155
Pestivirus A	MG950357	VIPR_ALG4_AWW87359_	1b	P7	B1A	2015	-1.1988	0.2235	1.2155
Pestivirus A	MG950358	VIPR_ALG4_AWW87360_	1b	P7	B2A	2016	-1.1988	0.2235	1.2155
Pestivirus A	MG950359	VIPR_ALG4_AWW87361_	1b	P7	B3A	2016	-1.1988	0.2235	1.2155
Pestivirus A	MG950360	VIPR_ALG4_AWW87362_	1b	P7	B4A	2016	-1.1988	0.2235	1.2155
Pestivirus A	MG950361	VIPR_ALG4_AWW87363_	1b	P7	B5A	2016	-1.1988	0.2235	1.2155
Pestivirus A	MG950362	VIPR_ALG4_AWW87364_	1b	P7	B6A	2016	-1.1988	0.2235	1.2155
Pestivirus A	MG950363	VIPR_ALG4_AWW87365_	1b	P7	O1A	2015	-1.0235	0.1872	1.2155
Pestivirus A	MG950364	VIPR_ALG4_AWW87366_	1b	P7	O2A	2015	-1.1988	0.2235	1.2155
Pestivirus A	MG950365	VIPR_ALG4_AWW87367_	1b	P7	O2B	2015	-1.1988	0.2235	1.2155
Pestivirus A	MG950366	VIPR_ALG4_AWW87368_	1b	P7	O4A	2015	-1.1988	0.2235	1.2155
Pestivirus A	MH311874	VIPR_ALG4_AWW87369_	1b	P7	B2A d168	2016	-1.1988	0.2235	1.2155
Pestivirus A	MH311875	VIPR_ALG4_AWW87370_	1b	P7	B3A d168	2016	-1.1988	0.2235	1.2155
Pestivirus A	MH311876	VIPR_ALG4_AWW87371_	1b	P7	B4A d84	2016	-1.1988	0.2235	1.2155
Pestivirus A	MH311877	VIPR_ALG4_AWW87372_	1b	P7	B4A d168	2016	-1.1988	0.2235	1.2155
Pestivirus A	MH311878	VIPR_ALG4_AWW87373_	1b	P7	B5A d84	2016	-1.1988	0.2235	1.2155
Pestivirus A	MH311879	VIPR_ALG4_AWW87374_	1b	P7	B5A d168	2016	-1.1988	0.2235	1.2155
Pestivirus A	MH311880	VIPR_ALG4_AWW87375_	1b	P7	B6A d84	2016	-1.1988	0.2235	1.2155
Pestivirus A	MH311881	VIPR_ALG4_AWW87376_	1b	P7	B6A d168	2016	-1.1988	0.2235	1.2155
Pestivirus A	MH379221	VIPR_ALG4_AWW87377_	1b	P7	P1	2017	-1.1988	0.2235	1.2155
Pestivirus A	MH379222	VIPR_ALG4_AWW87378_	1b	P7	P2	2017	-1.1988	0.2235	1.2155
Pestivirus A	MH379223	VIPR_ALG4_AWW87379_	1b	P7	P5	2017	-1.1988	0.2235	1.2155
Pestivirus A	MH379224	VIPR_ALG4_AWW87380_	1b	P7	P6	2017	-1.1988	0.2235	1.2155
Pestivirus A	MH379225	VIPR_ALG4_AWW87381_	1b	P7	P7	2017	-1.1988	0.2235	1.2155
Pestivirus A	MH379226	VIPR_ALG4_AWW87382_	1b	P7	P5A	2017	-1.1988	0.2235	1.2155
Pestivirus A	MH379227	VIPR_ALG4_AWW87383_	1b	P7	P5B	2017	-1.1988	0.2235	1.2155
Pestivirus A	MH379228	VIPR_ALG4_AWW87384_	1b	P7	P5C	2017	-1.9017	0.2231	1.2155
Pestivirus A	MH379229	VIPR_ALG4_AWW87385_	1b	P7	P5D	2017	-1.1988	0.2235	1.2155
Pestivirus A	MH379230	VIPR_ALG4_AWW87386_	1b	P7	P5F	2017	-1.9017	0.2231	1.2155
Pestivirus A	MH379231	VIPR_ALG4_AWW87387_	1b	P7	P7A	2018	-1.1988	0.2235	1.2155
Pestivirus A	MH379232	VIPR_ALG4_AWW87388_	1b	P7	P7C	2018	-1.1988	0.2235	1.2155
Pestivirus A	MH379233	VIPR_ALG4_AWW87389_	1b	P7	P7E	2018	-1.1988	0.2235	1.2155
Pestivirus A	MH379234	VIPR_ALG4_AWW87390_	1b	P7	P7F	2018	-1.1988	0.2235	1.2155
Pestivirus A	MH166806	VIPR_ALG4_AYA62524_1	1m	P7	XC	2015	-1.1475	0.2639	1.2415
Pestivirus A	MH490943	VIPR_ALG4_AZB53078_1	1b	P7	BVDV BJ-2016	2016	-0.7267	0.1897	1.2330
Pestivirus A	MH231153	VIPR_ALG4_AZQ00677_1	1b	P7	Nebraska	1990	-1.4742	0.2196	1.2225
Pestivirus A	AB078950	VIPR_ALG4_BAC55961_1	1j	P7	KS86-1ncp	1986	-0.8982	0.2030	1.2160

Species according to VIPRBRC	GenBank Accession	GenBank Protein Accession	Subgenotype	Protein	Strain Name	Collection Year	SVM Patho. Score	Vaxijen Antig. Score	Averged score of EMBOSS motifs
Pestivirus A	MH899941	VIPR_ALG4_QCE30388_1	1b	P7	SLO/3301/2014	2014	-1.1118	0.1804	1.2320
Pestivirus A	MH899942	VIPR_ALG4_QCE30389_1	1e	P7	SLO/33529/2015	2015	0.0855	0.2507	1.2340
Pestivirus A	MH899943	VIPR_ALG4_QCE30390_1	1f	P7	SLO/1361/2014	2014	-1.2243	0.1899	1.2345
Pestivirus A	MH899944	VIPR_ALG4_QCE30391_1	1f	P7	SLO/28537/2017	2017	-1.6034	0.1876	1.2480
Pestivirus A	MH899945	VIPR_ALG4_QCE30392_1	1h	P7	SLO/1883/2013	2013	-1.6203	0.2332	1.1770
Pestivirus A	MK102095	VIPR_ALG4_QCQ84262_1	1q	P7	20170226	2017	-1.8397	0.1491	1.2550
Pestivirus A	MK509774	VIPR_ALG4_QEK23510_1	1b	P7	BVD1b-JH	2008	-0.6501	0.2107	1.2140
Pestivirus A	MK775204	VIPR_ALG4_QFX66041_1	1i	P7	CA2006	2006	-1.5701	0.0954	1.2480
Pestivirus A	MN188073	VIPR_ALG4_QGZ19414_1	1a	P7	PI34	2017	-1.2501	0.1674	1.2295
Pestivirus A	MN188074	VIPR_ALG4_QGZ19415_1	1b	P7	PI285	2017	-0.7063	0.2411	1.2240
Pestivirus A	MT079816	VIPR_ALG4_QIM55913_1	1c	P7	GXNN1	2018	-1.2666	0.1343	1.2345
Pestivirus A	MN623291	VIPR_ALG4_QLL27013_1	1m	P7	NX2019/01	2019	-0.9434	0.1763	1.2920
Pestivirus A	MW014286	VIPR_ALG4_QPJ59878_1	1b	P7	GXSS01	2018	-1.3209	0.1947	1.2250
Pestivirus A	MW014287	VIPR_ALG4_QPJ59879_1	1b	P7	GXSS02	2018	-1.2028	0.2030	1.2250
Pestivirus A	MW014288	VIPR_ALG4_QPJ59880_1	1b	P7	GXSS03	2018	-1.2028	0.2030	1.2250
Pestivirus A	MT977117	VIPR_ALG4_QRZ20359_1	1b	P7	BVDV 1b IT16/5	2016	-0.2022	0.2222	1.2155
Pestivirus A	MT977118	VIPR_ALG4_QRZ20360_1	1b	P7	BVDV 1b IT16/439	2016	-0.2022	0.2222	1.2155
Pestivirus A	MT654137	VIPR_ALG4_QVK82311_1	1a	P7	20-8536	2020	-0.9909	0.1179	1.2185
Pestivirus A	LT837585	VIPR_ALG4_SLV80196_1	1r	P7	UNKNOWN-LT837585	2012	-2.4155	0.0824	1.2295
Pestivirus A	MW054933	VIPR_ALG4_UEC94252_1	1f	P7	LA/230/14	2014	-1.5170	0.2315	1.2480
Pestivirus A	MW054934	VIPR_ALG4_UEC94253_1	1f	P7	LA/87/05	2005	-1.7236	0.2250	1.2480
Pestivirus A	MW054935	VIPR_ALG4_UEC94254_1	1k	P7	TO/197/11	2011	-2.0817	0.1304	1.2370
Pestivirus A	MW054936	VIPR_ALG4_UEC94255_1	1g	P7	UM/111/06	2006	-1.9875	0.1526	1.1743
Pestivirus A	MW054937	VIPR_ALG4_UEC94256_1	1k	P7	SA/158/09	2009	-1.8699	0.1494	1.2345
Pestivirus A	MW054938	VIPR_ALG4_UEC94257_1	1k	P7	SA/159/09	2009	-1.8699	0.1494	1.2345
Pestivirus A	MW054939	VIPR_ALG4_UEC94258_1	1f	P7	LO/151/09	2009	-1.5039	0.2401	1.2480
Pestivirus A	MW054940	VIPR_ALG4_UEC94259_1	1e	P7	MA/101/05	2005	0.1205	0.2620	1.2225
Pestivirus A	MW250796	VIPR_ALG4_UEC94260_1	1i	P7	58-1	2008	-0.8146	0.1032	1.2295
Pestivirus A	MW250797	VIPR_ALG4_UEC94261_1	1i	P7	58-2	2008	-0.8146	0.1032	1.2295
Pestivirus A	MW250798	VIPR_ALG4_UEC94262_1	1a	P7	62-2	2008	-1.1270	0.1443	1.2295
Pestivirus A	MW250799	VIPR_ALG4_UEC94263_1	1a	P7	63-1	2008	-0.7632	0.1946	1.2295
Pestivirus A	MW250800	VIPR_ALG4_UEC94264_1	1d	P7	67-1	2008	-0.6247	0.1480	1.2305
Pestivirus A	MW250801	VIPR_ALG4_UEC94265_1	1d	P7	67-2	2008	-0.6247	0.1480	1.2305
Pestivirus A	MW250802	VIPR_ALG4_UEC94266_1	1e	P7	68-1	2008	-0.5143	0.2033	1.2575
Pestivirus A	MW250803	VIPR_ALG4_UEC94267_1	1i	P7	69-1	2008	-0.8928	0.1422	1.2295
Pestivirus A	MW655625	VIPR_ALG4_UEC94268_1	1h	P7	CH-04-01b	2004	-2.0127	0.3306	1.2345
Pestivirus A	MW655626	VIPR_ALG4_UEC94269_1	1e	P7	Maria	2004	-1.7790	0.2592	1.2575
Pestivirus A	MW655627	VIPR_ALG4_UEC94270_1	1e	P7	R2000-95	1995	0.2090	0.2486	1.2175
Pestivirus A	MW655628	VIPR_ALG4_UEC94271_1	1k	P7	R3230-95	1995	-1.8700	0.1485	1.2345
Pestivirus A	MW655629	VIPR_ALG4_UEC94272_1	1h	P7	R3572-90	1990	-2.4179	0.2604	1.2345
Pestivirus A	MW655630	VIPR_ALG4_UEC94273_1	1k	P7	R5013-96	1996	-1.8699	0.1494	1.2345
Pestivirus A	MW655631	VIPR_ALG4_UEC94274_1	1e	P7	S03-1175	2003	-1.4735	0.1113	1.2505
Pestivirus A	MW655632	VIPR_ALG4_UEC94275_1	1h	P7	SM09-20	2002	-2.5539	0.3141	1.2345
Pestivirus A	MW713361	VIPR_ALG4_UEC94276_1	1a	P7	BoAEC1190	2007	-1.0434	0.1239	1.2295
Pestivirus A	MW713362	VIPR_ALG4_UEC94277_1	1b	P7	PI819	2017	-1.1988	0.2235	1.2155
Pestivirus A	MW732738	VIPR_ALG4_UEC94278_1	1a	P7	PI407	2015	-1.7561	0.1515	1.2033
Pestivirus A	MW732739	VIPR_ALG4_UEC94279_1	1a	P7	YandaSpl	1993	-0.9729	0.1351	1.2295

Species according to VIPRBRC	GenBank Accession	GenBank Protein Accession	Subgenotype	Protein	Strain Name	Collection Year	SVM Patho. Score	Vaxijen Antig. Score	Averged score of EMBOSS motifs
Pestivirus A	MZ188972	VIPR_ALG4_UML14262_1	1q	P7	HB-1	2020	-0.4818	0.1948	1.2180
Pestivirus A	MZ484396	VIPR_ALG4_URN48308_1	1a	P7	Hubei	2017	-1.0222	0.2152	1.2390
Pestivirus A	ON337882	VIPR_ALG4_USZ80113_1	1c	P7	NM2103	2021	-1.6639	0.1457	1.2390
Pestivirus A	KU159365	VIPR_ALG4_1039262063	1a	NS3	USII-S15	2015	-0.5226	0.5838	1.1288
Pestivirus A	KU756226	VIPR_ALG4_1072900294	1b	NS3	HJ-1	2010	-0.5870	0.5891	1.1304
Pestivirus A	KT943518	VIPR_ALG4_1093530908	1d	NS3	BJ1201	2012	-0.6469	0.5899	1.1289
Pestivirus A	LT631725	VIPR_ALG4_1112914034	1h	NS3	UM/126/07	2007	-0.6717	0.5943	1.1291
Pestivirus A	KX170422	VIPR_ALG4_1129880110	1b	NS3	V015	2001	-0.5717	0.5871	1.1324
Pestivirus A	KX170423	VIPR_ALG4_1129880112	1a	NS3	V056	2009	-0.5723	0.5915	1.1324
Pestivirus A	KX170424	VIPR_ALG4_1129880114	1b	NS3	V075	2011	-0.5487	0.5956	1.1300
Pestivirus A	KX170425	VIPR_ALG4_1129880116	1b	NS3	V060	2004	-0.5261	0.5903	1.1277
Pestivirus A	KX170426	VIPR_ALG4_1129880118	1b	NS3	V100	1997	-0.5412	0.5807	1.1295
Pestivirus A	KX170427	VIPR_ALG4_1129880120	1b	NS3	V070	2007	-0.5462	0.5847	1.1302
Pestivirus A	KX170428	VIPR_ALG4_1129880122	1b	NS3	V031	2006	-0.5272	0.5860	1.1286
Pestivirus A	KX170429	VIPR_ALG4_1129880124	1b	NS3	V087	2006	-0.5443	0.5863	1.1289
Pestivirus A	KX170430	VIPR_ALG4_1129880126	1b	NS3	V045	2009	-0.5297	0.5865	1.1271
Pestivirus A	KX170431	VIPR_ALG4_1129880128	1b	NS3	V078	2012	-0.5120	0.5861	1.1287
Pestivirus A	KX170432	VIPR_ALG4_1129880130	1b	NS3	V036	2007	-0.5312	0.5812	1.1321
Pestivirus A	KX170433	VIPR_ALG4_1129880132	1a	NS3	V048	2009	-0.5549	0.5851	1.1290
Pestivirus A	KX170434	VIPR_ALG4_1129880134	1b	NS3	V098	1999	-0.5783	0.5770	1.1313
Pestivirus A	KX170435	VIPR_ALG4_1129880136	1b	NS3	V020	2005	-0.5431	0.5839	1.1302
Pestivirus A	KX170436	VIPR_ALG4_1129880138	1b	NS3	V029	2006	-0.5372	0.5872	1.1290
Pestivirus A	KX170437	VIPR_ALG4_1129880140	1a	NS3	V026	2006	-0.5579	0.5869	1.1294
Pestivirus A	KX170438	VIPR_ALG4_1129880142	1a	NS3	V027	2006	-0.5579	0.5869	1.1294
Pestivirus A	KX170439	VIPR_ALG4_1129880144	1a	NS3	V091	2003	-0.5201	0.5834	1.1293
Pestivirus A	KX170440	VIPR_ALG4_1129880146	1a	NS3	V007	2000	-0.5106	0.5851	1.1288
Pestivirus A	KX170441	VIPR_ALG4_1129880148	1a	NS3	V013	2001	-0.5106	0.5851	1.1288
Pestivirus A	KX170442	VIPR_ALG4_1129880150	1a	NS3	V033	2007	-0.5106	0.5851	1.1288
Pestivirus A	KX170443	VIPR_ALG4_1129880152	1a	NS3	V049	2009	-0.5106	0.5851	1.1288
Pestivirus A	KX170444	VIPR_ALG4_1129880154	1a	NS3	V067	2006	-0.5106	0.5851	1.1288
Pestivirus A	KX170445	VIPR_ALG4_1129880156	1a	NS3	V073	2011	-0.5106	0.5851	1.1288
Pestivirus A	KX170446	VIPR_ALG4_1129880158	1a	NS3	V074	2010	-0.5106	0.5851	1.1288
Pestivirus A	KX170447	VIPR_ALG4_1129880160	1a	NS3	V077	2012	-0.5106	0.5851	1.1288
Pestivirus A	KX170448	VIPR_ALG4_1129880162	1a	NS3	V080	2009	-0.5106	0.5851	1.1288
Pestivirus A	KX170449	VIPR_ALG4_1129880164	1a	NS3	V034	2007	-0.5106	0.5851	1.1288
Pestivirus A	KX170450	VIPR_ALG4_1129880166	1a	NS3	V022	2006	-0.5300	0.5811	1.1291
Pestivirus A	KX170451	VIPR_ALG4_1129880168	1a	NS3	V092	2004	-0.5139	0.5870	1.1276
Pestivirus A	KX170452	VIPR_ALG4_1129880170	1a	NS3	V054	2013	-0.4887	0.5799	1.1290
Pestivirus A	KX170453	VIPR_ALG4_1129880172	1a	NS3	V011	2001	-0.5233	0.5810	1.1310
Pestivirus A	KX170454	VIPR_ALG4_1129880174	1a	NS3	V012	2001	-0.5226	0.5843	1.1288
Pestivirus A	KX170455	VIPR_ALG4_1129880176	1a	NS3	V006	2000	-0.5482	0.5835	1.1288
Pestivirus A	KX170456	VIPR_ALG4_1129880178	1a	NS3	V057	2009	-0.5421	0.5868	1.1276
Pestivirus A	KX170457	VIPR_ALG4_1129880180	1a	NS3	V001	1999	-0.5236	0.5851	1.1303
Pestivirus A	KX170458	VIPR_ALG4_1129880182	1a	NS3	V035	2007	-0.5456	0.5934	1.1285
Pestivirus A	KX170459	VIPR_ALG4_1129880184	1a	NS3	V040	2008	-0.5593	0.5796	1.1297
Pestivirus A	KX170460	VIPR_ALG4_1129880186	1a	NS3	V052	2010	-0.5518	0.5899	1.1297
Pestivirus A	KX170461	VIPR_ALG4_1129880188	1a	NS3	V050	2009	-0.5518	0.5899	1.1297

Species according to VIPRBRC	GenBank Accession	GenBank Protein Accession	Subgenotype	Protein	Strain Name	Collection Year	SVM Patho. Score	Vaxijen Antig. Score	Averged score of EMBOSS motifs
Pestivirus A	KX170462	VIPR_ALG4_1129880190_	1a	NS3	V046	2009	-0.5518	0.5899	1.1297
Pestivirus A	KX170463	VIPR_ALG4_1129880192_	1a	NS3	V039	2008	-0.5518	0.5899	1.1297
Pestivirus A	KX170464	VIPR_ALG4_1129880194_	1a	NS3	V041	2008	-0.5518	0.5899	1.1297
Pestivirus A	KX170465	VIPR_ALG4_1129880196_	1a	NS3	V043	2008	-0.5681	0.5907	1.1297
Pestivirus A	KX170466	VIPR_ALG4_1129880198_	1a	NS3	V042	2008	-0.5518	0.5899	1.1297
Pestivirus A	KX170467	VIPR_ALG4_1129880200_	1a	NS3	V008	2000	-0.5333	0.5853	1.1297
Pestivirus A	KX170468	VIPR_ALG4_1129880202_	1a	NS3	V009	2000	-0.5333	0.5853	1.1297
Pestivirus A	KX170469	VIPR_ALG4_1129880204_	1a	NS3	V010	2001	-0.5333	0.5853	1.1297
Pestivirus A	KX170470	VIPR_ALG4_1129880206_	1a	NS3	V083	2008	-0.5084	0.5834	1.1272
Pestivirus A	KX170471	VIPR_ALG4_1129880208_	1a	NS3	V059	2004	-0.5247	0.5893	1.1274
Pestivirus A	KX170472	VIPR_ALG4_1129880210_	1a	NS3	V099	1998	-0.5566	0.5848	1.1313
Pestivirus A	EF101530	VIPR_ALG4_118498779_5	1b	NS3	KE9	2007	-0.4988	0.5841	1.1275
Pestivirus A	DQ088995	VIPR_ALG4_145309048_5	1a	NS3	Singer_Arg	1974	-0.5106	0.5851	1.1288
Pestivirus A	U63479	VIPR_ALG4_1518836_516	1b	NS3	CP7	1987	-0.5372	0.5853	1.1291
Pestivirus A	U86600	VIPR_ALG4_2149469_515	1b	NS3	ILLNC	1991	-0.6401	0.5822	1.1282
Pestivirus A	AF041040	VIPR_ALG4_2789677_513	1a	NS3	Oregon	1960	-0.5247	0.5893	1.1274
Pestivirus A	M96751	VIPR_ALG4_289508_5153	1a	NS3	UNKNOWN-M96751	1992	-0.5194	0.5871	1.1285
Pestivirus A	HQ174292	VIPR_ALG4_323145267_4	1a	NS3	180	2010	-0.4568	0.5833	1.1282
Pestivirus A	M31182	VIPR_ALG4_323206_5423	1a	NS3	UNKNOWN-M31182	1988	-0.5595	0.5852	1.1271
Pestivirus A	M96687	VIPR_ALG4_323230_5382	1b	NS3	Osloss	1967	-0.5806	0.5829	1.1273
Pestivirus A	JN400273	VIPR_ALG4_363990275_5	1q	NS3	SD0803	2008	-0.5536	0.5885	1.1268
Pestivirus A	AF091605	VIPR_ALG4_3661566_515	1a	NS3	Oregon C24V	1960	-0.5247	0.5893	1.1274
Pestivirus A	JN644055	VIPR_ALG4_373939303_4	1b	NS3	3156	2011	-0.5593	0.5838	1.1258
Pestivirus A	JN380080	VIPR_ALG4_378753653_4	1a	NS3	6010	2010	-0.4059	0.5810	1.1273
Pestivirus A	JQ799141	VIPR_ALG4_390132765_5	1u	NS3	M31182	2010	-0.6140	0.5866	1.1193
Pestivirus A	JX419397	VIPR_ALG4_404363562_5	1b	NS3	UNKNOWN-JX419397	2008	-0.6165	0.5835	1.1273
Pestivirus A	JX419398	VIPR_ALG4_404363564_5	1b	NS3	UNKNOWN-JX419398	2008	-0.5888	0.5853	1.1252
Pestivirus A	AF526381	VIPR_ALG4_42476348_51	1m	NS3	ZM-95	1995	-0.6142	0.5943	1.1345
Pestivirus A	JX297512	VIPR_ALG4_459284067_4	1b	NS3	10270	2007	-0.5373	0.5837	1.1303
Pestivirus A	JX297513	VIPR_ALG4_459284069_4	1b	NS3	Aries	2005	-0.5750	0.5872	1.1293
Pestivirus A	JX297514	VIPR_ALG4_459284071_4	1b	NS3	Columba	2005	-0.5639	0.5819	1.1290
Pestivirus A	JX297515	VIPR_ALG4_459284073_4	1b	NS3	Corona	2005	-0.5639	0.5819	1.1290
Pestivirus A	JX297516	VIPR_ALG4_459284075_4	1b	NS3	Gemini	2005	-0.5376	0.5847	1.1287
Pestivirus A	JX297517	VIPR_ALG4_459284077_4	1b	NS3	Hercules	2006	-0.5299	0.5871	1.1285
Pestivirus A	JX297518	VIPR_ALG4_459284079_4	1b	NS3	Leo	2006	-0.5520	0.5825	1.1290
Pestivirus A	JX297519	VIPR_ALG4_459284081_4	1b	NS3	Lyra	2006	-0.5889	0.5879	1.1280
Pestivirus A	JX297520	VIPR_ALG4_459284083_4	1b	NS3	Mars	2006	-0.5755	0.5867	1.1290
Pestivirus A	JX297521	VIPR_ALG4_459284085_4	1b	NS3	Scorpius	2006	-0.5889	0.5879	1.1280
Pestivirus A	KC853440	VIPR_ALG4_507144146_5	1k	NS3	SuwaNcp	1993	-0.5152	0.5944	1.1301
Pestivirus A	KC853441	VIPR_ALG4_507144148_5	1k	NS3	SuwaCp	1993	-0.4848	0.5873	1.1301
Pestivirus A	KC695810	VIPR_ALG4_507866685_5	1q	NS3	camel-6	2010	-0.5349	0.5868	1.1253
Pestivirus A	KC695814	VIPR_ALG4_507866704_5	1b	NS3	Av69 VEDEVAC	2011	-0.5635	0.5846	1.1258
Pestivirus A	KC757383	VIPR_ALG4_511775165_5	1d	NS3	10JJ-SKR	2010	-0.6477	0.5898	1.1277
Pestivirus A	KC963967	VIPR_ALG4_530291194_5	1b	NS3	12F004	2012	-0.5434	0.5855	1.1290
Pestivirus A	KF772785	VIPR_ALG4_575471151_5	1b	NS3	CC13B	2013	-0.5384	0.5854	1.1287
Pestivirus A	KF896608	VIPR_ALG4_586616532_5	1c	NS3	Bega-like	2012	-0.5041	0.5900	1.1315
Pestivirus A	KF835697	VIPR_ALG4_597437474_5	1b	NS3	AU526	2013	-0.5626	0.5869	1.1259

Species according to VIPRBRC	GenBank Accession	GenBank Protein Accession	Subgenotype	Protein	Strain Name	Collection Year	SVM Patho. Score	Vaxijen Antig. Score	Averged score of EMBOSS motifs
Pestivirus A	KJ541471	VIPR_ALG4_633265982_	1a	NS3	GS5	2013	-0.4777	0.5819	1.1316
Pestivirus A	KJ689448	VIPR_ALG4_635172915_	1b	NS3	GX4	2012	-0.5572	0.5847	1.1258
Pestivirus A	KF501393	VIPR_ALG4_669206614_	1b	NS3	BVDV JL-1	2009	-0.5390	0.5983	1.1308
Pestivirus A	AJ133738	VIPR_ALG4_7960754_54	1a	NS3	type 1	1963	-0.5393	0.5867	1.1268
Pestivirus A	KP941581	VIPR_ALG4_800924313_	1b	NS3	USMARC-51998	2014	-0.5431	0.5839	1.1302
Pestivirus A	KP941583	VIPR_ALG4_800924317_	1b	NS3	USMARC-53874	2014	-0.5396	0.5850	1.1290
Pestivirus A	KP941584	VIPR_ALG4_800924319_	1a	NS3	USMARC-53875	2014	-0.4277	0.5799	1.1275
Pestivirus A	KP941586	VIPR_ALG4_800924323_	1a	NS3	USMARC-55477	2014	-0.5279	0.5876	1.1288
Pestivirus A	KP941587	VIPR_ALG4_800924325_	1b	NS3	USMARC-55478	2014	-0.5206	0.5901	1.1247
Pestivirus A	KP941588	VIPR_ALG4_800924327_	1b	NS3	USMARC-55922	2014	-0.5580	0.5859	1.1290
Pestivirus A	KP941589	VIPR_ALG4_800924329_	1b	NS3	USMARC-55923	2014	-0.5580	0.5859	1.1290
Pestivirus A	KP941590	VIPR_ALG4_800924331_	1b	NS3	USMARC-55924	2014	-0.5725	0.5875	1.1287
Pestivirus A	KP941591	VIPR_ALG4_800924333_	1b	NS3	USMARC-55925	2014	-0.5614	0.5839	1.1328
Pestivirus A	KP941592	VIPR_ALG4_800924335_	1b	NS3	USMARC-55926	2014	-0.5349	0.5891	1.1262
Pestivirus A	KP313732	VIPR_ALG4_816850387_	1e	NS3	Carlito	2014	-0.5852	0.5904	1.1265
Pestivirus A	KR029825	VIPR_ALG4_887497286_	1b	NS3	Egy/Ismailia/2014	2014	-0.5438	0.5773	1.1302
Pestivirus A	LC089875	VIPR_ALG4_939106262_	1o	NS3	IS26/01ncp	2001	-0.6668	0.5868	1.1248
Pestivirus A	LC089876	VIPR_ALG4_939106264_	1n	NS3	Shitara/02/06	2006	-0.5704	0.5896	1.1327
Pestivirus A	KR866116	VIPR_ALG4_941508008_	1m	NS3	SD-15	2015	-0.5862	0.5921	1.1345
Pestivirus A	KU200260	VIPR_ALG4_972905813_	1b	NS3	BE/061536/2014	2014	-0.4830	0.5938	1.1311
Pestivirus A	KX577637	VIPR_ALG4_AOR50934_1	1e	NS3	SLO/2407/2006	2006	-0.4836	0.5890	1.1266
Pestivirus A	KX987157	VIPR_ALG4_APG30987_1	1f	NS3	SLO/1170/2000	2000	-0.5753	0.6029	1.1280
Pestivirus A	KX857724	VIPR_ALG4_APZ85839_1	1i	NS3	ACM/BR/2016	2016	-0.4910	0.5947	1.1310
Pestivirus A	KY849592	VIPR_ALG4_ART90617_1	1d	NS3	SLO/2416/2002	2002	-0.6266	0.5891	1.1247
Pestivirus A	MF278651	VIPR_ALG4_ASW18434_1	1b	NS3	XZ01	2016	-0.5471	0.5832	1.1230
Pestivirus A	MF278652	VIPR_ALG4_ASW18435_1	1b	NS3	XZ02	2016	-0.5489	0.5801	1.1261
Pestivirus A	MF693403	VIPR_ALG4_ATG71375_1	1a	NS3	UNKNOWN-MF693403	2016	-0.3113	0.5460	1.1291
Pestivirus A	KY964311	VIPR_ALG4_ATN39078_1	1b	NS3	Y2	2014	-0.5616	0.5849	1.1302
Pestivirus A	MF172980	VIPR_ALG4_AVI10261_1	1c	NS3	GSTZ	2012	-0.5034	0.5931	1.1302
Pestivirus A	MH379638	VIPR_ALG4_AWW14171_	1a	NS3	Ho916	1993	-0.5438	0.5835	1.1301
Pestivirus A	MG950344	VIPR_ALG4_AWW87346_	1b	NS3	AU526	2014	-0.5626	0.5869	1.1259
Pestivirus A	MG950345	VIPR_ALG4_AWW87347_	1b	NS3	B1	2015	-0.5626	0.5869	1.1259
Pestivirus A	MG950346	VIPR_ALG4_AWW87348_	1b	NS3	B2	2015	-0.5626	0.5869	1.1259
Pestivirus A	MG950347	VIPR_ALG4_AWW87349_	1b	NS3	B3	2015	-0.5633	0.5807	1.1259
Pestivirus A	MG950348	VIPR_ALG4_AWW87350_	1b	NS3	B4	2015	-0.5633	0.5807	1.1259
Pestivirus A	MG950349	VIPR_ALG4_AWW87351_	1b	NS3	B5	2015	-0.5908	0.5815	1.1259
Pestivirus A	MG950350	VIPR_ALG4_AWW87352_	1b	NS3	B6	2015	-0.5633	0.5807	1.1259
Pestivirus A	MG950351	VIPR_ALG4_AWW87353_	1b	NS3	O1	2015	-0.5626	0.5869	1.1259
Pestivirus A	MG950352	VIPR_ALG4_AWW87354_	1b	NS3	O2	2015	-0.5626	0.5869	1.1259
Pestivirus A	MG950353	VIPR_ALG4_AWW87355_	1b	NS3	O3	2015	-0.5710	0.5877	1.1259
Pestivirus A	MG950354	VIPR_ALG4_AWW87356_	1b	NS3	O4	2015	-0.5710	0.5877	1.1259
Pestivirus A	MG950355	VIPR_ALG4_AWW87357_	1b	NS3	O5	2015	-0.5710	0.5877	1.1259
Pestivirus A	MG950356	VIPR_ALG4_AWW87358_	1b	NS3	O6	2015	-0.5710	0.5877	1.1259
Pestivirus A	MG950357	VIPR_ALG4_AWW87359_	1b	NS3	B1A	2015	-0.5626	0.5869	1.1259
Pestivirus A	MG950358	VIPR_ALG4_AWW87360_	1b	NS3	B2A	2016	-0.5633	0.5807	1.1259
Pestivirus A	MG950359	VIPR_ALG4_AWW87361_	1b	NS3	B3A	2016	-0.5633	0.5807	1.1259
Pestivirus A	MG950360	VIPR_ALG4_AWW87362_	1b	NS3	B4A	2016	-0.5633	0.5807	1.1259

Species according to VIPRBRC	GenBank Accession	GenBank Protein Accession	Subgenotype	Protein	Strain Name	Collection Year	SVM Patho. Score	Vaxijen Antig. Score	Averged score of EMBOSS motifs
Pestivirus A	MG950361	VIPR_ALG4_AWW87363_	1b	NS3	B5A	2016	-0.5633	0.5807	1.1259
Pestivirus A	MG950362	VIPR_ALG4_AWW87364_	1b	NS3	B6A	2016	-0.5613	0.5785	1.1259
Pestivirus A	MG950363	VIPR_ALG4_AWW87365_	1b	NS3	O1A	2015	-0.5626	0.5869	1.1259
Pestivirus A	MG950364	VIPR_ALG4_AWW87366_	1b	NS3	O2A	2015	-0.5710	0.5877	1.1259
Pestivirus A	MG950365	VIPR_ALG4_AWW87367_	1b	NS3	O2B	2015	-0.5710	0.5877	1.1259
Pestivirus A	MG950366	VIPR_ALG4_AWW87368_	1b	NS3	O4A	2015	-0.5710	0.5877	1.1259
Pestivirus A	MH311874	VIPR_ALG4_AWW87369_	1b	NS3	B2A d168	2016	-0.5633	0.5807	1.1259
Pestivirus A	MH311875	VIPR_ALG4_AWW87370_	1b	NS3	B3A d168	2016	-0.5633	0.5807	1.1259
Pestivirus A	MH311876	VIPR_ALG4_AWW87371_	1b	NS3	B4A d84	2016	-0.5633	0.5807	1.1259
Pestivirus A	MH311877	VIPR_ALG4_AWW87372_	1b	NS3	B4A d168	2016	-0.5633	0.5807	1.1259
Pestivirus A	MH311878	VIPR_ALG4_AWW87373_	1b	NS3	B5A d84	2016	-0.5633	0.5807	1.1259
Pestivirus A	MH311879	VIPR_ALG4_AWW87374_	1b	NS3	B5A d168	2016	-0.5633	0.5807	1.1259
Pestivirus A	MH311880	VIPR_ALG4_AWW87375_	1b	NS3	B6A d84	2016	-0.5613	0.5785	1.1259
Pestivirus A	MH311881	VIPR_ALG4_AWW87376_	1b	NS3	B6A d168	2016	-0.5613	0.5785	1.1259
Pestivirus A	MH379221	VIPR_ALG4_AWW87377_	1b	NS3	P1	2017	-0.5710	0.5877	1.1259
Pestivirus A	MH379222	VIPR_ALG4_AWW87378_	1b	NS3	P2	2017	-0.5710	0.5877	1.1259
Pestivirus A	MH379223	VIPR_ALG4_AWW87379_	1b	NS3	P5	2017	-0.5710	0.5877	1.1259
Pestivirus A	MH379224	VIPR_ALG4_AWW87380_	1b	NS3	P6	2017	-0.5710	0.5877	1.1259
Pestivirus A	MH379225	VIPR_ALG4_AWW87381_	1b	NS3	P7	2017	-0.5710	0.5877	1.1259
Pestivirus A	MH379226	VIPR_ALG4_AWW87382_	1b	NS3	P5A	2017	-0.5710	0.5877	1.1259
Pestivirus A	MH379227	VIPR_ALG4_AWW87383_	1b	NS3	P5B	2017	-0.5710	0.5877	1.1259
Pestivirus A	MH379228	VIPR_ALG4_AWW87384_	1b	NS3	P5C	2017	-0.5710	0.5877	1.1259
Pestivirus A	MH379229	VIPR_ALG4_AWW87385_	1b	NS3	P5D	2017	-0.5710	0.5877	1.1259
Pestivirus A	MH379230	VIPR_ALG4_AWW87386_	1b	NS3	P5F	2017	-0.5710	0.5877	1.1259
Pestivirus A	MH379231	VIPR_ALG4_AWW87387_	1b	NS3	P7A	2018	-0.5710	0.5877	1.1259
Pestivirus A	MH379232	VIPR_ALG4_AWW87388_	1b	NS3	P7C	2018	-0.5710	0.5877	1.1259
Pestivirus A	MH379233	VIPR_ALG4_AWW87389_	1b	NS3	P7E	2018	-0.5710	0.5877	1.1259
Pestivirus A	MH379234	VIPR_ALG4_AWW87390_	1b	NS3	P7F	2018	-0.5710	0.5877	1.1259
Pestivirus A	MH166806	VIPR_ALG4_AYA62524_1	1m	NS3	XC	2015	-0.6412	0.5966	1.1279
Pestivirus A	MH490943	VIPR_ALG4_AZB53078_1	1b	NS3	BVDV BJ-2016	2016	-0.5596	0.5833	1.1302
Pestivirus A	MH231153	VIPR_ALG4_AZQ00677_1	1b	NS3	Nebraska	1990	-0.5345	0.5864	1.1301
Pestivirus A	AB078950	VIPR_ALG4_BAC55961_1	1j	NS3	KS86-1ncp	1986	-0.5493	0.5877	1.1290
Pestivirus A	MH899941	VIPR_ALG4_QCE30388_1	1b	NS3	SLO/3301/2014	2014	-0.5723	0.5851	1.1353
Pestivirus A	MH899942	VIPR_ALG4_QCE30389_1	1e	NS3	SLO/33529/2015	2015	-0.4732	0.5879	1.1266
Pestivirus A	MH899943	VIPR_ALG4_QCE30390_1	1f	NS3	SLO/1361/2014	2014	-0.5546	0.6031	1.1280
Pestivirus A	MH899944	VIPR_ALG4_QCE30391_1	1f	NS3	SLO/28537/2017	2017	-0.6081	0.6032	1.1244
Pestivirus A	MH899945	VIPR_ALG4_QCE30392_1	1h	NS3	SLO/1883/2013	2013	-0.6383	0.6029	1.1340
Pestivirus A	MK102095	VIPR_ALG4_QCQ84262_1	1q	NS3	20170226	2017	-0.5212	0.5873	1.1256
Pestivirus A	MK509774	VIPR_ALG4_QEK23510_1	1b	NS3	BVD1b-JH	2008	-0.5615	0.5844	1.1278
Pestivirus A	MK775204	VIPR_ALG4_QFX66041_1	1i	NS3	CA2006	2006	-0.5125	0.5829	1.1306
Pestivirus A	MN188073	VIPR_ALG4_QGZ19414_1	1a	NS3	PI34	2017	-0.5264	0.5850	1.1290
Pestivirus A	MN188074	VIPR_ALG4_QGZ19415_1	1b	NS3	PI285	2017	-0.5607	0.5842	1.1246
Pestivirus A	MT079816	VIPR_ALG4_QIM55913_1	1c	NS3	GXNN1	2018	-0.4861	0.5947	1.1310
Pestivirus A	MN623291	VIPR_ALG4_QLL27013_1	1m	NS3	NX2019/01	2019	-0.6951	0.5872	1.1346
Pestivirus A	MW014286	VIPR_ALG4_QPJ59878_1	1b	NS3	GXSS01	2018	-0.5572	0.5847	1.1258
Pestivirus A	MW014287	VIPR_ALG4_QPJ59879_1	1b	NS3	GXSS02	2018	-0.6461	0.5875	1.1279
Pestivirus A	MW014288	VIPR_ALG4_QPJ59880_1	1b	NS3	GXSS03	2018	-0.6151	0.5871	1.1252

Species according to VIPRBRC	GenBank Accession	GenBank Protein Accession	Subgenotype	Protein	Strain Name	Collection Year	SVM Patho. Score	Vaxijen Antig. Score	Averged score of EMBOSS motifs
Pestivirus A	MT977117	VIPR_ALG4_QRZ20359_1	1b	NS3	BVDV 1b IT16/5	2016	-0.5580	0.5859	1.1290
Pestivirus A	MT977118	VIPR_ALG4_QRZ20360_1	1b	NS3	BVDV 1b IT16/439	2016	-0.5580	0.5859	1.1290
Pestivirus A	MT654137	VIPR_ALG4_QVK82311_1	1a	NS3	20-8536	2020	-0.5227	0.5872	1.1267
Pestivirus A	LT837585	VIPR_ALG4_SLV80196_1	1r	NS3	UNKNOWN-LT837585	2012	-0.5859	0.5967	1.1260
Pestivirus A	MW054933	VIPR_ALG4_UEC94252_1	1f	NS3	LA/230/14	2014	-0.5753	0.6029	1.1280
Pestivirus A	MW054934	VIPR_ALG4_UEC94253_1	1f	NS3	LA/87/05	2005	-0.5847	0.5989	1.1268
Pestivirus A	MW054935	VIPR_ALG4_UEC94254_1	1k	NS3	TO/197/11	2011	-0.6291	0.5929	1.1290
Pestivirus A	MW054936	VIPR_ALG4_UEC94255_1	1g	NS3	UM/111/06	2006	-0.5143	0.5965	1.1305
Pestivirus A	MW054937	VIPR_ALG4_UEC94256_1	1k	NS3	SA/158/09	2009	-0.6383	0.5947	1.1261
Pestivirus A	MW054938	VIPR_ALG4_UEC94257_1	1k	NS3	SA/159/09	2009	-0.6383	0.5947	1.1261
Pestivirus A	MW054939	VIPR_ALG4_UEC94258_1	1f	NS3	LO/151/09	2009	-0.5861	0.6013	1.1286
Pestivirus A	MW054940	VIPR_ALG4_UEC94259_1	1e	NS3	MA/101/05	2005	-0.4729	0.5919	1.1296
Pestivirus A	MW250796	VIPR_ALG4_UEC94260_1	1i	NS3	58-1	2008	-0.5367	0.5951	1.1253
Pestivirus A	MW250797	VIPR_ALG4_UEC94261_1	1i	NS3	58-2	2008	-0.5367	0.5951	1.1253
Pestivirus A	MW250798	VIPR_ALG4_UEC94262_1	1a	NS3	62-2	2008	-0.5103	0.5846	1.1275
Pestivirus A	MW250799	VIPR_ALG4_UEC94263_1	1a	NS3	63-1	2008	-0.5076	0.5861	1.1297
Pestivirus A	MW250800	VIPR_ALG4_UEC94264_1	1d	NS3	67-1	2008	-0.6454	0.5915	1.1245
Pestivirus A	MW250801	VIPR_ALG4_UEC94265_1	1d	NS3	67-2	2008	-0.6454	0.5915	1.1245
Pestivirus A	MW250802	VIPR_ALG4_UEC94266_1	1e	NS3	68-1	2008	-0.6033	0.5869	1.1303
Pestivirus A	MW250803	VIPR_ALG4_UEC94267_1	1i	NS3	69-1	2008	-0.4959	0.5902	1.1304
Pestivirus A	MW655625	VIPR_ALG4_UEC94268_1	1h	NS3	CH-04-01b	2004	-0.6311	0.5985	1.1304
Pestivirus A	MW655626	VIPR_ALG4_UEC94269_1	1e	NS3	Maria	2004	-0.4811	0.5909	1.1314
Pestivirus A	MW655627	VIPR_ALG4_UEC94270_1	1e	NS3	R2000-95	1995	-0.4519	0.5914	1.1254
Pestivirus A	MW655628	VIPR_ALG4_UEC94271_1	1k	NS3	R3230-95	1995	-0.6416	0.5917	1.1268
Pestivirus A	MW655629	VIPR_ALG4_UEC94272_1	1h	NS3	R3572-90	1990	-0.5929	0.6001	1.1304
Pestivirus A	MW655630	VIPR_ALG4_UEC94273_1	1k	NS3	R5013-96	1996	-0.6427	0.6026	1.1280
Pestivirus A	MW655631	VIPR_ALG4_UEC94274_1	1e	NS3	S03-1175	2003	-0.5815	0.5866	1.1314
Pestivirus A	MW655632	VIPR_ALG4_UEC94275_1	1h	NS3	SM09-20	2002	-0.6137	0.5989	1.1304
Pestivirus A	MW713361	VIPR_ALG4_UEC94276_1	1a	NS3	BoAEC1190	2007	-0.5653	0.5830	1.1264
Pestivirus A	MW713362	VIPR_ALG4_UEC94277_1	1b	NS3	PI819	2017	-0.5533	0.5859	1.1259
Pestivirus A	MW732738	VIPR_ALG4_UEC94278_1	1a	NS3	PI407	2015	-0.5070	0.5808	1.1303
Pestivirus A	MW732739	VIPR_ALG4_UEC94279_1	1a	NS3	YandaSpl	1993	-0.5374	0.5882	1.1300
Pestivirus A	MZ188972	VIPR_ALG4_UML14262_1	1q	NS3	HB-1	2020	-0.5493	0.6054	1.1328
Pestivirus A	ON337882	VIPR_ALG4_USZ80113_1	1c	NS3	NM2103	2021	-0.5122	0.5828	1.1315
Pestivirus A	KU159365	VIPR_ALG4_1039262063	1a	NS4A	USII-S15	2015	0.0954	0.6743	1.1370
Pestivirus A	KU756226	VIPR_ALG4_1072900294	1b	NS4A	HJ-1	2010	-0.2950	0.7084	1.1205
Pestivirus A	KT943518	VIPR_ALG4_1093530908	1d	NS4A	BJ1201	2012	0.4928	0.6991	1.1205
Pestivirus A	LT631725	VIPR_ALG4_1112914034	1h	NS4A	UM/126/07	2007	-0.2950	0.7084	1.1205
Pestivirus A	KX170485	VIPR_ALG4_1129880236	1b	NS4A	V075	2011	-0.2950	0.7084	1.1205
Pestivirus A	KX170486	VIPR_ALG4_1129880238	1b	NS4A	V100	1997	-0.2950	0.7084	1.1205
Pestivirus A	KX170487	VIPR_ALG4_1129880240	1a	NS4A	V048	2009	-0.3142	0.7092	1.1205
Pestivirus A	KX170488	VIPR_ALG4_1129880242	1b	NS4A	V015	2001	-0.2950	0.7084	1.1205
Pestivirus A	KX170489	VIPR_ALG4_1129880244	1a	NS4A	V056	2009	-0.2950	0.7084	1.1205
Pestivirus A	KX170490	VIPR_ALG4_1129880246	1b	NS4A	V060	2004	-0.2950	0.7084	1.1205
Pestivirus A	KX170491	VIPR_ALG4_1129880248	1b	NS4A	V070	2007	-0.2950	0.7084	1.1205
Pestivirus A	KX170492	VIPR_ALG4_1129880250	1b	NS4A	V020	2005	-0.2950	0.7084	1.1205
Pestivirus A	KX170493	VIPR_ALG4_1129880252	1b	NS4A	V029	2006	-0.2950	0.7084	1.1205

Species according to VIPRBRC	GenBank Accession	GenBank Protein Accession	Subgenotype	Protein	Strain Name	Collection Year	SVM Patho. Score	Vaxijen Antig. Score	Averged score of EMBOSS motifs
Pestivirus A	KX170494	VIPR_ALG4_1129880254_	1b	NS4A	V036	2007	-0.2950	0.7084	1.1205
Pestivirus A	KX170495	VIPR_ALG4_1129880256_	1b	NS4A	V031	2006	-0.4562	0.7117	1.1205
Pestivirus A	KX170496	VIPR_ALG4_1129880258_	1b	NS4A	V098	1999	-0.2950	0.7084	1.1205
Pestivirus A	KX170497	VIPR_ALG4_1129880260_	1b	NS4A	V045	2009	-0.2950	0.7084	1.1205
Pestivirus A	KX170498	VIPR_ALG4_1129880262_	1b	NS4A	V078	2012	-0.2950	0.7084	1.1205
Pestivirus A	KX170499	VIPR_ALG4_1129880264_	1b	NS4A	V087	2006	-0.2950	0.7084	1.1205
Pestivirus A	KX170500	VIPR_ALG4_1129880266_	1a	NS4A	V059	2004	0.2169	0.6935	1.1370
Pestivirus A	KX170501	VIPR_ALG4_1129880268_	1a	NS4A	V099	1998	0.2247	0.6801	1.1370
Pestivirus A	KX170502	VIPR_ALG4_1129880270_	1a	NS4A	V083	2008	0.2169	0.6935	1.1370
Pestivirus A	KX170503	VIPR_ALG4_1129880272_	1a	NS4A	V016	2002	0.2830	0.6196	1.1370
Pestivirus A	KX170505	VIPR_ALG4_1129880276_	1a	NS4A	V035	2007	0.2136	0.7359	1.1370
Pestivirus A	KX170506	VIPR_ALG4_1129880278_	1a	NS4A	V039	2008	0.2136	0.7359	1.1370
Pestivirus A	KX170507	VIPR_ALG4_1129880280_	1a	NS4A	V040	2008	0.2136	0.7359	1.1370
Pestivirus A	KX170508	VIPR_ALG4_1129880282_	1a	NS4A	V041	2008	0.2136	0.7359	1.1370
Pestivirus A	KX170509	VIPR_ALG4_1129880284_	1a	NS4A	V042	2008	0.2136	0.7359	1.1370
Pestivirus A	KX170510	VIPR_ALG4_1129880286_	1a	NS4A	V043	2008	0.2136	0.7359	1.1370
Pestivirus A	KX170511	VIPR_ALG4_1129880288_	1a	NS4A	V046	2009	0.2136	0.7359	1.1370
Pestivirus A	KX170512	VIPR_ALG4_1129880290_	1a	NS4A	V050	2009	0.2136	0.7359	1.1370
Pestivirus A	KX170513	VIPR_ALG4_1129880292_	1a	NS4A	V052	2010	0.2136	0.7359	1.1370
Pestivirus A	KX170514	VIPR_ALG4_1129880294_	1a	NS4A	V001	1999	0.2169	0.6935	1.1370
Pestivirus A	KX170515	VIPR_ALG4_1129880296_	1a	NS4A	V008	2000	0.2169	0.6935	1.1370
Pestivirus A	KX170516	VIPR_ALG4_1129880298_	1a	NS4A	V009	2000	0.2169	0.6935	1.1370
Pestivirus A	KX170517	VIPR_ALG4_1129880300_	1a	NS4A	V010	2001	0.2169	0.6935	1.1370
Pestivirus A	KX170518	VIPR_ALG4_1129880302_	1a	NS4A	V026	2006	0.3719	0.6394	1.1370
Pestivirus A	KX170519	VIPR_ALG4_1129880304_	1a	NS4A	V027	2006	0.3719	0.6394	1.1370
Pestivirus A	KX170520	VIPR_ALG4_1129880306_	1a	NS4A	V007	2000	0.2169	0.6935	1.1370
Pestivirus A	KX170521	VIPR_ALG4_1129880308_	1a	NS4A	V013	2001	0.2169	0.6935	1.1370
Pestivirus A	KX170522	VIPR_ALG4_1129880310_	1a	NS4A	V033	2007	0.2169	0.6935	1.1370
Pestivirus A	KX170523	VIPR_ALG4_1129880312_	1a	NS4A	V034	2007	0.2169	0.6935	1.1370
Pestivirus A	KX170524	VIPR_ALG4_1129880314_	1a	NS4A	V049	2009	0.2169	0.6935	1.1370
Pestivirus A	KX170525	VIPR_ALG4_1129880316_	1a	NS4A	V067	2006	0.2169	0.6935	1.1370
Pestivirus A	KX170526	VIPR_ALG4_1129880318_	1a	NS4A	V073	2011	0.2169	0.6935	1.1370
Pestivirus A	KX170527	VIPR_ALG4_1129880320_	1a	NS4A	V074	2010	0.2169	0.6935	1.1370
Pestivirus A	KX170528	VIPR_ALG4_1129880322_	1a	NS4A	V077	2012	0.2169	0.6935	1.1370
Pestivirus A	KX170529	VIPR_ALG4_1129880324_	1a	NS4A	V080	2009	0.2169	0.6935	1.1370
Pestivirus A	KX170530	VIPR_ALG4_1129880326_	1a	NS4A	V091	2003	0.2169	0.6935	1.1370
Pestivirus A	KX170531	VIPR_ALG4_1129880328_	1a	NS4A	V092	2004	-0.2912	0.6668	1.1370
Pestivirus A	KX170532	VIPR_ALG4_1129880330_	1a	NS4A	V011	2001	-0.0041	0.7348	1.1370
Pestivirus A	KX170533	VIPR_ALG4_1129880332_	1a	NS4A	V022	2006	-0.2285	0.6659	1.1370
Pestivirus A	KX170534	VIPR_ALG4_1129880334_	1a	NS4A	V054	2013	-0.2285	0.6659	1.1370
Pestivirus A	KX170535	VIPR_ALG4_1129880336_	1a	NS4A	V012	2001	0.3719	0.6394	1.1370
Pestivirus A	KX170536	VIPR_ALG4_1129880338_	1a	NS4A	V006	2000	-0.3444	0.7188	1.1370
Pestivirus A	KX170537	VIPR_ALG4_1129880340_	1a	NS4A	V057	2009	-0.0145	0.6089	1.1370
Pestivirus A	EF101530	VIPR_ALG4_118498779_1	1b	NS4A	KE9	2007	-0.2950	0.7084	1.1205
Pestivirus A	DQ088995	VIPR_ALG4_145309048_1	1a	NS4A	Singer_Arg	1974	0.2169	0.6935	1.1370
Pestivirus A	U63479	VIPR_ALG4_1518836_72	1b	NS4A	CP7	1987	-0.2950	0.7084	1.1205
Pestivirus A	U86600	VIPR_ALG4_2149469_72	1b	NS4A	ILLNC	1991	-0.1498	0.7004	1.1205

Species according to VIPRBRC	GenBank Accession	GenBank Protein Accession	Subgenotype	Protein	Strain Name	Collection Year	SVM Patho. Score	Vaxijen Antig. Score	Averged score of EMBOSS motifs
Pestivirus A	AF041040	VIPR_ALG4_2789677_71{	1a	NS4A	Oregon	1960	0.2169	0.6935	1.1370
Pestivirus A	M96751	VIPR_ALG4_289508_720	1a	NS4A	UNKNOWN-M96751	1992	0.2169	0.6935	1.1370
Pestivirus A	HQ174292	VIPR_ALG4_323145267_	1a	NS4A	180	2010	0.2169	0.6935	1.1370
Pestivirus A	M31182	VIPR_ALG4_323206_747	1a	NS4A	UNKNOWN-M31182	1988	0.3719	0.6394	1.1370
Pestivirus A	M96687	VIPR_ALG4_323230_743	1b	NS4A	Osloss	1967	-0.2950	0.7084	1.1205
Pestivirus A	JN400273	VIPR_ALG4_363990275_	1q	NS4A	SD0803	2008	0.0429	0.7063	1.1370
Pestivirus A	AF091605	VIPR_ALG4_3661566_72	1a	NS4A	Oregon C24V	1960	0.2169	0.6935	1.1370
Pestivirus A	JN644055	VIPR_ALG4_373939303_	1b	NS4A	3156	2011	-0.9883	0.7105	1.1205
Pestivirus A	JN380080	VIPR_ALG4_378753653_	1a	NS4A	6010	2010	0.2169	0.6935	1.1370
Pestivirus A	JQ799141	VIPR_ALG4_390132765_	1u	NS4A	M31182	2010	1.0168	0.6021	1.1345
Pestivirus A	JX419397	VIPR_ALG4_404363562_	1b	NS4A	UNKNOWN-JX419397	2008	-0.2950	0.7084	1.1205
Pestivirus A	JX419398	VIPR_ALG4_404363564_	1b	NS4A	UNKNOWN-JX419398	2008	-0.2950	0.7084	1.1205
Pestivirus A	AF526381	VIPR_ALG4_42476348_7	1m	NS4A	ZM-95	1995	0.0017	0.7169	1.1205
Pestivirus A	JX297512	VIPR_ALG4_459284067_	1b	NS4A	10270	2007	-0.2950	0.7084	1.1205
Pestivirus A	JX297513	VIPR_ALG4_459284069_	1b	NS4A	Aries	2005	-0.2950	0.7084	1.1205
Pestivirus A	JX297514	VIPR_ALG4_459284071_	1b	NS4A	Columba	2005	-0.2950	0.7084	1.1205
Pestivirus A	JX297515	VIPR_ALG4_459284073_	1b	NS4A	Corona	2005	-0.2950	0.7084	1.1205
Pestivirus A	JX297516	VIPR_ALG4_459284075_	1b	NS4A	Gemini	2005	-0.2950	0.7084	1.1205
Pestivirus A	JX297517	VIPR_ALG4_459284077_	1b	NS4A	Hercules	2006	-0.2950	0.7084	1.1205
Pestivirus A	JX297518	VIPR_ALG4_459284079_	1b	NS4A	Leo	2006	-0.2950	0.7084	1.1205
Pestivirus A	JX297519	VIPR_ALG4_459284081_	1b	NS4A	Lyra	2006	-0.2950	0.7084	1.1205
Pestivirus A	JX297520	VIPR_ALG4_459284083_	1b	NS4A	Mars	2006	-0.2950	0.7084	1.1205
Pestivirus A	JX297521	VIPR_ALG4_459284085_	1b	NS4A	Scorpius	2006	-0.2950	0.7084	1.1205
Pestivirus A	KC853440	VIPR_ALG4_507144146_	1k	NS4A	SuwaNcp	1993	-0.3498	0.7094	1.1205
Pestivirus A	KC853441	VIPR_ALG4_507144148_	1k	NS4A	SuwaCp	1993	-0.3498	0.7094	1.1205
Pestivirus A	KC695810	VIPR_ALG4_507866685_	1q	NS4A	camel-6	2010	-0.5326	0.7337	1.1205
Pestivirus A	KC695814	VIPR_ALG4_507866704_	1b	NS4A	Av69 VEDEVAC	2011	-0.4612	0.7122	1.1205
Pestivirus A	KC757383	VIPR_ALG4_511775165_	1d	NS4A	10JJ-SKR	2010	0.1809	0.6906	1.1205
Pestivirus A	KC963967	VIPR_ALG4_530291194_	1b	NS4A	12F004	2012	-0.2950	0.7084	1.1205
Pestivirus A	KF772785	VIPR_ALG4_575471151_	1b	NS4A	CC13B	2013	-0.2950	0.7084	1.1205
Pestivirus A	KF896608	VIPR_ALG4_586616532_	1c	NS4A	Bega-like	2012	-0.2950	0.7084	1.1205
Pestivirus A	KF835697	VIPR_ALG4_597437474_	1b	NS4A	AU526	2013	-0.2950	0.7084	1.1205
Pestivirus A	KJ541471	VIPR_ALG4_633265982_	1a	NS4A	GS5	2013	0.5507	0.7019	1.1370
Pestivirus A	KJ689448	VIPR_ALG4_635172915_	1b	NS4A	GX4	2012	-0.4239	0.6667	1.1205
Pestivirus A	KF501393	VIPR_ALG4_669206614_	1b	NS4A	BVDV JL-1	2009	-0.2950	0.7084	1.1205
Pestivirus A	AJ133738	VIPR_ALG4_7960754_74	1a	NS4A	type 1	1963	0.3719	0.6394	1.1370
Pestivirus A	KP941581	VIPR_ALG4_800924313_	1b	NS4A	USMARC-51998	2014	-0.3556	0.7239	1.1205
Pestivirus A	KP941583	VIPR_ALG4_800924317_	1b	NS4A	USMARC-53874	2014	-0.2950	0.7084	1.1205
Pestivirus A	KP941584	VIPR_ALG4_800924319_	1a	NS4A	USMARC-53875	2014	-0.0289	0.7423	1.1320
Pestivirus A	KP941586	VIPR_ALG4_800924323_	1a	NS4A	USMARC-55477	2014	0.2169	0.6935	1.1370
Pestivirus A	KP941587	VIPR_ALG4_800924325_	1b	NS4A	USMARC-55478	2014	-0.2950	0.7084	1.1205
Pestivirus A	KP941588	VIPR_ALG4_800924327_	1b	NS4A	USMARC-55922	2014	-0.2950	0.7084	1.1205
Pestivirus A	KP941589	VIPR_ALG4_800924329_	1b	NS4A	USMARC-55923	2014	-0.2950	0.7084	1.1205
Pestivirus A	KP941590	VIPR_ALG4_800924331_	1b	NS4A	USMARC-55924	2014	-0.2950	0.7084	1.1205
Pestivirus A	KP941591	VIPR_ALG4_800924333_	1b	NS4A	USMARC-55925	2014	-0.2950	0.7084	1.1205
Pestivirus A	KP941592	VIPR_ALG4_800924335_	1b	NS4A	USMARC-55926	2014	-0.2950	0.7084	1.1205
Pestivirus A	KP313732	VIPR_ALG4_816850387_	1e	NS4A	Carlito	2014	-0.4612	0.7122	1.1205

Species according to VIPRBRC	GenBank Accession	GenBank Protein Accession	Subgenotype	Protein	Strain Name	Collection Year	SVM Patho. Score	Vaxijen Antig. Score	Averged score of EMBOSS motifs
Pestivirus A	KR029825	VIPR_ALG4_887497286_1	1b	NS4A	Egy/Ismailia/2014	2014	-0.2950	0.7084	1.1205
Pestivirus A	LC089875	VIPR_ALG4_939106262_1	1o	NS4A	IS26/01ncp	2001	0.0017	0.7169	1.1205
Pestivirus A	LC089876	VIPR_ALG4_939106264_1	1n	NS4A	Shitara/02/06	2006	-0.4754	0.7151	1.1205
Pestivirus A	KR866116	VIPR_ALG4_941508008_1	1m	NS4A	SD-15	2015	0.0017	0.7169	1.1205
Pestivirus A	KU200260	VIPR_ALG4_972905813_1	1b	NS4A	BE/061536/2014	2014	-0.0915	0.6595	1.1205
Pestivirus A	KX577637	VIPR_ALG4_AOR50934_1	1e	NS4A	SLO/2407/2006	2006	0.0113	0.7756	1.1133
Pestivirus A	KX987157	VIPR_ALG4_APG30987_1	1f	NS4A	SLO/1170/2000	2000	-0.3153	0.7350	1.1205
Pestivirus A	KX857724	VIPR_ALG4_APZ85839_1	1i	NS4A	ACM/BR/2016	2016	-0.1768	0.6633	1.1205
Pestivirus A	KY849592	VIPR_ALG4_ART90617_1	1d	NS4A	SLO/2416/2002	2002	0.1809	0.6906	1.1205
Pestivirus A	MF278651	VIPR_ALG4_ASW18434_1	1b	NS4A	XZ01	2016	-0.4612	0.7122	1.1205
Pestivirus A	MF278652	VIPR_ALG4_ASW18435_1	1b	NS4A	XZ02	2016	-0.4612	0.7122	1.1205
Pestivirus A	MF693403	VIPR_ALG4_ATG71375_1	1a	NS4A	UNKNOWN-MF693403	2016	0.1439	0.6783	1.1370
Pestivirus A	KY964311	VIPR_ALG4_ATN39078_1	1b	NS4A	Y2	2014	-0.2950	0.7084	1.1205
Pestivirus A	MF172980	VIPR_ALG4_AVI10261_1	1c	NS4A	GSTZ	2012	0.0950	0.7050	1.1205
Pestivirus A	MH379638	VIPR_ALG4_AWW14171_	1a	NS4A	Ho916	1993	0.1662	0.6944	1.1370
Pestivirus A	MG950344	VIPR_ALG4_AWW87346_	1b	NS4A	AU526	2014	-0.2950	0.7084	1.1205
Pestivirus A	MG950345	VIPR_ALG4_AWW87347_	1b	NS4A	B1	2015	-0.2950	0.7084	1.1205
Pestivirus A	MG950346	VIPR_ALG4_AWW87348_	1b	NS4A	B2	2015	-0.2950	0.7084	1.1205
Pestivirus A	MG950347	VIPR_ALG4_AWW87349_	1b	NS4A	B3	2015	-0.2950	0.7084	1.1205
Pestivirus A	MG950348	VIPR_ALG4_AWW87350_	1b	NS4A	B4	2015	-0.2950	0.7084	1.1205
Pestivirus A	MG950349	VIPR_ALG4_AWW87351_	1b	NS4A	B5	2015	-0.2950	0.7084	1.1205
Pestivirus A	MG950350	VIPR_ALG4_AWW87352_	1b	NS4A	B6	2015	-0.2950	0.7084	1.1205
Pestivirus A	MG950351	VIPR_ALG4_AWW87353_	1b	NS4A	O1	2015	-0.2950	0.7084	1.1205
Pestivirus A	MG950352	VIPR_ALG4_AWW87354_	1b	NS4A	O2	2015	-0.2950	0.7084	1.1205
Pestivirus A	MG950353	VIPR_ALG4_AWW87355_	1b	NS4A	O3	2015	-0.2950	0.7084	1.1205
Pestivirus A	MG950354	VIPR_ALG4_AWW87356_	1b	NS4A	O4	2015	-0.2950	0.7084	1.1205
Pestivirus A	MG950355	VIPR_ALG4_AWW87357_	1b	NS4A	O5	2015	-0.2950	0.7084	1.1205
Pestivirus A	MG950356	VIPR_ALG4_AWW87358_	1b	NS4A	O6	2015	-0.2950	0.7084	1.1205
Pestivirus A	MG950357	VIPR_ALG4_AWW87359_	1b	NS4A	B1A	2015	-0.2950	0.7084	1.1205
Pestivirus A	MG950358	VIPR_ALG4_AWW87360_	1b	NS4A	B2A	2016	-0.2950	0.7084	1.1205
Pestivirus A	MG950359	VIPR_ALG4_AWW87361_	1b	NS4A	B3A	2016	-0.2950	0.7084	1.1205
Pestivirus A	MG950360	VIPR_ALG4_AWW87362_	1b	NS4A	B4A	2016	-0.2950	0.7084	1.1205
Pestivirus A	MG950361	VIPR_ALG4_AWW87363_	1b	NS4A	B5A	2016	-0.2950	0.7084	1.1205
Pestivirus A	MG950362	VIPR_ALG4_AWW87364_	1b	NS4A	B6A	2016	-0.2950	0.7084	1.1205
Pestivirus A	MG950363	VIPR_ALG4_AWW87365_	1b	NS4A	O1A	2015	-0.2950	0.7084	1.1205
Pestivirus A	MG950364	VIPR_ALG4_AWW87366_	1b	NS4A	O2A	2015	-0.2950	0.7084	1.1205
Pestivirus A	MG950365	VIPR_ALG4_AWW87367_	1b	NS4A	O2B	2015	-0.2950	0.7084	1.1205
Pestivirus A	MG950366	VIPR_ALG4_AWW87368_	1b	NS4A	O4A	2015	-0.2950	0.7084	1.1205
Pestivirus A	MH311874	VIPR_ALG4_AWW87369_	1b	NS4A	B2A d168	2016	-0.2950	0.7084	1.1205
Pestivirus A	MH311875	VIPR_ALG4_AWW87370_	1b	NS4A	B3A d168	2016	-0.2950	0.7084	1.1205
Pestivirus A	MH311876	VIPR_ALG4_AWW87371_	1b	NS4A	B4A d84	2016	-0.2950	0.7084	1.1205
Pestivirus A	MH311877	VIPR_ALG4_AWW87372_	1b	NS4A	B4A d168	2016	-0.2950	0.7084	1.1205
Pestivirus A	MH311878	VIPR_ALG4_AWW87373_	1b	NS4A	B5A d84	2016	-0.2950	0.7084	1.1205
Pestivirus A	MH311879	VIPR_ALG4_AWW87374_	1b	NS4A	B5A d168	2016	-0.2950	0.7084	1.1205
Pestivirus A	MH311880	VIPR_ALG4_AWW87375_	1b	NS4A	B6A d84	2016	-0.2950	0.7084	1.1205
Pestivirus A	MH311881	VIPR_ALG4_AWW87376_	1b	NS4A	B6A d168	2016	-0.2950	0.7084	1.1205
Pestivirus A	MH379221	VIPR_ALG4_AWW87377_	1b	NS4A	P1	2017	-0.2950	0.7084	1.1205

Species according to VIPRBRC	GenBank Accession	GenBank Protein Accession	Subgenotype	Protein	Strain Name	Collection Year	SVM Patho. Score	Vaxijen Antig. Score	Averged score of EMBOSS motifs
Pestivirus A	MH379222	VIPR_ALG4_AWW87378_	1b	NS4A	P2	2017	-0.2950	0.7084	1.1205
Pestivirus A	MH379223	VIPR_ALG4_AWW87379_	1b	NS4A	P5	2017	-0.2950	0.7084	1.1205
Pestivirus A	MH379224	VIPR_ALG4_AWW87380_	1b	NS4A	P6	2017	-0.2950	0.7084	1.1205
Pestivirus A	MH379225	VIPR_ALG4_AWW87381_	1b	NS4A	P7	2017	-0.2950	0.7084	1.1205
Pestivirus A	MH379226	VIPR_ALG4_AWW87382_	1b	NS4A	P5A	2017	-0.2950	0.7084	1.1205
Pestivirus A	MH379227	VIPR_ALG4_AWW87383_	1b	NS4A	P5B	2017	-0.2950	0.7084	1.1205
Pestivirus A	MH379228	VIPR_ALG4_AWW87384_	1b	NS4A	P5C	2017	-0.2950	0.7084	1.1205
Pestivirus A	MH379229	VIPR_ALG4_AWW87385_	1b	NS4A	P5D	2017	-0.2950	0.7084	1.1205
Pestivirus A	MH379230	VIPR_ALG4_AWW87386_	1b	NS4A	P5F	2017	-0.2950	0.7084	1.1205
Pestivirus A	MH379231	VIPR_ALG4_AWW87387_	1b	NS4A	P7A	2018	-0.2950	0.7084	1.1205
Pestivirus A	MH379232	VIPR_ALG4_AWW87388_	1b	NS4A	P7C	2018	-0.2950	0.7084	1.1205
Pestivirus A	MH379233	VIPR_ALG4_AWW87389_	1b	NS4A	P7E	2018	-0.2950	0.7084	1.1205
Pestivirus A	MH379234	VIPR_ALG4_AWW87390_	1b	NS4A	P7F	2018	-0.2950	0.7084	1.1205
Pestivirus A	MH166806	VIPR_ALG4_AYA62524_1	1m	NS4A	XC	2015	0.0017	0.7169	1.1205
Pestivirus A	MH490943	VIPR_ALG4_AZB53078_1	1b	NS4A	BVDV BJ-2016	2016	-0.2950	0.7084	1.1205
Pestivirus A	MH231153	VIPR_ALG4_AZQ00677_1	1b	NS4A	Nebraska	1990	-0.2950	0.7084	1.1205
Pestivirus A	AB078950	VIPR_ALG4_BAC55961_1	1j	NS4A	KS86-1ncp	1986	-0.0046	0.6944	1.1205
Pestivirus A	MH899941	VIPR_ALG4_QCE30388_1	1b	NS4A	SLO/3301/2014	2014	-0.2950	0.7084	1.1205
Pestivirus A	MH899942	VIPR_ALG4_QCE30389_1	1e	NS4A	SLO/33529/2015	2015	-0.1439	0.7307	1.1023
Pestivirus A	MH899943	VIPR_ALG4_QCE30390_1	1f	NS4A	SLO/1361/2014	2014	-0.5648	0.6693	1.1205
Pestivirus A	MH899944	VIPR_ALG4_QCE30391_1	1f	NS4A	SLO/28537/2017	2017	-0.2950	0.7084	1.1205
Pestivirus A	MH899945	VIPR_ALG4_QCE30392_1	1h	NS4A	SLO/1883/2013	2013	-0.2950	0.7084	1.1205
Pestivirus A	MK102095	VIPR_ALG4_QCQ84262_1	1q	NS4A	20170226	2017	-0.3984	0.6836	1.1370
Pestivirus A	MK509774	VIPR_ALG4_QEK23510_1	1b	NS4A	BVD1b-JH	2008	-0.3498	0.7094	1.1205
Pestivirus A	MK775204	VIPR_ALG4_QFX66041_1	1i	NS4A	CA2006	2006	0.4928	0.6991	1.1205
Pestivirus A	MN188073	VIPR_ALG4_QGZ19414_1	1a	NS4A	PI34	2017	-0.0080	0.6839	1.1370
Pestivirus A	MN188074	VIPR_ALG4_QGZ19415_1	1b	NS4A	PI285	2017	-0.4612	0.7122	1.1205
Pestivirus A	MT079816	VIPR_ALG4_QIM55913_1	1c	NS4A	GXNN1	2018	0.2016	0.6962	1.1205
Pestivirus A	MN623291	VIPR_ALG4_QLL27013_1	1m	NS4A	NX2019/01	2019	-0.1559	0.7487	1.1057
Pestivirus A	MW014286	VIPR_ALG4_QPJ59878_1	1b	NS4A	GXSS01	2018	-0.4239	0.6667	1.1205
Pestivirus A	MW014287	VIPR_ALG4_QPJ59879_1	1b	NS4A	GXSS02	2018	-0.4612	0.7122	1.1205
Pestivirus A	MW014288	VIPR_ALG4_QPJ59880_1	1b	NS4A	GXSS03	2018	-0.4612	0.7122	1.1205
Pestivirus A	MT977117	VIPR_ALG4_QRZ20359_1	1b	NS4A	BVDV 1b IT16/5	2016	-0.2950	0.7084	1.1205
Pestivirus A	MT977118	VIPR_ALG4_QRZ20360_1	1b	NS4A	BVDV 1b IT16/439	2016	-0.2950	0.7084	1.1205
Pestivirus A	MT654137	VIPR_ALG4_QVK82311_1	1a	NS4A	20-8536	2020	-0.2285	0.6659	1.1370
Pestivirus A	LT837585	VIPR_ALG4_SLV80196_1	1r	NS4A	UNKNOWN-LT837585	2012	-0.2950	0.7084	1.1205
Pestivirus A	MW054933	VIPR_ALG4_UEC94252_1	1f	NS4A	LA/230/14	2014	-0.4562	0.7117	1.1205
Pestivirus A	MW054934	VIPR_ALG4_UEC94253_1	1f	NS4A	LA/87/05	2005	-0.1646	0.7201	1.1205
Pestivirus A	MW054935	VIPR_ALG4_UEC94254_1	1k	NS4A	TO/197/11	2011	-0.3498	0.7094	1.1205
Pestivirus A	MW054936	VIPR_ALG4_UEC94255_1	1g	NS4A	UM/111/06	2006	-0.2504	0.7403	1.1370
Pestivirus A	MW054937	VIPR_ALG4_UEC94256_1	1k	NS4A	SA/158/09	2009	-0.3498	0.7094	1.1205
Pestivirus A	MW054938	VIPR_ALG4_UEC94257_1	1k	NS4A	SA/159/09	2009	-0.3498	0.7094	1.1205
Pestivirus A	MW054939	VIPR_ALG4_UEC94258_1	1f	NS4A	LO/151/09	2009	-0.4562	0.7117	1.1205
Pestivirus A	MW054940	VIPR_ALG4_UEC94259_1	1e	NS4A	MA/101/05	2005	0.0850	0.7267	1.1133
Pestivirus A	MW250796	VIPR_ALG4_UEC94260_1	1i	NS4A	58-1	2008	0.0743	0.6055	1.1205
Pestivirus A	MW250797	VIPR_ALG4_UEC94261_1	1i	NS4A	58-2	2008	0.0743	0.6055	1.1205
Pestivirus A	MW250798	VIPR_ALG4_UEC94262_1	1a	NS4A	62-2	2008	0.2169	0.6935	1.1370

Species according to VIPRBRC	GenBank Accession	GenBank Protein Accession	Subgenotype	Protein	Strain Name	Collection Year	SVM Patho. Score	Vaxijen Antig. Score	Averged score of EMBOSS motifs
Pestivirus A	MW250799	VIPR_ALG4_UEC94263_1	1a	NS4A	63-1	2008	0.2169	0.6935	1.1370
Pestivirus A	MW250800	VIPR_ALG4_UEC94264_1	1d	NS4A	67-1	2008	-0.2950	0.7084	1.1205
Pestivirus A	MW250801	VIPR_ALG4_UEC94265_1	1d	NS4A	67-2	2008	-0.2950	0.7084	1.1205
Pestivirus A	MW250802	VIPR_ALG4_UEC94266_1	1e	NS4A	68-1	2008	-0.2950	0.7667	1.1023
Pestivirus A	MW250803	VIPR_ALG4_UEC94267_1	1i	NS4A	69-1	2008	-0.0286	0.6064	1.1205
Pestivirus A	MW655625	VIPR_ALG4_UEC94268_1	1h	NS4A	CH-04-01b	2004	-0.2950	0.7084	1.1205
Pestivirus A	MW655626	VIPR_ALG4_UEC94269_1	1e	NS4A	Maria	2004	-0.2950	0.7084	1.1205
Pestivirus A	MW655627	VIPR_ALG4_UEC94270_1	1e	NS4A	R2000-95	1995	-0.2950	0.7667	1.1023
Pestivirus A	MW655628	VIPR_ALG4_UEC94271_1	1k	NS4A	R3230-95	1995	-0.5654	0.7126	1.1205
Pestivirus A	MW655629	VIPR_ALG4_UEC94272_1	1h	NS4A	R3572-90	1990	-0.2950	0.7084	1.1205
Pestivirus A	MW655630	VIPR_ALG4_UEC94273_1	1k	NS4A	R5013-96	1996	-0.3498	0.7094	1.1205
Pestivirus A	MW655631	VIPR_ALG4_UEC94274_1	1e	NS4A	S03-1175	2003	-0.2950	0.7084	1.1205
Pestivirus A	MW655632	VIPR_ALG4_UEC94275_1	1h	NS4A	SM09-20	2002	-0.2950	0.7084	1.1205
Pestivirus A	MW713361	VIPR_ALG4_UEC94276_1	1a	NS4A	BoAEC1190	2007	0.4253	0.7452	1.1370
Pestivirus A	MW713362	VIPR_ALG4_UEC94277_1	1b	NS4A	PI819	2017	-0.2950	0.7084	1.1205
Pestivirus A	MW732738	VIPR_ALG4_UEC94278_1	1a	NS4A	PI407	2015	0.2169	0.6935	1.1370
Pestivirus A	MW732739	VIPR_ALG4_UEC94279_1	1a	NS4A	YandaSpl	1993	0.5507	0.7019	1.1370
Pestivirus A	MZ188972	VIPR_ALG4_UML14262_1	1q	NS4A	HB-1	2020	0.0017	0.7169	1.1205
Pestivirus A	ON337882	VIPR_ALG4_USZ80113_1	1c	NS4A	NM2103	2021	0.1292	0.7040	1.1205
Pestivirus A	KU159365	VIPR_ALG4_1039262063	1a	NS4B	USII-S15	2015	-0.3201	0.2962	1.1285
Pestivirus A	KU756226	VIPR_ALG4_1072900294	1b	NS4B	HJ-1	2010	0.0104	0.2988	1.1368
Pestivirus A	KT943518	VIPR_ALG4_1093530908	1d	NS4B	BJ1201	2012	-0.0546	0.2712	1.1405
Pestivirus A	LT631725	VIPR_ALG4_1112914034	1h	NS4B	UM/126/07	2007	-0.2254	0.2883	1.1350
Pestivirus A	KX170545	VIPR_ALG4_1129880356	1b	NS4B	V015	2001	-0.0340	0.2976	1.1343
Pestivirus A	KX170546	VIPR_ALG4_1129880358	1b	NS4B	V100	1997	0.0098	0.3049	1.1326
Pestivirus A	KX170547	VIPR_ALG4_1129880360	1b	NS4B	V075	2011	-0.2207	0.3025	1.1344
Pestivirus A	KX170548	VIPR_ALG4_1129880362	1b	NS4B	V060	2004	-0.0648	0.3024	1.1344
Pestivirus A	KX170549	VIPR_ALG4_1129880364	1b	NS4B	V070	2007	-0.1500	0.3037	1.1261
Pestivirus A	KX170550	VIPR_ALG4_1129880366	1a	NS4B	V048	2009	-0.1253	0.3002	1.1368
Pestivirus A	KX170551	VIPR_ALG4_1129880368	1b	NS4B	V098	1999	-0.1188	0.3027	1.1344
Pestivirus A	KX170552	VIPR_ALG4_1129880370	1b	NS4B	V020	2005	-0.0124	0.2916	1.1368
Pestivirus A	KX170553	VIPR_ALG4_1129880372	1b	NS4B	V029	2006	-0.0124	0.2916	1.1368
Pestivirus A	KX170554	VIPR_ALG4_1129880374	1b	NS4B	V036	2007	0.1011	0.3040	1.1295
Pestivirus A	KX170555	VIPR_ALG4_1129880376	1b	NS4B	V078	2012	-0.0749	0.2986	1.1315
Pestivirus A	KX170556	VIPR_ALG4_1129880378	1b	NS4B	V031	2006	-0.0791	0.2816	1.1344
Pestivirus A	KX170557	VIPR_ALG4_1129880380	1b	NS4B	V045	2009	-0.2209	0.2967	1.1344
Pestivirus A	KX170558	VIPR_ALG4_1129880382	1b	NS4B	V087	2006	-0.2151	0.2940	1.1291
Pestivirus A	KX170559	VIPR_ALG4_1129880384	1a	NS4B	V026	2006	-0.1663	0.3027	1.1287
Pestivirus A	KX170560	VIPR_ALG4_1129880386	1a	NS4B	V027	2006	-0.1663	0.3027	1.1287
Pestivirus A	KX170561	VIPR_ALG4_1129880388	1a	NS4B	V080	2009	-0.3020	0.2948	1.1267
Pestivirus A	KX170562	VIPR_ALG4_1129880390	1a	NS4B	V073	2011	-0.3400	0.2971	1.1267
Pestivirus A	KX170563	VIPR_ALG4_1129880392	1a	NS4B	V074	2010	-0.2885	0.2924	1.1285
Pestivirus A	KX170564	VIPR_ALG4_1129880394	1a	NS4B	V091	2003	-0.2710	0.2998	1.1267
Pestivirus A	KX170565	VIPR_ALG4_1129880396	1a	NS4B	V034	2007	-0.2890	0.3141	1.1307
Pestivirus A	KX170566	VIPR_ALG4_1129880398	1a	NS4B	V067	2006	-0.3020	0.2948	1.1267
Pestivirus A	KX170567	VIPR_ALG4_1129880400	1a	NS4B	V007	2000	-0.3020	0.2948	1.1267
Pestivirus A	KX170568	VIPR_ALG4_1129880402	1a	NS4B	V013	2001	-0.3020	0.2948	1.1267

Species according to VIPRBRC	GenBank Accession	GenBank Protein Accession	Subgenotype	Protein	Strain Name	Collection Year	SVM Patho. Score	Vaxijen Antig. Score	Averged score of EMBOSS motifs
Pestivirus A	KX170569	VIPR_ALG4_1129880404_	1a	NS4B	V033	2007	-0.3020	0.2948	1.1267
Pestivirus A	KX170570	VIPR_ALG4_1129880406_	1a	NS4B	V049	2009	-0.3020	0.2948	1.1267
Pestivirus A	KX170571	VIPR_ALG4_1129880408_	1a	NS4B	V077	2012	-0.3020	0.2948	1.1267
Pestivirus A	KX170572	VIPR_ALG4_1129880410_	1a	NS4B	V022	2006	-0.2442	0.3002	1.1285
Pestivirus A	KX170573	VIPR_ALG4_1129880412_	1a	NS4B	V092	2004	-0.2525	0.3058	1.1297
Pestivirus A	KX170574	VIPR_ALG4_1129880414_	1a	NS4B	V054	2013	-0.3262	0.2852	1.1303
Pestivirus A	KX170575	VIPR_ALG4_1129880416_	1a	NS4B	V011	2001	-0.2416	0.2990	1.1275
Pestivirus A	KX170576	VIPR_ALG4_1129880418_	1a	NS4B	V012	2001	-0.2416	0.2990	1.1275
Pestivirus A	KX170577	VIPR_ALG4_1129880420_	1a	NS4B	V057	2009	-0.2738	0.2958	1.1285
Pestivirus A	KX170578	VIPR_ALG4_1129880422_	1a	NS4B	V006	2000	-0.2495	0.2918	1.1285
Pestivirus A	KX170579	VIPR_ALG4_1129880424_	1a	NS4B	V059	2004	-0.2186	0.2943	1.1261
Pestivirus A	KX170580	VIPR_ALG4_1129880426_	1a	NS4B	V099	1998	-0.3138	0.3015	1.1280
Pestivirus A	KX170581	VIPR_ALG4_1129880428_	1a	NS4B	V016	2002	-0.0997	0.3491	1.1274
Pestivirus A	KX170582	VIPR_ALG4_1129880430_	1a	NS4B	V001	1999	-0.1507	0.3050	1.1295
Pestivirus A	KX170583	VIPR_ALG4_1129880432_	1a	NS4B	V008	2000	-0.1507	0.3050	1.1295
Pestivirus A	KX170584	VIPR_ALG4_1129880434_	1a	NS4B	V009	2000	-0.1507	0.3050	1.1295
Pestivirus A	KX170585	VIPR_ALG4_1129880436_	1a	NS4B	V010	2001	-0.0956	0.2954	1.1295
Pestivirus A	KX170586	VIPR_ALG4_1129880438_	1a	NS4B	V035	2007	-0.1394	0.3015	1.1295
Pestivirus A	KX170587	VIPR_ALG4_1129880440_	1a	NS4B	V050	2009	-0.2305	0.3006	1.1359
Pestivirus A	KX170588	VIPR_ALG4_1129880442_	1a	NS4B	V039	2008	-0.3082	0.3136	1.1295
Pestivirus A	KX170589	VIPR_ALG4_1129880444_	1a	NS4B	V041	2008	-0.3082	0.3136	1.1295
Pestivirus A	KX170590	VIPR_ALG4_1129880446_	1a	NS4B	V042	2008	-0.2697	0.3012	1.1295
Pestivirus A	KX170591	VIPR_ALG4_1129880448_	1a	NS4B	V043	2008	-0.2697	0.3012	1.1295
Pestivirus A	KX170592	VIPR_ALG4_1129880450_	1a	NS4B	V046	2009	-0.2878	0.2960	1.1295
Pestivirus A	KX170593	VIPR_ALG4_1129880452_	1a	NS4B	V052	2010	-0.2615	0.3004	1.1270
Pestivirus A	KX170594	VIPR_ALG4_1129880454_	1a	NS4B	V040	2008	-0.2697	0.3012	1.1295
Pestivirus A	EF101530	VIPR_ALG4_118498779_7	1b	NS4B	KE9	2007	-0.0193	0.3124	1.1337
Pestivirus A	DQ088995	VIPR_ALG4_145309048_7	1a	NS4B	Singer_Arg	1974	-0.3020	0.2948	1.1267
Pestivirus A	U63479	VIPR_ALG4_1518836_740	1b	NS4B	CP7	1987	0.1328	0.2967	1.1335
Pestivirus A	U86600	VIPR_ALG4_2149469_739	1b	NS4B	ILLNC	1991	-0.0043	0.3129	1.1330
Pestivirus A	AF041040	VIPR_ALG4_2789677_737	1a	NS4B	Oregon	1960	-0.2381	0.2953	1.1268
Pestivirus A	M96751	VIPR_ALG4_289508_7394	1a	NS4B	UNKNOWN-M96751	1992	-0.1507	0.3050	1.1295
Pestivirus A	HQ174292	VIPR_ALG4_323145267_7	1a	NS4B	180	2010	-0.1767	0.3024	1.1292
Pestivirus A	M31182	VIPR_ALG4_323206_7664	1a	NS4B	UNKNOWN-M31182	1988	-0.1968	0.2937	1.1285
Pestivirus A	M96687	VIPR_ALG4_323230_7623	1b	NS4B	Osloss	1967	-0.0182	0.2911	1.1195
Pestivirus A	JN400273	VIPR_ALG4_363990275_7	1q	NS4B	SD0803	2008	-0.1391	0.2960	1.1273
Pestivirus A	AF091605	VIPR_ALG4_3661566_739	1a	NS4B	Oregon C24V	1960	-0.2186	0.2943	1.1261
Pestivirus A	JN644055	VIPR_ALG4_373939303_7	1b	NS4B	3156	2011	-0.0981	0.3193	1.1303
Pestivirus A	JN380080	VIPR_ALG4_378753653_7	1a	NS4B	6010	2010	-0.2413	0.3003	1.1292
Pestivirus A	JX419397	VIPR_ALG4_404363562_7	1b	NS4B	UNKNOWN-JX419397	2008	0.1428	0.2846	1.1315
Pestivirus A	JX419398	VIPR_ALG4_404363564_7	1b	NS4B	UNKNOWN-JX419398	2008	0.1428	0.2846	1.1315
Pestivirus A	AF526381	VIPR_ALG4_42476348_73	1m	NS4B	ZM-95	1995	-0.4170	0.3024	1.1336
Pestivirus A	JX297512	VIPR_ALG4_459284067_7	1b	NS4B	10270	2007	-0.1671	0.3029	1.1350
Pestivirus A	JX297513	VIPR_ALG4_459284069_7	1b	NS4B	Aries	2005	-0.1671	0.3029	1.1350
Pestivirus A	JX297514	VIPR_ALG4_459284071_7	1b	NS4B	Columba	2005	-0.1671	0.3029	1.1350
Pestivirus A	JX297515	VIPR_ALG4_459284073_7	1b	NS4B	Corona	2005	-0.1671	0.3029	1.1350
Pestivirus A	JX297516	VIPR_ALG4_459284075_7	1b	NS4B	Gemini	2005	-0.1738	0.3078	1.1375

Species according to VIPRBRC	GenBank Accession	GenBank Protein Accession	Subgenotype	Protein	Strain Name	Collection Year	SVM Patho. Score	Vaxijen Antig. Score	Averged score of EMBOSS motifs
Pestivirus A	JX297517	VIPR_ALG4_459284077_1	1b	NS4B	Hercules	2006	-0.1418	0.3028	1.1350
Pestivirus A	JX297518	VIPR_ALG4_459284079_1	1b	NS4B	Leo	2006	-0.1671	0.3029	1.1350
Pestivirus A	JX297519	VIPR_ALG4_459284081_1	1b	NS4B	Lyra	2006	-0.2400	0.3052	1.1350
Pestivirus A	JX297520	VIPR_ALG4_459284083_1	1b	NS4B	Mars	2006	-0.1671	0.3029	1.1350
Pestivirus A	JX297521	VIPR_ALG4_459284085_1	1b	NS4B	Scorpius	2006	-0.2042	0.3019	1.1350
Pestivirus A	KC853440	VIPR_ALG4_507144146_1	1k	NS4B	SuwaNcp	1993	-0.2963	0.3154	1.1265
Pestivirus A	KC853441	VIPR_ALG4_507144148_1	1k	NS4B	SuwaCp	1993	-0.2535	0.3184	1.1265
Pestivirus A	KC695810	VIPR_ALG4_507866685_1	1q	NS4B	camel-6	2010	-0.1037	0.2866	1.1279
Pestivirus A	KC695814	VIPR_ALG4_507866704_1	1b	NS4B	Av69 VEDEVAC	2011	0.0095	0.3144	1.1294
Pestivirus A	KC757383	VIPR_ALG4_511775165_1	1d	NS4B	10JJ-SKR	2010	-0.3342	0.2850	1.1384
Pestivirus A	KC963967	VIPR_ALG4_530291194_1	1b	NS4B	12F004	2012	0.0233	0.2824	1.1344
Pestivirus A	KF772785	VIPR_ALG4_575471151_1	1b	NS4B	CC13B	2013	0.0668	0.3030	1.1368
Pestivirus A	KF896608	VIPR_ALG4_586616532_1	1c	NS4B	Bega-like	2012	-0.3321	0.3166	1.1314
Pestivirus A	KF835697	VIPR_ALG4_597437474_1	1b	NS4B	AU526	2013	0.0755	0.2961	1.1344
Pestivirus A	KJ541471	VIPR_ALG4_633265982_1	1a	NS4B	GS5	2013	-0.0946	0.2942	1.1269
Pestivirus A	KJ689448	VIPR_ALG4_635172915_1	1b	NS4B	GX4	2012	0.0095	0.3144	1.1294
Pestivirus A	KF501393	VIPR_ALG4_669206614_1	1b	NS4B	BVDV JL-1	2009	0.2248	0.2939	1.1394
Pestivirus A	AJ133738	VIPR_ALG4_7960754_766	1a	NS4B	type 1	1963	-0.1968	0.2937	1.1285
Pestivirus A	KP941581	VIPR_ALG4_800924313_1	1b	NS4B	USMARC-51998	2014	-0.0806	0.3005	1.1344
Pestivirus A	KP941583	VIPR_ALG4_800924317_1	1b	NS4B	USMARC-53874	2014	-0.0265	0.2929	1.1344
Pestivirus A	KP941584	VIPR_ALG4_800924319_1	1a	NS4B	USMARC-53875	2014	-0.1611	0.3145	1.1359
Pestivirus A	KP941586	VIPR_ALG4_800924323_1	1a	NS4B	USMARC-55477	2014	-0.0971	0.2925	1.1308
Pestivirus A	KP941587	VIPR_ALG4_800924325_1	1b	NS4B	USMARC-55478	2014	-0.0069	0.2928	1.1293
Pestivirus A	KP941588	VIPR_ALG4_800924327_1	1b	NS4B	USMARC-55922	2014	0.0747	0.2943	1.1356
Pestivirus A	KP941589	VIPR_ALG4_800924329_1	1b	NS4B	USMARC-55923	2014	-0.1005	0.3022	1.1344
Pestivirus A	KP941590	VIPR_ALG4_800924331_1	1b	NS4B	USMARC-55924	2014	-0.1644	0.3003	1.1273
Pestivirus A	KP941591	VIPR_ALG4_800924333_1	1b	NS4B	USMARC-55925	2014	-0.0780	0.2986	1.1343
Pestivirus A	KP941592	VIPR_ALG4_800924335_1	1b	NS4B	USMARC-55926	2014	-0.3790	0.2879	1.1405
Pestivirus A	KP313732	VIPR_ALG4_816850387_1	1e	NS4B	Carlito	2014	0.0708	0.2936	1.1315
Pestivirus A	KR029825	VIPR_ALG4_887497286_1	1b	NS4B	Egy/Ismailia/2014	2014	-0.1402	0.2901	1.1429
Pestivirus A	LC089875	VIPR_ALG4_939106262_1	1o	NS4B	IS26/01ncp	2001	-0.2654	0.2873	1.1276
Pestivirus A	LC089876	VIPR_ALG4_939106264_1	1n	NS4B	Shitara/02/06	2006	-0.0516	0.2929	1.1300
Pestivirus A	KR866116	VIPR_ALG4_941508008_1	1m	NS4B	SD-15	2015	-0.4203	0.2908	1.1327
Pestivirus A	KU200260	VIPR_ALG4_972905813_1	1b	NS4B	BE/061536/2014	2014	0.0341	0.2930	1.1314
Pestivirus A	KX577637	VIPR_ALG4_AOR50934_1	1e	NS4B	SLO/2407/2006	2006	-0.2945	0.3049	1.1337
Pestivirus A	KX987157	VIPR_ALG4_APG30987_1	1f	NS4B	SLO/1170/2000	2000	-0.1294	0.2900	1.1318
Pestivirus A	KX857724	VIPR_ALG4_APZ85839_1	1i	NS4B	ACM/BR/2016	2016	-0.1725	0.2871	1.1251
Pestivirus A	KY849592	VIPR_ALG4_ART90617_1	1d	NS4B	SLO/2416/2002	2002	-0.3278	0.3032	1.1349
Pestivirus A	MF278651	VIPR_ALG4_ASW18434_1	1b	NS4B	XZ01	2016	0.0509	0.3135	1.1318
Pestivirus A	MF278652	VIPR_ALG4_ASW18435_1	1b	NS4B	XZ02	2016	0.0185	0.3208	1.1311
Pestivirus A	MF693403	VIPR_ALG4_ATG71375_1	1a	NS4B	UNKNOWN-MF693403	2016	0.0197	0.3533	1.1295
Pestivirus A	KY964311	VIPR_ALG4_ATN39078_1	1b	NS4B	Y2	2014	-0.0835	0.3190	1.1352
Pestivirus A	MF172980	VIPR_ALG4_AVI10261_1_	1c	NS4B	GSTZ	2012	0.0076	0.2948	1.1343
Pestivirus A	MH379638	VIPR_ALG4_AWW14171_	1a	NS4B	Ho916	1993	-0.2690	0.2894	1.1289
Pestivirus A	MG950344	VIPR_ALG4_AWW87346_	1b	NS4B	AU526	2014	0.1134	0.2975	1.1344
Pestivirus A	MG950345	VIPR_ALG4_AWW87347_	1b	NS4B	B1	2015	0.0755	0.2961	1.1344
Pestivirus A	MG950346	VIPR_ALG4_AWW87348_	1b	NS4B	B2	2015	0.0755	0.2961	1.1344

Species according to VIPRBRC	GenBank Accession	GenBank Protein Accession	Subgenotype	Protein	Strain Name	Collection Year	SVM Patho. Score	Vaxijen Antig. Score	Averged score of EMBOSS motifs
Pestivirus A	MG950347	VIPR_ALG4_AWW87349_	1b	NS4B	B3	2015	0.0972	0.2978	1.1344
Pestivirus A	MG950348	VIPR_ALG4_AWW87350_	1b	NS4B	B4	2015	0.1134	0.2975	1.1344
Pestivirus A	MG950349	VIPR_ALG4_AWW87351_	1b	NS4B	B5	2015	0.1134	0.2975	1.1344
Pestivirus A	MG950350	VIPR_ALG4_AWW87352_	1b	NS4B	B6	2015	0.1134	0.2975	1.1344
Pestivirus A	MG950351	VIPR_ALG4_AWW87353_	1b	NS4B	O1	2015	0.1134	0.2975	1.1344
Pestivirus A	MG950352	VIPR_ALG4_AWW87354_	1b	NS4B	O2	2015	0.1134	0.2975	1.1344
Pestivirus A	MG950353	VIPR_ALG4_AWW87355_	1b	NS4B	O3	2015	0.1134	0.2975	1.1344
Pestivirus A	MG950354	VIPR_ALG4_AWW87356_	1b	NS4B	O4	2015	0.1134	0.2975	1.1344
Pestivirus A	MG950355	VIPR_ALG4_AWW87357_	1b	NS4B	O5	2015	0.1134	0.2975	1.1344
Pestivirus A	MG950356	VIPR_ALG4_AWW87358_	1b	NS4B	O6	2015	0.1134	0.2975	1.1344
Pestivirus A	MG950357	VIPR_ALG4_AWW87359_	1b	NS4B	B1A	2015	0.1235	0.3023	1.1344
Pestivirus A	MG950358	VIPR_ALG4_AWW87360_	1b	NS4B	B2A	2016	0.1134	0.2975	1.1344
Pestivirus A	MG950359	VIPR_ALG4_AWW87361_	1b	NS4B	B3A	2016	0.1134	0.2975	1.1344
Pestivirus A	MG950360	VIPR_ALG4_AWW87362_	1b	NS4B	B4A	2016	0.1134	0.2975	1.1344
Pestivirus A	MG950361	VIPR_ALG4_AWW87363_	1b	NS4B	B5A	2016	0.1134	0.2975	1.1344
Pestivirus A	MG950362	VIPR_ALG4_AWW87364_	1b	NS4B	B6A	2016	0.1134	0.2975	1.1344
Pestivirus A	MG950363	VIPR_ALG4_AWW87365_	1b	NS4B	O1A	2015	0.1257	0.3010	1.1344
Pestivirus A	MG950364	VIPR_ALG4_AWW87366_	1b	NS4B	O2A	2015	0.1134	0.2975	1.1344
Pestivirus A	MG950365	VIPR_ALG4_AWW87367_	1b	NS4B	O2B	2015	0.1134	0.2975	1.1344
Pestivirus A	MG950366	VIPR_ALG4_AWW87368_	1b	NS4B	O4A	2015	0.1134	0.2975	1.1344
Pestivirus A	MH311874	VIPR_ALG4_AWW87369_	1b	NS4B	B2A d168	2016	0.1134	0.2975	1.1344
Pestivirus A	MH311875	VIPR_ALG4_AWW87370_	1b	NS4B	B3A d168	2016	0.1134	0.2975	1.1344
Pestivirus A	MH311876	VIPR_ALG4_AWW87371_	1b	NS4B	B4A d84	2016	0.1134	0.2975	1.1344
Pestivirus A	MH311877	VIPR_ALG4_AWW87372_	1b	NS4B	B4A d168	2016	0.1134	0.2975	1.1344
Pestivirus A	MH311878	VIPR_ALG4_AWW87373_	1b	NS4B	B5A d84	2016	0.1134	0.2975	1.1344
Pestivirus A	MH311879	VIPR_ALG4_AWW87374_	1b	NS4B	B5A d168	2016	0.1134	0.2975	1.1344
Pestivirus A	MH311880	VIPR_ALG4_AWW87375_	1b	NS4B	B6A d84	2016	0.1134	0.2975	1.1344
Pestivirus A	MH311881	VIPR_ALG4_AWW87376_	1b	NS4B	B6A d168	2016	0.1134	0.2975	1.1344
Pestivirus A	MH379221	VIPR_ALG4_AWW87377_	1b	NS4B	P1	2017	0.1336	0.2923	1.1344
Pestivirus A	MH379222	VIPR_ALG4_AWW87378_	1b	NS4B	P2	2017	0.1336	0.2923	1.1344
Pestivirus A	MH379223	VIPR_ALG4_AWW87379_	1b	NS4B	P5	2017	0.1134	0.2975	1.1344
Pestivirus A	MH379224	VIPR_ALG4_AWW87380_	1b	NS4B	P6	2017	0.1131	0.3224	1.1344
Pestivirus A	MH379225	VIPR_ALG4_AWW87381_	1b	NS4B	P7	2017	0.1134	0.2975	1.1344
Pestivirus A	MH379226	VIPR_ALG4_AWW87382_	1b	NS4B	P5A	2017	0.1336	0.2923	1.1344
Pestivirus A	MH379227	VIPR_ALG4_AWW87383_	1b	NS4B	P5B	2017	0.1336	0.2923	1.1344
Pestivirus A	MH379228	VIPR_ALG4_AWW87384_	1b	NS4B	P5C	2017	0.1134	0.2975	1.1344
Pestivirus A	MH379229	VIPR_ALG4_AWW87385_	1b	NS4B	P5D	2017	0.1336	0.2923	1.1344
Pestivirus A	MH379230	VIPR_ALG4_AWW87386_	1b	NS4B	P5F	2017	0.1134	0.2975	1.1344
Pestivirus A	MH379231	VIPR_ALG4_AWW87387_	1b	NS4B	P7A	2018	0.1134	0.2975	1.1344
Pestivirus A	MH379232	VIPR_ALG4_AWW87388_	1b	NS4B	P7C	2018	0.1134	0.2975	1.1344
Pestivirus A	MH379233	VIPR_ALG4_AWW87389_	1b	NS4B	P7E	2018	0.1134	0.2975	1.1344
Pestivirus A	MH379234	VIPR_ALG4_AWW87390_	1b	NS4B	P7F	2018	0.1134	0.2975	1.1344
Pestivirus A	MH166806	VIPR_ALG4_AYA62524_1	1m	NS4B	XC	2015	-0.2766	0.3069	1.1318
Pestivirus A	MH490943	VIPR_ALG4_AZB53078_1	1b	NS4B	BVDV BJ-2016	2016	-0.0203	0.3098	1.1320
Pestivirus A	MH231153	VIPR_ALG4_AZQ00677_1	1b	NS4B	Nebraska	1990	0.0263	0.3119	1.1378
Pestivirus A	AB078950	VIPR_ALG4_BAC55961_1	1j	NS4B	KS86-1ncp	1986	-0.5093	0.2767	1.1389
Pestivirus A	MH899941	VIPR_ALG4_QCE30388_1	1b	NS4B	SLO/3301/2014	2014	-0.0724	0.2964	1.1334

Species according to VIPRBRC	GenBank Accession	GenBank Protein Accession	Subgenotype	Protein	Strain Name	Collection Year	SVM Patho. Score	Vaxijen Antig. Score	Averged score of EMBOSS motifs
Pestivirus A	MH899942	VIPR_ALG4_QCE30389_1	1e	NS4B	SLO/33529/2015	2015	-0.3150	0.2988	1.1370
Pestivirus A	MH899943	VIPR_ALG4_QCE30390_1	1f	NS4B	SLO/1361/2014	2014	0.0107	0.3003	1.1355
Pestivirus A	MH899944	VIPR_ALG4_QCE30391_1	1f	NS4B	SLO/28537/2017	2017	-0.0784	0.3091	1.1322
Pestivirus A	MH899945	VIPR_ALG4_QCE30392_1	1h	NS4B	SLO/1883/2013	2013	-0.2289	0.2895	1.1364
Pestivirus A	MK102095	VIPR_ALG4_QCQ84262_1	1q	NS4B	20170226	2017	-0.2110	0.3100	1.1327
Pestivirus A	MK509774	VIPR_ALG4_QEK23510_1	1b	NS4B	BVD1b-JH	2008	0.1286	0.2879	1.1346
Pestivirus A	MK775204	VIPR_ALG4_QFX66041_1	1i	NS4B	CA2006	2006	-0.1749	0.2999	1.1285
Pestivirus A	MN188073	VIPR_ALG4_QGZ19414_1	1a	NS4B	PI34	2017	-0.1299	0.2963	1.1331
Pestivirus A	MN188074	VIPR_ALG4_QGZ19415_1	1b	NS4B	PI285	2017	-0.0644	0.3037	1.1343
Pestivirus A	MT079816	VIPR_ALG4_QIM55913_1	1c	NS4B	GXNN1	2018	-0.0799	0.3284	1.1314
Pestivirus A	MN623291	VIPR_ALG4_QLL27013_1	1m	NS4B	NX2019/01	2019	-0.3873	0.3005	1.1241
Pestivirus A	MW014286	VIPR_ALG4_QPJ59878_1	1b	NS4B	GXSS01	2018	0.0095	0.3144	1.1294
Pestivirus A	MW014287	VIPR_ALG4_QPJ59879_1	1b	NS4B	GXSS02	2018	0.0328	0.2975	1.1325
Pestivirus A	MW014288	VIPR_ALG4_QPJ59880_1	1b	NS4B	GXSS03	2018	0.0328	0.2975	1.1325
Pestivirus A	MT977117	VIPR_ALG4_QRZ20359_1	1b	NS4B	BVDV 1b IT16/5	2016	0.1180	0.3004	1.1314
Pestivirus A	MT977118	VIPR_ALG4_QRZ20360_1	1b	NS4B	BVDV 1b IT16/439	2016	0.0934	0.2946	1.1314
Pestivirus A	MT654137	VIPR_ALG4_QVK82311_1	1a	NS4B	20-8536	2020	-0.4165	0.3004	1.1289
Pestivirus A	LT837585	VIPR_ALG4_SLV80196_1	1r	NS4B	UNKNOWN-LT837585	2012	-0.2139	0.3088	1.1299
Pestivirus A	MW054933	VIPR_ALG4_UEC94252_1	1f	NS4B	LA/230/14	2014	-0.0625	0.3002	1.1324
Pestivirus A	MW054934	VIPR_ALG4_UEC94253_1	1f	NS4B	LA/87/05	2005	-0.2028	0.3140	1.1324
Pestivirus A	MW054935	VIPR_ALG4_UEC94254_1	1k	NS4B	TO/197/11	2011	-0.3147	0.3110	1.1333
Pestivirus A	MW054936	VIPR_ALG4_UEC94255_1	1g	NS4B	UM/111/06	2006	-0.0343	0.3164	1.1309
Pestivirus A	MW054937	VIPR_ALG4_UEC94256_1	1k	NS4B	SA/158/09	2009	-0.1888	0.3101	1.1318
Pestivirus A	MW054938	VIPR_ALG4_UEC94257_1	1k	NS4B	SA/159/09	2009	-0.2026	0.3092	1.1331
Pestivirus A	MW054939	VIPR_ALG4_UEC94258_1	1f	NS4B	LO/151/09	2009	-0.0625	0.3002	1.1324
Pestivirus A	MW054940	VIPR_ALG4_UEC94259_1	1e	NS4B	MA/101/05	2005	-0.3395	0.3029	1.1324
Pestivirus A	MW250796	VIPR_ALG4_UEC94260_1	1i	NS4B	58-1	2008	-0.3816	0.3019	1.1296
Pestivirus A	MW250797	VIPR_ALG4_UEC94261_1	1i	NS4B	58-2	2008	-0.3816	0.3019	1.1296
Pestivirus A	MW250798	VIPR_ALG4_UEC94262_1	1a	NS4B	62-2	2008	-0.2692	0.3043	1.1294
Pestivirus A	MW250799	VIPR_ALG4_UEC94263_1	1a	NS4B	63-1	2008	-0.2326	0.3102	1.1342
Pestivirus A	MW250800	VIPR_ALG4_UEC94264_1	1d	NS4B	67-1	2008	-0.3804	0.2959	1.1349
Pestivirus A	MW250801	VIPR_ALG4_UEC94265_1	1d	NS4B	67-2	2008	-0.3804	0.2959	1.1349
Pestivirus A	MW250802	VIPR_ALG4_UEC94266_1	1e	NS4B	68-1	2008	-0.3825	0.3065	1.1275
Pestivirus A	MW250803	VIPR_ALG4_UEC94267_1	1i	NS4B	69-1	2008	-0.2807	0.3066	1.1290
Pestivirus A	MW655625	VIPR_ALG4_UEC94268_1	1h	NS4B	CH-04-01b	2004	-0.2456	0.2906	1.1371
Pestivirus A	MW655626	VIPR_ALG4_UEC94269_1	1e	NS4B	Maria	2004	-0.0414	0.2749	1.1405
Pestivirus A	MW655627	VIPR_ALG4_UEC94270_1	1e	NS4B	R2000-95	1995	-0.3309	0.3020	1.1303
Pestivirus A	MW655628	VIPR_ALG4_UEC94271_1	1k	NS4B	R3230-95	1995	-0.2019	0.3179	1.1338
Pestivirus A	MW655629	VIPR_ALG4_UEC94272_1	1h	NS4B	R3572-90	1990	-0.2959	0.2921	1.1322
Pestivirus A	MW655630	VIPR_ALG4_UEC94273_1	1k	NS4B	R5013-96	1996	-0.2506	0.3178	1.1318
Pestivirus A	MW655631	VIPR_ALG4_UEC94274_1	1e	NS4B	S03-1175	2003	-0.2196	0.3044	1.1403
Pestivirus A	MW655632	VIPR_ALG4_UEC94275_1	1h	NS4B	SM09-20	2002	-0.3217	0.2902	1.1350
Pestivirus A	MW713361	VIPR_ALG4_UEC94276_1	1a	NS4B	BoAEC1190	2007	-0.2458	0.2980	1.1306
Pestivirus A	MW713362	VIPR_ALG4_UEC94277_1	1b	NS4B	PI819	2017	0.1134	0.2975	1.1344
Pestivirus A	MW732738	VIPR_ALG4_UEC94278_1	1a	NS4B	PI407	2015	-0.3124	0.3043	1.1285
Pestivirus A	MW732739	VIPR_ALG4_UEC94279_1	1a	NS4B	YandaSpl	1993	-0.1021	0.2962	1.1244
Pestivirus A	MZ188972	VIPR_ALG4_UML14262_1	1q	NS4B	HB-1	2020	-0.3326	0.3173	1.1263

Species according to VIPRBRC	GenBank Accession	GenBank Protein Accession	Subgenotype	Protein	Strain Name	Collection Year	SVM Patho. Score	Vaxijen Antig. Score	Averged score of EMBOSS motifs
Pestivirus A	ON337882	VIPR_ALG4_USZ80113_1	1c	NS4B	NM2103	2021	-0.3316	0.3157	1.1301
Pestivirus B	KX170299	APT70822.1	2a	Npro	V002	1999	-0.1960	0.3544	1.1392
Pestivirus B	KX170298	APT70821.1	2a	Npro	V005	1999	-0.1960	0.3544	1.1392
Pestivirus B	KX170288	APT70811.1	2a	Npro	V017	2003	-0.2180	0.3359	1.1264
Pestivirus B	KX170282	APT70805.1	2a	Npro	V019	2004	-0.1220	0.3501	1.1304
Pestivirus B	KX170300	APT70823.1	2a	Npro	V021	2005	-0.1960	0.3544	1.1392
Pestivirus B	KX170290	APT70813.1	2a	Npro	V023	2006	-0.0750	0.3103	1.1358
Pestivirus B	KX170294	APT70817.1	2a	Npro	V024	2006	-0.2194	0.3331	1.1392
Pestivirus B	KX170295	APT70818.1	2a	Npro	V025	2006	-0.1902	0.3275	1.1226
Pestivirus B	KX170280	APT70803.1	2a	Npro	V028	2006	-0.0739	0.3189	1.1164
Pestivirus B	KX170297	APT70820.1	2a	Npro	V037	2008	-0.1960	0.3544	1.1392
Pestivirus B	KX170291	APT70814.1	2a	Npro	V044	2008	0.0033	0.3686	1.1277
Pestivirus B	KX170284	APT70807.1	2a	Npro	V047	2009	-0.3306	0.3226	1.1360
Pestivirus B	KX170292	APT70815.1	2a	Npro	V051	2010	-0.2557	0.3324	1.1358
Pestivirus B	KX170281	APT70804.1	2a	Npro	V062	2005	-0.1754	0.3377	1.1264
Pestivirus B	KX170283	APT70806.1	2a	Npro	V063	2005	-0.2332	0.3308	1.1369
Pestivirus B	KX170285	APT70808.1	2a	Npro	V076	2011	-0.3897	0.3298	1.1317
Pestivirus B	KX170301	APT70824.1	2a	Npro	V079	2010	-0.2868	0.3491	1.1392
Pestivirus B	KX170287	APT70810.1	2a	Npro	V081	2009	-0.2786	0.3431	1.1226
Pestivirus B	KX170289	APT70812.1	2a	Npro	V082	2008	-0.0890	0.3434	1.1172
Pestivirus B	KX170286	APT70809.1	2a	Npro	V085	2007	-0.4150	0.3140	1.1370
Pestivirus B	KX170278	APT70801.1	2a	Npro	V086	2006	0.0707	0.3384	1.1207
Pestivirus B	KX170277	APT70800.1	2a	Npro	V088	2005	-0.0958	0.3475	1.1164
Pestivirus B	KX170279	APT70802.1	2a	Npro	V089	2004	0.0707	0.3384	1.1207
Pestivirus B	KX170276	APT70799.1	2a	Npro	V095	1999	-0.0812	0.3389	1.1190
Pestivirus B	KX170296	APT70819.1	2a	Npro	V097	1998	-0.2312	0.3488	1.1286
Pestivirus B	FJ431189	VIPR_ALG4_21636083	2a	Npro	53099	2007	-0.2058	0.3442	1.1199
Pestivirus B	FJ431190	VIPR_ALG4_21636083	2a	Npro	53100	2007	-0.2058	0.3442	1.1199
Pestivirus B	FJ431192	VIPR_ALG4_21636085	2a	Npro	65	2007	-0.2058	0.3442	1.1199
Pestivirus B	FJ431193	VIPR_ALG4_21636086	2a	Npro	68	2007	-0.0907	0.3499	1.1199
Pestivirus B	FJ431194	VIPR_ALG4_21636086	2a	Npro	73	2007	-0.4041	0.3726	1.1199
Pestivirus B	KT875134	VIPR_ALG4_10037029	2a	Npro	12W	2005	-0.2022	0.3296	1.1264
Pestivirus B	KT875135	VIPR_ALG4_10037029	2a	Npro	13Y	2005	-0.2022	0.3296	1.1264
Pestivirus B	KP057803	VIPR_ALG4_80287536	2a	Npro	24515	1993	-0.2194	0.3331	1.1392
Pestivirus B	KT875136	VIPR_ALG4_10037029	2a	Npro	27Y	2005	-0.2022	0.3296	1.1264
Pestivirus B	KT875137	VIPR_ALG4_10037029	2a	Npro	29Y	2005	-0.2022	0.3296	1.1264
Pestivirus B	KT875138	VIPR_ALG4_10037029	2a	Npro	2Y	2005	-0.2022	0.3296	1.1264
Pestivirus B	KT875139	VIPR_ALG4_10037029	2a	Npro	32W	2005	-0.2022	0.3296	1.1264
Pestivirus B	KT875140	VIPR_ALG4_10037029	2a	Npro	34Y	2005	-0.2022	0.3296	1.1264
Pestivirus B	KT875141	VIPR_ALG4_10037029	2a	Npro	36W	2005	-0.1525	0.3430	1.1264
Pestivirus B	KT875142	VIPR_ALG4_10037029	2a	Npro	41Y	2005	-0.2022	0.3296	1.1264
Pestivirus B	KT875143	VIPR_ALG4_10037029	2a	Npro	42W	2005	-0.2022	0.3296	1.1264
Pestivirus B	KT875144	VIPR_ALG4_10037029	2a	Npro	43Y	2005	-0.2555	0.3192	1.1360
Pestivirus B	KT875145	VIPR_ALG4_10037029	2a	Npro	47Y	2005	-0.2022	0.3296	1.1264
Pestivirus B	KT875146	VIPR_ALG4_10037029	2a	Npro	50Y	2005	-0.2022	0.3296	1.1264
Pestivirus B	KT875147	VIPR_ALG4_10037029	2a	Npro	51W	2005	-0.2022	0.3296	1.1264
Pestivirus B	KT875148	VIPR_ALG4_10037029	2a	Npro	51Y	2005	-0.2022	0.3296	1.1264

Species according to VIPRBRC	GenBank Accession	GenBank Protein Accession	Subgenotype	Protein	Strain Name	Collection Year	SVM Patho. Score	Vaxijen Antig. Score	Averged score of EMBOSS motifs
Pestivirus B	KT875149	VIPR_ALG4_10037029	2a	Npro	53W	2005	-0.3636	0.3424	1.1264
Pestivirus B	KT875150	VIPR_ALG4_10037029	2a	Npro	58W	2005	-0.2022	0.3296	1.1264
Pestivirus B	KT875151	VIPR_ALG4_10037029	2a	Npro	58Y	2005	-0.2022	0.3296	1.1264
Pestivirus B	KT875152	VIPR_ALG4_10037029	2a	Npro	5Y	2005	-0.2022	0.3296	1.1264
Pestivirus B	KT875153	VIPR_ALG4_10037029	2a	Npro	62Y	2005	-0.2022	0.3296	1.1264
Pestivirus B	KT875154	VIPR_ALG4_10037029	2a	Npro	65Y	2005	-0.2022	0.3296	1.1264
Pestivirus B	KT875155	VIPR_ALG4_10037029	2a	Npro	67Y	2005	-0.2022	0.3296	1.1264
Pestivirus B	KT875156	VIPR_ALG4_10037029	2a	Npro	68W	2005	-0.2022	0.3296	1.1264
Pestivirus B	KT875157	VIPR_ALG4_10037029	2a	Npro	71Y	2005	-0.2022	0.3296	1.1264
Pestivirus B	KT875158	VIPR_ALG4_10037029	2a	Npro	73Y	2005	-0.2022	0.3296	1.1264
Pestivirus B	KT875159	VIPR_ALG4_10037029	2a	Npro	74Y	2005	-0.2022	0.3296	1.1264
Pestivirus B	KT875160	VIPR_ALG4_10037029	2a	Npro	75W	2005	-0.2022	0.3296	1.1264
Pestivirus B	KT875161	VIPR_ALG4_10037029	2a	Npro	75Y	2005	-0.2022	0.3296	1.1264
Pestivirus B	KT875162	VIPR_ALG4_10037029	2a	Npro	76Y	2005	-0.2022	0.3296	1.1264
Pestivirus B	KT875163	VIPR_ALG4_10037029	2a	Npro	78W	2005	-0.2022	0.3296	1.1264
Pestivirus B	KT875164	VIPR_ALG4_10037029	2a	Npro	79W	2005	-0.2022	0.3296	1.1264
Pestivirus B	KT875165	VIPR_ALG4_10037029	2a	Npro	7W	2005	-0.2022	0.3296	1.1264
Pestivirus B	KT875166	VIPR_ALG4_10037029	2a	Npro	82W	2005	-0.2022	0.3296	1.1264
Pestivirus B	KT875167	VIPR_ALG4_10037029	2a	Npro	83Y	2005	-0.2022	0.3296	1.1264
Pestivirus B	KT875168	VIPR_ALG4_10037029	2a	Npro	90W	2005	-0.2022	0.3296	1.1264
Pestivirus B	KT875169	VIPR_ALG4_10037030	2a	Npro	91W	2005	-0.2022	0.3296	1.1264
Pestivirus B	MN824468	VIPR_ALG4_QJF1227	2a	Npro	CPAE_contamination/2018	2018	-0.2411	0.3920	1.1337
Pestivirus B	KR093034	VIPR_ALG4_92904888	2a	Npro	NY-93	1993	-0.2194	0.3331	1.1392
Pestivirus B	KF925365	VIPR_ALG4_58659881	2b	Npro	SD1301	2012	-0.4593	0.4038	1.1203
Pestivirus B	KX170293	APT70816.1	2a	Npro	V065	2006	-0.1516	0.3338	1.1311
Pestivirus B	EU747875	VIPR_ALG4_19033620	2b	Npro	17237	2004	-0.8714	0.4435	1.1333
Pestivirus B	MW006485	VIPR_ALG4_QPF4972	2b	Npro	HEN01	2014	-0.4593	0.4038	1.1203
Pestivirus B	MH231142	VIPR_ALG4_AZQ0066	2c	Npro	Parker	1991	0.0103	0.2903	1.1318
Pestivirus B	MH806435	VIPR_ALG4_AZP5716	2a	Npro	1336H	2005	-0.3715	0.3861	1.1340
Pestivirus B	MH231136	VIPR_ALG4_AZQ0066	2a	Npro	JV14	1998	-0.1910	0.3235	1.1309
Pestivirus B	MK599227	VIPR_ALG4_QEU5262	2a	Npro	SD-1	2016	-0.1774	0.3358	1.1340
Pestivirus B	HG426488	VIPR_ALG4_61932575	2c	Npro	NRW 19-13-1_Dup(+)	2013	0.3649	0.2901	1.1318
Pestivirus B	HG426487	VIPR_ALG4_61932574	2c	Npro	NRW 19-13-1_Dup(-)	2013	0.3649	0.2901	1.1318
Pestivirus B	MH231138	VIPR_ALG4_AZQ0066	2a	Npro	MnFetus	1991	-0.2536	0.3395	1.1337
Pestivirus B	MH231131	VIPR_ALG4_AZQ0065	2a	Npro	AU501	2006	-0.2403	0.3309	1.1264
Pestivirus B	MH806434	VIPR_ALG4_AZP5716	2a	Npro	125c	1990	0.0436	0.3046	1.1423
Pestivirus B	MH231151	VIPR_ALG4_AZQ0067	2e	Npro	14622	2005	0.2733	0.2329	1.1264
Pestivirus B	MH231152	VIPR_ALG4_AZQ0067	2e	Npro	2412	1989	0.0906	0.2909	1.1264
Pestivirus B	MH231127	VIPR_ALG4_AZQ0065	2a	Npro	53637c	2004	-0.2981	0.3407	1.1358
Pestivirus B	MH231149	VIPR_ALG4_AZQ0067	2e	Npro	Short	1989	0.2733	0.2329	1.1264
Pestivirus B	MH231129	VIPR_ALG4_AZQ0065	2a	Npro	5912c	1995	0.0102	0.3167	1.1311
Pestivirus B	KJ000672	VIPR_ALG4_59423572	2b	Npro	SD1301	2012	-0.4593	0.4038	1.1203
Pestivirus B	MH231148	VIPR_ALG4_AZQ0067	2e	Npro	12-149150	2012	0.2786	0.3159	1.1264
Pestivirus B	MH231150	VIPR_ALG4_AZQ0067	2e	Npro	12-151955-317	2012	0.3027	0.2900	1.1289
Pestivirus B	MH231133	VIPR_ALG4_AZQ0065	2e	Npro	B69519c	2006	0.0906	0.2909	1.1264
Pestivirus B	HG426495	VIPR_ALG4_61932576	2c	Npro	VOE 4407	2007	-0.1588	0.2923	1.1285
Pestivirus B	HG426480	VIPR_ALG4_61932573	2c	Npro	D37-13-2_Dup(+)	2013	0.3649	0.2901	1.1318

Species according to VIPRBRC	GenBank Accession	GenBank Protein Accession	Subgenotype	Protein	Strain Name	Collection Year	SVM Patho. Score	Vaxijen Antig. Score	Averged score of EMBOSS motifs
Pestivirus B	HG426482	VIPR_ALG4_61932573	2c	Npro	D75-13-609_Dup(+)	2013	0.3649	0.2901	1.1318
Pestivirus B	HG426486	VIPR_ALG4_61932574	2c	Npro	NRW 14-13_Dup(+)	2013	0.3649	0.2901	1.1318
Pestivirus B	HG426491	VIPR_ALG4_61932575	2c	Npro	Potsdam 1600	2000	0.0103	0.2903	1.1318
Pestivirus B	MH231141	VIPR_ALG4_AZQ0066	2a	Npro	PI28	2016	-0.3263	0.3663	1.1392
Pestivirus B	HG426494	VIPR_ALG4_61932576	2c	Npro	SH2210-23	2010	-0.0559	0.2951	1.1372
Pestivirus B	MH806436	VIPR_ALG4_AZP5716	2a	Npro	296c	1995	-0.2170	0.3154	1.1428
Pestivirus B	MH231137	VIPR_ALG4_AZQ0066	2a	Npro	MadSpl	1991	-0.2399	0.3529	1.1392
Pestivirus B	KP941585	VIPR_ALG4_80092432	2a	Npro	USMARC-55476	2014	-0.0618	0.3418	1.1337
Pestivirus B	KT832820	VIPR_ALG4_99822633	2a	Npro	USMARC-60767	2014	-0.2810	0.3678	1.1392
Pestivirus B	MH231134	VIPR_ALG4_AZQ0065	2a	Npro	B9497	1997	-0.1155	0.2931	1.1190
Pestivirus B	MN527354	VIPR_ALG4_QLH0204	2a	Npro	GS2018	2018	-0.2617	0.3344	1.1188
Pestivirus B	MH231144	VIPR_ALG4_AZQ0066	2a	Npro	Sanderson6319	1992	-0.2671	0.3444	1.1388
Pestivirus B	HG426493	VIPR_ALG4_61932576	2c	Npro	SH2210-17	2010	-0.1148	0.2945	1.1318
Pestivirus B	KT832817	VIPR_ALG4_99822632	2a	Npro	USMARC-60764	2014	-0.1952	0.3099	1.1188
Pestivirus B	KT832822	VIPR_ALG4_99822633	2a	Npro	USMARC-60779	2014	-0.2523	0.3510	1.1392
Pestivirus B	MH231125	VIPR_ALG4_AZQ0064	2a	Npro	2139	1992	-0.4853	0.3629	1.1273
Pestivirus B	KP941582	VIPR_ALG4_80092431	2c	Npro	USMARC-53873	2014	0.3776	0.3033	1.1258
Pestivirus B	KT832819	VIPR_ALG4_99822632	2a	Npro	USMARC-60766	2014	-0.1990	0.3334	1.1337
Pestivirus B	KT832821	VIPR_ALG4_99822633	2c	Npro	USMARC-60768	2014	0.1008	0.2908	1.1111
Pestivirus B	HG426479	VIPR_ALG4_61932573	2c	Npro	D37-13-2_Dup(-)	2013	0.3649	0.2901	1.1318
Pestivirus B	HG426481	VIPR_ALG4_61932573	2c	Npro	D75-13-609_Dup(-)	2013	0.3649	0.2901	1.1318
Pestivirus B	MH231123	VIPR_ALG4_AZQ0064	2a	Npro	10406	1993	-0.2114	0.2951	1.1358
Pestivirus B	HG426492	VIPR_ALG4_61932575	2c	Npro	SH2210-14	2010	-0.1148	0.2945	1.1318
Pestivirus B	KT832823	VIPR_ALG4_99822633	2a	Npro	USMARC-60780	2014	-0.0670	0.3204	1.1423
Pestivirus B	HG426485	VIPR_ALG4_61932574	2c	Npro	NRW 14-13_Dup(-)	2013	0.3649	0.2901	1.1318
Pestivirus B	MH231126	VIPR_ALG4_AZQ0065	2b	Npro	3237	1990	0.1367	0.3542	1.1310
Pestivirus B	MH806438	VIPR_ALG4_AZP5716	2a	Npro	McCart_c	1989	-0.2380	0.3599	1.1337
Pestivirus B	KT832818	VIPR_ALG4_99822632	2a	Npro	USMARC-60765	2014	-0.2403	0.3486	1.1373
Pestivirus B	MH231124	VIPR_ALG4_AZQ0064	2e	Npro	1786c	1989	0.0906	0.2909	1.1264
Pestivirus B	HG426483	VIPR_ALG4_61932574	2c	Npro	NRW 12-13_Dup(-)	2013	0.3649	0.2901	1.1318
Pestivirus B	HG426484	VIPR_ALG4_61932574	2c	Npro	NRW 12-13_Dup(+)	2013	0.3649	0.2901	1.1318
Pestivirus B	HG426489	VIPR_ALG4_61932575	2c	Npro	NRW 19-13-8_Dup(-)	2013	0.3649	0.2901	1.1318
Pestivirus B	HG426490	VIPR_ALG4_61932575	2c	Npro	NRW 19-13-8_Dup(+)	2013	0.3649	0.2901	1.1318
Pestivirus B	MH231130	VIPR_ALG4_AZQ0065	2a	Npro	95-1501	1998	-0.2380	0.3599	1.1337
Pestivirus B	MH231132	VIPR_ALG4_AZQ0065	2a	Npro	AzSpl	1997	-0.1891	0.2903	1.1445
Pestivirus B	MH231128	VIPR_ALG4_AZQ0065	2a	Npro	570152	1992	-0.1197	0.3463	1.1385
Pestivirus B	MH231135	VIPR_ALG4_AZQ0065	2a	Npro	BV1907	1995	-0.2194	0.3331	1.1392
Pestivirus B	JF714967	VIPR_ALG4_34657797	2a	Npro	HLJ-10	2011	-0.1770	0.3358	1.1340
Pestivirus B	MH231139	VIPR_ALG4_AZQ0066	2a	Npro	Olwein #12	1990	0.0273	0.3066	1.1336
Pestivirus B	MH231140	VIPR_ALG4_AZQ0066	2a	Npro	PA	1992	-0.0894	0.3279	1.1278
Pestivirus B	MH231143	VIPR_ALG4_AZQ0066	2a	Npro	RS886	2014	-0.1990	0.3334	1.1337
Pestivirus B	HQ258810	VIPR_ALG4_31199027	2a	Npro	SH-28	2009	-0.1441	0.3143	1.1380
Pestivirus B	MH231145	VIPR_ALG4_AZQ0066	2a	Npro	Victor301	1990	-0.1910	0.3235	1.1309
Pestivirus B	MH231146	VIPR_ALG4_AZQ0067	2a	Npro	WiscA	1991	-0.2314	0.3428	1.1337
Pestivirus B	FJ527854	VIPR_ALG4_22961018	2a	Npro	XJ-04	2004	-0.3162	0.3103	1.1228
Pestivirus B	KC963968	VIPR_ALG4_53029119	2a	Npro	11F011	2011	-0.2536	0.3395	1.1337
Pestivirus B	MH806437	VIPR_ALG4_AZP5716	2a	Npro	9231	2004	-0.2407	0.3539	1.1304

Species according to VIPRBRC	GenBank Accession	GenBank Protein Accession	Subgenotype	Protein	Strain Name	Collection Year	SVM Patho. Score	Vaxijen Antig. Score	Averged score of EMBOSS motifs
Pestivirus B	MG879027	VIPR_ALG4_AVA3071	2a	Npro	CN10.2015.821	2014	-0.3214	0.3660	1.1264
Pestivirus B	KX096718	VIPR_ALG4_1113017C	2a	Npro	HB-1511	2015	-0.1513	0.3172	1.1313
Pestivirus B	LC649064	VIPR_ALG4_BDB0736	2c	Npro	KZ-91-NCP	1991	-0.1216	0.2737	1.1318
Pestivirus B	MH231147	VIPR_ALG4_AZQ0067	2c	Npro	PI12	2016	-0.0402	0.2819	1.1229
Pestivirus B	MW168422	VIPR_ALG4_QZM0693	2a	Npro	YNJG2020	2020	-0.3071	0.3250	1.1350
Pestivirus B	HQ444199	VIPR_ALG4_32680714	2a	Npro	Ind141353	2007	0.0217	0.2879	1.1478
Pestivirus B	GQ888686	VIPR_ALG4_27071949	2a	Npro	JZ05-1	2005	-0.0484	0.3254	1.1455
Pestivirus B	KY488630	VIPR_ALG4_AQS2337	2a	Npro	FarsB	2014	-0.3419	0.2864	1.1445
Pestivirus B	MF157331	VIPR_ALG4_AWB3605	2a	Npro	Ind_3012339	2015	-0.2398	0.3633	1.1237
Pestivirus B	KP743042	AKT26156.1	2b	Npro	LV/Hipra01/12	2012	-0.2754	0.4303	1.1255
Pestivirus B	KX170596	APT71119.1	2a	NS5A	V076	2011	-0.8892	0.5764	1.1126
Pestivirus B	KX170597	APT71120.1	2a	NS5A	V085	2007	-0.8762	0.5803	1.1133
Pestivirus B	KX170595	APT71118.1	2a	NS5A	V065	2006	-0.8661	0.6017	1.1128
Pestivirus B	KX838370	VIPR_ALG4_AOS5274	2a	NS5A	Ind141353	2007	-0.9871	0.6255	1.1137
Pestivirus B	KT875139	VIPR_ALG4_10037029	2a	NS5A	32W	2005	-0.9086	0.5650	1.1137
Pestivirus B	MN824468	VIPR_ALG4_QJF1227	2a	NS5A	CPAE_contamination/2018	2018	-0.7711	0.6199	1.1111
Pestivirus B	KT875134	VIPR_ALG4_10037029	2a	NS5A	12W	2005	-0.9086	0.5650	1.1137
Pestivirus B	KT875135	VIPR_ALG4_10037029	2a	NS5A	13Y	2005	-0.8939	0.5651	1.1137
Pestivirus B	KT875136	VIPR_ALG4_10037029	2a	NS5A	27Y	2005	-0.9086	0.5650	1.1137
Pestivirus B	KT875137	VIPR_ALG4_10037029	2a	NS5A	29Y	2005	-0.9086	0.5650	1.1137
Pestivirus B	KT875138	VIPR_ALG4_10037029	2a	NS5A	2Y	2005	-0.9086	0.5650	1.1137
Pestivirus B	KT875140	VIPR_ALG4_10037029	2a	NS5A	34Y	2005	-0.9086	0.5650	1.1137
Pestivirus B	KT875141	VIPR_ALG4_10037029	2a	NS5A	36W	2005	-0.8939	0.5651	1.1137
Pestivirus B	KT875142	VIPR_ALG4_10037029	2a	NS5A	41Y	2005	-0.9086	0.5650	1.1137
Pestivirus B	KT875143	VIPR_ALG4_10037029	2a	NS5A	42W	2005	-0.9086	0.5650	1.1137
Pestivirus B	KT875144	VIPR_ALG4_10037029	2a	NS5A	43Y	2005	-0.8939	0.5651	1.1137
Pestivirus B	KT875145	VIPR_ALG4_10037029	2a	NS5A	47Y	2005	-0.8664	0.5728	1.1137
Pestivirus B	KT875146	VIPR_ALG4_10037029	2a	NS5A	50Y	2005	-0.9086	0.5650	1.1137
Pestivirus B	KT875147	VIPR_ALG4_10037029	2a	NS5A	51W	2005	-0.9086	0.5650	1.1137
Pestivirus B	KT875148	VIPR_ALG4_10037029	2a	NS5A	51Y	2005	-0.8981	0.5629	1.1108
Pestivirus B	KT875149	VIPR_ALG4_10037029	2a	NS5A	53W	2005	-0.9086	0.5650	1.1137
Pestivirus B	KT875150	VIPR_ALG4_10037029	2a	NS5A	58W	2005	-0.9086	0.5650	1.1137
Pestivirus B	KT875151	VIPR_ALG4_10037029	2a	NS5A	58Y	2005	-0.9086	0.5650	1.1137
Pestivirus B	KT875152	VIPR_ALG4_10037029	2a	NS5A	5Y	2005	-0.9086	0.5650	1.1137
Pestivirus B	KT875153	VIPR_ALG4_10037029	2a	NS5A	62Y	2005	-0.9334	0.5671	1.1140
Pestivirus B	KT875154	VIPR_ALG4_10037029	2a	NS5A	65Y	2005	-0.9086	0.5650	1.1137
Pestivirus B	KT875155	VIPR_ALG4_10037029	2a	NS5A	67Y	2005	-0.9054	0.5677	1.1122
Pestivirus B	KT875156	VIPR_ALG4_10037029	2a	NS5A	68W	2005	-0.8939	0.5651	1.1137
Pestivirus B	KT875157	VIPR_ALG4_10037029	2a	NS5A	71Y	2005	-0.8475	0.5620	1.1133
Pestivirus B	KT875158	VIPR_ALG4_10037029	2a	NS5A	73Y	2005	-0.9086	0.5650	1.1137
Pestivirus B	KT875159	VIPR_ALG4_10037029	2a	NS5A	74Y	2005	-0.9086	0.5650	1.1137
Pestivirus B	KT875160	VIPR_ALG4_10037029	2a	NS5A	75W	2005	-0.9086	0.5650	1.1137
Pestivirus B	KT875161	VIPR_ALG4_10037029	2a	NS5A	75Y	2005	-0.9086	0.5650	1.1137
Pestivirus B	KT875162	VIPR_ALG4_10037029	2a	NS5A	76Y	2005	-0.9086	0.5650	1.1137
Pestivirus B	KT875163	VIPR_ALG4_10037029	2a	NS5A	78W	2005	-0.9086	0.5650	1.1137
Pestivirus B	KT875164	VIPR_ALG4_10037029	2a	NS5A	79W	2005	-0.8939	0.5651	1.1137
Pestivirus B	KT875165	VIPR_ALG4_10037029	2a	NS5A	7W	2005	-0.9086	0.5650	1.1137

Species according to VIPRBRC	GenBank Accession	GenBank Protein Accession	Subgenotype	Protein	Strain Name	Collection Year	SVM Patho. Score	Vaxijen Antig. Score	Averged score of EMBOSS motifs
Pestivirus B	KT875166	VIPR_ALG4_10037029	2a	NS5A	82W	2005	-0.9086	0.5650	1.1137
Pestivirus B	KT875167	VIPR_ALG4_10037029	2a	NS5A	83Y	2005	-0.9086	0.5650	1.1137
Pestivirus B	KT875168	VIPR_ALG4_10037029	2a	NS5A	90W	2005	-0.8939	0.5651	1.1137
Pestivirus B	KT875169	VIPR_ALG4_10037030	2a	NS5A	91W	2005	-0.8721	0.5730	1.1137
Pestivirus B	KP057803	VIPR_ALG4_80287536	2a	NS5A	24515	1993	-0.7829	0.6134	1.1113
Pestivirus B	KR093034	VIPR_ALG4_92904888	2a	NS5A	NY-93	1993	-0.8053	0.6091	1.1098
Pestivirus B	MW006485	VIPR_ALG4_QPF4972	2b	NS5A	HEN01	2014	-0.6550	0.5506	1.1122
Pestivirus B	MH231142	VIPR_ALG4_AZQ0066	2c	NS5A	Parker	1991	-0.9693	0.6023	1.1128
Pestivirus B	MH806435	VIPR_ALG4_AZP5716	2a	NS5A	1336H	2005	-0.7605	0.5831	1.1066
Pestivirus B	MH231136	VIPR_ALG4_AZQ0066	2a	NS5A	JV14	1998	-0.7481	0.6016	1.1100
Pestivirus B	MK599227	VIPR_ALG4_QEU5262	2a	NS5A	SD-1	2016	-0.6497	0.5873	1.1071
Pestivirus B	HG426487	VIPR_ALG4_61932574	2c	NS5A	NRW 19-13-1_Dup(-)	2013	-0.8376	0.6117	1.1130
Pestivirus B	MH231138	VIPR_ALG4_AZQ0066	2a	NS5A	MnFetus	1991	-0.8013	0.6079	1.1115
Pestivirus B	MH231131	VIPR_ALG4_AZQ0065	2a	NS5A	AU501	2006	-1.0094	0.5884	1.1177
Pestivirus B	MH231151	VIPR_ALG4_AZQ0067	2e	NS5A	14622	2005	-0.9664	0.5691	1.1108
Pestivirus B	MH231152	VIPR_ALG4_AZQ0067	2e	NS5A	2412	1989	-0.8844	0.5771	1.1110
Pestivirus B	MH231149	VIPR_ALG4_AZQ0067	2e	NS5A	Short	1989	-0.9664	0.5691	1.1108
Pestivirus B	KJ000672	VIPR_ALG4_59423572	2b	NS5A	SD1301	2012	-0.6618	0.5525	1.1122
Pestivirus B	MH231148	VIPR_ALG4_AZQ0067	2e	NS5A	12-149150	2012	-0.8350	0.5955	1.1124
Pestivirus B	MH231150	VIPR_ALG4_AZQ0067	2e	NS5A	12-151955-317	2012	-0.8762	0.5848	1.1116
Pestivirus B	HG426495	VIPR_ALG4_61932576	2c	NS5A	VOE 4407	2007	-0.9119	0.5844	1.1153
Pestivirus B	HG426491	VIPR_ALG4_61932575	2c	NS5A	Potsdam 1600	2000	-0.9562	0.5942	1.1117
Pestivirus B	MH231141	VIPR_ALG4_AZQ0066	2a	NS5A	PI28	2016	-0.6831	0.5812	1.1155
Pestivirus B	HG426494	VIPR_ALG4_61932576	2c	NS5A	SH2210-23	2010	-0.8717	0.5910	1.1105
Pestivirus B	MH231137	VIPR_ALG4_AZQ0066	2a	NS5A	MadSpl	1991	-0.7707	0.6089	1.1104
Pestivirus B	KP941585	VIPR_ALG4_80092432	2a	NS5A	USMARC-55476	2014	-0.8485	0.6060	1.1116
Pestivirus B	KT832820	VIPR_ALG4_99822633	2a	NS5A	USMARC-60767	2014	-0.7802	0.6100	1.1126
Pestivirus B	MH231134	VIPR_ALG4_AZQ0065	2a	NS5A	B9497	1997	-0.7752	0.5945	1.1142
Pestivirus B	MN527354	VIPR_ALG4_QLH0204	2a	NS5A	GS2018	2018	-0.9668	0.5914	1.1129
Pestivirus B	MH231144	VIPR_ALG4_AZQ0066	2a	NS5A	Sanderson6319	1992	-0.7904	0.6038	1.1107
Pestivirus B	HG426493	VIPR_ALG4_61932576	2c	NS5A	SH2210-17	2010	-0.8833	0.5925	1.1111
Pestivirus B	KT832817	VIPR_ALG4_99822632	2a	NS5A	USMARC-60764	2014	-0.9395	0.5837	1.1118
Pestivirus B	KT832822	VIPR_ALG4_99822633	2a	NS5A	USMARC-60779	2014	-0.8058	0.6116	1.1104
Pestivirus B	MH231125	VIPR_ALG4_AZQ0064	2a	NS5A	2139	1992	-0.7320	0.6095	1.1118
Pestivirus B	KP941582	VIPR_ALG4_80092431	2c	NS5A	USMARC-53873	2014	-0.7702	0.6219	1.1107
Pestivirus B	KT832819	VIPR_ALG4_99822632	2a	NS5A	USMARC-60766	2014	-0.7394	0.6056	1.1080
Pestivirus B	KT832821	VIPR_ALG4_99822633	2c	NS5A	USMARC-60768	2014	-0.8357	0.6074	1.1146
Pestivirus B	HG426479	VIPR_ALG4_61932573	2c	NS5A	D37-13-2_Dup(-)	2013	-0.8376	0.6117	1.1130
Pestivirus B	HG426481	VIPR_ALG4_61932573	2c	NS5A	D75-13-609_Dup(-)	2013	-0.9117	0.6071	1.1159
Pestivirus B	MH231123	VIPR_ALG4_AZQ0064	2a	NS5A	10406	1993	-0.7879	0.6210	1.1111
Pestivirus B	HG426492	VIPR_ALG4_61932575	2c	NS5A	SH2210-14	2010	-0.8737	0.5944	1.1109
Pestivirus B	KT832823	VIPR_ALG4_99822633	2a	NS5A	USMARC-60780	2014	-0.8287	0.6019	1.1080
Pestivirus B	HG426485	VIPR_ALG4_61932574	2c	NS5A	NRW 14-13_Dup(-)	2013	-0.8378	0.6104	1.1150
Pestivirus B	MH231126	VIPR_ALG4_AZQ0065	2b	NS5A	3237	1990	-0.7387	0.5633	1.1081
Pestivirus B	KT832818	VIPR_ALG4_99822632	2a	NS5A	USMARC-60765	2014	-0.9859	0.5973	1.1018
Pestivirus B	HG426483	VIPR_ALG4_61932574	2c	NS5A	NRW 12-13_Dup(-)	2013	-0.8376	0.6117	1.1130
Pestivirus B	HG426489	VIPR_ALG4_61932575	2c	NS5A	NRW 19-13-8_Dup(-)	2013	-0.8376	0.6117	1.1130

Species according to VIPRBRC	GenBank Accession	GenBank Protein Accession	Subgenotype	Protein	Strain Name	Collection Year	SVM Patho. Score	Vaxijen Antig. Score	Averged score of EMBOSS motifs
Pestivirus B	MH231130	VIPR_ALG4_AZQ0065	2a	NS5A	95-1501	1998	-0.7588	0.6248	1.1113
Pestivirus B	MH231132	VIPR_ALG4_AZQ0065	2a	NS5A	AzSpl	1997	-0.7457	0.5817	1.1180
Pestivirus B	MH231128	VIPR_ALG4_AZQ0065	2a	NS5A	570152	1992	-0.7939	0.6007	1.1094
Pestivirus B	MH231135	VIPR_ALG4_AZQ0065	2a	NS5A	BV1907	1995	-0.8437	0.6163	1.1094
Pestivirus B	JF714967	VIPR_ALG4_34657797	2a	NS5A	HLJ-10	2011	-0.8614	0.5748	1.1094
Pestivirus B	MH231139	VIPR_ALG4_AZQ0066	2a	NS5A	Olwein #12	1990	-0.8935	0.6104	1.1150
Pestivirus B	MH231140	VIPR_ALG4_AZQ0066	2a	NS5A	PA	1992	-0.7794	0.6103	1.1107
Pestivirus B	MH231143	VIPR_ALG4_AZQ0066	2a	NS5A	RS886	2014	-0.7908	0.6161	1.1111
Pestivirus B	HQ258810	VIPR_ALG4_31199027	2a	NS5A	SH-28	2009	-0.7133	0.5546	1.1193
Pestivirus B	MH231145	VIPR_ALG4_AZQ0066	2a	NS5A	Victor301	1990	-0.7157	0.6076	1.1103
Pestivirus B	MH231146	VIPR_ALG4_AZQ0067	2a	NS5A	WiscA	1991	-0.7435	0.6037	1.1118
Pestivirus B	FJ527854	VIPR_ALG4_22961018	2a	NS5A	XJ-04	2004	-0.8772	0.5937	1.1142
Pestivirus B	KC963968	VIPR_ALG4_53029119	2a	NS5A	11F011	2011	-0.7865	0.6035	1.1115
Pestivirus B	MH806437	VIPR_ALG4_AZP5716	2a	NS5A	9231	2004	-0.8968	0.5936	1.1163
Pestivirus B	MG879027	VIPR_ALG4_AVA3071	2a	NS5A	CN10.2015.821	2014	-0.9010	0.5820	1.1162
Pestivirus B	KX096718	VIPR_ALG4_11130170	2a	NS5A	HB-1511	2015	-0.6858	0.5686	1.1149
Pestivirus B	LC649064	VIPR_ALG4_BDB0736	2c	NS5A	KZ-91-NCP	1991	-0.7958	0.6315	1.1148
Pestivirus B	MH231147	VIPR_ALG4_AZQ0067	2c	NS5A	PI12	2016	-0.9786	0.6120	1.1137
Pestivirus B	GQ888686	VIPR_ALG4_27071949	2a	NS5A	JZ05-1	2005	-0.9277	0.6232	1.1150
Pestivirus B	MW168422	VIPR_ALG4_QZM0693	2a	NS5A	YNJG2020	2020	-0.6714	0.6084	1.1107
Pestivirus B	HG426488	VIPR_ALG4_61932575	2c	NS5A	NRW 19-13-1_Dup(+)	2013	-0.8376	0.6117	1.1130
Pestivirus B	HG426480	VIPR_ALG4_61932573	2c	NS5A	D37-13-2_Dup(+)	2013	-0.8376	0.6117	1.1130
Pestivirus B	HG426482	VIPR_ALG4_61932573	2c	NS5A	D75-13-609_Dup(+)	2013	-0.9117	0.6071	1.1159
Pestivirus B	HG426486	VIPR_ALG4_61932574	2c	NS5A	NRW 14-13_Dup(+)	2013	-0.8378	0.6104	1.1150
Pestivirus B	HG426484	VIPR_ALG4_61932574	2c	NS5A	NRW 12-13_Dup(+)	2013	-0.8376	0.6117	1.1130
Pestivirus B	HG426490	VIPR_ALG4_61932575	2c	NS5A	NRW 19-13-8_Dup(+)	2013	-0.8376	0.6117	1.1130
Pestivirus B	MH231129	VIPR_ALG4_AZQ0065	2a	NS5A	5912c	1995	-0.8564	0.6085	1.1135
Pestivirus B	MH806438	VIPR_ALG4_AZP5716	2a	NS5A	McCart_c	1989	-0.7588	0.6248	1.1113
Pestivirus B	MH806434	VIPR_ALG4_AZP5716	2a	NS5A	125c	1990	-0.9712	0.6087	1.1158
Pestivirus B	MH231133	VIPR_ALG4_AZQ0065	2e	NS5A	B69519c	2006	-0.8844	0.5771	1.1110
Pestivirus B	MH806436	VIPR_ALG4_AZP5716	2a	NS5A	296c	1995	-0.9171	0.6159	1.1155
Pestivirus B	MH231124	VIPR_ALG4_AZQ0064	2e	NS5A	1786c	1989	-0.8844	0.5771	1.1110
Pestivirus B	MH231127	VIPR_ALG4_AZQ0065	2a	NS5A	53637c	2004	-0.8553	0.6097	1.1103
Pestivirus B	MH231133	VIPR_ALG4_AZQ0065	2e	NS5B	B69519c	2006	-0.6908	0.4448	1.1120
Pestivirus B	MH231124	VIPR_ALG4_AZQ0064	2e	NS5B	1786c	1989	-0.6908	0.4448	1.1120
Pestivirus B	KX170653	APT71176.1	2a	NS5B	V028	2006	-0.5966	0.4507	1.1130
Pestivirus B	KX170654	APT71177.1	2a	NS5B	V076	2011	-0.5863	0.4771	1.1129
Pestivirus B	KX170655	APT71178.1	2a	NS5B	V085	2007	-0.6459	0.4664	1.1154
Pestivirus B	KX170651	APT71174.1	2a	NS5B	V086	2006	-0.6503	0.4587	1.1170
Pestivirus B	KX170652	APT71175.1	2a	NS5B	V089	2004	-0.6818	0.4669	1.1138
Pestivirus B	KX170650	APT71173.1	2a	NS5B	V095	1999	-0.6499	0.4606	1.1129
Pestivirus B	KX170649	APT71172.1	2a	NS5B	V097	1998	-0.5973	0.4656	1.1184
Pestivirus B	KX170648	APT71171.1	2a	NS5B	V065	2006	-0.5373	0.4577	1.1169
Pestivirus B	HG426488	VIPR_ALG4_61932575	2c	NS5B	NRW 19-13-1_Dup(+)	2013	-0.6701	0.4725	1.1146
Pestivirus B	HG426480	VIPR_ALG4_61932573	2c	NS5B	D37-13-2_Dup(+)	2013	-0.6360	0.4780	1.1146
Pestivirus B	HG426482	VIPR_ALG4_61932573	2c	NS5B	D75-13-609_Dup(+)	2013	-0.6590	0.4725	1.1146
Pestivirus B	HG426486	VIPR_ALG4_61932574	2c	NS5B	NRW 14-13_Dup(+)	2013	-0.6723	0.4715	1.1146

Species according to VIPRBRC	GenBank Accession	GenBank Protein Accession	Subgenotype	Protein	Strain Name	Collection Year	SVM Patho. Score	Vaxijen Antig. Score	Averged score of EMBOSS motifs
Pestivirus B	HG426484	VIPR_ALG4_61932574	2c	NS5B	NRW 12-13_Dup(+)	2013	-0.6701	0.4725	1.1146
Pestivirus B	HG426490	VIPR_ALG4_61932575	2c	NS5B	NRW 19-13-8_Dup(+)	2013	-0.6701	0.4725	1.1146
Pestivirus B	MH231129	VIPR_ALG4_AZQ0065	2a	NS5B	5912c	1995	-0.4581	0.4710	1.1156
Pestivirus B	MH806438	VIPR_ALG4_AZP5716	2a	NS5B	McCart_c	1989	-0.4698	0.4709	1.1176
Pestivirus B	MH806434	VIPR_ALG4_AZP5716	2a	NS5B	125c	1990	-0.5195	0.4537	1.1143
Pestivirus B	MH806436	VIPR_ALG4_AZP5716	2a	NS5B	296c	1995	-0.4850	0.4633	1.1116
Pestivirus B	MH231127	VIPR_ALG4_AZQ0065	2a	NS5B	53637c	2004	-0.5654	0.4641	1.1158
Pestivirus B	KX838370	VIPR_ALG4_AOS5274	2a	NS5B	Ind141353	2007	-0.4526	0.4645	1.1132
Pestivirus B	KT875139	VIPR_ALG4_10037029	2a	NS5B	32W	2005	-0.6113	0.4727	1.1166
Pestivirus B	MN824468	VIPR_ALG4_QJF1227	2a	NS5B	CPAE_contamination/2018	2018	-0.4852	0.4712	1.1174
Pestivirus B	KT875134	VIPR_ALG4_10037029	2a	NS5B	12W	2005	-0.6055	0.4744	1.1166
Pestivirus B	KT875135	VIPR_ALG4_10037029	2a	NS5B	13Y	2005	-0.6231	0.4690	1.1131
Pestivirus B	KT875136	VIPR_ALG4_10037029	2a	NS5B	27Y	2005	-0.6141	0.4709	1.1192
Pestivirus B	KT875137	VIPR_ALG4_10037029	2a	NS5B	29Y	2005	-0.6211	0.4694	1.1155
Pestivirus B	KT875138	VIPR_ALG4_10037029	2a	NS5B	2Y	2005	-0.6113	0.4727	1.1166
Pestivirus B	KT875140	VIPR_ALG4_10037029	2a	NS5B	34Y	2005	-0.6113	0.4727	1.1166
Pestivirus B	KT875141	VIPR_ALG4_10037029	2a	NS5B	36W	2005	-0.6231	0.4690	1.1131
Pestivirus B	KT875142	VIPR_ALG4_10037029	2a	NS5B	41Y	2005	-0.6113	0.4727	1.1166
Pestivirus B	KT875143	VIPR_ALG4_10037029	2a	NS5B	42W	2005	-0.6113	0.4727	1.1166
Pestivirus B	KT875144	VIPR_ALG4_10037029	2a	NS5B	43Y	2005	-0.6231	0.4690	1.1131
Pestivirus B	KT875145	VIPR_ALG4_10037029	2a	NS5B	47Y	2005	-0.6080	0.4721	1.1177
Pestivirus B	KT875146	VIPR_ALG4_10037029	2a	NS5B	50Y	2005	-0.6113	0.4727	1.1166
Pestivirus B	KT875147	VIPR_ALG4_10037029	2a	NS5B	51W	2005	-0.6113	0.4727	1.1166
Pestivirus B	KT875148	VIPR_ALG4_10037029	2a	NS5B	51Y	2005	-0.6113	0.4727	1.1166
Pestivirus B	KT875149	VIPR_ALG4_10037029	2a	NS5B	53W	2005	-0.6073	0.4720	1.1183
Pestivirus B	KT875150	VIPR_ALG4_10037029	2a	NS5B	58W	2005	-0.6101	0.4721	1.1192
Pestivirus B	KT875151	VIPR_ALG4_10037029	2a	NS5B	58Y	2005	-0.6112	0.4753	1.1192
Pestivirus B	KT875152	VIPR_ALG4_10037029	2a	NS5B	5Y	2005	-0.6113	0.4727	1.1166
Pestivirus B	KT875153	VIPR_ALG4_10037029	2a	NS5B	62Y	2005	-0.5952	0.4827	1.1157
Pestivirus B	KT875154	VIPR_ALG4_10037029	2a	NS5B	65Y	2005	-0.6113	0.4727	1.1166
Pestivirus B	KT875155	VIPR_ALG4_10037029	2a	NS5B	67Y	2005	-0.6113	0.4727	1.1166
Pestivirus B	KT875156	VIPR_ALG4_10037029	2a	NS5B	68W	2005	-0.6231	0.4690	1.1131
Pestivirus B	KT875157	VIPR_ALG4_10037029	2a	NS5B	71Y	2005	-0.6113	0.4727	1.1166
Pestivirus B	KT875158	VIPR_ALG4_10037029	2a	NS5B	73Y	2005	-0.6113	0.4727	1.1166
Pestivirus B	KT875159	VIPR_ALG4_10037029	2a	NS5B	74Y	2005	-0.6113	0.4727	1.1166
Pestivirus B	KT875160	VIPR_ALG4_10037029	2a	NS5B	75W	2005	-0.6113	0.4727	1.1166
Pestivirus B	KT875161	VIPR_ALG4_10037029	2a	NS5B	75Y	2005	-0.6113	0.4727	1.1166
Pestivirus B	KT875162	VIPR_ALG4_10037029	2a	NS5B	76Y	2005	-0.6113	0.4727	1.1166
Pestivirus B	KT875163	VIPR_ALG4_10037029	2a	NS5B	78W	2005	-0.6113	0.4727	1.1166
Pestivirus B	KT875164	VIPR_ALG4_10037029	2a	NS5B	79W	2005	-0.6231	0.4690	1.1131
Pestivirus B	KT875165	VIPR_ALG4_10037029	2a	NS5B	7W	2005	-0.6141	0.4709	1.1192
Pestivirus B	KT875166	VIPR_ALG4_10037029	2a	NS5B	82W	2005	-0.6369	0.4739	1.1166
Pestivirus B	KT875167	VIPR_ALG4_10037029	2a	NS5B	83Y	2005	-0.6113	0.4727	1.1166
Pestivirus B	KT875168	VIPR_ALG4_10037029	2a	NS5B	90W	2005	-0.6231	0.4690	1.1131
Pestivirus B	KT875169	VIPR_ALG4_10037030	2a	NS5B	91W	2005	-0.6080	0.4721	1.1177
Pestivirus B	KP057803	VIPR_ALG4_80287536	2a	NS5B	24515	1993	-0.5318	0.4680	1.1158
Pestivirus B	KR093034	VIPR_ALG4_92904888	2a	NS5B	NY-93	1993	-0.5522	0.4592	1.1195

Species according to VIPRBRC	GenBank Accession	GenBank Protein Accession	Subgenotype	Protein	Strain Name	Collection Year	SVM Patho. Score	Vaxijen Antig. Score	Averged score of EMBOSS motifs
Pestivirus B	MW006485	VIPR_ALG4_QPF4972	2b	NS5B	HEN01	2014	-0.6440	0.4501	1.1183
Pestivirus B	MH231142	VIPR_ALG4_AZQ0066	2c	NS5B	Parker	1991	-0.6291	0.4726	1.1203
Pestivirus B	MH806435	VIPR_ALG4_AZP5716	2a	NS5B	1336H	2005	-0.6431	0.4486	1.1161
Pestivirus B	MH231136	VIPR_ALG4_AZQ0066	2a	NS5B	JV14	1998	-0.6063	0.4673	1.1159
Pestivirus B	MK599227	VIPR_ALG4_QEU5262	2a	NS5B	SD-1	2016	-0.6050	0.4529	1.1182
Pestivirus B	HG426487	VIPR_ALG4_61932574	2c	NS5B	NRW 19-13-1_Dup(-)	2013	-0.6701	0.4725	1.1146
Pestivirus B	MH231138	VIPR_ALG4_AZQ0066	2a	NS5B	MnFetus	1991	-0.6584	0.4550	1.1209
Pestivirus B	MH231131	VIPR_ALG4_AZQ0065	2a	NS5B	AU501	2006	-0.6567	0.4640	1.1159
Pestivirus B	MH231151	VIPR_ALG4_AZQ0067	2e	NS5B	14622	2005	-0.5097	0.4680	1.1092
Pestivirus B	MH231152	VIPR_ALG4_AZQ0067	2e	NS5B	2412	1989	-0.6938	0.4573	1.1124
Pestivirus B	MH231149	VIPR_ALG4_AZQ0067	2e	NS5B	Short	1989	-0.5462	0.4647	1.1097
Pestivirus B	KJ000672	VIPR_ALG4_59423572	2b	NS5B	SD1301	2012	-0.6325	0.4510	1.1183
Pestivirus B	MH231148	VIPR_ALG4_AZQ0067	2e	NS5B	12-149150	2012	-0.7060	0.4444	1.1131
Pestivirus B	MH231150	VIPR_ALG4_AZQ0067	2e	NS5B	12-151955-317	2012	-0.7136	0.4465	1.1131
Pestivirus B	HG426495	VIPR_ALG4_61932576	2c	NS5B	VOE 4407	2007	-0.5651	0.4731	1.1180
Pestivirus B	HG426491	VIPR_ALG4_61932575	2c	NS5B	Potsdam 1600	2000	-0.6029	0.4723	1.1201
Pestivirus B	MH231141	VIPR_ALG4_AZQ0066	2a	NS5B	PI28	2016	-0.5446	0.4593	1.1201
Pestivirus B	HG426494	VIPR_ALG4_61932576	2c	NS5B	SH2210-23	2010	-0.6217	0.4776	1.1213
Pestivirus B	MH231137	VIPR_ALG4_AZQ0066	2a	NS5B	MadSpl	1991	-0.5688	0.4641	1.1191
Pestivirus B	KP941585	VIPR_ALG4_80092432	2a	NS5B	USMARC-55476	2014	-0.5479	0.4426	1.1210
Pestivirus B	KT832820	VIPR_ALG4_99822633	2a	NS5B	USMARC-60767	2014	-0.5805	0.4653	1.1205
Pestivirus B	MH231134	VIPR_ALG4_AZQ0065	2a	NS5B	B9497	1997	-0.7000	0.4484	1.1096
Pestivirus B	MN527354	VIPR_ALG4_QLH0204	2a	NS5B	GS2018	2018	-0.6141	0.4595	1.1105
Pestivirus B	MH231144	VIPR_ALG4_AZQ0066	2a	NS5B	Sanderson6319	1992	-0.6902	0.4527	1.1196
Pestivirus B	HG426493	VIPR_ALG4_61932576	2c	NS5B	SH2210-17	2010	-0.6082	0.4842	1.1183
Pestivirus B	KT832817	VIPR_ALG4_99822632	2a	NS5B	USMARC-60764	2014	-0.6618	0.4642	1.1128
Pestivirus B	KT832822	VIPR_ALG4_99822633	2a	NS5B	USMARC-60779	2014	-0.5704	0.4697	1.1185
Pestivirus B	MH231125	VIPR_ALG4_AZQ0064	2a	NS5B	2139	1992	-0.5856	0.4614	1.1193
Pestivirus B	KP941582	VIPR_ALG4_80092431	2c	NS5B	USMARC-53873	2014	-0.5548	0.4764	1.1188
Pestivirus B	KT832819	VIPR_ALG4_99822632	2a	NS5B	USMARC-60766	2014	-0.5535	0.4622	1.1152
Pestivirus B	KT832821	VIPR_ALG4_99822633	2c	NS5B	USMARC-60768	2014	-0.6337	0.4660	1.1156
Pestivirus B	HG426479	VIPR_ALG4_61932573	2c	NS5B	D37-13-2_Dup(-)	2013	-0.6360	0.4780	1.1146
Pestivirus B	HG426481	VIPR_ALG4_61932573	2c	NS5B	D75-13-609_Dup(-)	2013	-0.6590	0.4725	1.1146
Pestivirus B	MH231123	VIPR_ALG4_AZQ0064	2a	NS5B	10406	1993	-0.6683	0.4703	1.1201
Pestivirus B	HG426492	VIPR_ALG4_61932575	2c	NS5B	SH2210-14	2010	-0.6082	0.4842	1.1183
Pestivirus B	KT832823	VIPR_ALG4_99822633	2a	NS5B	USMARC-60780	2014	-0.4743	0.4487	1.1197
Pestivirus B	HG426485	VIPR_ALG4_61932574	2c	NS5B	NRW 14-13_Dup(-)	2013	-0.6723	0.4715	1.1146
Pestivirus B	MH231126	VIPR_ALG4_AZQ0065	2b	NS5B	3237	1990	-0.5722	0.4527	1.1167
Pestivirus B	KT832818	VIPR_ALG4_99822632	2a	NS5B	USMARC-60765	2014	-0.6875	0.4676	1.1153
Pestivirus B	HG426483	VIPR_ALG4_61932574	2c	NS5B	NRW 12-13_Dup(-)	2013	-0.6701	0.4725	1.1146
Pestivirus B	HG426489	VIPR_ALG4_61932575	2c	NS5B	NRW 19-13-8_Dup(-)	2013	-0.6701	0.4725	1.1146
Pestivirus B	MH231130	VIPR_ALG4_AZQ0065	2a	NS5B	95-1501	1998	-0.4442	0.4706	1.1185
Pestivirus B	MH231132	VIPR_ALG4_AZQ0065	2a	NS5B	AzSpl	1997	-0.7046	0.4702	1.1190
Pestivirus B	MH231128	VIPR_ALG4_AZQ0065	2a	NS5B	570152	1992	-0.5922	0.4575	1.1196
Pestivirus B	MH231135	VIPR_ALG4_AZQ0065	2a	NS5B	BV1907	1995	-0.5270	0.4696	1.1169
Pestivirus B	JF714967	VIPR_ALG4_34657797	2a	NS5B	HLJ-10	2011	-0.5917	0.4596	1.1132
Pestivirus B	MH231139	VIPR_ALG4_AZQ0066	2a	NS5B	Olwein #12	1990	-0.4912	0.4749	1.1115

Species according to VIPRBRC	GenBank Accession	GenBank Protein Accession	Subgenotype	Protein	Strain Name	Collection Year	SVM Patho. Score	Vaxijen Antig. Score	Averged score of EMBOSS motifs
Pestivirus B	MH231140	VIPR_ALG4_AZQ0066	2a	NS5B	PA	1992	-0.6165	0.4568	1.1152
Pestivirus B	MH231143	VIPR_ALG4_AZQ0066	2a	NS5B	RS886	2014	-0.6468	0.4635	1.1170
Pestivirus B	MH231145	VIPR_ALG4_AZQ0066	2a	NS5B	Victor301	1990	-0.6231	0.4580	1.1180
Pestivirus B	MH231146	VIPR_ALG4_AZQ0067	2a	NS5B	WiscA	1991	-0.5700	0.4631	1.1195
Pestivirus B	FJ527854	VIPR_ALG4_22961018	2a	NS5B	XJ-04	2004	-0.6317	0.4618	1.1129
Pestivirus B	KC963968	VIPR_ALG4_53029119	2a	NS5B	11F011	2011	-0.6783	0.4645	1.1184
Pestivirus B	MH806437	VIPR_ALG4_AZP5716	2a	NS5B	9231	2004	-0.5751	0.4735	1.1144
Pestivirus B	MG879027	VIPR_ALG4_AVA3071	2a	NS5B	CN10.2015.821	2014	-0.6094	0.4743	1.1166
Pestivirus B	KX096718	VIPR_ALG4_11130170	2a	NS5B	HB-1511	2015	-0.6477	0.4623	1.1143
Pestivirus B	LC649064	VIPR_ALG4_BDB0736	2c	NS5B	KZ-91-NCP	1991	-0.6315	0.4652	1.1201
Pestivirus B	MH231147	VIPR_ALG4_AZQ0067	2c	NS5B	PI12	2016	-0.6972	0.4541	1.1207
Pestivirus B	GQ888686	VIPR_ALG4_27071949	2a	NS5B	JZ05-1	2005	-0.4939	0.4672	1.1108
Pestivirus B	MW168422	VIPR_ALG4_QZM0693	2a	NS5B	YNJG2020	2020	-0.5729	0.4595	1.1157
Pestivirus B	HQ258810	VIPR_ALG4_31199027	2a	NS5B	SH-28	2009	-0.6796	0.4498	1.1105
Pestivirus B	MW168422	VIPR_ALG4_QZM0693	2a	C	YNJG2020	2020	-0.5364	0.5723	1.1700
Pestivirus B	KX169985	VIPR_ALG4_11298792	2a	C	V002	1999	-0.5429	0.5623	1.1830
Pestivirus B	KX169984	VIPR_ALG4_11298792	2a	C	V005	1999	-0.5429	0.5623	1.1830
Pestivirus B	KX169968	VIPR_ALG4_11298792	2a	C	V017	2003	-0.1923	0.5576	1.1830
Pestivirus B	KX169967	VIPR_ALG4_11298792	2a	C	V019	2004	-0.1923	0.5576	1.1830
Pestivirus B	KX169982	VIPR_ALG4_11298792	2a	C	V021	2005	-0.5429	0.5623	1.1830
Pestivirus B	KX169975	VIPR_ALG4_11298792	2a	C	V023	2006	-0.6383	0.5709	1.1830
Pestivirus B	KX169976	VIPR_ALG4_11298792	2a	C	V024	2006	-0.5429	0.5623	1.1830
Pestivirus B	KX169979	VIPR_ALG4_11298792	2a	C	V025	2006	-0.8965	0.5587	1.1830
Pestivirus B	KX169961	VIPR_ALG4_11298791	2a	C	V028	2006	-0.2804	0.5254	1.1463
Pestivirus B	KX169981	VIPR_ALG4_11298792	2a	C	V037	2008	-0.2922	0.5922	1.1830
Pestivirus B	KX169978	VIPR_ALG4_11298792	2a	C	V044	2008	-0.7586	0.5623	1.1725
Pestivirus B	KX169974	VIPR_ALG4_11298792	2a	C	V047	2009	-0.1923	0.5576	1.1830
Pestivirus B	KX169977	VIPR_ALG4_11298792	2a	C	V051	2010	-0.2736	0.5296	1.1830
Pestivirus B	KX169969	VIPR_ALG4_11298792	2a	C	V062	2005	-0.1923	0.5576	1.1830
Pestivirus B	KX169970	VIPR_ALG4_11298792	2a	C	V063	2005	-0.1923	0.5576	1.1830
Pestivirus B	KX169972	VIPR_ALG4_11298792	2a	C	V076	2011	-0.1923	0.5576	1.1830
Pestivirus B	KX169983	VIPR_ALG4_11298792	2a	C	V079	2010	-0.5429	0.5623	1.1830
Pestivirus B	KX169966	VIPR_ALG4_11298791	2a	C	V081	2009	-0.1923	0.5576	1.1830
Pestivirus B	KX169971	VIPR_ALG4_11298792	2a	C	V082	2008	-0.1923	0.5576	1.1830
Pestivirus B	KX169973	VIPR_ALG4_11298792	2a	C	V085	2007	-0.3077	0.5865	1.1393
Pestivirus B	KX169962	VIPR_ALG4_11298791	2a	C	V086	2006	-0.5426	0.5266	1.2055
Pestivirus B	KX169964	VIPR_ALG4_11298791	2a	C	V088	2005	-0.3051	0.5665	1.1830
Pestivirus B	KX169963	VIPR_ALG4_11298791	2a	C	V089	2004	-0.5426	0.5266	1.2055
Pestivirus B	KX169965	VIPR_ALG4_11298791	2a	C	V095	1999	-0.3822	0.5800	1.1830
Pestivirus B	KX169980	VIPR_ALG4_11298792	2a	C	V097	1998	-0.2250	0.5583	1.1830
Pestivirus B	MT024568	VIPR_ALG4_QPK4118	2a	C	560615-F0-22	2015	-0.2736	0.5296	1.1830
Pestivirus B	KT875134	VIPR_ALG4_10037029	2a	C	12W	2005	-0.2107	0.5311	1.1770
Pestivirus B	KT875135	VIPR_ALG4_10037029	2a	C	13Y	2005	-0.2107	0.5311	1.1770
Pestivirus B	KP057803	VIPR_ALG4_80287536	2a	C	24515	1993	-0.5429	0.5623	1.1830
Pestivirus B	KT875136	VIPR_ALG4_10037029	2a	C	27Y	2005	-0.2107	0.5311	1.1770
Pestivirus B	KT875137	VIPR_ALG4_10037029	2a	C	29Y	2005	-0.2107	0.5311	1.1770
Pestivirus B	KT875138	VIPR_ALG4_10037029	2a	C	2Y	2005	-0.2107	0.5311	1.1770

Species according to VIPRBRC	GenBank Accession	GenBank Protein Accession	Subgenotype	Protein	Strain Name	Collection Year	SVM Patho. Score	Vaxijen Antig. Score	Averged score of EMBOSS motifs
Pestivirus B	KT875139	VIPR_ALG4_10037029	2a	C	32W	2005	-0.2107	0.5311	1.1770
Pestivirus B	KT875140	VIPR_ALG4_10037029	2a	C	34Y	2005	-0.2107	0.5311	1.1770
Pestivirus B	KT875141	VIPR_ALG4_10037029	2a	C	36W	2005	-0.2107	0.5311	1.1770
Pestivirus B	KT875142	VIPR_ALG4_10037029	2a	C	41Y	2005	-0.2107	0.5311	1.1770
Pestivirus B	KT875143	VIPR_ALG4_10037029	2a	C	42W	2005	-0.2107	0.5311	1.1770
Pestivirus B	KT875144	VIPR_ALG4_10037029	2a	C	43Y	2005	-0.2107	0.5311	1.1770
Pestivirus B	KT875145	VIPR_ALG4_10037029	2a	C	47Y	2005	-0.2107	0.5311	1.1770
Pestivirus B	KT875146	VIPR_ALG4_10037029	2a	C	50Y	2005	-0.2107	0.5311	1.1770
Pestivirus B	KT875147	VIPR_ALG4_10037029	2a	C	51W	2005	-0.2107	0.5311	1.1770
Pestivirus B	KT875148	VIPR_ALG4_10037029	2a	C	51Y	2005	-0.2107	0.5311	1.1770
Pestivirus B	KT875149	VIPR_ALG4_10037029	2a	C	53W	2005	-0.2107	0.5311	1.1770
Pestivirus B	KT875150	VIPR_ALG4_10037029	2a	C	58W	2005	-0.2107	0.5311	1.1770
Pestivirus B	KT875151	VIPR_ALG4_10037029	2a	C	58Y	2005	-0.2107	0.5311	1.1770
Pestivirus B	KT875152	VIPR_ALG4_10037029	2a	C	5Y	2005	-0.3162	0.5574	1.1700
Pestivirus B	KT875153	VIPR_ALG4_10037029	2a	C	62Y	2005	-0.2824	0.5379	1.1770
Pestivirus B	KT875154	VIPR_ALG4_10037029	2a	C	65Y	2005	-0.2107	0.5311	1.1770
Pestivirus B	KT875155	VIPR_ALG4_10037029	2a	C	67Y	2005	-0.2107	0.5311	1.1770
Pestivirus B	KT875156	VIPR_ALG4_10037029	2a	C	68W	2005	-0.2107	0.5311	1.1770
Pestivirus B	KT875157	VIPR_ALG4_10037029	2a	C	71Y	2005	-0.2107	0.5311	1.1770
Pestivirus B	KT875158	VIPR_ALG4_10037029	2a	C	73Y	2005	-0.4302	0.5558	1.1710
Pestivirus B	KT875159	VIPR_ALG4_10037029	2a	C	74Y	2005	-0.2107	0.5311	1.1770
Pestivirus B	KT875160	VIPR_ALG4_10037029	2a	C	75W	2005	-0.2107	0.5311	1.1770
Pestivirus B	KT875161	VIPR_ALG4_10037029	2a	C	75Y	2005	-0.2107	0.5311	1.1770
Pestivirus B	KT875162	VIPR_ALG4_10037029	2a	C	76Y	2005	-0.2107	0.5311	1.1770
Pestivirus B	KT875163	VIPR_ALG4_10037029	2a	C	78W	2005	-0.2107	0.5311	1.1770
Pestivirus B	KT875164	VIPR_ALG4_10037029	2a	C	79W	2005	-0.2107	0.5311	1.1770
Pestivirus B	KT875165	VIPR_ALG4_10037029	2a	C	7W	2005	-0.2107	0.5311	1.1770
Pestivirus B	KT875166	VIPR_ALG4_10037029	2a	C	82W	2005	-0.2107	0.5311	1.1770
Pestivirus B	KT875167	VIPR_ALG4_10037029	2a	C	83Y	2005	-0.2107	0.5311	1.1770
Pestivirus B	KT875168	VIPR_ALG4_10037029	2a	C	90W	2005	-0.2107	0.5311	1.1770
Pestivirus B	KT875169	VIPR_ALG4_10037030	2a	C	91W	2005	-0.2107	0.5311	1.1770
Pestivirus B	MN824468	VIPR_ALG4_QJF1227	2a	C	CPAE_contamination/2018	2018	0.0430	0.5693	1.1700
Pestivirus B	KR093034	VIPR_ALG4_92904888	2a	C	NY-93	1993	-0.5429	0.5623	1.1830
Pestivirus B	EU747875	VIPR_ALG4_19033620	2b	C	17237	2004	-0.5135	0.5099	1.1565
Pestivirus B	MW006485	VIPR_ALG4_QPF4972	2b	C	HEN01	2014	-0.7808	0.5752	1.1830
Pestivirus B	MH231142	VIPR_ALG4_AZQ0066	2c	C	Parker	1991	-0.3488	0.5892	1.2055
Pestivirus B	MH806435	VIPR_ALG4_AZP5716	2a	C	1336H	2005	-0.4032	0.5882	1.1360
Pestivirus B	MH231136	VIPR_ALG4_AZQ0066	2a	C	JV14	1998	-0.8965	0.5587	1.1830
Pestivirus B	MK599227	VIPR_ALG4_QEU5262	2a	C	SD-1	2016	-0.5325	0.5690	1.1830
Pestivirus B	HG426488	VIPR_ALG4_61932575	2c	C	NRW 19-13-1_Dup(+)	2013	0.1527	0.5957	1.2055
Pestivirus B	HG426487	VIPR_ALG4_61932574	2c	C	NRW 19-13-1_Dup(-)	2013	0.1527	0.5957	1.2055
Pestivirus B	MH231138	VIPR_ALG4_AZQ0066	2a	C	MnFetus	1991	-0.5429	0.5623	1.1830
Pestivirus B	MH231131	VIPR_ALG4_AZQ0065	2a	C	AU501	2006	-0.0227	0.5506	1.1925
Pestivirus B	MH806434	VIPR_ALG4_AZP5716	2a	C	125c	1990	-0.4119	0.5620	1.1830
Pestivirus B	MH231151	VIPR_ALG4_AZQ0067	2e	C	14622	2005	0.3019	0.5315	1.1925
Pestivirus B	MH231152	VIPR_ALG4_AZQ0067	2e	C	2412	1989	-0.1700	0.4952	1.1925
Pestivirus B	MH231127	VIPR_ALG4_AZQ0065	2a	C	53637c	2004	-0.2736	0.5296	1.1830

Species according to VIPRBRC	GenBank Accession	GenBank Protein Accession	Subgenotype	Protein	Strain Name	Collection Year	SVM Patho. Score	Vaxijen Antig. Score	Averged score of EMBOSS motifs
Pestivirus B	MH231149	VIPR_ALG4_AZQ0067	2e	C	Short	1989	0.3019	0.5315	1.1925
Pestivirus B	MH231129	VIPR_ALG4_AZQ0065	2a	C	5912c	1995	-0.7438	0.5660	1.1830
Pestivirus B	KJ000672	VIPR_ALG4_59423572	2b	C	SD1301	2012	-0.7808	0.5752	1.1830
Pestivirus B	MH231148	VIPR_ALG4_AZQ0067	2e	C	12-149150	2012	-0.0823	0.4816	1.1925
Pestivirus B	MH231150	VIPR_ALG4_AZQ0067	2e	C	12-151955-317	2012	-0.2612	0.4948	1.1925
Pestivirus B	MH231133	VIPR_ALG4_AZQ0065	2e	C	B69519c	2006	-0.1700	0.4952	1.1925
Pestivirus B	HG426495	VIPR_ALG4_61932576	2c	C	VOE 4407	2007	-0.6033	0.5773	1.2055
Pestivirus B	HG426480	VIPR_ALG4_61932573	2c	C	D37-13-2_Dup(+)	2013	0.1527	0.5957	1.2055
Pestivirus B	HG426482	VIPR_ALG4_61932573	2c	C	D75-13-609_Dup(+)	2013	0.1527	0.5957	1.2055
Pestivirus B	HG426486	VIPR_ALG4_61932574	2c	C	NRW 14-13_Dup(+)	2013	0.1527	0.5957	1.2055
Pestivirus B	HG426491	VIPR_ALG4_61932575	2c	C	Potsdam 1600	2000	-0.1014	0.6089	1.2055
Pestivirus B	MH231141	VIPR_ALG4_AZQ0066	2a	C	PI28	2016	-0.4624	0.5459	1.1830
Pestivirus B	HG426494	VIPR_ALG4_61932576	2c	C	SH2210-23	2010	-0.3488	0.5892	1.2055
Pestivirus B	MH806436	VIPR_ALG4_AZP5716	2a	C	296c	1995	-0.6021	0.5609	1.1990
Pestivirus B	MH231137	VIPR_ALG4_AZQ0066	2a	C	MadSpl	1991	-0.2250	0.5583	1.1830
Pestivirus B	KP941585	VIPR_ALG4_80092432	2a	C	USMARC-55476	2014	-0.9738	0.5350	1.1373
Pestivirus B	KT832820	VIPR_ALG4_99822633	2a	C	USMARC-60767	2014	-0.2250	0.5583	1.1830
Pestivirus B	MH231134	VIPR_ALG4_AZQ0065	2a	C	B9497	1997	-0.5383	0.5444	1.1925
Pestivirus B	MN527354	VIPR_ALG4_QLH0204	2a	C	GS2018	2018	-0.3665	0.5490	1.1715
Pestivirus B	MH231144	VIPR_ALG4_AZQ0066	2a	C	Sanderson6319	1992	-0.5429	0.5623	1.1830
Pestivirus B	HG426493	VIPR_ALG4_61932576	2c	C	SH2210-17	2010	-0.6033	0.5773	1.2055
Pestivirus B	KT832817	VIPR_ALG4_99822632	2a	C	USMARC-60764	2014	-0.1543	0.5625	1.1715
Pestivirus B	KT832822	VIPR_ALG4_99822633	2a	C	USMARC-60779	2014	-0.2250	0.5583	1.1830
Pestivirus B	MH231125	VIPR_ALG4_AZQ0064	2a	C	2139	1992	-0.2250	0.5583	1.1830
Pestivirus B	KP941582	VIPR_ALG4_80092431	2c	C	USMARC-53873	2014	-0.4221	0.5656	1.1830
Pestivirus B	KT832819	VIPR_ALG4_99822632	2a	C	USMARC-60766	2014	-0.5429	0.5623	1.1830
Pestivirus B	KT832821	VIPR_ALG4_99822633	2c	C	USMARC-60768	2014	-0.5675	0.5962	1.2055
Pestivirus B	HG426479	VIPR_ALG4_61932573	2c	C	D37-13-2_Dup(-)	2013	0.1527	0.5957	1.2055
Pestivirus B	HG426481	VIPR_ALG4_61932573	2c	C	D75-13-609_Dup(-)	2013	0.1527	0.5957	1.2055
Pestivirus B	MH231123	VIPR_ALG4_AZQ0064	2a	C	10406	1993	-0.3053	0.5674	1.1830
Pestivirus B	HG426492	VIPR_ALG4_61932575	2c	C	SH2210-14	2010	-0.6033	0.5773	1.2055
Pestivirus B	KT832823	VIPR_ALG4_99822633	2a	C	USMARC-60780	2014	-0.6791	0.5649	1.1830
Pestivirus B	HG426485	VIPR_ALG4_61932574	2c	C	NRW 14-13_Dup(-)	2013	0.1527	0.5957	1.2055
Pestivirus B	MH231126	VIPR_ALG4_AZQ0065	2b	C	3237	1990	-0.4731	0.5819	1.1830
Pestivirus B	MH806438	VIPR_ALG4_AZP5716	2a	C	McCart_c	1989	-0.4737	0.5269	1.1830
Pestivirus B	KT832818	VIPR_ALG4_99822632	2a	C	USMARC-60765	2014	-0.0426	0.5638	1.1830
Pestivirus B	MH231124	VIPR_ALG4_AZQ0064	2e	C	1786c	1989	-0.1700	0.4952	1.1925
Pestivirus B	HG426483	VIPR_ALG4_61932574	2c	C	NRW 12-13_Dup(-)	2013	0.1527	0.5957	1.2055
Pestivirus B	HG426484	VIPR_ALG4_61932574	2c	C	NRW 12-13_Dup(+)	2013	0.1527	0.5957	1.2055
Pestivirus B	HG426489	VIPR_ALG4_61932575	2c	C	NRW 19-13-8_Dup(-)	2013	0.1527	0.5957	1.2055
Pestivirus B	HG426490	VIPR_ALG4_61932575	2c	C	NRW 19-13-8_Dup(+)	2013	0.1527	0.5957	1.2055
Pestivirus B	MH231130	VIPR_ALG4_AZQ0065	2a	C	95-1501	1998	-0.4136	0.5414	1.1830
Pestivirus B	MH231132	VIPR_ALG4_AZQ0065	2a	C	AzSpl	1997	-0.1475	0.4804	1.1830
Pestivirus B	MH231128	VIPR_ALG4_AZQ0065	2a	C	570152	1992	-0.4737	0.5269	1.1830
Pestivirus B	MH231135	VIPR_ALG4_AZQ0065	2a	C	BV1907	1995	-0.5429	0.5623	1.1830
Pestivirus B	JF714967	VIPR_ALG4_34657797	2a	C	HLJ-10	2011	-0.3399	0.5520	1.1490
Pestivirus B	MH231139	VIPR_ALG4_AZQ0066	2a	C	Olwein #12	1990	-0.6791	0.5649	1.1830

Species according to VIPRBRC	GenBank Accession	GenBank Protein Accession	Subgenotype	Protein	Strain Name	Collection Year	SVM Patho. Score	Vaxijen Antig. Score	Averged score of EMBOSS motifs
Pestivirus B	MH231140	VIPR_ALG4_AZQ0066	2a	C	PA	1992	-0.5429	0.5623	1.1830
Pestivirus B	MH231143	VIPR_ALG4_AZQ0066	2a	C	RS886	2014	-0.5429	0.5623	1.1830
Pestivirus B	HQ258810	VIPR_ALG4_31199027	2a	C	SH-28	2009	-0.5658	0.5070	1.1830
Pestivirus B	MH231145	VIPR_ALG4_AZQ0066	2a	C	Victor301	1990	-0.8965	0.5587	1.1830
Pestivirus B	MH231146	VIPR_ALG4_AZQ0067	2a	C	WiscA	1991	-0.0770	0.5543	1.1715
Pestivirus B	FJ527854	VIPR_ALG4_22961018	2a	C	XJ-04	2004	-0.8736	0.5296	1.1830
Pestivirus B	KC963968	VIPR_ALG4_53029119	2a	C	11F011	2011	-0.3418	0.5487	1.1830
Pestivirus B	MH806437	VIPR_ALG4_AZP5716	2a	C	9231	2004	-0.2122	0.5240	1.1830
Pestivirus B	MG879027	VIPR_ALG4_AVA3071	2a	C	CN10.2015.821	2014	-0.1923	0.5576	1.1830
Pestivirus B	KX096718	VIPR_ALG4_11130170	2a	C	HB-1511	2015	-0.5624	0.5969	1.2150
Pestivirus B	LC649064	VIPR_ALG4_BDB0736	2c	C	KZ-91-NCP	1991	-0.3488	0.5892	1.2055
Pestivirus B	MH231147	VIPR_ALG4_AZQ0067	2c	C	PI12	2016	-0.2696	0.6156	1.2090
Pestivirus B	HQ444199	VIPR_ALG4_32680714	2a	C	Ind141353	2007	-0.5912	0.6011	1.1830
Pestivirus B	GQ888686	VIPR_ALG4_27071949	2a	C	JZ05-1	2005	-0.7438	0.5660	1.1830
Pestivirus B	FJ431189	VIPR_ALG4_21636083	2a	C	53099	2007	-0.2879	0.5805	1.1700
Pestivirus B	FJ431190	VIPR_ALG4_21636083	2a	C	53100	2007	-0.3577	0.5963	1.1700
Pestivirus B	FJ431192	VIPR_ALG4_21636085	2a	C	65	2007	-0.2879	0.5805	1.1700
Pestivirus B	FJ431193	VIPR_ALG4_21636086	2a	C	68	2007	-0.2879	0.5805	1.1700
Pestivirus B	FJ431194	VIPR_ALG4_21636086	2a	C	73	2007	-0.2879	0.5805	1.1700
Pestivirus B	MT024568	VIPR_ALG4_QPK4118	2a	Erns	560615-F0-22	2015	-0.3620	0.4451	1.0680
Pestivirus B	EU747875	VIPR_ALG4_19033620	2b	Erns	17237	2004	0.0800	0.4873	1.0976
Pestivirus B	MW006485	VIPR_ALG4_QPF4972	2b	Erns	HEN01	2014	-0.4633	0.5635	1.0970
Pestivirus B	MH231142	VIPR_ALG4_AZQ0066	2c	Erns	Parker	1991	-0.2596	0.5274	1.1022
Pestivirus B	MH806435	VIPR_ALG4_AZP5716	2a	Erns	1336H	2005	-0.4534	0.5355	1.1104
Pestivirus B	MH231136	VIPR_ALG4_AZQ0066	2a	Erns	JV14	1998	-0.3602	0.5289	1.1044
Pestivirus B	MK599227	VIPR_ALG4_QEU5262	2a	Erns	SD-1	2016	-0.5750	0.5023	1.1073
Pestivirus B	HG426488	VIPR_ALG4_61932575	2c	Erns	NRW 19-13-1_Dup(+)	2013	-0.2300	0.5233	1.1072
Pestivirus B	HG426487	VIPR_ALG4_61932574	2c	Erns	NRW 19-13-1_Dup(-)	2013	-0.2300	0.5233	1.1072
Pestivirus B	MH231138	VIPR_ALG4_AZQ0066	2a	Erns	MnFetus	1991	-0.4370	0.5149	1.1063
Pestivirus B	MH231131	VIPR_ALG4_AZQ0065	2a	Erns	AU501	2006	-0.2962	0.5383	1.1156
Pestivirus B	MH806434	VIPR_ALG4_AZP5716	2a	Erns	125c	1990	-0.2732	0.5316	1.1056
Pestivirus B	MH231151	VIPR_ALG4_AZQ0067	2e	Erns	14622	2005	-0.4145	0.5513	1.0992
Pestivirus B	MH231152	VIPR_ALG4_AZQ0067	2e	Erns	2412	1989	-0.4090	0.5175	1.0992
Pestivirus B	MH231127	VIPR_ALG4_AZQ0065	2a	Erns	53637c	2004	-0.3701	0.5200	1.1044
Pestivirus B	MH231149	VIPR_ALG4_AZQ0067	2e	Erns	Short	1989	-0.4145	0.5513	1.0992
Pestivirus B	MH231129	VIPR_ALG4_AZQ0065	2a	Erns	5912c	1995	-0.4379	0.4789	1.1063
Pestivirus B	KJ000672	VIPR_ALG4_59423572	2b	Erns	SD1301	2012	-0.4748	0.5626	1.0970
Pestivirus B	MH231148	VIPR_ALG4_AZQ0067	2e	Erns	12-149150	2012	-0.4433	0.5179	1.0992
Pestivirus B	MH231150	VIPR_ALG4_AZQ0067	2e	Erns	12-151955-317	2012	-0.4433	0.5179	1.0992
Pestivirus B	MH231133	VIPR_ALG4_AZQ0065	2e	Erns	B69519c	2006	-0.4090	0.5175	1.0992
Pestivirus B	HG426495	VIPR_ALG4_61932576	2c	Erns	VOE 4407	2007	-0.1721	0.5313	1.1022
Pestivirus B	HG426480	VIPR_ALG4_61932573	2c	Erns	D37-13-2_Dup(+)	2013	-0.2300	0.5233	1.1072
Pestivirus B	HG426482	VIPR_ALG4_61932573	2c	Erns	D75-13-609_Dup(+)	2013	-0.2300	0.5233	1.1072
Pestivirus B	HG426486	VIPR_ALG4_61932574	2c	Erns	NRW 14-13_Dup(+)	2013	-0.2300	0.5233	1.1072
Pestivirus B	HG426491	VIPR_ALG4_61932575	2c	Erns	Potsdam 1600	2000	-0.2281	0.5269	1.1022
Pestivirus B	MH231141	VIPR_ALG4_AZQ0066	2a	Erns	PI28	2016	-0.3869	0.5353	1.1103
Pestivirus B	HG426494	VIPR_ALG4_61932576	2c	Erns	SH2210-23	2010	-0.1573	0.5222	1.0983

Species according to VIPRBRC	GenBank Accession	GenBank Protein Accession	Subgenotype	Protein	Strain Name	Collection Year	SVM Patho. Score	Vaxijen Antig. Score	Averged score of EMBOSS motifs
Pestivirus B	MH806436	VIPR_ALG4_AZP5716	2a	Erns	296c	1995	-0.3673	0.4772	1.1023
Pestivirus B	MH231137	VIPR_ALG4_AZQ0066	2a	Erns	MadSpl	1991	-0.3869	0.5353	1.1103
Pestivirus B	KP941585	VIPR_ALG4_80092432	2a	Erns	USMARC-55476	2014	-0.3573	0.5510	1.1011
Pestivirus B	KT832820	VIPR_ALG4_99822633	2a	Erns	USMARC-60767	2014	-0.3539	0.5292	1.1103
Pestivirus B	MH231134	VIPR_ALG4_AZQ0065	2a	Erns	B9497	1997	-0.2682	0.5242	1.1056
Pestivirus B	MN527354	VIPR_ALG4_QLH0204	2a	Erns	GS2018	2018	-0.3917	0.5416	1.1098
Pestivirus B	MH231144	VIPR_ALG4_AZQ0066	2a	Erns	Sanderson6319	1992	-0.4554	0.5253	1.1057
Pestivirus B	HG426493	VIPR_ALG4_61932576	2c	Erns	SH2210-17	2010	-0.1456	0.5359	1.0984
Pestivirus B	KT832817	VIPR_ALG4_99822632	2a	Erns	USMARC-60764	2014	-0.2632	0.5283	1.1058
Pestivirus B	KT832822	VIPR_ALG4_99822633	2a	Erns	USMARC-60779	2014	-0.3296	0.5259	1.1144
Pestivirus B	MH231125	VIPR_ALG4_AZQ0064	2a	Erns	2139	1992	-0.4886	0.5211	1.1103
Pestivirus B	KP941582	VIPR_ALG4_80092431	2c	Erns	USMARC-53873	2014	-0.2345	0.5336	1.1048
Pestivirus B	KT832819	VIPR_ALG4_99822632	2a	Erns	USMARC-60766	2014	-0.4860	0.5799	1.1013
Pestivirus B	KT832821	VIPR_ALG4_99822633	2c	Erns	USMARC-60768	2014	-0.3036	0.5172	1.1043
Pestivirus B	HG426479	VIPR_ALG4_61932573	2c	Erns	D37-13-2_Dup(-)	2013	-0.2300	0.5233	1.1072
Pestivirus B	HG426481	VIPR_ALG4_61932573	2c	Erns	D75-13-609_Dup(-)	2013	-0.2300	0.5233	1.1072
Pestivirus B	MH231123	VIPR_ALG4_AZQ0064	2a	Erns	10406	1993	-0.4908	0.5348	1.1064
Pestivirus B	HG426492	VIPR_ALG4_61932575	2c	Erns	SH2210-14	2010	-0.1456	0.5359	1.0984
Pestivirus B	KT832823	VIPR_ALG4_99822633	2a	Erns	USMARC-60780	2014	-0.6031	0.5185	1.1123
Pestivirus B	HG426485	VIPR_ALG4_61932574	2c	Erns	NRW 14-13_Dup(-)	2013	-0.2300	0.5233	1.1072
Pestivirus B	MH231126	VIPR_ALG4_AZQ0065	2b	Erns	3237	1990	-0.4036	0.5593	1.1001
Pestivirus B	MH806438	VIPR_ALG4_AZP5716	2a	Erns	McCart_c	1989	-0.2946	0.5271	1.1098
Pestivirus B	KT832818	VIPR_ALG4_99822632	2a	Erns	USMARC-60765	2014	-0.3527	0.5309	1.1036
Pestivirus B	MH231124	VIPR_ALG4_AZQ0064	2e	Erns	1786c	1989	-0.4090	0.5175	1.0992
Pestivirus B	HG426483	VIPR_ALG4_61932574	2c	Erns	NRW 12-13_Dup(-)	2013	-0.2300	0.5233	1.1072
Pestivirus B	HG426484	VIPR_ALG4_61932574	2c	Erns	NRW 12-13_Dup(+)	2013	-0.2300	0.5233	1.1072
Pestivirus B	HG426489	VIPR_ALG4_61932575	2c	Erns	NRW 19-13-8_Dup(-)	2013	-0.2300	0.5233	1.1072
Pestivirus B	HG426490	VIPR_ALG4_61932575	2c	Erns	NRW 19-13-8_Dup(+)	2013	-0.2300	0.5233	1.1072
Pestivirus B	MH231130	VIPR_ALG4_AZQ0065	2a	Erns	95-1501	1998	-0.3649	0.5308	1.1071
Pestivirus B	MH231132	VIPR_ALG4_AZQ0065	2a	Erns	AzSpl	1997	-0.3326	0.5203	1.1002
Pestivirus B	MH231128	VIPR_ALG4_AZQ0065	2a	Erns	570152	1992	-0.3939	0.5237	1.1063
Pestivirus B	MH231135	VIPR_ALG4_AZQ0065	2a	Erns	BV1907	1995	-0.3701	0.5200	1.1044
Pestivirus B	JF714967	VIPR_ALG4_34657797	2a	Erns	HLJ-10	2011	-0.5211	0.5732	1.1104
Pestivirus B	MH231139	VIPR_ALG4_AZQ0066	2a	Erns	Olwein #12	1990	-0.5811	0.5273	1.1063
Pestivirus B	MH231140	VIPR_ALG4_AZQ0066	2a	Erns	PA	1992	-0.3854	0.5325	1.1063
Pestivirus B	MH231143	VIPR_ALG4_AZQ0066	2a	Erns	RS886	2014	-0.3879	0.5410	1.1063
Pestivirus B	HQ258810	VIPR_ALG4_31199027	2a	Erns	SH-28	2009	-0.4056	0.5257	1.1138
Pestivirus B	MH231145	VIPR_ALG4_AZQ0066	2a	Erns	Victor301	1990	-0.3602	0.5289	1.1044
Pestivirus B	MH231146	VIPR_ALG4_AZQ0067	2a	Erns	WiscA	1991	-0.4832	0.5328	1.1121
Pestivirus B	FJ527854	VIPR_ALG4_22961018	2a	Erns	XJ-04	2004	-0.4522	0.4969	1.1088
Pestivirus B	KC963968	VIPR_ALG4_53029119	2a	Erns	11F011	2011	-0.4401	0.4729	1.1063
Pestivirus B	MH806437	VIPR_ALG4_AZP5716	2a	Erns	9231	2004	-0.2572	0.5555	1.1188
Pestivirus B	MG879027	VIPR_ALG4_AVA3071	2a	Erns	CN10.2015.821	2014	-0.2572	0.5555	1.1188
Pestivirus B	KX096718	VIPR_ALG4_11130170	2a	Erns	HB-1511	2015	-0.3395	0.5006	1.1086
Pestivirus B	LC649064	VIPR_ALG4_BDB0736	2c	Erns	KZ-91-NCP	1991	-0.0468	0.5233	1.1044
Pestivirus B	MH231147	VIPR_ALG4_AZQ0067	2c	Erns	PI12	2016	-0.1853	0.5344	1.1022
Pestivirus B	HQ444199	VIPR_ALG4_32680714	2a	Erns	Ind141353	2007	-0.6864	0.5520	1.1064

Species according to VIPRBRC	GenBank Accession	GenBank Protein Accession	Subgenotype	Protein	Strain Name	Collection Year	SVM Patho. Score	Vaxijen Antig. Score	Averged score of EMBOSS motifs
Pestivirus B	GQ888686	VIPR_ALG4_27071949	2a	Erns	JZ05-1	2005	-0.3437	0.4831	1.1023
Pestivirus B	KT875134	VIPR_ALG4_10037029	2a	Erns	12W	2005	-0.3155	0.5391	1.1156
Pestivirus B	KT875135	VIPR_ALG4_10037029	2a	Erns	13Y	2005	-0.3155	0.5391	1.1156
Pestivirus B	KP057803	VIPR_ALG4_80287536	2a	Erns	24515	1993	-0.3701	0.5200	1.1044
Pestivirus B	KT875136	VIPR_ALG4_10037029	2a	Erns	27Y	2005	-0.3155	0.5391	1.1156
Pestivirus B	KT875137	VIPR_ALG4_10037029	2a	Erns	29Y	2005	-0.3155	0.5391	1.1156
Pestivirus B	KT875138	VIPR_ALG4_10037029	2a	Erns	2Y	2005	-0.3155	0.5391	1.1156
Pestivirus B	KT875140	VIPR_ALG4_10037029	2a	Erns	34Y	2005	-0.3155	0.5391	1.1156
Pestivirus B	KT875141	VIPR_ALG4_10037029	2a	Erns	36W	2005	-0.3017	0.5405	1.1156
Pestivirus B	KT875142	VIPR_ALG4_10037029	2a	Erns	41Y	2005	-0.3155	0.5391	1.1156
Pestivirus B	KT875143	VIPR_ALG4_10037029	2a	Erns	42W	2005	-0.3155	0.5391	1.1156
Pestivirus B	KT875144	VIPR_ALG4_10037029	2a	Erns	43Y	2005	-0.3155	0.5391	1.1156
Pestivirus B	KT875145	VIPR_ALG4_10037029	2a	Erns	47Y	2005	-0.3155	0.5391	1.1156
Pestivirus B	KT875146	VIPR_ALG4_10037029	2a	Erns	50Y	2005	-0.2991	0.5401	1.1156
Pestivirus B	KT875147	VIPR_ALG4_10037029	2a	Erns	51W	2005	-0.3155	0.5391	1.1156
Pestivirus B	KT875148	VIPR_ALG4_10037029	2a	Erns	51Y	2005	-0.3155	0.5391	1.1156
Pestivirus B	KT875149	VIPR_ALG4_10037029	2a	Erns	53W	2005	-0.3155	0.5391	1.1156
Pestivirus B	KT875150	VIPR_ALG4_10037029	2a	Erns	58W	2005	-0.3155	0.5391	1.1156
Pestivirus B	KT875151	VIPR_ALG4_10037029	2a	Erns	58Y	2005	-0.3155	0.5391	1.1156
Pestivirus B	KT875152	VIPR_ALG4_10037029	2a	Erns	5Y	2005	-0.3155	0.5391	1.1156
Pestivirus B	KT875153	VIPR_ALG4_10037029	2a	Erns	62Y	2005	-0.3155	0.5391	1.1156
Pestivirus B	KT875154	VIPR_ALG4_10037029	2a	Erns	65Y	2005	-0.3665	0.5376	1.1156
Pestivirus B	KT875155	VIPR_ALG4_10037029	2a	Erns	67Y	2005	-0.3155	0.5391	1.1156
Pestivirus B	KT875156	VIPR_ALG4_10037029	2a	Erns	68W	2005	-0.3155	0.5391	1.1156
Pestivirus B	KT875157	VIPR_ALG4_10037029	2a	Erns	71Y	2005	-0.3155	0.5391	1.1156
Pestivirus B	KT875158	VIPR_ALG4_10037029	2a	Erns	73Y	2005	-0.3155	0.5391	1.1156
Pestivirus B	KT875159	VIPR_ALG4_10037029	2a	Erns	74Y	2005	-0.3155	0.5391	1.1156
Pestivirus B	KT875160	VIPR_ALG4_10037029	2a	Erns	75W	2005	-0.3155	0.5391	1.1156
Pestivirus B	KT875161	VIPR_ALG4_10037029	2a	Erns	75Y	2005	-0.3155	0.5391	1.1156
Pestivirus B	KT875162	VIPR_ALG4_10037029	2a	Erns	76Y	2005	-0.3155	0.5391	1.1156
Pestivirus B	KT875163	VIPR_ALG4_10037029	2a	Erns	78W	2005	-0.3155	0.5391	1.1156
Pestivirus B	KT875164	VIPR_ALG4_10037029	2a	Erns	79W	2005	-0.3155	0.5391	1.1156
Pestivirus B	KT875165	VIPR_ALG4_10037029	2a	Erns	7W	2005	-0.3155	0.5391	1.1156
Pestivirus B	KT875166	VIPR_ALG4_10037029	2a	Erns	82W	2005	-0.3155	0.5391	1.1156
Pestivirus B	KT875167	VIPR_ALG4_10037029	2a	Erns	83Y	2005	-0.3155	0.5391	1.1156
Pestivirus B	KT875168	VIPR_ALG4_10037029	2a	Erns	90W	2005	-0.3155	0.5391	1.1156
Pestivirus B	KT875169	VIPR_ALG4_10037030	2a	Erns	91W	2005	-0.3199	0.4970	1.1156
Pestivirus B	MN824468	VIPR_ALG4_QJF1227	2a	Erns	CPAE_contamination/2018	2018	-0.5823	0.5248	1.1101
Pestivirus B	KR093034	VIPR_ALG4_92904888	2a	Erns	NY-93	1993	-0.3701	0.5200	1.1044
Pestivirus B	KT875139	VIPR_ALG4_10037029	2a	Erns	32W	2005	-0.2835	0.5404	1.1156
Pestivirus B	KX170219	APT70742.1	2a	Erns	V002	1999	-0.4624	0.5441	1.1063
Pestivirus B	KX170218	APT70741.1	2a	Erns	V005	1999	-0.4178	0.5414	1.1063
Pestivirus B	KX170205	APT70728.1	2a	Erns	V017	2003	-0.3045	0.5633	1.1188
Pestivirus B	KX170200	APT70723.1	2a	Erns	V019	2004	-0.3548	0.5531	1.1174
Pestivirus B	KX170220	APT70743.1	2a	Erns	V021	2005	-0.4237	0.5339	1.1120
Pestivirus B	KX170211	APT70734.1	2a	Erns	V023	2006	-0.4192	0.5288	1.1094
Pestivirus B	KX170217	APT70740.1	2a	Erns	V024	2006	-0.5169	0.5382	1.1044

Species according to VIPRBRC	GenBank Accession	GenBank Protein Accession	Subgenotype	Protein	Strain Name	Collection Year	SVM Patho. Score	Vaxijen Antig. Score	Averged score of EMBOSS motifs
Pestivirus B	KX170213	APT70736.1	2a	Erns	V025	2006	-0.4430	0.5365	1.1044
Pestivirus B	KX170197	APT70720.1	2a	Erns	V028	2006	-0.5106	0.5531	1.1068
Pestivirus B	KX170221	APT70744.1	2a	Erns	V037	2008	-0.4567	0.5408	1.1063
Pestivirus B	KX170212	APT70735.1	2a	Erns	V044	2008	-0.4690	0.5355	1.1104
Pestivirus B	KX170206	APT70729.1	2a	Erns	V047	2009	-0.3045	0.5633	1.1188
Pestivirus B	KX170215	APT70738.1	2a	Erns	V051	2010	-0.4261	0.5195	1.1044
Pestivirus B	KX170198	APT70721.1	2a	Erns	V062	2005	-0.2076	0.5600	1.1149
Pestivirus B	KX170204	APT70727.1	2a	Erns	V063	2005	-0.2349	0.5593	1.1137
Pestivirus B	KX170202	APT70725.1	2a	Erns	V076	2011	-0.4102	0.4974	1.1224
Pestivirus B	KX170222	APT70745.1	2a	Erns	V079	2010	-0.6132	0.5470	1.1063
Pestivirus B	KX170201	APT70724.1	2a	Erns	V081	2009	-0.2921	0.5239	1.1188
Pestivirus B	KX170199	APT70722.1	2a	Erns	V082	2008	-0.3045	0.5633	1.1188
Pestivirus B	KX170203	APT70726.1	2a	Erns	V085	2007	-0.2989	0.5202	1.1188
Pestivirus B	KX170209	APT70732.1	2a	Erns	V086	2006	-0.4251	0.4935	1.1101
Pestivirus B	KX170207	APT70730.1	2a	Erns	V088	2005	-0.2796	0.5388	1.1036
Pestivirus B	KX170210	APT70733.1	2a	Erns	V089	2004	-0.3725	0.5508	1.1109
Pestivirus B	KX170208	APT70731.1	2a	Erns	V095	1999	-0.3003	0.5406	1.1156
Pestivirus B	KX170216	APT70739.1	2a	Erns	V097	1998	-0.4926	0.5314	1.1063
Pestivirus B	KX170214	APT70737.1	2a	Erns	V065	2006	-0.5358	0.5586	1.1043
Pestivirus B	KT875139	VIPR_ALG4_10037029	2a	E1	32W	2005	-0.7658	0.4848	1.1899
Pestivirus B	KT875134	VIPR_ALG4_10037029	2a	E1	12W	2005	-0.8125	0.4839	1.1899
Pestivirus B	KT875135	VIPR_ALG4_10037029	2a	E1	13Y	2005	-0.8125	0.4839	1.1899
Pestivirus B	KP057803	VIPR_ALG4_80287536	2a	E1	24515	1993	-1.1085	0.4144	1.1675
Pestivirus B	KT875136	VIPR_ALG4_10037029	2a	E1	27Y	2005	-0.7658	0.4848	1.1899
Pestivirus B	KT875137	VIPR_ALG4_10037029	2a	E1	29Y	2005	-0.7658	0.4848	1.1899
Pestivirus B	KT875138	VIPR_ALG4_10037029	2a	E1	2Y	2005	-0.8125	0.4839	1.1899
Pestivirus B	KT875140	VIPR_ALG4_10037029	2a	E1	34Y	2005	-0.7658	0.4848	1.1899
Pestivirus B	KT875141	VIPR_ALG4_10037029	2a	E1	36W	2005	-0.8125	0.4839	1.1899
Pestivirus B	KT875142	VIPR_ALG4_10037029	2a	E1	41Y	2005	-0.7658	0.4848	1.1899
Pestivirus B	KT875143	VIPR_ALG4_10037029	2a	E1	42W	2005	-0.8005	0.4922	1.1899
Pestivirus B	KT875144	VIPR_ALG4_10037029	2a	E1	43Y	2005	-0.8125	0.4839	1.1899
Pestivirus B	KT875145	VIPR_ALG4_10037029	2a	E1	47Y	2005	-0.7658	0.4848	1.1899
Pestivirus B	KT875146	VIPR_ALG4_10037029	2a	E1	50Y	2005	-0.7658	0.4848	1.1899
Pestivirus B	KT875147	VIPR_ALG4_10037029	2a	E1	51W	2005	-0.7658	0.4848	1.1899
Pestivirus B	KT875148	VIPR_ALG4_10037029	2a	E1	51Y	2005	-0.8125	0.4839	1.1899
Pestivirus B	KT875149	VIPR_ALG4_10037029	2a	E1	53W	2005	-0.7658	0.4848	1.1899
Pestivirus B	KT875150	VIPR_ALG4_10037029	2a	E1	58W	2005	-0.7658	0.4848	1.1899
Pestivirus B	KT875151	VIPR_ALG4_10037029	2a	E1	58Y	2005	-0.7658	0.4848	1.1899
Pestivirus B	KT875152	VIPR_ALG4_10037029	2a	E1	5Y	2005	-0.7658	0.4848	1.1899
Pestivirus B	KT875153	VIPR_ALG4_10037029	2a	E1	62Y	2005	-0.7658	0.4848	1.1899
Pestivirus B	KT875154	VIPR_ALG4_10037029	2a	E1	65Y	2005	-0.6842	0.4644	1.1899
Pestivirus B	KT875155	VIPR_ALG4_10037029	2a	E1	67Y	2005	-0.7658	0.4848	1.1899
Pestivirus B	KT875156	VIPR_ALG4_10037029	2a	E1	68W	2005	-0.8125	0.4839	1.1899
Pestivirus B	KT875157	VIPR_ALG4_10037029	2a	E1	71Y	2005	-0.7658	0.4848	1.1899
Pestivirus B	KT875158	VIPR_ALG4_10037029	2a	E1	73Y	2005	-0.8125	0.4839	1.1899
Pestivirus B	KT875159	VIPR_ALG4_10037029	2a	E1	74Y	2005	-0.7658	0.4848	1.1899
Pestivirus B	KT875160	VIPR_ALG4_10037029	2a	E1	75W	2005	-0.7658	0.4848	1.1899

Species according to VIPRBRC	GenBank Accession	GenBank Protein Accession	Subgenotype	Protein	Strain Name	Collection Year	SVM Patho. Score	Vaxijen Antig. Score	Averged score of EMBOSS motifs
Pestivirus B	KT875161	VIPR_ALG4_10037029	2a	E1	75Y	2005	-0.7658	0.4848	1.1899
Pestivirus B	KT875162	VIPR_ALG4_10037029	2a	E1	76Y	2005	-0.7658	0.4848	1.1899
Pestivirus B	KT875163	VIPR_ALG4_10037029	2a	E1	78W	2005	-0.8757	0.4897	1.1899
Pestivirus B	KT875164	VIPR_ALG4_10037029	2a	E1	79W	2005	-0.8125	0.4839	1.1899
Pestivirus B	KT875165	VIPR_ALG4_10037029	2a	E1	7W	2005	-0.7807	0.4883	1.1719
Pestivirus B	KT875166	VIPR_ALG4_10037029	2a	E1	82W	2005	-0.7658	0.4848	1.1899
Pestivirus B	KT875167	VIPR_ALG4_10037029	2a	E1	83Y	2005	-0.7658	0.4848	1.1899
Pestivirus B	KT875168	VIPR_ALG4_10037029	2a	E1	90W	2005	-0.8125	0.4839	1.1899
Pestivirus B	KT875169	VIPR_ALG4_10037030	2a	E1	91W	2005	-0.7658	0.4848	1.1899
Pestivirus B	MN824468	VIPR_ALG4_QJF1227	2a	E1	CPAE_contamination/2018	2018	-1.0825	0.5028	1.1789
Pestivirus B	KR093034	VIPR_ALG4_92904888	2a	E1	NY-93	1993	-1.0904	0.4121	1.1675
Pestivirus B	EU747875	VIPR_ALG4_19033620	2b	E1	17237	2004	-1.0502	0.5313	1.1911
Pestivirus B	MW006485	VIPR_ALG4_QPF4972	2b	E1	HEN01	2014	-0.7582	0.5662	1.1730
Pestivirus B	MH231142	VIPR_ALG4_AZQ0066	2c	E1	Parker	1991	-0.7762	0.4781	1.2032
Pestivirus B	MH806435	VIPR_ALG4_AZP5716	2a	E1	1336H	2005	-1.0975	0.4826	1.1939
Pestivirus B	MH231136	VIPR_ALG4_AZQ0066	2a	E1	JV14	1998	-1.1548	0.4803	1.1739
Pestivirus B	MK599227	VIPR_ALG4_QEU5262	2a	E1	SD-1	2016	-0.9046	0.4295	1.2137
Pestivirus B	HG426488	VIPR_ALG4_61932575	2c	E1	NRW 19-13-1_Dup(+)	2013	-0.7762	0.4781	1.2032
Pestivirus B	HG426487	VIPR_ALG4_61932574	2c	E1	NRW 19-13-1_Dup(-)	2013	-0.7762	0.4781	1.2032
Pestivirus B	MH231138	VIPR_ALG4_AZQ0066	2a	E1	MnFetus	1991	-1.2099	0.4789	1.1739
Pestivirus B	MH231131	VIPR_ALG4_AZQ0065	2a	E1	AU501	2006	-0.4918	0.4808	1.1913
Pestivirus B	MH806434	VIPR_ALG4_AZP5716	2a	E1	125c	1990	-0.9095	0.4732	1.1988
Pestivirus B	MH231151	VIPR_ALG4_AZQ0067	2e	E1	14622	2005	-1.0007	0.4930	1.1730
Pestivirus B	MH231152	VIPR_ALG4_AZQ0067	2e	E1	2412	1989	-1.1567	0.4923	1.1841
Pestivirus B	MH231127	VIPR_ALG4_AZQ0065	2a	E1	53637c	2004	-1.1292	0.4891	1.1756
Pestivirus B	MH231149	VIPR_ALG4_AZQ0067	2e	E1	Short	1989	-1.0007	0.4930	1.1730
Pestivirus B	MH231129	VIPR_ALG4_AZQ0065	2a	E1	5912c	1995	-0.9563	0.4750	1.1988
Pestivirus B	KJ000672	VIPR_ALG4_59423572	2b	E1	SD1301	2012	-0.6478	0.5739	1.1730
Pestivirus B	MH231148	VIPR_ALG4_AZQ0067	2e	E1	12-149150	2012	-1.0611	0.4841	1.1841
Pestivirus B	MH231150	VIPR_ALG4_AZQ0067	2e	E1	12-151955-317	2012	-1.0952	0.4977	1.1800
Pestivirus B	MH231133	VIPR_ALG4_AZQ0065	2e	E1	B69519c	2006	-1.1567	0.4923	1.1841
Pestivirus B	HG426495	VIPR_ALG4_61932576	2c	E1	VOE 4407	2007	-0.7247	0.4795	1.2032
Pestivirus B	HG426480	VIPR_ALG4_61932573	2c	E1	D37-13-2_Dup(+)	2013	-0.7762	0.4781	1.2032
Pestivirus B	HG426482	VIPR_ALG4_61932573	2c	E1	D75-13-609_Dup(+)	2013	-0.7762	0.4781	1.2032
Pestivirus B	HG426486	VIPR_ALG4_61932574	2c	E1	NRW 14-13_Dup(+)	2013	-0.7762	0.4781	1.2032
Pestivirus B	HG426491	VIPR_ALG4_61932575	2c	E1	Potsdam 1600	2000	-0.8723	0.4738	1.2032
Pestivirus B	MH231141	VIPR_ALG4_AZQ0066	2a	E1	PI28	2016	-1.1511	0.4981	1.1738
Pestivirus B	HG426494	VIPR_ALG4_61932576	2c	E1	SH2210-23	2010	-0.4778	0.4932	1.2032
Pestivirus B	MH806436	VIPR_ALG4_AZP5716	2a	E1	296c	1995	-0.9563	0.4750	1.1988
Pestivirus B	MH231137	VIPR_ALG4_AZQ0066	2a	E1	MadSpl	1991	-1.0876	0.5032	1.1738
Pestivirus B	KP941585	VIPR_ALG4_80092432	2a	E1	USMARC-55476	2014	-0.7963	0.4587	1.1823
Pestivirus B	KT832820	VIPR_ALG4_99822633	2a	E1	USMARC-60767	2014	-1.0063	0.4104	1.1803
Pestivirus B	MH231134	VIPR_ALG4_AZQ0065	2a	E1	B9497	1997	-0.6352	0.4759	1.1968
Pestivirus B	MN527354	VIPR_ALG4_QLH0204	2a	E1	GS2018	2018	-0.6352	0.4759	1.1968
Pestivirus B	MH231144	VIPR_ALG4_AZQ0066	2a	E1	Sanderson6319	1992	-1.3794	0.4886	1.1803
Pestivirus B	HG426493	VIPR_ALG4_61932576	2c	E1	SH2210-17	2010	-0.6900	0.4820	1.2087
Pestivirus B	KT832817	VIPR_ALG4_99822632	2a	E1	USMARC-60764	2014	-0.5829	0.4775	1.1800

Species according to VIPRBRC	GenBank Accession	GenBank Protein Accession	Subgenotype	Protein	Strain Name	Collection Year	SVM Patho. Score	Vaxijen Antig. Score	Averged score of EMBOSS motifs
Pestivirus B	KT832822	VIPR_ALG4_99822633	2a	E1	USMARC-60779	2014	-1.0393	0.4856	1.1806
Pestivirus B	MH231125	VIPR_ALG4_AZQ0064	2a	E1	2139	1992	-1.1719	0.4948	1.1789
Pestivirus B	KP941582	VIPR_ALG4_80092431	2c	E1	USMARC-53873	2014	-0.5125	0.5283	1.2032
Pestivirus B	KT832819	VIPR_ALG4_99822632	2a	E1	USMARC-60766	2014	-0.8924	0.5125	1.1800
Pestivirus B	KT832821	VIPR_ALG4_99822633	2c	E1	USMARC-60768	2014	-0.5213	0.4974	1.1780
Pestivirus B	HG426479	VIPR_ALG4_61932573	2c	E1	D37-13-2_Dup(-)	2013	-0.7762	0.4781	1.2032
Pestivirus B	HG426481	VIPR_ALG4_61932573	2c	E1	D75-13-609_Dup(-)	2013	-0.7762	0.4781	1.2032
Pestivirus B	MH231123	VIPR_ALG4_AZQ0064	2a	E1	10406	1993	-0.9319	0.4803	1.1664
Pestivirus B	HG426492	VIPR_ALG4_61932575	2c	E1	SH2210-14	2010	-0.6900	0.4820	1.2087
Pestivirus B	KT832823	VIPR_ALG4_99822633	2a	E1	USMARC-60780	2014	-1.0146	0.4781	1.1988
Pestivirus B	HG426485	VIPR_ALG4_61932574	2c	E1	NRW 14-13_Dup(-)	2013	-0.7762	0.4781	1.2032
Pestivirus B	MH231126	VIPR_ALG4_AZQ0065	2b	E1	3237	1990	-0.6488	0.5338	1.1771
Pestivirus B	MH806438	VIPR_ALG4_AZP5716	2a	E1	McCart_c	1989	-0.8825	0.4917	1.1714
Pestivirus B	KT832818	VIPR_ALG4_99822632	2a	E1	USMARC-60765	2014	-0.8872	0.4478	1.2057
Pestivirus B	MH231124	VIPR_ALG4_AZQ0064	2e	E1	1786c	1989	-1.1567	0.4923	1.1841
Pestivirus B	HG426483	VIPR_ALG4_61932574	2c	E1	NRW 12-13_Dup(-)	2013	-0.7762	0.4781	1.2032
Pestivirus B	HG426484	VIPR_ALG4_61932574	2c	E1	NRW 12-13_Dup(+)	2013	-0.7762	0.4781	1.2032
Pestivirus B	HG426489	VIPR_ALG4_61932575	2c	E1	NRW 19-13-8_Dup(-)	2013	-0.7762	0.4781	1.2032
Pestivirus B	HG426490	VIPR_ALG4_61932575	2c	E1	NRW 19-13-8_Dup(+)	2013	-0.7762	0.4781	1.2032
Pestivirus B	MH231130	VIPR_ALG4_AZQ0065	2a	E1	95-1501	1998	-0.8825	0.4917	1.1714
Pestivirus B	MH231132	VIPR_ALG4_AZQ0065	2a	E1	AzSpl	1997	-0.9463	0.5043	1.1865
Pestivirus B	MH231128	VIPR_ALG4_AZQ0065	2a	E1	570152	1992	-1.1582	0.4672	1.1700
Pestivirus B	MH231135	VIPR_ALG4_AZQ0065	2a	E1	BV1907	1995	-1.1484	0.4707	1.1688
Pestivirus B	JF714967	VIPR_ALG4_34657797	2a	E1	HLJ-10	2011	-0.7726	0.4800	1.1939
Pestivirus B	MH231139	VIPR_ALG4_AZQ0066	2a	E1	Olwein #12	1990	-0.9824	0.4803	1.1988
Pestivirus B	MH231140	VIPR_ALG4_AZQ0066	2a	E1	PA	1992	-1.2731	0.4740	1.1759
Pestivirus B	MH231143	VIPR_ALG4_AZQ0066	2a	E1	RS886	2014	-1.1474	0.4848	1.1720
Pestivirus B	HQ258810	VIPR_ALG4_31199027	2a	E1	SH-28	2009	-0.9016	0.4845	1.2032
Pestivirus B	MH231145	VIPR_ALG4_AZQ0066	2a	E1	Victor301	1990	-1.1439	0.4794	1.1664
Pestivirus B	MH231146	VIPR_ALG4_AZQ0067	2a	E1	WiscA	1991	-1.1511	0.4981	1.1738
Pestivirus B	FJ527854	VIPR_ALG4_22961018	2a	E1	XJ-04	2004	-1.0452	0.4540	1.2000
Pestivirus B	KC963968	VIPR_ALG4_53029119	2a	E1	11F011	2011	-1.1237	0.4760	1.1788
Pestivirus B	MH806437	VIPR_ALG4_AZP5716	2a	E1	9231	2004	-0.6244	0.4953	1.1899
Pestivirus B	MG879027	VIPR_ALG4_AVA3071	2a	E1	CN10.2015.821	2014	-0.4871	0.5063	1.1851
Pestivirus B	KX096718	VIPR_ALG4_11130170	2a	E1	HB-1511	2015	-0.8060	0.4583	1.2000
Pestivirus B	LC649064	VIPR_ALG4_BDB0736	2c	E1	KZ-91-NCP	1991	-0.5973	0.4829	1.2032
Pestivirus B	MH231147	VIPR_ALG4_AZQ0067	2c	E1	PI12	2016	-0.6318	0.4801	1.2032
Pestivirus B	HQ444199	VIPR_ALG4_32680714	2a	E1	Ind141353	2007	-1.0610	0.4749	1.2070
Pestivirus B	GQ888686	VIPR_ALG4_27071949	2a	E1	JZ05-1	2005	-0.9563	0.4750	1.1988
Pestivirus B	MW168422	VIPR_ALG4_QZM0693	2a	E1	YNJG2020	2020	-0.9227	0.4717	1.1724
Pestivirus B	KX170056	APT70579.1	2a	E1	V002	1999	-0.9718	0.4779	1.1932
Pestivirus B	KX170058	APT70581.1	2a	E1	V005	1999	-1.2177	0.4781	1.1691
Pestivirus B	KX170048	APT70571.1	2a	E1	V017	2003	-0.5132	0.5049	1.1851
Pestivirus B	KX170045	APT70568.1	2a	E1	V019	2004	-0.5866	0.4785	1.1968
Pestivirus B	KX170059	APT70582.1	2a	E1	V021	2005	-1.2850	0.4682	1.1664
Pestivirus B	KX170053	APT70576.1	2a	E1	V023	2006	-1.1308	0.4734	1.1797
Pestivirus B	KX170063	APT70586.1	2a	E1	V024	2006	-1.1508	0.4698	1.1688

Species according to VIPRBRC	GenBank Accession	GenBank Protein Accession	Subgenotype	Protein	Strain Name	Collection Year	SVM Patho. Score	Vaxijen Antig. Score	Averged score of EMBOSS motifs
Pestivirus B	KX170055	APT70578.1	2a	E1	V025	2006	-1.1536	0.4789	1.1705
Pestivirus B	KX170043	APT70566.1	2a	E1	V028	2006	-0.6399	0.5031	1.1899
Pestivirus B	KX170060	APT70583.1	2a	E1	V037	2008	-1.1486	0.4787	1.1664
Pestivirus B	KX170054	APT70577.1	2a	E1	V044	2008	-1.1255	0.4559	1.1807
Pestivirus B	KX170050	APT70573.1	2a	E1	V047	2009	-0.5363	0.5072	1.1899
Pestivirus B	KX170064	APT70587.1	2a	E1	V051	2010	-1.1164	0.4728	1.1789
Pestivirus B	KX170044	APT70567.1	2a	E1	V062	2005	-0.6127	0.4871	1.1800
Pestivirus B	KX170046	APT70569.1	2a	E1	V063	2005	-0.6177	0.4922	1.1899
Pestivirus B	KX170051	APT70574.1	2a	E1	V076	2011	-0.5742	0.5074	1.2023
Pestivirus B	KX170061	APT70584.1	2a	E1	V079	2010	-1.0872	0.4885	1.1414
Pestivirus B	KX170049	APT70572.1	2a	E1	V081	2009	-0.6177	0.4922	1.1899
Pestivirus B	KX170047	APT70570.1	2a	E1	V082	2008	-0.3480	0.5097	1.1851
Pestivirus B	KX170052	APT70575.1	2a	E1	V085	2007	-0.5742	0.5074	1.2023
Pestivirus B	KX170041	APT70564.1	2a	E1	V086	2006	-0.6819	0.4812	1.2023
Pestivirus B	KX170039	APT70562.1	2a	E1	V088	2005	-0.4751	0.4764	1.1930
Pestivirus B	KX170042	APT70565.1	2a	E1	V089	2004	-0.9527	0.4786	1.1968
Pestivirus B	KX170040	APT70563.1	2a	E1	V095	1999	-0.6289	0.4766	1.1968
Pestivirus B	KX170062	APT70585.1	2a	E1	V097	1998	-1.0628	0.4848	1.1716
Pestivirus B	KX170057	APT70580.1	2a	E1	V065	2006	-1.1720	0.4755	1.1705
Pestivirus B	LC016726	VIPR_ALG4_74092246	2c	E2	BVDV/Akkeshi/1170/13	2013	-0.5321	0.4823	1.1100
Pestivirus B	LC648409	VIPR_ALG4_BDB0405	2c	E2	BVDV/Akkeshi/1897/20	2020	-0.4990	0.4812	1.1105
Pestivirus B	LC648403	VIPR_ALG4_BDB0404	2c	E2	BVDV/Engaru/1308/15	2015	-0.5788	0.4692	1.1080
Pestivirus B	LC016727	VIPR_ALG4_74092246	2a	E2	BVDV/Hamanaka/843/10	2010	-0.7441	0.5161	1.1178
Pestivirus B	LC016725	VIPR_ALG4_74092245	2c	E2	BVDV/Honbetsu/597/07	2007	-0.5459	0.4640	1.1065
Pestivirus B	LC648405	VIPR_ALG4_BDB0404	2c	E2	BVDV/Monbetsu/1333/16	2016	-0.5633	0.4532	1.1077
Pestivirus B	LC648408	VIPR_ALG4_BDB0405	2c	E2	BVDV/Nakashibetsu/1682/18	2018	-0.4980	0.4768	1.1075
Pestivirus B	LC648406	VIPR_ALG4_BDB0404	2c	E2	BVDV/Nayoro/1623/18	2018	-0.5514	0.4613	1.1077
Pestivirus B	LC016724	VIPR_ALG4_74092245	2c	E2	BVDV/Okoppe/458/05	2005	-0.4859	0.4903	1.1048
Pestivirus B	LC648404	VIPR_ALG4_BDB0404	2c	E2	BVDV/Oumu/1311/15	2015	-0.4342	0.4624	1.1053
Pestivirus B	LC648407	VIPR_ALG4_BDB0404	2c	E2	BVDV/Oumu/1654/17	2017	-0.4920	0.4827	1.1064
Pestivirus B	LC648402	VIPR_ALG4_BDB0404	2c	E2	BVDV/Shihoro/1258/14	2014	-0.5001	0.4535	1.1081
Pestivirus B	LC016728	VIPR_ALG4_74092246	2a	E2	BVDV/Shikaoi/909/10	2010	-0.7423	0.5061	1.1185
Pestivirus B	LC016723	VIPR_ALG4_74092245	2c	E2	BVDV/Yuubetsu/71/01	2001	-0.4246	0.4817	1.1066
Pestivirus B	KX170138	APT70661.1	2a	E2	V002	1999	-0.4461	0.4931	1.1132
Pestivirus B	KX170137	APT70660.1	2a	E2	V005	1999	-0.4198	0.5287	1.1153
Pestivirus B	KX170128	APT70651.1	2a	E2	V017	2003	-0.7721	0.5287	1.1148
Pestivirus B	KX170142	APT70665.1	2a	E2	V021	2005	-0.4158	0.4721	1.1142
Pestivirus B	KX170132	APT70655.1	2a	E2	V023	2006	-0.5824	0.5472	1.1233
Pestivirus B	KX170135	APT70658.1	2a	E2	V024	2006	-0.4239	0.4760	1.1108
Pestivirus B	KX170133	APT70656.1	2a	E2	V025	2006	-0.4586	0.4899	1.1151
Pestivirus B	KX170118	APT70641.1	2a	E2	V028	2006	-0.5693	0.5501	1.1216
Pestivirus B	KX170141	APT70664.1	2a	E2	V037	2008	-0.4697	0.4753	1.1137
Pestivirus B	KX170134	APT70657.1	2a	E2	V044	2008	-0.5742	0.4879	1.1165
Pestivirus B	KX170129	APT70652.1	2a	E2	V047	2009	-0.7738	0.5234	1.1202
Pestivirus B	KX170136	APT70659.1	2a	E2	V051	2010	-0.4582	0.4919	1.1173
Pestivirus B	KX170124	APT70647.1	2a	E2	V062	2005	-0.8156	0.5259	1.1214

Species according to VIPRBRC	GenBank Accession	GenBank Protein Accession	Subgenotype	Protein	Strain Name	Collection Year	SVM Patho. Score	Vaxijen Antig. Score	Averged score of EMBOSS motifs
Pestivirus B	KX170125	APT70648.1	2a	E2	V063	2005	-0.6449	0.5134	1.1150
Pestivirus B	KX170130	APT70653.1	2a	E2	V076	2011	-0.6631	0.5287	1.1159
Pestivirus B	KX170143	APT70666.1	2a	E2	V079	2010	-0.5145	0.4664	1.1142
Pestivirus B	KX170123	APT70646.1	2a	E2	V081	2009	-0.7318	0.4924	1.1145
Pestivirus B	KX170126	APT70649.1	2a	E2	V082	2008	-0.7404	0.5124	1.1182
Pestivirus B	KX170131	APT70654.1	2a	E2	V085	2007	-0.7591	0.5022	1.1183
Pestivirus B	KX170119	APT70642.1	2a	E2	V086	2006	-0.6719	0.5045	1.1113
Pestivirus B	KX170121	APT70644.1	2a	E2	V088	2005	-0.7090	0.4934	1.1146
Pestivirus B	KX170120	APT70643.1	2a	E2	V089	2004	-0.6839	0.5122	1.1115
Pestivirus B	KX170122	APT70645.1	2a	E2	V095	1999	-0.6223	0.5041	1.1098
Pestivirus B	KX170140	APT70663.1	2a	E2	V097	1998	-0.3819	0.5114	1.1141
Pestivirus B	KJ146971	AHN60088.1	2a	E2	A063379B	2013	-0.4582	0.4919	1.1173
Pestivirus B	LC630480	VIPR_ALG4_BCV1984	2c	E2	BVDV/Gunma/01/19	2019	-0.5291	0.4955	1.1167
Pestivirus B	LC630477	VIPR_ALG4_BCV1983	2c	E2	BVDV/Gunma/06/18	2018	-0.4503	0.4475	1.1114
Pestivirus B	LC630481	VIPR_ALG4_BCV1984	2c	E2	BVDV/Gunma/08/19	2019	-0.5990	0.4884	1.1039
Pestivirus B	LC630478	VIPR_ALG4_BCV1983	2c	E2	BVDV/Gunma/20/18	2018	-0.3872	0.4692	1.1085
Pestivirus B	LC630479	VIPR_ALG4_BCV1983	2c	E2	BVDV/Gunma/30/18	2018	-0.6084	0.4661	1.1070
Pestivirus B	LC630484	VIPR_ALG4_BCV1984	2c	E2	BVDV/Kumamoto/05/20	2020	-0.5315	0.4542	1.1064
Pestivirus B	LC630485	VIPR_ALG4_BCV1984	2c	E2	BVDV/Kumamoto/09/20	2020	-0.5428	0.4580	1.1071
Pestivirus B	LC630486	VIPR_ALG4_BCV1984	2c	E2	BVDV/Oita/17/18	2018	-0.4257	0.4697	1.1075
Pestivirus B	LC630482	VIPR_ALG4_BCV1984	2c	E2	BVDV/Okayama/16/19	2019	-0.2980	0.4735	1.1095
Pestivirus B	LC630483	VIPR_ALG4_BCV1984	2c	E2	BVDV/Okayama/25/20	2020	-0.3870	0.4625	1.1053
Pestivirus B	JN377416	AEV54354.1	2a	E2	M10 3432	2010	-0.3738	0.5284	1.1163
Pestivirus B	JN377415	AEV54353.1	2a	E2	M10 5347	2010	-0.6067	0.5015	1.1186
Pestivirus B	JN387139	AEV76932.1	2a	E2	MS12	2006	-0.4208	0.5018	1.1138
Pestivirus B	KX170139	APT70662.1	2a	E2	V065	2006	-0.6052	0.5039	1.1201
Pestivirus B	KT875139	VIPR_ALG4_10037029	2a	E2	32W	2005	-0.4997	0.5126	1.1149
Pestivirus B	KT875134	VIPR_ALG4_10037029	2a	E2	12W	2005	-0.5163	0.5179	1.1164
Pestivirus B	KT875135	VIPR_ALG4_10037029	2a	E2	13Y	2005	-0.6586	0.5019	1.1092
Pestivirus B	KP057803	VIPR_ALG4_80287536	2a	E2	24515	1993	-0.5478	0.4862	1.1153
Pestivirus B	KT875136	VIPR_ALG4_10037029	2a	E2	27Y	2005	-0.4446	0.5135	1.1149
Pestivirus B	KT875137	VIPR_ALG4_10037029	2a	E2	29Y	2005	-0.4997	0.5126	1.1149
Pestivirus B	KT875138	VIPR_ALG4_10037029	2a	E2	2Y	2005	-0.4321	0.5250	1.1122
Pestivirus B	KT875140	VIPR_ALG4_10037029	2a	E2	34Y	2005	-0.4997	0.5126	1.1149
Pestivirus B	KT875141	VIPR_ALG4_10037029	2a	E2	36W	2005	-0.6180	0.4919	1.1092
Pestivirus B	KT875142	VIPR_ALG4_10037029	2a	E2	41Y	2005	-0.4963	0.5171	1.1128
Pestivirus B	KT875143	VIPR_ALG4_10037029	2a	E2	42W	2005	-0.4446	0.5135	1.1149
Pestivirus B	KT875144	VIPR_ALG4_10037029	2a	E2	43Y	2005	-0.5950	0.5039	1.1103
Pestivirus B	KT875145	VIPR_ALG4_10037029	2a	E2	47Y	2005	-0.5283	0.5088	1.1149
Pestivirus B	KT875146	VIPR_ALG4_10037029	2a	E2	50Y	2005	-0.6457	0.4980	1.1128
Pestivirus B	KT875147	VIPR_ALG4_10037029	2a	E2	51W	2005	-0.4446	0.5135	1.1149
Pestivirus B	KT875148	VIPR_ALG4_10037029	2a	E2	51Y	2005	-0.4852	0.5139	1.1168
Pestivirus B	KT875149	VIPR_ALG4_10037029	2a	E2	53W	2005	-0.5691	0.5135	1.1157
Pestivirus B	KT875150	VIPR_ALG4_10037029	2a	E2	58W	2005	-0.4446	0.5135	1.1149
Pestivirus B	KT875151	VIPR_ALG4_10037029	2a	E2	58Y	2005	-0.4997	0.5126	1.1149
Pestivirus B	KT875152	VIPR_ALG4_10037029	2a	E2	5Y	2005	-0.4997	0.5126	1.1149
Pestivirus B	KT875153	VIPR_ALG4_10037029	2a	E2	62Y	2005	-0.4815	0.4924	1.1125

Species according to VIPRBRC	GenBank Accession	GenBank Protein Accession	Subgenotype	Protein	Strain Name	Collection Year	SVM Patho. Score	Vaxijen Antig. Score	Averged score of EMBOSS motifs
Pestivirus B	KT875154	VIPR_ALG4_10037029	2a	E2	65Y	2005	-0.4641	0.5090	1.1168
Pestivirus B	KT875155	VIPR_ALG4_10037029	2a	E2	67Y	2005	-0.4997	0.5126	1.1149
Pestivirus B	KT875156	VIPR_ALG4_10037029	2a	E2	68W	2005	-0.6586	0.5019	1.1092
Pestivirus B	KT875157	VIPR_ALG4_10037029	2a	E2	71Y	2005	-0.4997	0.5126	1.1149
Pestivirus B	KT875158	VIPR_ALG4_10037029	2a	E2	73Y	2005	-0.6586	0.5019	1.1092
Pestivirus B	KT875159	VIPR_ALG4_10037029	2a	E2	74Y	2005	-0.4599	0.5117	1.1149
Pestivirus B	KT875160	VIPR_ALG4_10037029	2a	E2	75W	2005	-0.4855	0.5083	1.1156
Pestivirus B	KT875161	VIPR_ALG4_10037029	2a	E2	75Y	2005	-0.4997	0.5126	1.1149
Pestivirus B	KT875162	VIPR_ALG4_10037029	2a	E2	76Y	2005	-0.4446	0.5135	1.1149
Pestivirus B	KT875163	VIPR_ALG4_10037029	2a	E2	78W	2005	-0.5057	0.5066	1.1149
Pestivirus B	KT875164	VIPR_ALG4_10037029	2a	E2	79W	2005	-0.6373	0.4937	1.1092
Pestivirus B	KT875165	VIPR_ALG4_10037029	2a	E2	7W	2005	-0.4446	0.5135	1.1149
Pestivirus B	KT875166	VIPR_ALG4_10037029	2a	E2	82W	2005	-0.5057	0.5066	1.1149
Pestivirus B	KT875167	VIPR_ALG4_10037029	2a	E2	83Y	2005	-0.4738	0.5097	1.1149
Pestivirus B	KT875168	VIPR_ALG4_10037029	2a	E2	90W	2005	-0.6319	0.5023	1.1092
Pestivirus B	KT875169	VIPR_ALG4_10037030	2a	E2	91W	2005	-0.4561	0.5095	1.1149
Pestivirus B	MN824468	VIPR_ALG4_QJF12271	2a	E2	CPAE_contamination/2018	2018	-0.5451	0.5204	1.1239
Pestivirus B	KR093034	VIPR_ALG4_92904888	2a	E2	NY-93	1993	-0.5666	0.4630	1.1133
Pestivirus B	EU747875	VIPR_ALG4_19033620	2b	E2	17237	2004	-0.5276	0.4891	1.1329
Pestivirus B	MW006485	VIPR_ALG4_QPF4972	2b	E2	HEN01	2014	-0.5426	0.4827	1.1248
Pestivirus B	MH231142	VIPR_ALG4_AZQ0066	2c	E2	Parker	1991	-0.5386	0.4453	1.1060
Pestivirus B	MH806435	VIPR_ALG4_AZP5716	2a	E2	1336H	2005	-0.6858	0.4758	1.1069
Pestivirus B	MH231136	VIPR_ALG4_AZQ0066	2a	E2	JV14	1998	-0.4585	0.5030	1.1160
Pestivirus B	MK599227	VIPR_ALG4_QEU5262	2a	E2	SD-1	2016	-0.3535	0.4910	1.1115
Pestivirus B	HG426488	VIPR_ALG4_61932575	2c	E2	NRW 19-13-1_Dup(+)	2013	-0.5650	0.4598	1.1052
Pestivirus B	HG426487	VIPR_ALG4_61932574	2c	E2	NRW 19-13-1_Dup(-)	2013	-0.5650	0.4598	1.1052
Pestivirus B	MH231138	VIPR_ALG4_AZQ0066	2a	E2	MnFetus	1991	-0.4784	0.4645	1.1149
Pestivirus B	MH231131	VIPR_ALG4_AZQ0065	2a	E2	AU501	2006	-0.4379	0.5040	1.1142
Pestivirus B	MH806434	VIPR_ALG4_AZP5716	2a	E2	125c	1990	-0.4645	0.4693	1.1106
Pestivirus B	MH231151	VIPR_ALG4_AZQ0067	2e	E2	14622	2005	-0.2022	0.5130	1.1150
Pestivirus B	MH231152	VIPR_ALG4_AZQ0067	2e	E2	2412	1989	-0.2334	0.5288	1.1163
Pestivirus B	MH231127	VIPR_ALG4_AZQ0065	2a	E2	53637c	2004	-0.4719	0.4909	1.1173
Pestivirus B	MH231149	VIPR_ALG4_AZQ0067	2e	E2	Short	1989	-0.2022	0.5130	1.1150
Pestivirus B	MH231129	VIPR_ALG4_AZQ0065	2a	E2	5912c	1995	-0.4335	0.4929	1.1172
Pestivirus B	KJ000672	VIPR_ALG4_59423572	2b	E2	SD1301	2012	-0.4992	0.5007	1.1248
Pestivirus B	MH231148	VIPR_ALG4_AZQ0067	2e	E2	12-149150	2012	-0.1606	0.5487	1.1229
Pestivirus B	MH231150	VIPR_ALG4_AZQ0067	2e	E2	12-151955-317	2012	-0.3352	0.5145	1.1238
Pestivirus B	MH231133	VIPR_ALG4_AZQ0065	2e	E2	B69519c	2006	-0.2334	0.5288	1.1163
Pestivirus B	HG426495	VIPR_ALG4_61932576	2c	E2	VOE 4407	2007	-0.5625	0.4499	1.1040
Pestivirus B	HG426480	VIPR_ALG4_61932573	2c	E2	D37-13-2_Dup(+)	2013	-0.5111	0.4675	1.1052
Pestivirus B	HG426482	VIPR_ALG4_61932573	2c	E2	D75-13-609_Dup(+)	2013	-0.5111	0.4675	1.1052
Pestivirus B	HG426486	VIPR_ALG4_61932574	2c	E2	NRW 14-13_Dup(+)	2013	-0.6206	0.4569	1.1084
Pestivirus B	HG426491	VIPR_ALG4_61932575	2c	E2	Potsdam 1600	2000	-0.5693	0.4447	1.1144
Pestivirus B	MH231141	VIPR_ALG4_AZQ0066	2a	E2	PI28	2016	-0.3134	0.4840	1.1213
Pestivirus B	HG426494	VIPR_ALG4_61932576	2c	E2	SH2210-23	2010	-0.4670	0.4672	1.1134
Pestivirus B	MH806436	VIPR_ALG4_AZP5716	2a	E2	296c	1995	-0.5031	0.4944	1.1110
Pestivirus B	MH231137	VIPR_ALG4_AZQ0066	2a	E2	MadSpl	1991	-0.3628	0.4993	1.1135

Species according to VIPRBRC	GenBank Accession	GenBank Protein Accession	Subgenotype	Protein	Strain Name	Collection Year	SVM Patho. Score	Vaxijen Antig. Score	Averged score of EMBOSS motifs
Pestivirus B	KP941585	VIPR_ALG4_80092432	2a	E2	USMARC-55476	2014	-0.4867	0.4912	1.1218
Pestivirus B	KT832820	VIPR_ALG4_99822633	2a	E2	USMARC-60767	2014	-0.4220	0.4931	1.1119
Pestivirus B	MH231134	VIPR_ALG4_AZQ0065	2a	E2	B9497	1997	-0.6179	0.5035	1.1176
Pestivirus B	MN527354	VIPR_ALG4_QLH0204	2a	E2	GS2018	2018	-0.6343	0.5358	1.1147
Pestivirus B	MH231144	VIPR_ALG4_AZQ0066	2a	E2	Sanderson6319	1992	-0.5188	0.4673	1.1211
Pestivirus B	HG426493	VIPR_ALG4_61932576	2c	E2	SH2210-17	2010	-0.5842	0.4555	1.1137
Pestivirus B	KT832817	VIPR_ALG4_99822632	2a	E2	USMARC-60764	2014	-0.5208	0.5244	1.1127
Pestivirus B	KT832822	VIPR_ALG4_99822633	2a	E2	USMARC-60779	2014	-0.4493	0.5028	1.1116
Pestivirus B	MH231125	VIPR_ALG4_AZQ0064	2a	E2	2139	1992	-0.3794	0.5033	1.1153
Pestivirus B	KP941582	VIPR_ALG4_80092431	2c	E2	USMARC-53873	2014	-0.4841	0.4308	1.1094
Pestivirus B	KT832819	VIPR_ALG4_99822632	2a	E2	USMARC-60766	2014	-0.5688	0.4542	1.1079
Pestivirus B	KT832821	VIPR_ALG4_99822633	2c	E2	USMARC-60768	2014	-0.5185	0.4598	1.1096
Pestivirus B	HG426479	VIPR_ALG4_61932573	2c	E2	D37-13-2_Dup(-)	2013	-0.5111	0.4675	1.1052
Pestivirus B	HG426481	VIPR_ALG4_61932573	2c	E2	D75-13-609_Dup(-)	2013	-0.5111	0.4675	1.1052
Pestivirus B	MH231123	VIPR_ALG4_AZQ0064	2a	E2	10406	1993	-0.6209	0.5038	1.1165
Pestivirus B	HG426492	VIPR_ALG4_61932575	2c	E2	SH2210-14	2010	-0.6399	0.4606	1.1147
Pestivirus B	KT832823	VIPR_ALG4_99822633	2a	E2	USMARC-60780	2014	-0.4933	0.4823	1.1100
Pestivirus B	HG426485	VIPR_ALG4_61932574	2c	E2	NRW 14-13_Dup(-)	2013	-0.6206	0.4569	1.1084
Pestivirus B	MH231126	VIPR_ALG4_AZQ0065	2b	E2	3237	1990	-0.5314	0.4968	1.1249
Pestivirus B	MH806438	VIPR_ALG4_AZP5716	2a	E2	McCart_c	1989	-0.5497	0.5646	1.1175
Pestivirus B	KT832818	VIPR_ALG4_99822632	2a	E2	USMARC-60765	2014	-0.7090	0.5049	1.1197
Pestivirus B	MH231124	VIPR_ALG4_AZQ0064	2e	E2	1786c	1989	-0.2183	0.5305	1.1208
Pestivirus B	HG426483	VIPR_ALG4_61932574	2c	E2	NRW 12-13_Dup(-)	2013	-0.5779	0.4606	1.1052
Pestivirus B	HG426484	VIPR_ALG4_61932574	2c	E2	NRW 12-13_Dup(+)	2013	-0.5779	0.4606	1.1052
Pestivirus B	HG426489	VIPR_ALG4_61932575	2c	E2	NRW 19-13-8_Dup(-)	2013	-0.5111	0.4675	1.1052
Pestivirus B	HG426490	VIPR_ALG4_61932575	2c	E2	NRW 19-13-8_Dup(+)	2013	-0.5111	0.4675	1.1052
Pestivirus B	MH231130	VIPR_ALG4_AZQ0065	2a	E2	95-1501	1998	-0.5244	0.5508	1.1175
Pestivirus B	MH231132	VIPR_ALG4_AZQ0065	2a	E2	AzSpl	1997	-0.4218	0.5093	1.1174
Pestivirus B	MH231128	VIPR_ALG4_AZQ0065	2a	E2	570152	1992	-0.5254	0.5033	1.1122
Pestivirus B	MH231135	VIPR_ALG4_AZQ0065	2a	E2	BV1907	1995	-0.4528	0.4818	1.1132
Pestivirus B	JF714967	VIPR_ALG4_34657797	2a	E2	HLJ-10	2011	-0.6149	0.4396	1.1086
Pestivirus B	MH231139	VIPR_ALG4_AZQ0066	2a	E2	Olwein #12	1990	-0.2978	0.4871	1.1067
Pestivirus B	MH231140	VIPR_ALG4_AZQ0066	2a	E2	PA	1992	-0.4396	0.4806	1.1156
Pestivirus B	MH231143	VIPR_ALG4_AZQ0066	2a	E2	RS886	2014	-0.7249	0.4590	1.1111
Pestivirus B	HQ258810	VIPR_ALG4_31199027	2a	E2	SH-28	2009	-0.3979	0.4465	1.1169
Pestivirus B	MH231145	VIPR_ALG4_AZQ0066	2a	E2	Victor301	1990	-0.4233	0.5114	1.1177
Pestivirus B	MH231146	VIPR_ALG4_AZQ0067	2a	E2	WiscA	1991	-0.2530	0.5065	1.1159
Pestivirus B	FJ527854	VIPR_ALG4_22961018	2a	E2	XJ-04	2004	-0.4811	0.4942	1.1146
Pestivirus B	KC963968	VIPR_ALG4_53029119	2a	E2	11F011	2011	-0.7418	0.4809	1.1181
Pestivirus B	MH806437	VIPR_ALG4_AZP5716	2a	E2	9231	2004	-0.7513	0.4632	1.1145
Pestivirus B	MG879027	VIPR_ALG4_AVA3071	2a	E2	CN10.2015.821	2014	-0.7445	0.5114	1.1182
Pestivirus B	KX096718	VIPR_ALG4_11130170	2a	E2	HB-1511	2015	-0.6480	0.4855	1.1179
Pestivirus B	LC649064	VIPR_ALG4_BDB0736	2c	E2	KZ-91-NCP	1991	-0.5969	0.4797	1.1088
Pestivirus B	MH231147	VIPR_ALG4_AZQ0067	2c	E2	PI12	2016	-0.5108	0.4536	1.1062
Pestivirus B	HQ444199	VIPR_ALG4_32680714	2a	E2	Ind141353	2007	-0.3393	0.4740	1.1109
Pestivirus B	GQ888686	VIPR_ALG4_27071949	2a	E2	JZ05-1	2005	-0.4744	0.4935	1.1145
Pestivirus B	MW168422	VIPR_ALG4_QZM0693	2a	E2	YNJG2020	2020	-0.5153	0.5130	1.1128

Species according to VIPRBRC	GenBank Accession	GenBank Protein Accession	Subgenotype	Protein	Strain Name	Collection Year	SVM Patho. Score	Vaxijen Antig. Score	Averged score of EMBOSS motifs
Pestivirus B	KT875139	VIPR_ALG4_10037029	2a	P7	32W	2005	-0.5316	0.1796	1.1620
Pestivirus B	KT875134	VIPR_ALG4_10037029	2a	P7	12W	2005	-0.5316	0.1796	1.1620
Pestivirus B	KT875135	VIPR_ALG4_10037029	2a	P7	13Y	2005	-0.5316	0.1796	1.1620
Pestivirus B	KP057803	VIPR_ALG4_80287536	2a	P7	24515	1993	-0.1360	0.2366	1.1730
Pestivirus B	KT875136	VIPR_ALG4_10037029	2a	P7	27Y	2005	-0.5316	0.1796	1.1620
Pestivirus B	KT875137	VIPR_ALG4_10037029	2a	P7	29Y	2005	-0.5316	0.1796	1.1620
Pestivirus B	KT875138	VIPR_ALG4_10037029	2a	P7	2Y	2005	-0.5316	0.1796	1.1620
Pestivirus B	KT875140	VIPR_ALG4_10037029	2a	P7	34Y	2005	-0.5316	0.1796	1.1620
Pestivirus B	KT875141	VIPR_ALG4_10037029	2a	P7	36W	2005	-0.5316	0.1796	1.1620
Pestivirus B	KT875142	VIPR_ALG4_10037029	2a	P7	41Y	2005	-0.5316	0.1796	1.1620
Pestivirus B	KT875143	VIPR_ALG4_10037029	2a	P7	42W	2005	-0.5316	0.1796	1.1620
Pestivirus B	KT875144	VIPR_ALG4_10037029	2a	P7	43Y	2005	-0.5316	0.1796	1.1620
Pestivirus B	KT875145	VIPR_ALG4_10037029	2a	P7	47Y	2005	-0.5316	0.1796	1.1620
Pestivirus B	KT875146	VIPR_ALG4_10037029	2a	P7	50Y	2005	-0.5316	0.1796	1.1620
Pestivirus B	KT875147	VIPR_ALG4_10037029	2a	P7	51W	2005	-0.5316	0.1796	1.1620
Pestivirus B	KT875148	VIPR_ALG4_10037029	2a	P7	51Y	2005	-0.4638	0.2241	1.1620
Pestivirus B	KT875149	VIPR_ALG4_10037029	2a	P7	53W	2005	-0.5316	0.1796	1.1620
Pestivirus B	KT875150	VIPR_ALG4_10037029	2a	P7	58W	2005	-0.5316	0.1796	1.1620
Pestivirus B	KT875151	VIPR_ALG4_10037029	2a	P7	58Y	2005	-0.2112	0.1848	1.1620
Pestivirus B	KT875152	VIPR_ALG4_10037029	2a	P7	5Y	2005	-0.5316	0.1796	1.1620
Pestivirus B	KT875153	VIPR_ALG4_10037029	2a	P7	62Y	2005	-0.5316	0.1796	1.1620
Pestivirus B	KT875154	VIPR_ALG4_10037029	2a	P7	65Y	2005	-0.5316	0.1796	1.1620
Pestivirus B	KT875155	VIPR_ALG4_10037029	2a	P7	67Y	2005	-0.5316	0.1796	1.1620
Pestivirus B	KT875156	VIPR_ALG4_10037029	2a	P7	68W	2005	-0.5316	0.1796	1.1620
Pestivirus B	KT875157	VIPR_ALG4_10037029	2a	P7	71Y	2005	-0.5316	0.1796	1.1620
Pestivirus B	KT875158	VIPR_ALG4_10037029	2a	P7	73Y	2005	-0.5316	0.1796	1.1620
Pestivirus B	KT875159	VIPR_ALG4_10037029	2a	P7	74Y	2005	-0.5316	0.1796	1.1620
Pestivirus B	KT875160	VIPR_ALG4_10037029	2a	P7	75W	2005	-0.5316	0.1796	1.1620
Pestivirus B	KT875161	VIPR_ALG4_10037029	2a	P7	75Y	2005	-0.5316	0.1796	1.1620
Pestivirus B	KT875162	VIPR_ALG4_10037029	2a	P7	76Y	2005	-0.5316	0.1796	1.1620
Pestivirus B	KT875163	VIPR_ALG4_10037029	2a	P7	78W	2005	-0.5299	0.2338	1.1620
Pestivirus B	KT875164	VIPR_ALG4_10037029	2a	P7	79W	2005	-0.5316	0.1796	1.1620
Pestivirus B	KT875165	VIPR_ALG4_10037029	2a	P7	7W	2005	-0.5316	0.1796	1.1620
Pestivirus B	KT875166	VIPR_ALG4_10037029	2a	P7	82W	2005	-0.5316	0.1796	1.1620
Pestivirus B	KT875167	VIPR_ALG4_10037029	2a	P7	83Y	2005	-0.5316	0.1796	1.1620
Pestivirus B	KT875168	VIPR_ALG4_10037029	2a	P7	90W	2005	-0.5316	0.1796	1.1620
Pestivirus B	KT875169	VIPR_ALG4_10037030	2a	P7	91W	2005	-0.5316	0.1796	1.1620
Pestivirus B	MN824468	VIPR_ALG4_QJF1227	2a	P7	CPAE_contamination/2018	2018	-0.0504	0.2493	1.2065
Pestivirus B	KR093034	VIPR_ALG4_92904888	2a	P7	NY-93	1993	-0.2052	0.2897	1.1900
Pestivirus B	MW006485	VIPR_ALG4_QPF4972	2b	P7	HEN01	2014	0.0282	0.2472	1.1757
Pestivirus B	MH231142	VIPR_ALG4_AZQ0066	2c	P7	Parker	1991	0.6042	0.2558	1.2490
Pestivirus B	MH806435	VIPR_ALG4_AZP5716	2a	P7	1336H	2005	-0.0713	0.1734	1.2065
Pestivirus B	MH231136	VIPR_ALG4_AZQ0066	2a	P7	JV14	1998	-0.0030	0.2358	1.2065
Pestivirus B	MK599227	VIPR_ALG4_QEU5262	2a	P7	SD-1	2016	0.2109	0.1420	1.2137
Pestivirus B	HG426488	VIPR_ALG4_61932575	2c	P7	NRW 19-13-1_Dup(+)	2013	0.7514	0.2575	1.2490
Pestivirus B	HG426487	VIPR_ALG4_61932574	2c	P7	NRW 19-13-1_Dup(-)	2013	0.7514	0.2575	1.2490
Pestivirus B	MH231138	VIPR_ALG4_AZQ0066	2a	P7	MnFetus	1991	-0.4043	0.2514	1.1915

Species according to VIPRBRC	GenBank Accession	GenBank Protein Accession	Subgenotype	Protein	Strain Name	Collection Year	SVM Patho. Score	Vaxijen Antig. Score	Averged score of EMBOSS motifs
Pestivirus B	MH231131	VIPR_ALG4_AZQ0065	2a	P7	AU501	2006	-0.7254	0.2435	1.1620
Pestivirus B	MH806434	VIPR_ALG4_AZP5716	2a	P7	125c	1990	0.9450	0.2160	1.2065
Pestivirus B	MH231151	VIPR_ALG4_AZQ0067	2e	P7	14622	2005	-0.5101	0.3529	1.1870
Pestivirus B	MH231152	VIPR_ALG4_AZQ0067	2e	P7	2412	1989	-0.5101	0.3529	1.1870
Pestivirus B	MH231127	VIPR_ALG4_AZQ0065	2a	P7	53637c	2004	-0.6150	0.2707	1.2065
Pestivirus B	MH231149	VIPR_ALG4_AZQ0067	2e	P7	Short	1989	-0.5101	0.3529	1.1870
Pestivirus B	MH231129	VIPR_ALG4_AZQ0065	2a	P7	5912c	1995	0.1203	0.2855	1.2065
Pestivirus B	KJ000672	VIPR_ALG4_59423572	2b	P7	SD1301	2012	-0.5072	0.2708	1.1757
Pestivirus B	MH231148	VIPR_ALG4_AZQ0067	2e	P7	12-149150	2012	-0.5101	0.3529	1.1870
Pestivirus B	MH231150	VIPR_ALG4_AZQ0067	2e	P7	12-151955-317	2012	-0.5101	0.3529	1.1870
Pestivirus B	MH231133	VIPR_ALG4_AZQ0065	2e	P7	B69519c	2006	-0.5101	0.3529	1.1870
Pestivirus B	HG426495	VIPR_ALG4_61932576	2c	P7	VOE 4407	2007	0.4188	0.2402	1.2380
Pestivirus B	HG426480	VIPR_ALG4_61932573	2c	P7	D37-13-2_Dup(+)	2013	0.7514	0.2575	1.2490
Pestivirus B	HG426482	VIPR_ALG4_61932573	2c	P7	D75-13-609_Dup(+)	2013	0.7514	0.2575	1.2490
Pestivirus B	HG426486	VIPR_ALG4_61932574	2c	P7	NRW 14-13_Dup(+)	2013	0.7514	0.2575	1.2490
Pestivirus B	HG426491	VIPR_ALG4_61932575	2c	P7	Potsdam 1600	2000	0.4188	0.2402	1.2380
Pestivirus B	MH231141	VIPR_ALG4_AZQ0066	2a	P7	PI28	2016	-0.5533	0.2449	1.2065
Pestivirus B	HG426494	VIPR_ALG4_61932576	2c	P7	SH2210-23	2010	0.3980	0.2432	1.2050
Pestivirus B	MH806436	VIPR_ALG4_AZP5716	2a	P7	296c	1995	0.5086	0.2753	1.2065
Pestivirus B	MH231137	VIPR_ALG4_AZQ0066	2a	P7	MadSpl	1991	-0.0030	0.2358	1.2065
Pestivirus B	KP941585	VIPR_ALG4_80092432	2a	P7	USMARC-55476	2014	-0.6983	0.3590	1.2283
Pestivirus B	KT832820	VIPR_ALG4_99822633	2a	P7	USMARC-60767	2014	-0.5533	0.2449	1.2065
Pestivirus B	MH231134	VIPR_ALG4_AZQ0065	2a	P7	B9497	1997	0.0711	0.1597	1.1620
Pestivirus B	MN527354	VIPR_ALG4_QLH0204	2a	P7	GS2018	2018	-0.4637	0.1621	1.1620
Pestivirus B	MH231144	VIPR_ALG4_AZQ0066	2a	P7	Sanderson6319	1992	-0.2465	0.2255	1.2065
Pestivirus B	HG426493	VIPR_ALG4_61932576	2c	P7	SH2210-17	2010	0.0154	0.2374	1.2333
Pestivirus B	KT832817	VIPR_ALG4_99822632	2a	P7	USMARC-60764	2014	-0.6447	0.1594	1.1620
Pestivirus B	KT832822	VIPR_ALG4_99822633	2a	P7	USMARC-60779	2014	0.1829	0.1765	1.2065
Pestivirus B	MH231125	VIPR_ALG4_AZQ0064	2a	P7	2139	1992	-0.0030	0.2358	1.2065
Pestivirus B	KP941582	VIPR_ALG4_80092431	2c	P7	USMARC-53873	2014	0.9566	0.1628	1.2380
Pestivirus B	KT832819	VIPR_ALG4_99822632	2a	P7	USMARC-60766	2014	-0.0030	0.2358	1.2065
Pestivirus B	KT832821	VIPR_ALG4_99822633	2c	P7	USMARC-60768	2014	0.6600	0.1803	1.2227
Pestivirus B	HG426479	VIPR_ALG4_61932573	2c	P7	D37-13-2_Dup(-)	2013	-0.0121	0.3589	1.2263
Pestivirus B	HG426481	VIPR_ALG4_61932573	2c	P7	D75-13-609_Dup(-)	2013	-0.2269	0.3603	1.2113
Pestivirus B	MH231123	VIPR_ALG4_AZQ0064	2a	P7	10406	1993	0.0546	0.2180	1.2065
Pestivirus B	HG426492	VIPR_ALG4_61932575	2c	P7	SH2210-14	2010	0.6844	0.2350	1.2333
Pestivirus B	KT832823	VIPR_ALG4_99822633	2a	P7	USMARC-60780	2014	1.0519	0.2023	1.2065
Pestivirus B	HG426485	VIPR_ALG4_61932574	2c	P7	NRW 14-13_Dup(-)	2013	-0.0121	0.3589	1.2263
Pestivirus B	MH231126	VIPR_ALG4_AZQ0065	2b	P7	3237	1990	0.7135	0.3186	1.2485
Pestivirus B	MH806438	VIPR_ALG4_AZP5716	2a	P7	McCart_c	1989	0.0687	0.2404	1.2065
Pestivirus B	KT832818	VIPR_ALG4_99822632	2a	P7	USMARC-60765	2014	0.7749	0.1575	1.2720
Pestivirus B	MH231124	VIPR_ALG4_AZQ0064	2e	P7	1786c	1989	-0.5101	0.3529	1.1870
Pestivirus B	HG426483	VIPR_ALG4_61932574	2c	P7	NRW 12-13_Dup(-)	2013	-0.0121	0.3589	1.2263
Pestivirus B	HG426484	VIPR_ALG4_61932574	2c	P7	NRW 12-13_Dup(+)	2013	0.7514	0.2575	1.2490
Pestivirus B	HG426489	VIPR_ALG4_61932575	2c	P7	NRW 19-13-8_Dup(-)	2013	-0.0121	0.3589	1.2263
Pestivirus B	HG426490	VIPR_ALG4_61932575	2c	P7	NRW 19-13-8_Dup(+)	2013	0.7514	0.2575	1.2490
Pestivirus B	MH231130	VIPR_ALG4_AZQ0065	2a	P7	95-1501	1998	0.0687	0.2404	1.2065

Species according to VIPRBRC	GenBank Accession	GenBank Protein Accession	Subgenotype	Protein	Strain Name	Collection Year	SVM Patho. Score	Vaxijen Antig. Score	Averged score of EMBOSS motifs
Pestivirus B	MH231132	VIPR_ALG4_AZQ0065	2a	P7	AzSpl	1997	0.5174	0.2153	1.2065
Pestivirus B	MH231128	VIPR_ALG4_AZQ0065	2a	P7	570152	1992	-0.0030	0.2358	1.2065
Pestivirus B	MH231135	VIPR_ALG4_AZQ0065	2a	P7	BV1907	1995	0.2211	0.2536	1.2230
Pestivirus B	JF714967	VIPR_ALG4_34657797	2a	P7	HLJ-10	2011	1.0155	0.1129	1.2137
Pestivirus B	MH231139	VIPR_ALG4_AZQ0066	2a	P7	Olwein #12	1990	0.7707	0.2177	1.2065
Pestivirus B	MH231140	VIPR_ALG4_AZQ0066	2a	P7	PA	1992	-0.2465	0.2255	1.2065
Pestivirus B	MH231143	VIPR_ALG4_AZQ0066	2a	P7	RS886	2014	0.0687	0.2404	1.2065
Pestivirus B	HQ258810	VIPR_ALG4_31199027	2a	P7	SH-28	2009	0.5121	0.1738	1.2720
Pestivirus B	MH231145	VIPR_ALG4_AZQ0066	2a	P7	Victor301	1990	-0.6303	0.2467	1.2065
Pestivirus B	MH231146	VIPR_ALG4_AZQ0067	2a	P7	WiscA	1991	-0.1360	0.2366	1.1730
Pestivirus B	FJ527854	VIPR_ALG4_22961018	2a	P7	XJ-04	2004	-0.1177	0.1601	1.2065
Pestivirus B	KC963968	VIPR_ALG4_53029119	2a	P7	11F011	2011	-0.4043	0.2514	1.1915
Pestivirus B	MH806437	VIPR_ALG4_AZP5716	2a	P7	9231	2004	-1.0580	0.1884	1.1620
Pestivirus B	MG879027	VIPR_ALG4_AVA3071	2a	P7	CN10.2015.821	2014	-1.0580	0.1884	1.1620
Pestivirus B	KX096718	VIPR_ALG4_11130170	2a	P7	HB-1511	2015	0.0457	0.1785	1.2065
Pestivirus B	LC649064	VIPR_ALG4_BDB0736	2c	P7	KZ-91-NCP	1991	0.8933	0.2222	1.2380
Pestivirus B	MH231147	VIPR_ALG4_AZQ0067	2c	P7	PI12	2016	1.3468	0.1788	1.2380
Pestivirus B	HQ444199	VIPR_ALG4_32680714	2a	P7	Ind141353	2007	0.6158	0.2766	1.2065
Pestivirus B	GQ888686	VIPR_ALG4_27071949	2a	P7	JZ05-1	2005	0.6924	0.1761	1.2470
Pestivirus B	MW168422	VIPR_ALG4_QZM0693	2a	P7	YNJG2020	2020	-1.1383	0.1657	1.2065
Pestivirus B	KX170419	VIPR_ALG4_11298801	2a	NS3	V028	2006	-0.5219	0.5907	1.1185
Pestivirus B	KX170420	VIPR_ALG4_11298801	2a	NS3	V047	2009	-0.5295	0.5889	1.1182
Pestivirus B	KX170421	VIPR_ALG4_11298801	2a	NS3	V085	2007	-0.5295	0.5889	1.1182
Pestivirus B	KX170417	VIPR_ALG4_11298801	2a	NS3	V088	2005	-0.5219	0.5907	1.1185
Pestivirus B	KX170418	VIPR_ALG4_11298801	2a	NS3	V089	2004	-0.5219	0.5907	1.1185
Pestivirus B	KT875139	VIPR_ALG4_10037029	2a	NS3	32W	2005	-0.5314	0.5968	1.1185
Pestivirus B	KT875134	VIPR_ALG4_10037029	2a	NS3	12W	2005	-0.5314	0.5968	1.1185
Pestivirus B	KT875135	VIPR_ALG4_10037029	2a	NS3	13Y	2005	-0.5049	0.6005	1.1185
Pestivirus B	KT875136	VIPR_ALG4_10037029	2a	NS3	27Y	2005	-0.5314	0.5968	1.1185
Pestivirus B	KT875137	VIPR_ALG4_10037029	2a	NS3	29Y	2005	-0.5314	0.5968	1.1185
Pestivirus B	KT875138	VIPR_ALG4_10037029	2a	NS3	2Y	2005	-0.5314	0.5968	1.1185
Pestivirus B	KT875140	VIPR_ALG4_10037029	2a	NS3	34Y	2005	-0.5314	0.5968	1.1185
Pestivirus B	KT875141	VIPR_ALG4_10037029	2a	NS3	36W	2005	-0.5314	0.5968	1.1185
Pestivirus B	KT875142	VIPR_ALG4_10037029	2a	NS3	41Y	2005	-0.5314	0.5968	1.1185
Pestivirus B	KT875143	VIPR_ALG4_10037029	2a	NS3	42W	2005	-0.5314	0.5968	1.1185
Pestivirus B	KT875144	VIPR_ALG4_10037029	2a	NS3	43Y	2005	-0.5314	0.5968	1.1185
Pestivirus B	KT875145	VIPR_ALG4_10037029	2a	NS3	47Y	2005	-0.5314	0.5968	1.1185
Pestivirus B	KT875146	VIPR_ALG4_10037029	2a	NS3	50Y	2005	-0.5314	0.5968	1.1185
Pestivirus B	KT875147	VIPR_ALG4_10037029	2a	NS3	51W	2005	-0.5314	0.5968	1.1185
Pestivirus B	KT875148	VIPR_ALG4_10037029	2a	NS3	51Y	2005	-0.5314	0.5968	1.1185
Pestivirus B	KT875149	VIPR_ALG4_10037029	2a	NS3	53W	2005	-0.5314	0.5968	1.1185
Pestivirus B	KT875150	VIPR_ALG4_10037029	2a	NS3	58W	2005	-0.5314	0.5968	1.1185
Pestivirus B	KT875151	VIPR_ALG4_10037029	2a	NS3	58Y	2005	-0.5314	0.5968	1.1185
Pestivirus B	KT875152	VIPR_ALG4_10037029	2a	NS3	5Y	2005	-0.5314	0.5968	1.1185
Pestivirus B	KT875153	VIPR_ALG4_10037029	2a	NS3	62Y	2005	-0.5225	0.5973	1.1185
Pestivirus B	KT875154	VIPR_ALG4_10037029	2a	NS3	65Y	2005	-0.5314	0.5968	1.1185
Pestivirus B	KT875155	VIPR_ALG4_10037029	2a	NS3	67Y	2005	-0.5314	0.5968	1.1185

Species according to VIPRBRC	GenBank Accession	GenBank Protein Accession	Subgenotype	Protein	Strain Name	Collection Year	SVM Patho. Score	Vaxijen Antig. Score	Averged score of EMBOSS motifs
Pestivirus B	KT875156	VIPR_ALG4_10037029	2a	NS3	68W	2005	-0.5049	0.6005	1.1185
Pestivirus B	KT875157	VIPR_ALG4_10037029	2a	NS3	71Y	2005	-0.5314	0.5968	1.1185
Pestivirus B	KT875158	VIPR_ALG4_10037029	2a	NS3	73Y	2005	-0.5314	0.5968	1.1185
Pestivirus B	KT875159	VIPR_ALG4_10037029	2a	NS3	74Y	2005	-0.5314	0.5968	1.1185
Pestivirus B	KT875160	VIPR_ALG4_10037029	2a	NS3	75W	2005	-0.5314	0.5968	1.1185
Pestivirus B	KT875161	VIPR_ALG4_10037029	2a	NS3	75Y	2005	-0.5314	0.5968	1.1185
Pestivirus B	KT875162	VIPR_ALG4_10037029	2a	NS3	76Y	2005	-0.5314	0.5968	1.1185
Pestivirus B	KT875163	VIPR_ALG4_10037029	2a	NS3	78W	2005	-0.5314	0.5968	1.1185
Pestivirus B	KT875164	VIPR_ALG4_10037029	2a	NS3	79W	2005	-0.5314	0.5968	1.1185
Pestivirus B	KT875165	VIPR_ALG4_10037029	2a	NS3	7W	2005	-0.5314	0.5968	1.1185
Pestivirus B	KT875166	VIPR_ALG4_10037029	2a	NS3	82W	2005	-0.5314	0.5968	1.1185
Pestivirus B	KT875167	VIPR_ALG4_10037029	2a	NS3	83Y	2005	-0.5314	0.5968	1.1185
Pestivirus B	KT875168	VIPR_ALG4_10037029	2a	NS3	90W	2005	-0.5314	0.5968	1.1185
Pestivirus B	KT875169	VIPR_ALG4_10037030	2a	NS3	91W	2005	-0.5314	0.5968	1.1185
Pestivirus B	KP057803	VIPR_ALG4_80287536	2a	NS3	24515	1993	-0.5191	0.5896	1.1185
Pestivirus B	KR093034	VIPR_ALG4_92904888	2a	NS3	NY-93	1993	-0.5191	0.5896	1.1185
Pestivirus B	MW006485	VIPR_ALG4_QPF4972	2b	NS3	HEN01	2014	-0.5891	0.5942	1.1193
Pestivirus B	MH231142	VIPR_ALG4_AZQ0066	2c	NS3	Parker	1991	-0.4926	0.5911	1.1198
Pestivirus B	MH806435	VIPR_ALG4_AZP5716	2a	NS3	1336H	2005	-0.4805	0.5903	1.1198
Pestivirus B	MH231136	VIPR_ALG4_AZQ0066	2a	NS3	JV14	1998	-0.4841	0.5909	1.1185
Pestivirus B	MK599227	VIPR_ALG4_QEU5262	2a	NS3	SD-1	2016	-0.4912	0.5829	1.1188
Pestivirus B	HG426487	VIPR_ALG4_61932574	2c	NS3	NRW 19-13-1_Dup(-)	2013	-0.5138	0.5892	1.1223
Pestivirus B	MH231138	VIPR_ALG4_AZQ0066	2a	NS3	MnFetus	1991	-0.5191	0.5896	1.1185
Pestivirus B	MH231131	VIPR_ALG4_AZQ0065	2a	NS3	AU501	2006	-0.5224	0.5917	1.1185
Pestivirus B	MH231151	VIPR_ALG4_AZQ0067	2e	NS3	14622	2005	-0.4975	0.5954	1.1193
Pestivirus B	MH231152	VIPR_ALG4_AZQ0067	2e	NS3	2412	1989	-0.4829	0.5934	1.1203
Pestivirus B	MH231149	VIPR_ALG4_AZQ0067	2e	NS3	Short	1989	-0.4975	0.5954	1.1193
Pestivirus B	KJ000672	VIPR_ALG4_59423572	2b	NS3	SD1301	2012	-0.6317	0.5837	1.1200
Pestivirus B	MH231148	VIPR_ALG4_AZQ0067	2e	NS3	12-149150	2012	-0.4784	0.5999	1.1193
Pestivirus B	MH231150	VIPR_ALG4_AZQ0067	2e	NS3	12-151955-317	2012	-0.5012	0.6004	1.1205
Pestivirus B	HG426495	VIPR_ALG4_61932576	2c	NS3	VOE 4407	2007	-0.4926	0.5911	1.1198
Pestivirus B	HG426491	VIPR_ALG4_61932575	2c	NS3	Potsdam 1600	2000	-0.4863	0.5912	1.1198
Pestivirus B	MH231141	VIPR_ALG4_AZQ0066	2a	NS3	PI28	2016	-0.5239	0.5856	1.1184
Pestivirus B	HG426494	VIPR_ALG4_61932576	2c	NS3	SH2210-23	2010	-0.4978	0.5984	1.1188
Pestivirus B	MH231137	VIPR_ALG4_AZQ0066	2a	NS3	MadSpl	1991	-0.5359	0.5901	1.1185
Pestivirus B	KP941585	VIPR_ALG4_80092432	2a	NS3	USMARC-55476	2014	-0.4805	0.5971	1.1185
Pestivirus B	KT832820	VIPR_ALG4_99822633	2a	NS3	USMARC-60767	2014	-0.5359	0.5901	1.1185
Pestivirus B	MH231134	VIPR_ALG4_AZQ0065	2a	NS3	B9497	1997	-0.5219	0.5907	1.1185
Pestivirus B	MN527354	VIPR_ALG4_QLH0204	2a	NS3	GS2018	2018	-0.5540	0.5987	1.1159
Pestivirus B	MH231144	VIPR_ALG4_AZQ0066	2a	NS3	Sanderson6319	1992	-0.5418	0.5862	1.1185
Pestivirus B	HG426493	VIPR_ALG4_61932576	2c	NS3	SH2210-17	2010	-0.4952	0.5868	1.1226
Pestivirus B	KT832817	VIPR_ALG4_99822632	2a	NS3	USMARC-60764	2014	-0.5191	0.5896	1.1185
Pestivirus B	KT832822	VIPR_ALG4_99822633	2a	NS3	USMARC-60779	2014	-0.5359	0.5901	1.1185
Pestivirus B	MH231125	VIPR_ALG4_AZQ0064	2a	NS3	2139	1992	-0.5255	0.5957	1.1185
Pestivirus B	KP941582	VIPR_ALG4_80092431	2c	NS3	USMARC-53873	2014	-0.4880	0.5939	1.1171
Pestivirus B	KT832819	VIPR_ALG4_99822632	2a	NS3	USMARC-60766	2014	-0.5284	0.5911	1.1202
Pestivirus B	KT832821	VIPR_ALG4_99822633	2c	NS3	USMARC-60768	2014	-0.4764	0.5896	1.1206

Species according to VIPRBRC	GenBank Accession	GenBank Protein Accession	Subgenotype	Protein	Strain Name	Collection Year	SVM Patho. Score	Vaxijen Antig. Score	Averged score of EMBOSS motifs
Pestivirus B	HG426479	VIPR_ALG4_61932573	2c	NS3	D37-13-2_Dup(-)	2013	-0.5138	0.5892	1.1223
Pestivirus B	HG426481	VIPR_ALG4_61932573	2c	NS3	D75-13-609_Dup(-)	2013	-0.5138	0.5892	1.1223
Pestivirus B	MH231123	VIPR_ALG4_AZQ0064	2a	NS3	10406	1993	-0.5308	0.5937	1.1185
Pestivirus B	HG426492	VIPR_ALG4_61932575	2c	NS3	SH2210-14	2010	-0.4952	0.5868	1.1226
Pestivirus B	KT832823	VIPR_ALG4_99822633	2a	NS3	USMARC-60780	2014	-0.5586	0.5916	1.1197
Pestivirus B	HG426485	VIPR_ALG4_61932574	2c	NS3	NRW 14-13_Dup(-)	2013	-0.5138	0.5892	1.1223
Pestivirus B	MH231126	VIPR_ALG4_AZQ0065	2b	NS3	3237	1990	-0.5023	0.5964	1.1204
Pestivirus B	KT832818	VIPR_ALG4_99822632	2a	NS3	USMARC-60765	2014	-0.4918	0.5917	1.1181
Pestivirus B	HG426483	VIPR_ALG4_61932574	2c	NS3	NRW 12-13_Dup(-)	2013	-0.5138	0.5892	1.1223
Pestivirus B	HG426489	VIPR_ALG4_61932575	2c	NS3	NRW 19-13-8_Dup(-)	2013	-0.5138	0.5892	1.1223
Pestivirus B	MH231130	VIPR_ALG4_AZQ0065	2a	NS3	95-1501	1998	-0.4651	0.5897	1.1225
Pestivirus B	MH231132	VIPR_ALG4_AZQ0065	2a	NS3	AzSpl	1997	-0.5032	0.5911	1.1209
Pestivirus B	MH231128	VIPR_ALG4_AZQ0065	2a	NS3	570152	1992	-0.5440	0.5915	1.1174
Pestivirus B	MH231135	VIPR_ALG4_AZQ0065	2a	NS3	BV1907	1995	-0.5191	0.5896	1.1185
Pestivirus B	JF714967	VIPR_ALG4_34657797	2a	NS3	HLJ-10	2011	-0.5092	0.5961	1.1225
Pestivirus B	MH231139	VIPR_ALG4_AZQ0066	2a	NS3	Olwein #12	1990	-0.5359	0.5901	1.1185
Pestivirus B	MH231140	VIPR_ALG4_AZQ0066	2a	NS3	PA	1992	-0.5304	0.5878	1.1185
Pestivirus B	MH231143	VIPR_ALG4_AZQ0066	2a	NS3	RS886	2014	-0.5657	0.5902	1.1189
Pestivirus B	HQ258810	VIPR_ALG4_31199027	2a	NS3	SH-28	2009	-0.5292	0.5860	1.1209
Pestivirus B	MH231145	VIPR_ALG4_AZQ0066	2a	NS3	Victor301	1990	-0.5009	0.5897	1.1185
Pestivirus B	MH231146	VIPR_ALG4_AZQ0067	2a	NS3	WiscA	1991	-0.4841	0.5909	1.1185
Pestivirus B	FJ527854	VIPR_ALG4_22961018	2a	NS3	XJ-04	2004	-0.5834	0.5824	1.1196
Pestivirus B	KC963968	VIPR_ALG4_53029119	2a	NS3	11F011	2011	-0.5191	0.5896	1.1185
Pestivirus B	MH806437	VIPR_ALG4_AZP5716	2a	NS3	9231	2004	-0.5219	0.5907	1.1185
Pestivirus B	MG879027	VIPR_ALG4_AVA3071	2a	NS3	CN10.2015.821	2014	-0.5269	0.5937	1.1176
Pestivirus B	KX096718	VIPR_ALG4_11130170	2a	NS3	HB-1511	2015	-0.4856	0.5715	1.1158
Pestivirus B	LC649064	VIPR_ALG4_BDB0736	2c	NS3	KZ-91-NCP	1991	-0.4926	0.5911	1.1198
Pestivirus B	MH231147	VIPR_ALG4_AZQ0067	2c	NS3	PI12	2016	-0.4593	0.5944	1.1193
Pestivirus B	GQ888686	VIPR_ALG4_27071949	2a	NS3	JZ05-1	2005	-0.5941	0.5950	1.1168
Pestivirus B	MW168422	VIPR_ALG4_QZM0693	2a	NS3	YNJG2020	2020	-0.4496	0.5875	1.1132
Pestivirus B	HG426488	VIPR_ALG4_61932575	2c	NS3	NRW 19-13-1_Dup(+)	2013	-0.5138	0.5892	1.1223
Pestivirus B	HG426480	VIPR_ALG4_61932573	2c	NS3	D37-13-2_Dup(+)	2013	-0.5138	0.5892	1.1223
Pestivirus B	HG426482	VIPR_ALG4_61932573	2c	NS3	D75-13-609_Dup(+)	2013	-0.5138	0.5892	1.1223
Pestivirus B	HG426486	VIPR_ALG4_61932574	2c	NS3	NRW 14-13_Dup(+)	2013	-0.5138	0.5892	1.1223
Pestivirus B	HG426484	VIPR_ALG4_61932574	2c	NS3	NRW 12-13_Dup(+)	2013	-0.5138	0.5892	1.1223
Pestivirus B	HG426490	VIPR_ALG4_61932575	2c	NS3	NRW 19-13-8_Dup(+)	2013	-0.5138	0.5892	1.1223
Pestivirus B	MH231129	VIPR_ALG4_AZQ0065	2a	NS3	5912c	1995	-0.5362	0.5884	1.1185
Pestivirus B	MH806438	VIPR_ALG4_AZP5716	2a	NS3	McCart_c	1989	-0.4651	0.5897	1.1225
Pestivirus B	MH806434	VIPR_ALG4_AZP5716	2a	NS3	125c	1990	-0.5237	0.5926	1.1176
Pestivirus B	MH231133	VIPR_ALG4_AZQ0065	2e	NS3	B69519c	2006	-0.4829	0.5934	1.1203
Pestivirus B	MH806436	VIPR_ALG4_AZP5716	2a	NS3	296c	1995	-0.5191	0.5896	1.1185
Pestivirus B	MH231124	VIPR_ALG4_AZQ0064	2e	NS3	1786c	1989	-0.4829	0.5934	1.1203
Pestivirus B	MH231127	VIPR_ALG4_AZQ0065	2a	NS3	53637c	2004	-0.5486	0.5796	1.1204
Pestivirus B	MN824468	VIPR_ALG4_QJF1227	2a	NS3	CPAE_contamination/2018	2018	-0.5394	0.5932	1.1179
Pestivirus B	HQ444199	VIPR_ALG4_32680714	2a	NS3	Ind141353	2007	-0.5285	0.5993	1.1202
Pestivirus B	MH231127	VIPR_ALG4_AZQ0065	2a	NS2	53637c	2004	-0.8683	0.5912	1.1429
Pestivirus B	MH806434	VIPR_ALG4_AZP5716	2a	NS2	125c	1990	-0.9754	0.6066	1.1420

Species according to VIPRBRC	GenBank Accession	GenBank Protein Accession	Subgenotype	Protein	Strain Name	Collection Year	SVM Patho. Score	Vaxijen Antig. Score	Averged score of EMBOSS motifs
Pestivirus B	MH231133	VIPR_ALG4_AZQ0065	2e	NS2	B69519c	2006	-0.6259	0.5676	1.1308
Pestivirus B	MH231124	VIPR_ALG4_AZQ0064	2e	NS2	1786c	1989	-0.6684	0.5728	1.1308
Pestivirus B	MH806436	VIPR_ALG4_AZP5716	2a	NS2	296c	1995	-0.8582	0.5642	1.1413
Pestivirus B	MH806438	VIPR_ALG4_AZP5716	2a	NS2	McCart_c	1989	-0.7096	0.6017	1.1467
Pestivirus B	MH231129	VIPR_ALG4_AZQ0065	2a	NS2	5912c	1995	-0.8926	0.5690	1.1430
Pestivirus B	HG426488	VIPR_ALG4_61932575	2c	NS2	NRW 19-13-1_Dup(+)	2013	-0.8072	0.5820	1.1557
Pestivirus B	HG426480	VIPR_ALG4_61932573	2c	NS2	D37-13-2_Dup(+)	2013	-0.7783	0.5857	1.1557
Pestivirus B	HG426482	VIPR_ALG4_61932573	2c	NS2	D75-13-609_Dup(+)	2013	-0.7981	0.5853	1.1529
Pestivirus B	HG426486	VIPR_ALG4_61932574	2c	NS2	NRW 14-13_Dup(+)	2013	-0.7484	0.5728	1.1557
Pestivirus B	HG426484	VIPR_ALG4_61932574	2c	NS2	NRW 12-13_Dup(+)	2013	-0.8072	0.5820	1.1557
Pestivirus B	HG426490	VIPR_ALG4_61932575	2c	NS2	NRW 19-13-8_Dup(+)	2013	-0.8161	0.5892	1.1557
Pestivirus B	KP057803	VIPR_ALG4_80287536	2a	NS2	24515	1993	-1.0851	0.5750	1.1535
Pestivirus B	KR093034	VIPR_ALG4_92904888	2a	NS2	NY-93	1993	-1.0204	0.5719	1.1523
Pestivirus B	KT875139	VIPR_ALG4_10037029	2a	NS2	32W	2005	-0.8442	0.5884	1.1581
Pestivirus B	KT875134	VIPR_ALG4_10037029	2a	NS2	12W	2005	-0.8007	0.5897	1.1571
Pestivirus B	KT875135	VIPR_ALG4_10037029	2a	NS2	13Y	2005	-0.8632	0.5872	1.1581
Pestivirus B	KT875136	VIPR_ALG4_10037029	2a	NS2	27Y	2005	-0.8442	0.5884	1.1581
Pestivirus B	KT875137	VIPR_ALG4_10037029	2a	NS2	29Y	2005	-0.8442	0.5884	1.1581
Pestivirus B	KT875138	VIPR_ALG4_10037029	2a	NS2	2Y	2005	-0.8883	0.5890	1.1581
Pestivirus B	KT875140	VIPR_ALG4_10037029	2a	NS2	34Y	2005	-0.8442	0.5884	1.1581
Pestivirus B	KT875141	VIPR_ALG4_10037029	2a	NS2	36W	2005	-0.8884	0.5894	1.1581
Pestivirus B	KT875142	VIPR_ALG4_10037029	2a	NS2	41Y	2005	-0.8442	0.5884	1.1581
Pestivirus B	KT875143	VIPR_ALG4_10037029	2a	NS2	42W	2005	-0.8514	0.5805	1.1573
Pestivirus B	KT875144	VIPR_ALG4_10037029	2a	NS2	43Y	2005	-0.8632	0.5872	1.1581
Pestivirus B	KT875145	VIPR_ALG4_10037029	2a	NS2	47Y	2005	-0.8496	0.5858	1.1573
Pestivirus B	KT875146	VIPR_ALG4_10037029	2a	NS2	50Y	2005	-0.8442	0.5884	1.1581
Pestivirus B	KT875147	VIPR_ALG4_10037029	2a	NS2	51W	2005	-0.8442	0.5884	1.1581
Pestivirus B	KT875148	VIPR_ALG4_10037029	2a	NS2	51Y	2005	-0.8442	0.5884	1.1581
Pestivirus B	KT875149	VIPR_ALG4_10037029	2a	NS2	53W	2005	-0.8442	0.5884	1.1581
Pestivirus B	KT875150	VIPR_ALG4_10037029	2a	NS2	58W	2005	-0.8442	0.5884	1.1581
Pestivirus B	KT875151	VIPR_ALG4_10037029	2a	NS2	58Y	2005	-0.8442	0.5884	1.1581
Pestivirus B	KT875152	VIPR_ALG4_10037029	2a	NS2	5Y	2005	-0.8442	0.5884	1.1581
Pestivirus B	KT875153	VIPR_ALG4_10037029	2a	NS2	62Y	2005	-0.8442	0.5884	1.1581
Pestivirus B	KT875154	VIPR_ALG4_10037029	2a	NS2	65Y	2005	-0.8442	0.5884	1.1581
Pestivirus B	KT875155	VIPR_ALG4_10037029	2a	NS2	67Y	2005	-0.8442	0.5884	1.1581
Pestivirus B	KT875156	VIPR_ALG4_10037029	2a	NS2	68W	2005	-0.8632	0.5872	1.1581
Pestivirus B	KT875157	VIPR_ALG4_10037029	2a	NS2	71Y	2005	-0.8442	0.5884	1.1581
Pestivirus B	KT875158	VIPR_ALG4_10037029	2a	NS2	73Y	2005	-0.8442	0.5884	1.1581
Pestivirus B	KT875159	VIPR_ALG4_10037029	2a	NS2	74Y	2005	-0.8442	0.5884	1.1581
Pestivirus B	KT875160	VIPR_ALG4_10037029	2a	NS2	75W	2005	-0.8442	0.5884	1.1581
Pestivirus B	KT875161	VIPR_ALG4_10037029	2a	NS2	75Y	2005	-0.8224	0.5912	1.1589
Pestivirus B	KT875162	VIPR_ALG4_10037029	2a	NS2	76Y	2005	-0.8514	0.5805	1.1573
Pestivirus B	KT875163	VIPR_ALG4_10037029	2a	NS2	78W	2005	-0.8514	0.5805	1.1573
Pestivirus B	KT875164	VIPR_ALG4_10037029	2a	NS2	79W	2005	-0.8884	0.5894	1.1581
Pestivirus B	KT875165	VIPR_ALG4_10037029	2a	NS2	7W	2005	-0.8442	0.5884	1.1581
Pestivirus B	KT875166	VIPR_ALG4_10037029	2a	NS2	82W	2005	-0.8442	0.5884	1.1581
Pestivirus B	KT875167	VIPR_ALG4_10037029	2a	NS2	83Y	2005	-0.8514	0.5805	1.1573

Species according to VIPRBRC	GenBank Accession	GenBank Protein Accession	Subgenotype	Protein	Strain Name	Collection Year	SVM Patho. Score	Vaxijen Antig. Score	Averged score of EMBOSS motifs
Pestivirus B	KT875168	VIPR_ALG4_10037029	2a	NS2	90W	2005	-0.8884	0.5894	1.1581
Pestivirus B	KT875169	VIPR_ALG4_10037030	2a	NS2	91W	2005	-0.8496	0.5858	1.1573
Pestivirus B	MW006485	VIPR_ALG4_QPF4972	2b	NS2	HEN01	2014	-0.9556	0.5819	1.1495
Pestivirus B	MH231142	VIPR_ALG4_AZQ0066	2c	NS2	Parker	1991	-0.7461	0.5746	1.1550
Pestivirus B	MH806435	VIPR_ALG4_AZP5716	2a	NS2	1336H	2005	-0.8149	0.5503	1.1526
Pestivirus B	MH231136	VIPR_ALG4_AZQ0066	2a	NS2	JV14	1998	-1.0067	0.5956	1.1475
Pestivirus B	MK599227	VIPR_ALG4_QEU5262	2a	NS2	SD-1	2016	-0.8013	0.5492	1.1481
Pestivirus B	HG426487	VIPR_ALG4_61932574	2c	NS2	NRW 19-13-1_Dup(-)	2013	-0.7145	0.5691	1.1550
Pestivirus B	MH231138	VIPR_ALG4_AZQ0066	2a	NS2	MnFetus	1991	-0.8734	0.6022	1.1463
Pestivirus B	MH231131	VIPR_ALG4_AZQ0065	2a	NS2	AU501	2006	-0.7212	0.5778	1.1632
Pestivirus B	MH231151	VIPR_ALG4_AZQ0067	2e	NS2	14622	2005	-0.6096	0.5557	1.1381
Pestivirus B	MH231152	VIPR_ALG4_AZQ0067	2e	NS2	2412	1989	-0.5782	0.5529	1.1381
Pestivirus B	MH231149	VIPR_ALG4_AZQ0067	2e	NS2	Short	1989	-0.6096	0.5557	1.1381
Pestivirus B	KJ000672	VIPR_ALG4_59423572	2b	NS2	SD1301	2012	-0.9556	0.5819	1.1495
Pestivirus B	MH231148	VIPR_ALG4_AZQ0067	2e	NS2	12-149150	2012	-0.7245	0.5772	1.1379
Pestivirus B	MH231150	VIPR_ALG4_AZQ0067	2e	NS2	12-151955-317	2012	-0.7002	0.5733	1.1349
Pestivirus B	HG426495	VIPR_ALG4_61932576	2c	NS2	VOE 4407	2007	-0.7629	0.5711	1.1560
Pestivirus B	HG426491	VIPR_ALG4_61932575	2c	NS2	Potsdam 1600	2000	-0.8686	0.5720	1.1550
Pestivirus B	MH231141	VIPR_ALG4_AZQ0066	2a	NS2	PI28	2016	-0.8459	0.6122	1.1469
Pestivirus B	HG426494	VIPR_ALG4_61932576	2c	NS2	SH2210-23	2010	-0.7285	0.5762	1.1568
Pestivirus B	MH231137	VIPR_ALG4_AZQ0066	2a	NS2	MadSpl	1991	-0.9435	0.6004	1.1462
Pestivirus B	KP941585	VIPR_ALG4_80092432	2a	NS2	USMARC-55476	2014	-0.9152	0.6119	1.1478
Pestivirus B	KT832820	VIPR_ALG4_99822633	2a	NS2	USMARC-60767	2014	-0.8256	0.5952	1.1443
Pestivirus B	MH231134	VIPR_ALG4_AZQ0065	2a	NS2	B9497	1997	-0.7434	0.5765	1.1560
Pestivirus B	MN527354	VIPR_ALG4_QLH0204	2a	NS2	GS2018	2018	-0.6466	0.5764	1.1636
Pestivirus B	MH231144	VIPR_ALG4_AZQ0066	2a	NS2	Sanderson6319	1992	-0.9293	0.6001	1.1505
Pestivirus B	HG426493	VIPR_ALG4_61932576	2c	NS2	SH2210-17	2010	-0.7389	0.5693	1.1572
Pestivirus B	KT832817	VIPR_ALG4_99822632	2a	NS2	USMARC-60764	2014	-0.6921	0.5797	1.1615
Pestivirus B	KT832822	VIPR_ALG4_99822633	2a	NS2	USMARC-60779	2014	-1.0237	0.5976	1.1444
Pestivirus B	MH231125	VIPR_ALG4_AZQ0064	2a	NS2	2139	1992	-0.8798	0.5980	1.1471
Pestivirus B	KP941582	VIPR_ALG4_80092431	2c	NS2	USMARC-53873	2014	-0.6864	0.5935	1.1548
Pestivirus B	KT832819	VIPR_ALG4_99822632	2a	NS2	USMARC-60766	2014	-0.7622	0.6129	1.1460
Pestivirus B	KT832821	VIPR_ALG4_99822633	2c	NS2	USMARC-60768	2014	-0.5343	0.5846	1.1537
Pestivirus B	HG426479	VIPR_ALG4_61932573	2c	NS2	D37-13-2_Dup(-)	2013	-0.7145	0.5691	1.1550
Pestivirus B	HG426481	VIPR_ALG4_61932573	2c	NS2	D75-13-609_Dup(-)	2013	-0.7715	0.5834	1.1523
Pestivirus B	MH231123	VIPR_ALG4_AZQ0064	2a	NS2	10406	1993	-0.8745	0.5997	1.1474
Pestivirus B	HG426492	VIPR_ALG4_61932575	2c	NS2	SH2210-14	2010	-0.7389	0.5693	1.1572
Pestivirus B	KT832823	VIPR_ALG4_99822633	2a	NS2	USMARC-60780	2014	-0.9701	0.5766	1.1582
Pestivirus B	HG426485	VIPR_ALG4_61932574	2c	NS2	NRW 14-13_Dup(-)	2013	-0.7218	0.5709	1.1550
Pestivirus B	MH231126	VIPR_ALG4_AZQ0065	2b	NS2	3237	1990	-0.6297	0.5661	1.1564
Pestivirus B	KT832818	VIPR_ALG4_99822632	2a	NS2	USMARC-60765	2014	-0.9501	0.5461	1.1548
Pestivirus B	HG426483	VIPR_ALG4_61932574	2c	NS2	NRW 12-13_Dup(-)	2013	-0.7798	0.5801	1.1550
Pestivirus B	HG426489	VIPR_ALG4_61932575	2c	NS2	NRW 19-13-8_Dup(-)	2013	-0.7875	0.5873	1.1550
Pestivirus B	MH231130	VIPR_ALG4_AZQ0065	2a	NS2	95-1501	1998	-0.7976	0.5732	1.1614
Pestivirus B	MH231132	VIPR_ALG4_AZQ0065	2a	NS2	AzSpl	1997	-0.9827	0.5903	1.1635
Pestivirus B	MH231128	VIPR_ALG4_AZQ0065	2a	NS2	570152	1992	-1.0113	0.6005	1.1474
Pestivirus B	MH231135	VIPR_ALG4_AZQ0065	2a	NS2	BV1907	1995	-1.0350	0.6033	1.1506

Species according to VIPRBRC	GenBank Accession	GenBank Protein Accession	Subgenotype	Protein	Strain Name	Collection Year	SVM Patho. Score	Vaxijen Antig. Score	Averged score of EMBOSS motifs
Pestivirus B	JF714967	VIPR_ALG4_34657797	2a	NS2	HLJ-10	2011	-0.8562	0.5568	1.1558
Pestivirus B	MH231139	VIPR_ALG4_AZQ0066	2a	NS2	Olwein #12	1990	-0.8642	0.5785	1.1590
Pestivirus B	MH231140	VIPR_ALG4_AZQ0066	2a	NS2	PA	1992	-0.9444	0.5990	1.1507
Pestivirus B	MH231143	VIPR_ALG4_AZQ0066	2a	NS2	RS886	2014	-0.7656	0.5943	1.1449
Pestivirus B	HQ258810	VIPR_ALG4_31199027	2a	NS2	SH-28	2009	-0.9705	0.5943	1.1588
Pestivirus B	MH231145	VIPR_ALG4_AZQ0066	2a	NS2	Victor301	1990	-0.9696	0.5895	1.1507
Pestivirus B	MH231146	VIPR_ALG4_AZQ0067	2a	NS2	WiscA	1991	-0.9342	0.5992	1.1474
Pestivirus B	FJ527854	VIPR_ALG4_22961018	2a	NS2	XJ-04	2004	-0.9142	0.5783	1.1566
Pestivirus B	KC963968	VIPR_ALG4_53029119	2a	NS2	11F011	2011	-0.9503	0.6027	1.1463
Pestivirus B	MH806437	VIPR_ALG4_AZP5716	2a	NS2	9231	2004	-0.7942	0.5764	1.1651
Pestivirus B	MG879027	VIPR_ALG4_AVA3071	2a	NS2	CN10.2015.821	2014	-0.8432	0.5858	1.1689
Pestivirus B	KX096718	VIPR_ALG4_11130170	2a	NS2	HB-1511	2015	-0.7200	0.5804	1.1558
Pestivirus B	LC649064	VIPR_ALG4_BDB0736	2c	NS2	KZ-91-NCP	1991	-0.5786	0.5934	1.1622
Pestivirus B	MH231147	VIPR_ALG4_AZQ0067	2c	NS2	PI12	2016	-0.6631	0.5823	1.1544
Pestivirus B	GQ888686	VIPR_ALG4_27071949	2a	NS2	JZ05-1	2005	-0.5697	0.5539	1.1420
Pestivirus B	MW168422	VIPR_ALG4_QZM0693	2a	NS2	YNJG2020	2020	-0.7741	0.5966	1.1553
Pestivirus B	MN824468	VIPR_ALG4_QJF1227	2a	NS2	CPAE_contamination/2018	2018	-0.8477	0.6027	1.1574
Pestivirus B	HQ444199	VIPR_ALG4_32680714	2a	NS2	Ind141353	2007	-0.9142	0.5789	1.1582
Pestivirus B	KX170479	APT71002.1	2a	NS4A	V017	2003	-1.0117	0.7116	1.1017
Pestivirus B	KX170504	APT71027.1	2a	NS4A	V023	2006	0.5910	0.5945	1.1210
Pestivirus B	KX170476	APT70999.1	2a	NS4A	V028	2006	-0.9335	0.6656	1.0960
Pestivirus B	KX170480	APT71003.1	2a	NS4A	V047	2009	-0.9335	0.6656	1.0960
Pestivirus B	KX170483	APT71006.1	2a	NS4A	V051	2010	-0.9335	0.6656	1.0960
Pestivirus B	KX170482	APT71005.1	2a	NS4A	V063	2005	-0.9335	0.6656	1.0960
Pestivirus B	KX170473	APT70996.1	2a	NS4A	V076	2011	-0.4170	0.7144	1.1205
Pestivirus B	KX170481	APT71004.1	2a	NS4A	V085	2007	-0.9335	0.6656	1.0960
Pestivirus B	KX170477	APT71000.1	2a	NS4A	V086	2006	-0.9335	0.6656	1.0960
Pestivirus B	KX170478	APT71001.1	2a	NS4A	V088	2005	-0.9335	0.6656	1.0960
Pestivirus B	KX170475	APT70998.1	2a	NS4A	V089	2004	-0.9335	0.6656	1.0960
Pestivirus B	KX170474	APT70997.1	2a	NS4A	V095	1999	-0.9335	0.6656	1.0960
Pestivirus B	KX170484	APT71007.1	2a	NS4A	V065	2006	-0.9335	0.6656	1.0960
Pestivirus B	KX838370	VIPR_ALG4_AOS5274	2a	NS4A	Ind141353	2007	-0.9335	0.6656	1.0960
Pestivirus B	KT875139	VIPR_ALG4_10037029	2a	NS4A	32W	2005	-0.9335	0.6656	1.0960
Pestivirus B	MN824468	VIPR_ALG4_QJF1227	2a	NS4A	CPAE_contamination/2018	2018	-0.9335	0.6656	1.0960
Pestivirus B	KT875134	VIPR_ALG4_10037029	2a	NS4A	12W	2005	-0.9335	0.6656	1.0960
Pestivirus B	KT875135	VIPR_ALG4_10037029	2a	NS4A	13Y	2005	-0.9335	0.6656	1.0960
Pestivirus B	KT875136	VIPR_ALG4_10037029	2a	NS4A	27Y	2005	-0.9335	0.6656	1.0960
Pestivirus B	KT875137	VIPR_ALG4_10037029	2a	NS4A	29Y	2005	-0.9335	0.6656	1.0960
Pestivirus B	KT875138	VIPR_ALG4_10037029	2a	NS4A	2Y	2005	-0.9335	0.6656	1.0960
Pestivirus B	KT875140	VIPR_ALG4_10037029	2a	NS4A	34Y	2005	-0.9335	0.6656	1.0960
Pestivirus B	KT875141	VIPR_ALG4_10037029	2a	NS4A	36W	2005	-0.9335	0.6656	1.0960
Pestivirus B	KT875142	VIPR_ALG4_10037029	2a	NS4A	41Y	2005	-0.9335	0.6656	1.0960
Pestivirus B	KT875143	VIPR_ALG4_10037029	2a	NS4A	42W	2005	-0.9335	0.6656	1.0960
Pestivirus B	KT875144	VIPR_ALG4_10037029	2a	NS4A	43Y	2005	-0.9335	0.6656	1.0960
Pestivirus B	KT875145	VIPR_ALG4_10037029	2a	NS4A	47Y	2005	-0.9335	0.6656	1.0960
Pestivirus B	KT875146	VIPR_ALG4_10037029	2a	NS4A	50Y	2005	-0.9335	0.6656	1.0960
Pestivirus B	KT875147	VIPR_ALG4_10037029	2a	NS4A	51W	2005	-0.9335	0.6656	1.0960

Species according to VIPRBRC	GenBank Accession	GenBank Protein Accession	Subgenotype	Protein	Strain Name	Collection Year	SVM Patho. Score	Vaxijen Antig. Score	Averged score of EMBOSS motifs
Pestivirus B	KT875148	VIPR_ALG4_10037029	2a	NS4A	51Y	2005	-0.9335	0.6656	1.0960
Pestivirus B	KT875149	VIPR_ALG4_10037029	2a	NS4A	53W	2005	-0.9335	0.6656	1.0960
Pestivirus B	KT875150	VIPR_ALG4_10037029	2a	NS4A	58W	2005	-0.9335	0.6656	1.0960
Pestivirus B	KT875151	VIPR_ALG4_10037029	2a	NS4A	58Y	2005	-0.9335	0.6656	1.0960
Pestivirus B	KT875152	VIPR_ALG4_10037029	2a	NS4A	5Y	2005	-0.9335	0.6656	1.0960
Pestivirus B	KT875153	VIPR_ALG4_10037029	2a	NS4A	62Y	2005	-0.9335	0.6656	1.0960
Pestivirus B	KT875154	VIPR_ALG4_10037029	2a	NS4A	65Y	2005	-0.9335	0.6656	1.0960
Pestivirus B	KT875155	VIPR_ALG4_10037029	2a	NS4A	67Y	2005	-0.9335	0.6656	1.0960
Pestivirus B	KT875156	VIPR_ALG4_10037029	2a	NS4A	68W	2005	-0.9335	0.6656	1.0960
Pestivirus B	KT875157	VIPR_ALG4_10037029	2a	NS4A	71Y	2005	-0.9335	0.6656	1.0960
Pestivirus B	KT875158	VIPR_ALG4_10037029	2a	NS4A	73Y	2005	-0.9335	0.6656	1.0960
Pestivirus B	KT875159	VIPR_ALG4_10037029	2a	NS4A	74Y	2005	-0.9335	0.6656	1.0960
Pestivirus B	KT875160	VIPR_ALG4_10037029	2a	NS4A	75W	2005	-0.9335	0.6656	1.0960
Pestivirus B	KT875161	VIPR_ALG4_10037029	2a	NS4A	75Y	2005	-0.9335	0.6656	1.0960
Pestivirus B	KT875162	VIPR_ALG4_10037029	2a	NS4A	76Y	2005	-0.9335	0.6656	1.0960
Pestivirus B	KT875163	VIPR_ALG4_10037029	2a	NS4A	78W	2005	-0.9335	0.6656	1.0960
Pestivirus B	KT875164	VIPR_ALG4_10037029	2a	NS4A	79W	2005	-0.9335	0.6656	1.0960
Pestivirus B	KT875165	VIPR_ALG4_10037029	2a	NS4A	7W	2005	-0.9335	0.6656	1.0960
Pestivirus B	KT875166	VIPR_ALG4_10037029	2a	NS4A	82W	2005	-0.9335	0.6656	1.0960
Pestivirus B	KT875167	VIPR_ALG4_10037029	2a	NS4A	83Y	2005	-0.9335	0.6656	1.0960
Pestivirus B	KT875168	VIPR_ALG4_10037029	2a	NS4A	90W	2005	-0.9335	0.6656	1.0960
Pestivirus B	KT875169	VIPR_ALG4_10037030	2a	NS4A	91W	2005	-0.9335	0.6656	1.0960
Pestivirus B	KP057803	VIPR_ALG4_80287536	2a	NS4A	24515	1993	-0.9335	0.6656	1.0960
Pestivirus B	KR093034	VIPR_ALG4_92904888	2a	NS4A	NY-93	1993	-0.9335	0.6656	1.0960
Pestivirus B	MW006485	VIPR_ALG4_QPF4972	2b	NS4A	HEN01	2014	-0.8974	0.6708	1.0960
Pestivirus B	MH231142	VIPR_ALG4_AZQ0066	2c	NS4A	Parker	1991	-0.9335	0.6656	1.0960
Pestivirus B	MH806435	VIPR_ALG4_AZP5716	2a	NS4A	1336H	2005	-0.9335	0.6656	1.0960
Pestivirus B	MH231136	VIPR_ALG4_AZQ0066	2a	NS4A	JV14	1998	-0.6148	0.6746	1.1070
Pestivirus B	MK599227	VIPR_ALG4_QEU5262	2a	NS4A	SD-1	2016	-0.9335	0.6656	1.0960
Pestivirus B	HG426487	VIPR_ALG4_61932574	2c	NS4A	NRW 19-13-1_Dup(-)	2013	-0.9335	0.6656	1.0960
Pestivirus B	MH231138	VIPR_ALG4_AZQ0066	2a	NS4A	MnFetus	1991	-0.9335	0.6656	1.0960
Pestivirus B	MH231131	VIPR_ALG4_AZQ0065	2a	NS4A	AU501	2006	-0.9335	0.6656	1.0960
Pestivirus B	MH231151	VIPR_ALG4_AZQ0067	2e	NS4A	14622	2005	-0.9335	0.6656	1.0960
Pestivirus B	MH231152	VIPR_ALG4_AZQ0067	2e	NS4A	2412	1989	-0.9335	0.6656	1.0960
Pestivirus B	MH231149	VIPR_ALG4_AZQ0067	2e	NS4A	Short	1989	-0.9335	0.6656	1.0960
Pestivirus B	KJ000672	VIPR_ALG4_59423572	2b	NS4A	SD1301	2012	-0.8974	0.6708	1.0960
Pestivirus B	MH231148	VIPR_ALG4_AZQ0067	2e	NS4A	12-149150	2012	-0.9335	0.6656	1.0960
Pestivirus B	MH231150	VIPR_ALG4_AZQ0067	2e	NS4A	12-151955-317	2012	-0.9335	0.6656	1.0960
Pestivirus B	HG426495	VIPR_ALG4_61932576	2c	NS4A	VOE 4407	2007	-0.9335	0.6656	1.0960
Pestivirus B	HG426491	VIPR_ALG4_61932575	2c	NS4A	Potsdam 1600	2000	-0.9335	0.6656	1.0960
Pestivirus B	MH231141	VIPR_ALG4_AZQ0066	2a	NS4A	PI28	2016	-0.9335	0.6656	1.0960
Pestivirus B	HG426494	VIPR_ALG4_61932576	2c	NS4A	SH2210-23	2010	-0.9335	0.6656	1.0960
Pestivirus B	MH231137	VIPR_ALG4_AZQ0066	2a	NS4A	MadSpl	1991	-0.9335	0.6656	1.0960
Pestivirus B	KP941585	VIPR_ALG4_80092432	2a	NS4A	USMARC-55476	2014	-0.9335	0.6656	1.0960
Pestivirus B	KT832820	VIPR_ALG4_99822633	2a	NS4A	USMARC-60767	2014	-0.9335	0.6656	1.0960
Pestivirus B	MH231134	VIPR_ALG4_AZQ0065	2a	NS4A	B9497	1997	-0.9335	0.6656	1.0960
Pestivirus B	MN527354	VIPR_ALG4_QLH0204	2a	NS4A	GS2018	2018	-0.6499	0.6624	1.1007

Species according to VIPRBRC	GenBank Accession	GenBank Protein Accession	Subgenotype	Protein	Strain Name	Collection Year	SVM Patho. Score	Vaxijen Antig. Score	Averged score of EMBOSS motifs
Pestivirus B	MH231144	VIPR_ALG4_AZQ0066	2a	NS4A	Sanderson6319	1992	-0.9335	0.6656	1.0960
Pestivirus B	HG426493	VIPR_ALG4_61932576	2c	NS4A	SH2210-17	2010	-0.8196	0.6647	1.0960
Pestivirus B	KT832817	VIPR_ALG4_99822632	2a	NS4A	USMARC-60764	2014	-0.9335	0.6656	1.0960
Pestivirus B	KT832822	VIPR_ALG4_99822633	2a	NS4A	USMARC-60779	2014	-0.9335	0.6656	1.0960
Pestivirus B	MH231125	VIPR_ALG4_AZQ0064	2a	NS4A	2139	1992	-0.9335	0.6656	1.0960
Pestivirus B	KP941582	VIPR_ALG4_80092431	2c	NS4A	USMARC-53873	2014	-0.9335	0.6656	1.0960
Pestivirus B	KT832819	VIPR_ALG4_99822632	2a	NS4A	USMARC-60766	2014	-0.9335	0.6656	1.0960
Pestivirus B	KT832821	VIPR_ALG4_99822633	2c	NS4A	USMARC-60768	2014	-0.9335	0.6656	1.0960
Pestivirus B	HG426479	VIPR_ALG4_61932573	2c	NS4A	D37-13-2_Dup(-)	2013	-0.9335	0.6656	1.0960
Pestivirus B	HG426481	VIPR_ALG4_61932573	2c	NS4A	D75-13-609_Dup(-)	2013	-0.9335	0.6656	1.0960
Pestivirus B	MH231123	VIPR_ALG4_AZQ0064	2a	NS4A	10406	1993	-0.8884	0.6758	1.1070
Pestivirus B	HG426492	VIPR_ALG4_61932575	2c	NS4A	SH2210-14	2010	-0.8196	0.6647	1.0960
Pestivirus B	KT832823	VIPR_ALG4_99822633	2a	NS4A	USMARC-60780	2014	-0.6407	0.6594	1.0960
Pestivirus B	HG426485	VIPR_ALG4_61932574	2c	NS4A	NRW 14-13_Dup(-)	2013	-0.9335	0.6656	1.0960
Pestivirus B	MH231126	VIPR_ALG4_AZQ0065	2b	NS4A	3237	1990	-0.9335	0.6656	1.0960
Pestivirus B	KT832818	VIPR_ALG4_99822632	2a	NS4A	USMARC-60765	2014	-0.6148	0.6746	1.1070
Pestivirus B	HG426483	VIPR_ALG4_61932574	2c	NS4A	NRW 12-13_Dup(-)	2013	-0.9335	0.6656	1.0960
Pestivirus B	HG426489	VIPR_ALG4_61932575	2c	NS4A	NRW 19-13-8_Dup(-)	2013	-0.9335	0.6656	1.0960
Pestivirus B	MH231130	VIPR_ALG4_AZQ0065	2a	NS4A	95-1501	1998	-0.9335	0.6656	1.0960
Pestivirus B	MH231132	VIPR_ALG4_AZQ0065	2a	NS4A	AzSpl	1997	-0.6694	0.6120	1.0960
Pestivirus B	MH231128	VIPR_ALG4_AZQ0065	2a	NS4A	570152	1992	-0.9335	0.6656	1.0960
Pestivirus B	MH231135	VIPR_ALG4_AZQ0065	2a	NS4A	BV1907	1995	-0.9335	0.6656	1.0960
Pestivirus B	JF714967	VIPR_ALG4_34657797	2a	NS4A	HLJ-10	2011	-0.9335	0.6656	1.0960
Pestivirus B	MH231139	VIPR_ALG4_AZQ0066	2a	NS4A	Olwein #12	1990	-0.9335	0.6656	1.0960
Pestivirus B	MH231140	VIPR_ALG4_AZQ0066	2a	NS4A	PA	1992	-0.9335	0.6656	1.0960
Pestivirus B	MH231143	VIPR_ALG4_AZQ0066	2a	NS4A	RS886	2014	-0.9335	0.6656	1.0960
Pestivirus B	HQ258810	VIPR_ALG4_31199027	2a	NS4A	SH-28	2009	-0.8196	0.6647	1.0960
Pestivirus B	MH231145	VIPR_ALG4_AZQ0066	2a	NS4A	Victor301	1990	-0.6148	0.6746	1.1070
Pestivirus B	MH231146	VIPR_ALG4_AZQ0067	2a	NS4A	WiscA	1991	-0.9335	0.6656	1.0960
Pestivirus B	FJ527854	VIPR_ALG4_22961018	2a	NS4A	XJ-04	2004	-0.8354	0.6206	1.0993
Pestivirus B	KC963968	VIPR_ALG4_53029119	2a	NS4A	11F011	2011	-1.0455	0.7309	1.0960
Pestivirus B	MH806437	VIPR_ALG4_AZP5716	2a	NS4A	9231	2004	-0.9335	0.6656	1.0960
Pestivirus B	MG879027	VIPR_ALG4_AVA3071	2a	NS4A	CN10.2015.821	2014	-0.9335	0.6656	1.0960
Pestivirus B	KX096718	VIPR_ALG4_11130170	2a	NS4A	HB-1511	2015	-0.7859	0.6607	1.1155
Pestivirus B	LC649064	VIPR_ALG4_BDB0736	2c	NS4A	KZ-91-NCP	1991	-0.9335	0.6656	1.0960
Pestivirus B	MH231147	VIPR_ALG4_AZQ0067	2c	NS4A	PI12	2016	-0.9335	0.6656	1.0960
Pestivirus B	GQ888686	VIPR_ALG4_27071949	2a	NS4A	JZ05-1	2005	-0.5150	0.6692	1.1180
Pestivirus B	MW168422	VIPR_ALG4_QZM0693	2a	NS4A	YNJG2020	2020	-0.4350	0.5409	1.0923
Pestivirus B	HG426488	VIPR_ALG4_61932575	2c	NS4A	NRW 19-13-1_Dup(+)	2013	-0.9335	0.6656	1.0960
Pestivirus B	HG426480	VIPR_ALG4_61932573	2c	NS4A	D37-13-2_Dup(+)	2013	-0.9335	0.6656	1.0960
Pestivirus B	HG426482	VIPR_ALG4_61932573	2c	NS4A	D75-13-609_Dup(+)	2013	-0.9335	0.6656	1.0960
Pestivirus B	HG426486	VIPR_ALG4_61932574	2c	NS4A	NRW 14-13_Dup(+)	2013	-0.9335	0.6656	1.0960
Pestivirus B	HG426484	VIPR_ALG4_61932574	2c	NS4A	NRW 12-13_Dup(+)	2013	-0.9335	0.6656	1.0960
Pestivirus B	HG426490	VIPR_ALG4_61932575	2c	NS4A	NRW 19-13-8_Dup(+)	2013	-0.9335	0.6656	1.0960
Pestivirus B	MH231129	VIPR_ALG4_AZQ0065	2a	NS4A	5912c	1995	-0.9335	0.6656	1.0960
Pestivirus B	MH806438	VIPR_ALG4_AZP5716	2a	NS4A	McCart_c	1989	-0.9335	0.6656	1.0960
Pestivirus B	MH806434	VIPR_ALG4_AZP5716	2a	NS4A	125c	1990	-0.6216	0.7019	1.1070

Species according to VIPRBRC	GenBank Accession	GenBank Protein Accession	Subgenotype	Protein	Strain Name	Collection Year	SVM Patho. Score	Vaxijen Antig. Score	Averged score of EMBOSS motifs
Pestivirus B	MH231133	VIPR_ALG4_AZQ0065	2e	NS4A	B69519c	2006	-0.9335	0.6656	1.0960
Pestivirus B	MH806436	VIPR_ALG4_AZP5716	2a	NS4A	296c	1995	-0.9335	0.6656	1.0960
Pestivirus B	MH231124	VIPR_ALG4_AZQ0064	2e	NS4A	1786c	1989	-0.9335	0.6656	1.0960
Pestivirus B	MH231127	VIPR_ALG4_AZQ0065	2a	NS4A	53637c	2004	-0.9335	0.6656	1.0960
Pestivirus B	KX170542	APT71065.1	2a	NS4B	V047	2009	0.0051	0.3162	1.1404
Pestivirus B	KX170543	APT71066.1	2a	NS4B	V063	2005	-0.0048	0.3045	1.1317
Pestivirus B	KX170544	APT71067.1	2a	NS4B	V085	2007	-0.0032	0.3022	1.1357
Pestivirus B	KX170540	APT71063.1	2a	NS4B	V086	2006	0.0338	0.2946	1.1327
Pestivirus B	KX170541	APT71064.1	2a	NS4B	V089	2004	0.0603	0.3050	1.1364
Pestivirus B	KX170539	APT71062.1	2a	NS4B	V095	1999	0.0572	0.2951	1.1401
Pestivirus B	KX170538	APT71061.1	2a	NS4B	V065	2006	0.2043	0.2884	1.1428
Pestivirus B	KX838370	VIPR_ALG4_AOS5274	2a	NS4B	Ind141353	2007	0.0847	0.3001	1.1348
Pestivirus B	KT875139	VIPR_ALG4_10037029	2a	NS4B	32W	2005	0.0095	0.3029	1.1423
Pestivirus B	MN824468	VIPR_ALG4_QJF1227	2a	NS4B	CPAE_contamination/2018	2018	0.1288	0.2894	1.1379
Pestivirus B	KT875134	VIPR_ALG4_10037029	2a	NS4B	12W	2005	0.0095	0.3029	1.1423
Pestivirus B	KT875135	VIPR_ALG4_10037029	2a	NS4B	13Y	2005	-0.0657	0.3032	1.1423
Pestivirus B	KT875136	VIPR_ALG4_10037029	2a	NS4B	27Y	2005	-0.0657	0.3032	1.1423
Pestivirus B	KT875137	VIPR_ALG4_10037029	2a	NS4B	29Y	2005	0.0095	0.3029	1.1423
Pestivirus B	KT875138	VIPR_ALG4_10037029	2a	NS4B	2Y	2005	-0.0657	0.3032	1.1423
Pestivirus B	KT875140	VIPR_ALG4_10037029	2a	NS4B	34Y	2005	0.0095	0.3029	1.1423
Pestivirus B	KT875141	VIPR_ALG4_10037029	2a	NS4B	36W	2005	-0.0657	0.3032	1.1423
Pestivirus B	KT875142	VIPR_ALG4_10037029	2a	NS4B	41Y	2005	-0.0657	0.3032	1.1423
Pestivirus B	KT875143	VIPR_ALG4_10037029	2a	NS4B	42W	2005	-0.0657	0.3032	1.1423
Pestivirus B	KT875144	VIPR_ALG4_10037029	2a	NS4B	43Y	2005	-0.0657	0.3032	1.1423
Pestivirus B	KT875145	VIPR_ALG4_10037029	2a	NS4B	47Y	2005	-0.0657	0.3032	1.1423
Pestivirus B	KT875146	VIPR_ALG4_10037029	2a	NS4B	50Y	2005	-0.0657	0.3032	1.1423
Pestivirus B	KT875147	VIPR_ALG4_10037029	2a	NS4B	51W	2005	0.0095	0.3029	1.1423
Pestivirus B	KT875148	VIPR_ALG4_10037029	2a	NS4B	51Y	2005	0.0095	0.3029	1.1423
Pestivirus B	KT875149	VIPR_ALG4_10037029	2a	NS4B	53W	2005	0.0095	0.3029	1.1423
Pestivirus B	KT875150	VIPR_ALG4_10037029	2a	NS4B	58W	2005	-0.0657	0.3032	1.1423
Pestivirus B	KT875151	VIPR_ALG4_10037029	2a	NS4B	58Y	2005	-0.0657	0.3032	1.1423
Pestivirus B	KT875152	VIPR_ALG4_10037029	2a	NS4B	5Y	2005	0.0095	0.3029	1.1423
Pestivirus B	KT875153	VIPR_ALG4_10037029	2a	NS4B	62Y	2005	-0.0657	0.3032	1.1423
Pestivirus B	KT875154	VIPR_ALG4_10037029	2a	NS4B	65Y	2005	0.0095	0.3029	1.1423
Pestivirus B	KT875155	VIPR_ALG4_10037029	2a	NS4B	67Y	2005	0.0095	0.3029	1.1423
Pestivirus B	KT875156	VIPR_ALG4_10037029	2a	NS4B	68W	2005	-0.0657	0.3032	1.1423
Pestivirus B	KT875157	VIPR_ALG4_10037029	2a	NS4B	71Y	2005	0.0095	0.3029	1.1423
Pestivirus B	KT875158	VIPR_ALG4_10037029	2a	NS4B	73Y	2005	-0.0657	0.3032	1.1423
Pestivirus B	KT875159	VIPR_ALG4_10037029	2a	NS4B	74Y	2005	0.0095	0.3029	1.1423
Pestivirus B	KT875160	VIPR_ALG4_10037029	2a	NS4B	75W	2005	0.0095	0.3029	1.1423
Pestivirus B	KT875161	VIPR_ALG4_10037029	2a	NS4B	75Y	2005	-0.0363	0.2937	1.1412
Pestivirus B	KT875162	VIPR_ALG4_10037029	2a	NS4B	76Y	2005	-0.0657	0.3032	1.1423
Pestivirus B	KT875163	VIPR_ALG4_10037029	2a	NS4B	78W	2005	-0.0657	0.3032	1.1423
Pestivirus B	KT875164	VIPR_ALG4_10037029	2a	NS4B	79W	2005	-0.0657	0.3032	1.1423
Pestivirus B	KT875165	VIPR_ALG4_10037029	2a	NS4B	7W	2005	-0.0657	0.3032	1.1423
Pestivirus B	KT875166	VIPR_ALG4_10037029	2a	NS4B	82W	2005	0.0095	0.3029	1.1423
Pestivirus B	KT875167	VIPR_ALG4_10037029	2a	NS4B	83Y	2005	-0.0657	0.3032	1.1423

Species according to VIPRBRC	GenBank Accession	GenBank Protein Accession	Subgenotype	Protein	Strain Name	Collection Year	SVM Patho. Score	Vaxijen Antig. Score	Averged score of EMBOSS motifs
Pestivirus B	KT875168	VIPR_ALG4_10037029	2a	NS4B	90W	2005	-0.0657	0.3032	1.1423
Pestivirus B	KT875169	VIPR_ALG4_10037030	2a	NS4B	91W	2005	-0.0657	0.3032	1.1423
Pestivirus B	KP057803	VIPR_ALG4_80287536	2a	NS4B	24515	1993	0.1694	0.2849	1.1399
Pestivirus B	KR093034	VIPR_ALG4_92904888	2a	NS4B	NY-93	1993	0.1694	0.2849	1.1399
Pestivirus B	MW006485	VIPR_ALG4_QPF4972	2b	NS4B	HEN01	2014	0.0810	0.3035	1.1354
Pestivirus B	MH231142	VIPR_ALG4_AZQ0066	2c	NS4B	Parker	1991	-0.0151	0.2754	1.1376
Pestivirus B	MH806435	VIPR_ALG4_AZP5716	2a	NS4B	1336H	2005	0.1321	0.2837	1.1358
Pestivirus B	MH231136	VIPR_ALG4_AZQ0066	2a	NS4B	JV14	1998	0.1302	0.2963	1.1386
Pestivirus B	MK599227	VIPR_ALG4_QEU5262	2a	NS4B	SD-1	2016	-0.0075	0.2893	1.1456
Pestivirus B	HG426487	VIPR_ALG4_61932574	2c	NS4B	NRW 19-13-1_Dup(-)	2013	-0.0495	0.2758	1.1376
Pestivirus B	MH231138	VIPR_ALG4_AZQ0066	2a	NS4B	MnFetus	1991	0.0181	0.2808	1.1354
Pestivirus B	MH231131	VIPR_ALG4_AZQ0065	2a	NS4B	AU501	2006	0.0880	0.3019	1.1371
Pestivirus B	MH231151	VIPR_ALG4_AZQ0067	2e	NS4B	14622	2005	-0.0580	0.2834	1.1367
Pestivirus B	MH231152	VIPR_ALG4_AZQ0067	2e	NS4B	2412	1989	-0.0716	0.2831	1.1367
Pestivirus B	MH231149	VIPR_ALG4_AZQ0067	2e	NS4B	Short	1989	-0.0580	0.2834	1.1367
Pestivirus B	KJ000672	VIPR_ALG4_59423572	2b	NS4B	SD1301	2012	0.1287	0.3064	1.1354
Pestivirus B	MH231148	VIPR_ALG4_AZQ0067	2e	NS4B	12-149150	2012	-0.0325	0.2816	1.1367
Pestivirus B	MH231150	VIPR_ALG4_AZQ0067	2e	NS4B	12-151955-317	2012	-0.1594	0.2848	1.1370
Pestivirus B	HG426495	VIPR_ALG4_61932576	2c	NS4B	VOE 4407	2007	-0.0151	0.2754	1.1376
Pestivirus B	HG426491	VIPR_ALG4_61932575	2c	NS4B	Potsdam 1600	2000	0.1163	0.2736	1.1370
Pestivirus B	MH231141	VIPR_ALG4_AZQ0066	2a	NS4B	PI28	2016	0.2019	0.2925	1.1439
Pestivirus B	HG426494	VIPR_ALG4_61932576	2c	NS4B	SH2210-23	2010	-0.0190	0.2784	1.1414
Pestivirus B	MH231137	VIPR_ALG4_AZQ0066	2a	NS4B	MadSpl	1991	0.3106	0.2990	1.1422
Pestivirus B	KP941585	VIPR_ALG4_80092432	2a	NS4B	USMARC-55476	2014	0.1572	0.2959	1.1371
Pestivirus B	KT832820	VIPR_ALG4_99822633	2a	NS4B	USMARC-60767	2014	0.1173	0.2855	1.1376
Pestivirus B	MH231134	VIPR_ALG4_AZQ0065	2a	NS4B	B9497	1997	-0.0481	0.2903	1.1344
Pestivirus B	MN527354	VIPR_ALG4_QLH0204	2a	NS4B	GS2018	2018	0.0054	0.3026	1.1414
Pestivirus B	MH231144	VIPR_ALG4_AZQ0066	2a	NS4B	Sanderson6319	1992	0.0720	0.2948	1.1375
Pestivirus B	HG426493	VIPR_ALG4_61932576	2c	NS4B	SH2210-17	2010	-0.0201	0.2730	1.1371
Pestivirus B	KT832817	VIPR_ALG4_99822632	2a	NS4B	USMARC-60764	2014	0.0188	0.2991	1.1378
Pestivirus B	KT832822	VIPR_ALG4_99822633	2a	NS4B	USMARC-60779	2014	0.3145	0.2764	1.1384
Pestivirus B	MH231125	VIPR_ALG4_AZQ0064	2a	NS4B	2139	1992	0.2938	0.2817	1.1399
Pestivirus B	KP941582	VIPR_ALG4_80092431	2c	NS4B	USMARC-53873	2014	0.0444	0.2785	1.1364
Pestivirus B	KT832819	VIPR_ALG4_99822632	2a	NS4B	USMARC-60766	2014	0.1324	0.2804	1.1306
Pestivirus B	KT832821	VIPR_ALG4_99822633	2c	NS4B	USMARC-60768	2014	-0.0083	0.2769	1.1376
Pestivirus B	HG426479	VIPR_ALG4_61932573	2c	NS4B	D37-13-2_Dup(-)	2013	-0.0495	0.2758	1.1376
Pestivirus B	HG426481	VIPR_ALG4_61932573	2c	NS4B	D75-13-609_Dup(-)	2013	-0.0495	0.2758	1.1376
Pestivirus B	MH231123	VIPR_ALG4_AZQ0064	2a	NS4B	10406	1993	0.2607	0.2608	1.1441
Pestivirus B	HG426492	VIPR_ALG4_61932575	2c	NS4B	SH2210-14	2010	-0.0201	0.2730	1.1371
Pestivirus B	KT832823	VIPR_ALG4_99822633	2a	NS4B	USMARC-60780	2014	-0.0026	0.2918	1.1354
Pestivirus B	HG426485	VIPR_ALG4_61932574	2c	NS4B	NRW 14-13_Dup(-)	2013	-0.0495	0.2758	1.1376
Pestivirus B	MH231126	VIPR_ALG4_AZQ0065	2b	NS4B	3237	1990	0.0368	0.3007	1.1394
Pestivirus B	KT832818	VIPR_ALG4_99822632	2a	NS4B	USMARC-60765	2014	0.0842	0.2893	1.1440
Pestivirus B	HG426483	VIPR_ALG4_61932574	2c	NS4B	NRW 12-13_Dup(-)	2013	-0.0495	0.2758	1.1376
Pestivirus B	HG426489	VIPR_ALG4_61932575	2c	NS4B	NRW 19-13-8_Dup(-)	2013	-0.0495	0.2758	1.1376
Pestivirus B	MH231130	VIPR_ALG4_AZQ0065	2a	NS4B	95-1501	1998	0.0480	0.3063	1.1371
Pestivirus B	MH231132	VIPR_ALG4_AZQ0065	2a	NS4B	AzSpl	1997	0.0164	0.2875	1.1422

Species according to VIPRBRC	GenBank Accession	GenBank Protein Accession	Subgenotype	Protein	Strain Name	Collection Year	SVM Patho. Score	Vaxijen Antig. Score	Averged score of EMBOSS motifs
Pestivirus B	MH231128	VIPR_ALG4_AZQ0065	2a	NS4B	570152	1992	0.1985	0.2814	1.1384
Pestivirus B	MH231135	VIPR_ALG4_AZQ0065	2a	NS4B	BV1907	1995	0.1915	0.2845	1.1392
Pestivirus B	JF714967	VIPR_ALG4_34657797	2a	NS4B	HLJ-10	2011	0.0457	0.2833	1.1385
Pestivirus B	MH231139	VIPR_ALG4_AZQ0066	2a	NS4B	Olwein #12	1990	0.0083	0.2983	1.1331
Pestivirus B	MH231140	VIPR_ALG4_AZQ0066	2a	NS4B	PA	1992	0.0737	0.2985	1.1383
Pestivirus B	MH231143	VIPR_ALG4_AZQ0066	2a	NS4B	RS886	2014	0.1978	0.2944	1.1369
Pestivirus B	HQ258810	VIPR_ALG4_31199027	2a	NS4B	SH-28	2009	0.0544	0.2755	1.1377
Pestivirus B	MH231145	VIPR_ALG4_AZQ0066	2a	NS4B	Victor301	1990	0.1578	0.2941	1.1361
Pestivirus B	MH231146	VIPR_ALG4_AZQ0067	2a	NS4B	WiscA	1991	0.1701	0.2818	1.1392
Pestivirus B	FJ527854	VIPR_ALG4_22961018	2a	NS4B	XJ-04	2004	-0.0404	0.2908	1.1378
Pestivirus B	KC963968	VIPR_ALG4_53029119	2a	NS4B	11F011	2011	0.1928	0.3018	1.1299
Pestivirus B	MH806437	VIPR_ALG4_AZP5716	2a	NS4B	9231	2004	-0.0599	0.3025	1.1357
Pestivirus B	MG879027	VIPR_ALG4_AVA3071	2a	NS4B	CN10.2015.821	2014	0.1325	0.2991	1.1377
Pestivirus B	KX096718	VIPR_ALG4_11130170	2a	NS4B	HB-1511	2015	0.1605	0.2938	1.1413
Pestivirus B	LC649064	VIPR_ALG4_BDB0736	2c	NS4B	KZ-91-NCP	1991	-0.0151	0.2754	1.1376
Pestivirus B	MH231147	VIPR_ALG4_AZQ0067	2c	NS4B	PI12	2016	0.0078	0.2714	1.1376
Pestivirus B	GQ888686	VIPR_ALG4_27071949	2a	NS4B	JZ05-1	2005	0.0877	0.2948	1.1396
Pestivirus B	MW168422	VIPR_ALG4_QZM0693	2a	NS4B	YNJG2020	2020	0.1293	0.2988	1.1340
Pestivirus B	HG426488	VIPR_ALG4_61932575	2c	NS4B	NRW 19-13-1_Dup(+)	2013	-0.0495	0.2758	1.1376
Pestivirus B	HG426480	VIPR_ALG4_61932573	2c	NS4B	D37-13-2_Dup(+)	2013	-0.0495	0.2758	1.1376
Pestivirus B	HG426482	VIPR_ALG4_61932573	2c	NS4B	D75-13-609_Dup(+)	2013	-0.0495	0.2758	1.1376
Pestivirus B	HG426486	VIPR_ALG4_61932574	2c	NS4B	NRW 14-13_Dup(+)	2013	-0.0495	0.2758	1.1376
Pestivirus B	HG426484	VIPR_ALG4_61932574	2c	NS4B	NRW 12-13_Dup(+)	2013	-0.0495	0.2758	1.1376
Pestivirus B	HG426490	VIPR_ALG4_61932575	2c	NS4B	NRW 19-13-8_Dup(+)	2013	-0.0495	0.2758	1.1376
Pestivirus B	MH231129	VIPR_ALG4_AZQ0065	2a	NS4B	5912c	1995	-0.0107	0.2921	1.1379
Pestivirus B	MH806438	VIPR_ALG4_AZP5716	2a	NS4B	McCart_c	1989	-0.0244	0.3021	1.1339
Pestivirus B	MH806434	VIPR_ALG4_AZP5716	2a	NS4B	125c	1990	0.1022	0.2931	1.1379
Pestivirus B	MH231133	VIPR_ALG4_AZQ0065	2e	NS4B	B69519c	2006	-0.0716	0.2831	1.1367
Pestivirus B	MH806436	VIPR_ALG4_AZP5716	2a	NS4B	296c	1995	-0.0107	0.2921	1.1379
Pestivirus B	MH231124	VIPR_ALG4_AZQ0064	2e	NS4B	1786c	1989	-0.0716	0.2831	1.1367
Pestivirus B	MH231127	VIPR_ALG4_AZQ0065	2a	NS4B	53637c	2004	0.1755	0.2918	1.1391
