# Supplementary material for: Immunoinformatic prediction of the pathogenicity of bovine viral diarrhea virus genotypes: implications for viral virulence determinants, designing novel diagnostic assays and vaccines development
Source: Front Vet Sci. 2023 Jul 6;10:1130147. doi: 10.3389/fvets.2023.1130147 (PMC10359904; doi:10.3389/fvets.2023.1130147)
Supplement: Supplementary file 4 [file Table_3.pdf]

Supp. table 3: Count of involved sequences and averaged predicted pathogenicity and antigenicity scores at level of subgenotypes and proteins

I-Count of the involved protein sequences used in the present study for the BVDV subgenotypes and proteins

Protein\Subgenotype	1a	1b	1c	1d	1e	1f	1g	1h	1i	1j	1k	1l	1m	1n	1o	1p	1q	1r	1u	1v	BVDV1	2a	2b	2c	2e	BVDV2
Npro	66	172	8	13	8	6	1	6	5	3	7	4	16	1	15	10	16	1	2	1	361	115	6	22	7	150
C	64	114	5	6	8	6	1	5	5	1	7	none	5	1	1	none	6	1	1	none	237	113	4	22	7	146
Erns	65	119	6	10	8	6	1	5	5	1	7	none	18	1	2	5	11	1	1	2	274	108	4	22	7	141
E1	63	111	5	6	8	6	1	5	5	1	7	none	5	1	1	none	6	1	1	none	233	108	4	22	7	141
E2	78	273	8	7	8	6	1	5	5	1	7	none	7	1	2	3	7	1	1	none	421	114	4	47	7	172
P7	25	93	4	5	8	6	1	5	5	1	7	none	4	1	1	none	4	1	1	none	172	82	3	22	7	114
NS2	56	104	4	5	8	6	1	5	4	1	7	none	4	1	1	none	4	1	1	none	213	82	3	22	7	114
NS3	59	106	4	5	8	6	1	5	5	1	7	none	4	1	1	none	4	1	1	none	219	87	3	22	7	119
NS4A	60	106	4	5	8	6	1	5	5	1	7	none	4	1	1	none	4	1	1	none	220	95	3	22	7	127
NS4B	58	106	4	5	8	6	1	5	5	1	7	none	4	1	1	none	4	1	none	none	217	89	3	22	7	121
NS5A	58	106	4	5	8	6	1	5	5	1	7	none	4	1	1	none	4	1	none	none	217	85	3	22	7	117
NS5B	56	106	4	5	8	6	1	5	5	1	7	none	4	1	1	none	4	1	none	none	215	90	3	22	7	122
Total count of seq.	708	1516	60	77	96	72	12	61	59	14	84	4	79	12	28	18	74	12	10	3	2999	1168	43	289	84	1584
Count of seq. of pathogenic proteins	182	326	13	16	24	18	3	15	15	3	21	0	13	3	3	0	14	3	2	0	674	286	12	66	21	385
Count of seq. of antigenic proteins	559	1145	44	54	72	54	9	45	44	9	63	0	55	9	11	8	50	9	7	2	2249	882	31	223	63	1199

II-Average of the predicted pathogenicity scores for BVDV subgenotypes and proteins (values higher than threshold are red highlighted), (pathogenic proteins: C, NS4A, NS4B for BVDV1; Npro, P7 and NS4B for BVDV2)

Protein\Subgenotype	1a	1b	1c	1d	1e	1f	1g	1h	1i	1j	1k	1l	1m	1n	1o	1p	1q	1r	1u	1v	BVDV1	2a	2b	2c	2e	BVDV2
Npro	-0.724	-0.67	-0.656	-0.524	-0.565	-0.732	-0.769	-0.678	-0.928	-0.937	-0.771	-0.792	-0.605	-0.779	-0.478	-0.74	-0.765	-0.572	-0.596	-0.564	-0.677	-0.199	-0.398	0.194	0.2	-0.131
C	-0.414	0.294	-0.122	-0.108	0.04	-0.369	0.197	0.195	-0.267	-0.199	-0.156		-0.195	-0.32	-0.068		-0.017	0.261	0.2		0.006	-0.356	-0.637	-0.108	-0.036	-0.311
Erns	-0.463	-0.446	-0.466	-0.249	-0.524	-0.261	-0.105	-0.261	-0.36	-0.32	-0.332		-0.198	-0.644	-0.189	-0.131	-0.251	-0.389	-0.075	-0.113	-0.397	-0.372	-0.315	-0.211	-0.42	-0.348
E1	-0.816	-0.78	-0.309	-0.706	-0.561	-0.279	-0.579	-0.718	-0.525	-0.112	-0.958		-0.422	-0.672	-0.308		-0.561	-0.307	-0.286		-0.732	-0.878	-0.776	-0.719	-1.09	-0.861
E2	-0.652	-0.473	-0.652	-0.477	-0.782	-0.662	-0.977	-0.691	-0.741	-0.837	-0.59		-0.817	-0.29	-0.899	-0.726	-0.809	-0.711	-0.527		-0.543	-0.533	-0.525	-0.515	-0.226	-0.515
P7	-1.04	-1.071	-1.539	-0.672	-0.491	-1.515	-1.987	-2.232	-1.08	-0.898	-1.9		-1.133	0.056	-1.733		-0.862	-2.416	-0.933		-1.127	-0.249	0.078	0.517	-0.51	-0.108
NS2	-0.404	-0.525	-0.482	-0.672	-0.387	-0.878	-1.011	-0.844	-0.544	-0.602	-0.544		-0.486	-0.852	-0.824		-0.768	-0.839	-0.628		-0.52	-0.862	-0.847	-0.741	-0.645	-0.825
NS3	-0.521	-0.561	-0.501	-0.642	-0.517	-0.581	-0.514	-0.63	-0.515	-0.549	-0.599		-0.634	-0.57	-0.667		-0.54	-0.586	-0.614		-0.553	-0.525	-0.574	-0.502	-0.489	-0.52
NS4A	0.168	-0.313	0.033	0.053	-0.211	-0.375	-0.25	-0.295	0.087	-0.005	-0.381		-0.038	-0.475	0.002		-0.222	-0.295	1.017		-0.143	-0.878	-0.909	-0.923	-0.933	-0.89
NS4B	-0.232	0.024	-0.184	-0.295	-0.232	-0.088	-0.034	-0.264	-0.278	-0.509	-0.244		-0.375	-0.052	-0.265		-0.197	-0.214			-0.108	0.044	0.082	-0.024	-0.075	0.025
NS5A	-0.813	-0.838	-0.643	-0.975	-0.89	-0.701	-0.686	-0.719	-0.755	-1.123	-0.428		-0.605	-0.789	-0.428		-0.767	-0.379			-0.802	-0.854	-0.685	-0.866	-0.9	-0.855
NS5B	-0.411	-0.303	-0.312	-0.307	-0.298	-0.355	-0.47	-0.443	-0.455	-0.3	-0.238		-0.303	-0.177	-0.446		-0.399	-0.364			-0.34	-0.598	-0.616	-0.641	-0.65	-0.61
Overall average	-0.508	-0.472	-0.499	-0.456	-0.452	-0.566	-0.599	-0.632	-0.53	-0.59	-0.595	-0.792	-0.46	-0.464	-0.503	-0.568	-0.529	-0.567	-0.304	-0.263	-0.493	-0.515	-0.507	-0.39	-0.481	-0.49
Average of pathogenic proteins	-0.164	0.009	-0.093	-0.116	-0.134	-0.277	-0.029	-0.121	-0.153	-0.238	-0.26		-0.202	-0.282	-0.111		-0.127	-0.082	0.608		-0.079	-0.138	-0.159	0.229	-0.128	-0.075

III-Average of the predicted antigenicity scores for BVDV subgenotypes and proteins (values higher than threshold are green highlighted), (antigenic proteins: C, Erns, E1, E2, NS2, NS3, NS4A, NS5A, NS5B)

Protein\Subgenotype	1a	1b	1c	1d	1e	1f	1g	1h	1i	1j	1k	1l	1m	1n	1o	1p	1q	1r	1u	1v	BVDV1	2a	2b	2c	2e	BVDV2
Npro	0.332	0.295	0.31	0.347	0.297	0.297	0.324	0.309	0.22	0.3	0.347	0.297	0.368	0.314	0.331	0.289	0.294	0.267	0.374	0.224	0.309	0.335	0.407	0.29	0.278	0.328
C	0.593	0.589	0.596	0.591	0.545	0.684	0.615	0.591	0.52	0.585	0.575		0.651	0.548	0.67		0.641	0.703	0.479		0.592	0.55	0.561	0.592	0.504	0.554
Erns	0.524	0.493	0.509	0.538	0.496	0.513	0.444	0.505	0.458	0.508	0.553		0.496	0.514	0.476	0.491	0.494	0.475	0.51	0.499	0.504	0.533	0.543	0.526	0.527	0.532
E1	0.512	0.464	0.512	0.542	0.508	0.432	0.478	0.507	0.493	0.469	0.507		0.519	0.487	0.473		0.534	0.484	0.499		0.487	0.481	0.551	0.482	0.492	0.484
E2	0.641	0.568	0.631	0.628	0.642	0.592	0.643	0.58	0.632	0.599	0.619		0.616	0.587	0.628	0.651	0.599	0.61	0.548		0.59	0.502	0.492	0.465	0.525	0.492
P7	0.175	0.211	0.151	0.145	0.227	0.218	0.153	0.29	0.119	0.203	0.147		0.199	0.112	0.116		0.187	0.082	0.174		0.197	0.203	0.279	0.263	0.353	0.226
NS2	0.574	0.633	0.577	0.551	0.59	0.589	0.579	0.561	0.586	0.588	0.576		0.6	0.504	0.61		0.593	0.575	0.486		0.603	0.587	0.577	0.579	0.565	0.584
NS3	0.585	0.585	0.59	0.59	0.589	0.602	0.597	0.599	0.592	0.588	0.594		0.593	0.59	0.587		0.592	0.597	0.587		0.587	0.593	0.591	0.59	0.596	0.593
NS4A	0.694	0.707	0.703	0.699	0.737	0.709	0.74	0.708	0.636	0.694	0.71		0.725	0.715	0.717		0.71	0.708	0.602		0.703	0.665	0.669	0.666	0.666	0.665
NS4B	0.301	0.299	0.314	0.29	0.299	0.302	0.316	0.29	0.299	0.277	0.314		0.3	0.293	0.287		0.302	0.309			0.3	0.297	0.304	0.275	0.283	0.292
NS5A	0.52	0.541	0.555	0.48	0.561	0.564	0.536	0.529	0.526	0.571	0.56		0.51	0.509	0.519		0.517	0.568			0.535	0.586	0.555	0.607	0.579	0.589
NS5B	0.533	0.511	0.531	0.52	0.516	0.494	0.496	0.497	0.512	0.512	0.506		0.471	0.497	0.482		0.503	0.494			0.515	0.466	0.451	0.473	0.453	0.466
Overall average	0.517	0.494	0.497	0.487	0.5	0.5	0.493	0.494	0.464	0.464	0.501	0.297	0.488	0.472	0.415	0.406	0.473	0.489	0.463	0.407	0.497	0.484	0.496	0.483	0.485	0.484
Average of antigenic proteins	0.577	0.565	0.579	0.57	0.576	0.575	0.57	0.564	0.55	0.568	0.578		0.558	0.55	0.57	0.551	0.567	0.579	0.53	0.499	0.569	0.548	0.552	0.544	0.545	0.547
